# Identifying the World's Most Climate Change Vulnerable Species: A Systematic Trait-Based Assessment of all Birds, Amphibians and Corals

**DOI:** 10.1371/journal.pone.0065427

**Published:** 2013-06-12

**Authors:** Wendy B. Foden, Stuart H. M. Butchart, Simon N. Stuart, Jean-Christophe Vié, H. Resit Akçakaya, Ariadne Angulo, Lyndon M. DeVantier, Alexander Gutsche, Emre Turak, Long Cao, Simon D. Donner, Vineet Katariya, Rodolphe Bernard, Robert A. Holland, Adrian F. Hughes, Susannah E. O’Hanlon, Stephen T. Garnett, Çagan H. Şekercioğlu, Georgina M. Mace

**Affiliations:** 1 Global Species Programme, International Union for Conservation of Nature, Cambridge, United Kingdom; 2 Animal, Plant and Environmental Sciences, University of the Witwatersrand, Johannesburg, South Africa; 3 Science, BirdLife International, Cambridge, United Kingdom; 4 Species Survival Commission, International Union for Conservation of Nature, Gland, Switzerland; 5 United Nations Environment Programme World Conservation Monitoring Centre, Cambridge, United Kingdom; 6 Al Ain Wildlife Park and Resort, Al Ain, United Arab Emirates; 7 Department of Biology and Biochemistry, University of Bath, Bath, Somerset, United Kingdom; 8 Global Species Programme, International Union for Conservation of Nature, Gland, Switzerland; 9 Department of Ecology and Evolution, Stony Brook University, Stony Brook, New York, United States of America; 10 Noosaville, Queensland, Australia; 11 Museum für Naturkunde, Leibniz Institute for Research on Evolution and Biodiversity, Berlin, Germany; 12 Cormec, Cergy, France; 13 Catchment to Reef Research Group, Centre for Tropical Water and Aquatic Ecosystem Research, James Cook University, Townsville, Queensland, Australia; 14 Department of Earth Sciences, ZheJiang University, Hangzhou, China; 15 Department of Geography, University of British Columbia, Vancouver, British Columbia, Canada; 16 Centre for Population Biology, Imperial College London, Ascot, Berkshire, United Kingdom; 17 Centre for Biological Sciences, University of Southampton, Southampton, United Kingdom; 18 Department of Life Sciences, Anglia Ruskin University, Cambridge, United Kingdom; 19 Research Institute for Environment and Livelihoods, Charles Darwin University, Darwin, Northern Territory, Australia; 20 Department of Biology, University of Utah, Salt Lake City, Utah, United States of America; 21 Centre for Biodiversity & Environment Research, Department of Genetics, Evolution and Environment, University College London, London, United Kingdom; CNRS/Université Joseph-Fourier, France

## Abstract

Climate change will have far-reaching impacts on biodiversity, including increasing extinction rates. Current approaches to quantifying such impacts focus on measuring exposure to climatic change and largely ignore the biological differences between species that may significantly increase or reduce their vulnerability. To address this, we present a framework for assessing three dimensions of climate change vulnerability, namely sensitivity, exposure and adaptive capacity; this draws on species’ biological traits and their modeled exposure to projected climatic changes. In the largest such assessment to date, we applied this approach to each of the world’s birds, amphibians and corals (16,857 species). The resulting assessments identify the species with greatest relative vulnerability to climate change and the geographic areas in which they are concentrated, including the Amazon basin for amphibians and birds, and the central Indo-west Pacific (Coral Triangle) for corals. We found that high concentration areas for species with traits conferring highest sensitivity and lowest adaptive capacity differ from those of highly exposed species, and we identify areas where exposure-based assessments alone may over or under-estimate climate change impacts. We found that 608–851 bird (6–9%), 670–933 amphibian (11–15%), and 47–73 coral species (6–9%) are both highly climate change vulnerable and already threatened with extinction on the IUCN Red List. The remaining highly climate change vulnerable species represent new priorities for conservation. Fewer species are highly climate change vulnerable under lower IPCC SRES emissions scenarios, indicating that reducing greenhouse emissions will reduce climate change driven extinctions. Our study answers the growing call for a more biologically and ecologically inclusive approach to assessing climate change vulnerability. By facilitating independent assessment of the three dimensions of climate change vulnerability, our approach can be used to devise species and area-specific conservation interventions and indices. The priorities we identify will strengthen global strategies to mitigate climate change impacts.

## Introduction

Vertebrate extinction rates are currently estimated to be 10–100 times greater than background [1], largely due to the effects of habitat loss, over-exploitation and invasive species [2], [3]. However, anthropogenic climate change is becoming a significant new threat [4]–[7]. Based on regional studies, the Intergovernmental Panel on Climate Change (IPCC) estimated that 20–30% of the world’s species are likely to be at increasingly high risk of extinction from climate change impacts within this century if global mean temperatures exceed 2–3°C above pre-industrial levels [6], while Thomas *et al.* [5] predicted that 15–37% of species could be ‘committed to extinction’ due to climate change by 2050. These high rates of potential local and global extinction have stimulated urgent calls for proactive conservation planning [8] and there is a pressing need to identify the most climate change vulnerable species, habitats and regions.

Until now, the IPCC and Thomas *et al.* studies have been the only global-scale assessments of potential climate change impacts on species that cover multiple taxonomic groups. These and most other similar large-scale assessments are based primarily on species distribution (bioclimatic envelope) models, which use correlations between species’ observed distributions and climate variables to predict their distributions and hence their extinction risk under future climate scenarios [9]–[11]. Such models focus on changes in the distribution or extent of species’ ‘climate space’, but the broad range of climate-change-related stresses that affect population ecology and physiology and that may have consequences at ecosystem and community levels [12] are not fully reflected. A growing number of studies show that observed climate-change-induced stresses are mediated by species’ biological traits [13], [14]. Incorporating species’ physiological, ecological and evolutionary characteristics, in conjunction with their predicted climate change exposure, will therefore facilitate more accurate identification of the species most at risk from climate change [15]–[17].

**Figure 1:**
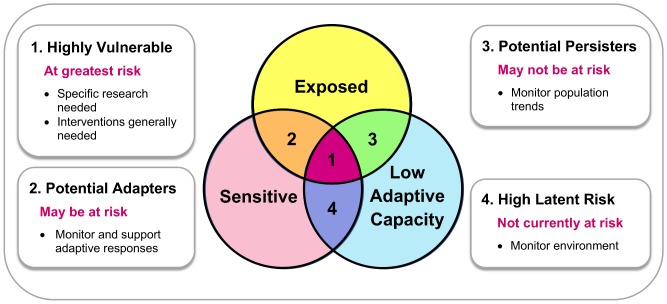
Framework to assess the impacts of climate change on species. Combinations of the three dimensions of climate change vulnerability, namely sensitivity, exposure and low adaptive capacity describe four distinct classes of climate change vulnerable species, each with particular implications for conservation prioritisation and strategic planning. Species that are ‘highly climate change vulnerable’ (1), being sensitive, exposed and of low adaptive capacity, are of greatest concern. They are the first priority for monitoring responses to climate change and for assessment of the interventions needed to support them. ‘Potential adapters’ (2) are sensitive and exposed (but high adaptive capacity) species that may be able to mitigate negative climate change impacts by dispersal or microevolution, although close monitoring is needed to verify this. ‘Potential persisters’ (3) have low adaptive capacity and are exposed (but are not sensitive) so may be able to withstand climate change *in situ* by themselves, but again, monitoring is needed to ensure that the assumptions about insensitivity are realized in practice. Finally, species of ‘high latent risk’ (4) have low adaptive capacity and are sensitive (but are not exposed). Although not of immediate concern if climate change projections and emissions scenarios are accurate, they could become climate change vulnerable if exposed beyond selected time frames (e.g., 2050).

We developed a framework for identifying the species most vulnerable to extinction from a range of climate change induced stresses. The framework guides users to independently measure three dimensions of climate change vulnerability, namely sensitivity (the lack of potential for a species to persist *in situ)*, exposure (the extent to which each species’ physical environment will change) and low adaptive capacity (a species’ inability to avoid the negative impacts of climate change through dispersal and/or micro-evolutionary change). The three dimensions can then be used to allocate species to one of four classes of climate change vulnerability, each with different implications for conservation (Figure 1). Species are considered to be highly climate change vulnerable if they qualify as highly sensitive, highly exposed and of lowest adaptive capacity.

In order to identify the biological traits associated with species’ climate change vulnerability, we held two workshops and undertook a range of consultations with over 30 experts whose collective experience in extinction risk assessment spans a broad range of taxonomic groups, ecosystems and regions. Combined with an extensive literature survey, this process identified more than 90 biological, ecological, physiological and environmental traits likely to influence climate change sensitivity and adaptive capacity in different major taxa. We consolidated these into ‘trait sets’, including five for sensitivity and two for adaptive capacity. Trait sets associated with heightened sensitivity were (a) habitat and/or microhabitat specialization, (b) narrow environmental tolerances, (c) the potential for disruption of both environmental triggers and (d) interspecific interactions, and (e) rarity. Low adaptive capacity trait sets included (f) poor dispersal potential due to low inherent dispersal ability and/or extrinsic barriers to dispersal, and (g) poor micro-evolutionary potential due to low genetic diversity, long generation lengths and/or low reproductive output (Table 1; Methods and Materials; Supporting Methods in Supporting Information S1). Exposure may be estimated by examining the magnitude of projected climatic changes across a species’ distribution, considering parameters relevant to each species group (e.g., changes in temperature, precipitation, sea level and/or hydrological regimes).

**Table 1:** Trait sets associated with species’ heightened sensitivity and low adaptive capacity to climate change.

SENSITIVITY
**a. Specialised habitat and/or microhabitat requirements**As climate change-driven environmental changes unfold, species that are less tightly coupled to specific conditions and requirements are likely to be more resilient because they will have a wider range of habitat and microhabitat options available to them. Sensitivity is further increased for species with several life stages, each requiring different habitats or microhabitats (e.g., water-dependent larval amphibians). We note, however, that this does not hold in all cases, and extreme specialization may allow some species to escape the full impacts of climate change exposure (e.g., deep sea fishes).
**b. Environmental tolerances or thresholds (at any life stage) that are likely to be exceeded due to climate change**Species with physiological tolerances that are tightly coupled to specific environmental conditions (e.g., temperature or precipitation regimes, water pH or oxygen levels) are likely to be particularly sensitive to climatic changes (e.g., tropical ectotherms) [37], [38]. However, even species with broad environmental tolerances may already be close to thresholds beyond which physiological function quickly breaks down (e.g., drought tolerant desert plants [39]).
**c. Dependence on environmental triggers that are likely to be disrupted by climate change**Many species rely on environmental triggers or cues to initiate life stages (e.g., migration, breeding, egg laying, seed germination, hibernation and spring emergence). While cues such as day length and lunar cycles will be unaffected by climate change, those driven by climate and season may alter in both their timing and magnitude, leading to asynchrony and uncoupling with environmental factors [40] (e.g., mismatches between advancing spring food availability peaks and hatching dates [41]). Climate change sensitivity is likely to be compounded when different sexes or life stages rely on different cues.
**d. Dependence on interspecific interactions that are likely to be disrupted by climate change**Climate change driven alterations in species’ ranges, phenologies and relative abundances may affect their beneficial inter-specific interactions (e.g., with prey, pollinators, hosts and symbionts) and/or those that may cause declines (e.g., with predators, competitors, pathogens and parasites). Species are likely to be particularly sensitive to climate change if, for example, they are highly dependent on one or few specific resource species and are unlikely to be able to substitute these for other species [42].
**e. Rarity**The inherent vulnerability of small populations to Allee effects and catastrophic events, as well as their generally reduced capacity to recover quickly following local extinction events, suggest that many rare species will be more sensitive to climate change than common species. Rare species include those with very small population sizes, as well as those that may be locally abundant but are geographically highly restricted.
**LOW ADAPTIVE CAPACITY**
**f. Poor dispersal ability:** Intrinsic dispersal limitations: Species with low dispersal rates or low potential for long distance dispersal (e.g., land snails, ant and raindrop splash-dispersed plants) have lowest adaptive capacity since they are unlikely to be able to keep up with a shifting climate envelope.Extrinsic dispersal limitations: Even where species are intrinsically capable of long distance or rapid dispersal, movement and/or successful colonisation may be reduced by low permeability or physical barriers along dispersal routes. These include natural barriers (e.g., oceans or rivers for terrestrial species), anthropogenic barriers (e.g., dams for freshwater species) and unsuitable habitats or conditions (e.g., ocean currents and temperature gradients for marine species). Species for which no suitable habitat or ‘climate space’ is likely to remain (e.g., Arctic ice-dependent species) may also be considered in this trait set.
**g. Poor evolvability:**Species’ potential for rapid genetic change will determine whether evolutionary adaptation can result at a rate sufficient to keep up with climate change driven changes to their environments. Species with low genetic diversity, often indicated by recent bottlenecks in population numbers, generally exhibit lower ranges of both phenotypic and genotypic variation. As a result, such species tend to have fewer novel characteristics that could facilitate adaptation to the new climatic conditions.Since direct measures of species’ genetic diversity are few, proxy measures of evolvability such as those relating to reproductive rates and outputs, and hence the rate at which advantageous novel genotypes could accumulate in populations and species [43], may be useful. Evidence suggests that evolutionary adaptation is possible in relatively short timeframes (e.g., 5 to 30 years [44]) but for most species with long generation lengths (e.g., large animals and many perennial plants), this is likely to be too slow to have any serious minimising effect on climate change impacts.

Using this framework, we assessed the climate change vulnerability of each of the world’s birds (9,856 species), amphibians (6,204 species) and warm-water reef-building corals (797 species). These taxonomic groups were selected as they are relatively well-studied and include species from terrestrial, freshwater and marine biomes. Gathering trait data involved extensive literature surveys, data compilation and expert consultation. The taxon-specific traits selected under each trait set are shown in Tables S1, S2, S3. Exposure assessments were based on projected changes in temperature and precipitation (considering both means and variability) for birds and amphibians, and in bleaching frequency and ocean acidification for corals (Methods and Materials; Supporting Methods in Supporting Information S1). Sea level rise was also considered for terrestrial species.

The resulting combinations of ordinal and categorical data posed challenges for assigning the thresholds needed to distinguish relatively high versus low climate change vulnerability (discussed in Methods and Materials and Supporting Discussion in Supporting Information S1). For the many cases where no empirical basis for selecting trait thresholds existed, we set expert-informed thresholds where possible, and for the remainder of traits, classified the worst impacted 25% of species as of highest vulnerability. As result, our findings assess *relative* rather than absolute vulnerability to climate change. They therefore indicate which species are likely to be at greatest risk of climate change driven extinction, but cannot be used to infer how many species will be impacted, nor to compare vulnerability between taxonomic groups.

## Results

We identified the 2,323–4,890 bird species (24–50%), 1,368–2,740 amphibian species (22–44%), and 121–253 coral species (15–32%) that are most vulnerable to climate change (see Table 2; Appendices A, B and C in Supporting Information S1 for results by species; Tables S4, S5, S6, S7 for results by family; Tables S1, S2, S3 for results by trait; lower and upper estimates derived from optimistic and pessimistic assumptions for missing trait data). Although sensitivity, exposure and low adaptive capacity were not completely independent of each other (Figure S1), certain regions contain disproportionate numbers of species qualifying under these vulnerability dimensions (Figures S2, S3, S4, S5).

**Table 2:** Summary of relative climate change vulnerability in birds, amphibians and corals.

Climate Change Vulnerability				Birds				Amphibians				Corals			
Vulnerability type	Sensitivity	Exposure	LowAdaptiveCapacity	Optimistic		Pessimistic		Optimistic		Pessimistic		Optimistic		Pessimistic	
				No.	*%*	No.	*%*	No.	*%*	No.	*%*	No.	*%*	No.	*%*
Highly vulnerable (1)	√	√	√	2,323	*24*	4,890	*50*	1,368	*22*	2,740	*44*	121	*15*	253	*32*
Potential adapters (2)	√	√	–	1,496	*15*	1,565	*16*	1,068	*17*	115	*2*	150	*19*	109	*14*
Potential persisters (3)	–	√	√	493	*5*	214	*2*	523	*8*	643	*10*	0	*0*	0	*0*
High latent risk (4)	√	–	√	1,511	*15*	1,976	*20*	957	*15*	1,663	*27*	299	*38*	257	*32*
Sensitive only	√	–	–	960	*10*	706	*7*	1,060	*17*	321	*5*	226	*28*	177	*22*
Exposed only		√	–	608	*6*	105	*1*	397	*6*	64	*1*	0	*0*	0	*0*
Low adaptive capacity only		–	√	1,010	*10*	269	*3*	385	*6*	537	*9*	0	*0*	0	*0*
None	–	–	–	1,455	*15*	131	*1*	446	*7*	121	*2*	1	*0*	1	*0*
**Total numbers of species**				**9,856**				**6,204**				**797**			

This includes the total numbers and percentages of species in the climate change vulnerability categories highlighted in Figure 1, as well as those in each climate change vulnerability dimension alone. To represent the uncertainty resulting from missing biological trait data, vulnerability was calculated assuming optimistic and pessimistic extremes for missing values. It is important to note that scores represent relative measures within each taxonomic groups and comparisons between groups are not meaningful.

**Figure 2:**
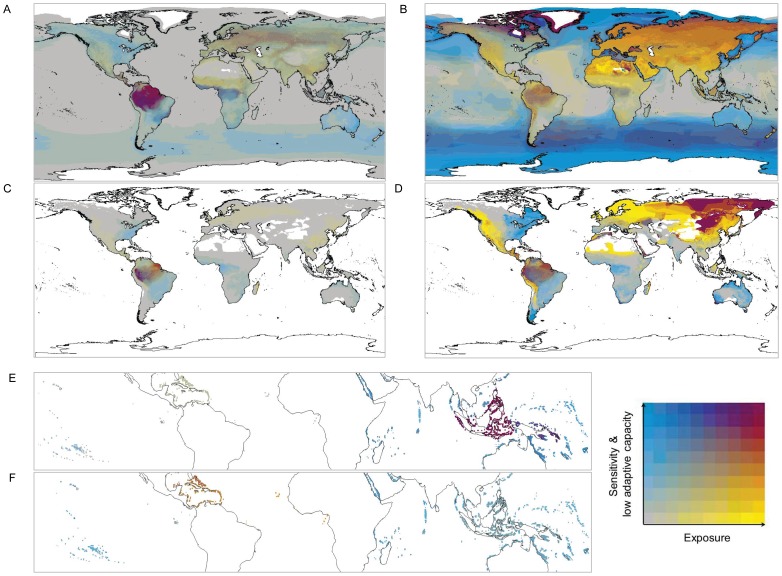
Concentrations of climate change vulnerable species. Areas with greatest concentrations of species with high sensitivity and low adaptive capacity only are shown in blue, and those with high exposure to climatic change only are in yellow. Areas with high concentrations of species that have high sensitivity and low adaptive capacity species, as well as of highly exposed species, are shown in maroon; they correspond with areas of high overall climate change vulnerability. Total numbers of climate change vulnerable birds, amphibians and corals are shown in A, C and E respectively, while B, D and F show the proportions of species occurring in a region that are climate change vulnerable. Grey areas show where species are present, but concentrations of focal species groups are low; colours increase in intensity as total numbers (for A, C and E) and proportions (for B, D and F) of focal species increase. These results were based on the moderate A1B emissions scenario for 2050 and assume an optimistic scenario for missing trait information.

To explore the relationship between climate change vulnerability conferred by biological traits vs. exposure, we present bivariate plots (Figure 2) which highlight areas with greatest concentrations of (i) species that have traits conferring high sensitivity and low adaptive capacity but that are not highly exposed (in blue), (ii) highly exposed species that lack high sensitivity and low adaptability traits (in yellow), and (iii) species that are highly exposed, highly sensitive and have low adaptive capacity (i.e., those of highest climate change vulnerability overall; in maroon). Since both the total numbers of such species in a region and their proportion relative to all species occurring there provide important information for priority-setting and policy, we include both in the results. Areas containing the greatest *numbers* of highly climate change vulnerable birds, amphibians and corals are indicated by the maroon areas in Figure 2A, C and E respectively, while this colour in B, D and F highlights regions with the greatest *proportions* of species that are highly climate change vulnerable.

The Amazon emerges as a region of high climate change vulnerability for both birds and amphibians, due to the large overall numbers and proportions of such species occurring there (Figure 2A–D). For birds, large numbers of highly climate change vulnerable species are also found in Mesoamerica, central Eurasia, the Congo basin, the Himalayas and Sundaland (Malaysia, Indonesia and southern Thailand) (Figure 2A). Large proportions of highly climate change vulnerable bird species also occur in these areas, as well as in north-eastern North America, Greenland and Iceland, the southern oceans and northern Africa (Figure 2B). For amphibians, in addition to the Amazon, high proportions of highly climate change vulnerable species occur in Mesoamerica, the northern Andes, North Africa, eastern Russia to Mongolia, the Himalayas and the western Arabian Peninsula (Figure 2D). Highly climate change vulnerable corals are concentrated in the Coral Triangle, Sumatra and Java (Figure 2E). The proportion of coral species that are of highest climate change vulnerability shows no strong spatial patterns (i.e., no markedly maroon areas), although there is a slight concentration of such species in the Caribbean (Figure 2F). (See Tables S8–S9 for a full description of the maroon areas highlighted in Figure 2).

Regions where species’ relatively low sensitivity and/or higher adaptive capacity may help them to avoid or adjust to climate change impacts, and hence where assessments based on climate change exposure alone may over-estimate extinction risk, are highlighted in yellow in Figure 2. Considering total numbers of species, such regions include southern Asia and western North America for birds and amphibians, the Sahel for birds, and parts of Europe for amphibians (Figure 2A and C). For corals, no such areas emerged (Figure 2 E) (see Tables S8–S9 for a full description of the yellow areas highlighted in all parts of Figure 2). Of those species assessed as highly exposed, we found that 1,844–2,597 bird species (28–53%), 822–1,988 amphibian species (23–59%), and 109–150 coral species (30–55%) do not have the high sensitivity and low adaptability traits that, in combination, would otherwise render them highly climate change vulnerable; these species are therefore ‘potential adapters’ and/or ‘potential persisters’.

Concentrations of highly sensitive species with low adaptive capacity that are not facing high exposure to climate change (i.e., high latent risk species) are indicated by blue areas in Figure 2. Although probably not currently at high risk, these species should be monitored since they could rapidly become so should their climates change more than predicted or if timeframes beyond those considered for this component of the assessment (i.e., 2050) are considered. The highest numbers of such species for both birds and amphibians are found in the Congo basin, eastern North and South America, southern Africa and Australia, with additional high concentration areas for birds in northern North America, oceans south of 30^o^S, northern Eurasia and New Guinea (Figure 2A and C). For corals, highest concentrations are found in the Indian Ocean, Red Sea, Australia and Pacific Ocean (Figure 2E; see Tables S8–S9 for a full description of the areas highlighted in blue in all components of Figure 2).

We compared species’ climate change vulnerability with their current extinction risk category on the IUCN Red List of Threatened Species [18], [19]. Habitat loss, overexploitation and invasive alien species are currently the dominant stressors to Red Listed species [3], and although climate change was specified as a threat for several species, at the time of this study, none was listed as threatened solely due to climate change. We found that 608–851 birds (6–9%), 670–933 amphibians (11–15%), and 47–73 coral species (6–9%) are both highly climate change vulnerable and already listed as threatened on the IUCN Red List. These threatened species represent 17–26%, 34–49%, and 30–39% of all climate change vulnerable birds, amphibians and corals respectively. Climate change vulnerable species are significantly more threatened than the average for all taxonomic groups, except corals under a pessimistic scenario ([birds (n = 9,856): χ^2^ = 530.95, p<0.001 (optimistic); χ^2^ = 223.78, p<0.001 (pessimistic)]; [amphibians (n = 6,204): χ^2^ = 290.93, p<0.001 (optimistic); χ^2^ = 33.22, p<0.01 (pessimistic)]; [corals (n = 797): χ^2^ = 9.02, p<0.01 (optimistic); χ^2^ = 0.68, p>0.05 (pessimistic)]) (Table S10).

**Figure 3:**
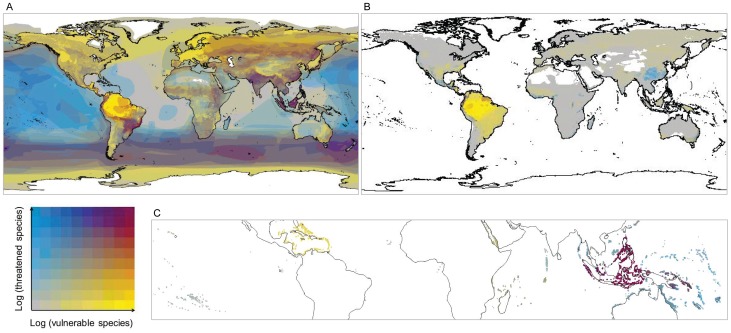
Concentrations of species that are both climate change vulnerable and threatened by non-climate stressors. Areas with high concentrations of species that are climate change vulnerable only are in yellow, threatened species (according to the IUCN Red List) only are in blue, and areas with high concentrations of both are shown in maroon. The log of total numbers of these birds, amphibians and corals are represented by A, B and C respectively (see Figure S6 for maps of the proportions of these species relative to species richness). Grey areas show where species are present but concentrations of species that are either climate change vulnerable or threatened are low; colours increase in intensity as species concentrations increase. These results are based on the moderate A1B emissions scenario for 2050 and assume optimistic assumptions for missing trait information.

Species that are both highly climate change vulnerable and threatened and the regions in which they are concentrated deserve particular conservation attention to both mitigate current threats and plan for future climate change adaptation interventions. Regions containing highest numbers of such species (maroon areas in Figure 3) include, for birds, Sundaland, the Indian subcontinent, south-eastern South America, southern oceans from 30–60^o^S, the northern Andes, much of central and eastern Asia, Africa excluding the Sahara and Congo basin, and parts of North America (Figure 3A). High concentration areas for highly climate change vulnerable and threatened amphibians include parts of the northern Andes and Mesoamerica (Figure 3B) and for corals they include the Coral Triangle extending to Sumatra and Java, the Great Barrier Reef and northern Australia, the Red Sea, East Africa and the central Indian Ocean Islands (Figure 3C). (See Figure S6 for maps of the proportions of focal species; Tables S11–S12 for a full description of the focal areas highlighted in Figure 3 and Figure S6.).

Species that are highly climate change vulnerable but are not currently threatened potentially represent new priorities for conservation. These include 1,715–4,039 (17–41%) bird species, 698–1,807 (11–29%) amphibian species and 74–174 (9–22%) coral species, and represent 74–83%, 51–66% and 61–70% of all highly climate change vulnerable birds, amphibians and corals respectively. Areas of their greatest concentrations (yellow areas in Figure 3) include, for birds, the Amazon basin and eastern South America, Europe, the Congo basin, parts of North America, northern and central Asia, and Australia. For amphibians, species that are highly climate change vulnerable but not threatened are concentrated in the Amazon basin, Eurasia, southern North America to Mesoamerica and Madagascar, and for corals, the Caribbean and southern Red Sea (Figure S6; Tables S11–S12).

**Figure 4:**
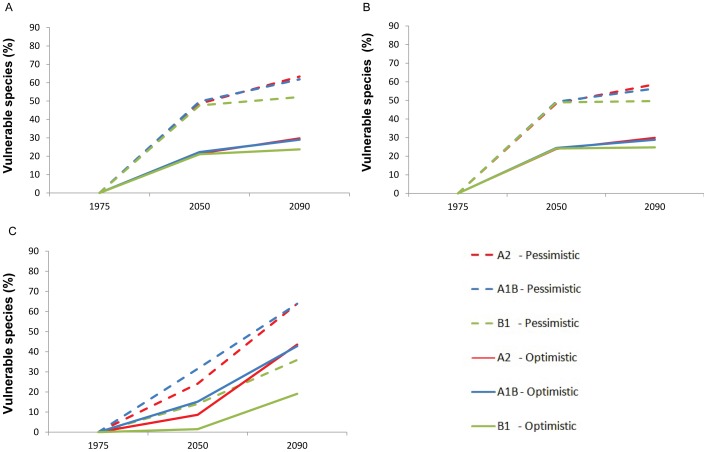
Climate change vulnerability under different emissions scenarios. Red, black, and blue lines represent the percentages of highly climate change vulnerable species under high (A2), mid-range (A1B) and low (B1) emissions scenarios for birds (A), amphibians (B) and corals (C) for 1975–2050 and 1975–2090. Optimistic and pessimistic estimates for missing biological trait data are represented by solid and dashed lines respectively.

To investigate how different climate trajectories might influence climate change vulnerability, we assessed species using high (A2), moderate (A1B) and low (B2) IPCC SRES emissions scenarios for 2050 and 2090 [20] (Figure 4; Supporting Methods in Supporting Information S1). For corals, the moderate and high scenarios produced progressively higher proportions of highly climate change vulnerable species than low scenarios in both 2050 and 2090, and mean proportions of highly climate change vulnerable corals (across all scenarios) approximately triple from 2050–2090. For birds and amphibians, high climate change vulnerability was relatively similar across scenarios for 2050, but estimates diverged by 2090, increasing overall by factors of 1.42 and 1.25 respectively. These results and the emerging additional regions of highest climate change vulnerability under high emissions scenarios (Figures S7, S8, S9) suggest that global policies that mitigate greenhouse gas emissions will substantially reduce species’ climate change vulnerability.

## Discussion

This analysis highlights the importance of broadening climate change vulnerability assessment methods, and introduces a new approach that is comparable to that used by the IUCN Red List to identify species at elevated risk of extinction. By considering biological traits that contribute significantly to species’ sensitivity and low adaptive capacity, alongside climate change projections, we assess climate change vulnerability for all species in three taxonomic groups. Since the results are relative rather than absolute climate change vulnerability measures, they cannot meaningfully be compared with statistics presented in previous global assessments (e.g., Thomas *et al.* [5] and IPCC [6]), but the considerable refinements our approach introduces provide several important contributions to climate change adaptation strategies.

Our findings identify species and regions for which assessments based on climate change exposure alone may need to be moderated. The species and regions we highlight as having high climate change sensitivity and low adaptive capacity should be considered as more vulnerable than exposure-based assessments alone may suggest. Conversely, there are also species for which climatic changes are projected to be substantial, but our assessments suggest that they may be able to cope with these better than other species, and so while monitoring and other conservation interventions might continue to be necessary, they represent a lower priority for climate change related conservation interventions in the immediate future. Trait-based climate change vulnerability assessments may be particularly valuable for species whose distributions are not reliably predicted by climate alone. Comparisons of the results of this study with those from other approaches are needed. However, given the difficulties associated with empirical validation of *all* methods of climate change vulnerability assessment, and the urgency for conservation response to climate change, the safest practical way ahead is to diversify the range and number of methods employed.

Case-by-case assessment of species’ climate change sensitivity, exposure and adaptive capacity also provides relevant information to tailor conservation interventions. We identify the species and regions of highest climate change vulnerability, as well as ‘potential persisters’, ‘potential adapters’ and species of ‘high latent risk’, and recommend generalised conservation interventions for each vulnerability class. Vulnerability traits prevalent in particular species, species groups or regions may also provide valuable information for informing more detailed management plans. As species’ traits will change little over assessment timeframes, while exposure estimates, which depend on human actions and model predictions, will be more frequently updated, climate change vulnerability assessments can be updated based primarily on changes in exposure, making them useful both as indices of change and for continually adapting management strategies.

There are some important caveats to our results that also indicate priority areas for new research (see Supporting Discussion in Supporting Information S1 for full discussion). Empirical validation that the framework and assessments are ecologically robust is of high priority [21]. The current paucity of investigations into the mechanisms of climate change impacts also hampers quantification of the extinction risk attributable to each selected trait. Our approach of highlighting the worst affected species where such evidence is lacking means that the relative climate change vulnerability measures we present cannot be meaningfully compared between birds, amphibians and corals (although comparisons should be robust within each of these groups). For corals, where bleaching frequency tolerance thresholds *are* established (i.e., a maximum of once per 5 years; p≥0.2 year^−1^ [22]), we find that these are far exceeded by our top 25% threshold (p≥0.85 year^−1^), underscoring the importance of interpreting scores as relative measures and supporting other findings that corals are at extremely high risk from climate change [22], [23]. Trait values are likely to be correlated among species and to be linked to environmental change in many different ways, resulting in thresholds and abrupt state changes [24]; detailed field studies will be required to disentangle the causes and effects and to make reliable attributions to climate change versus other pressures [25]. We also note that climate change may benefit a proportion of species. Sensitivity analyses carried out by adjusting the thresholds for the climate change vulnerability dimensions (see Supporting Methods in Supporting Information S1; Figures S10, S11, S12; Tables S13, S14, S15, S16, S17, S18, S19, S20, S21) show that the geographic focal areas we identify for each taxonomic group are fairly robust to these caveats and uncertainties.

Finally, since previous global-scale climate change vulnerability assessments [5], [6] were based on inferences made from *ad hoc* or geographically restricted studies of samples of species, our results provide the first global maps of climate change vulnerability for entire taxonomic groups. By comparing regions of highest climate change vulnerability with those of greatest threat from largely non-climate change related stressors, we identify areas of greatest concern overall, as well as those newly emerging as at risk due to climate change. This information is vital for large-scale conservation planning exercises, and highlights where more detailed assessment is needed. The approach we describe, as well as the priorities identified through this study, will strengthen global strategies to reduce climate change impacts.

## Materials and Methods

### Assessing Sensitivity and Low Adaptive Capacity

Within each of the seven trait sets, outlined as (a) to (g) in the main text, we selected traits appropriate for birds, amphibians and corals, and gathered trait data for each species using published and grey literature, online databases (e.g., [26]–[28]) and experts’ knowledge. For birds (see Table S1), we estimated habitat specialization based on the number of IUCN Red List defined habitats in which each species is known to occur, its dependence on microhabitats, and its ability to tolerate disturbance (for forest species). Environmental tolerance breadths were estimated using spatial and seasonal variability in temperature and precipitation across species’ ranges as proxies. This was calculated as average absolute deviations in historical mean temperature and mean precipitation across a species’ range and for each month, based on WorldClim’s [29] interpolated observational data for 1975 (mean 1950–2000) (see Supporting Methods in Supporting Information S1). We identified species with high dependence on fewer than 5 species (typically invertebrates) as well as those with small total or effective population sizes. We estimated intrinsic dispersal abilities using data on known mean maximum dispersal distances, and identified species with extrinsic barriers to dispersal, specifically those restricted to mountains, islands and/or polar edges of land masses. We recorded low genetic diversity where known, and used measures of generation length and reproductive output to estimate potential relative rates of evolvability.

For amphibians (see Table S2), habitat specialization was assessed based on number of IUCN Red List habitats occupied and dependence on microhabitats. Environmental tolerance ranges were estimated using spatial and seasonal variability in temperature and precipitation across species’ ranges as proxies, as for birds. We identified species that are dependent on a rainfall or increased water availability cues for their mass breeding, as well as those known or suspected to be susceptible to non-benign infection from Chytrid fungus. Species that are not known to have become established outside their natural ranges, are not associated with flowing water and have very small ranges were regarded as having relatively low intrinsic dispersal capacities. Exclusively montane and island species, and those at the polar edges of land masses or suitable natural habitats were assessed as having extrinsic dispersal barriers. Species known to have very low annual reproductive output were regarded as of lower evolvability.

For corals (see Table S3), we identified habitat specialists as species occurring exclusively in few habitats, as well as those with narrow depth ranges. Species with larvae that are likely to be particularly exposed to sea surface warming (i.e., obligatory broadcast spawners and/or brooders) were regarded as having lower tolerance to warming, and we used evidence of past mass high temperature mortality as a proxy for measuring adult colonies’ tolerances. Exclusively shallow-water species, for which impacts of rising temperatures, irradiance and storms will be unattenuated by depth, were also highlighted. We identified species not known to be associated with thermally tolerant algal *Symbiodinium* symbionts from clades D, C1 and C15, as well as those not known to be able to change or ‘shuffle’ clades and/or types over time. Particularly slow-growing and long-lived species were also highlighted. Maximum time for larval settlement was used as a proxy for species’ intrinsic dispersal capacities, and species where currents and/or cold water could present extrinsic barriers to larval dispersal were also identified.

### Assessing Exposure

#### Habitat and elevation suitability modelling to refine species’ distribution ranges

Since distribution maps for many of our focal species are only available as generalised range polygons, they often include unoccupied and potentially unsuitable areas which may be unrepresentative of the species’ climatic requirements and tolerances. To improve the accuracy of our exposure and environmental tolerance assessments, we refined species’ distribution maps (from the IUCN Red List) by excluding areas of known unsuitable habitat and elevation. Habitat suitability modelling was carried out by rasterizing the IUCN Red List maps to 10 minute resolution and cross-referencing habitat affiliations recorded in the IUCN Red List (2009) with the spatially explicit Global Land Cover 2000 habitat types [30]. The 1×1 km Global Land Cover 2000 was rasterized into twenty-three 10 minute grids, each representing one of the 23 Global Land Cover 2000 types. For each grid, cells’ values represented the percentage of the underlying 1×1 km vector covered by the land cover type in question. The probability of the presence of suitable habitat in each cell of a species’ range was calculated as the sum of the percentage presence of all suitable habitat types; following a conservative approach, we excluded only cells with zero probability of suitable habitat (see Supporting Methods in Supporting Information S1 for full details).

To exclude areas with unsuitable elevations, we used the IUCN Red List and literature to estimate species’ individual elevational limits. The 1×1 km GTOPO30 elevation dataset was rasterized to two 10 minute grids, one containing the maximum elevation and one the minimum value in the underlying vector data. Elevation suitability of the cell was calculated as the extent to which each species’ elevation range lies between the minimum and maximum elevation for the cell; again, following a conservative approach, we excluded from species’ ranges only cells with no overlap between the species’ and cell’s elevation ranges.

For corals, IUCN Red List distribution polygons (rasterized to 10 minutes) were refined by excluding areas that did not intersect with a coral reef, as defined by ReefBase’s global dataset of coral reef locations [31].

#### Calculating exposure parameters

For birds and amphibians, we considered exposure to five components of climate change, namely changes in mean temperature, temperature variability, mean precipitation, precipitation variability and sea level rise. Climate change projections were based on an ensemble of four General Circulation Models (UKMO HadCM3, MPIM ECHAM5, CSIRO MK3.5 and GFDL CM2.1), downscaled to 10 minutes [32], considering three emissions scenarios (B2, A1B and A2) for 1975 (mean 1961–1990), 2050 (mean 2041–2060) and 2090 (mean 2081–2100). The paper’s main results are based on the mid-range A1B emission scenario for projected changes from 1975 to 2050. To determine the potential role of alternative emissions pathways and longer timeframes, we then compared these results with those for A2 (high) and B1 (low) scenarios, and extended assessment timeframes to 2090 (Figure 4; Figures S7, S8, S9; Tables S19, S20, S21).

Mean temperature change was modelled as the absolute change in projected mean annual temperature across each species’ current distribution range, and change in temperature variability was calculated as the absolute difference in projected average absolute deviation in mean monthly temperatures between each month and all cells in a species’ range. To assess mean precipitation changes we calculated the absolute ratio of change in projected mean annual precipitation and measured change in precipitation variability as the absolute ratio of change in projected average absolute deviation in mean monthly precipitation between each month and all cells in a species’ range. Species were assessed as highly exposed if they were among the 25% of species with greatest projected changes for any of these four measures. They were considered to be highly exposed to sea level rise impacts if they are known to occur exclusively or primarily in one or more climate change vulnerable coastal habitats (as listed in the Supporting Methods in Supporting Information S1).

Coral exposure estimates were based on two measures. Risk of mortality due to bleaching was estimated by calculating the mean probability of severe bleaching across a species’ range (severe bleaching is projected to occur due to thermal stress resulting from degree heating month values exceeding 2°C-month) [22], [33]. Global spatial projections of maximum annual degree heating months were calculated using output from simulations of the Geophysical Fluid Dynamics Laboratory CM2.0 and CM2.1 climate models [22]. Secondly, we calculated the proportion of coral species’ ranges exposed to ‘extremely marginal’ ocean acidification levels (i.e., aragonite saturation states <3 [34]), using projections by Cao and Caldeira [35] based on the University of Victoria Earth System Climate Model version 2.8 [36]. Species were assessed as highly exposed if they were among the 25% of species with highest probability of bleaching and/or the greatest proportions of their ranges deemed unsuitable due to ocean acidification. As for birds and amphibians, the paper’s main coral results are based on changes projected by the mid-range A1B emission scenario from 1975 to 2050, and potential variation due to alternative emissions pathways (i.e., A2 and B1) and longer timeframes (i.e., 1975–2090) is explored in Figure 4, Figures S7, S8, S9 and Tables S19, S20, S21.

### Assigning Climate Change Vulnerability Scores

Species were assigned scores of ‘high’, ‘low/lower’ or ‘unknown’ risk for each trait or exposure measure. While data for some traits were qualitative or thresholds for the ‘high’ category were clear (e.g., ‘occurs only on mountain tops’), in ∼66% of traits, there was no *a priori* basis for setting a particular threshold (e.g., for projected mean precipitation change). In such cases we scored the worst affected 25% of species as ‘high’. We explored the sensitivity of our results to shifting this threshold to include the worst affected 35% and 15% of species, as well as to stricter and more lenient expert-defined thresholds (Figures S10, S11, S12; Tables S16, S17, S18, S19, S20, S21), as well as to the choice of the individual traits included (Tables S13, S14, S15, S16).

A species that scored ‘high’ under *any* trait or exposure measure triggered a score of ‘high’ for the vulnerability dimension to which it belonged (e.g., a species with a ‘high’ score under habitat specialisation was then considered to have a ‘high’ sensitivity score). To qualify as highly climate change vulnerable overall, species required ‘high’ scores for *all three* of sensitivity, low adaptive capacity and exposure (see Figure S13). We repeat the important caveat that, due to the scarcity of direct evidence to support trait scoring thresholds, climate change vulnerability scores must be interpreted as relative measures, and comparison of percentages of climate change vulnerable species between taxonomic groups is not meaningful (See Supporting Discussion in Supporting Information S1).

We document the regions and families containing highest numbers of climate change vulnerable species, and compare results with assessments of non-climatic threat from the IUCN Red List. To reflect uncertainty due to unknown values for some species-trait combinations, we repeated our analyses treating unknowns as either ‘high’ (pessimistic scenario) or ‘low/lower’ (optimistic scenario) and present results as ranges of plausible values between these extremes.

Full Methods and associated references are available in Supporting Information S1.

## Supporting Information

Figure S1 **The relationship between climate change vulnerability dimensions for families containing ten or more species (based on an optimistic scenario for unknown trait values).** Graphs show the percentages of each family’s species that are highly sensitive vs. of low adaptive capacity (A–C), sensitive vs. exposed (D–F), and of low adaptive capacity vs. exposed (H–J) for birds, amphibians and corals respectively.
(TIF)

Figure S2 **Geographic concentrations of bird species that are highly sensitive (A–B), exposed (C–D), have low adaptive capacity (E–F) and are highly climate change vulnerable overall (G–H), based on an optimistic scenario for unknown trait values.** Parts A, C, E and G represent total numbers of species, while B, D, F and H show the proportions of total species in the groups i.e., relative to total species richness.
(TIF)

Figure S3 **Geographic concentrations of amphibian species that are highly sensitive (A–B), exposed (C–D), have low adaptive capacity (E–F) and are highly climate change vulnerable overall (G–H), based on an optimistic scenario for unknown trait values.** Parts A, C, E and G represent total numbers of species, while B, D, F and H show the proportions of total species in the groups i.e., relative to total species richness.
(TIF)

Figure S4 **Geographic concentrations of coral species that are highly sensitive (A–B), exposed (C–D), have low adaptive capacity (E–F) and are highly climate change vulnerable overall (G–H), based on an optimistic scenario for unknown trait values.** Parts A, C, E and G represent total numbers of species, while B, D, F and H show the proportions of total species in the groups i.e., relative to total species richness.
(TIF)

Figure S5 **Geographic concentrations of species that are highly vulnerable under a pessimistic scenario (i.e., when unknown trait scores are assumed to be high climate change vulnerability scores) but not under an optimistic scenario (i.e., when unknown trait scores are assumed to be low climate change vulnerability scores), for birds, amphibians and corals (A, C, and E respectively).** B, D, and F show the numbers of the above species relative to the number of species already known to be climate change vulnerable there (e.g., a score of six shows that there could be up to six times more highly climate change vulnerable species if unknown trait values represent high vs. low values).
(TIF)

Figure S6 **Bivariate plots showing areas with highest logged proportions (relative to species richness) of species that are climate change vulnerable only in yellow, threatened only in blue, and both highly climate change vulnerable and threatened in maroon.** Logged total numbers of birds, amphibians and corals are represented by A, B and C respectively (see Fig. 3 for maps of the total numbers of species). Grey areas show where species are present, but few are climate change vulnerable or threatened; colours increase in intensity as species concentrations increase. Plots assume optimistic assumptions for missing trait information.
(TIF)

Figure S7 **Foci of highly climate change vulnerable birds under three IPCC SRES climate change scenarios for 2050 and 2090.** Low range scenario B1, moderate A1B (used as the baseline for all other assessments in this study) and high range A2 are represented by A, C and E respectively for 2050, while B, D and F show the same scenarios for 2090.
(TIF)

Figure S8 **Foci of highly climate change vulnerable amphibians under three IPCC SRES climate change scenarios for 2050 and 2090.** Low range scenario B1, moderate A1B (used as the baseline for all other assessments in this study) and high range A2 are represented by A, C and E respectively for 2050, while B, D and F show the same scenarios for 2090.
(TIF)

Figure S9 **Foci of highly climate change vulnerable corals under three IPCC SRES climate change scenarios for 2050 and 2090.** Low range scenario B1, moderate A1B (used as the baseline for all other assessments in this study) and high range A2 are represented by A, C and E respectively for 2050, while B, D and F show the same scenarios for 2090.
(TIF)

Figure S10 **Foci of highly climate change vulnerable birds calculated using five trait threshold scenarios, namely:** strict percentage thresholds (A), strict expert thresholds (B), a moderate scenario for percentage and expert thresholds (i.e., as used for the results presented in Table 2 and Figure 2) (C), lenient percentage thresholds (D), and lenient expert thresholds (E). Results are calculated based on an optimistic scenario for unknowns under emission scenario A1B for 2050.
(TIF)

Figure S11 **Foci of highly climate change vulnerable amphibians calculated using five trait threshold scenarios, namely:** strict percentage thresholds (A), strict expert thresholds (B), a moderate scenario for percentage and expert thresholds (i.e., as used for the results presented in Table 2 and Figure 2) (C), lenient percentage thresholds (D), and lenient expert thresholds (E). Results are calculated based on an optimistic scenario for unknowns under emission scenario A1B for 2050.
(TIF)

Figure S12 **Foci of highly climate change vulnerable corals calculated using five trait threshold scenarios, namely:** strict percentage thresholds (A), strict expert thresholds (B), a moderate scenario for percentage and expert thresholds (i.e., as used for the results presented in Table 2 and Figure 2) (C), lenient percentage thresholds (D), and lenient expert thresholds (E). Results are calculated based on an optimistic scenario for unknowns under emission scenario A1B for 2050.
(TIF)

Figure S13 **Schematic diagram showing the three dimensions of climate change vulnerability (sensitivity, exposure and low adaptive capacity) and the biological and environmental trait sets contributing to them.** The three boxes explain the logic system used to classify species as high in each climate change vulnerability dimension. Species are considered highly climate change vulnerable overall if they score high under all three of sensitivity, exposure and low adaptive capacity.
(TIF)

Table S1 **Traits rendering bird species as of ‘high’ and ‘low/lower’ climate change vulnerability, and the number of species qualifying under these categories and as unknown according to each trait.**
(DOCX)

Table S2 **Traits rendering amphibian species as of ‘high’ and ‘low/lower’ climate change vulnerability, and the number of species qualifying under these categories and as unknown according to each trait.**
(DOCX)

Table S3 **Traits rendering coral species as of ‘high’ and ‘low/lower’ climate change vulnerability, and the number of species qualifying under these categories and as unknown according to each trait.**
(DOCX)

Table S4 **The number and percentage of bird, amphibian and coral families with significantly more and less highly climate change vulnerable species than expected from the observed overall frequency in each group (based on an optimistic scenario for missing data).**
(DOCX)

Table S5 **Summary of the 5 most and least climate change vulnerable bird families.** Percentages represent the proportions of species qualifying as high under each climate change vulnerability dimension (i.e., sensitivity, exposure, low adaptive capacity and overall climate change vulnerability). Climate change vulnerability traits are listed where they characterise more than 25% of species in the family.
(DOCX)

Table S6 **Summary of the 5 most and least climate change vulnerable amphibian families.** Percentages represent the proportions of species qualifying as high under each climate change vulnerability dimension (i.e., sensitivity, exposure, low adaptive capacity and overall climate change vulnerability). Climate change vulnerability traits are listed where they characterise more than 25% of species in the family.
(DOCX)

Table S7 **Summary of the four families that have mean climate change vulnerability scores that are significantly greater than the mean for all corals, as well as the three with significantly lower mean susceptibilities.** Percentages represent the proportions of species qualifying as high under each climate change vulnerability dimension (i.e., sensitivity, exposure, low adaptive capacity and overall climate change vulnerability). Climate change vulnerability traits are listed where they characterise more than 25% of species in the family.
(DOCX)

Table S8 **Summary of geographic focal areas (identified in** **Figure 2** **(A, C, and E)) that contain high total numbers of species that are (i) highly sensitive and of low adaptive capacity, (ii) highly exposed, and both (i) and (ii).**
(DOCX)

Table S9 **Summary of geographic focal areas (identified in** **Figure 2** **(B, D and F)) that contain high proportions of species, relative to species richness, that are (i) highly sensitive and of low adaptive capacity, (ii) highly exposed and both (i) and (ii).**
(DOCX)

Table S10 **The numbers and percentages of birds, amphibians and coral species with various combinations of threat status (according to the IUCN Red List) and high climate change vulnerability. Optimistic scores are based on climate change vulnerability scores calculated on the assumption that unknown trait values reflect ‘not high’ scores; pessimistic scores are based on the assumption that unknown trait values reflect high scores.** Independence between numbers of species that are threatened and highly climate change vulnerable was tested using Pearson’s Chi-square test (d.f.  =  1); total species numbers (n), Chi-squared coefficients and P values are shown for each taxonomic group.
(DOCX)

Table S11 **Summary of the geographic focal areas identified in** **Figure 3** **that contain high total numbers of species that are threatened (according to the IUCN Red List^TM^), climate change vulnerable and high numbers of both.**
(DOCX)

Table S12 **Summary of the geographic focal areas identified in Figure S6 that contain high relative numbers of species that are threatened (according to the IUCN Red List), climate change vulnerable and high numbers of both.**
(DOCX)

Table S13 **Summary of the numbers of species and size of geographic area uniquely identified by each of the biological trait used to assess overall climate change vulnerability of birds.** Traits highlighted in yellow identify the five most influential traits for uniquely identifying numbers of species and those in red text identify these traits for geographic areas. Trait and trait group descriptions are shortened versions; full titles are shown in Table S1.
(DOCX)

Table S14 **Summary of the numbers of species and size of geographic area uniquely identified by each of the biological traits used to assess overall climate change vulnerability of amphibians.** Traits highlighted in yellow identify the five most influential traits for uniquely identifying numbers of species and those in red text identify these traits for geographic areas. Trait and trait group descriptions are shortened versions; full titles are shown in Table S2.
(DOCX)

Table S15 **Summary of the numbers of species and size of geographic area uniquely identified by each of the biological traits used to assess overall climate change vulnerability of corals.** Traits highlighted in yellow identify the five most influential traits for uniquely identifying numbers of species and those in red text identify these traits for geographic areas. Trait and trait group descriptions are shortened versions; full titles are shown in Table S3.
(DOCX)

Table S16 **Traits rendering bird species as of ‘high’ climate change vulnerability, and the number of species qualifying under these categories and as unknown, according to three trait threshold scenarios, namely more lenient thresholds, the original or moderate thresholds (i.e., as used for the results presented in** **Table 2** **and** **Figure 2** **) and stricter thresholds.** Thresholds for traits indicated with a **(P)** and highlighted in blue were selected based on arbitrary percentage thresholds (35%, 25% and 15%) while those indicated by an **(E)** and highlighted in green were selected based on experts’ judgements. All results shown are based on an optimistic scenario for 2050 under the A1B emission scenario.
(DOCX)

Table S17 **Traits rendering amphibian species as of ‘high’ climate change vulnerability, and the number of species qualifying under these categories and as unknown, according to three trait threshold scenarios, namely more lenient thresholds, the original or moderate thresholds (i.e., as used for the results presented in** **Table 2** **and** **Figure 2** **) and stricter thresholds.** Thresholds for traits indicated with a **(P)** and highlighted in blue were selected based on arbitrary percentage thresholds (35%, 25% and 15%) while those indicated by an **(E)** and highlighted in green were selected based on experts’ judgements. All results shown are based on an optimistic scenario for 2050 under the A1B emission scenario.
(DOCX)

Table S18 **Traits rendering coral species as of ‘high’ climate change vulnerability, and the number of species qualifying under these categories and as unknown, according to three trait threshold scenarios, namely more lenient thresholds, the original or moderate thresholds (i.e., as used for the results presented in** **Table 2** **and** **Figure 2** **) and stricter thresholds.** Thresholds for traits indicated with a **(P)** and highlighted in blue were selected based on arbitrary percentage thresholds (35%, 25% and 15%) while those indicated by an **(E)** and highlighted in green were selected based on experts’ judgements. All results shown are based on an optimistic scenario for 2050 under the A1B emission scenario.
(DOCX)

Table S19 **Summary of the potential impacts of sources of uncertainty on numbers of climate change vulnerable bird species.** These include scenarios of impacts of missing data (unknowns), the choice of percentage thresholds, the selection of thresholds by experts, the greenhouse gas emission scenario applied and the time frames considered. Percentages represent the numbers of climate change vulnerable species relative to the total number of species. Emissions scenarios and time frame results presented are for terrestrial regions only. Except where specified, assessments are based on optimistic unknowns scenario under emissions scenario A1B for 2050.
(DOCX)

Table S20 **Summary of the potential impacts of sources of uncertainty on numbers of climate change vulnerable amphibian species.** These include scenarios of impacts of missing data (unknowns), the choice of percentage thresholds, the selection of thresholds by experts, the greenhouse gas emission scenario applied and the time frames considered. Percentages represent the numbers of climate change vulnerable species relative to the total number of species. Emissions scenarios and time frame results presented are for terrestrial regions only. Except where specified, assessments are based on optimistic unknowns scenario under emissions scenario A1B for 2050.
(DOCX)

Table S21 **Summary of the potential impacts of sources of uncertainty on numbers of climate change vulnerable coral species.** These include scenarios of impacts of missing data (unknowns), the choice of percentage thresholds, the selection of thresholds by experts, the greenhouse gas emission scenario applied and the time frames considered. Percentages represent the numbers of climate change vulnerable species relative to the total number of species. Except where specified, assessments are based on optimistic unknowns scenario under emissions scenario A1B for 2050.
(DOCX)

Supporting Information S1 **Supporting Methods, Supporting Discussion, Figures S1–13, Tables S1–21 and Supporting Information References.**
(PDF)

Appendix A **Climate change vulnerability scores for bird species.**
(PDF)

Appendix B **Climate change vulnerability scores for amphibian species.**
(PDF)

Appendix C **Climate change vulnerability scores for coral species.**
(PDF)
