# Supplementary figures and images for: Identifying the World's Most Climate Change Vulnerable Species: A Systematic Trait-Based Assessment of all Birds, Amphibians and Corals
Source: PLoS One. 2013 Jun 12;8(6):e65427. doi: 10.1371/journal.pone.0065427 (PMC3680427; doi:10.1371/journal.pone.0065427)

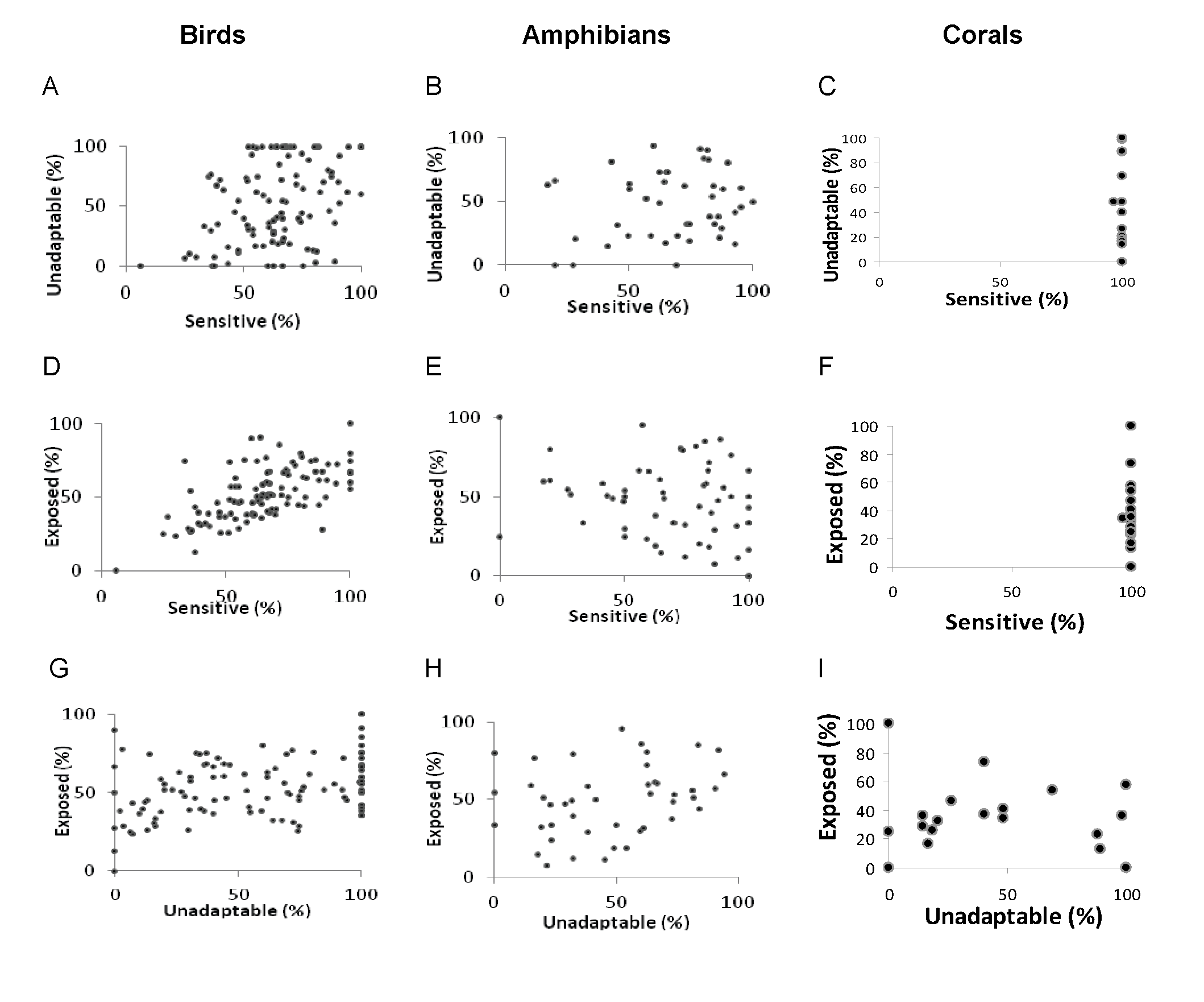

Supplement: Figure S1 — The relationship between climate change vulnerability dimensions for families containing ten or more species (based on an optimistic scenario for unknown trait values). Graphs show the percentages of each family’s species that are highly sensitive vs. of low adaptive capacity (A–C), sensitive vs. exposed (D–F), and of low adaptive capacity vs. exposed (H–J) for birds, amphibians and corals respectively. (TIF) [file pone.0065427.s001.tif]

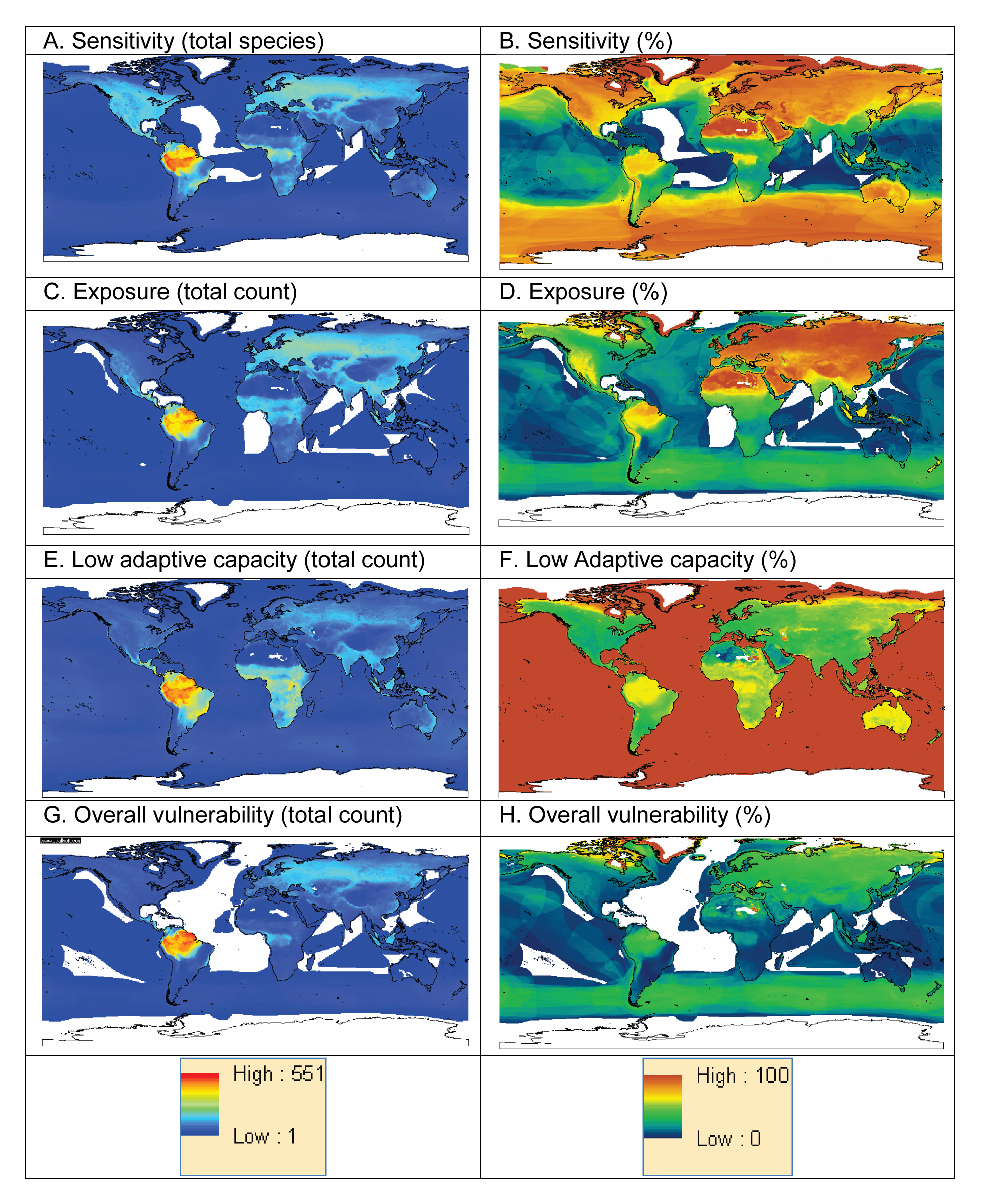

Supplement: Figure S2 — Geographic concentrations of bird species that are highly sensitive (A–B), exposed (C–D), have low adaptive capacity (E–F) and are highly climate change vulnerable overall (G–H), based on an optimistic scenario for unknown trait values. Parts A, C, E and G represent total numbers of species, while B, D, F and H show the proportions of total species in the groups i.e., relative to total species richness. (TIF) [file pone.0065427.s002.tif]

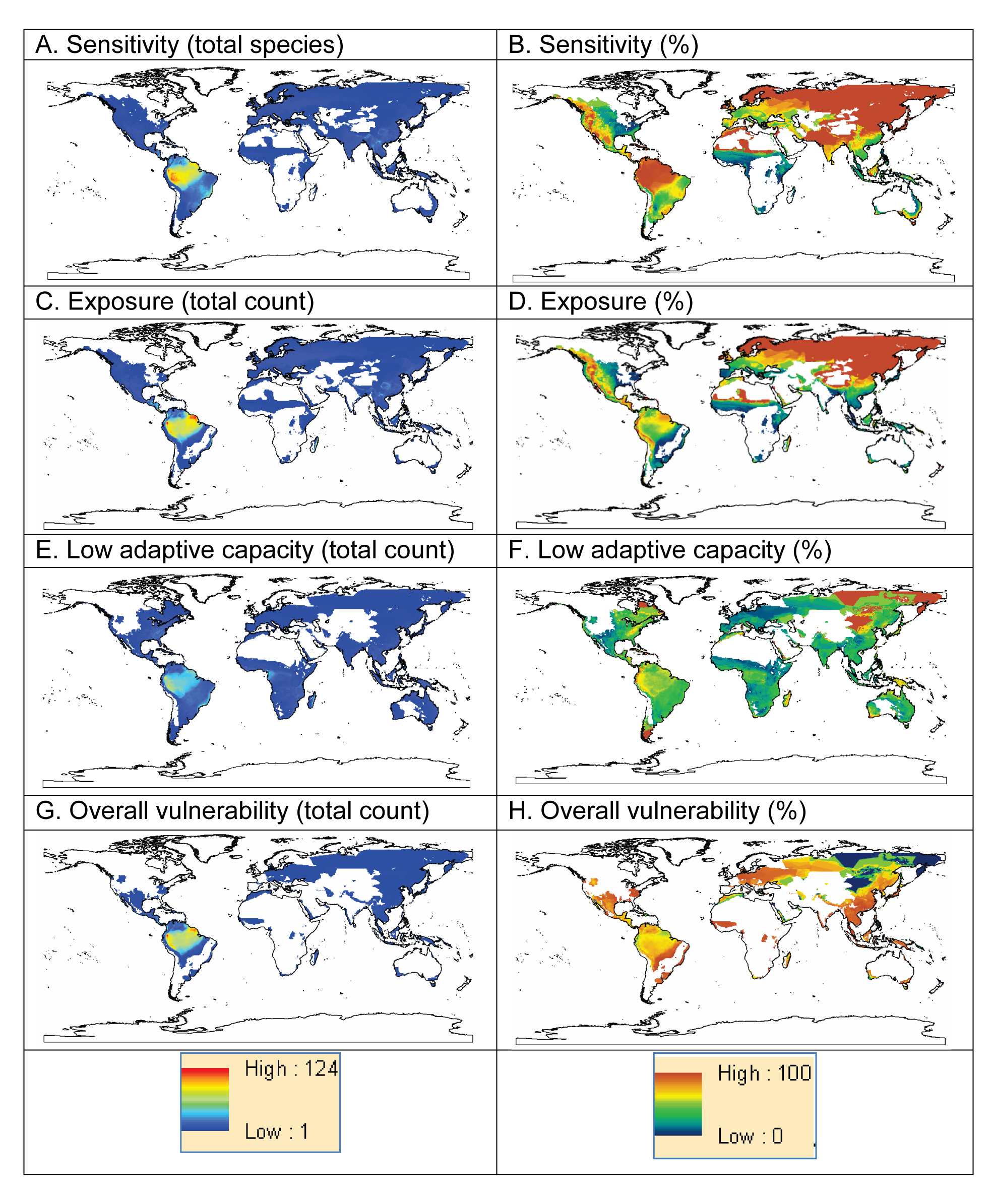

Supplement: Figure S3 — Geographic concentrations of amphibian species that are highly sensitive (A–B), exposed (C–D), have low adaptive capacity (E–F) and are highly climate change vulnerable overall (G–H), based on an optimistic scenario for unknown trait values. Parts A, C, E and G represent total numbers of species, while B, D, F and H show the proportions of total species in the groups i.e., relative to total species richness. (TIF) [file pone.0065427.s003.tif]

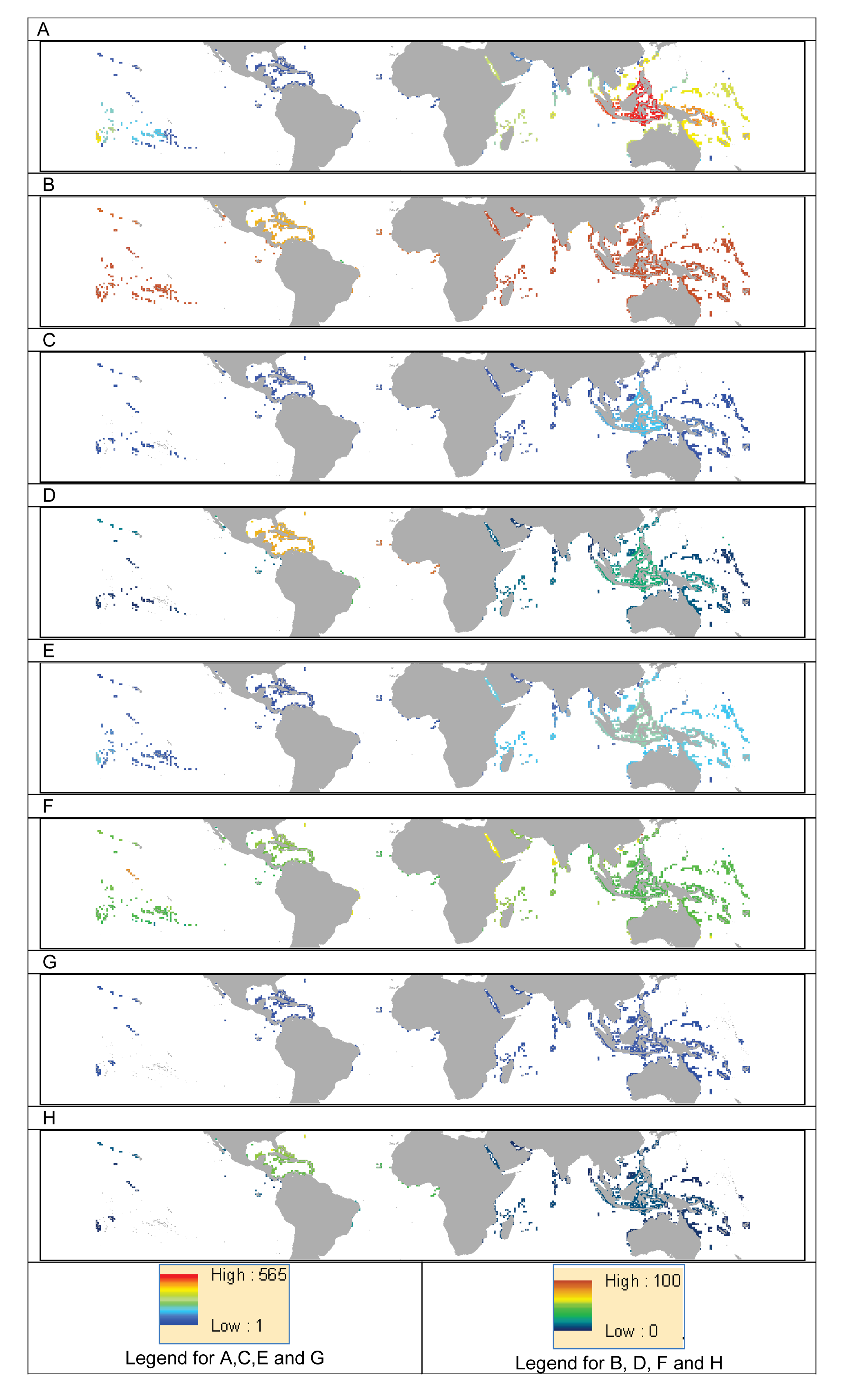

Supplement: Figure S4 — Geographic concentrations of coral species that are highly sensitive (A–B), exposed (C–D), have low adaptive capacity (E–F) and are highly climate change vulnerable overall (G–H), based on an optimistic scenario for unknown trait values. Parts A, C, E and G represent total numbers of species, while B, D, F and H show the proportions of total species in the groups i.e., relative to total species richness. (TIF) [file pone.0065427.s004.tif]

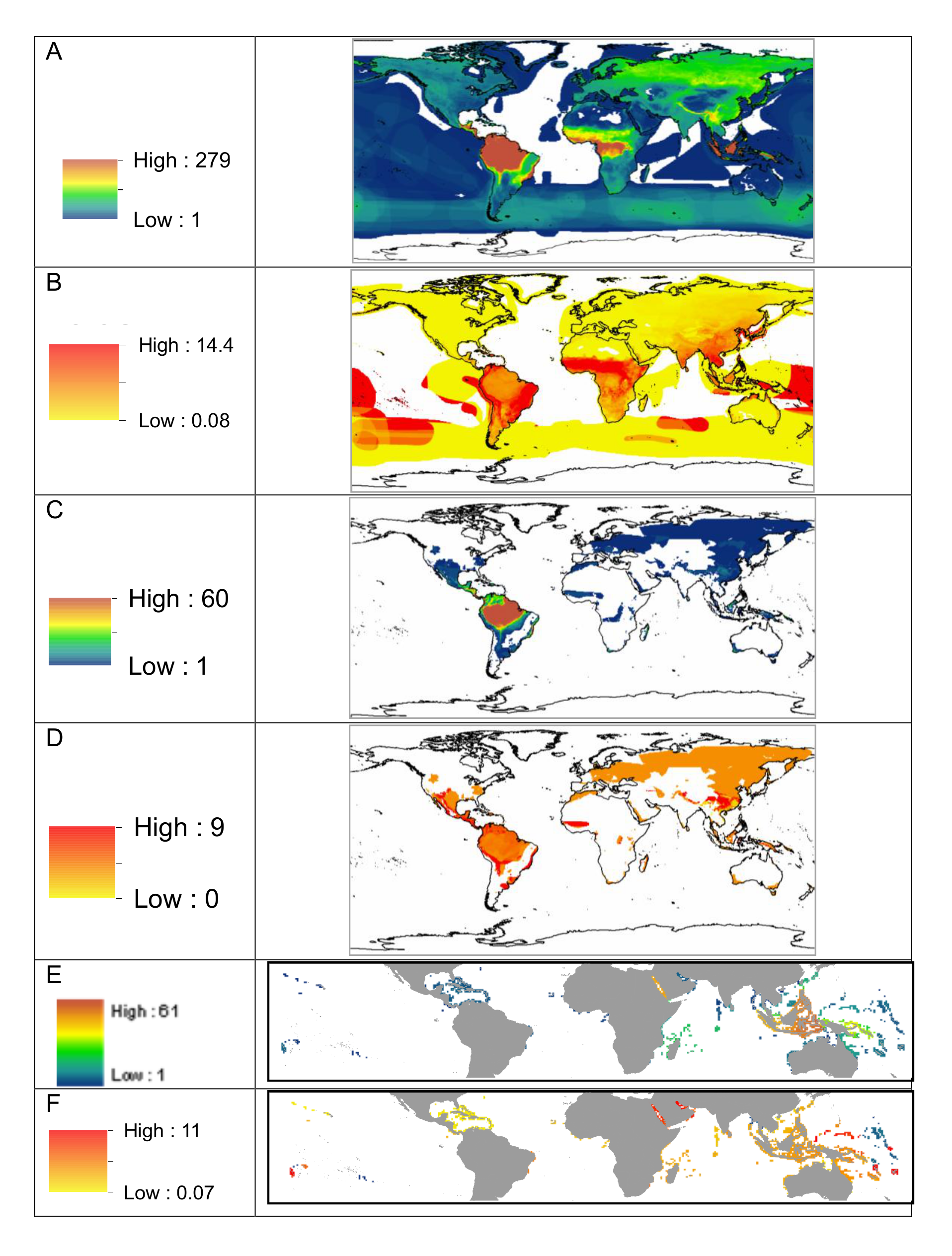

Supplement: Figure S5 — Geographic concentrations of species that are highly vulnerable under a pessimistic scenario (i.e., when unknown trait scores are assumed to be high climate change vulnerability scores) but not under an optimistic scenario (i.e., when unknown trait scores are assumed to be low climate change vulnerability scores), for birds, amphibians and corals (A, C, and E respectively). B, D, and F show the numbers of the above species relative to the number of species already known to be climate change vulnerable there (e.g., a score of six shows that there could be up to six times more highly climate change vulnerable species if unknown trait values represent high vs. low values). (TIF) [file pone.0065427.s005.tif]

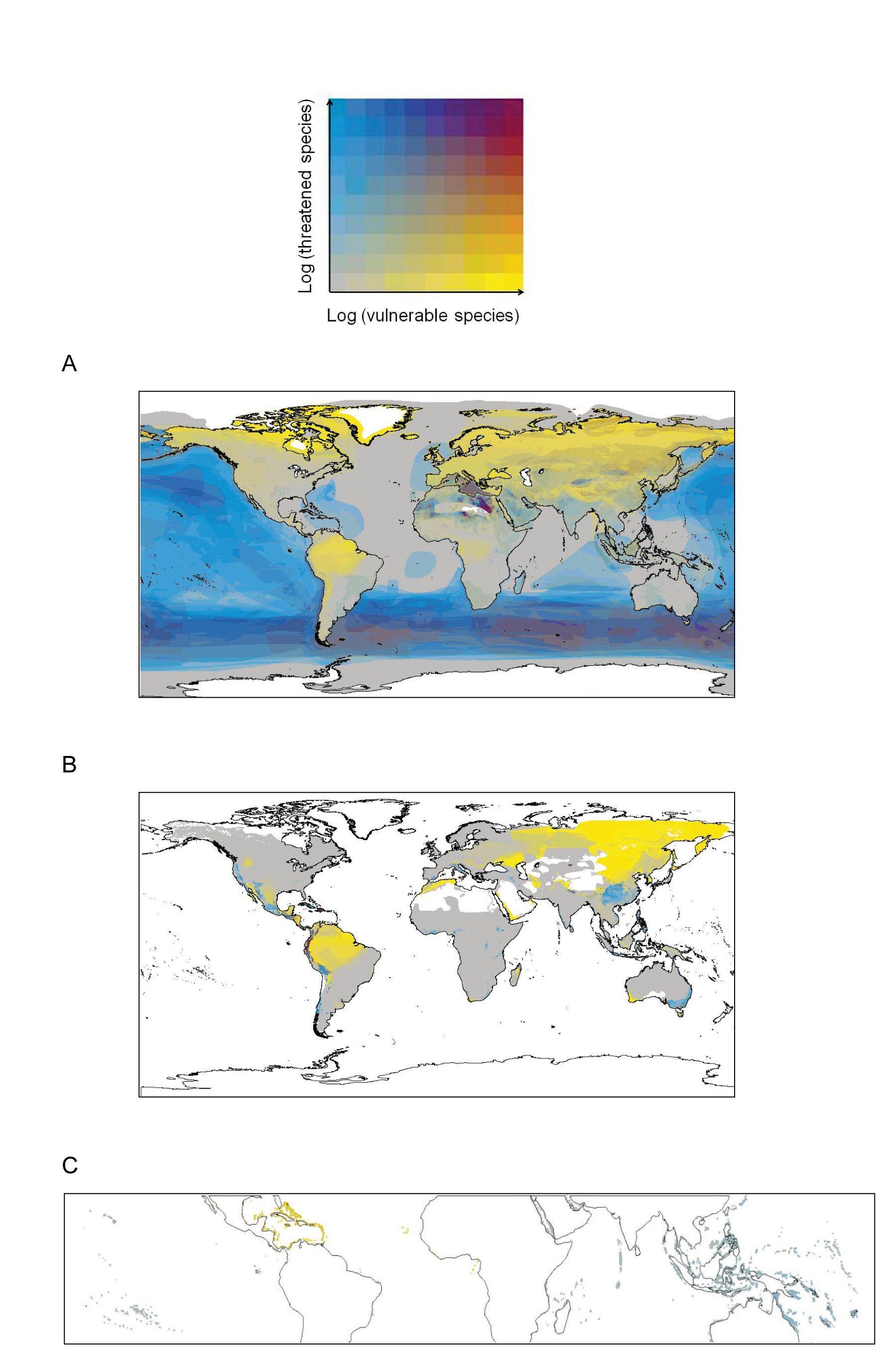

Supplement: Figure S6 — Bivariate plots showing areas with highest logged proportions (relative to species richness) of species that are climate change vulnerable only in yellow, threatened only in blue, and both highly climate change vulnerable and threatened in maroon. Logged total numbers of birds, amphibians and corals are represented by A, B and C respectively (see Fig. 3 for maps of the total numbers of species). Grey areas show where species are present, but few are climate change vulnerable or threatened; colours increase in intensity as species concentrations increase. Plots assume optimistic assumptions for missing trait information. (TIF) [file pone.0065427.s006.tif]

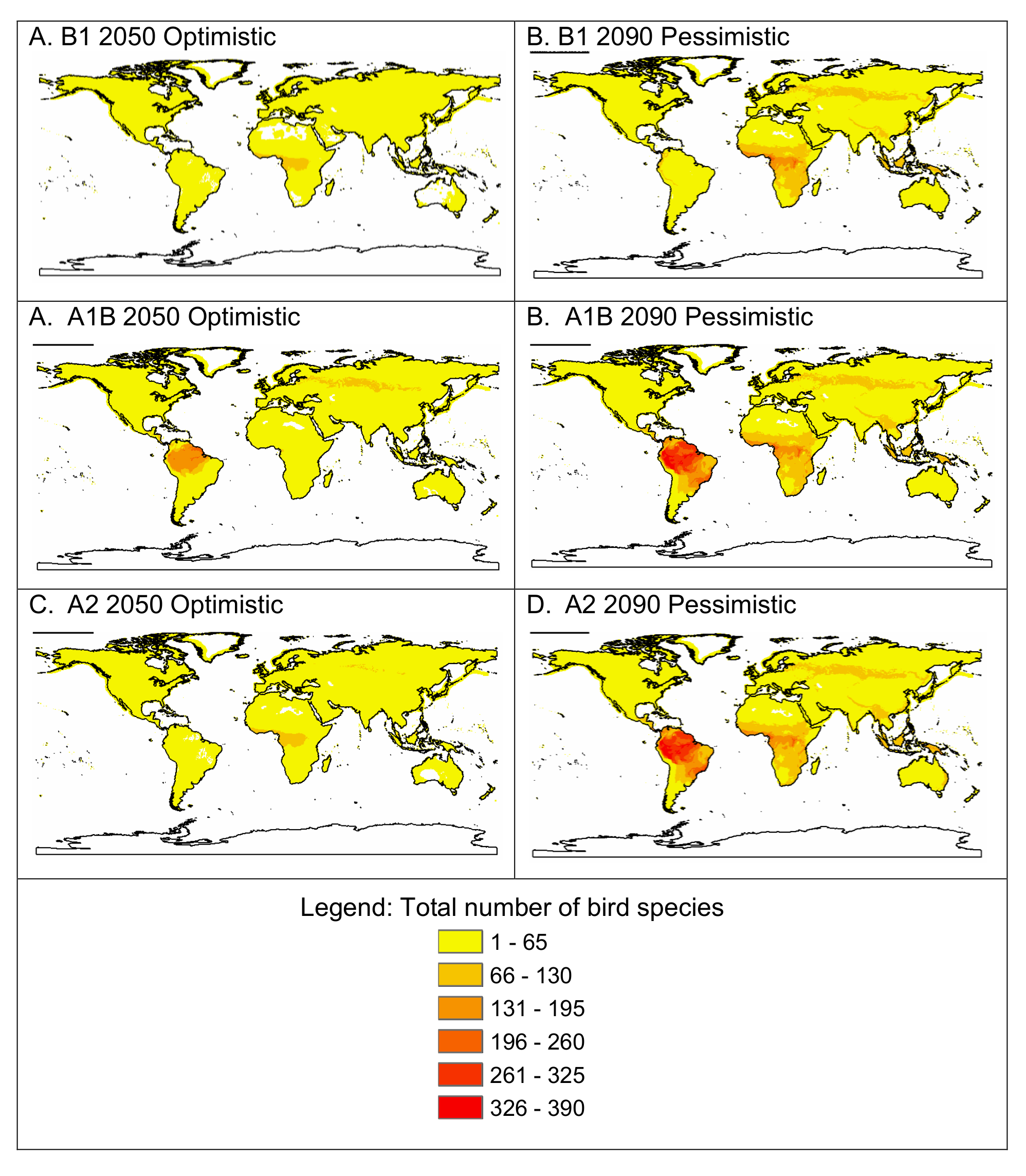

Supplement: Figure S7 — Foci of highly climate change vulnerable birds under three IPCC SRES climate change scenarios for 2050 and 2090. Low range scenario B1, moderate A1B (used as the baseline for all other assessments in this study) and high range A2 are represented by A, C and E respectively for 2050, while B, D and F show the same scenarios for 2090. (TIF) [file pone.0065427.s007.tif]

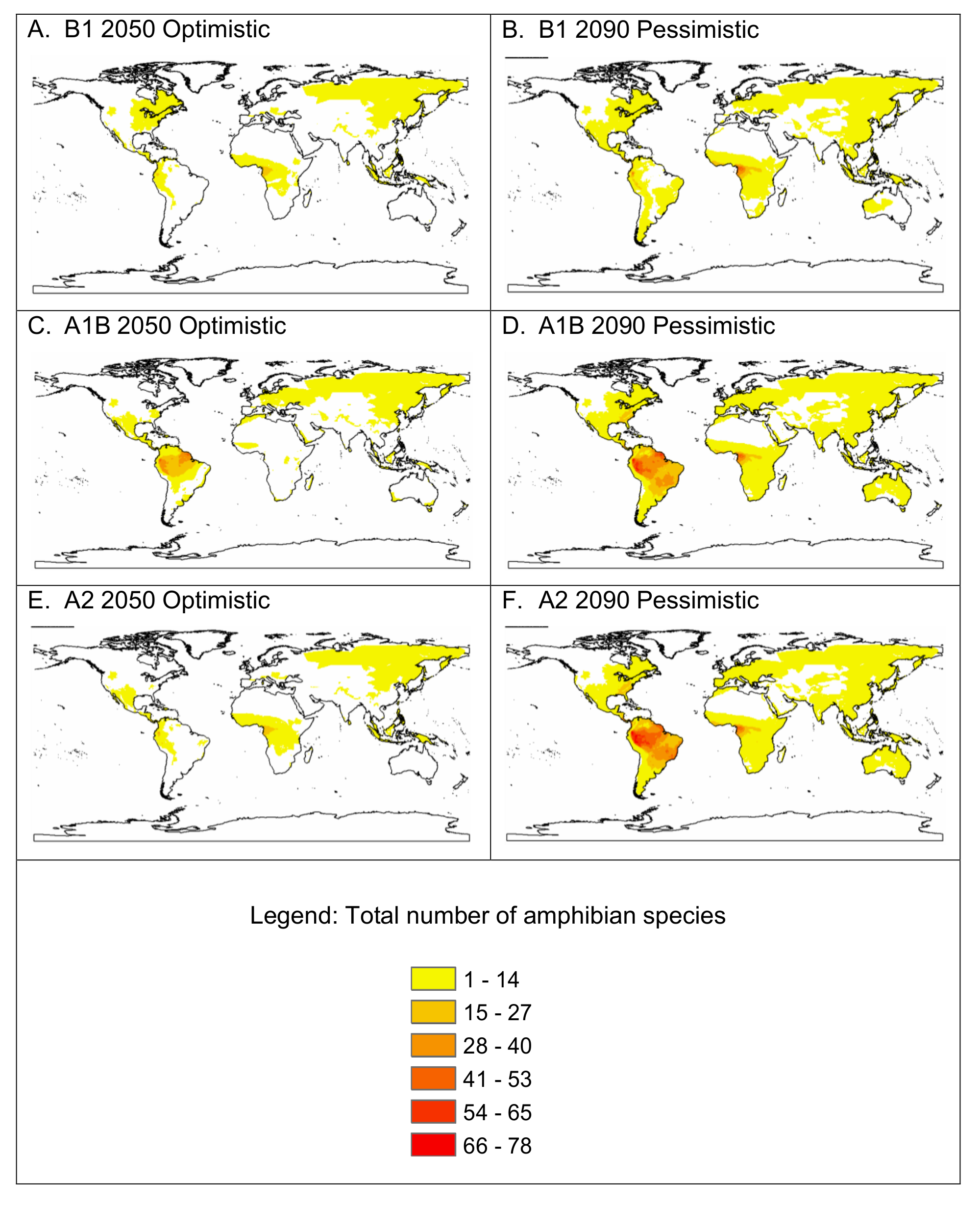

Supplement: Figure S8 — Foci of highly climate change vulnerable amphibians under three IPCC SRES climate change scenarios for 2050 and 2090. Low range scenario B1, moderate A1B (used as the baseline for all other assessments in this study) and high range A2 are represented by A, C and E respectively for 2050, while B, D and F show the same scenarios for 2090. (TIF) [file pone.0065427.s008.tif]

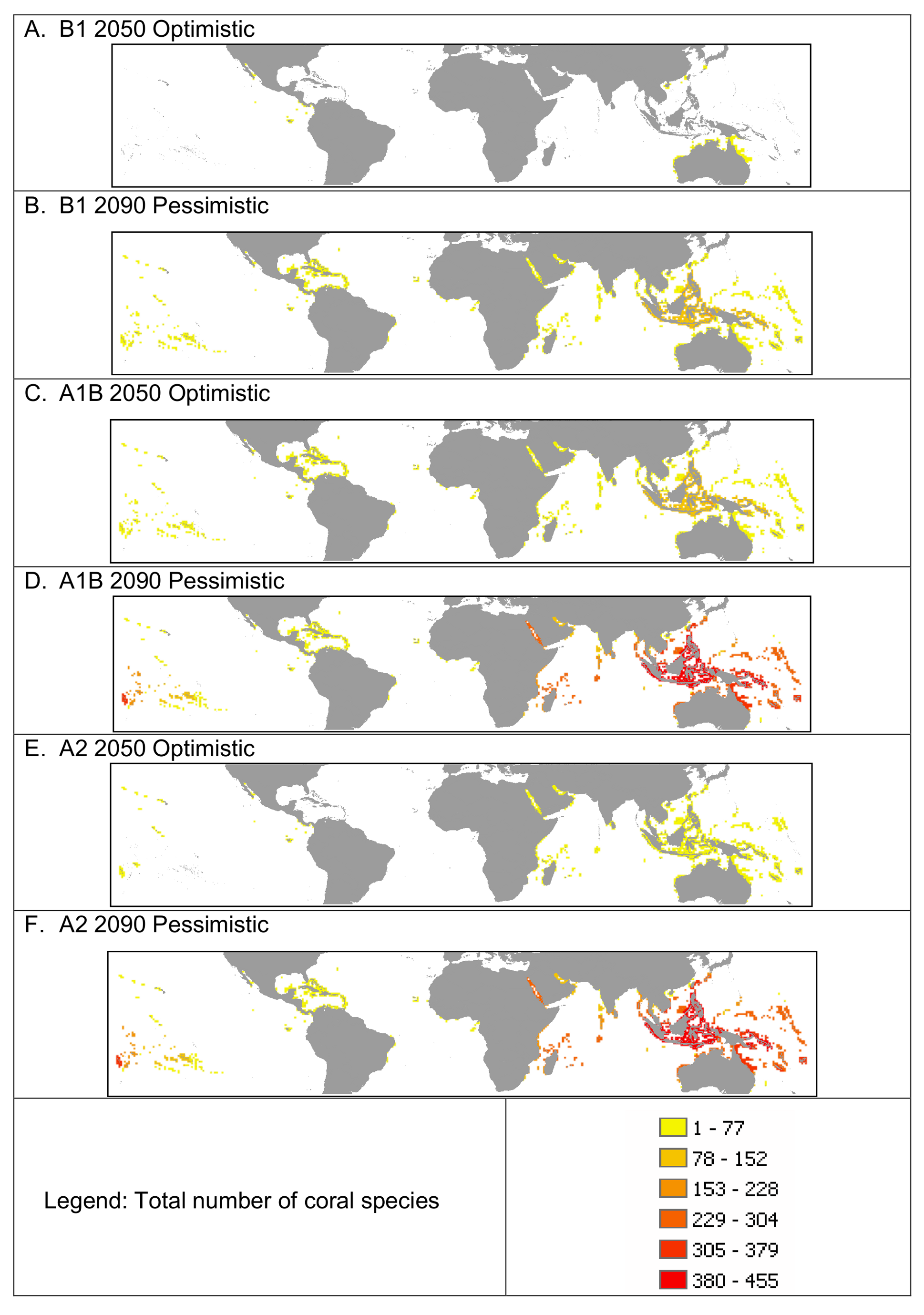

Supplement: Figure S9 — Foci of highly climate change vulnerable corals under three IPCC SRES climate change scenarios for 2050 and 2090. Low range scenario B1, moderate A1B (used as the baseline for all other assessments in this study) and high range A2 are represented by A, C and E respectively for 2050, while B, D and F show the same scenarios for 2090. (TIF) [file pone.0065427.s009.tif]

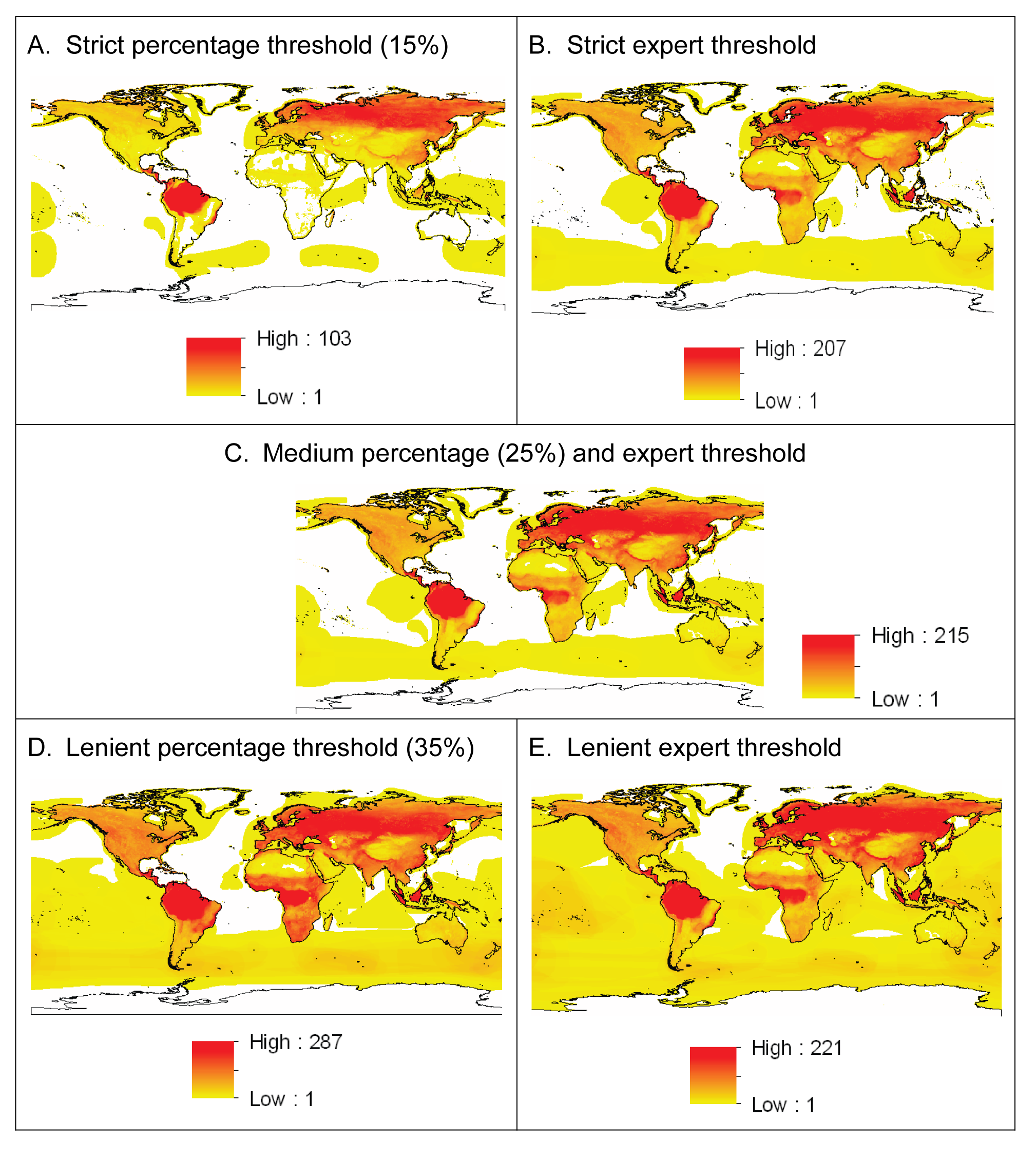

Supplement: Figure S10 — Foci of highly climate change vulnerable birds calculated using five trait threshold scenarios, namely: strict percentage thresholds (A), strict expert thresholds (B), a moderate scenario for percentage and expert thresholds (i.e., as used for the results presented in Table 2 and Figure 2) (C), lenient percentage thresholds (D), and lenient expert thresholds (E). Results are calculated based on an optimistic scenario for unknowns under emission scenario A1B for 2050. (TIF) [file pone.0065427.s010.tif]

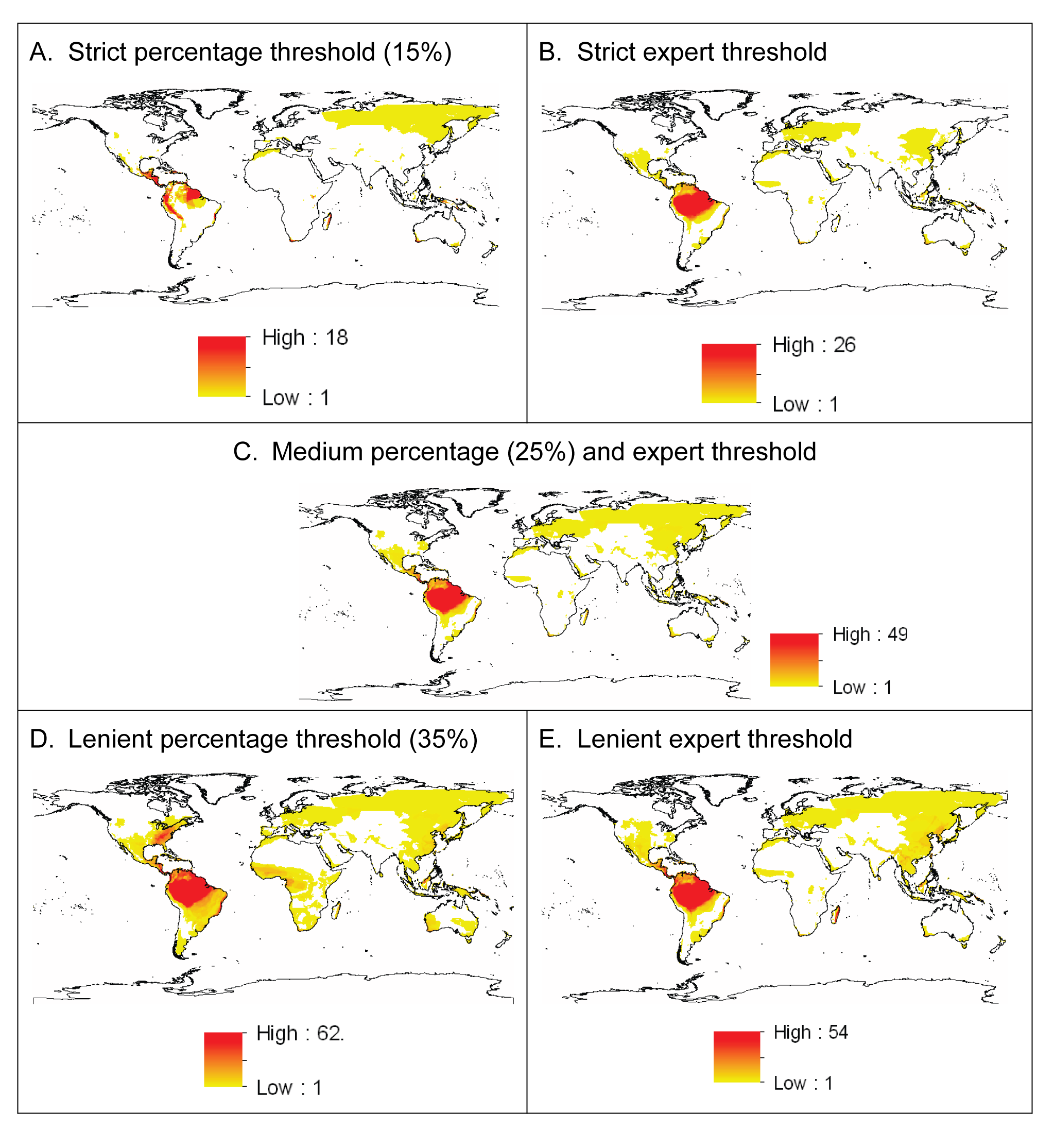

Supplement: Figure S11 — Foci of highly climate change vulnerable amphibians calculated using five trait threshold scenarios, namely: strict percentage thresholds (A), strict expert thresholds (B), a moderate scenario for percentage and expert thresholds (i.e., as used for the results presented in Table 2 and Figure 2) (C), lenient percentage thresholds (D), and lenient expert thresholds (E). Results are calculated based on an optimistic scenario for unknowns under emission scenario A1B for 2050. (TIF) [file pone.0065427.s011.tif]

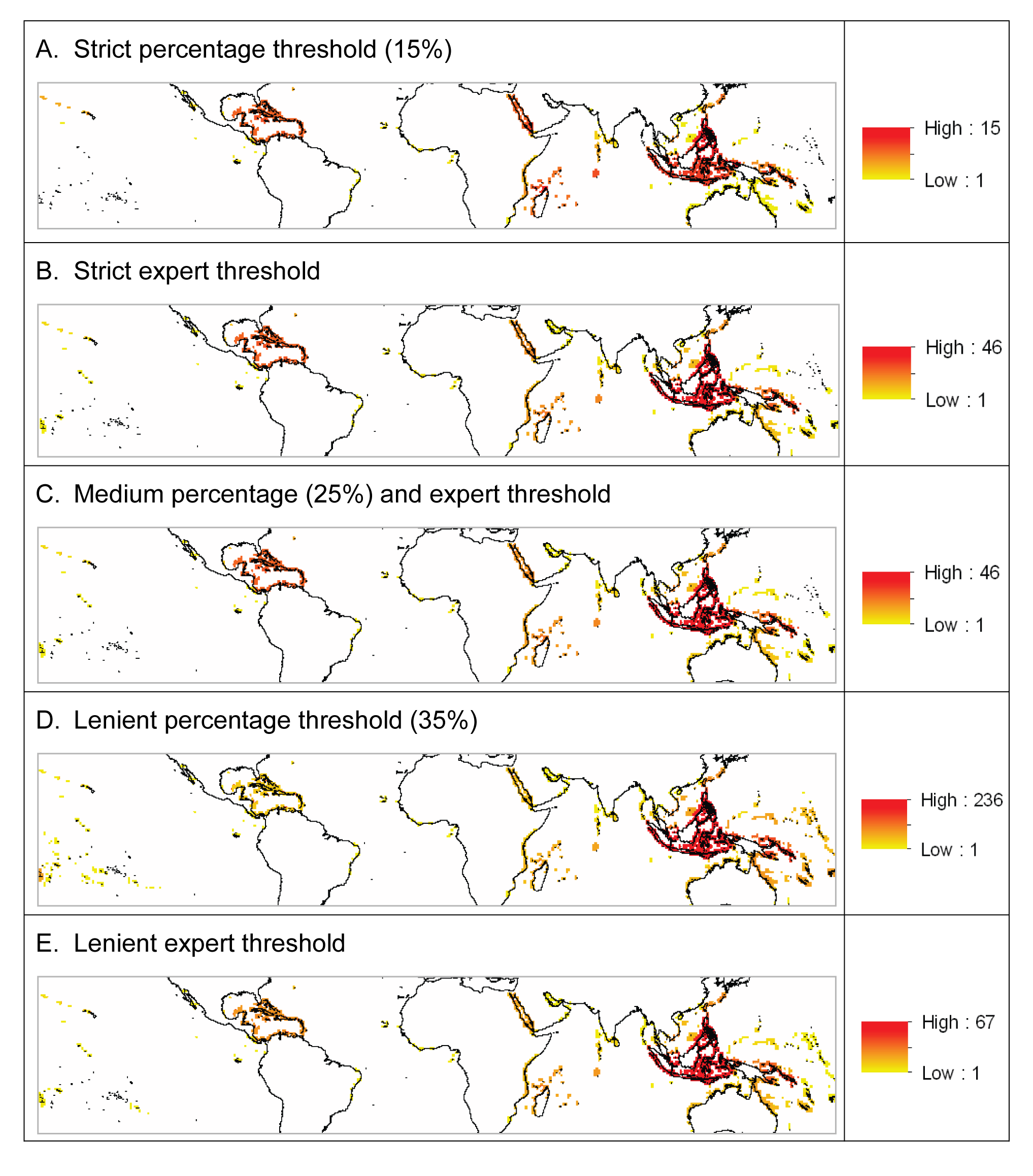

Supplement: Figure S12 — Foci of highly climate change vulnerable corals calculated using five trait threshold scenarios, namely: strict percentage thresholds (A), strict expert thresholds (B), a moderate scenario for percentage and expert thresholds (i.e., as used for the results presented in Table 2 and Figure 2) (C), lenient percentage thresholds (D), and lenient expert thresholds (E). Results are calculated based on an optimistic scenario for unknowns under emission scenario A1B for 2050. (TIF) [file pone.0065427.s012.tif]

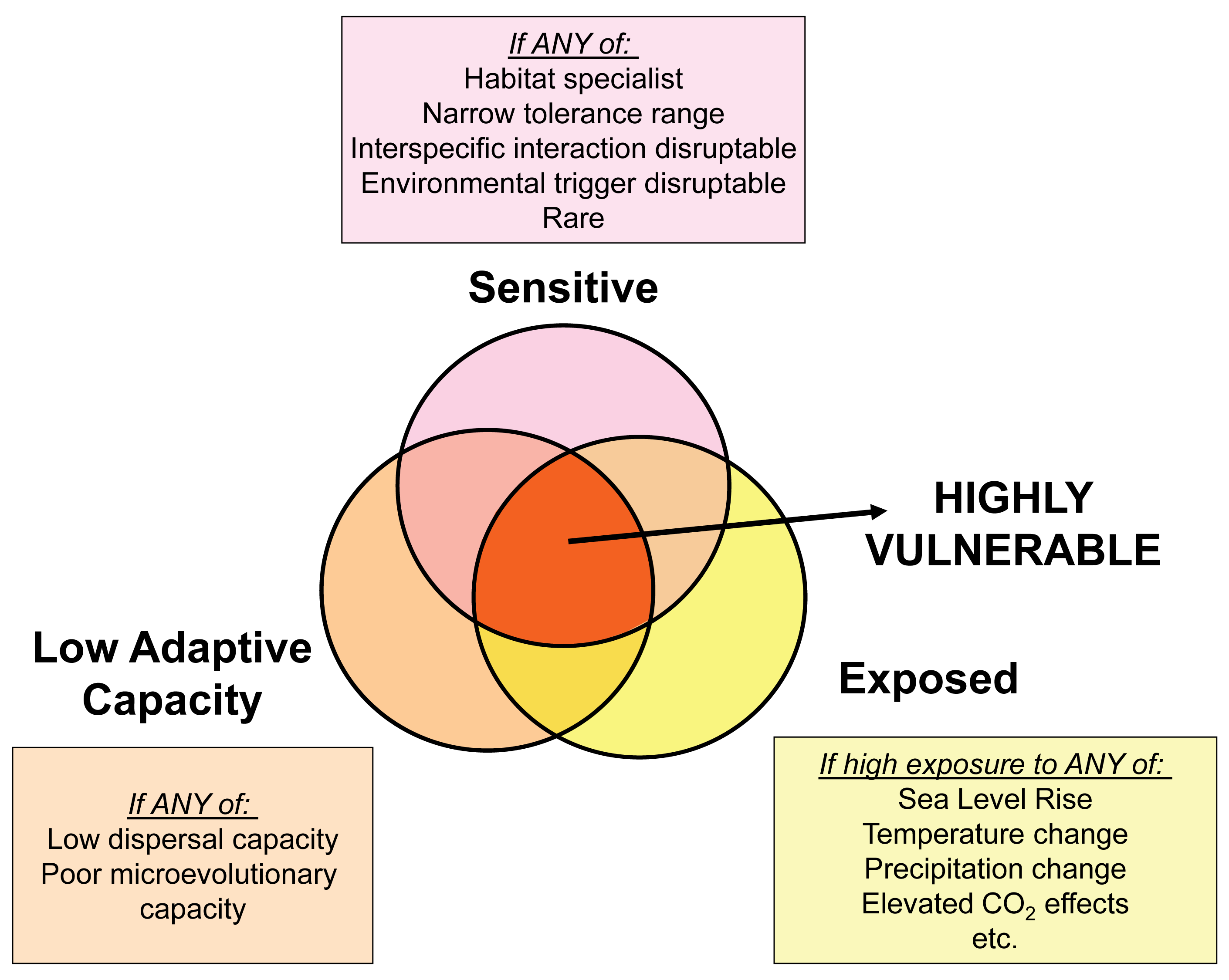

Supplement: Figure S13 — Schematic diagram showing the three dimensions of climate change vulnerability (sensitivity, exposure and low adaptive capacity) and the biological and environmental trait sets contributing to them. The three boxes explain the logic system used to classify species as high in each climate change vulnerability dimension. Species are considered highly climate change vulnerable overall if they score high under all three of sensitivity, exposure and low adaptive capacity. (TIF) [file pone.0065427.s013.tif]
