# Supplementary material for: Identifying the World's Most Climate Change Vulnerable Species: A Systematic Trait-Based Assessment of all Birds, Amphibians and Corals
Source: PLoS One. 2013 Jun 12;8(6):e65427. doi: 10.1371/journal.pone.0065427 (PMC3680427; doi:10.1371/journal.pone.0065427)
Supplement: Table S1 — Traits rendering bird species as of ‘high’ and ‘low/lower’ climate change vulnerability, and the number of species qualifying under these categories and as unknown according to each trait. (DOCX) [file pone.0065427.s014.docx]

### Table S1: Traits rendering bird species as of ‘high’ and ‘low/lower’ climate change vulnerability, and the number of species qualifying under these categories and as unknown according to each trait.

| **Trait Group** | **Trait** | **Not of high vulnerability** | | **High vulnerability** | | Unknown |
| --- | --- | --- | --- | --- | --- | --- |
|  |  | Threshold | No. species | Threshold | No. species | No. Species |
| **Sensitivity** | | | | | | |
| **a. Specialised habitat and/or microhabitat requirements** | Habitat specialist | Occurs in 2-24 habitats | 8,306 | Occurs in 1 habitat | 1,530 | 20 |
|  | Dependence on a particular microhabitat | No microhabitat dependency known | 8,855 | Has one or more microhabitat dependencies | 1,001 | 0 |
|  | Intolerance of disturbance (forest species) | Scored as 'Low' or 'Medium' or does not occur in forest | 7,277 | Scored as 'High' | 2,575 | 4 |
| **b. Narrow environmental tolerances or thresholds that are likely to be exceeded due to climate change at any stage in the life cycle** | Narrow temperature tolerance | Highest 75%: Average absolute deviation in temperature across the species' historical range > 1.44 ^o^C | 6,118 | Lowest 25%: Average absolute deviation in temperature across the species' historical range ≤ 1.44 ^o^C | 1,974 | 1,764 |
|  | Narrow precipitation tolerance | Highest 75%: Average absolute deviation in precipitation across the species' historical range (lowest 25%) **>** 46.32 mm | 5,997 | Lowest 25%: Average absolute deviation in precipitation across the species' historical range (lowest 25%) ≤ 46.32 mm | 2,095 | 1,764 |
| **d. Dependence on interspecific interactions which are likely to be disrupted by climate change** | Declining positive interactions with other species | No dependency | 9,767 | Dependence on one or more interspecific interactions that are likely to be impacted by climate change (e.g. specialised dependency on army ants) | 89 | 0 |
| **e. Rarity** | Small population size | ≥ 10,000 individuals | 2,319 | < 10,000 individuals | 1,084 | 6,453 |
|  | Small population size and heightened sensitivity to threatening processes | All other species | 1,993 | < 20,000 and [(skewed sex ratio)  OR (polygynous or polyandrous breeding system)  OR (cooperative breeding system)  OR (declining or extremely fluctuating population trend)] | 1,410 | 6,453 |
| **Total** |  | **719** |  | **6,290** |  | **2,847** |
| **Percentage** |  | **7%** |  | **64%** |  | **29%** |
| **Exposure** | | | | | | |
| **Sea level rise** | Habitat types exposed to sea level inundation | All other species | 9,673 | Occurs largely in inundation exposed coastal habitats and in no or only one other habitat type | 163 | 20 |
| **Changes in temperature** | Substantial changes in **mean temperature** occur across the species' range | Lowest 75%: Absolute difference between **(mean temperatures** across the species' range for all months) from 1975-2050 < 2.52 ^o^C | 6,066 | Highest 25%: Absolute difference between (**mean temperatures** across the species' range for all months) from 1975-2050 ≥ 2.52 ^o^C | 1,921 | 1,869 |
|  | Substantial changes in **temperature variability** across the species' range | Lowest 75%: Absolute difference between **(average absolute deviation in temperatures** across the species' range for all months) from 1975 to 2050 < 1.2 ^o^C | 6,062 | Highest 25%: Absolute difference between (**average absolute deviation in temperatures** across the species' range for all months) from 1975 to 2050 ≥1.2 ^o^C | 1,925 | 1,869 |
| **Changes in precipitation** | Substantial changes in **mean precipitation** occur across the species' range | Lowest 75%: Absolute ratio of change in (**mean precipitation** across the species' range for all months) from 1975 to 2050 < 0.49 | 5,989 | Highest 25%: Absolute ratio of change in **(mean precipitation** across the species' range for all months) from 1975 to 2050 ≥ 0.49 | 1,998 | 1,869 |
|  | Substantial changes in **precipitation variability** across the species' range | Lowest 75%: Absolute ratio of change in (**average absolute deviation in precipitation** across the species' range for all months) from 1975 to 2050 < 0.33 | 5,835 | Highest 25%: Absolute ratio of change in (**average absolute deviation in precipitation** across the species' range for all months) from 1975 to 2050 ≥ 0.33 | 2,152 | 1,869 |
| **Total** |  | **3,082** |  | **4,920** |  | **1,854** |
| **Percentage** |  | **31%** |  | **50%** |  | **19%** |
| **Low adaptive capacity** | | | | | | |
| **f. Poor dispersability** | Low intrinsic dispersal capacity | Maximum intrinsic dispersal distance > 1 km/year | 7,863 | Maximum intrinsic dispersal distance 0.5- 1 km/year | 1,993 | 0 |
|  | Extrinsic barriers to dispersal | No known barriers | 9,156 | Occurs exclusively on mountaintops, small islands and/or polar edges of land masses | 700 | 0 |
| **g. Poor evolvability** | Low genetic diversity | All other species | 9,787 | Evidence of low genetic diversity or known genetic bottleneck | 69 | 0 |
|  | Slow turnover of generations | Generation length < 6 years | 7,356 | Generation length ≥ 6 years | 2,500 | 0 |
|  | Low reproductive capacity | Mean clutch size >2 | 3,946 | Mean clutch size: ≤ 2 | 2,414 | 3,496 |
| **Total** |  | **2,507** |  | **5,337** |  | **2,012** |
| **Percentage** |  | **25%** |  | **54%** |  | **20%** |
