# Supplementary material for: Identifying the World's Most Climate Change Vulnerable Species: A Systematic Trait-Based Assessment of all Birds, Amphibians and Corals
Source: PLoS One. 2013 Jun 12;8(6):e65427. doi: 10.1371/journal.pone.0065427 (PMC3680427; doi:10.1371/journal.pone.0065427)
Supplement: Table S2 — Traits rendering amphibian species as of ‘high’ and ‘low/lower’ climate change vulnerability, and the number of species qualifying under these categories and as unknown according to each trait. (DOCX) [file pone.0065427.s015.docx]

### Table S2: Traits rendering amphibian species as of ‘high’ and ‘low/lower’ climate change vulnerability, and the number of species qualifying under these categories and as unknown according to each trait.

| **Trait Group** | **Trait** | **Not of high vulnerability** | | | **High vulnerability** | | Unknown | |
| --- | --- | --- | --- | --- | --- | --- | --- | --- |
|  |  | Threshold | | No. species | Threshold | No. species | No. species | |
| **Sensitivity** | | | | | | | | |
| **a. Specialised habitat and/or microhabitat requirements** | Habitat specialist | | Occurs in 2-33 habitats | 4,539 | Occurs in 1 habitat | 1,509 | 156 | |
|  | Dependence on a particular microhabitat | | All other species | 5,085 | Larval development and freshwater dependent and occurs exclusively in an unbuffered habitat (i.e. not forest) | 955 | 164 | |
| **b. Narrow environmental tolerances or thresholds that are likely to be exceeded due to climate change at any stage in the life cycle** | Narrow temperature tolerance (adults) | | Highest 75%: **Average absolute deviation** in temperature across the species' historical range > 1.20 ^o^C | 4,556 | Lowest 25%: **Average absolute deviation** in temperature across the species' historical range ≤1.20 ^o^C | 1,520 | 128 | |
|  | Narrow precipitation tolerance (adults) | | Highest 75%: **Average absolute deviation** in precipitation across the species' historical range > 45.84 mm | 4,557 | Lowest 25%: **Average absolute deviation** in precipitation across the species' historical range ≤ 45.84 mm | 1,519 | 128 | |
| **c. Dependence on a specific environmental trigger that’s likely to be disrupted by climate change** | Dependence on a specific environmental trigger that’s likely to be disrupted by climate change | | All other species | 4,113 | Explosive breeder on rainfall or increased water availability cue (not in forest) | 316 | 1,775 | |
| **d. Dependence on interspecific interactions which are likely to be disrupted by climate change** | Increasing negative interactions with other species | | All other species | 4,897 | (Chytridiomycosis related decline recorded) or (vulnerable to enigmatic decline) or (likely future infection (in a genus with a recorded infection and is freshwater dependent and in subtropical or tropical (forest, shrubland or grassland habitats)) | 1,307 | 0 | |
|  |  | | **1,365** |  | **4,453** |  | **386** | |
|  |  | | **22.00%** |  | **71.80%** |  | **6.20%** | |
| **Exposure** | | | | | | | | |
| **Sea level rise** | Habitat types exposed to sea level inundation | All other species | |  | Occurs largely in inundation exposed coastal habitats and in no or only one other habitat type | 4 | | 156 |
| **Changes in temperature** | Substantial changes in **mean temperature** occur across the species' range | Lowest 75%: Absolute difference between (**mean temperatures** across the species' range for all months) from 1975-2050 < 2.96 ^o^C | |  | Highest 25%: Absolute difference between (**mean temperatures** across the species' range for all months) from 1975-2050 ≥ 2.96 ^o^C | 1,515 | | 145 |
|  | Substantial changes in **temperature variability** across the species' range | Lowest 75%: Absolute difference between (**average absolute deviation in temperature** across the species' range for all months) from 1975 to 2050 < 1.93 ^o^C | |  | Highest 25%: Absolute difference between (**average absolute deviation in temperature** across the species' range for all months) from 1975 to 2050 ≥ 1.93 ^o^C | 1,515 | | 145 |
| **Changes in precipitation** | Substantial changes in **mean precipitation** occur across the species' range | Lowest 75%: Absolute ratio of change in (**mean precipitation** across the species' range for all months) from 1975 to 2050 < 0.59 | |  | Highest 25%: Absolute ratio of change in (**mean precipitation** across the species' range for all months) from 1975 to 2050 ≥ 0.59 | 1,515 | | 145 |
|  | Substantial changes in **precipitation variability** across the species' range | Lowest 75%: Absolute ratio of change in (**average absolute deviation in precipitation** across the species' range for all months) from 1975 to 2050 < 0.65 | |  | Highest 25%: Absolute ratio of change in (**average absolute deviation in precipitation** across the species' range for all months) from 1975 to 2050 ≥ 0.65 | 1,515 | | 145 |
|  |  | **2,642** | |  | **3,356** |  | | **206** |
|  |  | **42.60%** | |  | **54%** |  | | **3.30%** |
| **Low adaptive capacity** | | | | | | | | |
| **f. Poor dispersability** | Low intrinsic dispersal capacity | All other species | | 4,522 | Has not become established outside its natural range, and not associated with flowing water, and range size ≤ 4,000 km2 | 1,569 | | 113 |
|  | Extrinsic barriers to dispersal | All other species | | 3,900 | Occurs exclusively on mountaintops, small islands, polar edges of land masses and/or polar edges of suitable natural habitat | 745 | | 1,559 |
| **g. Poor Evolvability** | Low reproductive capacity | All other species | | 899 | Annual reproductive output ≤ 50 or viviparous | 2,073 | | 3,232 |
|  |  | **2,898** | |  | **3,233** |  | | **73** |
|  |  | **46.70%** | |  | **52.10%** |  | | **1.20%** |
