# Supplementary material for: Identifying the World's Most Climate Change Vulnerable Species: A Systematic Trait-Based Assessment of all Birds, Amphibians and Corals
Source: PLoS One. 2013 Jun 12;8(6):e65427. doi: 10.1371/journal.pone.0065427 (PMC3680427; doi:10.1371/journal.pone.0065427)
Supplement: Table S3 — Traits rendering coral species as of ‘high’ and ‘low/lower’ climate change vulnerability, and the number of species qualifying under these categories and as unknown according to each trait. (DOCX) [file pone.0065427.s016.docx]

### Table S3: Traits rendering coral species as of ‘high’ and ‘low/lower’ climate change vulnerability, and the number of species qualifying under these categories and as unknown according to each trait.

| **Trait Group** | **Trait** | **Not of high vulnerability** | | **High vulnerability** | | Unknown |
| --- | --- | --- | --- | --- | --- | --- |
|  |  | Threshold | No. species | Threshold | No. species | No. species |
| **Sensitivity** | | | | | | |
| **a. Specialised habitat and/or microhabitat requirements** | Habitat specialist | Occurs in 14-32 habitats | 605 | Occurs in <=13 habitats | 192 | 0 |
|  | Dependence on a particular microhabitat | Depth range > 14m | 570 | Depth range <= 14m | 192 | 35 |
| **b. Narrow environmental tolerances or thresholds that are likely to be exceeded due to climate change at any stage in the life cycle** | Narrow temperature tolerance - larvae | All other species | 658 | Broadcast spawning and/or brooding are the only known method(s) of reproduction | 137 | 2 |
|  | Evidence of exceedance of tolerance - adults | All other species | 475 | Evidence of past high temperature mortality of > 30% of local population on a reef or reef tract | 322 | 0 |
|  | Lower buffering from depth | Maximum depth ≥ 20m | 578 | Maximum depth < 20m | 188 | 31 |
| **d. Dependence on interspecific interactions which are likely to be disrupted by climate change** | Disruption of symbioses with Zooxanthellae algae | All other species | 58 | Obligate Zooxanthellae interaction and {(not known to have clades D, C1 or C15)  or (known to have D, C1 or C15 but not known to 'shuffle' Zooxanthellae)} | 738 | 1 |
| **e. Rarity** | Rarity | Not rare | 595 | Rare (geographically restricted or sparsely distributed) | 196 | 6 |
| **Total** |  | **1** |  | **796** |  | **0** |
| **Percentage** |  | **0.10%** |  | **99.90%** |  | **0.00%** |
| **Exposure** | | | | | | |
| **Temperature change** | Exposure to temperatures known to cause bleaching | **Lowest 75%:** Mean probability of severe bleaching across species' range (/10years) < 8.48 | 518 | **Highest 25%:** Mean probability of severe bleaching across species' range (/10years) ≥ 8.48 | 184 | 95 |
| **Elevated CO_2_** | Exposure to low aragonite saturation states | **Lowest 75%:** Proportion of species' range with aragonite saturation ≤3 by 2050 < 95.29% | 529 | **Highest 25%:** Proportion of species' range with aragonite saturation ≤ 3 by 2050 ≥ 95.29% | 177 | 91 |
| **Total** |  | **447** |  | **271** |  | **79** |
| **Percentage** |  | **56.10%** |  | **34.00%** |  | **9.90%** |
| **Low adaptive capacity** | | | | | | |
| **f. Poor dispersability** | Low intrinsic dispersal capacity | Maximum time to settlement of larvae > 14 days | 521 | Maximum time to settlement of larvae ≤ 14 days | 72 | 204 |
|  | Extrinsic barriers to dispersal | No known barriers | 669 | Dispersal likely to be retarded by currents and/or temperature | 117 | 11 |
| **g. Poor evolvability** | Slow turnover of generations | Typical colony longevity < 50 years | 771 | Typical colony longevity ≥ 50 years | 13 | 13 |
|  | Low growth rate | Typical maximum growth rate > 30 mm per year | 495 | Typical maximum growth rate ≤ 30 mm year | 293 | 9 |
| **Total** |  | **373** |  | **420** |  | **4** |
| **Percentage** |  | **46.80%** |  | **52.70%** |  | **0.50%** |
