# Supplementary material for: Identifying the World's Most Climate Change Vulnerable Species: A Systematic Trait-Based Assessment of all Birds, Amphibians and Corals
Source: PLoS One. 2013 Jun 12;8(6):e65427. doi: 10.1371/journal.pone.0065427 (PMC3680427; doi:10.1371/journal.pone.0065427)
Supplement: Table S4 — The number and percentage of bird, amphibian and coral families with significantly more and less highly climate change vulnerable species than expected from the observed overall frequency in each group (based on an optimistic scenario for missing data). (DOCX) [file pone.0065427.s017.docx]

### Table S4: The number and percentage of bird, amphibian and coral families with significantly more and less highly climate change vulnerable species than expected from the observed overall frequency in each group (based on an optimistic scenario for missing data).

| Group | No. of families (% highly vulnerable) | No. of families more vulnerable than expected (%) | No. of families less vulnerable than expected (%) | Number of families not different (%) |
| --- | --- | --- | --- | --- |
| Birds | 192 (2%) | 38 (20%) | 439 (20%) | 115 (60%) |
| Amphibians | 60 (22%) | 11 (18%) | 21 (35%) | 28 (47%) |
| Corals | 21 (15%) | 4 (19%) | 3 (14%) | 14 (67%) |
