# Supplementary material for: Identifying the World's Most Climate Change Vulnerable Species: A Systematic Trait-Based Assessment of all Birds, Amphibians and Corals
Source: PLoS One. 2013 Jun 12;8(6):e65427. doi: 10.1371/journal.pone.0065427 (PMC3680427; doi:10.1371/journal.pone.0065427)
Supplement: Table S5 — Summary of the 5 most and least climate change vulnerable bird families. Percentages represent the proportions of species qualifying as high under each climate change vulnerability dimension (i.e., sensitivity, exposure, low adaptive capacity and overall climate change vulnerability). Climate change vulnerability traits are listed where they characterise more than 25% of species in the family. (DOCX) [file pone.0065427.s018.docx]

### Table S5: Summary of the 5 most and least climate change vulnerable bird families. Percentages represent the proportions of species qualifying as high under each climate change vulnerability dimension (i.e., sensitivity, exposure, low adaptive capacity and overall climate change vulnerability). Climate change vulnerability traits are listed where they characterise more than 25% of species in the family.

| **BIRDS** | **No. of species in family** | **Mean Sensitivity** | **Mean Exposure** | **Mean Low Adaptive Capacity** | **Mean OVER-ALL Vulnera-bility** |
| --- | --- | --- | --- | --- | --- |
| **Five most vulnerable families** | | | | | |
| **Thamnophilidae**  (antbirds) | 210 | **87.1%**   - Habitat specialists - Microhabitat requirements - Forest dependent - Narrow temperature niches | **61.9%**  Facing relatively large changes in:   - Temperature variability - Precipitation variability | **79.1%**   - Short maximum dispersal distances - Slow turnover of generations | **47.1%** |
| **Trogonidae**  **(trogons)** | 40 | **100%**   - Microhabitat requirements - Forest dependent | **67.5%**  Facing relatively large changes in:   - Precipitation variability - Temperature variability - Mean precipitation | **100%**   - Short maximum dispersal distances - Slow turnover of generations | **67.5%** |
| **Bucerotidae**  **(hornbills)** | 55 | **100%**   - Microhabitat requirements - Forest dependent - Narrow temperature niches | **60.0%**  Facing relatively large changes in:   - Precipitation variability | **100%**   - Short maximum dispersal distances - Slow turnover of generations | **60.0%** |
| **Pipridae**  (manakins) | 54 | **90.7%**   - Forest dependent - Narrow temperature niches - Habitat specialists | **72.2%**  Facing relatively large changes in:   - Temperature variability - Precipitation variability | **92.6%**   - Short maximum dispersal distances - Slow turnover of generations | **59.2%** |
| **Trochilidae**  **(hummingbirds)** | 335 | **66.2%**   - Forest dependent | **76.7%**  Facing relatively large changes in:   - Temperature variability - Mean precipitation - Precipitation variability - Mean temperature | **71.9%**   - Short maximum dispersal distances - Slow turnover of generations | **36.7%** |
| **Five least vulnerable families** | | | | | |
| **Picidae**  (woodpeckers) | 218 | **79.4%**   - Forest dependence - Microhabitat requirements | **45.0%** | **13.3%** | **5.5%** |
| **Emberizidae**  (buntings, American sparrows and allies) | 317 | **64.7%**   - Narrow precipitation niches | **58.4%**  Facing relatively large changes in:   - Mean temperature - Temperature variability | **18.9%** | **9.2%** |
| **Estrildidae**  (waxbills, grass finches, munias and allies) | 137 | **29.9%** | **23.4%** | **7.3%** | **4.4%** |
| **Hirundinidae**  (swallows and martins) | 83 | **37.4%** | **43.4%** | **7.2%** | **1.2%** |
| **Cisticolidae**  (cisticolas and allies) | 114 | **36.0%** | **26.3%** | **29.8%** | **6.1%** |
