# Supplementary material for: Identifying the World's Most Climate Change Vulnerable Species: A Systematic Trait-Based Assessment of all Birds, Amphibians and Corals
Source: PLoS One. 2013 Jun 12;8(6):e65427. doi: 10.1371/journal.pone.0065427 (PMC3680427; doi:10.1371/journal.pone.0065427)
Supplement: Table S6 — Summary of the 5 most and least climate change vulnerable amphibian families. Percentages represent the proportions of species qualifying as high under each climate change vulnerability dimension (i.e., sensitivity, exposure, low adaptive capacity and overall climate change vulnerability). Climate change vulnerability traits are listed where they characterise more than 25% of species in the family. (DOCX) [file pone.0065427.s019.docx]

### Table S6: Summary of the 5 most and least climate change vulnerable amphibian families. Percentages represent the proportions of species qualifying as high under each climate change vulnerability dimension (i.e., sensitivity, exposure, low adaptive capacity and overall climate change vulnerability). Climate change vulnerability traits are listed where they characterise more than 25% of species in the family.

| **AMPHIBIANS** | | **No. of species in family** | **Mean Sensitivity** | **Mean Exposure** | **Mean Low Adaptive Capacity** | **Mean OVER-ALL Vulnera-bility** |
| --- | --- | --- | --- | --- | --- | --- |
| **Five most vulnerable families** | | | | | | |
| **Straboman-tidae**  (robber frogs) | 519 | | **82.1%**   - Habitat specialists - Narrow temperature niches - Narrow precipitation niches | **84.8%**  Facing relatively large changes in:   - Temperature variability - Mean temperature - Precipitation variability | **83.4%**   - Slow turnover of generations - Short maximum dispersal distances | **56.8%** |
| **Plethodontidae**  (lungless salamanders) | 371 | | **81.7%**   - Habitat specialists - Narrow precipitation niches | **56.9%**  Facing relatively large changes in:   - Precipiation variability - Mean temperature - Mean precipitation | **90.6%**   - Slow turnover of generations - Short maximum dispersal distances | **41.5%** |
| **Amphignatho-dontidae**  (marsupial frogs) | 61 | | **78.7%**   - Habitat specialists - Narrow precipitation niches | **82.0%**  Facing relatively large changes in:   - Temperature variability - Mean temperature - Mean precipitation | **91.8%**   - Slow turnover of generations - Short maximum dispersal distances | **63.9%** |
| **Aromobatidae**  (modern frogs) | 93 | | **88.2%**   - High disease vulnerability - Narrow temperature niches - Narrow precipitation niches - Habitat specialists | **86.0%**  Facing relatively large changes in:   - Mean precipitation - Precipitation variability - Temperature variability - Mean temperature | **60.2%**   - Slow turnover of generations | **48.4%** |
| **Eleuthero-dactylidae**  (robber frogs) | 186 | | **59.7%**   - Habitat specialists | **66.1%**  Facing relatively large changes in:   - Precipitation variability - Mean precipitation | **94.1%**   - Slow turnover of generations - Short maximum dispersal distances - Barriers to dispersal | **36.9%** |
| **Five least vulnerable families** | | | | | | |
| **Hylidae**  (tree frogs and their allies) | | 851 | **87.7%**  Disease vulnerability | **28.9%** | **47.6%**  Facing relatively large changes in:   - Mean precipitation | **11.5%** |
| **Ranidae**  (true frogs) | | 316 | **49.7%** | **22.8%** | **46.8%**  Facing relatively large changes in:   - Mean temperature | **6.7%** |
| **Hyperoliidae**  (African reed frogs) | | 210 | **83.8%**   - Freshwater dependent aquatic larvae - Narrow temperature niche | **54.3%**   - Short maximum dispersal distances | **18.6%** | **7.1%** |
| **Arthroleptidae**  (squeakers) | | 133 | **62.4%**   - Narrow temperature niche | **48.9%**   - Slow turnover of generations | **18.8%** | **4.5%** |
| **Dicroglossidae**  (forked tongued frogs) | | 163 | **45.4%** |  | **49.1%**  Facing relatively large changes in:   - Mean temperature | **6.8%** |
