# Supplementary material for: Identifying the World's Most Climate Change Vulnerable Species: A Systematic Trait-Based Assessment of all Birds, Amphibians and Corals
Source: PLoS One. 2013 Jun 12;8(6):e65427. doi: 10.1371/journal.pone.0065427 (PMC3680427; doi:10.1371/journal.pone.0065427)
Supplement: Table S7 — Summary of the four families that have mean climate change vulnerability scores that are significantly greater than the mean for all corals, as well as the three with significantly lower mean susceptibilities. Percentages represent the proportions of species qualifying as high under each climate change vulnerability dimension (i.e., sensitivity, exposure, low adaptive capacity and overall climate change vulnerability). Climate change vulnerability traits are listed where they characterise more than 25% of species in the family. (DOCX) [file pone.0065427.s020.docx]

### Table S7: Summary of the four families that have mean climate change vulnerability scores that are significantly greater than the mean for all corals, as well as the three with significantly lower mean susceptibilities. Percentages represent the proportions of species qualifying as high under each climate change vulnerability dimension (i.e., sensitivity, exposure, low adaptive capacity and overall climate change vulnerability). Climate change vulnerability traits are listed where they characterise more than 25% of species in the family.

| **CORALS** | **No. of species in family** | **Mean Sensitivity** | **Mean Exposure** | **Mean Low Adaptive Capacity** | **Mean OVER-ALL Vulnera-bility** |
| --- | --- | --- | --- | --- | --- |
| **Most vulnerable families** | | | | | |
| **Mussidae**  (including some brain corals) | 13 | **100%**   - Dependent on heat-intolerant Zooxanthellae | **36.0%**   - Facing relatively large increases in bleaching frequency | **98.0%**   - Very slow growth rate | **34.0%** |
| **Meandrinidae** | 7 | **100%**   - Dependent on heat-intolerant Zooxanthellae - Evidence of large-scale past mortality events in response to bleaching | **57.1**   - Facing relatively large increases in bleaching frequency | **100%**   - Very slow growth rate | **57.1%** |
| **Astrocoeniidae** | 13 | **100%**   - Dependent on heat-intolerant Zooxanthellae - Habitat specialists | **53.8%**   - Facing relatively large increases in bleaching frequency | **69.2%**   - Very slow growth rate - Slow turnover of generations | **38.5%** |
| **Faviidae**  (including stony corals) | 127 | **100%**   - Dependent on heat-intolerant Zooxanthellae - Evidence of large-scale past mortality events in response to bleaching - Larvae are sensitive to surface warming - Rare | **22.8%**   - Facing relatively large increases in bleaching frequency - Facing relatively large decreases in aragonite saturation | **88.2%**   - Very slow growth rate | **19.7%** |
| **Least vulnerable families** | | | | | |
| **Agariciidae** | 43 | **100%**   - Dependent on heat-intolerant Zooxanthellae - Evidence of large-scale past mortality events in response to bleaching - Habitat specialists | **32.6%**   - Facing relatively large increases in bleaching frequency | **20.9%** | **2.3%** |
| **Pectiniidae** | 27 | **100%**   - Dependent on heat-intolerant Zooxanthellae - Evidence of large-scale past mortality events in response to bleaching - Habitat specialists | **25.9%** | **18.5%**   - Very slow growth rate | **0%** |
| **Acroporidae**  (including staghorn corals) | 264 | **100%**   - Dependent on heat-intolerant Zooxanthellae - Evidence of large-scale past mortality events in response to bleaching - Unbuffered by broad depth ranges - Unbuffered by depth - Habitat specialists | **37.5%**   - Facing relatively large decreases in aragonite saturation | **40.2%**   - Short maximum dispersal distances | **12.9%** |
