# Supplementary material for: Identifying the World's Most Climate Change Vulnerable Species: A Systematic Trait-Based Assessment of all Birds, Amphibians and Corals
Source: PLoS One. 2013 Jun 12;8(6):e65427. doi: 10.1371/journal.pone.0065427 (PMC3680427; doi:10.1371/journal.pone.0065427)
Supplement: Table S8 — Summary of geographic focal areas (identified in Figure 2 (A, C, and E)) that contain high total numbers of species that are (i) highly sensitive and of low adaptive capacity, (ii) highly exposed, and both (i) and (ii). (DOCX) [file pone.0065427.s021.docx]

### Table S8: Summary of geographic focal areas (identified in Figure 2 (A, C and E)) that contain high total numbers of species that are (i) highly sensitive and of low adaptive capacity, (ii) highly exposed, and both (i) and (ii).

|  | **[High sensitivity and low adaptive capacity] and [high exposure]**  (purple in Fig. 2) | **High sensitivity and low adaptive capacity only**  (blue in Fig. 2) | **High exposure only**  (yellow in Fig. 2) |
| --- | --- | --- | --- |
| Birds | - Amazon basin and Mesoamerica, eastern Europe through central to eastern Asia, excluding the Tibetan Plateau - Congo basin and tropical West Africa - Himalayas - Malesia | - Northern and eastern North America - Southern oceans north to c.30^o^S - Australia and New Guinea - Tropical West Africa, Congo basin through sub-Saharan Africa and Madagascar - Southern and Eastern Brazil - Northern Eurasia | - Western USA and Mexico - North Africa and Sahel, excluding Sahara - Indian subcontinent through Indochina to north-eastern Asia |
| Amphibians | - Amazon basin | - Eastern USA - Tropical and southern Africa - Eastern and northern Australia | - South-western USA and Mexico - Northern South America Temperate Europe and north-western Asia - Indochina to north-eastern Asia - Madagascar |
| Corals | - Coral Triangle (Philippines to Solomon Islands), Sumatra and Java | - Australia - Pacific Islands - Indian Ocean including East Africa, Madagascar, India and Indochina - Red Sea - South China Sea |  |
