# Supplementary material for: Identifying the World's Most Climate Change Vulnerable Species: A Systematic Trait-Based Assessment of all Birds, Amphibians and Corals
Source: PLoS One. 2013 Jun 12;8(6):e65427. doi: 10.1371/journal.pone.0065427 (PMC3680427; doi:10.1371/journal.pone.0065427)
Supplement: Table S9 — Summary of geographic focal areas (identified in Figure 2 (B, D and F)) that contain high proportions of species, relative to species richness, that are (i) highly sensitive and of low adaptive capacity, (ii) highly exposed and both (i) and (ii). (DOCX) [file pone.0065427.s022.docx]

### Table S9: Summary of geographic focal areas (identified in Figure 2 (B, D and F)) that contain high proportions of species, relative to species richness, that are (i) highly sensitive and of low adaptive capacity, (ii) highly exposed and both (i) and (ii).

|  | **[High sensitivity and low adaptive capacity] and [high exposure]**  (purple in Fig. 2) | **High sensitivity and low adaptive capacity only**  (blue in Fig. 2) | **High exposure only**  (yellow in Fig. 2) |
| --- | --- | --- | --- |
| Birds | - Greenland, Iceland and north-eastern North America - Northern Eurasia, Black Sea and Himalayas - Southern oceans between c.30-60^o^S - Amazon basin and central Andes - Parts of the Eastern Sahara - Tropical West Africa to Congo basin - Sundaland | - The northern oceans, from c.45^o^N, extending southwards through the eastern Atlantic to West Africa - The Mediterranean and Red Seas and the Persian Gulf - Australia and parts of New Guinea - The southern oceans, from c. 25^o^S, extending northwards to the equator in the Eastern Pacific | - Africa, excluding tropical West Africa, the Congo basin and southern East Africa - Southern Eurasia and Arabia - Western USA through Central America - Northern South America and the Andes - New Zealand |
| Amphibians | - Amazon basin, northern Andes, and Mesoamerica - Eastern Russia and Mongolia - Himalayas - Parts of North Africa - North of the Caspian Sea - Western and eastern Arabia | - Congo basin, southern Africa and western Madagascar - Eastern North America - Eastern and southern South America - Most of Australia and New Guinea - Southern India and parts of Pakistan - The Iberian peninsula | - Northern Europe, Scandinavia and Asia to Malesia, excluding eastern Russia and Mongolia - Peripheral areas of the Sahara - Western North America - Central and southern Andes - Indonesia - South-western Pakistan and southern Iran |
| Corals | - The Caribbean (this region contains high proportions of highly exposed species, approximately half of which are also of high sensitivity and low adaptive capacity) | - All reefs from Red Sea and East Africa through Asia to central Pacific | - The Caribbean (this region contains high proportions of highly exposed species, approximately half of which are also of high sensitivity and low adaptive capacity) - East Atlantic coast and islands of Cape Verde, Sao Tome and Principe |
