# Supplementary material for: Identifying the World's Most Climate Change Vulnerable Species: A Systematic Trait-Based Assessment of all Birds, Amphibians and Corals
Source: PLoS One. 2013 Jun 12;8(6):e65427. doi: 10.1371/journal.pone.0065427 (PMC3680427; doi:10.1371/journal.pone.0065427)
Supplement: Table S10 — The numbers and percentages of birds, amphibians and coral species with various combinations of threat status (according to the IUCN Red List) and high climate change vulnerability. Optimistic scores are based on climate change vulnerability scores calculated on the assumption that unknown trait values reflect ‘not high’ scores; pessimistic scores are based on the assumption that unknown trait values reflect high scores. Independence between numbers of species that are threatened and highly climate change vulnerable was tested using Pearson’s Chi-square test (d.f. = 1); total species numbers (n), Chi-squared coefficients and P values are shown for each taxonomic group. (DOCX) [file pone.0065427.s023.docx]

### Table S10: The numbers and percentages of birds, amphibians and coral species with various combinations of threat status (according to the IUCN Red List) and high climate change vulnerability. Optimistic scores are based on climate change vulnerability scores calculated on the assumption that unknown trait values reflect ‘not high’ scores; pessimistic scores are based on the assumption that unknown trait values reflect high scores. Independence between numbers of species that are threatened by non-climatic stressors and highly climate change vulnerable was tested using Pearson’s Chi-square test (d.f. = 1); total species numbers (n), Chi-squared coefficients and P values are shown for each taxonomic group.

|  | **Birds** | | **Amphibians** | | **Corals** | |
| --- | --- | --- | --- | --- | --- | --- |
|  | Optimistic | Pessimistic | Optimistic | Pessimistic | Optimistic | Pessimistic |
| Vulnerable | *2,323* | *4,890* | *1,368* | *2,740* | *121* | *247* |
| Threatened | *1,222* | *1,222* | *1,878* | *1,878* | *220* | *220* |
| Threatened and vulnerable | **608** (6%) | **851** (9%) | **670** (11%) | **933** (15%) | **47** (6%) | **73** (9%) |
| Threatened and not vulnerable | **614** (6%) | **371** (4%) | **1,208** (19%) | **945** (15%) | **173** (22%) | **147** (18%) |
| Not threatened and vulnerable | **1,715** (17%) | **4,039** (41%) | **698** (11%) | **1,807** (29%) | **74** (9%) | **174** (22%) |
| Not threatened and not vulnerable | **6,919** (70%) | **4,595** (47%) | **3,628** (58%) | **2,519** (41%) | **503** (63%) | **403** (51%) |
| *Total species* | *9,856* | *9,856* | *6,204* | *6,204* | *797* | *797* |
| Chi-squared coefficient | 530.95 | 223.78 | 290.93 | 33.22 | 9.02 | 0.68 |
| Significance | P<0.001 | P<0.001 | P<0.001 | P<0.001 | P<0.01 | n.s. |
|  |  |  |  |  |  |  |
| What % of vulnerable species is threatened? | *26* | *17* | *49* | *34* | *39* | *30* |
| What % of vulnerable species is not threatened? | *74* | *83* | *51* | *66* | *61* | *70* |
| What % of threatened species is vulnerable? | *50* | *70* | *36* | *50* | *21* | *33* |
| What % of threatened species is not vulnerable? | *50* | *30* | *64* | *50* | *79* | *67* |
