# Supplementary material for: Identifying the World's Most Climate Change Vulnerable Species: A Systematic Trait-Based Assessment of all Birds, Amphibians and Corals
Source: PLoS One. 2013 Jun 12;8(6):e65427. doi: 10.1371/journal.pone.0065427 (PMC3680427; doi:10.1371/journal.pone.0065427)
Supplement: Table S11 — Summary of the geographic focal areas identified in Figure 3 that contain high total numbers of species that are threatened (according to the IUCN Red ListTM), climate change vulnerable and high numbers of both. (DOCX) [file pone.0065427.s024.docx]

**Table S11: Summary of the geographic focal areas identified in Figure 3 that contain high total numbers of species that are threatened** (according to the IUCN Red List^TM^), **highly climate change vulnerable and high numbers of both.**

|  | **Threatened and vulnerable**  (purple in Fig. 3) | **Vulnerable only**  (yellow in Fig. 3) | **Threatened only**  (blue in Fig. 3) |
| --- | --- | --- | --- |
| Birds | - Northern Andes - South-eastern South America - The Himalayas and Indian subcontinent - Sundaland (Indonesia, Malaysia and southern Thailand) and the Philippines - South eastern China - The southern oceans between c. 30-60^o^S - South eastern Russia, and parts of north-central Asia - Much of sub-Saharan Africa, excluding Congo basin - Parts of central and southern North America | - Amazon basin and south western South America - Parts of northern, western and southern North America - Central America - Europe, Scandinavia and much northern and central Asia - The Congo basin and parts of south-central Africa - Eastern and south western Australia - Guinea | - Throughout the northern and central Pacific - The southern Atlantic and Indian oceans from c. 20-30 ^o^S, and some areas south of 50^o^S - Western Arabian peninsula - Parts of central and South East Asia |
| Amphibians | - Parts of northern Andes and Mesoamerica | - Amazon basin - Southern USA to Mesoamerica - Eurasia excluding central Asia and eastern China - Madagascar - New Guinea - South-western Australia - The southern tip of Africa - Western Sahel - North-western Africa | - South-eastern China - Parts of western USA, Mexico Mesoamerica - Central Andes - Parts of tropical West Africa - Highlands of East Africa - South-eastern Australia |
| Corals | - Coral triangle, Sumatra and Java, extending northwards to the East China sea - The Red Sea - East Africa and central Indian ocean islands including Madagascar - The Great Barrier Reef and northern Australia | - Caribbean - Islands of the eastern Atlantic | - Arabian gulf - Northern Indian ocean (Bay of Bengal and Andaman sea) - The Pacific ocean, excluding the Coral Triangle |
