# Supplementary material for: Identifying the World's Most Climate Change Vulnerable Species: A Systematic Trait-Based Assessment of all Birds, Amphibians and Corals
Source: PLoS One. 2013 Jun 12;8(6):e65427. doi: 10.1371/journal.pone.0065427 (PMC3680427; doi:10.1371/journal.pone.0065427)
Supplement: Table S12 — Summary of the geographic focal areas identified in Figure S6 that contain high relative numbers of species that are threatened (according to the IUCN Red List), climate change vulnerable and high numbers of both. (DOCX) [file pone.0065427.s025.docx]

**Table S12: Summary of the geographic focal areas identified in Figure S6 that contain high relative numbers of species that are threatened** (according to the IUCN Red List^TM^), **climate change vulnerable, and high numbers of both.**

|  | **Threatened and vulnerable**  (purple in Suppl. Fig. 6) | **Vulnerable only**  (yellow in Suppl. Fig. 6) | **Threatened only**  (blue in Suppl. Fig. 6) |
| --- | --- | --- | --- |
| - Birds | - Oceans between c.30- 60^o^S - Northern Pacific - Peripheral areas of the Sahara - The eastern Mediterranean | - Amazon basin and Andes - Northern North America, extending to Greenland and Iceland - Europe and northern and central Eurasia, including the Himalayas - The Congo basin | - Northern and central Pacific - Western Atlantic and Caribbean and south-central and north-western Atlantic - Central Sahara - Arabian Peninsula - Indian subcontinent, extending to Indochina - Madagascar and central Indian Ocean - West-central Australia |
| - Amphibians | - Parts of northern Andes and Mesoamerica | - Amazon basin to Argentina - Parts of western and southern USA, Mexico and Mesoamerica - Eurasia excluding central Asia and eastern China - South-western Australia - North-western Africa | - Parts of western and southern USA, Mexico and Mesoamerica - The central Andes and parts of the southern Andes - Parts of tropical West Africa - Highlands of East Africa - Madagascar - South-eastern China - South-eastern Australia |
| - Corals |  | - Caribbean - East Atlantic coast and islands of Cape Verde, Sao Tome and Principe | - South-western Pacific and Australia - Coral Triangle - Indian Ocean |
