# Supplementary material for: Identifying the World's Most Climate Change Vulnerable Species: A Systematic Trait-Based Assessment of all Birds, Amphibians and Corals
Source: PLoS One. 2013 Jun 12;8(6):e65427. doi: 10.1371/journal.pone.0065427 (PMC3680427; doi:10.1371/journal.pone.0065427)
Supplement: Table S13 — Summary of the numbers of species and size of geographic area uniquely identified by each of the biological trait used to assess overall climate change vulnerability of birds. Traits highlighted in yellow identify the five most influential traits for uniquely identifying numbers of species and those in red text identify these traits for geographic areas. Trait and trait group descriptions are shortened versions; full titles are shown in Table S1. (DOCX) [file pone.0065427.s026.docx]

### Table S13: Summary of the numbers of species and size of geographic area uniquely identified by each of the biological traits used to assess overall climate change vulnerability of birds. Traits highlighted in yellow identify the five most influential traits for uniquely identifying numbers of species and those in red text identify these traits for geographic areas. Trait and trait group descriptions are shortened versions; full titles are shown in Table S1.

| **Vulner-ability dimension** | **Trait Group** | **Trait** | Species qualifying under this trait | Species qualifying exclusively based on this trait | % of total suscept-ible species | **Rank of import-ance by species** | Geographic area (km^2^) identified exclusively by this trait | % of total suscept-ible area | **Rank of import-ance by area** | Species with unknown score for this trait |
| --- | --- | --- | --- | --- | --- | --- | --- | --- | --- | --- |
| **Sensitivity** | Specialised habitat and/or microhabitat requirements | Habitat specialist | 1,530 | 19 | 0.8 | **14** | 93,964,466 | 17.7 | **9** | 20 |
|  |  | Microhabitat required | 1,001 | 56 | 2.4 | **11** | 94,788,888 | 17.8 | **8** | 0 |
|  |  | Intolerance of disturbance | 2,575 | 234 | 10.1 | **8** | 93,964,812 | 17.7 | **9** | 4 |
|  | Narrow environmental tolerances | Narrow temperature tolerance | 1,974 | 198 | 8.5 | **9** | 127,174,896 | 23.9 | **1** | 1,764 |
|  |  | Narrow precipitation tolerance | 2,095 | 0 | 0.0 | **16** | 93,963,082 | 17.7 | **9** | 1,764 |
|  | Interspecific interactions | Declining positive interactions with other species | 89 | 18 | 0.8 | **14** | 108,493,748 | 20.4 | **5** | 0 |
|  | Rarity | Small population size | 1,084 | 0 | 0.0 | **16** | 93,963,082 | 17.7 | **9** | 6,453 |
|  |  | Small effective population size | 1,410 | 24 | 1.0 | **13** | 94,248,845 | 17.7 | **9** | 6,453 |
| **Exposure** | Sea level rise | Exposed to sea level rise | 163 | 47 | 2.0 | **12** | 20,768,671 | 3.9 | **11** | 20 |
|  | Changes in temperature | Changes in mean temperature | 1,921 | 280 | 12.1 | **6** | 101,564,515 | 19.1 | **7** | 1,869 |
|  |  | Changes in temperature variability | 1,925 | 245 | 10.5 | **7** | 66,141,671 | 12.4 | **4** | 1,869 |
|  | Changes in precipitation | Changes in mean precipitation | 1,998 | 337 | 14.5 | **4** | 3,051,713 | 0.6 | **12** | 1,869 |
|  |  | Changes in precipitation variability | 2,152 | 298 | 12.8 | **5** | 62,813,190 | 11.8 | **10** | 1,869 |
| **Low adaptive capacity** | Poor dispersability | Limited dispersal ability | 1,993 | 524 | 22.6 | **1** | 106,611,380 | 20.1 | **6** | 0 |
|  |  | Geographical barriers | 700 | 112 | 4.8 | **10** | 109,168,370 | 20.5 | **3** | 0 |
|  | Poor evolvability | Low genetic diversity | 69 | 5 | 0.2 | **15** | 106,610,688 | 20.1 | **6** | 0 |
|  |  | Slow turnover of generations | 2,500 | 411 | 17.7 | **3** | 106,649,089 | 20.1 | **6** | 0 |
|  |  | Low reproductive output | 2,414 | 439 | 18.9 | **2** | 109,374,216 | 20.6 | **2** | 3,496 |
| **Total numbers of vulnerable species/area** | | |  | **2,323** |  |  | **531,435,729** |  |  |  |
| **Total number of species** | | |  | **9,856** |  |  |  |  |  |  |
