# Supplementary material for: Identifying the World's Most Climate Change Vulnerable Species: A Systematic Trait-Based Assessment of all Birds, Amphibians and Corals
Source: PLoS One. 2013 Jun 12;8(6):e65427. doi: 10.1371/journal.pone.0065427 (PMC3680427; doi:10.1371/journal.pone.0065427)
Supplement: Table S15 — Summary of the numbers of species and size of geographic area uniquely identified by each of the biological traits used to assess overall climate change vulnerability of corals. Traits highlighted in yellow identify the five most influential traits for uniquely identifying numbers of species and those in red text identify these traits for geographic areas. Trait and trait group descriptions are shortened versions; full titles are shown in Table S3. (DOCX) [file pone.0065427.s028.docx]

### Table S15: Summary of the numbers of species and size of geographic area uniquely identified by each of the biological traits used to assess overall climate change vulnerability of corals. Traits highlighted in yellow identify the five most influential traits for uniquely identifying numbers of species and those in red text identify these traits for geographic areas. Trait and trait group descriptions are shortened versions; full titles are shown in Table S3.

|  | **Trait Group** | **Trait** | Species qualify-ing under this trait | Species qualifying exclusively based on this trait | % of total vulnerable species | **Rank of import-ance by species** | Geographic area (km^2^) identified exclusively by this trait | % of total vulnerable area | **Rank of import-ance by area** | Species with unknown score for this trait |
| --- | --- | --- | --- | --- | --- | --- | --- | --- | --- | --- |
| **Sensitivity** | Specialised habitat and/or microhabitat | Habitat specialist | 192 | 2 | 1.7 | 6 | 0 | 0.0 | 7 | 0 |
|  |  | Microhabitat required | 192 | 0 | 0.0 | 8 | 0 | 0.0 | 7 | 35 |
|  | Narrow environmental tolerances | Narrow temperature tolerance - larvae | 137 | 2 | 1.7 | 6 | 0 | 0.0 | 7 | 2 |
|  |  | Buffering of temperature change | 188 | 0 | 0.0 | 8 | 0 | 0.0 | 7 | 31 |
|  |  | Evidence of bleaching | 322 | 20 | 16.5 | 5 | 22,487 | 0.6 | 4 | 0 |
|  | Interspecific interactions | No heat tolerant Zooxanthellae and/or not shufflers | 739 | 1 | 0.8 | 7 | 0 | 0.0 | 7 | 1 |
|  | Rarity | Lower ability to recover following declines | 196 | 0 | 0.0 | 8 | 0 | 0.0 | 7 | 6 |
| **Exposure** | Temperature change | Frequent exposure to temperatures known to cause bleaching | 184 | 52 | 43.0 | 2 | 583,980 | 16.6 | 1 | 95 |
|  | Ocean acidification | Exposure to low aragonite saturation states | 177 | 32 | 26.4 | 3 | 462,894 | 13.1 | 2 | 91 |
| **Low adaptive capacity** | Poor dispersability | Limited dispersal ability | 72 | 22 | 18.2 | 4 | 6,573 | 0.2 | 5 | 204 |
|  |  | Dispersal barriers | 117 | 22 | 18.2 | 4 | 4,152 | 0.1 | 6 | 11 |
|  | Poor evolvability | Slow turnover of generations | 13 | 1 | 0.8 | 7 | 0 | 0.0 | 7 | 11 |
|  |  | Slow growth rate | 293 | 56 | 46.3 | 1 | 83,030 | 2.4 | 3 | 9 |
| **Total numbers of vulnerable species/area** | | | | **121** | **15.2** |  | **3,521,181** |  |  |  |
| **Total number of species** | | | | **797** |  |  |  |  |  |  |
