# Supplementary material for: Identifying the World's Most Climate Change Vulnerable Species: A Systematic Trait-Based Assessment of all Birds, Amphibians and Corals
Source: PLoS One. 2013 Jun 12;8(6):e65427. doi: 10.1371/journal.pone.0065427 (PMC3680427; doi:10.1371/journal.pone.0065427)
Supplement: Table S16 — Traits rendering bird species as of ‘high’ climate change vulnerability, and the number of species qualifying under these categories and as unknown, according to three trait threshold scenarios, namely more lenient thresholds, the original or moderate thresholds (i.e., as used for the results presented in Table 2 and Figure 2 ) and stricter thresholds. Thresholds for traits indicated with a (P) and highlighted in blue were selected based on arbitrary percentage thresholds (35%, 25% and 15%) while those indicated by an (E) and highlighted in green were selected based on experts’ judgements. All results shown are based on an optimistic scenario for 2050 under the A1B emission scenario. (DOCX) [file pone.0065427.s029.docx]

### Table S16: Traits rendering bird species as of ‘high’ climate change vulnerability, and the number of species qualifying under these categories and as unknown, according to three trait threshold scenarios, namely more lenient thresholds, the original or moderate thresholds (i.e., as used for the results presented in Table 2 and Figure 2) and stricter thresholds. Thresholds for traits indicated with a (P) and highlighted in blue were selected based on arbitrary percentage thresholds (35%, 25% and 15%) while those indicated by an (E) and highlighted in green were selected based on experts’ judgements. All results shown are based on an optimistic scenario for 2050 under the A1B emission scenario.

| **Trait Group** | | **Trait** | **More Lenient Estimate** | | | **Original Estimate** | | **Stricter Estimate** | | Un- known |
| --- | --- | --- | --- | --- | --- | --- | --- | --- | --- | --- |
|  |  |  | Threshold | | No. spp. | Threshold | No. spp. | Threshold | No. spp. | No. spp. |
| **Sensitivity** | | | | | | | | | | |
| A. Specialised habitat and/or micro-habitat require-ments | Habitat specialist | | | NA | 1,530 | Occurs in 1 habitat | 1,530 | NA | 1,530 | 20 |
|  | Dependence on a particular microhabitat | | | NA | 1,001 | Has one or more microhabitat dependencies | 1,001 | NA | 1,001 | 0 |
|  | Intolerance of disturbance | | | NA | 2,575 | Scored as 'High' | 2,575 | NA | 2,575 | 4 |
| B. Narrow environ-mental tolerances | Narrow temperature tolerance **(P)** | | | Lowest 35%:   ≤ 1.83 ^o^C | 2,772 | Lowest 25%: Average absolute deviation in temperature across the species' historical range ≤ 1.44 ^o^C | 1,974 | Lowest 15%:   ≤ 1.13 ^o^C | 1,182 | 1,764 |
|  | Narrow precipitation tolerance **(P)** | | | Lowest 35%:   ≤ 56.09 mm | 2,904 | Lowest 25%: Average absolute deviation in precipitation across the species' historical range ≤ 46.32 mm | 2,095 | Lowest 15%:  ≤ 35.23 mm | 1,279 | 1,764 |
| D. Depen-dence on inter-specific inter-actions | Declining positive interactions with other species | | | NA | 89 | Dependence on one or more interspecific interactions that are likely to be impacted by climate change (e.g. specialised dependency on army ants) | 89 | NA | 89 | 0 |
| E. Rarity | Small population size **(P)** | | | < 20,000 individuals | 1,410 | < 10,000 individuals | 1,084 | < 2,500 individuals | 477 | 6,453 |
|  | Small population size and heightened sensitivity to threatening processes **(E)** | | | **< 50,000** and [(skewed sex ratio) | 1,423 | **< 20,000** and [(skewed sex ratio) | 1,410 | **< 10,000** and [(skewed sex ratio) | 869 | 6,453 |
|  |  |  |  | OR (polygynous or polyandrous breeding system) |  | OR (polygynous or polyandrous breeding system) |  | OR (polygynous or polyandrous breeding system) |  |  |
|  |  |  |  | OR (co-operative breeding system) |  | OR (cooperative breeding system) |  | OR (co-operative breeding system) |  |  |
|  |  |  |  | OR (declining or extremely fluctuating population trend)] |  | OR (declining or extremely fluctuating population trend)] |  | OR (declining or extremely fluctuating popn trend)] |  |  |
| **Low adaptive capacity** | | | | | | | | | | |
| A. Poor dispers-ability | | Low intrinsic dispersal capacity **(E)** | Maximum intrinsic dispersal distance <=1.5 km/year | | 2,628 | Maximum intrinsic dispersal distance <= 1 km/year | 1,993 | Maximum intrinsic dispersal distance <= 0.5 km/year | 1,510 | 0 |
|  |  | Extrinsic barriers to dispersal | NA | | 700 | Occurs exclusively on mountaintops, small islands and/or polar edges of land masses | 700 | NA | 700 | 0 |
| B. Poor evolva-bility | | Low genetic diversity | NA | | 69 | Evidence of low genetic diversity or known genetic bottleneck | 69 | NA | 69 | 0 |
|  |  | Slow turnover of generations **(P)** | Generation length ≥ 5.7 years | | 2,809 | Generation length ≥ 6 years | 2,500 | Generation length ≥ 8 years | 1,196 | 0 |
|  |  | Low reproductive capacity **(P)** | Mean clutch size: ≤ 2.5 | | 3,288 | Mean clutch size: ≤ 2 | 2,414 | Mean clutch size: ≤ 1.5 | 749 | 3,496 |
| **Exposure** | | | | | | | | | | |
| A. Sea level rise | | Habitat types exposed to sea level inundation **(E)** | Occurs largely in inundation exposed coastal habitats and **up to 2 other habitat types** | | 425 | Occurs largely in inundation exposed coastal habitats **and up to 1 other habitat type** | 163 | Occurs in inundation exposed coastal habitats only | 42 | 20 |
| B. Changes in temp-erature | | Substantial changes in **mean temper-ature** occur across the species' range **(P)** | Highest 35%:   ≥ 2.1 ^o^C | | 2,735 | Highest 25%: Absolute difference between (**mean temperatures** across the species' range for all months) from 1975-2050 ≥ 2.5 ^o^C | 1,921 | Highest 15%:   ≥ 3.1 ^o^C | 1,097 | 1,869 |
|  |  | Substantial changes in **temper-ature variability** across the species' range **(P)** | Highest 35%:  ≥ 1.0 ^o^C | | 2,668 | Highest 25%: Absolute difference between (**average absolute deviation in temperatures** across the species' range for all months) from 1975 to 2050 ≥ 1.2 ^o^C | 1,925 | Highest 15%:  ≥ 1.6 ^o^C | 1,144 | 1,869 |
| C. Changes in precip-itation | | Substantial changes in **mean precip-itation** occur across the species' range **(P)** | Highest 35%:  ≥ 0.38 | | 2,738 | Highest 25%: Absolute ratio of change in **(mean precipitation** across the species' range for all months) from 1975 to 2050 ≥ 0.49 | 1,998 | Highest 15%:  ≥ 0.61 | 1,233 | 1,869 |
|  |  | Substantial changes in **precip-itation variability** across the species' range **(P)** | Highest 35%:  ≥ 0.23 | | 2,959 | Highest 25%: Absolute ratio of change in (**average absolute deviation in precipitation** across the species' range for all months) from 1975 to 2050 ≥ 0.33 | 2,152 | Highest 15%:   ≥ 0.52 | 1,351 | 1,869 |
