# Supplementary material for: Identifying the World's Most Climate Change Vulnerable Species: A Systematic Trait-Based Assessment of all Birds, Amphibians and Corals
Source: PLoS One. 2013 Jun 12;8(6):e65427. doi: 10.1371/journal.pone.0065427 (PMC3680427; doi:10.1371/journal.pone.0065427)
Supplement: Table S17 — Traits rendering amphibian species as of ‘high’ climate change vulnerability, and the number of species qualifying under these categories and as unknown, according to three trait threshold scenarios, namely more lenient thresholds, the original or moderate thresholds (i.e., as used for the results presented in Table 2 and Figure 2 ) and stricter thresholds. Thresholds for traits indicated with a (P) and highlighted in blue were selected based on arbitrary percentage thresholds (35%, 25% and 15%) while those indicated by an (E) and highlighted in green were selected based on experts’ judgements. All results shown are based on an optimistic scenario for 2050 under the A1B emission scenario. (DOCX) [file pone.0065427.s030.docx]

### Table S17: Traits rendering amphibian species as of ‘high’ climate change vulnerability, and the number of species qualifying under these categories and as unknown, according to three trait threshold scenarios, namely more lenient thresholds, the original or moderate thresholds (i.e., as used for the results presented in Table 2 and Figure 2) and stricter thresholds. Thresholds for traits indicated with a (P) and highlighted in blue were selected based on arbitrary percentage thresholds (35%, 25% and 15%) while those indicated by an (E) and highlighted in green were selected based on experts’ judgements. All results shown are based on an optimistic scenario for 2050 under the A1B emission scenario.

| **Trait Group** | **Trait** | **More Lenient Estimate** | | | | **Original Estimate** | | | | | **Stricter Estimate** | | | Un- known |
| --- | --- | --- | --- | --- | --- | --- | --- | --- | --- | --- | --- | --- | --- | --- |
|  |  | Threshold | | No. spp. | | Threshold | | | No. spp. | | Threshold | No. spp. | | No. spp. |
| **Sensitivity** | | | | | | | | | | | | | | |
| **A. Special-ised habitat and/or micro-habitat require-ments** | Habitat specialist | NA | | 1,509 | | Occurs in only 1 habitat | | | 1,509 | | NA | | 1,509 | 156 |
|  | Dependence on a particular micro-habitat **(E)** | Larval development and freshwater dependent | | 3,948 | | Larval development and freshwater dependent and occurs exclusively in an unbuffered habitat (i.e. not in forest) | | | 955 | | NA | | 955 | 156 |
| **B. Narrow environ-mental tolerances or thres-holds** | Narrow temperature tolerance **(P)** | Lowest 35%:  ≤1.50 ^o^C | | 2,129 | | Lowest 25%: **Average absolute deviation** in temperature across the species' historical range ≤1.20 ^o^C | | | 1,520 | | Lowest 15%:   ≤0.86 ^o^C | | 911 | 128 |
|  | Narrow precipitation tolerance **(P)** | Lowest 35%:   ≤ 56.00 mm | | 2,127 | | Lowest 25%: **Average absolute deviation** in precipitation across the species' historical range ≤ 45.84 mm | | | 1,519 | | Lowest 15%:   ≤ 34.05 mm | | 911 | 128 |
| **C. Depend-ence on a specific environ-mental trigger** | Dependence on a specific environmental trigger that’s likely to be disrupted by climate change | NA | | 316 | | Explosive breeder on rainfall or increased water availability cue (not in forest) | | | 316 | | NA | | 316 | 1,775 |
| **D. Depend-ence on inter-specific inter-actions** | Increasing negative interactions with other species **(E)** | (Chytridio-mycosis related decline recorded) or (vulnerable to enigmatic decline) or (likely future infection (in a genus with a recorded infection) | | 2,719 | | (Chytridiomycosis related decline recorded) or (vulnerable to enigmatic decline) or (likely future infection (in a genus with a recorded infection and is freshwater dependent and in subtropical or tropical (forest, shrubland or grassland habitats)) | | | 1,307 | | (Chytrid-iomycosis related decline recorded) or (vulnerable to enigmatic decline) | | 595 | 0 |
| **Low adaptive capacity** | | | | | | | | | | | | | | |
| **A. Poor dispers-ability** | Low intrinsic dispersal capacity **(E)** | | Has not become established outside its natural range, and  not associated with flowing water, and  range size ≤ 8,000 km2 | | 1,768 | | Has not become established outside its natural range, and not associated with flowing water, and range size ≤ 4,000 km2 | 1,569 | | Has not become established outside its natural range, and not associated with flowing water, and  range size ≤ 2,000 km2 | | | 1,382 | 113 |
|  | Extrinsic barriers to dispersal | | NA | | 745 | | Occurs exclusively on mountaintops, small islands, polar edges of land masses and/or polar edges of suitable natural habitat | 745 | | NA | | | 745 | 1,559 |
| **B. Poor evolva-bility** | Low reproductive capacity **(E)** | | Annual reproductive output ≤ 100 or viviparous | | 2,081 | | Annual reproductive output <= 50 or viviparous | 2,073 | | Annual reproductive output ≤ 25 or viviparous | | | 62 | 3,232 |
| **Exposure** | | | | | | | | | | | | | | |
| **A. Sea level rise** | Habitat types exposed to sea level inundation **(E)** | | Occurs in inundation exposed coastal habitats and up to 2 other habitat types | | 5 | | Occurs largely in inundation exposed coastal habitats and up to 1 other habitat type | 4 | | Occurs in inundation exposed coastal habitats only | | | 2 | 156 |
| **B. Changes in temper-ature** | Substantial changes in **mean temperature** occur across the species' range **(P)** | | Highest 35%:   >= 2.4 ^o^C | | 2,121 | | Highest 25%: Absolute difference between (**mean temperatures** across the species' range for all months) from 1975-2050 ≥ 3.0 ^o^C | 1,515 | | Highest 15%:  >= 4.1 ^o^C | | | 908 | 145 |
|  | Substantial changes in **temperature variability** across the species' range **(P)** | | Highest 35%:   >= 1.5 ^o^C | | 2,121 | | Highest 25%: Absolute difference between (**average absolute deviation in temperature** across the species' range for all months) from 1975 to 2050 ≥ 1.9 ^o^C | 1,515 | | Highest 15%:   >= 2.8 ^o^C | | | 908 | 145 |
| **C. Changes in precip-itation** | Substantial changes in **mean precipitation** occur across the species' range **(P)** | | Highest 35%: Absolute ratio of change in (**mean precipitation** across the species' range for all months) from 1975 to 2050 >= 0.5 | | 2,121 | | Highest 25%: Absolute ratio of change in (**mean precipitation** across the species' range for all months) from 1975 to 2050 ≥ 0.6 | 1,515 | | Highest 15%: Absolute ratio of change in (**mean precipitation** across the species' range for all months) from 1975 to 2050 >= 0.8 | | | 908 | 145 |
|  | Substantial changes in **precipitation variability** across the species' range **(P)** | | Highest 35%:   >= 0.5 | | 2,121 | | Highest 25%: Absolute ratio of change in (**average absolute deviation in precipitation** across the species' range for all months) from 1975 to 2050 ≥ 0.7 | 1,515 | | Highest 15%: >= 1.0 | | | 908 | 145 |
