# Supplementary material for: Identifying the World's Most Climate Change Vulnerable Species: A Systematic Trait-Based Assessment of all Birds, Amphibians and Corals
Source: PLoS One. 2013 Jun 12;8(6):e65427. doi: 10.1371/journal.pone.0065427 (PMC3680427; doi:10.1371/journal.pone.0065427)
Supplement: Table S18 — Traits rendering coral species as of ‘high’ climate change vulnerability, and the number of species qualifying under these categories and as unknown, according to three trait threshold scenarios, namely more lenient thresholds, the original or moderate thresholds (i.e., as used for the results presented in Table 2 and Figure 2 ) and stricter thresholds. Thresholds for traits indicated with a (P) and highlighted in blue were selected based on arbitrary percentage thresholds (35%, 25% and 15%) while those indicated by an (E) and highlighted in green were selected based on experts’ judgements. All results shown are based on an optimistic scenario for 2050 under the A1B emission scenario. (DOCX) [file pone.0065427.s031.docx]

### Table S18: Traits rendering coral species as of ‘high’ climate change vulnerability, and the number of species qualifying under these categories and as unknown, according to three trait threshold scenarios, namely more lenient thresholds, the original or moderate thresholds (i.e., as used for the results presented in Table 2 and Figure 2) and stricter thresholds. Thresholds for traits indicated with a (P) and highlighted in blue were selected based on arbitrary percentage thresholds (35%, 25% and 15%) while those indicated by an (E) and highlighted in green were selected based on experts’ judgements. All results shown are based on an optimistic scenario for 2050 under the A1B emission scenario.

| **Trait Group** |  | **More Lenient Estimate** | | **Original Estimate** | | **Stricter Estimate** | | Un-known |
| --- | --- | --- | --- | --- | --- | --- | --- | --- |
|  | **Trait** | Threshold | No. spp. | Threshold | No. spp. | Threshold | No. spp. | No. spp. |
| **Sensitivity** | | | | | | | | |
| **A. Special-ised habitat and/or micro-habitat** | Habitat specialist **(P)** | Occurs in <=16 habitats | 270 | Occurs in <=13 habitats | 192 | Occurs in <=10 habitats | 113 | 0 |
|  | Microhabitat dependence **(P)** | Depth range <= 16m | 261 | Depth range <= 14m | 192 | Depth range <= 11m | 108 | 35 |
| **B. Narrow environ-mental tolerances or thresholds** | Narrow temperature tolerance - larvae | NA | 137 | Broadcast spawning and/or brooding are the only known method(s) of reproduction | 137 | NA | 137 | 2 |
|  | Evidence of exceedance of tolerance - adults | NA | 322 | Evidence of past high temperature mortality of > 30% of local population on a reef or reef tract | 322 | NA | 322 | 0 |
|  | Lower buffering from depth **(P)** | Maximum depth < 21m | 393 | Maximum depth < 20m | 188 | Maximum depth < 15 m | 76 | 31 |
| **D. Depend-ence on inter-specific inter-actions** | Disruption of symbioses with Zooxan-thellae algae **(E)** | NA | 738 | Obligate Zooxanthellae interaction AND {(not known to have clades D, C1 or C15)  **OR**  (known to have D, C1 or C15 but not known to 'shuffle' Zooxanthellae)} | 738 | Obligate Zooxanthellae interaction AND {(not known to have clades D, C1 or C15)  **AND**  (known to have D, C1 or C15 but not known to 'shuffle' Zooxanthellae)} | 585 | 1 |
| **E. Rarity** | Rarity | NA | 196 | Rare (geographically restricted or sparsely distributed) | 196 | NA | 196 | 6 |
| **Low adaptive capacity** | | | | | | | | |
| **A. Poor dispersa-bility** | Low intrinsic dispersal capacity **(E)** | Maximum time to settlement of larvae ≤ 30 days | 350 | Maximum time to settlement of larvae ≤ 14 days | 72 | NA (no smaller data classes available) | 72 | 204 |
|  | Extrinsic barriers to dispersal | NA | 117 | Dispersal likely to be retarded by currents and/or temperature | 117 | NA | 117 | 11 |
| **B. Poor evolva-bility** | Slow turnover of generations **(P)** | Typical colony longevity ≥ 10 years | 773 | Typical colony longevity ≥ 50 years | 13 | NA (no larger data classes available) | 13 | 13 |
|  | Low growth rate **(P)** | Typical maximum growth rate ≤ 100 mm year | 620 | Typical maximum growth rate ≤ 30 mm per year | 293 | Typical maximum growth rate ≤ 10 mm per year | 91 | 9 |
| **Exposure** | | | | | | | | |
| **A. Temper-ature change** | Exposure to temperatures known to cause bleaching **(P)** | **Highest 35%:**  ≥ 8.35 | 250 | **Highest 25%:** Mean probability of severe bleaching across species' range (/10years) ≥ 8.48 | 184 | **Highest 15%:**  ≥ 8.59 | 105 | 95 |
| **B. Elevated CO_2_** | Exposure to low aragonite saturation states **(P)** | **Highest 35%:**  ≥ 93.32% | 247 | **Highest 25%:** Proportion of species' range with aragonite saturation ≤ 3 by 2050 ≥ 95.29% | 177 | **Highest 15%: >=**96.75% | 107 | 91 |
