# Supplementary material for: Identifying the World's Most Climate Change Vulnerable Species: A Systematic Trait-Based Assessment of all Birds, Amphibians and Corals
Source: PLoS One. 2013 Jun 12;8(6):e65427. doi: 10.1371/journal.pone.0065427 (PMC3680427; doi:10.1371/journal.pone.0065427)
Supplement: Table S21 — Summary of the potential impacts of sources of uncertainty on numbers of climate change vulnerable coral species. These include scenarios of impacts of missing data (unknowns), the choice of percentage thresholds, the selection of thresholds by experts, the greenhouse gas emission scenario applied and the time frames considered. Percentages represent the numbers of climate change vulnerable species relative to the total number of species. Except where specified, assessments are based on optimistic unknowns scenario under emissions scenario A1B for 2050. (DOCX) [file pone.0065427.s034.docx]

### Table S21: Summary of the potential impacts of sources of uncertainty on numbers of climate change vulnerable coral species. These include scenarios of impacts of missing data (unknowns), the choice of percentage thresholds, the selection of thresholds by experts, the greenhouse gas emission scenario applied and the time frames considered. Percentages represent the numbers of vulnerable species relative to the total number of species. Emissions scenarios and time frame results presented are for terrestrial regions only. Except where specified, assessments are based on optimistic unknowns scenario under emissions scenario A1B for 2050.

|  |  | Low/Lenient | ***%*** | Mid | ***%*** | High/Strict | ***%*** |
| --- | --- | --- | --- | --- | --- | --- | --- |
| Numbers of vulnerable species  *(% of total species)* | Unknowns  (pessimistic - optimistic) | 2,285 | ***23*** | 121 | ***15*** | - | ***-*** |
|  | Percent thresholds  (35%-25%-15%) | 354 | ***44*** | 121 | ***15*** | 59 | ***7*** |
|  | Expert thresholds | 145 | ***18*** | 121 | ***15*** | 121 | ***15*** |
|  | Emissions Scenarios (B1-A1B-A2) | 12 | ***2*** | 121 | ***15*** | 69 | ***9*** |
|  | Time frames  (2090-2050) | 341 | ***43*** | 121 | ***15*** | - | ***-*** |
