# Supplementary material for: Identifying the World's Most Climate Change Vulnerable Species: A Systematic Trait-Based Assessment of all Birds, Amphibians and Corals
Source: PLoS One. 2013 Jun 12;8(6):e65427. doi: 10.1371/journal.pone.0065427 (PMC3680427; doi:10.1371/journal.pone.0065427)
Supplement: Supporting Information S1 — Supporting Methods, Supporting Discussion, Figures S1–13, Tables S1–21 and Supporting Information References. (PDF) [file pone.0065427.s035.pdf]

Supporting Information

Identifying the World's Most Climate Change Vulnerable Species:

A Systematic Trait-Based Assessment of all Birds, Amphibians and Corals

Foden et al. 2013 (PLOS ONE)

Table of Contents

Supporting Methods 5

The Vulnerability Framework 5

Determining the trait sets 5

Sensitivity 6

Exposure 7

Low adaptive capacity 8

Selecting appropriate traits and assigning scores 9

Using the framework for birds, amphibians and corals 11

Taxonomy and baseline databases 11

Preparing maps of species' distribution ranges 11

Birds 13

Amphibians 17

Corals 19

Plotting areas of greatest concentrations of vulnerable species 25

Analyses 26

Comparisons between species' climate change vulnerability and their IUCN Red List threat statuses 26

Calculating numbers of vulnerable species under different emissions scenarios and time frames 26

Assessing the influence of each trait on overall vulnerability 27

Assessing the influence of trait thresholds and other sources of uncertainty on overall vulnerability 29

Supporting Discussion 31

Opportunities for validation of the framework 31

Caveats and uncertainty 32

Supporting Figures 34

S1: The relationship between climate change vulnerability dimensions for families containing ten or more species 34

S2: Geographic concentrations of **bird** species that are highly sensitive, exposed, have low adaptive capacity and are highly climate change vulnerable overall 35

S3: Geographic concentrations of **amphibian** species that are highly sensitive, exposed, have low adaptive capacity and are highly climate change vulnerable overall 36

S4: Geographic concentrations of **coral** species that are highly sensitive, exposed, have low adaptive capacity and are highly climate change vulnerable overall 38

S5: Geographic concentrations of species that are highly vulnerable under a pessimistic scenario but not under an optimistic scenario for **birds**, **amphibians** and **corals** 39

S6: Bivariate plots showing areas with highest logged proportions of species that are climate change vulnerable only, threatened only, and both highly climate change vulnerable and threatened 40

S7: Foci of highly climate change vulnerable **birds** under three IPCC SRES climate change scenarios for 2050 and 2090....41

S8: Foci of highly climate change vulnerable **amphibians** under three IPCC SRES climate change scenarios for 2050 and 2090....42

S9: Foci of highly climate change vulnerable **corals** under three IPCC SRES climate change scenarios for 2050 and 2090....43

S10: Foci of highly climate change vulnerable **birds** calculated using five trait threshold scenarios....44

S11: Foci of highly climate change vulnerable **amphibians** calculated using five trait threshold scenarios....45

S12: Foci of highly climate change vulnerable **corals** calculated using five trait threshold scenarios....46

S13: Schematic diagram showing the three dimensions of climate change vulnerability (sensitivity, exposure and low adaptive capacity) and the biological and environmental trait sets contributing to them.. 47

Supporting Tables

S1: Traits rendering **bird** species as of 'high' and 'low/lower' climate change vulnerability, and the number of species qualifying under these categories and as unknown according to each trait. 48

S2: Traits rendering **amphibian** species as of 'high' and 'low/lower' climate change vulnerability, and the number of species qualifying under these categories and as unknown according to each trait. 51

S3: Traits rendering **coral** species as of 'high' and 'low/lower' climate change vulnerability, and the number of species qualifying under these categories and as unknown according to each trait. 54

S4: The number and percentage of **bird**, **amphibian** and **coral** families with significantly more and less highly climate change vulnerable species than expected 56

S5: Summary of the 5 most and least climate change vulnerable **bird** families....57

S6: Summary of the 5 most and least climate change vulnerable **amphibian** families. ...59

S7: Summary of the four families that have mean climate change vulnerability scores that are significantly greater than the mean for all **corals**, as well as the three with significantly lower mean susceptibilities. 61

S8: Summary of geographic focal areas (identified in Figure 2 (A, C and E)) that contain high **total numbers** of species that are highly sensitive and of low adaptive capacity, highly exposed, and both 63

S9: Summary of geographic focal areas (identified in Figure 2 (B, D and F)) that contain **high proportions** of species, relative to species richness, that are highly sensitive and of low adaptive capacity, highly exposed and both 64

S10: The numbers and percentages of **bird**, **amphibian** and **coral** species with various combinations of threat status and high climate change vulnerability....65

S11: Summary of the geographic focal areas identified in Figure 3 that contain high **total numbers** of species that are threatened, highly climate change vulnerable and high numbers of both. 66

S12: Summary of the geographic focal areas identified in Figure S6 that contain high **relative numbers** of species that are threatened, climate change vulnerable, and high numbers of both. 67

S13: Summary of the numbers of species and size of geographic area uniquely identified by each of the biological traits used to assess overall climate change vulnerability of **birds**.. 68

S14: Summary of the numbers of species and size of geographic area uniquely identified by each of the biological traits used to assess overall climate change vulnerability of **amphibians**. 69

S15: Summary of the numbers of species and size of geographic area uniquely identified by each of the biological traits used to assess overall climate change vulnerability of **corals**. 70

S16: Traits rendering **bird** species as of 'high' climate change vulnerability, and the number of species qualifying under these categories and as unknown, according to three trait threshold scenarios. 71

S17: Traits rendering **amphibian** species as of 'high' climate change vulnerability, and the number of species qualifying under these categories and as unknown, according to three trait threshold scenarios. 74

S18: Traits rendering **coral** species as of 'high' climate change vulnerability, and the number of species qualifying under these categories and as unknown, according to three trait threshold scenarios. 77

S19: Summary of the potential impacts of sources of uncertainty on numbers of climate change vulnerable **bird** species. 79

S20: Summary of the potential impacts of sources of uncertainty on numbers of climate change vulnerable **amphibian** species. 80

S21: Summary of the potential impacts of sources of uncertainty on numbers of climate change vulnerable **coral** species. 81

Supporting Information References 82

Appendix A: Climate change vulnerability scores by species for birds

Appendix B: Climate change vulnerability scores by species for amphibians

Appendix C: Climate change vulnerability scores by species for corals

Supporting Methods

The Vulnerability Framework

Vulnerability or vulnerability frameworks are used in a variety of contexts to support decision making to address risks to the most vulnerable members of a system to change [1]. The three dimensions that make up vulnerability are usually described as sensitivity (the lack of potential for a species to persist *in situ)*, exposure (the extent to which each species' physical environment will change) and low adaptive capacity (a species' inability to avoid the negative impacts of climate change through dispersal and/or micro-evolutionary change). This general idea has been suggested for assessing species risks from climate change [2] and is used in some case studies (e.g., [3–5]).

A body of work already exists on biological traits associated with vulnerability to extinction due to historic threatening processes [6–9]. Because climate change poses a new threat, there is little empirical information with which to assess vulnerability, except in a limited way for species that were exposed to relatively rapid climate shifts during the quaternary [10] and rare case studies [11–13]. Instead, we used literature review, expert opinion based on expectations from ecological and evolutionary theory to identify traits associated with each dimension of climate change vulnerability (hereafter referred to simply as vulnerability).

Determining the trait sets

Through two workshops and various other consultations, we gathered input from over 30 scientists whose collective expertise covers a broad range of taxonomic groups and ecosystems (see Supporting Discussion, 'Caveats and uncertainty', point 1). Together with extensive literature survey, this process identified more than 90 biological traits that may be associated with species' vulnerability to climate change. The traits were consolidated, firstly according to the three dimensions of vulnerability i.e., sensitivity, exposure and low adaptive capacity, and subsequently into five 'trait sets' for sensitivity, a variable number for exposure, and two for low adaptive capacity, as outlined below.

Sensitivity

In the vulnerability framework context, sensitivity is regarded as the lack of potential for a species to persist *in situ).* Here we describe five components of sensitivity, termed ‘trait groups’ (adapted from [14]).

A. **Specialised habitat and/or microhabitat requirements**: Across many studies of both animals and plants, threatened and declining species include a disproportionate number of specialists compared to generalists and of species with extensive geographic ranges [15]. Under a changing climate, most species are likely to face changes in their habitats and microhabitats and those less tightly coupled to specific conditions and requirements are likely to be more resilient. Sensitivity is increased where a species has several life stages, each with different habitat or microhabitat requirements (e.g. water-dependent larval amphibians), or when the habitat or microhabitat to which the species is specialized is particularly vulnerable to climate change impacts (e.g. mangroves, cloud forests or polar habitats). However, in some cases (e.g. deep sea fishes), extreme specialization may allow species to escape the full impacts of competition from native or invading species, so the interaction of such traits with climate change must be considered carefully for each species group assessed. This trait group is not independent of species’ low adaptive capacity as habitat and/or microhabitat specialisation also decreases the chances of successful colonisation if species are able to disperse to new climatically suitable areas, (e.g., plants confined to limestone outcrops; cave-roosting bats).

B. **Narrow environmental tolerances or thresholds that are likely to be exceeded due to climate change at any stage in the life cycle**: The physiology and ecology of many species is tightly coupled to very specific ranges of climatic variables such as temperature, precipitation, pH and carbon dioxide levels, and those with narrow tolerance ranges are particularly vulnerable to climate [16]. Even species with broad environmental tolerances and unspecialized habitat requirements may already be close to thresholds beyond which ecological or physiological function quickly breaks down (e.g., photosynthesis in plants; protein and enzyme function in animals).

C. **Dependence on a specific environmental trigger that is likely to be disrupted by climate change:** Many species rely on environmental triggers or cues for migration, breeding, egg laying, seed germination, hibernation, spring emergence, and a range of other essential processes. While some cues such as day length and lunar cycles will be unaffected by climate change, others such as rainfall and temperature (including their

interacting and cumulative effects) may be severely impacted. Species tend to become vulnerable to changes in the magnitude and timing of these cues when this leads to an uncoupling with resources or other essential ecological processes e.g., early spring warming causes the emergence of a species before its food sources are available. Climate change vulnerability is compounded when different stages of a species' life history or different sexes rely on different cues.

D. **Dependence on interspecific interactions which are likely to be disrupted by climate change:** Many species' interactions with prey, hosts, symbionts, pathogens and competitors will be affected by climate change, either due to the decline or loss of these resource species from the dependent species' ranges or loss of synchronization in phenology. Species dependent on interactions that are vulnerable to disruption by climate change are at risk of extinction, particularly where they have high degree of specialization for the particular resource species and are unlikely to be able to switch to or substitute other species.

E. **Rarity:** The inherent vulnerability of small populations to allee effects and catastrophic events, as well as their generally reduced capacity to recover quickly following local extinction events, suggest that many rare species will face greater impacts from climate change than more common and/or widespread species. We consider rare species to be those with small population sizes and those that may be abundant but are geographically highly restricted. In cases where only a small proportion of individuals reproduce (e.g., species with polygynous or polyandrous breeding systems or skewed sex ratios), we use an estimate of effective population size to assess species' rarity, and where species are known to be declining or subject to extreme (greater than ten-fold) fluctuations in population size, we set less conservative population size thresholds. Similarly, thresholds of larger population sizes were used for species with congregatory breeding systems, since they are more likely to experience catastrophic population declines.

Exposure

These measures reflect the climate change driven environmental pressures on species, based on their geographic locations. For the main results of our study, we consider projected changes in four pressures by 2050, though other pressures, their combinations and alternative time frames could also be used.

A. **Sea level rise:** Although no global projections of sea level rise are currently available, regional models or surrogate measures such as occurrence in coastal habitat types can be used to assess species' likelihood of threat from sea water inundation due to rising sea levels.

B. **Temperature change:** Projections of temperature change are typically based on General Circulation Model outputs and interpreted based on the ecosystem occupied by the focal species group (e.g., air temperature for amphibians, sea surface temperature for corals). Biologically relevant components of temperature change typically include changes in means, variability and/or extremes (magnitude and frequency).

C. **Precipitation change**: As for temperature changes, these are typically based on General Circulation Model outputs and biologically relevant components may include changes in means, variability and/or extremes (magnitude and frequency).

D. **Elevated atmospheric CO_2 impacts:** While not strictly a climate change phenomenon, we consider this otherwise overlooked potential threat in the general suit of climate-change related impacts. Both direct impacts of elevated CO_2 levels and resulting ocean acidification (e.g., on corals), and indirect impacts (e.g., through changes in competitive relationships between C_3 and C_4 plants) should be considered. Aquatic species are likely to be affected by increased CO_2 absorption by water bodies, the effects of which are projected to be particularly marked in marine ecosystems where ocean acidification and the resulting lowering of calcite and aragonite saturation levels lead to reduced growth and dissolution of organisms with calcium-carbonate exoskeletons or plates, including corals, coccolithophore algae, coralline algae, foraminifera, shellfish and pteropods [17].

Low adaptive capacity

This set of traits reflects the extent to which species have the capacity to reduce the impacts of changes in their immediate environment through dispersal or adaptive change. We define two low adaptive capacity 'trait groups' (adapted from [14]):

A. **Poor dispersability:** In general, the particular set of environmental conditions to which each species is adapted will shift to increasing latitudes and altitudes in response to climate change. Species with low rates or short distances of dispersal (e.g., land snails, ant and rain drop splash dispersed plants) are unlikely to migrate fast enough to keep up with these shifting climatic envelopes and will face increasing extinction risk as their

habitats become exposed to progressively greater climatic changes. Even where species could disperse to newly suitable areas, extrinsic barriers may decrease changes of dispersal success. Dispersal barriers may be geographic features such as unsuitable elevations (e.g., species confined to mountain ranges), oceans (e.g., for species on small islands or at the polar tip of a land mass), rivers, and for marine species, ocean currents and temperature gradients; unsuitable habitats and/or anthropogenic transformation may also act as dispersal barriers for habitat specialised species. In this context we describe species as having dispersal barriers both when suitable areas exist but extrinsic factors make them unlikely to reach them, as well as when no newly suitable areas are likely to exist (e.g., for polar species).

B. Poor evolvability: Species' potential for rapid genetic change will determine whether they will be able to undergo evolutionary adaptation at a rate sufficient to keep up with climate driven changes to their environments. Species with low genetic diversity, often indicated by recent bottlenecks in population numbers, potentially face inbreeding depression and generally exhibit lower ranges of both phenotypic and genotypic variation. As a result, such species tend to have fewer novel characteristics that could facilitate adaptation to the new climatic conditions. Where they exist, direct measures of genetic variability can be supplemented with information on naturalization outside species' native ranges and on the success of any past translocation efforts. Indirect measures of evolvability relate to the speed and output of reproduction and hence the rate at which advantageous novel genotypes could accumulate in populations and species [18]. Evidence suggests that evolutionary adaptation is possible in relatively short time frames (e.g. 5 to 30 years [19]) but for most species with long life cycles (e.g., large animals and many perennial plants), such adaptation is unlikely to keep up with the rate of climate driven changes to their environments.

Selecting appropriate traits and assigning scores

Guided by the trait groups described above, we conducted a second round of expert consultation and through consensus we compiled biological, ecological, physiological and environmental traits that are pertinent for assessing the particular climate change vulnerability of each taxonomic group. The traits selected for birds, amphibians and corals are shown in Tables S1-3 and are discussed in the next section; except for sensitivity trait group C (dependence on environmental triggers or cues) for birds, we were able to represent

each of the trait groups with at least one trait for each taxonomic group. Challenges in selecting traits included balancing selection of the most theoretically sound traits with the practicalities of data availability and collection. A further challenge was defining traits in objective and replicable ways and, as far as possible, developing quantitative measures for them.

Species were assigned scores of ‘high’, ‘low/lower’ or ‘unknown’ for each trait, based on a broad range of information sources (discussed below). While in some cases, thresholds of extinction risk were clear (e.g., ‘occurs only on mountain tops’), in most cases there is no *a priori* basis for setting a particular extinction risk threshold. For such traits (e.g., projected temperature change exposure), we arbitrarily selected a threshold of the worst affected 25% of species; those ranked in this group were scored ‘high’, while the remaining species were assigned scores of ‘lower’, or ‘unknown’ where data were lacking.

Data on, for example, population sizes, temperature-tolerance thresholds and inter-species interactions, were particularly sparse, necessitating frequent scores of ‘unknown’ for corresponding species. In some cases where empirical data were unavailable, experts were able to provide information either from unpublished data, their own field knowledge or, where justified, through inference from similar species. For our study, measures of experts’ confidence in the data were recorded in most cases, and data that were regarded as particularly uncertain were treated as ‘unknown’ values in subsequent stages of assessment.

To qualify as highly vulnerable overall, species must have high scores for *all* three of vulnerability dimensions of sensitivity, exposure and low adaptive capacity. A species scored high under sensitivity if any of the several biological traits in sensitivity trait groups scored high; similarly low adaptive capacity and high exposure were triggered if *any single* trait in these groups was listed as high (see Fig. S13 for a schematic summary of the logic used to assign species’ scores). Uncertainty at the level of unknown trait data is accounted for by calculating scores assuming all unknowns represent high scores (pessimistic scenario) and as ‘not high’ scores (optimistic scenario) and presenting overall vulnerability results as the range of possible values between these extremes.

Using the framework for birds, amphibians and corals

Taxonomy and baseline databases

The list of bird species followed BirdLife International (2008), as used by the 2008 IUCN Red List. For amphibians, we followed the taxonomy in Amphibian Species of the World (http://research.amnh.org/vz/herpetology/amphibia/, 2008). Coral species lists were based on the warm-water reef-building corals assessed for the Global Marine Conservation Assessment [20] (obtained from the IUCN Red List), but we excluded 46 species due to unresolved taxonomic problems, and incorporated taxonomic updates to 2010. Although not intended to be a definitive taxonomic source, the IUCN Red List strives to be taxonomically coherent and consistent at all ranks. Higher-level classification follows accepted classifications, but deviates in some respects; further information is available at http://www.iucnredlist.org/technical-documents/information-sources-and-quality. The IUCN Red List (http://www.iucnredlist.org/), BirdLife International's World Bird Database, and AmphibiaWeb (http://amphibiaweb.org/) provided essential information such as distribution maps, habitats and threats, and additional information was gathered from published and unpublished data, online resources, literature and expert knowledge. Where justifiable, we addressed data gaps with experts' inferences and assumptions, though many remain.

Preparing maps of species' distribution ranges

Bioclimatic modelling traditionally relies on the availability of detailed information on points of occurrence (and ideally absence) to 'train' statistical models about focal species' climatic 'requirements' or correlates. The intensive data requirements of these methods limit their application to few taxa and geographical regions, and prevent systematic global-scale assessments. Instead we derive an estimation of species' exposure to climate change by calculating simple metrics of climatic change across refined species' ranges. Species' ranges for birds, amphibians and warm-water reef-building corals have been mapped by experts as part of IUCN Red List assessments and are available at http://www.iucnredlist.org/technical-documents/spatial-data; range polygons were available for 81% of birds, 98% of amphibians and 99.9% of corals at the time this component of our analyses was carried out. Range polygons were compiled from a combination of known localities and extrapolation of areas within them that have been assessed by experts as suitable. They represent best estimates of each species' current limits within its historical native range (any introductions are coded accordingly and were excluded from this analysis), but we note that some species will almost

certainly occur more or less widely than mapped. Understudied regions include the Andes, most of Central Africa, parts of West Africa, Angola, parts of South and Southeast Asia, and Melanesia [21]. As a result, the biodiversity and potential climate change vulnerability in these regions will be under represented in this study. We also note that, although some of our analyses assume homogeneity of species within distribution ranges, this is unlikely to be the case for most species.

Because IUCN Red List range maps are often generalised polygons, they frequently represent species' Extents of Occurrence (calculated by drawing a polygon around all known places that a species occurs) and thus may include areas not actually occupied by the species and for which climate projections differ. For example, a range polygon may have been drawn around a lowland amphibian's occupied range on either side of a mountain range, or similarly around a coral's range on either side of an ocean. To refine species' ranges for our assessments, we excluded unsuitable within-range habitats and, for terrestrial species, elevations in which the species is known not to occur.

To facilitate processing of the large volume of range data involved, we rasterised range maps at a resolution of 10 minutes (~20x20 km); this is believed to be the scale at which the poorest resolution maps are reliable for each of the three taxonomic groups assessed. A species was regarded as 'present' in a 10 minute grid cell if any part of the underlying range polygon was occupied. For corals, areas of unsuitable habitat were defined as those where any 10 minute grid cell failed to intersected with a coral reef, as defined by ReefBase's global dataset of coral reef locations (www.reefbase.org).

For birds and amphibians, this process was more complex. Habitat affiliations (based on 126 IUCN Red List (2009) habitat categories which include natural and human-transformed habitats in terrestrial, freshwater and marine ecosystems) were obtained from the IUCN Red List database and BirdLife's World Bird Database (and are based on published literature and experts' knowledge), but as these habitat types are not spatially explicit, we cross-referenced them to the Global Land Cover 2000 habitat types (23 categories, including natural and human-transformed habitats and water bodies at1x1 km resolution; http://ies.jrc.ec.europa.eu/global-land-cover-2000), as guided by available literature [22,23]. We aimed to remove only habitats for which we had high confidence of their unsuitability, so we included all expert-listed IUCN habitat types (i.e. those described as 'suitable', 'marginal', or of unknown suitability) and removed none where habitat preferences are not known. We cross-referenced each IUCN habitat type with any potentially similar Global Land Cover 2000 habitat types (e.g., any IUCN forest type triggered all Global Land Cover

2000 forest types). The 1x1 km Global Land Cover 2000 was rasterised into twenty-three 10 minute grids, each representing one of the Global Land Cover 2000 types. For each grid, cells' values represented the percentage of the underlying 1x1 km vector covered by the land cover type in question. The probability of the presence of suitable habitat in each cell of a species' range was calculated as the sum of the percentage presence of all such suitable habitat types; again following a conservative approach, we excluded only cells with zero probability of suitable habitat.

To exclude areas with unsuitable elevations for terrestrial species' ranges, we again used IUCN Red List information on species' individual elevation preferences, comparing these with the U.S. Geological Survey's GTOPO30 global digital elevation model (http://eros.usgs.gov/#/Find_Data/Products_and_Data_Available/gtopo30_info). Species' elevation ranges were buffered to a minimum of 100 metres, and for those with unknown ranges, all elevations were included. The 1x1 km GTOPO30 elevation dataset was rasterised to two 10 minute grids, one containing the maximum elevation and one the minimum value in the underlying vector data. To determine elevation suitability in the cell, we calculated the extent to which each species' elevation range lies between the minimum and maximum elevation for the cell; following the conservative approach, we excluded from species' ranges only cells with no overlap between the species' and cell's elevation ranges.

Birds

The traits, biological information and thresholds used to assess birds' vulnerability are summarised in Table S1.

Sensitivity

The degree of birds' habitat and microhabitat specialisation was estimated using three traits. The first, termed **habitat specialisation,** is based on the number of habitats listed in the IUCN Red List (2009) as of major importance (defined as where the habitat is suitable and furthermore is important for the survival of the species, either because it has an absolute requirement for the habitat at some point in its life cycle e.g., for breeding or as a critical food source, or it is the primary habitat or one of two primary habitats within which the species usually occurs or within which most individuals occur), suitable (the species occurs in the habitat regularly or frequently), or as marginal (the species occurs in the habitat only irregularly or infrequently, or only a small proportion of individuals are found in the habitat). Birds for which only a single habitat was recorded were assessed as of high sensitivity.

Secondly, species were considered **dependent on specialised microhabitats** if they have a particular requirement for one or more of the following microhabitats: bamboo, vines, tree falls, dead wood, tree hollows [24–27], rocky outcrops in forests, caves, streams and bromeliads. Lastly, as more detailed data were available on species' dependence on forest specifically, they were assessed as of high sensitivity if they are **unable to tolerate forest disturbance**. Intolerance was categorised as high for forest specialists characteristic of the interior of undisturbed forest, but that may persist in secondary forest and forest patches if their particular ecological requirements are met. Where such species do occur away from the interior, they are usually less common and are rarely seen in non-forest habitats, and breeding is almost invariably within forest. Species with 'Medium' intolerance are forest generalists that may occur in undisturbed forest but are also regularly found in forest strips, edges and gaps, and tend to be commoner in such situations and in secondary forest than in the interior of intact forest, and breeding is typically within forest. Species with 'Low' intolerance are often recorded in forest, but are not dependent on it, and are almost always more common in non-forest habitats where most individuals breed. Intolerance was coded as unknown for species that occur or probably occur in forest but for which their degree of dependency on it is unknown.

Because empirical evidence of bird species' **environmental tolerances** is sparse, we use the range of historical temperatures and precipitation levels tolerated by the species across its historical range as a proxy**.** Based on the Worldclim global dataset's 1950-2000 monthly means for terrestrial areas (excluding Antarctica) at 10 minute resolution [28] (http://www.worldclim.org), we calculated the average absolute deviation across all cells in each species' refined range, for each of the 1975 (mean 1950-2000) monthly means, producing two measures, one for precipitation and one for temperature, that represent tolerance of variability both seasonally and spatially. The average absolute deviation (AAD) is a summary statistic of dispersion, and, for a data set {x1, x2, ..., xn}, AAD is defined [29] as:

$$\frac{1}{n}\sum_{i=1}^{n}|x_i - m(X)|.$$

In our calculations, each x represents a monthly mean for a cell in a species' refined range. Species were ranked according to their AAD scores and the 25% with the narrowest values for temperature and/or precipitation were regarded as of highest sensitivity.

In a minority of cases (3.9% of birds e.g., for marine and Antarctic birds and some small island species), less than 80% of species' ranges were covered by the Worldclim dataset. As a result, we also calculated all species' rankings based on modelled HadCM3 global projections (supplied by the U.K. Meteorology Office) for 1975 (mean 1961-1990), downscaled to 10 minutes using a cubic spline; for species with <80% of their ranges covered above, exposure scores were based on these rankings, and thresholds for AAD temperature and precipitation were 1.24 ^{o}C and 44.02 mm respectively.

Species with high dependence on very few (typically <5) species of ants, termites, insects, bees or wasps were assessed as having high vulnerability to potentially **declining positive interactions with other species**. Rare species were defined as those with estimated total **population sizes** (from BirdLife's World Bird Database) of fewer than 10,000 individuals, or those where the total population size numbers less than 20,000 and **sensitivity to threatening processes is heightened** due to skewed sex ratios (males to female ratio of ≤0.4 or ≥0.6), polygynous or polyandrous breeding systems, cooperative breeding systems, or declining or extremely fluctuating populations (fluctuations >10-fold). Cooperative breeding systems include lekking, as well as those that regularly or seasonally congregate at particular sites, and then disperse over a wide area. It also includes species that breed colonially (e.g., Southern Royal Albatross), congregate during migration (e.g., European Honey-buzzard) or during the non-breeding season (e.g., Snow Goose). At least 1% of the global population must be found at one or more sites to qualify, and hence this excludes species that congregate to breed, feed or move in numbers that are small relative to the global population (e.g., Little Swift).

Exposure

Since no global projections of sea level rise are available, we used habitat types as a proxy for high exposure to sea level rise impacts. Mangroves, intertidal salt marshes, coastal freshwater, brackish or saline lakes and lagoons, marine lakes, coastal caves, intertidal shorelines (including rocks, beaches, flats and tide pools), sea cliffs, rocky offshore islands, and coastal sand dunes were regarded as at high risk from sea level rise. Species were considered to have high exposure if they occur exclusively in one or more of these habitats (with the habitat ranked as suitable or of major importance for the species), or in these and only one other habitat.

To estimate which species will be most exposed to future changes in temperature, we calculated, firstly, the absolute difference between mean projected historical temperatures across each species' range in 1975 (1961-1990 average of mean annual temperature) and the

mean projected temperature across the same range for 2050 (2041-2060 average of mean annual temperature). Secondly, to incorporate projected changes in temperature variability, we calculated the absolute difference in projected AAD (i.e. a measure of variability across all cells and months; see above section on determining environmental tolerances to assess sensitivity for birds for details) between 1975 (based on mean monthly temperatures from 1961-1990) and 2050 (mean monthly temperatures for 2041-2060). For precipitation, we first calculated the ratio of absolute change in projected precipitation means between 1975 (1961-1990 average of mean annual precipitation) and 2050 (2041-2060 average of mean annual temperature) precipitation means (**absolute [((precip in 2050)-(precip in 1975)/(precip in 1975)])**, and secondly, the ratio of projected absolute change between 1975 (based on mean monthly precipitation from 1961-1990) and 2050 (based on mean monthly temperatures from 2041-2060) AAD (**absolute [((AAD in 2050)-(AAD in 1975)/(AAD in 1975)])**. Species were then ranked under each of these four exposure measures and those within the 25% greatest projected change by 2050 for any were scored as of high exposure.

Because we are not attempting to project species' future ranges but rather to examine projected climatic anomalies within existing ranges, we used General Circulation Model (GCM) projections for both historical and future climates; this avoids the incorrect attribution of method-caused differences between the two global datasets to climatic forcing. To model climate change exposure, we used the mean of outputs from four General Circulation Models, namely UKMO HadCM3, MPIM ECHAM5, CSIRO MK3.5 and GFDL CM2.1, for 1975 (mean 1961-1990) and 2050 (mean 2041-2060). These model outputs are available for terrestrial areas excluding Antarctica and some small islands, and are downscaled to 10 minute resolution according to methods described in Tabor (2010) [30] (available for download from http://ccr.aos.wisc.edu/model/ipcc10min/). For main results, we based exposure on the moderate SRES A1B greenhouse gas emissions scenario [31]. Under section 'Calculating numbers of vulnerable species under different emissions scenarios and time frames' below, we compare these results (based on A1B for 1975-2050) with those for A2 (high) and B1 (low) emissions scenarios, and for 1975-2090.

The coarse scale of original General Circulation Model (GCM) projections results in a poor match with the fine-scale coastal boundaries used to map species. Additionally, the dataset above does not include marine areas or Antarctica. We found that 10.4% of birds, namely those restricted to small oceanic islands, with narrow coastal distributions, in Antarctica or with largely marine ranges, had less than 80% of their ranges covered by the above GCM model ensemble. As a result, we also calculated all species' rankings based on HadCM3

projections for scenario A1B (supplied by the U.K. Meteorology Office) for 1975 (mean 1961-1990) and 2050 (mean 2046-2055), downscaled to 10 minutes using a cubic spline, which cover all land and marine areas. Exposure scores for the species with <80% overlap with the GCM ensemble projections were based on these rankings. Temperature and precipitation thresholds for all of the above exposure measures are shown in Table S1.

Low adaptive capacity

We estimated bird species' **intrinsic dispersal ability** using published or unpublished data on known mean and maximum dispersal (usually from studies involving ringing or marking nestlings and then recording the distance to where they first breed). Estimates were placed in logarithmic bands, and extrapolated from close relatives where no direct estimates were available. According to Malcolm *et al.* [32], required migration rates of ≥1 km per year were relatively common in all models if species were to remain within their bioclimatic envelopes, so we selected a threshold of 1 km per year, below which species were considered to have low adaptive capacity. To include species whose climate change driven migration might be **extrinsically limited by dispersal barriers,** we assigned low adaptive capacity scores to species with distribution ranges entirely within approximately 2000 m of a mountain-top or described as having "mountain-top" distribution, on small islands with maximum altitudes <500 m, and those with ranges within c.10° latitude from the polar edge of a land mass and within which ≥20% of current vegetation type is projected to disappear under doubled CO_2 levels.

Species with poor evolvability were identified in three ways. Information on **species' genetic diversity** is rare, but 69 species were reported in published studies to either have gone through a genetic bottleneck and/or have low genetic diversity. Slow turnover of generations was assessed based on species' generation lengths, estimated according to the IUCN Red List Guidelines [33]. We have no empirical reference point for a threshold of low adaptive capacity for this trait, so following our established methodology, we selected the ~25% of species with the longest generation lengths, resulting in a threshold of 6 years. Similarly, the 37.96% of species with the **lowest reproductive output** (mean annual clutch sizes ≤2) provided the closest threshold to 25% for data categories available.

Amphibians

The traits, biological information and thresholds used to assess amphibians' vulnerability are summarised in Table S2.

Sensitivity

Amphibians' habitat and microhabitat specialisation was assessed based on two traits. As for birds, **habitat specialisation** was assessed according to the number of IUCN Red List habitats listed for the species; species occurring in only one habitat were considered of high sensitivity, while those with 2-33 habitat types were considered of 'not high' sensitivity. Species' **microhabitat dependencies** were considered to confer high sensitivity if species are larval developers and dependent on freshwater microhabitats (based on the IUCN Red List (2008)). Forests are anticipated to buffer the climate change impacts on freshwater microhabitats, so species occurring in forests were excluded.

Narrow temperature and precipitation tolerances were measured in the same way as those for birds. Only one species had <80% of its range covered by the Worldclim dataset, so the HadCM3 1975 modelled climate was not used for amphibians.

Although amphibians are likely to be affected by a range of climate change driven **disruptions in environmental triggers**, insufficient data are available to systematically assess the group. Based on literature, expert knowledge and phylogenetic inference, we were, however, able to identify species dependent on the particularly vulnerable cue of rainfall or increased water availability for their mass (often termed 'explosive') breeding. This excludes species buffered by occurring in forests, and typically includes mud-aestivating grassland representatives of the frog genera *Hyperolius*, *Litoria* and *Leptodactylus*.

Amphibians' **interspecies interactions** with the pathogenic chytrid fungus *Batrachochytrium dendrobatidis* have been associated with population declines and extinction around the world [34–38]. One of the leading explanatory hypotheses proposes that the physiological stress caused by changing climates has a synergistic negative effect in combination with chytridiomycosis ([36,39,40], but see [41,42]), while climatic changes appear to facilitate the fungus' expansion into new areas [43]. We considered species to be subject to high sensitivity to increasing negative interactions with chytrid where (i) a chytridiomycosis-implicated decline or threat has already been recorded or is suspected (i.e. according to the IUCN Red List (2008), experts have listed threat by native or alien pathogens in the past, present and/or future); (ii) they are considered to be experiencing enigmatic decline [34]; or (iii) where future infection is probable and could potentially cause decline. The extent to which chytrid infection causes negative impacts on species appears to have a phylogenetic basis [44], so making the assumption that chytrid will be globally ubiquitous by 2050, we considered species to have high probability of future infection under (iii) where they are in a

genus with a recorded non-benign infection, are freshwater dependent and occur in subtropical or tropical forests, shrublands or grasslands.

Exposure

Amphibian exposure to sea level rise and temperature and precipitation changes was estimated in the same way as for birds. The coarse scale of the original GCM data resulted in poor coverage of the ranges of some small island and coastal species. 2.6% of amphibians had <80% of their ranges covered by the GCM ensemble projections for terrestrial areas (those on small oceanic islands and/or with narrow coastal ranges) and these, like birds, were assessed using rankings based on HadCM3 global projections. Temperature and precipitation thresholds for all of the above exposure measures are shown in Table S2.

Low adaptive capacity

We considered species to have **low intrinsic dispersal capacity** if they are not known to have become established outside their natural ranges, are not associated with flowing water, and have small ranges (≤ 4,000 km2). Since there is no empirical threshold for what constitutes a small range size, we identified the 25% of species with smallest ranges, in combination with the other characteristics of low intrinsic dispersal capacity described above. **Extrinsic dispersal barriers** were identified for species that occur exclusively on mountaintops, small islands, at polar edges of land masses and/or at polar edges of suitable natural habitat. Species were considered to have **low reproductive capacity** and hence poor evolvability where they are have low annual reproductive output (≤50 offspring (where known) or they are viviparous).

Corals

The traits, biological information and thresholds used to assess corals' vulnerability are summarised in Table S3.

Sensitivity

We assessed corals' habitat and microhabitat specialisation using two traits. We defined coral habitats as: barrier and patch reefs; atolls; fringing reefs; incipient, submerged and non-accreting reefs; and non-reefal rocky shores. The first three types each have subtypes: outer upper reef slope; outer lower reef slope; inter reef channel; spur and groove; outer reef crest; outer reef flat; inner reef flat; reef lagoon; back reef slope; and back reef crest. These describe a total of 32 habitats, the definitions of which accompany deposited data. The detailed nature

of these habitats meant that almost all species occur in multiple habitat types; since an empirical threshold for **high specificity to these habitats** is not available, we assessed the ~25% of species with the fewest habits (<13) as of high sensitivity. We consider depth range (maximum known depth minus minimum known depth) to be a component of **microhabitat specialisation** and, lacking an empirically-based threshold, we selected the ~25% of species' with greatest depth specificity (depth ranges ≤14m) to provide a relative estimate of this characteristic.

We used corals' reproductive strategy as a proxy for **larval temperature tolerance.** Because coral larvae must undergo dispersal via the water column, and broadcast spawners in particular require fertilisation and larval development near the sea surface, these corals are likely to be more at risk from climate change associated changes in sea surface temperatures and irradiance than those that are able reproduce by budding or fragmentation. We therefore scored species known to reproduce by means of only broadcast spawning and/or brooding as of high sensitivity to climate change. Secondly, we used evidence (published or observational) of past high temperature mortality of > 30% of local population on a reef or reef tract (typically inferred from smaller sample sizes) as a proxy for the magnitude of **adult coral colonies' temperature tolerances**. Lastly, because the impacts of increasing sea surface temperatures, irradiance and storms are known to attenuate depth, we considered species occurring exclusively above 20 m depth to have high sensitivity relative to those with ranges where such **impacts are buffered by depth**.

While some corals species have azooxanthellate colonies that are not dependent on dinoflagellate algae, that vast majority (>99%) of reef-building corals form obligate symbioses with Zooxanthellae algae [45]. The relationship between corals and their Zooxanthellae is a rapidly expanding field of research, and although massive advances have been made in recent years, the highly complex physiological relationship between the host coral and its endosymbiont Zooxanthellae, and the extreme challenges in Zooxanthallae taxonomy and identification, including inconsistency between researchers, leave large unknowns in our understanding of coral bleaching. Because certain clades of the Zoothanthellae genus *Symbiodinium*, including clades D, C1 and C15, are known to have relatively higher temperature tolerances [46,47] and be less vulnerable to bleaching, we used the most recent published and grey literature to record associations between each coral species and types of *Symbiodinium*, including clades A (2 types), B (20 types), C (59 types), D (6 types), F, and G (i.e. a total of 89 individual *Symbiodinium* types).

While relatively heat-tolerant Zooxanthellae types can confer an advantage to symbiont corals under high temperatures, their lower photosynthetic efficiency under typical, favourable temperature conditions confers a disadvantage due to resulting lower energy reserves [48,49] and slower growth [50]. Some coral colonies are known to experience changes in the relative and absolute abundance of different Zooxanthellae clades and/or types (that were already present within that coral colony) over time, a phenomenon often referred to as Zooxanthellae 'shuffling'. Typically one clade may be dominant and the others may be present at low to very low abundance [51]. Their presence at low abundance can facilitate shuffling [52], generally after a bleaching episode. We regard shuffling potential as likely to provide the flexibility needed for coral colonies to both survive high temperature events, and to retain a sufficiently rapid growth potential under favourable temperatures to compensate for ongoing erosion. We considered coral species to be capable of shuffling if a single colony sample has been found to harbour more than one Zooxanthellae clade or type simultaneously, a phenomenon reported for 55 species to date.

In conclusion, we regarded coral species as of high sensitivity to **disruption of Zooxanthellae symbioses** where these interactions are obligatory and where species are either not known to have temperature tolerant Zooxanthellae types D, C1 or C15, or where these clades are present but colonies are not known to 'shuffle' to more photosynthetically efficient types under favourable temperatures. Ongoing research is likely to add to the numbers of temperature tolerant types, the species known to harbour them, and reports of Zooxanthellae shuffling. We believe the logic applied to identifying corals highly sensitive to disruption of Zooxanthellae is justifiable and, based on the information available at the time of this assessment, this trait identifies 92.7% of corals as of high sensitivity to climate change.

We defined **rare species** as those occurring in geographically restricted areas (for example the Hawaiian Islands, Chagos Archipelago, Japan or parts of Arabia), as well as those that are typically sparsely distributed across their geographic ranges. The vast majority of sub-populations of virtually all reef-building coral species have not been adequately censused by researchers, and no overarching, detailed, quantitative data on actual abundances of meta-populations are available to assess global rarity. In the absence of such data, Veron [45] and our own published and unpublished datasets on local abundance estimates from multiple sites at more than 30 different locations including the Red Sea and other areas of Arabia, Madagascar, India, Thailand, E and W Australia, Vietnam, China, Indonesia, Philippines,

Micronesia, Papua New Guinea, Solomon Islands and Fiji [45,53–58] provided the basis for our qualitative assessments.

Exposure

To estimate which corals will be most exposed to climate change impacts, we calculated, firstly, their probability of experiencing bleaching, and secondly, the proportion of their ranges exposed to levels of ocean acidification beyond which no corals are currently known to exist. Mass bleaching events are commonly predicted based on the accumulation of sea surface temperatures in excess of a local climatological maximum. For example, mass bleaching is expected to be severe and lead to some coral mortality when the accumulation of 'degree heating months' (DHM) exceeds 2°C-month [59]. Because corals can recover from mass bleaching events if intervals between bleaching events are of sufficient durations, we use each species' mean frequency of severe bleaching (DHM>2°C) across its range as a metric of mortality-causing bleaching exposure.

Global spatial projections of maximum annual DHM for 2046-2055 were calculated by Donner *et al.* [59] using output from simulations of the Geophysical Fluid Dynamics Laboratory (GFDL) CM2.0 and CM2.1 climate models [60], based on the SRES A1B scenario. These models were chosen as they provide three key advantages for coral reef research over other GCMs[55]. Firstly, the ocean component of CM2.0 andCM2.1 operates on a higher resolution grid than most other IPCC Fourth Assessment Report (AR4) GCMs. The similarity between the models' resolution in the tropics and that of the available satellite-derived data eliminates the need for statistical downscaling of model output to the resolution of the satellite data. It also reduces the conflict between coastal geography in the GCM and the satellite map. The higher model resolution is particularly critical for research on tropical coastal ecosystems because a large proportion of more closed ocean basins like the Coral Triangle in Southeast Asia and the Caribbean are represented as land in lower resolution GCMs. Secondly, since the goal of this analysis was to estimate the response of biodiversity to climate change, models with climate sensitivities in the middle of the range of models used in the AR4 were regarded as most suitable. The difference between the climate sensitivities of CM2.0 and CM2.1 is caused by assumptions in the dynamic core of the atmospheric component of the otherwise similar models, but taken together the output of the two models represents a median estimate from the AR4. Lastly, accurately representing the frequency of thermal stress events on coral reefs requires climate projections from models that adequately describe the El Nino / Southern Oscillation. GFDL's CM2.0 and CM2.1 have among the best representations of the ENSO cycle of the models employed in the IPCC AR4[55].We down-

scaled the results to a 10 minute grid using a cubic spline and calculated the projected mean bleaching frequency in 2050 for each grid cell based on the number of years from 2046-2055 in each model simulation in which mean annual DHM exceeded 2°C.

Corals' projected exposure to ocean acidification was calculated based on projections of ocean aragonite saturation [61], low levels of which are known to reduce their growth rates, disrupt metabolic processes and, at particularly low rates, lead to dissolution [17]. While the aragonite saturation states (Ω aragonite) of 3.5 [61] and 3.25 [62] have been proposed as thresholds below which almost no reefs currently occur, for this study we used an optimistic threshold of 3, levels below which are described as "extremely marginal" by Guinotte *et al.* [63].

To represent the SRES A1B scenario for 2050, we used spatially explicit projections of aragonite saturation levels, corresponding to atmospheric CO_2 concentrations of 550ppm, created by Cao and Caldeira [61]. We down-scaled these from 2.5 x 3.75 degrees to 10 minutes using a cubic spline. For both bleaching frequency and aragonite saturation projections, the downscaling of data from coarse scales to 10 minutes resulted in a poor coastline definition and non-overlapping of surfaces with some reefs, particularly in small or narrow marine areas such as the Red Sea and Persian Gulf. Where ≤50% of a species' range was not covered by the surfaces (9.0% of species for bleaching frequency and 8.6% for aragonite saturation), the species was assessed as unknown for the respective trait. Thresholds for the above exposure measures are shown in Table S3.

Low adaptive capacity

We used larval competency, specifically the maximum time known for successful larval settlement, as a proxy for species' **intrinsic dispersal capacities**. Some coral species' larvae can survive up to several months in the water column, potentially being transported enormous distances if no appropriate habitat for settlement is available and environmental conditions in the plankton are suitable. We categorised maximum settlement time into five categories (<7 days, 7-14 days, 14-30 days, >30 days, and unknown); lacking an empirical basis for a threshold, we selected a cut-off of <14 days to settlement which identified the worst 14.0%, the closest possible to the 'worst 25%' used elsewhere in this study. For species whose larval competency times to settlement are unknown, the 'typical' values for their particular sexual reproductive modes were used to infer dispersal distances, and for species that are both brooders and spawners, and for which specific larval competency data were unavailable, the typical competency period of the brooding mode were used. For species whose reproductive

mode(s) are also unknown, the major mode of either their congeners or confamilials was assumed where appropriate.

While ocean currents can provide an excellent vector of dispersal, together with ocean temperature, they can also be **barriers to dispersal** (e.g., [64,65]). For example, the combination of currents and cold waters of the southern and northern Pacific and Atlantic Oceans and southern Indian Ocean preclude natural inter-ocean dispersal for virtually all reef-building corals (with the possible exception of *Madracis pharensis)*. We identified barriers typically for species with restricted distributions at the end of uni-directional currents (e.g., Kurishio, Leeuwin and East Australian currents), or which appear isolated by areas of unsuitable oceanographic conditions (e.g., the cool upwelling in the Arabian Seas). Patterns of species' distribution and endemism in the Western Indian Ocean and Arabian Seas are consistent with a barrier to dispersal of some species eastward across the Indian Ocean and thence into the Pacific, likely related to current flows, including the Indonesian Through-Flow which transports water from the Pacific into the Indian Ocean. This trait is particularly difficult to quantify, given the high geographic and temporal variability in ocean current flow patterns and because different coral populations within a species' meta-population experience different oceanographic features and hence have differing dispersal potentials in various locations. We identified potential dispersal barriers based on known existing oceanographic characteristics, and while climate change is likely to affect inter-seasonal and inter-annual variability in onset and velocity of currents, as well as in ocean temperatures, we have not attempted to incorporate this in our assessments.

Corals' evolvability was estimated, firstly, based on each species' **generational turnover**, as estimated by typical colony longevity. Age of colonies can be difficult to determine accurately, although there are relatively consistent relations between growth rate, colony size and age in some species, and coring of some large massive corals has provided independent minimum age estimates. For others, fragmentation, injury, disease and other factors can confound such relations. Furthermore, fragmentation and budding produce clones that can ensure that the same genotypes persist on reefs for millennia. Such genotypic ages are not considered here, and we focus on the age of 'individual' colonies. Because many species are widespread and researchers have examined only a very small fraction of the populations in detail, it is not possible to assign a maximum age definitively to colonies of any species. As a result, we used broad categories of colony longevity, namely <10 years, 11-50 years, 50-100 years, >100 years, and unknown, and selected species with colonies typically living more than 50 years (1.6% of species) as those with low adaptive capacity according to this trait.

Corals with **slow growth rates** tend to have lower reproductive capacity on average, because colony size and reproductive output are related. Conversely, faster growing species attain larger size and, in some species at least, reach reproductive maturity sooner than their slower growing counterparts, and hence contribute more rapidly to the gene pool [66]. Field studies over the past century have established coral **colonies' growth rates** for species representing most of the main growth form categories. These vary with environment and phenotype, so we have assigned species to broad growth rate categories (<10mm yr^{-1}, 11-30 mm yr^{-1}, 31-100mm yr^{-1}, >100mm yr^{-1}, unknown) making inferences based on growth form and phylogeny where specific growth rate data are lacking. Once again, without an empirically-based threshold for this trait, we identify the ~25% of species in the slowest growth categories.

Plotting areas of greatest concentrations of vulnerable species

Here we use bivariate plots to highlight the relationship between vulnerability dimensions based on biological traits (i.e. sensitivity and low adaptive capacity) and exposure, since this largely a function of how much climatic change is projected in the geographical area in which a species occurs (Fig. 2). Bivariate plots were produced by dividing per cell frequencies of (i) species that are both sensitive and of low adaptive capacity and (ii) exposed species into 10 classes based on Jenks natural breaks. These classes were used as coordinates on a 10 x 10 grid, with the biological trait-derived dimensions on the y-axis and exposure on the x-axis. Each grid cell was assigned a colour which graduated from muted colours for low frequencies to highly saturated colours representing extreme values (blue for sensitivity and low adaptive capacity (i), yellow for climatic exposure (ii) and purple for areas with high numbers of both groups; see legend for Fig. 2). Each grid cell of the global map was assigned a colour value according to the projected frequency of species in these groups, thereby illustrating spatial covariation between the two variables of interest [67]. Areas of greatest concentrations of species in groups (i) and (ii), as well as of their overlap, and are described in Tables S8-9.

Analyses

Comparisons between species' climate change vulnerability and their IUCN Red List threat statuses

We used the IUCN Red List (2008) as a basis for establishing each species' level of extinction risk. The IUCN Red List Categories and Criteria are the most widely accepted system for classifying species' extinction risks [68–70]. The IUCN Red List includes seven categories of threat, namely Extinct (EX), Extinct in the Wild (EW), Critically Endangered (CR), Endangered (EN), Vulnerable (VU), Near Threatened (NT), Least Concern (LC), as well as the category Data Deficient (DD). A species is regarded as threatened if it falls into categories CR, EN or VU. It may qualify under these categories by meeting thresholds in any one of five criteria (A-E). The criteria are designed to be objective, quantitative, repeatable, and to deal with uncertainty [33]. Assessments are based on an evaluation of information on all known individuals of a species (i.e. at a global scale), integrating the information from all populations, subpopulations and subspecies. As a result, the extinction risk category reflects the overall status of the species, which may, for example, be of Least Concern despite some populations/subspecies being at risk [21]. In particular cases, separate assessments of subspecies and/or populations are carried out, but these are not included in the analyses presented in this paper.

Although climate change is frequently listed as a threat during red listing, no species of birds, amphibians or corals were listed as threatened solely or principally due to climate change. As a result, we included all species in our comparison between threatened and vulnerable species. For each of birds, amphibians and corals, we used a chi-square test to compare the numbers of species that were threatened and vulnerable, threatened and not vulnerable, vulnerable and not threatened, and neither threatened nor vulnerable (see Table S10). To show areas containing greatest concentrations of 'threatened', 'vulnerable' and 'threatened and vulnerable' species, we used bivariate plots (as described above in Supporting Methods section 'Plotting areas of greatest concentrations of vulnerable species'), which are shown in Fig. 3 and Fig. S6 and described in Tables S11-12.

Calculating numbers of vulnerable species under different emissions scenarios and time frames

To investigate the roles that differing concentrations of atmospheric greenhouse gas emissions could have on species' vulnerability, we compared the numbers of vulnerable species presented in previous analyses in this paper (i.e. based on the 'midrange' A1B

scenario for 2050; terrestrial species ranges) with those calculated for A1B for 2090, B1 (low emissions) for 2050 and 2090, and A2 (high emissions) for 2050 and 2090. These three standard scenarios represent a range of the possible future scenarios explored by the IPCC [71,72].

We applied the threshold values identified for each exposure variable using the baseline scenario (A1B for 2050; threshold values shown in Tables S1-3) to the same four variables under the alternative scenarios and timeframes discussed above, and recalculated exposure and vulnerability scores accordingly. For example, under the baseline scenario, amphibians were regarded as highly exposed to changes in mean temperatures where the absolute changes in mean temperatures between 1975 and 2050 are ≥ 2.96 ^{o}C. We used the same threshold of 2.96 ^{o}C to classify species as exposed under the A1B 2090 scenario, and since projected temperature changes are generally greater, more species qualified as exposed under this trait. Recalculating overall exposure and then vulnerability based on these results yielded larger numbers of vulnerable species overall.

As expected, the high (A2) and low (B1) scenarios for 2090 yielded higher and lower numbers of vulnerable species than the midrange (A1B) for 2090, except for corals under a pessimistic scenario, where A2 and A1B produced the same number of vulnerable species. We note that A2 produced fewer vulnerable species than A1B at 2050, reflecting correspondingly higher mean global temperatures and precipitation for A1B relative to A2 for 2050, as projected by the four GCMs selected for this study. The numbers of vulnerable species under each scenario in 2050 and 2090 are shown in Fig. 4 and maps of their greatest concentration are shown in Figs S7-9. Given assessment methods, the absolute values of estimates are uninformative, but changes from 2050 to 2090, the differences between scenarios and the differing geographical distributions of vulnerable species are valuable.

Assessing the influence of each trait on overall vulnerability

To explore the relative contribution of each trait to overall vulnerability, we calculated the number of species and the size of geographic priority area uniquely identified by each biological trait, for each taxonomic group. We present these results in Tables S13-15 and rank traits according to their relative contributions to both numbers of vulnerable species and size of geographic areas containing vulnerable species. We find, firstly, that traits contributing most to numbers of vulnerable species are, in many cases, not the same as those contributing most to the geographic priorities identified. Secondly, we find that highest

ranking traits are generally not consistent across taxonomic groups. Each trait accounts for uniquely identifying an average of 7.8 ±7.4% of vulnerable bird species, 12.6 ±9.7% of vulnerable amphibian species and 13.4 ±16.6% of vulnerable coral species, while in terms of uniquely identified vulnerable geographic areas (i.e., those containing one or more vulnerable species), these figures are 16.7 ±6.0% for birds, 14.8 ±21.0% for amphibians and 2.5 ±5.6% for corals.

'Limited intrinsic dispersal capacity' is the only trait that falls within the top five ranking for uniquely identifying vulnerable species for all three taxonomic groups, although birds and amphibians additionally share 'slow turnover of generations' as a top ranking trait. 'Limited dispersal capacity' and 'low reproductive output' identify the greatest numbers of vulnerable birds, while for amphibians highest ranking traits are 'slow turnover of generations' and 'limited dispersal capacity'.

There is no overlap between birds and amphibians in the highest ranking traits for the identification of unique geographic areas. For birds, 'narrow temperature tolerances' ranks highly, as do 'low reproductive output' and 'geographical barriers to dispersal'. For amphibians, 'changes in mean temperature', 'narrow precipitation ranges' and 'slow turnover' play the greatest roles in uniquely identifying regions of high vulnerability.

For corals, sensitivity traits show a particularly high level of redundancy, partly due to the high percentage of species qualifying due to 'declining positive interactions with Zooxanthellae'. Exposure measures, however, show very little redundancy and approximately two thirds of species are uniquely identified by either 'high bleaching frequency' or 'high acidification'. This result reflects the largely complementary geographic areas affected by the two factors in the time frame considered (i.e., by 2050), and highlights the particularly challenging nature of the threats corals face.

These analyses provide useful information to inform prioritisation of ongoing trait data collection for birds, amphibians and corals. For example, 11% of birds qualified as vulnerable due to relatively small population sizes, but no species were uniquely identified by this trait, suggesting that it is not a priority for further data collection. We note, however, that because (like several other traits) it contains a number of unknown values, and even a few species or regions identified could be of particular significance, we do not suggest dropping any traits altogether at this stage.

Assessing the influence of trait thresholds and other sources of uncertainty on overall vulnerability

We distinguished four types of traits, each of which required distinct threshold selection approaches. Firstly, where species' tolerance thresholds are clearly established and widely accepted in the peer-reviewed scientific literature (e.g., ocean temperature conditions at which coral bleaching occurs), we referenced and used these. The second trait threshold type applies to data that are binary and where independent, widely accepted categorisations are available (e.g., occurrence only on islands; occurrence in only one habitat type). We regard these thresholds as objective and do not consider them to be a significant source of uncertainty in vulnerability assessments.

The third trait threshold type, used when no binary or established thresholds were known and where trait data were continuous or categorical, involved selecting the worst affected 25% of species (e.g., temperature and precipitation change tolerances), or the species in categories with a break closest to 25% (e.g., generation length for birds; depth ranges for corals). The fourth threshold type was used for traits where sufficient information and/or experience were available for experts to believe that they could defensibly set thresholds for heightened vulnerability (e.g. exposure to sea level rise based on habitat affiliations; inherent rate of dispersal required for birds based on projections in the literature [73]). Because these thresholds could introduce subjectivity into assessments, we explored sensitivity of vulnerability scores to shifting them to higher and lower values. In Tables S16-18 we identify the traits for which these 'percentage thresholds' (marked as **(P),** blue text) and 'expert thresholds' (marked as **(E),** green text) were used and examined their influence on overall vulnerability scores (see Tables S19-21).

We found that shifting percentage thresholds by 10% (i.e., to a more lenient 35% and a stricter 15%) changes the numbers of vulnerable species by only +11% to -12% for birds and +9% to -9% for amphibians, suggesting that these groups are relatively robust to the percentage thresholds selected. For corals, however, changes of +29% and -8% suggest that threshold choices play a larger role. Shifting expert threshold had an even lower impact on numbers of vulnerable species, shifting them by +1% to -1% for birds, +7% to -9% for amphibians and +3% and -0% for corals.

The geographic regions highlighted as having high concentrations of vulnerable species for birds, amphibians and corals respectively under different percentage threshold scenarios are shown in Figures S10-13. For birds, the location of priority regions differ little under the

different threshold scenarios, with the exception of disappearance of sub-Saharan Africa and particularly the Congo basin as a priority when strict percentage thresholds were applied (Figure S10). Similarly, priorities for amphibians are largely consistent across all scenarios, though the central Amazon basin is excluded under a strict percentage threshold, Madagascar is excluded under a strict expert scenario and south-eastern North America and west tropical Africa begin to appear as priorities under a lenient percentage threshold (Figure S11). For corals, the scenario results are particularly consistent, though the Caribbean declines slightly in prominence as a priority under a lenient percentage scenario (Figure S12).

Comparisons of potential sources of uncertainty (Tables S19-21) show that for birds and amphibians, missing/unknown data plays by far the greatest role, with the choice of percentage thresholds and the time frames for the assessment as second most important source for both groups. For corals, percentage thresholds play a marked role, with the time frame selected for the assessment and greenhouse gas emission scenarios the second and third most important sources of uncertainty respectively. For all three groups, uncertainty introduced by expert thresholds plays only a relatively small role.

In conclusion, the broad range of sensitivity analyses conducted shows that missing data, choice of traits and their thresholds and expert judgement all introduce a degree of uncertainty into vulnerability assessments. We find, however, that the geographic priorities identified by our approach are notably robust to this uncertainty, strengthening confidence in the main results of this paper. By presenting results as ranges of possible numbers of vulnerable species under different scenarios, emphasising repeatedly that scores are relative measures, and conducting sensitivity analyses on all main possible sources of uncertainty, we believe that we have dealt responsibly with the uncertainty inherent in assessments of future impacts of climate change on complex biological systems, and that the results presented provide the best assessments possible given available data and knowledge.

Supporting Discussion

Opportunities for validation of the framework

Vulnerability assessments, including the one we present, should be empirically evaluated to determine whether they produce robust ecological or conservation assessments of the impacts of climate change. At this stage, however, this remains challenging. A body of *ad hoc* observations of climate change impacts on species is emerging (as summarised in [73–75]) but the use of these studies for testing global assessments such as ours has several serious limitations. They cover only a small fraction of our study's species and generally address a limited range of possible climate change impacts (typically distribution range shifts and phenological changes), ignoring a broad range of other possible impacts that our approach considers. Such studies have strong geographic and ecosystem biases (typically towards Northern Hemisphere temperate regions); species in other regions and ecosystems may not respond in the same way. They tend to demonstrate population changes rather than the species-scale responses we project, and are based on non-standardised surveying methods. Lastly, existing observation studies represent a non-random subset of climate change responses and due to publication bias, are likely to under-represent species of lower vulnerability to climate change (e.g. those of high latent risk, and many potential persisters and potential adapters (as identified in Fig. 1)).

Another possibility for validation, particularly of trait selection, is to examine species' past responses to climatic changes, as evidenced in the paleorecord. We plan to explore this avenue of research, but are aware that it suffers from many of the limitations described above for *ad hoc* observational studies. Cross-referencing results of our assessment with others based on, for example, species distribution models [76,77], dynamic global vegetation models [32] and novel and disappearing climates [78,79] provides a further avenue for investigation, but since such models are simply alternative predictions with their own limitations and assumptions, also often unvalidated, results will need to be interpreted with caution. We note that outputs from other approaches could be incorporated into our assessment framework. For example, global vegetation models could be used to assess a species' exposure to habitat changes, and projections of species' range shifts could inform assessments of the likelihood of a species' successful dispersal in response to climate change.

Finally, we propose that the most effective and reliable means of gathering observational data for validating this and other approaches is through standardised monitoring schemes that adequately sample environmental gradients [5]. Several such schemes are being established (e.g., [80–82]) and we believe that, with further expansion, this approach can deliver the information needed to effectively validate climate change vulnerability assessments. We emphasize the need for immediate and ongoing support and expansion of standardised monitoring schemes globally.

Caveats and uncertainty

Since the results of this assessment are, at this stage, largely unvalidated, we note some important caveats to our methods. These are necessary to consider when interpreting the results, but also form priority areas for new research.

1. We acknowledge that experts' judgements can be subject to certain biases [83], but emphasise their value, particularly where timely decisions are needed in the face of novel, future or uncertain situations [84], for example for IPCC assessment reports and the IUCN Red List.
2. The selected trait threshold we chose (25%) is arbitrary and is unlikely to represent any real limit to species' tolerances. It simply highlights the top scoring species as a basis for analysis. Sensitivity of results to this threshold is explored in section '*Assessing the influence of trait thresholds on overall vulnerability'* above, but ideally the threshold would be updated or validated through observations and or experiments of the way in which climate change and traits interact (e.g., [85,86]). When interpreting the absolute values of the percentages for each group, it is important to recognise that these simply represent the degree of overlap between sensitivity, low adaptive capacity and exposure within the taxonomic group (e.g., highest overlap in birds (24-50% of species highly vulnerable) vs. lower overlap for corals (15-32% highly vulnerable)). It is particularly important to emphasise that comparisons between the percentages of high vulnerability species cannot be interpreted to represent any real differences in vulnerability between taxonomic groups.
3. Our framework's scoring system is based on the assumption that species have multiple pathways to extinction; traits were selected and scores calibrated such that a 'high' score on any single e.g. sensitivity trait would result in the species being ranked as 'sensitive' overall. As anthropogenic climate change progresses, the range, species-

specificity and frequencies of extinction pathways (no doubt including some not yet identified) will become apparent, but at this point, we believe it is premature to rank one trait as more important than another or exclude any that have been identified as possibilities. We acknowledge that this simple, equally-weighted combination of traits and trait groups fails to account for their potentially differing importance in conferring climate change vulnerability, but we are unable to quantify or justify relative trait weightings.

4. In practice, the biological traits are likely to interact with each other and with environmental change in non-linear ways, and there will be thresholds and abrupt state changes as a result. These effects are likely to be very specific and context-dependent and the only way to develop an understanding will be through detailed field studies over many years with a great deal of relevant climate and environmental information. This is simply going to be impossible for many species, but the availability of a few such studies [11,85,87] and the deployment of more mechanistic models (e.g., [88,89]) should start to support more sophisticated approaches than the very broad brush approach we use here.

5. Our approach does not specify the relationship between vulnerability scores and the risk of extinction. Although our analysis shows vulnerability to be correlated with extinction risk (as determined by the IUCN Red List™ Criteria) within a taxonomic group, it is not possible to equate vulnerability with a specific level of threat, and the relationship between vulnerability and extinction risk may be different for different groups. Results may be interpreted, for example, to predict which bird species and geographic regions will be at relatively higher risk of climate driven extinction than others, but not to quantify this risk, nor to compare birds' vulnerability with that of amphibians or corals. Our exposure modelling suggests that corals, in particular, are likely to face a much higher risk of extinction than the other taxonomic groups, though this is not reflected in the results of this study.

6. We recognize that climate will have positive effects on many species. In fact many species are already benefitting from climate change especially in temperate areas [5], and to date most range shifts recorded have resulted in range expansions more than range contraction [90]. However, our framework does not attempt to incorporate this – we are interested in identifying species at risk from climate change.

Supporting Figures

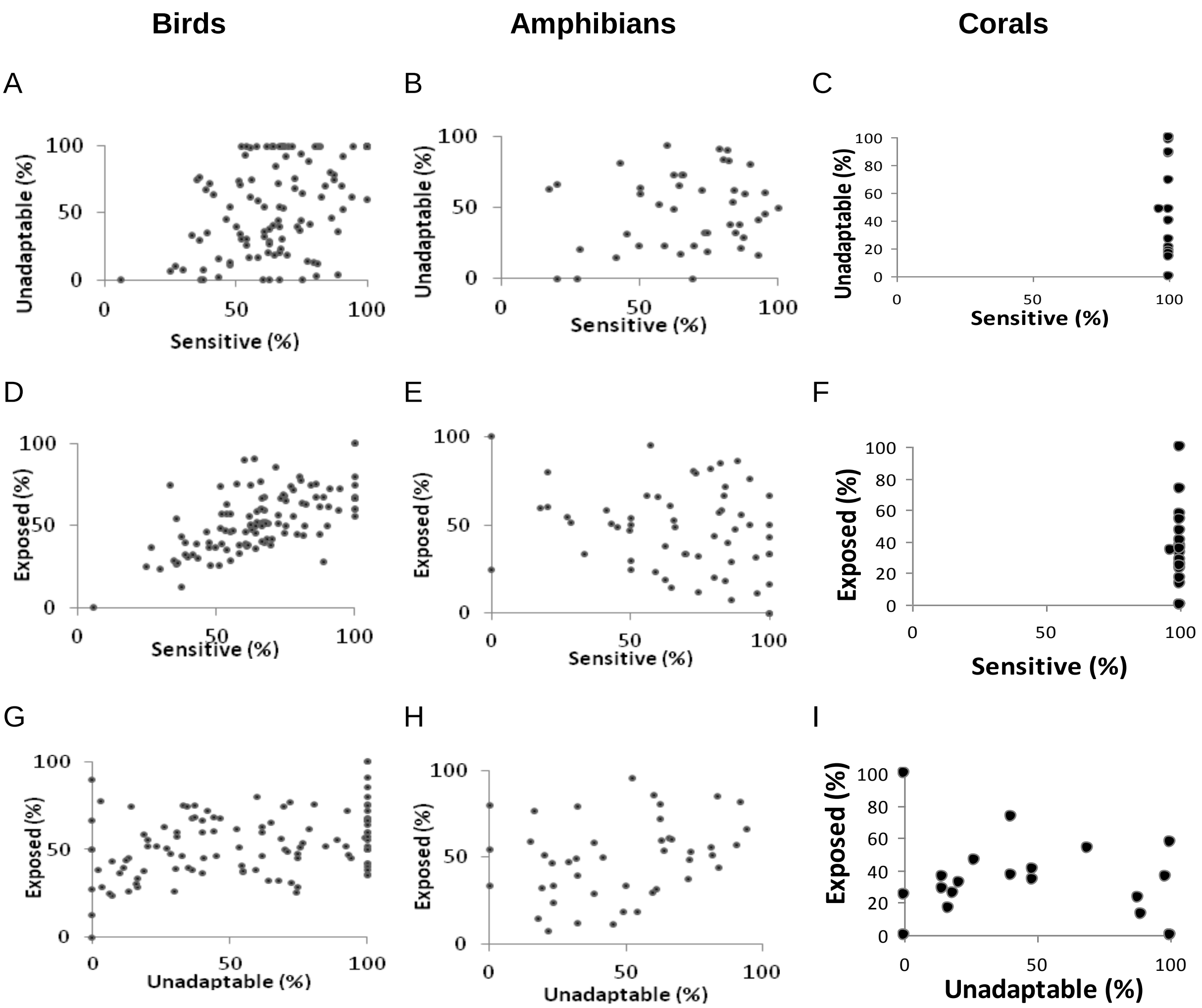

Figure S1: The relationship between climate change vulnerability dimensions for families containing ten or more species (based on an optimistic scenario for unknown trait values). Graphs show the percentages of each family's species that are highly sensitive vs. of low adaptive capacity (A-C), sensitive vs. exposed (D-F), and of low adaptive capacity vs. exposed (H-J) for birds, amphibians and corals respectively.

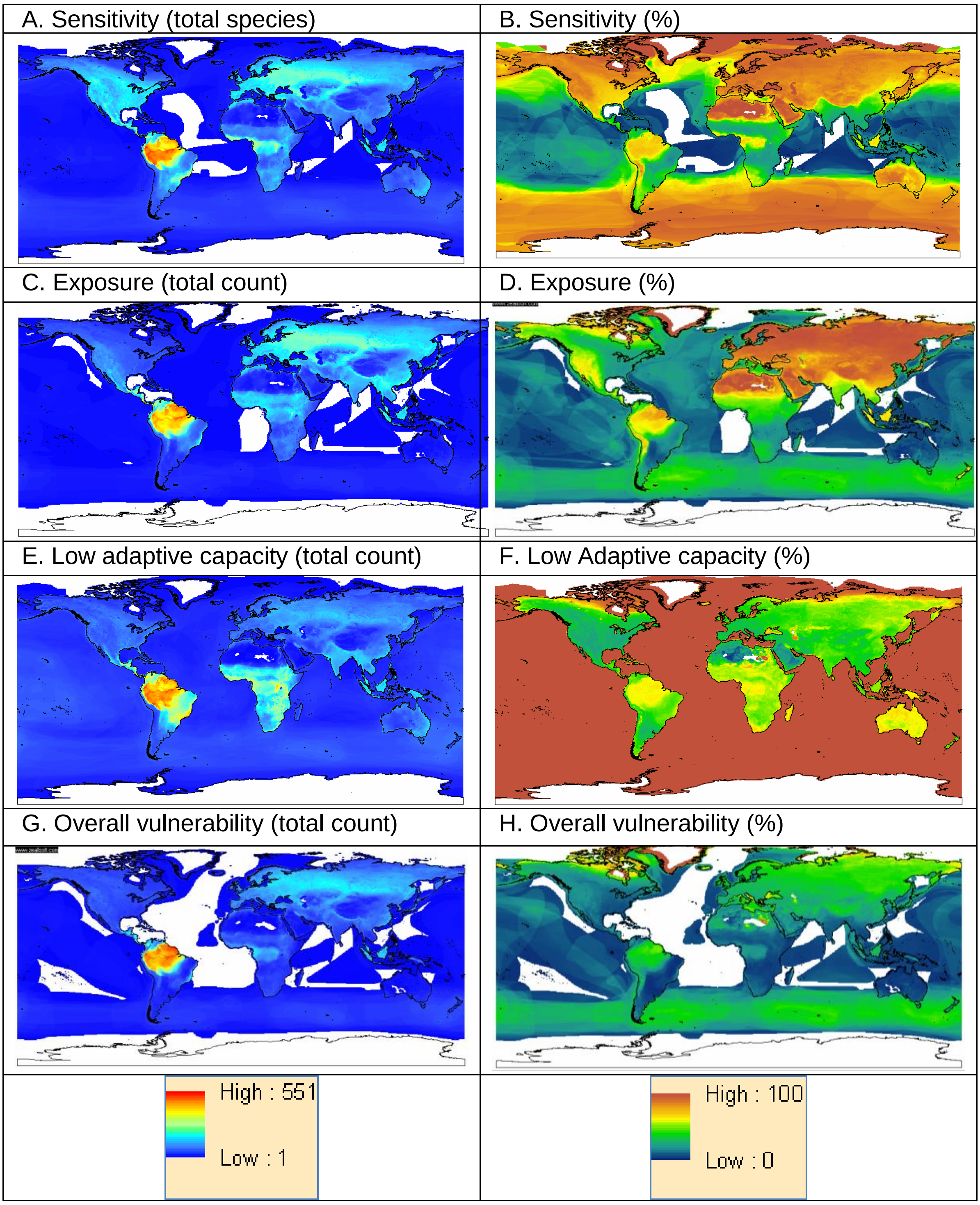

Figure S2: Geographic concentrations of bird species that are highly sensitive (A-B), exposed (C-D), have low adaptive capacity (E-F) and are highly climate change vulnerable overall (G-H), based on an optimistic scenario for unknown trait values. Parts A, C, E and G represent total numbers of species, while B, D, F and H show the proportions of total species in the groups i.e., relative to total species richness.

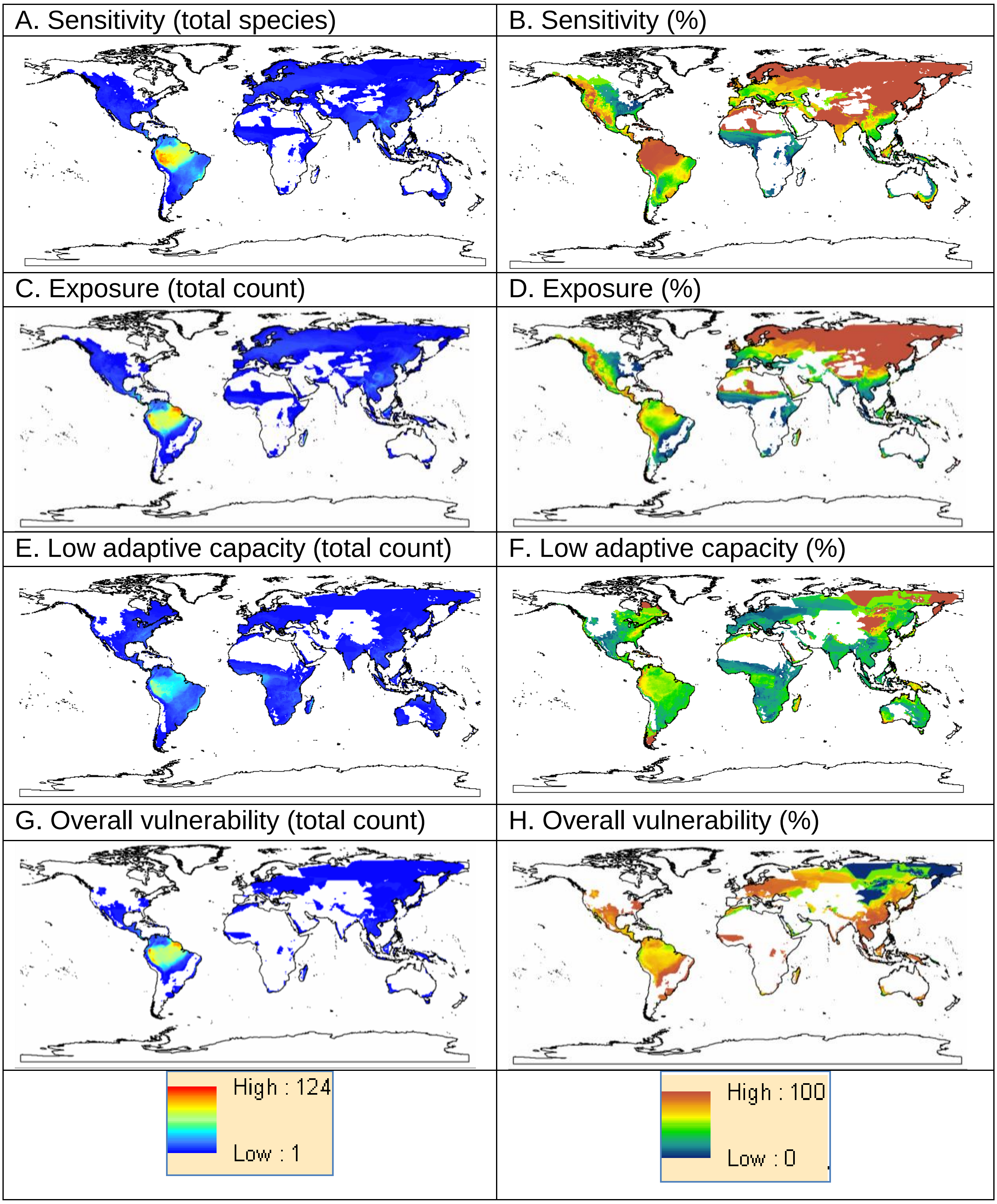

Figure S3: Geographic concentrations of amphibian species that are highly sensitive (A-B), exposed (C-D), have low adaptive capacity (E-F) and are highly climate change vulnerable overall (G-H), based on an optimistic scenario for unknown trait values. Parts A, C, E and G represent total numbers of species, while B, D, F and H show the proportions of total species in the groups i.e., relative to total species richness.

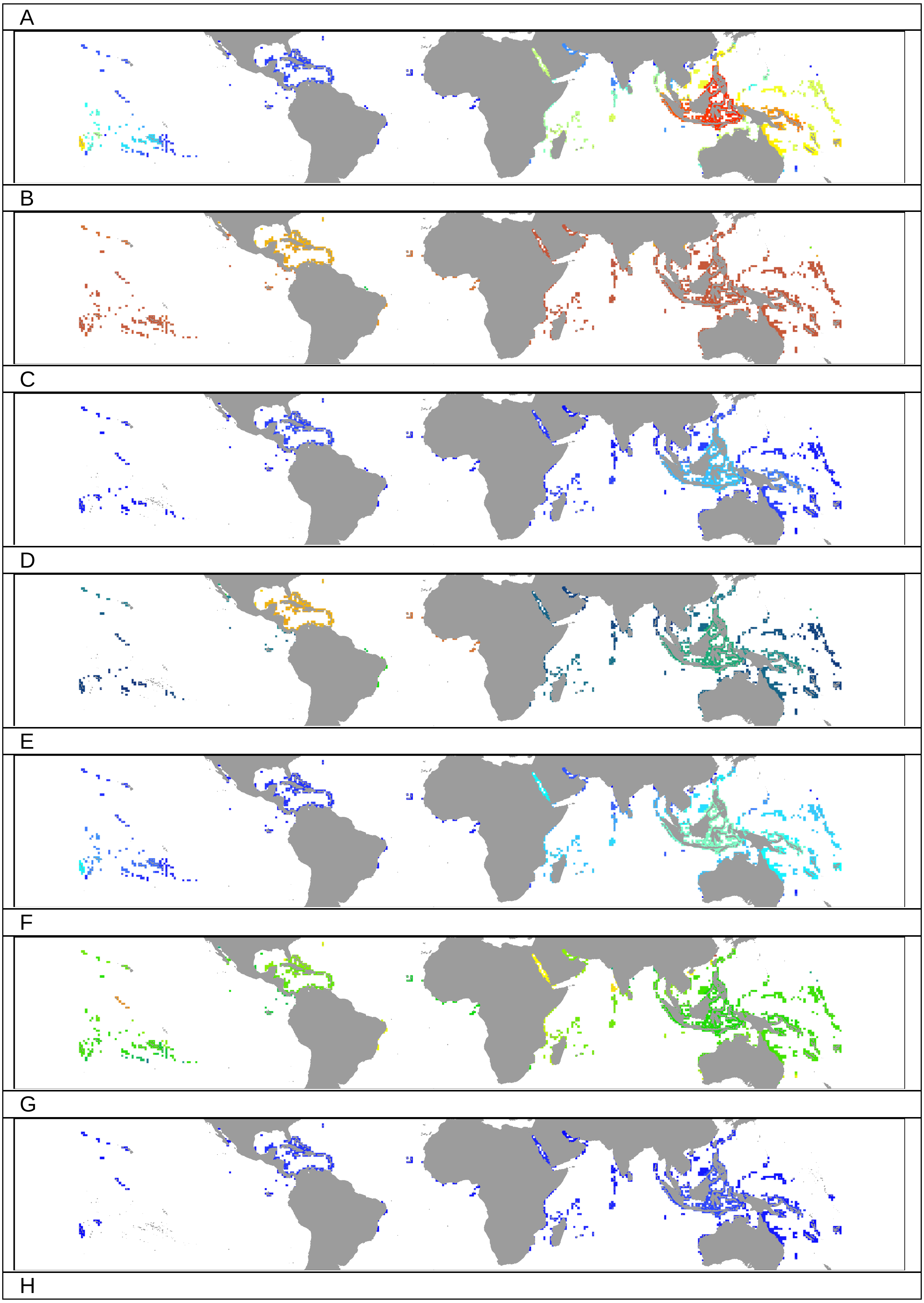
A
B
C
D
E
F
G
H

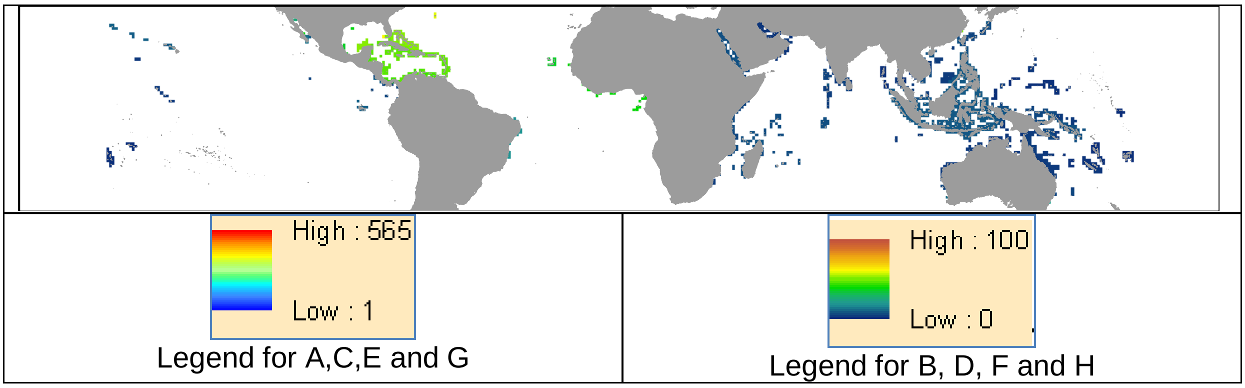

Figure S4: Geographic concentrations of coral species that are highly sensitive (A-B), exposed (C-D), have low adaptive capacity (E-F) and are highly climate change vulnerable overall (G-H), based on an optimistic scenario for unknown trait values. Parts A, C, E and G represent total numbers of species, while B, D, F and H show the proportions of total species in the groups i.e., relative to total species richness.

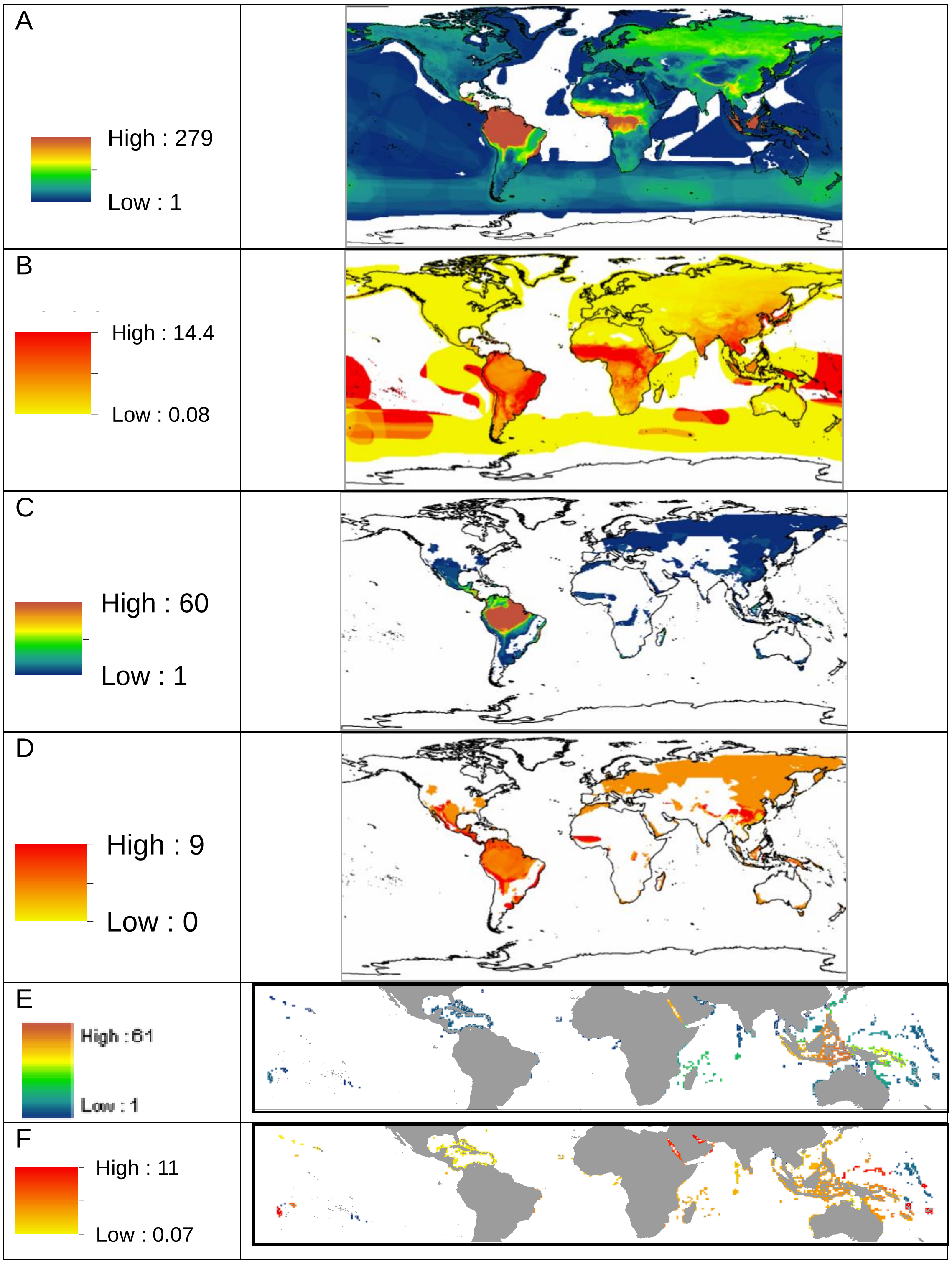

Figure S5: Geographic concentrations of species that are highly vulnerable under a pessimistic scenario (i.e., when unknown trait scores are assumed to be high climate change vulnerability scores) **but not under an optimistic scenario** (i.e., when unknown trait scores are assumed to be low climate change vulnerability scores), **for birds, amphibians and corals** (A, C, and E respectively). B, D, and F show the numbers of the above species relative to the number of species already known to be climate change vulnerable there (e.g., a score of six shows that there could be up to six times more highly climate change vulnerable species if unknown trait values represent high vs. low values).

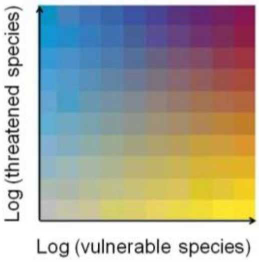

A

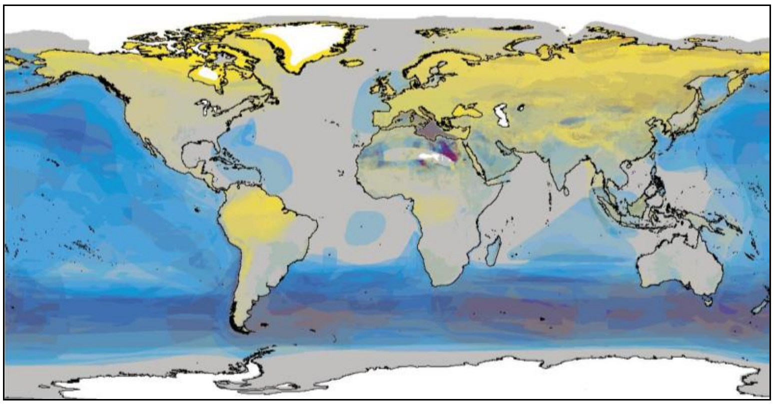

B

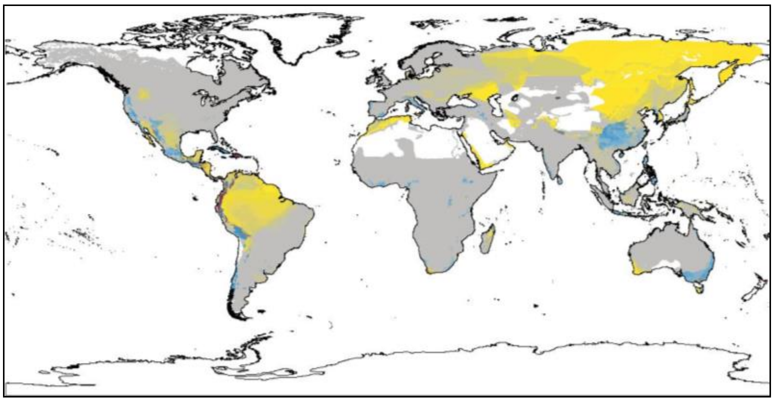

C

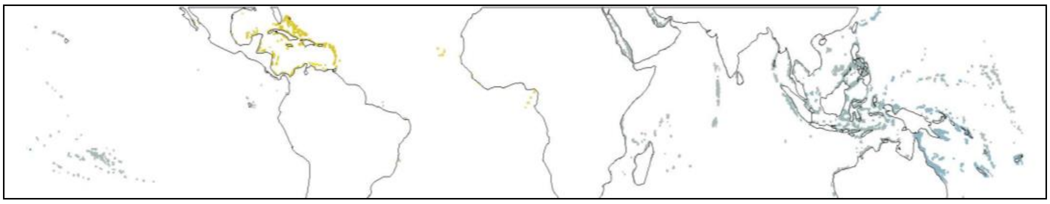

Figure S6: Bivariate plots showing areas with highest logged proportions (relative to species richness) **of species that are climate change vulnerable only in yellow, threatened only in blue, and both highly climate change vulnerable and threatened in maroon.** Logged total numbers of birds, amphibians and corals are represented by A, B and C respectively (see Fig. 3 for maps of the total numbers of species). Grey areas show where species are present, but few are climate change vulnerable or threatened; colours increase in intensity as species concentrations increase. Plots assume optimistic assumptions for missing trait information.

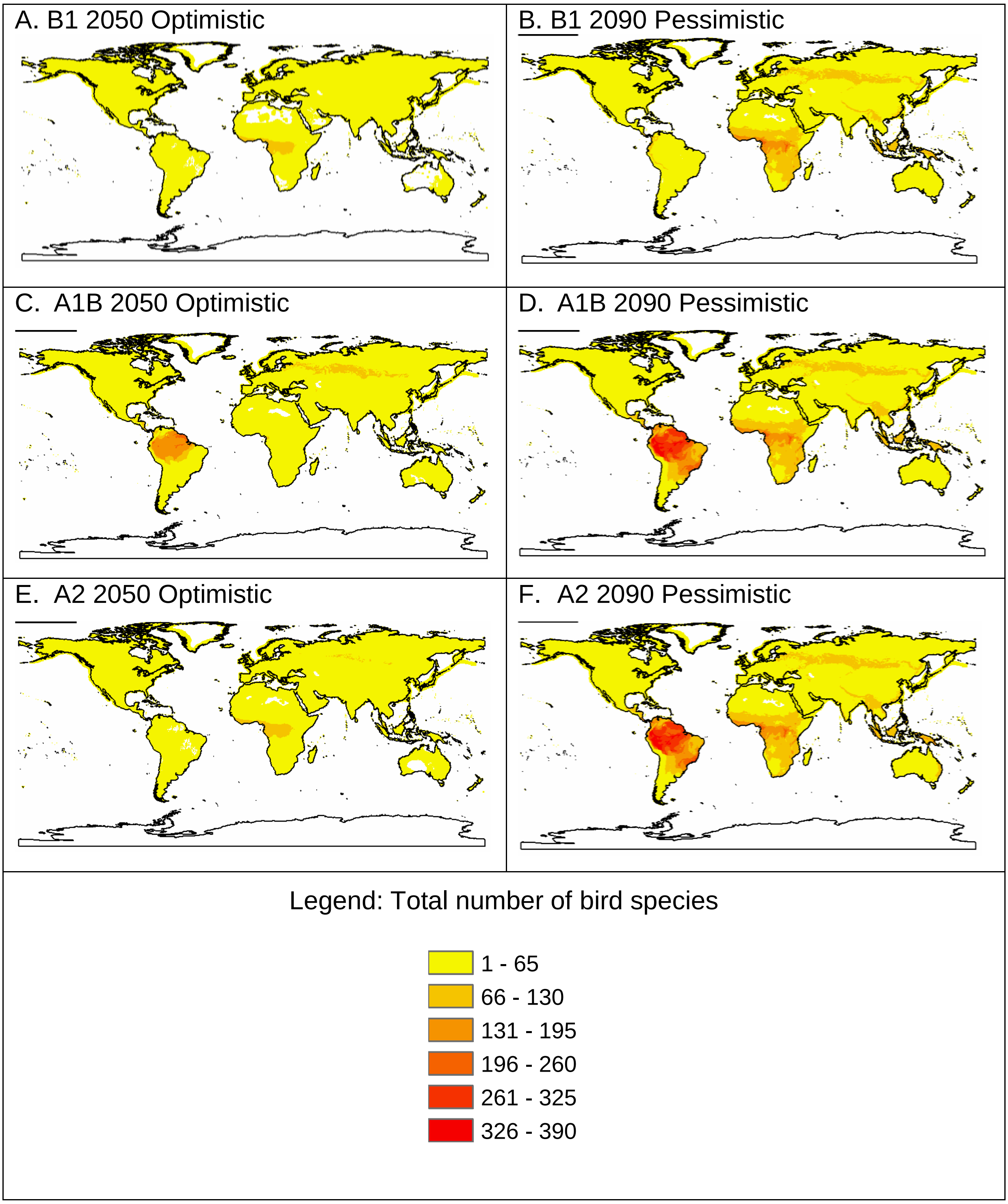

Figure S7: Foci of highly climate change vulnerable birds under three IPCC SRES climate change scenarios for 2050 and 2090. Low range scenario B1, moderate A1B (used as the baseline for all other assessments in this study) and high range A2 are represented by A, C and E respectively for 2050, while B, D and F show the same scenarios for 2090.

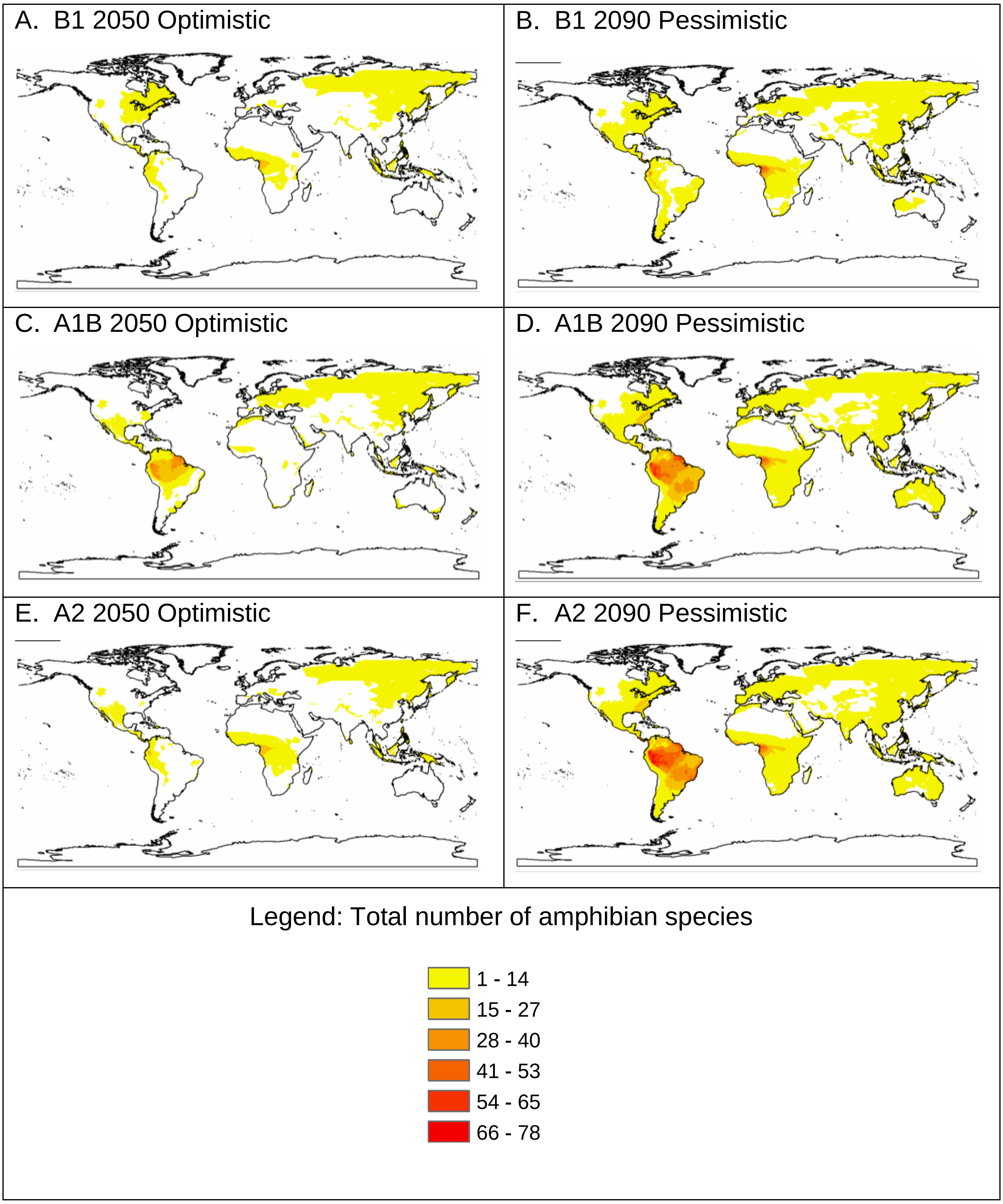

Figure S8: Foci of highly climate change vulnerable amphibians under three IPCC SRES climate change scenarios for 2050 and 2090. Low range scenario B1, moderate A1B (used as the baseline for all other assessments in this study) and high range A2 are represented by A, C and E respectively for 2050, while B, D and F show the same scenarios for 2090.

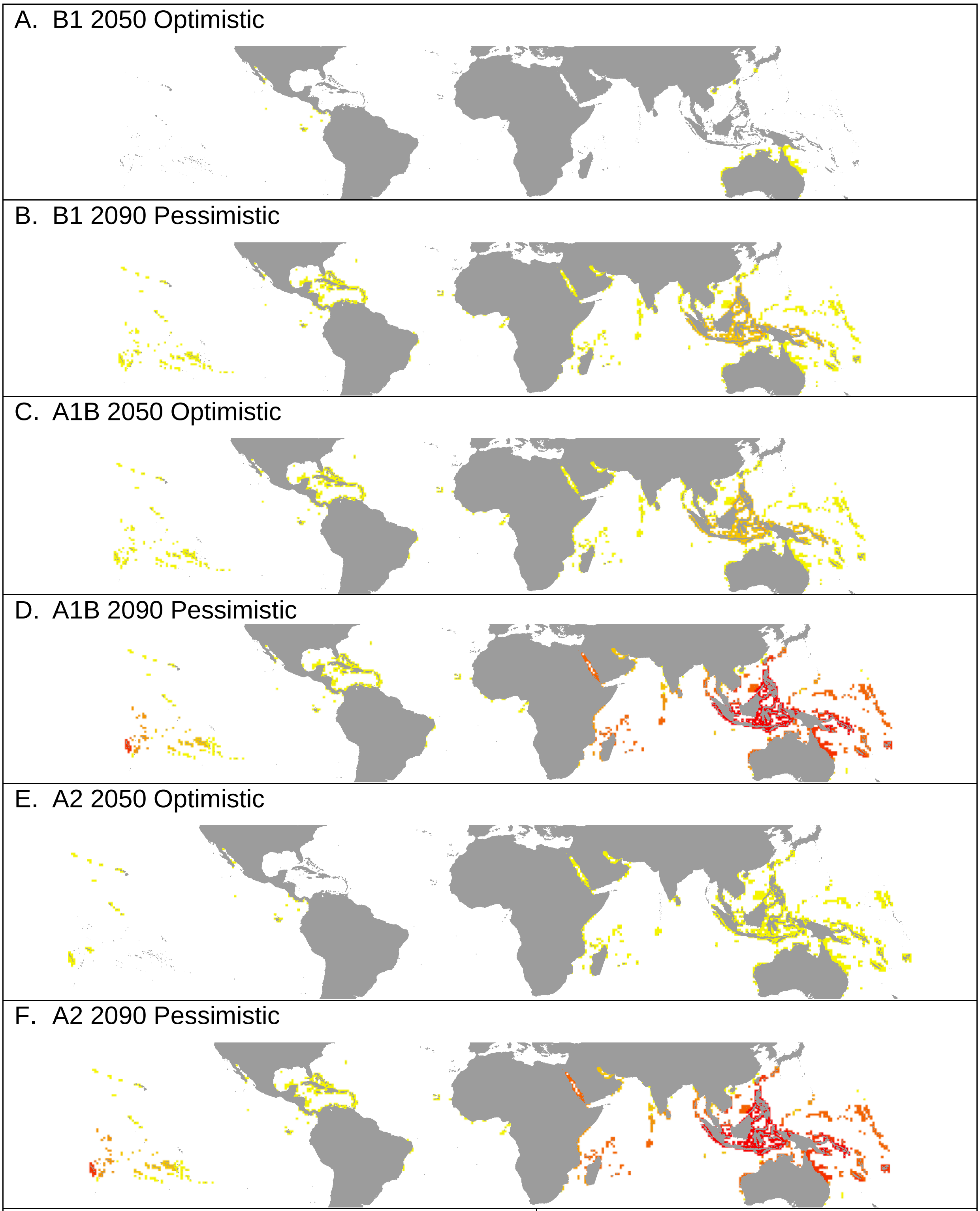

Figure S9: Foci of highly climate change vulnerable corals under three IPCC SRES climate change scenarios for 2050 and 2090. Low range scenario B1, moderate A1B (used as the baseline for all other assessments in this study) and high range A2 are represented by A, C and E respectively for 2050, while B, D and F show the same scenarios for 2090.

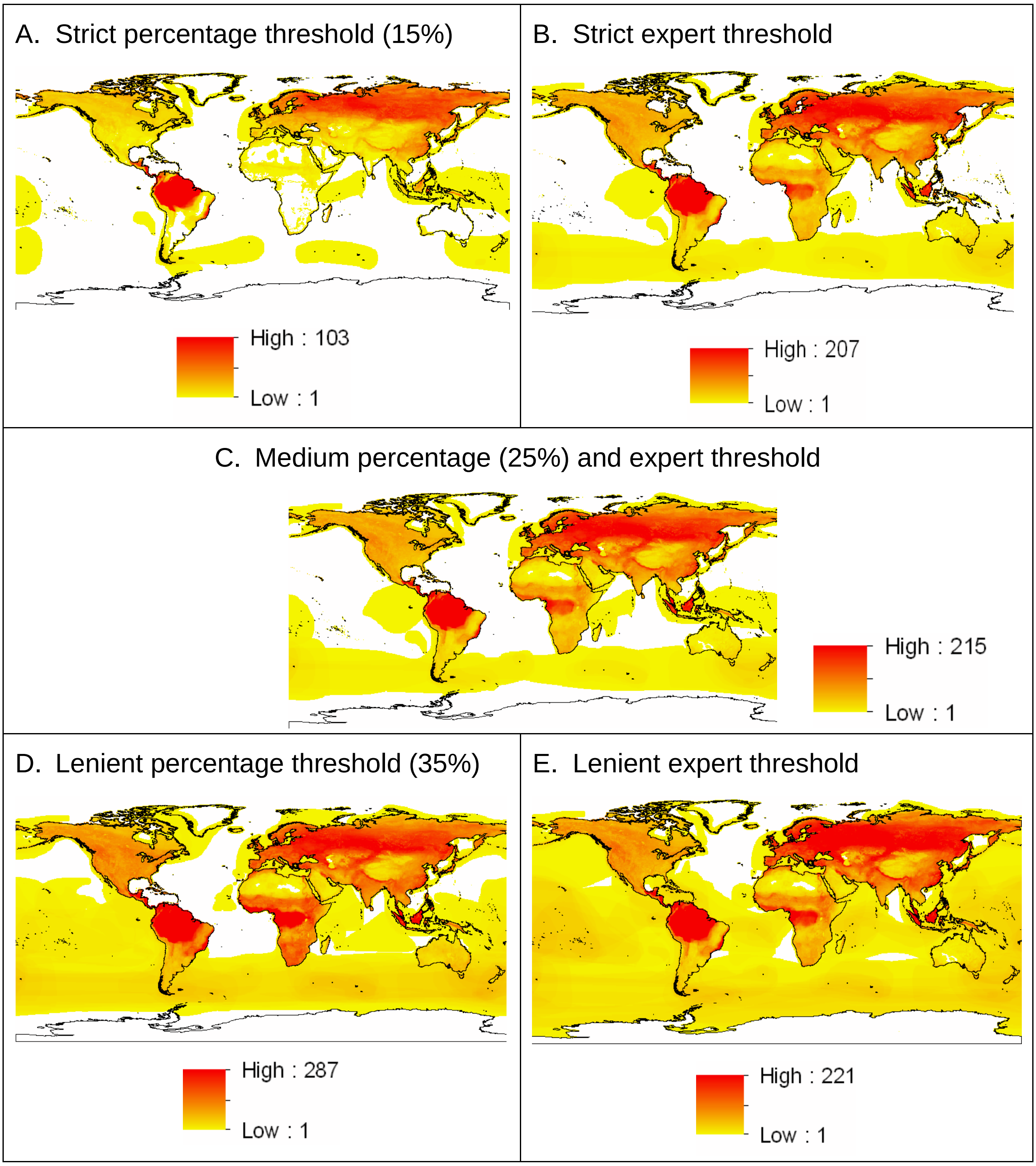

Figure S10: Foci of highly climate change vulnerable birds calculated using five trait threshold scenarios, namely: strict percentage thresholds (A), strict expert thresholds (B), a moderate scenario for percentage and expert thresholds (i.e., as used for the results presented in Table 2 and Figure 2) (C), lenient percentage thresholds (D), and lenient expert thresholds (E). Results are calculated based on an optimistic scenario for unknowns under emission scenario A1B for 2050.

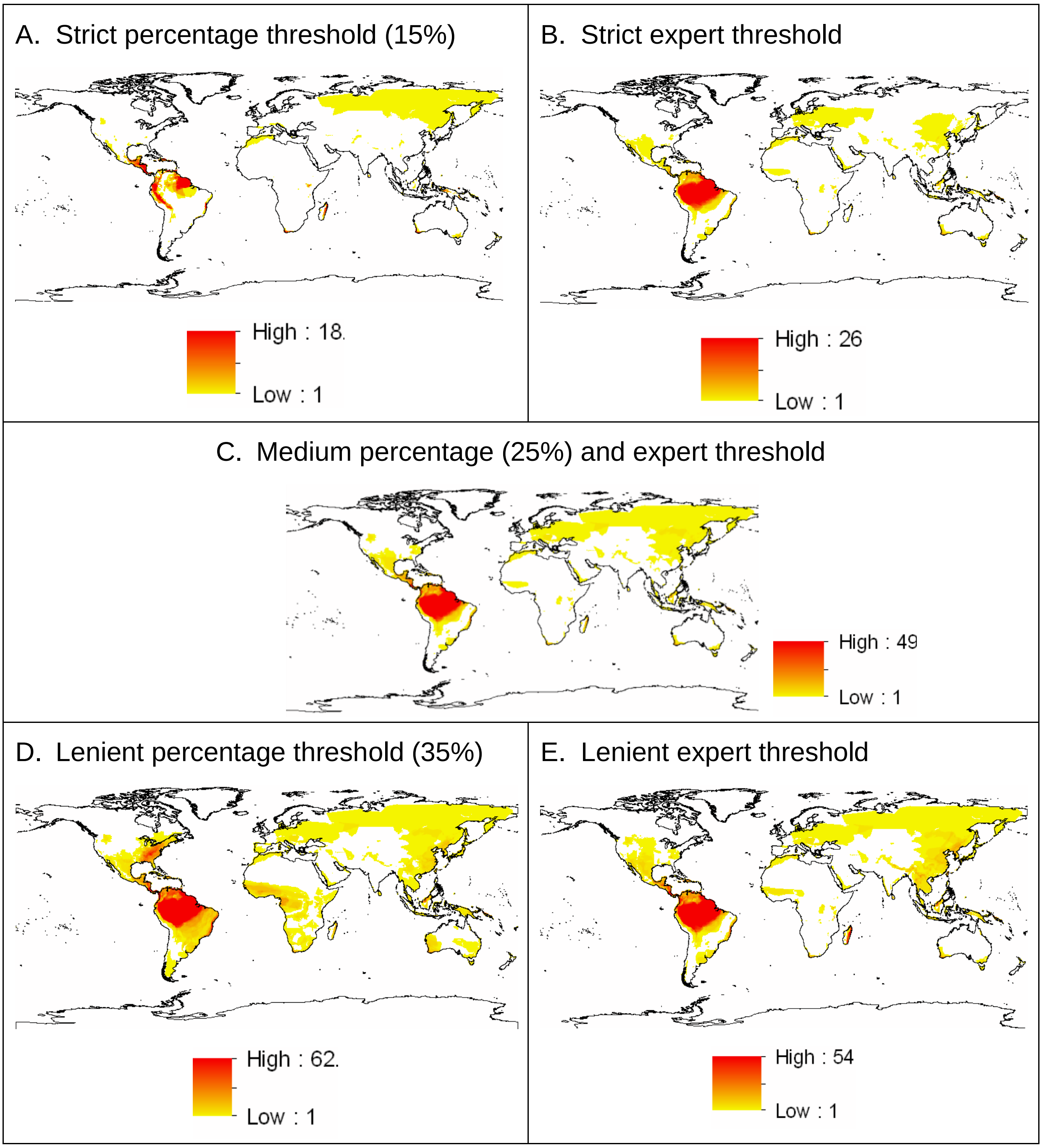

Figure S11: Foci of highly climate change vulnerable amphibians calculated using five trait threshold scenarios, namely: strict percentage thresholds (A), strict expert thresholds (B), a moderate scenario for percentage and expert thresholds (i.e., as used for the results presented in Table 2 and Figure 2) (C), lenient percentage thresholds (D), and lenient expert thresholds (E). Results are calculated based on an optimistic scenario for unknowns under emission scenario A1B for 2050.

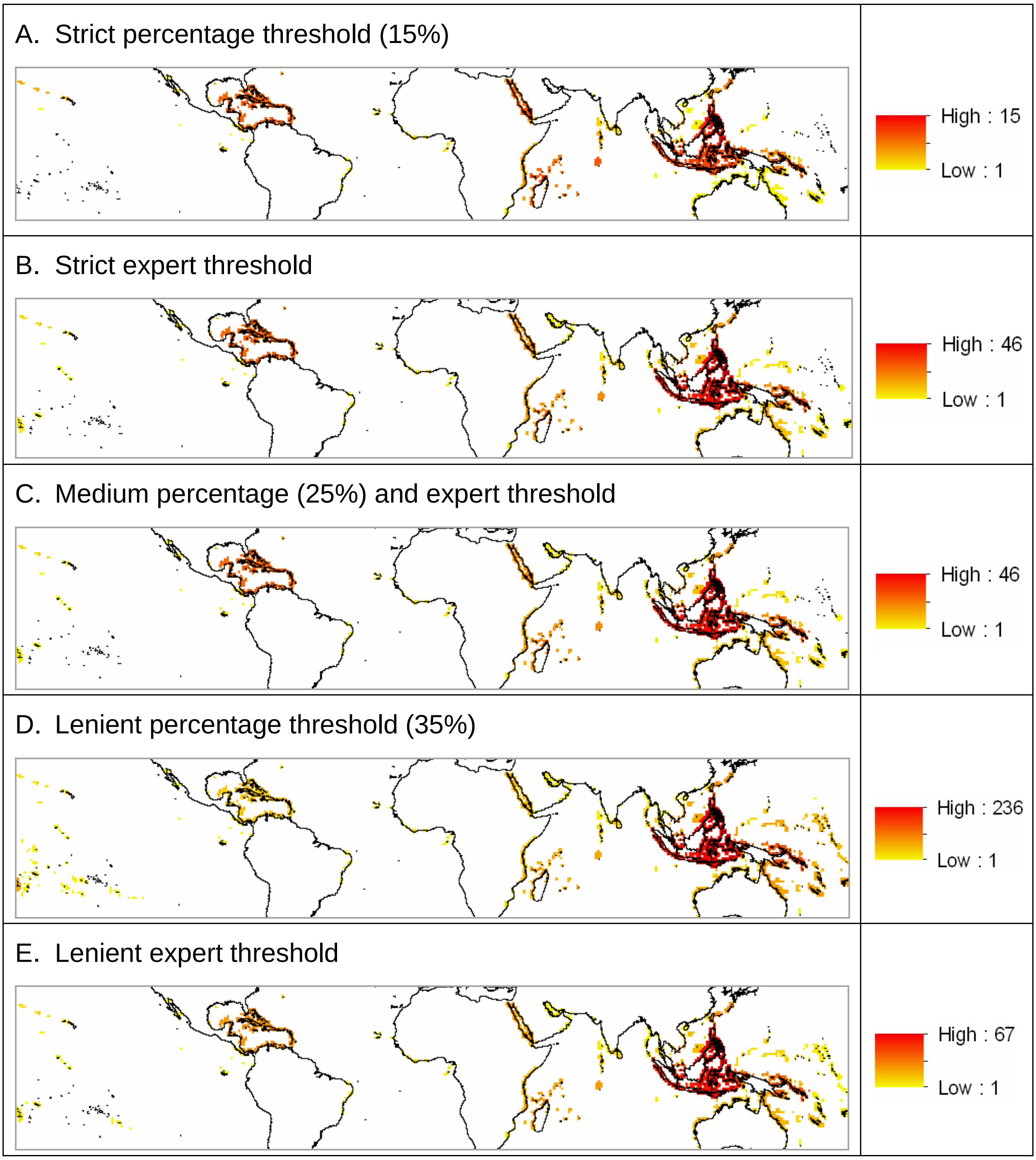

Figure S12: Foci of highly climate change vulnerable corals calculated using five trait threshold scenarios, namely: strict percentage thresholds (A), strict expert thresholds (B), a moderate scenario for percentage and expert thresholds (i.e., as used for the results presented in Table 2 and Figure 2) (C), lenient percentage thresholds (D), and lenient expert thresholds (E). Results are calculated based on an optimistic scenario for unknowns under emission scenario A1B for 2050.

.

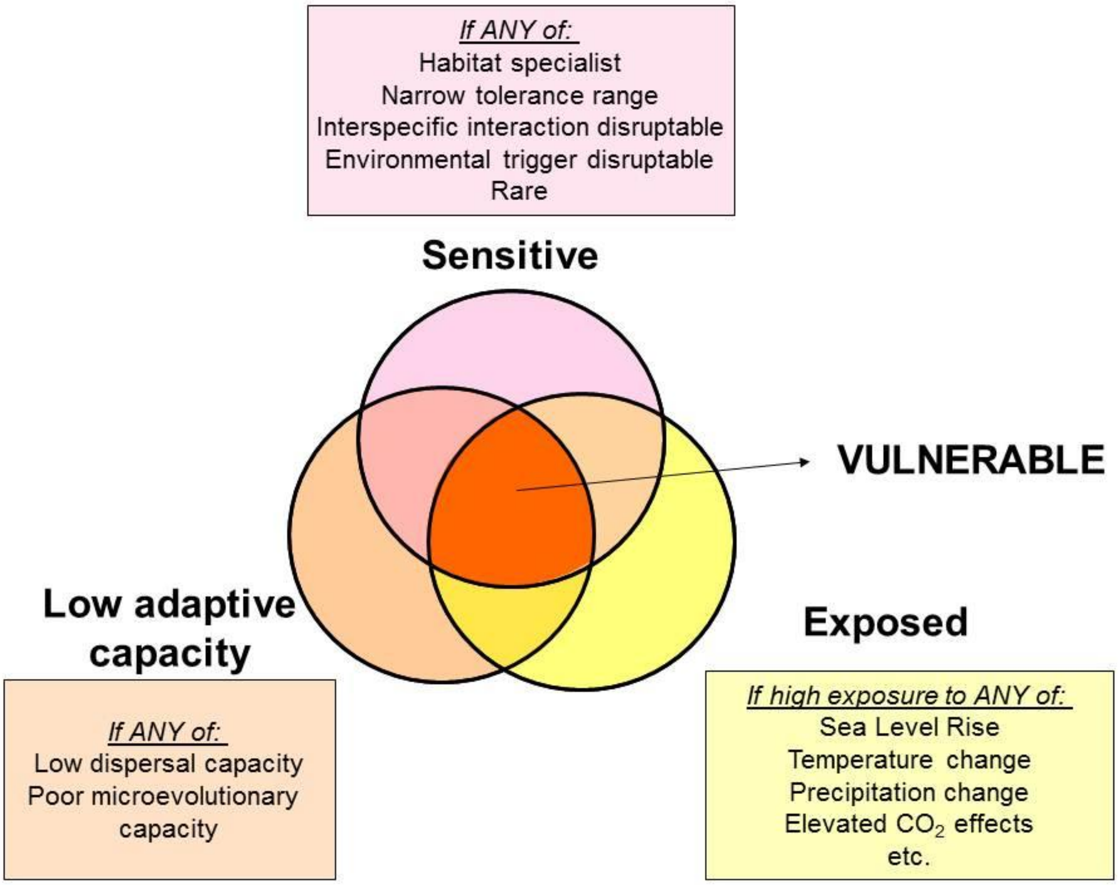

Figure S13: Schematic diagram showing the three dimensions of climate change vulnerability (sensitivity, exposure and low adaptive capacity) and the biological and environmental trait sets contributing to them. The three boxes explain the logic system used to classify species as high in each climate change vulnerability dimension. Species are considered highly climate change vulnerable overall if they score high under all three of sensitivity, exposure and adaptive capacity.

Supporting Tables

Table S1: Traits rendering bird species as of 'high' and 'low/lower' climate change vulnerability, and the number of species qualifying under these categories and as unknown according to each trait.

Trait Group	Trait	Not of high vulnerability		High vulnerability		Unknown
		Threshold	No. species	Threshold	No. species	No. Species
**Sensitivity**						
**a. Specialised habitat and/or microhabitat requirements**	Habitat specialist	Occurs in 2-24 habitats	8,306	Occurs in 1 habitat	1,530	20
	Dependence on a particular microhabitat	No microhabitat dependency known	8,855	Has one or more microhabitat dependencies	1,001	0
	Intolerance of disturbance (forest species)	Scored as 'Low' or 'Medium' or does not occur in forest	7,277	Scored as 'High'	2,575	4
**b. Narrow environmental tolerances or thresholds that are likely to be exceeded due to climate change at any stage in the life cycle**	Narrow temperature tolerance	Highest 75%: Average absolute deviation in temperature across the species' historical range > 1.44 ^{o}C	6,118	Lowest 25%: Average absolute deviation in temperature across the species' historical range ≤ 1.44 ^{o}C	1,974	1,764
	Narrow precipitation tolerance	Highest 75%: Average absolute deviation in precipitation across the species' historical range (lowest 25%) > 46.32 mm	5,997	Lowest 25%: Average absolute deviation in precipitation across the species' historical range (lowest 25%) ≤ 46.32 mm	2,095	1,764
**d. Dependence on interspecific interactions which are likely to be disrupted by climate change**	Declining positive interactions with other species	No dependency	9,767	Dependence on one or more interspecific interactions that are likely to be impacted by climate change (e.g. specialised dependency on army ants)	89	0
**e. Rarity**	Small population size	≥ 10,000 individuals	2,319	< 10,000 individuals	1,084	6,453

	Small population size and heightened sensitivity to threatening processes	All other species	1,993	< 20,000 and [(skewed sex ratio) OR (polygynous or polyandrous breeding system) OR (cooperative breeding system) OR (declining or extremely fluctuating population trend)]	1,410	6,453
**Total**		**719**		**6,290**		**2,847**
**Percentage**		**7%**		**64%**		**29%**
**Exposure**						
**Sea level rise**	Habitat types exposed to sea level inundation	All other species	9,673	Occurs largely in inundation exposed coastal habitats and in no or only one other habitat type	163	20
**Changes in temperature**	Substantial changes in **mean temperature** occur across the species' range	Lowest 75%: Absolute difference between (**mean temperatures** across the species' range for all months) from 1975-2050 < 2.52 °C	6,066	Highest 25%: Absolute difference between (**mean temperatures** across the species' range for all months) from 1975-2050 ≥ 2.52 °C	1,921	1,869
	Substantial changes in **temperature variability** across the species' range	Lowest 75%: Absolute difference between (**average absolute deviation in temperatures** across the species' range for all months) from 1975 to 2050 < 1.2 °C	6,062	Highest 25%: Absolute difference between (**average absolute deviation in temperatures** across the species' range for all months) from 1975 to 2050 ≥1.2 °C	1,925	1,869
**Changes in precipitation**	Substantial changes in **mean precipitation** occur across the species' range	Lowest 75%: Absolute ratio of change in (**mean precipitation** across the species' range for all months) from 1975 to 2050 < 0.49	5,989	Highest 25%: Absolute ratio of change in (**mean precipitation** across the species' range for all months) from 1975 to 2050 ≥ 0.49	1,998	1,869
	Substantial changes in **precipitation variability** across the species' range	Lowest 75%: Absolute ratio of change in (**average absolute deviation in precipitation** across the species' range for all months) from 1975 to 2050 < 0.33	5,835	Highest 25%: Absolute ratio of change in (**average absolute deviation in precipitation** across the species' range for all months) from 1975 to 2050 ≥ 0.33	2,152	1,869
**Total**		**3,082**		**4,920**		**1,854**
**Percentage**		**31%**		**50%**		**19%**
**Low adaptive capacity**						
**f. Poor dispersability**	Low intrinsic dispersal capacity	Maximum intrinsic dispersal distance > 1 km/year	7,863	Maximum intrinsic dispersal distance 0.5- 1 km/year	1,993	0

	Extrinsic barriers to dispersal	No known barriers	9,156	Occurs exclusively on mountaintops, small islands and/or polar edges of land masses	700	0
**g. Poor evolvability**	Low genetic diversity	All other species	9,787	Evidence of low genetic diversity or known genetic bottleneck	69	0
	Slow turnover of generations	Generation length < 6 years	7,356	Generation length ≥ 6 years	2,500	0
	Low reproductive capacity	Mean clutch size >2	3,946	Mean clutch size: ≤ 2	2,414	3,496
**Total**		**2,507**		**5,337**		**2,012**
**Percentage**		**25%**		**54%**		**20%**

Table S2: Traits rendering amphibian species as of 'high' and 'low/lower' climate change vulnerability, and the number of species qualifying under these categories and as unknown according to each trait.

Trait Group	Trait	Not of high vulnerability		High vulnerability		Unknown
		Threshold	No. species	Threshold	No. species	No. species
**Sensitivity**						
**a. Specialised habitat and/or microhabitat requirements**	Habitat specialist	Occurs in 2-33 habitats	4,539	Occurs in 1 habitat	1,509	156
	Dependence on a particular microhabitat	All other species	5,085	Larval development and freshwater dependent and occurs exclusively in an unbuffered habitat (i.e. not forest)	955	164
**b. Narrow environmental tolerances or thresholds that are likely to be exceeded due to climate change at any stage in the life cycle**	Narrow temperature tolerance (adults)	Highest 75%: **Average absolute deviation** in temperature across the species' historical range > 1.20 °C	4,556	Lowest 25%: **Average absolute deviation** in temperature across the species' historical range ≤1.20 °C	1,520	128
	Narrow precipitation tolerance (adults)	Highest 75%: **Average absolute deviation** in precipitation across the species' historical range > 45.84 mm	4,557	Lowest 25%: **Average absolute deviation** in precipitation across the species' historical range ≤ 45.84 mm	1,519	128
**c. Dependence on a specific environmental trigger that's likely to be disrupted by climate change**	Dependence on a specific environmental trigger that's likely to be disrupted by climate change	All other species	4,113	Explosive breeder on rainfall or increased water availability cue (not in forest)	316	1,775
**d. Dependence on interspecific interactions which are likely to be disrupted by climate change**	Increasing negative interactions with other species	All other species	4,897	(Chytridiomycosis related decline recorded) or (vulnerable to enigmatic decline) or (likely future infection (in a genus with a recorded infection and is freshwater dependent and in subtropical or tropical (forest, shrubland or grassland habitats))	1,307	0
Total		**1,365**		**4,453**		**386**
Percentage		**22.00%**		**71.80%**		**6.20%**

**Exposure**						
**Sea level rise**	Habitat types exposed to sea level inundation	All other species		Occurs largely in inundation exposed coastal habitats and in no or only one other habitat type	4	156
**Changes in temperature**	Substantial changes in **mean temperature** occur across the species' range	Lowest 75%: Absolute difference between (**mean temperatures** across the species' range for all months) from 1975-2050 < 2.96 ^{o}C		Highest 25%: Absolute difference between (**mean temperatures** across the species' range for all months) from 1975-2050 $\geq$ 2.96 ^{o}C	1,515	145
	Substantial changes in **temperature variability** across the species' range	Lowest 75%: Absolute difference between (**average absolute deviation in temperature** across the species' range for all months) from 1975 to 2050 < 1.93 ^{o}C		Highest 25%: Absolute difference between (**average absolute deviation in temperature** across the species' range for all months) from 1975 to 2050 $\geq$ 1.93 ^{o}C	1,515	145
**Changes in precipitation**	Substantial changes in **mean precipitation** occur across the species' range	Lowest 75%: Absolute ratio of change in (**mean precipitation** across the species' range for all months) from 1975 to 2050 < 0.59		Highest 25%: Absolute ratio of change in (**mean precipitation** across the species' range for all months) from 1975 to 2050 $\geq$ 0.59	1,515	145
	Substantial changes in **precipitation variability** across the species' range	Lowest 75%: Absolute ratio of change in (**average absolute deviation in precipitation** across the species' range for all months) from 1975 to 2050 < 0.65		Highest 25%: Absolute ratio of change in (**average absolute deviation in precipitation** across the species' range for all months) from 1975 to 2050 $\geq$ 0.65	1,515	145
Total		**2,642**		**3,356**		**206**
Percentage		**42.60%**		**54%**		**3.30%**
**Low adaptive capacity**						
**f. Poor dispersability**	Low intrinsic dispersal capacity	All other species	4,522	Has not become established outside its natural range, and not associated with flowing water, and range size $\leq$ 4,000 km2	1,569	113
	Extrinsic barriers to dispersal	All other species	3,900	Occurs exclusively on mountaintops, small islands, polar edges of land masses and/or polar edges of suitable natural habitat	745	1,559

**g. Poor evolvability**	Low reproductive capacity	All other species	899	Annual reproductive output ≤ 50 or viviparous	2,073	3,232
Total		**2,898**		**3,233**		**73**
Percentage		**46.70%**		**52.10%**		**1.20%**

Table S3: Traits rendering <u>coral</u> species as of 'high' and 'low/lower' climate change vulnerability, and the number of species qualifying under these categories and as unknown according to each trait.

Trait Group	Trait	Not of high vulnerability		High vulnerability		Unknown
		Threshold	No. species	Threshold	No. species	No. species
**Sensitivity**						
**a. Specialised habitat and/or microhabitat requirements**	Habitat specialist	Occurs in 14-32 habitats	605	Occurs in <=13 habitats	192	0
	Dependence on a particular microhabitat	Depth range > 14m	570	Depth range <= 14m	192	35
**b. Narrow environmental tolerances or thresholds that are likely to be exceeded due to climate change at any stage in the life cycle**	Narrow temperature tolerance - larvae	All other species	658	Broadcast spawning and/or brooding are the only known method(s) of reproduction	137	2
	Evidence of exceedance of tolerance - adults	All other species	475	Evidence of past high temperature mortality of > 30% of local population on a reef or reef tract	322	0
	Lower buffering from depth	Maximum depth ≥ 20m	578	Maximum depth < 20m	188	31
**d. Dependence on interspecific interactions which are likely to be disrupted by climate change**	Disruption of symbioses with Zooxanthellae algae	All other species	58	Obligate Zooxanthellae interaction and {(not known to have clades D, C1 or C15) or (known to have D, C1 or C15 but not known to 'shuffle' Zooxanthellae)}	738	1
**e. Rarity**	Rarity	Not rare	595	Rare (geographically restricted or sparsely distributed)	196	6
		**1**		**796**		**0**
		**0.10%**		**99.90%**		**0.00%**
**Exposure**						
**Temperature change**	Exposure to temperatures known to cause bleaching	**Lowest 75%:** Mean probability of severe bleaching across species' range (/10years) < 8.48	518	**Highest 25%:** Mean probability of severe bleaching across species' range (/10years) ≥ 8.48	184	95
**Elevated CO_2**	Exposure to low aragonite saturation	**Lowest 75%:** Proportion of species' range	529	**Highest 25%:** Proportion of species' range with	177	91

	states	with aragonite saturation ≤3 by 2050 < 95.29%		aragonite saturation ≤ 3 by 2050 ≥ 95.29%		
		**447**		**271**		**79**
		**56.10%**		**34.00%**		**9.90%**
**Low adaptive capacity**						
**f. Poor dispersability**	Low intrinsic dispersal capacity	Maximum time to settlement of larvae > 14 days	521	Maximum time to settlement of larvae ≤ 14 days	72	204
	Extrinsic barriers to dispersal	No known barriers	669	Dispersal likely to be retarded by currents and/or temperature	117	11
**g. Poor evolvability**	Slow turnover of generations	Typical colony longevity < 50 years	771	Typical colony longevity ≥ 50 years	13	13
	Low growth rate	Typical maximum growth rate > 30 mm per year	495	Typical maximum growth rate ≤ 30 mm year	293	9
		**373**		**420**		**4**
		**46.80%**		**52.70%**		**0.50%**

Table S4: The number and percentage of bird, amphibian and coral families with significantly more and less highly climate change vulnerable species than expected from the observed overall frequency in each group (based on an optimistic scenario for missing data).

Group	No. of families (% highly vulnerable)	No. of families **more** vulnerable than expected (%)	No. of families **less** vulnerable than expected (%)	Number of families not different (%)
Birds	192 (2%)	38 (20%)	439 (20%)	115 (60%)
Amphibians	60 (22%)	11 (18%)	21 (35%)	28 (47%)
Corals	21 (15%)	4 (19%)	3 (14%)	14 (67%)

Table S5: Summary of the 5 most and least climate change vulnerable bird families. Percentages represent the proportions of species qualifying as high under each climate change vulnerability dimension (i.e., sensitivity, exposure, low adaptive capacity and overall climate change vulnerability). Climate change vulnerability traits are listed where they characterise more than 25% of species in the family.

BIRDS	No. of species in family	Mean Sensitivity	Mean Exposure	Mean Low Adaptive Capacity	Mean OVER-ALL Vulnera-bility
**Five most vulnerable families**					
**Thamnophilidae** (antbirds)	210	**87.1%**<br>• Habitat specialists<br>• Microhabitat requirements<br>• Forest dependent<br>• Narrow temperature niches	**61.9%**<br>Facing relatively large changes in:<br>• Temperature variability<br>• Precipitation variability	**79.1%**<br>• Short maximum dispersal distances<br>• Slow turnover of generations	**47.1%**
**Trogonidae (trogons)**	40	**100%**<br>• Microhabitat requirements<br>• Forest dependent	**67.5%**<br>Facing relatively large changes in:<br>• Precipitation variability<br>• Temperature variability<br>• Mean precipitation	**100%**<br>• Short maximum dispersal distances<br>• Slow turnover of generations	**67.5%**
**Bucerotidae (hornbills)**	55	**100%**<br>• Microhabitat requirements<br>• Forest dependent<br>• Narrow temperature niches	**60.0%**<br>Facing relatively large changes in:<br>• Precipitation variability	**100%**<br>• Short maximum dispersal distances<br>• Slow turnover of generations	**60.0%**
**Pipridae** (manakins)	54	**90.7%**<br>• Forest dependent<br>• Narrow temperature niches<br>• Habitat specialists	**72.2%**<br>Facing relatively large changes in:<br>• Temperature variability<br>• Precipitation variability	**92.6%**<br>• Short maximum dispersal distances<br>• Slow turnover of generations	**59.2%**

**Trochilidae**<br>**(hummingbirds)**	335	**66.2%**<br>• Forest dependent	**76.7%**<br>Facing relatively large changes in:<br>• Temperature variability<br>• Mean precipitation<br>• Precipitation variability<br>• Mean temperature	**71.9%**<br>• Short maximum dispersal distances<br>• Slow turnover of generations	**36.7%**
**Five least vulnerable families**					
**Picidae**<br>(woodpeckers)	218	**79.4%**<br>• Forest dependence<br>• Microhabitat requirements	**45.0%**	**13.3%**	**5.5%**
**Emberizidae**<br>(buntings, American sparrows and allies)	317	**64.7%**<br>• Narrow precipitation niches	**58.4%**<br>Facing relatively large changes in:<br>• Mean temperature<br>• Temperature variability	**18.9%**	**9.2%**
**Estrildidae**<br>(waxbills, grass finches, munias and allies)	137	**29.9%**	**23.4%**	**7.3%**	**4.4%**
**Hirundinidae**<br>(swallows and martins)	83	**37.4%**	**43.4%**	**7.2%**	**1.2%**
**Cisticolidae**<br>(cisticolas and allies)	114	**36.0%**	**26.3%**	**29.8%**	**6.1%**

Table S6: Summary of the 5 most and least climate change vulnerable amphibian families. Percentages represent the proportions of species qualifying as high under each climate change vulnerability dimension (i.e., sensitivity, exposure, low adaptive capacity and overall climate change vulnerability). Climate change vulnerability traits are listed where they characterise more than 25% of species in the family.

AMPHIBIANS	No. of species in family	Mean Sensitivity	Mean Exposure	Mean Low Adaptive Capacity	Mean OVER-ALL Vulnera-bility
**Five most vulnerable families**					
**Straboman-tidae** (robber frogs)	519	**82.1%**<br>• Habitat specialists<br>• Narrow temperature niches<br>• Narrow precipitation niches	**84.8%**<br>Facing relatively large changes in:<br>• Temperature variability<br>• Mean temperature<br>• Precipitation variability	**83.4%**<br>• Slow turnover of generations<br>• Short maximum dispersal distances	**56.8%**
**Plethodontidae** (lungless salamanders)	371	**81.7%**<br>• Habitat specialists<br>• Narrow precipitation niches	**56.9%**<br>Facing relatively large changes in:<br>• Precipitation variability<br>• Mean temperature<br>• Mean precipitation	**90.6%**<br>• Slow turnover of generations<br>• Short maximum dispersal distances	**41.5%**
**Amphignatho-dontidae** (marsupial frogs)	61	**78.7%**<br>• Habitat specialists<br>• Narrow precipitation niches	**82.0%**<br>Facing relatively large changes in:<br>• Temperature variability<br>• Mean temperature<br>• Mean precipitation	**91.8%**<br>• Slow turnover of generations<br>• Short maximum dispersal distances	**63.9%**
**Aromobatidae** (modern frogs)	93	**88.2%**<br>• High disease vulnerability<br>• Narrow temperature niches<br>• Narrow precipitation niches<br>• Habitat specialists	**86.0%**<br>Facing relatively large changes in:<br>• Mean precipitation<br>• Precipitation variability<br>• Temperature variability<br>• Mean temperature	**60.2%**<br>• Slow turnover of generations	**48.4%**
**Eleuthero-dactylidae** (robber frogs)	186	**59.7%**<br>• Habitat specialists	**66.1%**<br>Facing relatively large changes in:	**94.1%**<br>• Slow turnover of generations<br>• Short	**36.9%**

			• Precipitation variability<br>• Mean precipitation	maximum dispersal distances<br>• Barriers to dispersal	
**Five least vulnerable families**					
**Hylidae**<br>(tree frogs and their allies)	851	**87.7%**<br>Disease vulnerability	**28.9%**	**47.6%**<br>Facing relatively large changes in:<br>• Mean precipitation	**11.5%**
**Ranidae**<br>(true frogs)	316	**49.7%**	**22.8%**	**46.8%**<br>Facing relatively large changes in:<br>• Mean temperature	**6.7%**
**Hyperoliidae**<br>(African reed frogs)	210	**83.8%**<br>• Freshwater dependent aquatic larvae<br>• Narrow temperature niche	**54.3%**<br>• Short maximum dispersal distances	**18.6%**	**7.1%**
**Arthroleptidae**<br>(squeakers)	133	**62.4%**<br>• Narrow temperature niche	**48.9%**<br>• Slow turnover of generations	**18.8%**	**4.5%**
**Dicroglossidae**<br>(forked tongued frogs)	163	**45.4%**	•	**49.1%**<br>Facing relatively large changes in:<br>• Mean temperature	**6.8%**

Table S7: Summary of the four families that have mean climate change vulnerability scores that are significantly greater than the mean for all corals, as well as the three with significantly lower mean susceptibilities. Percentages represent the proportions of species qualifying as high under each climate change vulnerability dimension (i.e., sensitivity, exposure, low adaptive capacity and overall climate change vulnerability). Climate change vulnerability traits are listed where they characterise more than 25% of species in the family.

CORALS	No. of species in family	Mean Sensitivity	Mean Exposure	Mean Low Adaptive Capacity	Mean OVER-ALL Vulnera-bility
**Most vulnerable families**					
**Mussidae**<br>(including some brain corals)	13	**100%**<br>• Dependent on heat-intolerant Zooxanthellae	**36.0%**<br>• Facing relatively large increases in bleaching frequency	**98.0%**<br>• Very slow growth rate	**34.0%**
**Meandrinidae**	7	**100%**<br>• Dependent on heat-intolerant Zooxanthellae<br>• Evidence of large-scale past mortality events in response to bleaching	**57.1**<br>• Facing relatively large increases in bleaching frequency	**100%**<br>• Very slow growth rate	**57.1%**
**Astrocoeniidae**	13	**100%**<br>• Dependent on heat-intolerant Zooxanthellae<br>• Habitat specialists	**53.8%**<br>• Facing relatively large increases in bleaching frequency	**69.2%**<br>• Very slow growth rate<br>• Slow turnover of generations	**38.5%**
**Faviidae**<br>(including stony corals)	127	**100%**<br>• Dependent on heat-intolerant Zooxanthellae<br>• Evidence of large-scale past mortality events in response to bleaching<br>• Larvae are sensitive to surface warming	**22.8%**<br>• Facing relatively large increases in bleaching frequency<br>• Facing relatively large decreases in	**88.2%**<br>• Very slow growth rate	**19.7%**

		• Rare	aragonite saturation		
**Least vulnerable families**					
**Agariciidae**	43	**100%**<br>• Dependent on heat-intolerant Zooxanthellae<br>• Evidence of large-scale past mortality events in response to bleaching<br>• Habitat specialists	**32.6%**<br>• Facing relatively large increases in bleaching frequency	**20.9%**	**2.3%**
**Pectiniidae**	27	**100%**<br>• Dependent on heat-intolerant Zooxanthellae<br>• Evidence of large-scale past mortality events in response to bleaching<br>• Habitat specialists	**25.9%**	**18.5%**<br>• Very slow growth rate	**0%**
**Acroporidae**<br>(including staghorn corals)	264	**100%**<br>• Dependent on heat-intolerant Zooxanthellae<br>• Evidence of large-scale past mortality events in response to bleaching<br>• Unbuffered by broad depth ranges<br>• Unbuffered by depth<br>• Habitat specialists	**37.5%**<br>• Facing relatively large decreases in aragonite saturation	**40.2%**<br>• Short maximum dispersal distances	**12.9%**

Table S8: Summary of geographic focal areas (identified in Figure 2 (A, C and E)) **that contain high total numbers of species** that are (i) **highly sensitive and of low adaptive capacity**, (ii) **highly exposed, and both** (i) and (ii).

	**[High sensitivity and low adaptive capacity] and [high exposure]**<br>(purple in Fig. 2)	**High sensitivity and low adaptive capacity only**<br>(blue in Fig. 2)	**High exposure only**<br>(yellow in Fig. 2)
Birds	• Amazon basin and Mesoamerica, eastern Europe through central to eastern Asia, excluding the Tibetan Plateau<br>• Congo basin and tropical West Africa<br>• Himalayas<br>• Malesia	• Northern and eastern North America<br>• Southern oceans north to c.30°S<br>• Australia and New Guinea<br>• Tropical West Africa, Congo basin through sub-Saharan Africa and Madagascar<br>• Southern and Eastern Brazil<br>• Northern Eurasia	• Western USA and Mexico<br>• North Africa and Sahel, excluding Sahara<br>• Indian subcontinent through Indochina to north-eastern Asia
Amphibians	• Amazon basin	• Eastern USA<br>• Tropical and southern Africa<br>• Eastern and northern Australia	• South-western USA and Mexico<br>• Northern South America Temperate Europe and north-western Asia<br>• Indochina to north-eastern Asia<br>• Madagascar
Corals	• Coral Triangle (Philippines to Solomon Islands), Sumatra and Java	• Australia<br>• Pacific Islands<br>• Indian Ocean including East Africa, Madagascar, India and Indochina<br>• Red Sea<br>• South China Sea	

Table S9: Summary of geographic focal areas (identified in Figure 2 (B, D and F)) **that contain high proportions of species**, relative to species richness, that are (i) **highly sensitive and of low adaptive capacity**, (ii) **highly exposed and both** (i) and (ii).

	**[High sensitivity and low adaptive capacity] and [high exposure]** (purple in Fig. 2)	**High sensitivity and low adaptive capacity only** (blue in Fig. 2)	**High exposure only** (yellow in Fig. 2)
Birds	• Greenland, Iceland and north-eastern North America<br>• Northern Eurasia, Black Sea and Himalayas<br>• Southern oceans between c.30-60 ^{o}S<br>• Amazon basin and central Andes<br>• Parts of the Eastern Sahara<br>• Tropical West Africa to Congo basin<br>• Sundaland	• The northern oceans, from c.45°N, extending southwards through the eastern Atlantic to West Africa<br>• The Mediterranean and Red Seas and the Persian Gulf<br>• Australia and parts of New Guinea<br>• The southern oceans, from c. 25°S, extending northwards to the equator in the Eastern Pacific	• Africa, excluding tropical West Africa, the Congo basin and southern East Africa<br>• Southern Eurasia and Arabia<br>• Western USA through Central America<br>• Northern South America and the Andes<br>• New Zealand
Amphibians	• Amazon basin, northern Andes, and Mesoamerica<br>• Eastern Russia and Mongolia<br>• Himalayas<br>• Parts of North Africa<br>• North of the Caspian Sea<br>• Western and eastern Arabia	• Congo basin, southern Africa and western Madagascar<br>• Eastern North America<br>• Eastern and southern South America<br>• Most of Australia and New Guinea<br>• Southern India and parts of Pakistan<br>• The Iberian peninsula	• Northern Europe, Scandinavia and Asia to Malesia, excluding eastern Russia and Mongolia<br>• Peripheral areas of the Sahara<br>• Western North America<br>• Central and southern Andes<br>• Indonesia<br>• South-western Pakistan and southern Iran
Corals	• The Caribbean (this region contains high proportions of highly exposed species, approximately half of which are also of high sensitivity and low adaptive capacity)	• All reefs from Red Sea and East Africa through Asia to central Pacific	• The Caribbean (this region contains high proportions of highly exposed species, approximately half of which are also of high sensitivity and low adaptive capacity)<br>• East Atlantic coast and islands of Cape Verde, Sao Tome and Principe

Table S10: The numbers and percentages of bird, amphibian and coral species with various combinations of threat status (according to the IUCN Red List) **and high climate change vulnerability**. Optimistic scores are based on climate change vulnerability scores calculated on the assumption that unknown trait values reflect 'not high' scores; pessimistic scores are based on the assumption that unknown trait values reflect high scores. Independence between numbers of species that are threatened by non-climatic stressors and highly climate change vulnerable was tested using Pearson's Chi-square test (d.f. = 1); total species numbers (n), Chi-squared coefficients and P values are shown for each taxonomic group.

	**Birds**		**Amphibians**		**Corals**	
	Optimistic	Pessimistic	Optimistic	Pessimistic	Optimistic	Pessimistic
Vulnerable	*2,323*	*4,890*	*1,368*	*2,740*	*121*	*247*
Threatened	*1,222*	*1,222*	*1,878*	*1,878*	*220*	*220*
Threatened and vulnerable	**608** (6%)	**851** (9%)	**670** (11%)	**933** (15%)	**47** (6%)	**73** (9%)
Threatened and not vulnerable	**614** (6%)	**371** (4%)	**1,208** (19%)	**945** (15%)	**173** (22%)	**147** (18%)
Not threatened and vulnerable	**1,715** (17%)	**4,039** (41%)	**698** (11%)	**1,807** (29%)	**74** (9%)	**174** (22%)
Not threatened and not vulnerable	**6,919** (70%)	**4,595** (47%)	**3,628** (58%)	**2,519** (41%)	**503** (63%)	**403** (51%)
Total species	*9,856*	*9,856*	*6,204*	*6,204*	*797*	*797*
Chi-squared coefficient	530.95	223.78	290.93	33.22	9.02	0.68
Significance	P<0.001	P<0.001	P<0.001	P<0.001	P<0.01	n.s.
What % of vulnerable species is threatened?	*26*	*17*	*49*	*34*	*39*	*30*
What % of vulnerable species is not threatened?	*74*	*83*	*51*	*66*	*61*	*70*
What % of threatened species is vulnerable?	*50*	*70*	*36*	*50*	*21*	*33*
What % of threatened species is not vulnerable?	*50*	*30*	*64*	*50*	*79*	*67*

Table S11: Summary of the geographic focal areas identified in Figure 3 that contain <u>high total numbers</u> of species that are threatened (according to the IUCN Red List^{TM}), **highly climate change vulnerable and high numbers of both.**

	**Threatened and vulnerable** (purple in Fig. 3)	**Vulnerable only** (yellow in Fig. 3)	**Threatened only** (blue in Fig. 3)
Birds	• Northern Andes<br>• South-eastern South America<br>• The Himalayas and Indian subcontinent<br>• Sundaland (Indonesia, Malaysia and southern Thailand) and the Philippines<br>• South eastern China<br>• The southern oceans between c. 30-60°S<br>• South eastern Russia, and parts of north-central Asia<br>• Much of sub-Saharan Africa, excluding Congo basin<br>• Parts of central and southern North America	• Amazon basin and south western South America<br>• Parts of northern, western and southern North America<br>• Central America<br>• Europe, Scandinavia and much northern and central Asia<br>• The Congo basin and parts of south-central Africa<br>• Eastern and south western Australia<br>• Guinea	• Throughout the northern and central Pacific<br>• The southern Atlantic and Indian oceans from c. 20-30 ^{o}S, and some areas south of 50°S<br>• Western Arabian peninsula<br>• Parts of central and South East Asia
Amphibians	• Parts of northern Andes and Mesoamerica	• Amazon basin<br>• Southern USA to Mesoamerica<br>• Eurasia excluding central Asia and eastern China<br>• Madagascar<br>• New Guinea<br>• South-western Australia<br>• The southern tip of Africa<br>• Western Sahel<br>• North-western Africa	• South-eastern China<br>• Parts of western USA, Mexico Mesoamerica<br>• Central Andes<br>• Parts of tropical West Africa<br>• Highlands of East Africa<br>• South-eastern Australia
Corals	• Coral triangle, Sumatra and Java, extending northwards to the East China sea<br>• The Red Sea<br>• East Africa and central Indian ocean islands including Madagascar<br>• The Great Barrier Reef and northern Australia	• Caribbean<br>• Islands of the eastern Atlantic	• Arabian gulf<br>• Northern Indian ocean (Bay of Bengal and Andaman sea)<br>• The Pacific ocean, excluding the Coral Triangle

Table S12: Summary of the geographic focal areas identified in Figure S6 that contain high relative numbers of species that are threatened (according to the IUCN Red List™), **climate change vulnerable, and high numbers of both.**

	**Threatened and vulnerable** (purple in Suppl. Fig. 6)	**Vulnerable only** (yellow in Suppl. Fig. 6)	**Threatened only** (blue in Suppl. Fig. 6)
• Birds	• Oceans between c.30- 60°S<br>• Northern Pacific<br>• Peripheral areas of the Sahara<br>• The eastern Mediterranean	• Amazon basin and Andes<br>• Northern North America, extending to Greenland and Iceland<br>• Europe and northern and central Eurasia, including the Himalayas<br>• The Congo basin	• Northern and central Pacific<br>• Western Atlantic and Caribbean and south-central and north-western Atlantic<br>• Central Sahara<br>• Arabian Peninsula<br>• Indian subcontinent, extending to Indochina<br>• Madagascar and central Indian Ocean<br>• West-central Australia
• Amphibians	• Parts of northern Andes and Mesoamerica	• Amazon basin to Argentina<br>• Parts of western and southern USA, Mexico and Mesoamerica<br>• Eurasia excluding central Asia and eastern China<br>• South-western Australia<br>• North-western Africa	• Parts of western and southern USA, Mexico and Mesoamerica<br>• The central Andes and parts of the southern Andes<br>• Parts of tropical West Africa<br>• Highlands of East Africa<br>• Madagascar<br>• South-eastern China<br>• South-eastern Australia
• Corals		• Caribbean<br>• East Atlantic coast and islands of Cape Verde, Sao Tome and Principe	• South-western Pacific and Australia<br>• Coral Triangle<br>• Indian Ocean

Table S13: Summary of the numbers of species and size of geographic area uniquely identified by each of the biological traits used to assess overall climate change vulnerability of birds. Traits highlighted in yellow identify the five most influential traits for uniquely identifying numbers of species and those in red text identify these traits for geographic areas. Trait and trait group descriptions are shortened versions; full titles are shown in Table S1.

**Vulner-ability dimension**	**Trait Group**	**Trait**	Species qualifying under this trait	Species qualifying exclusively based on this trait	% of total suscept-ible species	**Rank of import-ance by species**	Geographic area (km^2) identified exclusively by this trait	% of total suscept-ible area	**Rank of import-ance by area**	Species with unknown score for this trait
**Sensitivity**	Specialised habitat and/or microhabitat requirements	Habitat specialist	1,530	19	0.8	**14**	93,964,466	17.7	**9**	20
		Microhabitat required	1,001	56	2.4	**11**	94,788,888	17.8	**8**	0
		Intolerance of disturbance	2,575	234	10.1	**8**	93,964,812	17.7	**9**	4
	Narrow environmental tolerances	Narrow temperature tolerance	1,974	198	8.5	**9**	127,174,896	23.9	**1**	1,764
		Narrow precipitation tolerance	2,095	0	0.0	**16**	93,963,082	17.7	**9**	1,764
	Interspecific interactions	Declining positive interactions with other species	89	18	0.8	**14**	108,493,748	20.4	**5**	0
	Rarity	Small population size	1,084	0	0.0	**16**	93,963,082	17.7	**9**	6,453
		Small effective population size	1,410	24	1.0	**13**	94,248,845	17.7	**9**	6,453
**Exposure**	Sea level rise	Exposed to sea level rise	163	47	2.0	**12**	20,768,671	3.9	**11**	20
	Changes in temperature	Changes in mean temperature	1,921	280	12.1	**6**	101,564,515	19.1	**7**	1,869
		Changes in temperature variability	1,925	245	10.5	**7**	66,141,671	12.4	**4**	1,869
	Changes in precipitation	Changes in mean precipitation	1,998	337	14.5	**4**	3,051,713	0.6	**12**	1,869
		Changes in precipitation variability	2,152	298	12.8	**5**	62,813,190	11.8	**10**	1,869
**Low adaptive capacity**	Poor dispersability	Limited dispersal ability	1,993	524	22.6	**1**	106,611,380	20.1	**6**	0
		Geographical barriers	700	112	4.8	**10**	109,168,370	20.5	**3**	0
	Poor evolvability	Low genetic diversity	69	5	0.2	**15**	106,610,688	20.1	**6**	0
		Slow turnover of generations	2,500	411	17.7	**3**	106,649,089	20.1	**6**	0
		Low reproductive output	2,414	439	18.9	**2**	109,374,216	20.6	**2**	3,496
**Total numbers of vulnerable species/area**				**2,323**			**531,435,729**			
**Total number of species**				**9,856**						

Table S14: Summary of the numbers of species and size of geographic area uniquely identified by each of the biological traits used to assess overall climate change vulnerability of amphibians. Traits highlighted in yellow identify the five most influential traits for uniquely identifying numbers of species and those in red text identify these traits for geographic areas. Trait and trait group descriptions are shortened versions; full titles are shown in Table S2.

	**Trait Group**	**Trait**	Species qualifying under this trait	Species qualifying exclusively based on this trait	% of total suscept-ible species	**Rank of import-ance by species**	Geographic area (km^2) identified exclusively by this trait	% of total suscept-ible area	**Rank of import-ance by area**	Species with unknown score for this trait
**Sensitivity**	Specialised habitat and/or microhabitat requirements	Habitat specialist	1,509	313	20.4	**3**	427,953	0.6	**10**	156
		Microhabitat required	955	31	2.0	**12**	4,956,569	6.8	**5**	164
	Narrow environmental tolerances	Narrow temperature tolerance	1,520	188	12.3	**6**	811,622	1.1	**10**	128
		Narrow precipitation tolerance	1,520	184	12.0	**7**	39,127,730	53.8	**2**	128
	Environmental trigger	Breeder on rainfall cue	316	1	0.1	**13**	0	0.0	**11**	1,775
	Interspecific interaction	Disease	1,307	169	11.0	**9**	1,136,479	1.6	**9**	0
**Exposure**	Sea level rise	Exposed to sea level rise	4	1	0.1	**13**	346	0.0	**11**	156
	Changes in temperature	Changes in mean temperature	1,515	256	16.7	**4**	42,763,078	58.8	**1**	145
		Changes in temperature variability	1,515	238	15.5	**5**	4,680,839	6.4	**6**	145
	Changes in precipitation	Changes in mean precipitation	1,515	113	7.4	**10**	1,589,686	2.2	**9**	145
		Changes in precipitation variability	1,515	173	11.3	**8**	3,817,669	5.3	**7**	145
**Low adaptive capacity**	Poor dispersability	Limited dispersal ability	1,569	411	26.8	**2**	19,939,059	27.4	**4**	113
		Geographical barriers	745	106	6.9	**11**	2,369,480	3.3	**8**	1,559
	Poor evolvability	Slow turnover of generations	2,073	520	34.0	**1**	28,501,569	39.2	**3**	3,232
**Total number of vulnerable species/area**				**1,531**			**72,683,082**			
**Total number of species**				**6,204**						

Table S15: Summary of the numbers of species and size of geographic area uniquely identified by each of the biological traits used to assess overall climate change vulnerability of corals. Traits highlighted in yellow identify the five most influential traits for uniquely identifying numbers of species and those in red text identify these traits for geographic areas. Trait and trait group descriptions are shortened versions; full titles are shown in Table S3.

	Trait Group	Trait	Species qualify-ing under this trait	Species qualifying exclusively based on this trait	% of total vulnerable species	Rank of import-ance by species	Geographic area (km^2) identified exclusively by this trait	% of total vulnerable area	Rank of import-ance by area	Species with unknown score for this trait
Sensitivity	Specialised habitat and/or microhabitat	Habitat specialist	192	2	1.7	6	0	0.0	7	0
		Microhabitat required	192	0	0.0	8	0	0.0	7	35
	Narrow environmental tolerances	Narrow temperature tolerance - larvae	137	2	1.7	6	0	0.0	7	2
		Buffering of temperature change	188	0	0.0	8	0	0.0	7	31
		Evidence of bleaching	322	20	16.5	5	22,487	0.6	4	0
	Interspecific interactions	No heat tolerant Zooxanthellae and/or not shufflers	739	1	0.8	7	0	0.0	7	1
	Rarity	Lower ability to recover following declines	196	0	0.0	8	0	0.0	7	6
Exposure	Temperature change	Frequent exposure to temperatures known to cause bleaching	184	52	43.0	2	583,980	16.6	1	95
	Ocean acidification	Exposure to low aragonite saturation states	177	32	26.4	3	462,894	13.1	2	91
Low adaptive capacity	Poor dispersability	Limited dispersal ability	72	22	18.2	4	6,573	0.2	5	204
		Dispersal barriers	117	22	18.2	4	4,152	0.1	6	11
	Poor evolvability	Slow turnover of generations	13	1	0.8	7	0	0.0	7	11
		Slow growth rate	293	56	46.3	1	83,030	2.4	3	9
**Total numbers of vulnerable species/area**				**121**	**15.2**		**3,521,181**			
**Total number of species**				**797**						

Table S16: Traits rendering <u>bird</u> species as of 'high' climate change vulnerability, and the number of species qualifying under these categories and as unknown, according to three trait threshold scenarios, namely more lenient thresholds, the original or moderate thresholds (i.e., as used for the results presented in Table 2 and Figure 2) and stricter thresholds. Thresholds for traits indicated with a (P) and highlighted in blue were selected based on arbitrary percentage thresholds (35%, 25% and 15%) while those indicated by an (E) and highlighted in green were selected based on experts' judgements. All results shown are based on an optimistic scenario for 2050 under the A1B emission scenario.

**Trait Group**	**Trait**	**More Lenient Estimate**		**Original Estimate**		**Stricter Estimate**		Un-known
		Threshold	No. spp.	Threshold	No. spp.	Threshold	No. spp.	No. spp.
**Sensitivity**								
A. Specialised habitat and/or micro-habitat require-ments	Habitat specialist	NA	1,530	Occurs in 1 habitat	1,530	NA	1,530	20
	Dependence on a particular microhabitat	NA	1,001	Has one or more microhabitat dependencies	1,001	NA	1,001	0
	Intolerance of disturbance	NA	2,575	Scored as 'High'	2,575	NA	2,575	4
B. Narrow environ-mental tolerances	Narrow temperature tolerance **(P)**	Lowest 35%: ≤ 1.83 °C	2,772	Lowest 25%: Average absolute deviation in temperature across the species' historical range ≤ 1.44 °C	1,974	Lowest 15%: ≤ 1.13 °C	1,182	1,764
	Narrow precipitation tolerance **(P)**	Lowest 35%: ≤ 56.09 mm	2,904	Lowest 25%: Average absolute deviation in precipitation across the species' historical range ≤ 46.32 mm		Lowest 15%: ≤ 35.23 mm		
D. Depen-dence on inter-specific inter-actions	Declining positive interactions with other species	NA	89	Dependence on one or more interspecific interactions that are likely to be impacted by climate change (e.g. specialised dependency on army ants)	89	NA	89	0

E. Rarity	Small population size **(P)**	< 20,000 individuals	1,410	< 10,000 individuals	1,084	< 2,500 individuals	477	6,453
	Small population size and heightened sensitivity to threatening processes **(E)**	**< 50,000** and [(skewed sex ratio)<br>OR (polygynous or polyandrous breeding system)<br>OR (co-operative breeding system)<br>OR (declining or extremely fluctuating population trend)]	1,423	**< 20,000** and [(skewed sex ratio)<br>OR (polygynous or polyandrous breeding system)<br>OR (cooperative breeding system)<br>OR (declining or extremely fluctuating population trend)]	1,410	**< 10,000** and [(skewed sex ratio)<br>OR (polygynous or polyandrous breeding system)<br>OR (co-operative breeding system)<br>OR (declining or extremely fluctuating popn trend)]	869	6,453
**Low adaptive capacity**								
A. Poor dispers-ability	Low intrinsic dispersal capacity **(E)**	Maximum intrinsic dispersal distance <=1.5 km/year	2,628	Maximum intrinsic dispersal distance <= 1 km/year	1,993	Maximum intrinsic dispersal distance <= 0.5 km/year	1,510	0
	Extrinsic barriers to dispersal	NA	700	Occurs exclusively on mountaintops, small islands and/or polar edges of land masses	700	NA	700	0
B. Poor evolva-bility	Low genetic diversity	NA	69	Evidence of low genetic diversity or known genetic bottleneck	69	NA	69	0
	Slow turnover of generations **(P)**	Generation length ≥ 5.7 years	2,809	Generation length ≥ 6 years	2,500	Generation length ≥ 8 years	1,196	0
	Low reproductive capacity **(P)**	Mean clutch size: ≤ 2.5	3,288	Mean clutch size: ≤ 2	2,414	Mean clutch size: ≤ 1.5	749	3,496
**Exposure**								
A. Sea level rise	Habitat types exposed to sea level inundation **(E)**	Occurs largely in inundation exposed coastal habitats and **up to**	425	Occurs largely in inundation exposed coastal habitats **and up to 1 other habitat type**	163	Occurs in inundation exposed coastal habitats only	42	20

		**2 other habitat types**						
B. Changes in temp-erature	Substantial changes in **mean temper-ature** occur across the species' range **(P)**	Highest 35%: ≥ 2.1 °C	2,735	Highest 25%: Absolute difference between (**mean temperatures** across the species' range for all months) from 1975-2050 ≥ 2.5 °C	1,921	Highest 15%: ≥ 3.1 °C	1,097	1,869
	Substantial changes in **temper-ature variability** across the species' range **(P)**	Highest 35%: ≥ 1.0 °C	2,668	Highest 25%: Absolute difference between (**average absolute deviation in temperatures** across the species' range for all months) from 1975 to 2050 ≥ 1.2 °C	1,925	Highest 15%: ≥ 1.6 °C	1,144	1,869
C. Changes in precip-itation	Substantial changes in **mean precip-itation** occur across the species' range **(P)**	Highest 35%: ≥ 0.38	2,738	Highest 25%: Absolute ratio of change in (**mean precipitation** across the species' range for all months) from 1975 to 2050 ≥ 0.49	1,998	Highest 15%: ≥ 0.61	1,233	1,869
	Substantial changes in **precip-itation variability** across the species' range **(P)**	Highest 35%: ≥ 0.23	2,959	Highest 25%: Absolute ratio of change in (**average absolute deviation in precipitation** across the species' range for all months) from 1975 to 2050 ≥ 0.33	2,152	Highest 15%: ≥ 0.52	1,351	1,869

Table S17: Traits rendering <u>amphibian</u> species as of 'high' climate change vulnerability, and the number of species qualifying under these categories and as unknown, according to three trait threshold scenarios, namely more lenient thresholds, the original or moderate thresholds (i.e., as used for the results presented in Table 2 and Figure 2) and stricter thresholds. Thresholds for traits indicated with a (P) and highlighted in blue were selected based on arbitrary percentage thresholds (35%, 25% and 15%) while those indicated by an (E) and highlighted in green were selected based on experts' judgements. All results shown are based on an optimistic scenario for 2050 under the A1B emission scenario.

**Trait Group**	**Trait**	**More Lenient Estimate**		**Original Estimate**		**Stricter Estimate**		**Un-known**
		Threshold	No. spp.	Threshold	No. spp.	Threshold	No. spp.	No. spp.
**Sensitivity**								
**A. Special-ised habitat and/or micro-habitat require-ments**	Habitat specialist	NA	1,509	Occurs in only 1 habitat	1,509	NA	1,509	156
	Dependence on a particular micro-habitat **(E)**	Larval development and freshwater dependent	3,948	Larval development and freshwater dependent and occurs exclusively in an unbuffered habitat (i.e. not in forest)	955	NA	955	156
**B. Narrow environ-mental tolerances or thres-holds**	Narrow temperature tolerance **(P)**	Lowest 35%: ≤1.50 °C	2,129	Lowest 25%: **Average absolute deviation** in temperature across the species' historical range ≤1.20 °C	1,520	Lowest 15%: ≤0.86 °C	911	128
	Narrow precipitation tolerance **(P)**	Lowest 35%: ≤ 56.00 mm	2,127	Lowest 25%: **Average absolute deviation** in precipitation across the species' historical range ≤ 45.84 mm	1,519	Lowest 15%: ≤ 34.05 mm	911	128
**C. Depend-ence on a specific environ-mental trigger**	Dependence on a specific environmental trigger that's likely to be disrupted by climate change	NA	316	Explosive breeder on rainfall or increased water availability cue (not in forest)	316	NA	316	1,775

**D. Dependence on inter-specific inter-actions**	Increasing negative interactions with other species **(E)**	(Chytridio-mycosis related decline recorded) or (vulnerable to enigmatic decline) or (likely future infection (in a genus with a recorded infection)	2,719	(Chytridiomycosis related decline recorded) or (vulnerable to enigmatic decline) or (likely future infection (in a genus with a recorded infection and is freshwater dependent and in subtropical or tropical (forest, shrubland or grassland habitats))	1,307	(Chytrid-iomycosis related decline recorded) or (vulnerable to enigmatic decline)	595	0
**Low adaptive capacity**								
**A. Poor dispers-ability**	Low intrinsic dispersal capacity **(E)**	Has not become established outside its natural range, and not associated with flowing water, and range size ≤ 8,000 km2	1,768	Has not become established outside its natural range, and not associated with flowing water, and range size ≤ 4,000 km2	1,569	Has not become established outside its natural range, and not associated with flowing water, and range size ≤ 2,000 km2	1,382	113
	Extrinsic barriers to dispersal	NA	745	Occurs exclusively on mountaintops, small islands, polar edges of land masses and/or polar edges of suitable natural habitat	745	NA	745	1,559
**B. Poor evolva-bility**	Low reproductive capacity **(E)**	Annual reproductive output ≤ 100 or viviparous	2,081	Annual reproductive output <= 50 or viviparous	2,073	Annual reproductive output ≤ 25 or viviparous	62	3,232
**Exposure**								
**A. Sea level rise**	Habitat types exposed to sea level inundation **(E)**	Occurs in inundation exposed coastal habitats and up to 2 other habitat types	5	Occurs largely in inundation exposed coastal habitats and up to 1 other habitat type	4	Occurs in inundation exposed coastal habitats only	2	156

**B. Changes in temper-ature**	Substantial changes in **mean temperature** occur across the species' range **(P)**	Highest 35%: >= 2.4 °C	2,121	Highest 25%: Absolute difference between (**mean temperatures** across the species' range for all months) from 1975-2050 ≥ 3.0 °C	1,515	Highest 15%: >= 4.1 °C	908	145
	Substantial changes in **temperature variability** across the species' range **(P)**	Highest 35%: >= 1.5 °C	2,121	Highest 25%: Absolute difference between (**average absolute deviation in temperature** across the species' range for all months) from 1975 to 2050 ≥ 1.9 °C	1,515	Highest 15%: >= 2.8 °C	908	145
**C. Changes in precip-itation**	Substantial changes in **mean precipitation** occur across the species' range **(P)**	Highest 35%: Absolute ratio of change in (**mean precipitation** across the species' range for all months) from 1975 to 2050 >= 0.5	2,121	Highest 25%: Absolute ratio of change in (**mean precipitation** across the species' range for all months) from 1975 to 2050 ≥ 0.6	1,515	Highest 15%: Absolute ratio of change in (**mean precipitation** across the species' range for all months) from 1975 to 2050 >= 0.8	908	145
	Substantial changes in **precipitation variability** across the species' range **(P)**	Highest 35%: >= 0.5	2,121	Highest 25%: Absolute ratio of change in (**average absolute deviation in precipitation** across the species' range for all months) from 1975 to 2050 ≥ 0.7	1,515	Highest 15%: >= 1.0	908	145

Table S18: Traits rendering coral species as of 'high' climate change vulnerability, and the number of species qualifying under these categories and as unknown, according to three trait threshold scenarios, namely more lenient thresholds, the original or moderate thresholds (i.e., as used for the results presented in Table 2 and Figure 2) and stricter thresholds. Thresholds for traits indicated with a (P) and highlighted in blue were selected based on arbitrary percentage thresholds (35%, 25% and 15%) while those indicated by an (E) and highlighted in green were selected based on experts' judgements. All results shown are based on an optimistic scenario for 2050 under the A1B emission scenario.

Trait Group		More Lenient Estimate		Original Estimate		Stricter Estimate		Un-known
	**Trait**	Threshold	No. spp.	Threshold	No. spp.	Threshold	No. spp.	No. spp.
**Sensitivity**								
**A. Special-ised habitat and/or micro-habitat**	Habitat specialist **(P)**	Occurs in <=16 habitats	270	Occurs in <=13 habitats	192	Occurs in <=10 habitats	113	0
	Microhabitat dependence **(P)**	Depth range <= 16m	261	Depth range <= 14m	192	Depth range <= 11m	108	35
**B. Narrow environ-mental tolerances or thresholds**	Narrow temperature tolerance - larvae	NA	137	Broadcast spawning and/or brooding are the only known method(s) of reproduction	137	NA	137	2
	Evidence of exceedance of tolerance - adults	NA	322	Evidence of past high temperature mortality of > 30% of local population on a reef or reef tract	322	NA	322	0
	Lower buffering from depth **(P)**	Maximum depth < 21m	393	Maximum depth < 20m	188	Maximum depth < 15 m	76	31
**D. Depend-ence on inter-specific inter-actions**	Disruption of symbioses with Zooxan-thellae algae **(E)**	NA	738	Obligate Zooxanthellae interaction AND {(not known to have clades D, C1 or C15) **OR** (known to have D, C1 or C15 but not known to 'shuffle' Zooxanthellae)}	738	Obligate Zooxanthellae interaction AND {(not known to have clades D, C1 or C15) **AND** (known to have D, C1 or C15 but not known to 'shuffle' Zooxanthellae)}	585	1

**E. Rarity**	Rarity	NA	196	Rare (geographically restricted or sparsely distributed)	196	NA	196	6
**Low adaptive capacity**								
**A. Poor dispersa-bility**	Low intrinsic dispersal capacity **(E)**	Maximum time to settlement of larvae ≤ 30 days	350	Maximum time to settlement of larvae ≤ 14 days	72	NA (no smaller data classes available)	72	204
	Extrinsic barriers to dispersal	NA	117	Dispersal likely to be retarded by currents and/or temperature	117	NA	117	11
**B. Poor evolva-bility**	Slow turnover of generations **(P)**	Typical colony longevity ≥ 10 years	773	Typical colony longevity ≥ 50 years	13	NA (no larger data classes available)	13	13
	Low growth rate **(P)**	Typical maximum growth rate ≤ 100 mm year	620	Typical maximum growth rate ≤ 30 mm per year	293	Typical maximum growth rate ≤ 10 mm per year	91	9
**Exposure**								
**A. Temper-ature change**	Exposure to temperatures known to cause bleaching **(P)**	**Highest 35%:** ≥ 8.35	250	**Highest 25%:** Mean probability of severe bleaching across species' range (/10years) ≥ 8.48	184	**Highest 15%:** ≥ 8.59	105	95
**B. Elevated CO_2**	Exposure to low aragonite saturation states **(P)**	**Highest 35%:** ≥ 93.32%	247	**Highest 25%:** Proportion of species' range with aragonite saturation ≤ 3 by 2050 ≥ 95.29%	177	**Highest 15%:** >=96.75%	107	91

Table S19: Summary of the potential impacts of sources of uncertainty on numbers of climate change vulnerable bird species. These include scenarios of impacts of missing data (unknowns), the choice of percentage thresholds, the selection of thresholds by experts, the greenhouse gas emission scenario applied and the time frames considered. Percentages represent the numbers of vulnerable species relative to the total number of species. Emissions scenarios and time frame results presented are for terrestrial regions only. Except where specified, assessments are based on optimistic unknowns scenario under emissions scenario A1B for 2050.

		Low/Lenient	%	Mid	%	High/Strict	%
Numbers of vulnerable species *(% of total species)*	Unknowns (pessimistic - optimistic)	4,342	***44***	2,285	***23***	-	**-**
	Percent thresholds (35%-25%-15%)	3,302	***34***	2,285	***23***	1,071	***11***
	Expert thresholds	2,410	***24***	2,285	***23***	2,191	***22***
	Emissions Scenarios (B1-A1B-A2)	1,525	***15***	1,945	***20***	1,600	***16***
	Time frames (2090-2050)	2,686	***27***	1,945	***20***	-	**-**

Table S20: Summary of the potential impacts of sources of uncertainty on numbers of climate change vulnerable amphibian species. These include scenarios of impacts of missing data (unknowns), the choice of percentage thresholds, the selection of thresholds by experts, the greenhouse gas emission scenario applied and the time frames considered. Percentages represent the numbers of vulnerable species relative to the total number of species. Emissions scenarios and time frame results presented are for terrestrial regions only. Except where specified, assessments are based on optimistic unknowns scenario under emissions scenario A1B for 2050.

		Low/Lenient	%	Mid	%	High/Strict	%
Numbers of vulnerable species *(% of total species)*	Unknowns (pessimistic - optimistic)	2,740	***44***	1,368	***22***	-	-
	Percent thresholds (35%-25%-15%)	1,898	***31***	1,368	***22***	787	***13***
	Expert thresholds	1,790	***29***	1,368	***22***	824	***13***
	Emissions Scenarios (B1-A1B-A2)	1,209	***19***	1,300	***21***	1,249	***20***
	Time frames (2090-2050)	1,625	***26***	1,300	***21***	-	-

Table S21: Summary of the potential impacts of sources of uncertainty on numbers of climate change vulnerable coral species. These include scenarios of impacts of missing data (unknowns), the choice of percentage thresholds, the selection of thresholds by experts, the greenhouse gas emission scenario applied and the time frames considered. Percentages represent the numbers of vulnerable species relative to the total number of species. Emissions scenarios and time frame results presented are for terrestrial regions only. Except where specified, assessments are based on optimistic unknowns scenario under emissions scenario A1B for 2050.

		Low/Lenient	%	Mid	%	High/Strict	%
Numbers of vulnerable species *(% of total species)*	Unknowns (pessimistic - optimistic)	2,285	***23***	121	***15***	-	-
	Percent thresholds (35%-25%-15%)	354	***44***	121	***15***	59	***7***
	Expert thresholds	145	***18***	121	***15***	121	***15***
	Emissions Scenarios (B1-A1B-A2)	12	***2***	121	***15***	69	***9***
	Time frames (2090-2050)	341	***43***	121	***15***	-	-

Supporting Information References

1. Turner BL, Kasperson RE, Matson P a, McCarthy JJ, Corell RW, et al. (2003) A framework for vulnerability analysis in sustainability science. Proceedings of the National Academy of Sciences of the United States of America 100: 8074–8079. Available: http://www.pubmedcentral.nih.gov/articlerender.fcgi?artid=166184&tool=pmcentrez&rendertype=abstract.

2. Williams SE, Shoo LP, Isaac JL, Hoffmann AA, Langham G (2008) Towards an integrated framework for assessing the vulnerability of species to climate change. PLoS Biology 6: 2621–2626. Available: http://www.pubmedcentral.nih.gov/articlerender.fcgi?artid=2605927&tool=pmcentrez&rendertype=abstract.

3. Chin A, Kyne PM, Walker TI, McAuley RB (2010) An integrated risk assessment for climate change: analysing the vulnerability of sharks and rays on Australia's Great Barrier Reef. Global Change Biology 16: 1936–1953. Available: http://doi.wiley.com/10.1111/j.1365-2486.2009.02128.x. Accessed 17 January 2011.

4. Graham NAJ, Chabanet P, Evans RD, Jennings S, Letourneur Y, et al. (2011) Extinction vulnerability of coral reef fishes. Ecology Letters 14: 341–348. Available: http://doi.wiley.com/10.1111/j.1461-0248.2011.01592.x. Accessed 15 February 2011.

5. Thomas CD, Hill JK, Anderson BJ, Bailey S, Beale CM, et al. (2010) A framework for assessing threats and benefits to species responding to climate change. Methods in Ecology and Evolution 2: 125–142. Available: http://doi.wiley.com/10.1111/j.2041-210X.2010.00065.x. Accessed 5 October 2010.

6. Cardillo M, Mace GM, Gittleman JL, Jones KE, Bielby J, et al. (2008) The predictability of extinction: biological and external correlates of decline in mammals. Proceedings Biological Sciences / The Royal Society 275: 1441–1448. Available: http://www.ncbi.nlm.nih.gov/pubmed/18367443.

7. Cardillo M, Mace GM, Gittleman JL, Purvis A (2006) Latent extinction risk and the future battlegrounds of mammal conservation. Proceedings of the National Academy of Sciences of the United States of America 103: 4157–4161. Available: http://www.pubmedcentral.nih.gov/articlerender.fcgi?artid=1449663&tool=pmcentrez&rendertype=abstract.

8. Owens IP, Bennett PM (2000) Ecological basis of extinction risk in birds: habitat loss versus human persecution and introduced predators. Proceedings of the National Academy of Sciences of the United States of America 97: 12144–12148. Available: http://www.pubmedcentral.nih.gov/articlerender.fcgi?artid=17308&tool=pmcentrez&rendertype=abstract.

9. Purvis A, Gittleman JL, Cowlishaw G, Mace GM (2000) Predicting extinction risk in declining species. Proceedings Biological Sciences / The Royal Society 267: 1947–1952. Available: http://www.ncbi.nlm.nih.gov/pubmed/11075706.

10. Johnson CN (2002) Determinants of loss of mammal species during the Late Quaternary "megafauna" extinctions: life history and ecology, but not body size. Proceedings Biological Sciences / The Royal Society 269: 2221–2227. Available: http://www.ncbi.nlm.nih.gov/pubmed/12427315.

11. Foden W, Midgley GF, Hughes G, Bond WJ, Thuiller W, et al. (2007) A changing climate is eroding the geographical range of the Namib Desert tree Aloe through population declines and dispersal lags. Diversity and Distributions 13: 645–653. Available: http://blackwell-synergy.com/doi/abs/10.1111/j.1472-4642.2007.00391.x.

12. Jiguet F, Gadot A-S, Julliard R, Newson SE, Couvet D (2007) Climate envelope, life history traits and the resilience of birds facing global change. Global Change Biology 13: 1672–1684. Available: http://www.blackwell-synergy.com/doi/abs/10.1111/j.1365-2486.2007.01386.x.

13. Both C, Bouwhuis S, Lessells CM, Visser ME (2006) Climate change and population declines in a long-distance migratory bird. Nature 441: 81–83. Available: http://www.ncbi.nlm.nih.gov/pubmed/16672969. Accessed 13 July 2010.

14. Foden W, Mace G, Vié J, Angulo A, Butchart S, et al. (2008) Species susceptibility to climate change impacts. In: Vié J-C, Hilton-Taylor C, Stuart SN, editors. Wildlife in a changing world. Barcelona, Spain: Lynx. pp. 77–88.

15. Cardillo M, Mace GM, Jones KE, Bielby J, Bininda-Emonds ORP, et al. (2005) Multiple causes of high extinction risk in large mammal species. Science 309: 1239–1241. Available: http://www.ncbi.nlm.nih.gov/pubmed/16037416. Accessed 5 January 2011.

16. Deutsch CA, Tewksbury JJ, Huey RB, Sheldon KS, Ghalambor CK, et al. (2008) Impacts of climate warming on terrestrial ectotherms across latitude. Proceedings of the National Academy of Sciences of the United States of America 105: 6668–6672.

17. Raven J, Caldeira K, Elderfiled H, Liss P, Riebesell U, et al. (2005) Ocean acidification due to increasing atmospheric carbon dioxide. London.

18. Chevin L-M, Lande R, Mace GM (2010) Adaptation, plasticity, and extinction in a changing environment: towards a predictive theory. PLoS Biology 8: e1000357. Available: http://dx.plos.org/10.1371/journal.pbio.1000357.

19. Bradshaw WE, Holzapfel CM (2006) Evolutionary response to rapid climate change. Science 312: 1477–1478. Available: http://www.ncbi.nlm.nih.gov/pubmed/16763134.

20. Carpenter KE, Abrar M, Aeby G, Aronson RB, Banks S, et al. (2008) One-third of reef-building corals face elevated extinction risk from climate change and local impacts. Science 321: 560–563. Available: http://www.ncbi.nlm.nih.gov/pubmed/18653892. Accessed 27 July 2010.

21. Hoffmann M, Brooks TM, Butchart SHM, Carpenter KE, Chanson J, et al. (2010) The impact of conservation on the status of the world's vertebrates. Science 330: 1503–1509. doi:10.1126/science.1194442.

22. Beresford AE, Buchanan GM, Donald PF, Butchart SHM, Fishpool LDC, et al. (2011) Poor overlap between the distribution of protected areas and globally threatened birds in Africa. Animal Conservation 14: 99–107. Available: http://doi.wiley.com/10.1111/j.1469-1795.2010.00398.x. Accessed 4 March 2012.

23. Buchanan GM, Butchart SHM, Dutson G, Pilgrim JD, Steininger MK, et al. (2008) Using remote sensing to inform conservation status assessment: Estimates of recent deforestation rates on New Britain and the impacts upon endemic birds. Biological Conservation 141: 56–66. Available: http://linkinghub.elsevier.com/retrieve/pii/S0006320707003564. Accessed 16 March 2012.

24. Saunders DA, Smith GT, Rowley I, Clayton C, Helena R (1982) The Availability and Dimensions of Tree Hollows that Provide Nest Sites for Cockatoos (Psittaciformes) in Western Australia. Australian Wildlife Research.

25. Newton I (1994) The role of nest sites in limiting the numbers of hole-nesting birds: a review. Biological Conservation 70: 265–276. Available: http://linkinghub.elsevier.com/retrieve/pii/0006320794901724.

26. Monterrubio-Rico TC, Escalante-Pliego P (2006) Richness, distribution and conservation status of cavity nesting birds in Mexico. Biological Conservation 128: 67–78. Available: http://linkinghub.elsevier.com/retrieve/pii/S0006320705003782.

27. Cockle KL, Martin K, Drever MC (2010) Supply of tree-holes limits nest density of cavity-nesting birds in primary and logged subtropical Atlantic forest. Biological Conservation 143: 2851–2857. Available: http://linkinghub.elsevier.com/retrieve/pii/S0006320710003423.

28. Hijmans RJ, Cameron SE, Parra JL, Jones PG, Jarvis A (2005) Very high resolution interpolated climate surfaces for global land areas. International Journal of Climatology 25: 1965–1978. Available: http://doi.wiley.com/10.1002/joc.1276. Accessed 20 July 2010.

29. Staff M-H (2002) McGraw-Hill Dictionary of Scientific and Technical Terms. 6th ed. Parker S, editor New York.

30. Tabor K, Williams JW (2010) Globally downscaled climate projections for assessing the conservation impacts of climate change. Ecological Applications 20: 554–565. Available: http://www.ncbi.nlm.nih.gov/pubmed/20405806.

31. Nakicenovic N, Alcamo J, Davis G, Vries B de, Fenhann J, et al. (2001) An overview of scenarios. IPCC Special Report on Emissions Scenarios. Norway: GRID-Arendal.

32. Malcolm JR, Markham A, Neilson RP, Garaci M (2002) Estimated migration rates under scenarios of global climate change. Journal of Biogeography 29: 835–849. Available: http://doi.wiley.com/10.1046/j.1365-2699.2002.00702.x.

33. IUCN. (2010) Guidelines for Using the IUCN Red List Categories and Criteria Version 8.1. Available: http://intranet.iucn.org/webfiles/doc/SSC/RedList/RedListGuidelines.pdf.

34. Stuart SN, Chanson JS, Cox N a, Young BE, Rodrigues ASL, et al. (2004) Status and trends of amphibian declines and extinctions worldwide. Science 306: 1783–1786. Available: http://www.ncbi.nlm.nih.gov/pubmed/15486254.

35. Lips KR, Brem F, Brenes R, Reeve JD, Alford R a, et al. (2006) Emerging infectious disease and the loss of biodiversity in a Neotropical amphibian community. Proceedings of the National Academy of Sciences of the United States of America 103: 3165–3170. Available: http://www.pubmedcentral.nih.gov/articlerender.fcgi?artid=1413869&tool=pmcentrez&rendertype=abstract.

36. Rohr JR, Raffel TR (2010) Linking global climate and temperature variability to widespread amphibian declines putatively caused by disease. Proceedings of the National Academy of Sciences of the United States of America 107. Available: http://www.ncbi.nlm.nih.gov/pubmed/20404180.

37. Fisher MC (2007) Potential interactions between amphibian immunity, infectious disease and climate change. Animal Conservation 10: 420–421. Available: http://doi.wiley.com/10.1111/j.1469-1795.2007.00148.x. Accessed 5 April 2011.

38. Bosch J, Rincón P a. (2008) Chytridiomycosis-mediated expansion of Bufo bufo in a montane area of Central Spain: an indirect effect of the disease. Diversity and Distributions 14: 637–643. Available: http://doi.wiley.com/10.1111/j.1472-4642.2007.00461.x. Accessed 23 March 2011.

39. Pounds JA, Bustamante MR, Coloma L a, Consuegra J a, Fogden MPL, et al. (2006) Widespread amphibian extinctions from epidemic disease driven by global warming. Nature 439: 161–167. Available: http://www.ncbi.nlm.nih.gov/pubmed/16407945. Accessed 22 July 2010.

40. Raffel TR, Rohr JR, Kiesecker JM, Hudson PJ (2006) Negative effects of changing temperature on amphibian immunity under field conditions. Functional Ecology 20: 819–828. Available: http://doi.wiley.com/10.1111/j.1365-2435.2006.01159.x. Accessed 21 February 2011.

41. Lips KR, Diffendorfer J, Mendelson JRI, Sears MW (2008) Riding the Wave : Reconciling the Roles of Disease and Climate Change in Amphibian Declines. PLoS Biology 6. doi:10.1371/journal.pbio.0060072.

42. Parmesan C, Singer MC (2008) Amphibian extinctions: Disease not the whole story. PLoS biology. doi:10.1371/journal.pbio.0060072.

43. Seimon TA, Seimon A, Daszak P, Halloy SRP, Schloegel LM, et al. (2007) Upward range extension of Andean anurans and chytridiomycosis to extreme elevations in response to tropical deglaciation. Global Change Biology 13: 288–299. Available: http://www.blackwell-synergy.com/doi/abs/10.1111/j.1365-2486.2006.01278.x.

44. LaMarca YE (2006) A chytridiomycosis epidemic and a severe dry season precede the disappearance of Atelopis species from the Venezuelan Andes. Copeia 16: 395–402.

45. Veron JEN (2000) Corals of the World. Townsville, Australia: Australian Institute of Marine Science.

46. Bhagooli R, Hidaka M (2004) Photoinhibition, bleaching susceptibility and mortality in two scleractinian corals, Platygyra ryukyuensis and Stylophora pistillata, in response to thermal and light stresses. Comparative biochemistry and physiology Part A, Molecular & integrative physiology 137: 547–555. Available: http://www.ncbi.nlm.nih.gov/pubmed/15123191. Accessed 26 January 2011.

47. Tchernov D, Gorbunov MY, De Vargas C, Narayan Yadav S, Milligan AJ, et al. (2004) Membrane lipids of symbiotic algae are diagnostic of sensitivity to thermal bleaching in corals. Proceedings of the National Academy of Sciences of the United States of America 101: 13531–13535. Available: http://www.pubmedcentral.nih.gov/articlerender.fcgi?artid=518791&tool=pmcentrez&rendertype=abstract.

48. Hoogenboom M, Anthony K, Connolly S (2006) Energetic cost of photoinhibition in corals. Marine Ecology Progress Series 313: 1–12. Available: http://www.int-res.com/abstracts/meps/v313/p1-12/.

49. Loram JE, Boonham N, O'Toole P, Trapido-Rosenthal HG, Douglas AE (2007) Molecular quantification of symbiotic dinoflagellate algae of the genus Symbiodinium. The Biological Bulletin 212: 259–268. Available: http://www.ncbi.nlm.nih.gov/pubmed/17565115.

50. Little AF, Van Oppen MJH, Willis BL (2004) Flexibility in algal endosymbioses shapes growth in reef corals. Science 304: 1492–1494. Available: http://www.ncbi.nlm.nih.gov/pubmed/15178799.

51. Mieog JC, Oppen MJH, Cantin NE, Stam WT, Olsen JL (2007) Real-time PCR reveals a high incidence of Symbiodinium clade D at low levels in four scleractinian corals across the Great Barrier Reef: implications for symbiont shuffling. Coral Reefs 26: 449–457. Available: http://www.springerlink.com/index/10.1007/s00338-007-0244-8.

52. Jones AM, Berkelmans R, Van Oppen MJH, Mieog JC, Sinclair W (2008) A community change in the algal endosymbionts of a scleractinian coral following a natural bleaching event: field evidence of acclimatization. Proceedings Biological Sciences / The Royal Society 275: 1359–1365. Available: http://www.ncbi.nlm.nih.gov/pubmed/18348962.

53. Veron JEN, Hoegh-Guldberg O, Lenton TM, Lough JM, Obura DO, et al. (2009) The coral reef crisis: the critical importance of<350 ppm CO2. Marine Pollution Bulletin 58: 1428–1436. Available: http://www.ncbi.nlm.nih.gov/pubmed/19782832.

54. DeVantier L, De'Ath G, Done T, Turak E (1998) Ecological Assessment of a Complex Natural System: a Case Study from the Great Barrier Reef. Ecological Applications 8: 480–496.

55. DeVantier L, Turak E, Al-shaikh K, De G (2000) Coral communities of the central-northern Saudi Arabian Red Sea. Fauna of Arabia 18: 23–66.

56. DeVantier L, De'Ath G, Klaus R, Al-moghrabi S, Abdulaziz M, et al. (2004) Reef-building corals and coral communities of the Socotra Archipelago , a zoogeographic "crossroads" in the Arabian Sea. Fauna of Arabia 20: 117–168.

57. DeVantier L, De'ath G, Turak E, Done T, Fabricius K (2006) Species richness and community structure of reef-building corals on the nearshore Great Barrier Reef. Coral Reefs 25: 329–340. doi:10.1007/s00338-006-0115-8.

58. Turak E, Brodie J, DeVantier L (2007) Reef-building corals and coral communities of the Yemen Red Sea. Fauna of Arabia 23: 1–40.

59. Donner SD (2009) Coping with commitment: projected thermal stress on coral reefs under different future scenarios. PLOS ONE 4: e5712. Available: http://www.ncbi.nlm.nih.gov/pubmed/19492060.

60. Delworth TL, Broccoli AJ, Rosati A, Stouffer RJ, Balaji V, et al. (2006) GFDL's CM2 Global Coupled Climate Models. Part I: Formulation and Simulation Characteristics. Journal of Climate 19: 643–674. Available: http://journals.ametsoc.org/doi/abs/10.1175/JCLI3629.1.

61. Cao L, Caldeira K (2008) Atmospheric CO2 stabilization and ocean acidification. Geophysical Research Letters 35: 1–5. Available: http://www.agu.org/pubs/crossref/2008/2008GL035072.shtml. Accessed 9 March 2011.

62. Hoegh-Guldberg O, Mumby PJ, Hooten a J, Steneck RS, Greenfield P, et al. (2007) Coral reefs under rapid climate change and ocean acidification. Science 318: 1737–1742. Available: http://www.ncbi.nlm.nih.gov/pubmed/18079392.

63. Guinotte JM, Buddemeier RW, Kleypas JA (2003) Future coral reef habitat marginality: temporal and spatial effects of climate change in the Pacific basin. Coral Reefs 22: 551–558. Available: http://www.springerlink.com/openurl.asp?genre=article&id=doi:10.1007/s00338-003-0331-4.

64. Sheppard C, Price A, Roberts C (1992) Ecosystem Responses to Extreme Natural Stresses. Marine Ecology of the Arabian Region: Patterns and Processes in Extreme Tropical Environments. San Diego, CA: Academic Press. pp. 242–260.

65. Keith SA, Herbert RJH, Norton PA, Hawkins SJ, Newton AC (2010) Individualistic species limitations of climate-induced range expansions generated by meso-scale dispersal barriers. Diversity and Distributions 17: 275–286. Available: http://doi.wiley.com/10.1111/j.1472-4642.2010.00734.x. Accessed 29 December 2010.

66. Harrison PL (2011) Sexual reproduction of scleractinian corals. In: Stambler ZD and N, editor. Coral Reefs: An Ecosystem in Transition Part 3. Springer Publishers. pp. 59–85. doi:10.1007/978-94-007-0114-4_6.

67. Williams PH, Gaston KJ (1998) Biodiversity indicators: graphical techniques, smoothing and searching for what makes relationships work. Ecography 21: 551–560. Available: http://doi.wiley.com/10.1111/j.1600-0587.1998.tb00447.x.

68. Lamoreux J, Akcakaya H., Bennun L, Al E (2003) Value of the IUCN Red List. Trends in Ecology & Evolution 18: 214–215. Available: http://linkinghub.elsevier.com/retrieve/pii/S0169534703000740. Accessed 21 July 2010.

69. De Grammont PC, Cuarón AD (2006) An evaluation of threatened species categorization systems used on the American continent. Conservation Biology 20: 14–27. Available: http://doi.wiley.com/10.1111/j.1523-1739.2006.00352.x. Accessed 4 January 2011.

70. Hoffmann M, Brooks T, Da Fonseca G, Gascon C, Hawkins A, et al. (2008) Conservation planning and the IUCN Red List. Endangered Species Research 6: 113–125. Available: http://www.int-res.com/abstracts/esr/v6/n2/p113-125/.

71. Nakicenovic N, Swart R (Eds) (2000) Emission scenarios: a special report of Working Group III of the Intergovernmental Panel on Climate Change. Cambridge, UK: Cambridge University Press.

72. Meehl GA, Stocker TF, Collins WD, P. F, Gaye AT, et al. (2007) Global Climate Projections. In: Solomon S, Qin D, Manning M, Chen Z, Marquis M, et al., editors. Climate Change 2007: The Physical Science Basis. Contribution of Working Group I to the Fourth Assessment Report of the Intergovernmental Panel on Climate Change. Cambridge, UK: Cambridge University Press. pp. 747–845.

73. Parmesan C, Yohe G (2003) A globally coherent fingerprint of climate change impacts across natural systems. Nature 421: 37–42. Available: http://www.ncbi.nlm.nih.gov/pubmed/12511946.

74. Root TL, Price JT, Hall KR, Schneider SH (2003) Fingerprints of global warming on wild animals and plants. Nature 421: 57–60. doi:10.1038/nature01309.1.

75. Chen I, Hill JK, Ohlemüller R, Roy DB, Thomas CD (2011) Rapid Range Shifts of Species of Climate Warming. Science 333: 1024–1026.

76. Huntley B, Collingham YC, Willis SG, Green RE (2008) Potential impacts of climatic change on European breeding birds. PloS one 3: e1439. Available: http://www.ncbi.nlm.nih.gov/pubmed/18197250.

77. Araujo MB, Thuiller W, Pearson RG (2006) Climate warming and the decline of amphibians and reptiles in Europe. Journal of Biogeography 33: 1712–1728. Available: http://doi.wiley.com/10.1111/j.1365-2699.2006.01482.x. Accessed 10 June 2011.

78. Williams JW, Jackson ST, Kutzbach JE (2007) Projected distributions of novel and disappearing climates by 2100 AD. Proceedings of the National Academy of Sciences of the United States of America 104: 5738–5742. Available: http://www.pubmedcentral.nih.gov/articlerender.fcgi?artid=1851561&tool=pmcentrez&rendertype=abstract.

79. Ohlemuller R, Gritti ES, Sykes MT, Thomas CD (2006) Quantifying components of risk for European woody species under climate change. Global Change Biology 12: 1788–1799. Available: http://doi.wiley.com/10.1111/j.1365-2486.2006.01231.x. Accessed 8 August 2011.

80. Tropical Ecology Assessment and Monitoring Network (n.d.). Available: http://www.teamnetwork.org/en/.

81. Global Observation Research Initiative in Alpine Environments (n.d.). Available: http://www.gloria.ac.at/.

82. NOAA’s Coral Health and Monitoring Program (n.d.). Available: www.coral.noaa.gov/.

83. Burgman M, Carr A, Godden L, Gregory R, McBride M, et al. (2011) Redefining expertise and improving ecological judgment. Conservation Letters 4: 81–87. Available: http://doi.wiley.com/10.1111/j.1755-263X.2011.00165.x. Accessed 13 July 2011.

84. Burgman M a., McBride M, Ashton R, Speirs-Bridge A, Flander L, et al. (2011) Expert Status and Performance. PLoS ONE 6: e22998. Available: http://dx.plos.org/10.1371/journal.pone.0022998. Accessed 30 July 2011.

85. Ozgul A, Childs DZ, Oli MK, Armitage KB, Blumstein DT, et al. (2010) Coupled dynamics of body mass and population growth in response to environmental change. Nature 466: 482–485. Available: http://www.nature.com/doifinder/10.1038/nature09210. Accessed 22 July 2010.

86. Van Bocxlaer I, Loader SP, Roelants K, Biju SD, Menegon M, et al. (2010) Gradual adaptation toward a range-expansion phenotype initiated the global radiation of toads. Science 327: 679–682. Available: http://www.ncbi.nlm.nih.gov/pubmed/20133569.

87. Franks SJ, Sim S, Weis AE (2007) Rapid evolution of flowering time by an annual plant in response to a climate fluctuation. Proceedings of the National Academy of Sciences of the United States of America 104: 1278–1282. Available: http://www.pubmedcentral.nih.gov/articlerender.fcgi?artid=1783115&tool=pmcentrez&rendertype=abstract.

88. Keith DA, Akçakaya HR, Thuiller W, Midgley GF, Pearson RG, et al. (2008) Predicting extinction risks under climate change: coupling stochastic population models with dynamic bioclimatic habitat models. Biology Letters 4: 560–563. Available: http://www.pubmedcentral.nih.gov/articlerender.fcgi?artid=2610061&tool=pmcentrez&rendertype=abstract. Accessed 16 July 2010.

89. Morin X, Viner D, Chuine I (2008) Tree species range shifts at a continental scale : new predictive insights from a process-based model. Journal of Ecology 96: 784–794. doi:10.1111/j.1365-2745.2007.0.

90. Walther G-R, Beißner S, Burga C (2005) Trends in the upward shift of alpine plants. Journal of Vegetation Science 16: 541. Available: http://doi.wiley.com/10.1658/1100-9233(2005)16[541:TITUSO]2.0.CO;2.
