# Supplementary material for: Identifying the World's Most Climate Change Vulnerable Species: A Systematic Trait-Based Assessment of all Birds, Amphibians and Corals
Source: PLoS One. 2013 Jun 12;8(6):e65427. doi: 10.1371/journal.pone.0065427 (PMC3680427; doi:10.1371/journal.pone.0065427)
Supplement: Appendix A — Climate change vulnerability scores for bird species. (PDF) [file pone.0065427.s036.pdf]

Appendix A:
Climate change vulnerability assessments by species for **birds**

*Assessments of overall vulnerability are based on optimistic assumptions for missing trait information.

Species	Common name	SENSI-TIVITY	UN-ADAPTA-BILITY	EXPO-SURE	**OVERALL VULNERA-BILITY**
Abeillia abeillei	Emerald-chinned Hummingbird	H	H	H	**H**
Abroscopus albogularis	Rufous-faced Warbler	H	L	U	**L**
Abroscopus schisticeps	Black-faced Warbler	H	H	U	**L**
Abroscopus superciliaris	Yellow-bellied Warbler	H	L	U	**L**
Aburria aburri	Wattled Guan	H	U	L	**L**
Acanthagenys rufogularis	Spiny-cheeked Honeyeater	H	H	L	**L**
Acanthidops bairdii	Peg-billed Finch	H	H	H	**H**
Acanthisitta chloris	Rifleman	U	L	H	**L**
Acanthiza apicalis	Inland Thornbill	H	L	L	**L**
Acanthiza chrysorrhoa	Yellow-rumped Thornbill	H	L	L	**L**
Acanthiza ewingii	Tasmanian Thornbill	H	L	H	**L**
Acanthiza inornata	Western Thornbill	H	H	U	**L**
Acanthiza iredalei	Slender-billed Thornbill	U	L	U	**L**
Acanthiza katherina	Mountain Thornbill	H	H	L	**L**
Acanthiza lineata	Striated Thornbill	H	H	H	**H**
Acanthiza murina	Papuan Thornbill	H	H	H	**H**
Acanthiza nana	Yellow Thornbill	U	L	U	**L**
Acanthiza pusilla	Brown Thornbill	H	H	L	**L**
Acanthiza reguloides	Buff-rumped Thornbill	U	L	U	**L**
Acanthiza robustirostris	Slaty-backed Thornbill	U	L	U	**L**
Acanthiza uropygialis	Chestnut-rumped Thornbill	U	L	U	**L**
Acanthorhynchus superciliosus	Western Spinebill	H	H	H	**H**
Acanthorhynchus tenuirostris	Eastern Spinebill	H	L	L	**L**
Acanthornis magna	Scrubtit	H	L	H	**L**
Accipiter albogularis	Pied Goshawk	H	H	H	**H**
Accipiter badius	Shikra	L	H	L	**L**
Accipiter bicolor	Bicoloured Hawk	L	H	L	**L**
Accipiter brachyurus	New Britain Sparrowhawk	H	H	H	**H**
Accipiter brevipes	Levant Sparrowhawk	H	H	H	**H**
Accipiter butleri	Nicobar Sparrowhawk	H	H	H	**H**
Accipiter castanilius	Chestnut-flanked Sparrowhawk	H	H	H	**H**
Accipiter cirrocephalus	Collared Sparrowhawk	L	H	L	**L**
Accipiter collaris	Semicollared Hawk	H	H	L	**L**

Accipiter cooperii	Cooper's Hawk	H	H	H	**H**
Accipiter erythrauchen	Rufous-necked Sparrowhawk	H	H	H	**H**
Accipiter erythropus	Red-thighed Sparrowhawk	H	H	H	**H**
Accipiter fasciatus	Brown Goshawk	L	H	L	**L**
Accipiter francesiae	Frances's Sparrowhawk	U	H	U	**L**
Accipiter gentilis	Northern Goshawk	H	H	H	**H**
Accipiter griseiceps	Sulawesi Goshawk	H	H	H	**H**
Accipiter gularis	Japanese Sparrowhawk	L	H	H	**L**
Accipiter gundlachi	Gundlach's Hawk	H	H	H	**H**
Accipiter haplochrous	White-bellied Goshawk	H	H	H	**H**
Accipiter henicogrammus	Moluccan Goshawk	H	H	H	**H**
Accipiter henstii	Henst's Goshawk	H	H	L	**L**
Accipiter imitator	Imitator Sparrowhawk	H	H	L	**L**
Accipiter luteoschistaceus	Slaty-mantled Sparrowhawk	H	H	H	**H**
Accipiter madagascariensis	Madagascar Sparrowhawk	L	H	L	**L**
Accipiter melanochlamys	Black-mantled Goshawk	H	H	H	**H**
Accipiter melanoleucus	Black Goshawk	L	H	L	**L**
Accipiter meyerianus	Meyer's Goshawk	H	H	H	**H**
Accipiter minullus	Little Sparrowhawk	L	H	L	**L**
Accipiter nanus	Small Sparrowhawk	H	H	H	**H**
Accipiter nisus	Eurasian Sparrowhawk	H	H	H	**H**
Accipiter novaehollandiae	Grey Goshawk	H	H	L	**L**
Accipiter ovampensis	Ovampo Sparrowhawk	L	H	L	**L**
Accipiter poliocephalus	Grey-headed Goshawk	U	H	U	**L**
Accipiter poliogaster	Gray-bellied Hawk	H	H	H	**H**
Accipiter princeps	New Britain Goshawk	H	H	H	**H**
Accipiter rhodogaster	Vinous-breasted Sparrowhawk	H	H	H	**H**
Accipiter rufitorques	Fiji Goshawk	H	H	L	**L**
Accipiter rufiventris	Rufous-chested Sparrowhawk	H	H	L	**L**
Accipiter soloensis	Chinese Goshawk	L	H	L	**L**
Accipiter striatus	Sharp-shinned Hawk	H	H	L	**L**
Accipiter superciliosus	Tiny Hawk	H	H	H	**H**
Accipiter tachiro	African Goshawk	U	H	L	**L**
Accipiter trinotatus	Spot-tailed Goshawk	H	H	H	**H**
Accipiter trivirgatus	Crested Goshawk	H	H	L	**L**
Accipiter virgatus	Besra	H	H	H	**H**
Aceros cassidix	Knobbed Hornbill	H	H	H	**H**
Aceros comatus	White-crowned Hornbill	H	H	H	**H**
Aceros corrugatus	Wrinkled Hornbill	H	H	H	**H**
Aceros everetti	Sumba Hornbill	H	H	H	**H**
Aceros leucocephalus	Writhed Hornbill	H	H	H	**H**
Aceros narcondami	Narcondam Hornbill	H	H	U	**L**

Aceros nipalensis	Rufous-necked Hornbill	H	H	H	**H**
Aceros plicatus	Papuan Hornbill	H	H	L	**L**
Aceros subruficollis	Plain-pouched Hornbill	H	H	H	**H**
Aceros undulatus	Wreathed Hornbill	H	H	L	**L**
Aceros waldeni	Rufous-headed Hornbill	H	H	H	**H**
Achaetops pycnopygius	Rockrunner	H	L	H	**L**
Acridotheres albocinctus	Collared Myna	U	L	U	**L**
Acridotheres cinereus	Pale-bellied Myna	U	U	U	**L**
Acridotheres cristatellus	Crested Myna	U	L	U	**L**
Acridotheres fuscus	Jungle Myna	U	L	U	**L**
Acridotheres ginginianus	Bank Myna	U	L	U	**L**
Acridotheres grandis	White-vented Myna	U	L	U	**L**
Acridotheres tristis	Common Myna	U	L	L	**L**
Acrobatornis fonsecai	Pink-legged Graveteiro	H	U	H	**L**
Acrocephalus aedon	Thick-billed Warbler	U	U	U	**L**
Acrocephalus aequinoctialis	Kiritimati Reed-warbler	H	H	U	**L**
Acrocephalus agricola	Paddyfield Warbler	H	L	H	**L**
Acrocephalus arundinaceus	Great Reed-warbler	L	L	H	**L**
Acrocephalus atyphus	Tuamotu Reed-warbler	U	H	U	**L**
Acrocephalus australis	Australian Reed-warbler	H	L	L	**L**
Acrocephalus bistrigiceps	Black-browed Reed-warbler	U	U	U	**L**
Acrocephalus brevipennis	Cape Verde Warbler	H	L	H	**L**
Acrocephalus caffer	Tahiti Reed-warbler	H	H	U	**L**
Acrocephalus concinens	Blunt-winged Warbler	U	U	U	**L**
Acrocephalus dumetorum	Blyth's Reed-warbler	H	L	H	**L**
Acrocephalus familiaris	Millerbird	H	H	U	**L**
Acrocephalus gracilirostris	Lesser Swamp-warbler	U	L	U	**L**
Acrocephalus griseldis	Basra Reed-warbler	H	U	L	**L**
Acrocephalus kerearako	Cook Islands Reed-warbler	H	H	U	**L**
Acrocephalus luscinius	Nightingale Reed-warbler	H	H	H	**H**
Acrocephalus melanopogon	Moustached Warbler	H	L	L	**L**
Acrocephalus mendanae	Marquesan Reed-warbler	U	U	U	**L**
Acrocephalus newtoni	Madagascar Swamp-warbler	U	L	U	**L**
Acrocephalus orinus	Large-billed Reed-warbler	H	U	U	**L**
Acrocephalus paludicola	Aquatic Warbler	H	L	H	**L**
Acrocephalus palustris	Marsh Warbler	H	L	H	**L**
Acrocephalus rehsei	Nauru Reed-warbler	H	H	U	**L**
Acrocephalus rimatarae	Rimatara Reed-warbler	H	H	U	**L**
Acrocephalus rodericanus	Rodrigues Warbler	H	H	H	**H**
Acrocephalus rufescens	Greater Swamp-warbler	U	L	U	**L**
Acrocephalus schoenobaenus	Sedge Warbler	H	L	H	**L**

Acrocephalus scirpaceus	Eurasian Reed-warbler	H	L	H	**L**
Acrocephalus sechellensis	Seychelles Warbler	H	H	U	**L**
Acrocephalus sorghophilus	Streaked Reed-warbler	H	U	L	**L**
Acrocephalus stentoreus	Clamorous Reed-warbler	U	L	U	**L**
Acrocephalus syrinx	Caroline Islands Reed-warbler	H	U	H	**L**
Acrocephalus taiti	Henderson Reed-warbler	H	H	U	**L**
Acrocephalus tangorum	Manchurian Reed-warbler	H	U	H	**L**
Acrocephalus vaughani	Pitcairn Reed-warbler	H	H	U	**L**
Acropternis orthonyx	Ocellated Tapaculo	H	H	H	**H**
Acryllium vulturinum	Vulturine Guineafowl	H	L	L	**L**
Actenoides bougainvillei	Moustached Kingfisher	H	H	H	**H**
Actenoides concretus	Rufous-collared Kingfisher	H	H	H	**H**
Actenoides hombroni	Blue-capped Kingfisher	H	H	H	**H**
Actenoides lindsayi	Spotted Kingfisher	H	H	U	**L**
Actenoides monachus	Green-backed Kingfisher	H	H	H	**H**
Actenoides princeps	Scaly Kingfisher	H	H	H	**H**
Actinodura egertoni	Rusty-fronted Barwing	U	L	U	**L**
Actinodura morrisoniana	Taiwan Barwing	H	H	H	**H**
Actinodura nipalensis	Hoary-throated Barwing	H	H	H	**H**
Actinodura ramsayi	Spectacled Barwing	U	H	U	**L**
Actinodura sodangorum	Black-crowned Barwing	H	U	L	**L**
Actinodura souliei	Streaked Barwing	H	H	U	**L**
Actinodura waldeni	Streak-throated Barwing	H	H	H	**H**
Actitis hypoleucos	Common Sandpiper	L	H	H	**L**
Actitis macularius	Spotted Sandpiper	L	L	L	**L**
Actophilornis africanus	African Jacana	L	L	L	**L**
Actophilornis albinucha	Madagascar Jacana	H	L	L	**L**
Adelomyia melanogenys	Speckled Hummingbird	H	H	L	**L**
Aechmophorus clarkii	Clark's Grebe	H	H	H	**H**
Aechmophorus occidentalis	Western Grebe	H	H	H	**H**
Aegithalos caudatus	Long-tailed Tit	H	L	H	**L**
Aegithalos concinnus	Black-throated Tit	U	L	H	**L**
Aegithalos fuliginosus	White-necklaced Tit	U	U	H	**L**
Aegithalos iouschistos	Black-browed Tit	U	L	H	**L**
Aegithalos leucogenys	White-cheeked Tit	H	L	H	**L**
Aegithalos niveogularis	White-throated Tit	U	L	H	**L**
Aegithina lafresnayei	Great Iora	H	H	L	**L**
Aegithina nigrolutea	White-tailed Iora	U	L	U	**L**
Aegithina tiphia	Common Iora	U	L	L	**L**
Aegithina viridissima	Green Iora	H	H	H	**H**
Aegolius acadicus	Northern Saw-whet Owl	H	L	L	**L**
Aegolius funereus	Boreal Owl	H	L	H	**L**

Aegolius harrisii	Buff-fronted Owl	H	L	H	**L**
Aegolius ridgwayi	Southern Saw-whet Owl	H	L	H	**L**
Aegotheles albertisi	Mountain Owlet-nightjar	H	U	H	**L**
Aegotheles archboldi	Archbold's Owlet-nightjar	H	U	H	**L**
Aegotheles bennettii	Barred Owlet-nightjar	H	U	L	**L**
Aegotheles crinifrons	Long-whiskered Owlet-nightjar	H	U	H	**L**
Aegotheles cristatus	Australian Owlet-nightjar	H	L	L	**L**
Aegotheles insignis	Feline Owlet-nightjar	H	U	H	**L**
Aegotheles savesi	New Caledonian Owlet-nightjar	H	H	H	**H**
Aegotheles tatei	Starry Owlet-nightjar	H	U	H	**L**
Aegotheles wallacii	Wallace's Owlet-nightjar	H	U	H	**L**
Aegypius monachus	Cinereous Vulture	H	H	H	**H**
Aenigmatolimnas marginalis	Striped Crake	H	L	L	**L**
Aepypodius arfakianus	Wattled Brush-turkey	H	H	H	**H**
Aepypodius bruijnii	Bruijn's Brush-turkey	H	H	H	**H**
Aeronautes andecolus	Andean Swift	H	U	H	**L**
Aeronautes montivagus	White-tipped Swift	H	U	L	**L**
Aeronautes saxatalis	White-throated Swift	H	L	H	**L**
Aethia cristatella	Crested Auklet	H	H	H	**H**
Aethia psittacula	Parakeet Auklet	H	H	L	**L**
Aethia pusilla	Least Auklet	H	H	H	**H**
Aethia pygmaea	Whiskered Auklet	H	H	H	**H**
Aethopyga bella	Handsome Sunbird	U	U	U	**L**
Aethopyga boltoni	Apo Sunbird	U	H	H	**L**
Aethopyga christinae	Fork-tailed Sunbird	H	L	H	**L**
Aethopyga duyvenbodei	Elegant Sunbird	H	H	H	**H**
Aethopyga eximia	White-flanked Sunbird	U	U	H	**L**
Aethopyga flagrans	Flaming Sunbird	H	U	U	**L**
Aethopyga gouldiae	Gould's Sunbird	U	L	H	**L**
Aethopyga ignicauda	Fire-tailed Sunbird	U	L	U	**L**
Aethopyga linaraborae	Lina's Sunbird	H	H	H	**H**
Aethopyga mystacalis	Scarlet Sunbird	U	U	U	**L**
Aethopyga nipalensis	Green-tailed Sunbird	U	L	U	**L**
Aethopyga primigenia	Grey-hooded Sunbird	H	H	H	**H**
Aethopyga pulcherrima	Metallic-winged Sunbird	U	L	U	**L**
Aethopyga saturata	Black-throated Sunbird	U	H	U	**L**
Aethopyga shelleyi	Lovely Sunbird	U	L	U	**L**
Aethopyga siparaja	Crimson Sunbird	U	H	L	**L**
Afropavo congensis	Congo Peafowl	H	H	L	**L**
Agamia agami	Agami Heron	H	H	H	**H**
Agapornis canus	Grey-headed Lovebird	U	L	L	**L**
Agapornis fischeri	Fischer's Lovebird	L	L	H	**L**

Agapornis lilianae	Lilian's Lovebird	H	L	H	**L**
Agapornis nigrigenis	Black-cheeked Lovebird	H	L	H	**L**
Agapornis personatus	Yellow-collared Lovebird	H	L	L	**L**
Agapornis pullarius	Red-headed Lovebird	H	L	H	**L**
Agapornis roseicollis	Rosy-faced Lovebird	H	L	H	**L**
Agapornis swindernianus	Black-collared Lovebird	H	U	H	**L**
Agapornis taranta	Black-winged Lovebird	U	L	L	**L**
Agelaioides badius	Bay-winged Cowbird	U	L	L	**L**
Agelaius assimilis	Red-shouldered Blackbird	U	H	H	**L**
Agelaius humeralis	Tawny-shouldered Blackbird	U	H	H	**L**
Agelaius phoeniceus	Red-winged Blackbird	H	H	L	**L**
Agelaius tricolor	Tricoloured Blackbird	H	L	H	**L**
Agelaius xanthomus	Yellow-shouldered Blackbird	H	H	H	**H**
Agelastes meleagrides	White-breasted Guineafowl	H	L	L	**L**
Agelastes niger	Black Guineafowl	H	U	H	**L**
Agelasticus cyanopus	Unicoloured Blackbird	H	L	L	**L**
Agelasticus thilius	Yellow-winged Blackbird	H	L	L	**L**
Agelasticus xanthophthalmus	Pale-eyed Blackbird	H	U	H	**L**
Aglaeactis aliciae	Purple-backed Sunbeam	H	H	H	**H**
Aglaeactis castelnaudii	White-tufted Sunbeam	H	U	H	**L**
Aglaeactis cupripennis	Shining Sunbeam	U	H	H	**L**
Aglaeactis pamela	Black-hooded Sunbeam	U	U	H	**L**
Aglaiocercus berlepschi	Venezuelan Sylph	H	U	H	**L**
Aglaiocercus coelestis	Violet-tailed Sylph	U	H	H	**L**
Aglaiocercus kingi	Long-tailed Sylph	U	H	L	**L**
Agriornis albicauda	White-tailed Shrike-tyrant	H	U	H	**L**
Agriornis lividus	Great Shrike-tyrant	U	L	H	**L**
Agriornis micropterus	Grey-bellied Shrike-tyrant	H	L	L	**L**
Agriornis montanus	Black-billed Shrike-tyrant	U	L	U	**L**
Agriornis murinus	Lesser Shrike-tyrant	H	L	L	**L**
Ailuroedus buccoides	White-eared Catbird	H	H	U	**L**
Ailuroedus crassirostris	Green Catbird	H	H	L	**L**
Ailuroedus melanotis	Spotted Catbird	U	H	L	**L**
Aimophila aestivalis	Bachman's Sparrow	H	L	L	**L**
Aimophila botterii	Botteri's Sparrow	L	L	H	**L**
Aimophila carpalis	Rufous-winged Sparrow	H	L	H	**L**
Aimophila cassinii	Cassin's Sparrow	H	L	H	**L**
Aimophila humeralis	Black-chested Sparrow	H	L	H	**L**
Aimophila mystacalis	Bridled Sparrow	H	L	L	**L**
Aimophila notosticta	Oaxaca Sparrow	L	U	L	**L**
Aimophila quinquestriata	Five-striped Sparrow	H	L	H	**L**
Aimophila rufescens	Rusty Sparrow	L	L	L	**L**
Aimophila ruficauda	Stripe-headed Sparrow	L	L	L	**L**
Aimophila ruficeps	Rufous-crowned Sparrow	H	L	L	**L**

Aimophila stolzmanni	Tumbes Sparrow	H	L	H	**L**
Aimophila strigiceps	Stripe-capped Sparrow	H	L	L	**L**
Aimophila sumichrasti	Cinnamon-tailed Sparrow	L	U	H	**L**
Aix galericulata	Mandarin Duck	H	H	H	**H**
Aix sponsa	Wood Duck	H	H	L	**L**
Alaemon alaudipes	Greater Hoopoe-lark	H	L	H	**L**
Alaemon hamertoni	Lesser Hoopoe-lark	H	L	L	**L**
Alauda arvensis	Eurasian Skylark	H	L	H	**L**
Alauda gulgula	Oriental Skylark	U	L	H	**L**
Alauda japonica	Japanese Skylark	U	L	U	**L**
Alauda razae	Raso Lark	H	H	U	**L**
Alca torda	Razorbill	H	H	L	**L**
Alcedo argentata	Silvery Kingfisher	H	U	H	**L**
Alcedo atthis	Common Kingfisher	H	L	H	**L**
Alcedo azurea	Azure Kingfisher	U	L	U	**L**
Alcedo coerulescens	Small Blue Kingfisher	H	L	U	**L**
Alcedo cristata	Malachite Kingfisher	U	L	U	**L**
Alcedo cyanopectus	Indigo-banded Kingfisher	H	U	L	**L**
Alcedo euryzona	Blue-banded Kingfisher	H	L	H	**L**
Alcedo hercules	Blyth's Kingfisher	U	L	H	**L**
Alcedo leucogaster	White-bellied Kingfisher	H	H	H	**H**
Alcedo meninting	Blue-eared Kingfisher	U	L	U	**L**
Alcedo nais	Principe Kingfisher	H	U	L	**L**
Alcedo pusilla	Little Kingfisher	U	L	L	**L**
Alcedo quadribrachys	Shining Blue Kingfisher	H	L	H	**L**
Alcedo semitorquata	Half-collared Kingfisher	U	L	L	**L**
Alcedo thomensis	Sao Tome Kingfisher	H	U	L	**L**
Alcedo vintsioides	Madagascar Kingfisher	U	L	U	**L**
Alcedo websteri	Bismarck Kingfisher	H	U	H	**L**
Alcippe brunnea	Dusky Fulvetta	H	H	U	**L**
Alcippe brunneicauda	Brown Fulvetta	H	U	H	**L**
Alcippe castaneceps	Rufous-winged Fulvetta	H	H	H	**H**
Alcippe chrysotis	Golden-breasted Fulvetta	H	L	U	**L**
Alcippe cinerea	Yellow-throated Fulvetta	U	L	U	**L**
Alcippe cinereiceps	Grey-hooded Fulvetta	U	U	U	**L**
Alcippe dubia	Rusty-capped Fulvetta	H	H	U	**L**
Alcippe formosana	Taiwan Fulvetta	U	U	U	**L**
Alcippe grotei	Black-browed Fulvetta	U	L	U	**L**
Alcippe klossi	Black-crowned Fulvetta	H	H	U	**L**
Alcippe ludlowi	Ludlow's Fulvetta	U	U	H	**L**
Alcippe manipurensis	Streak-throated Fulvetta	U	U	U	**L**
Alcippe morrisonia	Grey-cheeked Fulvetta	U	L	U	**L**
Alcippe nipalensis	Nepal Fulvetta	U	L	U	**L**
Alcippe peracensis	Mountain Fulvetta	H	H	U	**L**
Alcippe poioicephala	Brown-cheeked Fulvetta	U	L	U	**L**

Alcippe pyrrhoptera	Javan Fulvetta	H	H	H	**H**
Alcippe ruficapilla	Spectacled Fulvetta	U	U	U	**L**
Alcippe rufogularis	Rufous-throated Fulvetta	U	L	U	**L**
Alcippe striaticollis	Chinese Fulvetta	H	L	U	**L**
Alcippe variegaticeps	Gold-fronted Fulvetta	H	H	L	**L**
Alcippe vinipectus	White-browed Fulvetta	U	L	U	**L**
Aleadryas rufinucha	Rufous-naped Whistler	U	H	U	**L**
Alectoris barbara	Barbary Partridge	H	L	H	**L**
Alectoris chukar	Chukar	H	H	H	**H**
Alectoris graeca	Rock Partridge	H	L	L	**L**
Alectoris magna	Rusty-necklaced Partridge	H	U	H	**L**
Alectoris melanocephala	Arabian Partridge	U	L	U	**L**
Alectoris philbyi	Philby's Partridge	H	L	H	**L**
Alectoris rufa	Red-legged Partridge	H	L	L	**L**
Alectroenas madagascariensis	Madagascar Blue-pigeon	U	H	H	**L**
Alectroenas pulcherrima	Seychelles Blue-pigeon	H	H	L	**L**
Alectroenas sganzini	Comoro Blue-pigeon	H	H	H	**H**
Alectrurus risora	Strange-tailed Tyrant	H	L	H	**L**
Alectrurus tricolor	Cock-tailed Tyrant	H	U	L	**L**
Alectura lathami	Australian Brush-turkey	L	H	L	**L**
Alethe choloensis	Thyolo Alethe	H	H	L	**L**
Alethe diademata	White-tailed Alethe	H	H	L	**L**
Alethe fuelleborni	White-chested Alethe	H	H	L	**L**
Alethe poliocephala	Brown-chested Alethe	U	H	H	**L**
Alethe poliophrys	Red-throated Alethe	H	H	H	**H**
Alipiopsitta xanthops	Yellow-faced Amazon	H	H	L	**L**
Alisterus amboinensis	Moluccan King-parrot	U	H	U	**L**
Alisterus chloropterus	Papuan King-parrot	H	H	U	**L**
Alisterus scapularis	Australian King-parrot	H	H	L	**L**
Alle alle	Little Auk	H	H	L	**L**
Alophoixus affinis	Golden Bulbul	U	U	U	**L**
Alophoixus bres	Grey-cheeked Bulbul	U	H	U	**L**
Alophoixus finschii	Finsch's Bulbul	H	U	H	**L**
Alophoixus flaveolus	White-throated Bulbul	U	L	U	**L**
Alophoixus ochraceus	Ochraceous Bulbul	H	H	U	**L**
Alophoixus pallidus	Puff-throated Bulbul	H	H	U	**L**
Alophoixus phaeocephalus	Yellow-bellied Bulbul	H	H	H	**H**
Alopochelidon fucata	Tawny-headed Swallow	U	L	L	**L**
Alopochen aegyptiaca	Egyptian Goose	L	H	L	**L**
Amadina erythrocephala	Red-headed Finch	H	L	H	**L**
Amadina fasciata	Cut-throat	U	U	H	**L**
Amalocichla incerta	Lesser Ground-robin	H	H	U	**L**
Amalocichla sclateriana	Greater Ground-robin	H	U	H	**L**

Amandava amandava	Red Avadavat	U	L	L	**L**
Amandava formosa	Green Avadavat	H	L	H	**L**
Amandava subflava	Zebra Waxbill	U	L	L	**L**
Amaurocichla bocagei	Sao Tome Short-tail	H	H	L	**L**
Amaurolimnas concolor	Uniform Crake	H	L	H	**L**
Amaurornis akool	Brown Crake	U	L	U	**L**
Amaurornis bicolor	Black-tailed Crake	U	L	U	**L**
Amaurornis flavirostra	Black Crake	L	L	L	**L**
Amaurornis isabellina	Isabelline Waterhen	U	U	H	**L**
Amaurornis magnirostris	Talaud Bush-hen	H	H	H	**H**
Amaurornis moluccana	Rufous-tailed Waterhen	U	L	U	**L**
Amaurornis olivacea	Bush-hen	U	L	U	**L**
Amaurornis olivieri	Sakalava Rail	H	H	L	**L**
Amaurornis phoenicurus	White-breasted Waterhen	U	L	U	**L**
Amaurospiza carrizalensis	Carrizal Seedeater	H	H	H	**H**
Amaurospiza concolor	Blue Seedeater	H	H	H	**H**
Amaurospiza moesta	Blackish-blue Seedeater	H	H	L	**L**
Amazilia amabilis	Blue-chested Hummingbird	U	H	L	**L**
Amazilia amazilia	Amazilia Hummingbird	H	H	H	**H**
Amazilia beryllina	Berylline Hummingbird	L	H	L	**L**
Amazilia boucardi	Mangrove Hummingbird	H	H	H	**H**
Amazilia brevirostris	White-chested Emerald	H	H	H	**H**
Amazilia candida	White-bellied Emerald	H	H	H	**H**
Amazilia castaneiventris	Chestnut-bellied Hummingbird	H	H	H	**H**
Amazilia chionogaster	White-bellied Hummingbird	U	H	H	**L**
Amazilia cyanifrons	Indigo-capped Hummingbird	U	H	H	**L**
Amazilia cyanocephala	Azure-crowned Hummingbird	L	H	H	**L**
Amazilia cyanura	Blue-tailed Hummingbird	L	U	H	**L**
Amazilia decora	Charming Hummingbird	U	H	H	**L**
Amazilia edward	Snowy-breasted Hummingbird	U	H	H	**L**
Amazilia fimbriata	Glittering-throated Emerald	U	H	H	**L**
Amazilia franciae	Andean Emerald	H	H	H	**H**
Amazilia lactea	Sapphire-spangled Emerald	U	H	H	**L**
Amazilia leucogaster	Plain-bellied Emerald	H	H	H	**H**
Amazilia luciae	Honduran Emerald	H	H	H	**H**
Amazilia rosenbergi	Purple-chested Hummingbird	H	H	H	**H**
Amazilia rutila	Cinnamon Hummingbird	L	H	H	**L**
Amazilia saucerrottei	Steely-vented Hummingbird	U	H	H	**L**
Amazilia tobaci	Copper-rumped Hummingbird	U	H	H	**L**
Amazilia tzacatl	Rufous-tailed Hummingbird	L	H	L	**L**
Amazilia versicolor	Versicoloured Emerald	U	H	H	**L**

Amazilia violiceps	Violet-crowned Hummingbird	L	H	H	**L**
Amazilia viridicauda	Green-and-white Hummingbird	H	H	H	**H**
Amazilia viridifrons	Green-fronted Hummingbird	L	H	L	**L**
Amazilia viridigaster	Green-bellied Hummingbird	U	H	H	**L**
Amazilia yucatanensis	Buff-bellied Hummingbird	L	H	H	**L**
Amazona aestiva	Blue-fronted Amazon	U	H	L	**L**
Amazona agilis	Black-billed Amazon	H	H	H	**H**
Amazona albifrons	White-fronted Amazon	H	H	H	**H**
Amazona amazonica	Orange-winged Amazon	H	H	H	**H**
Amazona arausiaca	Red-necked Amazon	H	H	H	**H**
Amazona auropalliata	Yellow-naped Amazon	H	H	H	**H**
Amazona autumnalis	Red-lored Amazon	H	H	H	**H**
Amazona barbadensis	Yellow-shouldered Amazon	H	H	H	**H**
Amazona brasiliensis	Red-tailed Amazon	H	H	H	**H**
Amazona collaria	Yellow-billed Amazon	H	H	H	**H**
Amazona dufresniana	Blue-cheeked Amazon	H	H	H	**H**
Amazona farinosa	Mealy Amazon	H	H	H	**H**
Amazona festiva	Festive Amazon	H	H	H	**H**
Amazona finschi	Lilac-crowned Amazon	H	H	H	**H**
Amazona guildingii	St Vincent Amazon	H	H	H	**H**
Amazona imperialis	Imperial Amazon	H	H	H	**H**
Amazona kawalli	Kawall's Amazon	H	H	H	**H**
Amazona leucocephala	Cuban Amazon	U	H	H	**L**
Amazona mercenaria	Scaly-naped Amazon	U	H	L	**L**
Amazona ochrocephala	Yellow-crowned Amazon	H	H	H	**H**
Amazona oratrix	Yellow-headed Amazon	H	H	H	**H**
Amazona pretrei	Red-spectacled Amazon	H	H	L	**L**
Amazona rhodocorytha	Red-browed Amazon	H	H	H	**H**
Amazona tucumana	Tucuman Amazon	H	H	H	**H**
Amazona ventralis	Hispaniolan Amazon	H	H	H	**H**
Amazona versicolor	St Lucia Amazon	H	H	H	**H**
Amazona vinacea	Vinaceous Amazon	H	H	L	**L**
Amazona viridigenalis	Red-crowned Amazon	H	H	H	**H**
Amazona vittata	Puerto Rican Amazon	H	H	H	**H**
Amazona xantholora	Yellow-lored Amazon	H	H	H	**H**
Amazonetta brasiliensis	Brazilian Teal	L	H	L	**L**
Amblycercus holosericeus	Yellow-billed Cacique	L	H	L	**L**
Amblyornis flavifrons	Golden-fronted Bowerbird	H	H	H	**H**
Amblyornis inornata	Vogelkop Bowerbird	H	H	H	**H**
Amblyornis macgregoriae	Macgregor's Bowerbird	H	H	H	**H**
Amblyornis subalaris	Streaked Bowerbird	H	H	H	**H**
Amblyospiza albifrons	Grosbeak Weaver	U	U	H	**L**
Amblyramphus	Scarlet-headed Blackbird	H	L	L	**L**

holosericeus					
Ammodramus aurifrons	Yellow-browed Sparrow	H	L	L	**L**
Ammodramus bairdii	Baird's Sparrow	H	L	H	**L**
Ammodramus caudacutus	Saltmarsh Sharp-tailed Sparrow	H	L	H	**L**
Ammodramus henslowii	Henslow's Sparrow	H	L	L	**L**
Ammodramus humeralis	Grassland Sparrow	U	L	L	**L**
Ammodramus leconteii	Le Conte's Sparrow	H	L	L	**L**
Ammodramus maritimus	Seaside Sparrow	H	L	H	**L**
Ammodramus nelsoni	Nelson's Sharp-tailed Sparrow	H	L	L	**L**
Ammodramus savannarum	Grasshopper Sparrow	H	L	L	**L**
Ammomanes cinctura	Bar-tailed Lark	H	L	H	**L**
Ammomanes deserti	Desert Lark	H	L	H	**L**
Ammomanes grayi	Gray's Lark	H	H	H	**H**
Ammomanes phoenicura	Rufous-tailed Lark	U	L	U	**L**
Ammoperdix griseogularis	See-see Partridge	H	L	H	**L**
Ammoperdix heyi	Sand Partridge	U	L	U	**L**
Ampeliceps coronatus	Golden-crested Myna	H	L	U	**L**
Ampelioides tschudii	Scaled Fruiteater	H	U	H	**L**
Ampelion rubrocristatus	Red-crested Cotinga	U	U	H	**L**
Ampelion rufaxilla	Chestnut-crested Cotinga	H	U	L	**L**
Amphilais seebohmi	Grey Emu-tail	U	H	H	**L**
Amphispiza belli	Sage Sparrow	H	L	H	**L**
Amphispiza bilineata	Black-throated Sparrow	H	L	H	**L**
Amytornis barbatus	Grey Grasswren	H	H	L	**L**
Amytornis dorotheae	Carpentarian Grasswren	L	H	L	**L**
Amytornis goyderi	Eyrean Grasswren	H	H	H	**H**
Amytornis housei	Black Grasswren	U	H	L	**L**
Amytornis merrotsyi	Short-tailed Grasswren	U	H	U	**L**
Amytornis purnelli	Dusky Grasswren	U	H	U	**L**
Amytornis striatus	Striated Grasswren	H	H	H	**H**
Amytornis textilis	Thick-billed Grasswren	U	H	U	**L**
Amytornis woodwardi	White-throated Grasswren	H	H	L	**L**
Anabacerthia amaurotis	White-browed Foliage-gleaner	H	H	L	**L**
Anabacerthia striaticollis	Montane Foliage-gleaner	H	H	L	**L**
Anabacerthia variegaticeps	Scaly-throated Foliage-gleaner	H	H	L	**L**
Anabazenops dorsalis	Dusky-cheeked Foliage-gleaner	H	U	H	**L**
Anabazenops fuscus	White-collared Foliage-gleaner	H	H	L	**L**
Anairetes agilis	Agile Tit-tyrant	H	U	H	**L**
Anairetes agraphia	Unstreaked Tit-tyrant	H	U	H	**L**

Anairetes alpinus	Ash-breasted Tit-tyrant	H	H	H	**H**
Anairetes fernandezianus	Juan Fernandez Tit-tyrant	H	H	H	**H**
Anairetes flavirostris	Yellow-billed Tit-tyrant	H	L	L	**L**
Anairetes nigrocristatus	Maranon Tit-tyrant	H	U	H	**L**
Anairetes parulus	Tufted Tit-tyrant	U	L	L	**L**
Anairetes reguloides	Pied-crested Tit-tyrant	H	U	H	**L**
Anaplectes rubriceps	Red-headed Weaver	U	U	L	**L**
Anarhynchus frontalis	Wrybill	H	H	H	**H**
Anas acuta	Northern Pintail	H	H	H	**H**
Anas americana	American Wigeon	H	H	L	**L**
Anas aucklandica	Auckland Islands Teal	H	H	H	**H**
Anas bahamensis	White-cheeked Pintail	U	H	U	**L**
Anas bernieri	Madagascar Teal	H	H	L	**L**
Anas capensis	Cape Teal	H	H	L	**L**
Anas castanea	Chestnut Teal	H	H	U	**L**
Anas chlorotis	Brown Teal	H	H	H	**H**
Anas clypeata	Northern Shoveler	H	H	H	**H**
Anas crecca	Common Teal	H	H	H	**H**
Anas cyanoptera	Cinnamon Teal	L	H	L	**L**
Anas discors	Blue-winged Teal	L	H	L	**L**
Anas eatoni	Eaton's Pintail	H	H	U	**L**
Anas erythrorhyncha	Red-billed Duck	L	H	L	**L**
Anas falcata	Falcated Duck	H	H	H	**H**
Anas flavirostris	Speckled Teal	L	H	L	**L**
Anas formosa	Baikal Teal	L	H	H	**L**
Anas fulvigula	Mottled Duck	H	H	H	**H**
Anas georgica	Yellow-billed Pintail	U	H	U	**L**
Anas gibberifrons	Sunda Teal	H	H	H	**H**
Anas gracilis	Grey Teal	H	H	U	**L**
Anas hottentota	Hottentot Teal	L	H	L	**L**
Anas laysanensis	Laysan Duck	H	H	H	**H**
Anas luzonica	Philippine Duck	H	H	L	**L**
Anas melleri	Meller's Duck	H	H	H	**H**
Anas nesiotis	Campbell Islands Teal	H	H	H	**H**
Anas penelope	Eurasian Wigeon	H	H	H	**H**
Anas platalea	Red Shoveler	U	H	U	**L**
Anas platyrhynchos	Mallard	H	H	H	**H**
Anas poecilorhyncha	Spot-billed Duck	U	H	U	**L**
Anas puna	Puna Teal	H	H	H	**H**
Anas querquedula	Garganey	L	H	H	**L**
Anas rhynchotis	Australasian Shoveler	U	H	U	**L**
Anas rubripes	American Black Duck	H	H	L	**L**
Anas sibilatrix	Chiloe Wigeon	L	H	L	**L**
Anas smithii	Cape Shoveler	H	H	L	**L**
Anas sparsa	African Black Duck	L	H	L	**L**

Anas strepera	Gadwall	H	H	H	**H**
Anas superciliosa	Pacific Black Duck	U	H	U	**L**
Anas undulata	Yellow-billed Duck	L	H	L	**L**
Anas versicolor	Silver Teal	U	H	U	**L**
Anas wyvilliana	Hawaiian Duck	H	H	H	**H**
Anastomus lamelligerus	African Openbill	L	H	L	**L**
Anastomus oscitans	Asian Openbill	U	H	U	**L**
Ancistrops strigilatus	Chestnut-winged Hookbill	H	H	L	**L**
Andigena cucullata	Hooded Mountain-toucan	H	H	H	**H**
Andigena hypoglauca	Grey-breasted Mountain-toucan	H	H	H	**H**
Andigena laminirostris	Plate-billed Mountain-toucan	H	H	H	**H**
Andigena nigrirostris	Black-billed Mountain-toucan	U	H	H	**L**
Androdon aequatorialis	Tooth-billed Hummingbird	H	U	H	**L**
Andropadus ansorgei	Ansorge's Greenbul	H	H	L	**L**
Andropadus curvirostris	Plain Greenbul	U	U	U	**L**
Andropadus gracilirostris	Slender-billed Greenbul	H	U	L	**L**
Andropadus gracilis	Grey Greenbul	H	H	H	**H**
Andropadus importunus	Sombre Greenbul	U	H	L	**L**
Andropadus latirostris	Yellow-whiskered Greenbul	U	H	U	**L**
Andropadus masukuensis	Shelley's Greenbul	H	H	U	**L**
Andropadus milanjensis	Stripe-cheeked Greenbul	U	H	U	**L**
Andropadus montanus	Cameroon Montane Greenbul	U	U	H	**L**
Andropadus nigriceps	Eastern Mountain Greenbul	U	H	U	**L**
Andropadus tephrolaemus	Western Mountain Greenbul	U	H	L	**L**
Andropadus virens	Little Greenbul	U	H	U	**L**
Androphobus viridis	Papuan Whipbird	H	U	H	**L**
Anhima cornuta	Horned Screamer	H	L	H	**L**
Anhinga anhinga	Anhinga	U	H	L	**L**
Anhinga melanogaster	Oriental Darter	L	H	L	**L**
Anhinga novaehollandiae	Australian Darter	U	H	U	**L**
Anhinga rufa	African Darter	L	H	L	**L**
Anisognathus igniventris	Scarlet-bellied Mountain-tanager	U	H	H	**L**
Anisognathus lacrymosus	Lacrimose Mountain-tanager	H	U	H	**L**
Anisognathus melanogenys	Santa Marta Mountain-tanager	U	U	H	**L**
Anisognathus notabilis	Black-chinned Mountain-tanager	H	U	H	**L**
Anisognathus somptuosus	Blue-winged Mountain-tanager	H	U	L	**L**
Anodorhynchus glaucus	Glaucous Macaw	H	H	L	**L**
Anodorhynchus	Hyacinth Macaw	H	H	L	**L**

hyacinthinus					
Anodorhynchus leari	Lear's Macaw	H	H	L	**L**
Anomalospiza imberbis	Cuckoo Finch	U	U	L	**L**
Anopetia gounellei	Broad-tipped Hermit	H	U	L	**L**
Anorrhinus austeni	Austen's Brown Hornbill	H	H	L	**L**
Anorrhinus galeritus	Bushy-crested Hornbill	H	H	H	**H**
Anorrhinus tickelli	Tickell's Brown Hornbill	H	H	H	**H**
Anous minutus	Black Noddy	L	H	L	**L**
Anous stolidus	Brown Noddy	L	H	L	**L**
Anous tenuirostris	Lesser Noddy	L	H	H	**L**
Anser albifrons	Greater White-fronted Goose	H	H	H	**H**
Anser anser	Greylag Goose	H	H	H	**H**
Anser brachyrhynchus	Pink-footed Goose	H	H	H	**H**
Anser cygnoides	Swan Goose	H	H	H	**H**
Anser erythropus	Lesser White-fronted Goose	H	H	H	**H**
Anser fabalis	Bean Goose	H	H	H	**H**
Anser indicus	Bar-headed Goose	L	H	H	**L**
Anseranas semipalmata	Magpie Goose	L	H	L	**L**
Anthocephala floriceps	Blossomcrown	H	U	H	**L**
Anthochaera carunculata	Red Wattlebird	H	H	L	**L**
Anthochaera chrysoptera	Little Wattlebird	U	H	U	**L**
Anthochaera paradoxa	Yellow Wattlebird	H	H	H	**H**
Anthornis melanura	New Zealand Bellbird	U	L	H	**L**
Anthoscopus caroli	African Penduline-tit	U	L	U	**L**
Anthoscopus flavifrons	Forest Penduline-tit	H	U	H	**L**
Anthoscopus minutus	Southern Penduline-tit	H	L	L	**L**
Anthoscopus musculus	Mouse-coloured Penduline-tit	U	L	U	**L**
Anthoscopus parvulus	Yellow Penduline-tit	U	H	U	**L**
Anthoscopus punctifrons	Sennar Penduline-tit	H	U	H	**L**
Anthracoceros albirostris	Oriental Pied Hornbill	H	H	L	**L**
Anthracoceros coronatus	Malabar Pied Hornbill	H	H	L	**L**
Anthracoceros malayanus	Black Hornbill	H	H	H	**H**
Anthracoceros marchei	Palawan Hornbill	H	H	H	**H**
Anthracoceros montani	Sulu Hornbill	H	H	L	**L**
Anthracothorax dominicus	Antillean Mango	U	H	H	**L**
Anthracothorax mango	Jamaican Mango	H	H	H	**H**
Anthracothorax nigricollis	Black-throated Mango	H	H	H	**H**
Anthracothorax prevostii	Green-breasted Mango	H	H	H	**H**
Anthracothorax veraguensis	Veraguan Mango	H	U	H	**L**
Anthracothorax viridigula	Green-throated Mango	H	H	H	**H**
Anthracothorax viridis	Green Mango	H	H	H	**H**

Anthreptes anchietae	Anchieta's Sunbird	H	H	U	**L**
Anthreptes aurantium	Violet-tailed Sunbird	H	H	H	**H**
Anthreptes collaris	Collared Sunbird	U	L	L	**L**
Anthreptes fraseri	Scarlet-tufted Sunbird	U	U	U	**L**
Anthreptes gabonicus	Mouse-brown Sunbird	H	H	H	**H**
Anthreptes longuemarei	Western Violet-backed Sunbird	U	H	L	**L**
Anthreptes malacensis	Plain-throated Sunbird	U	H	U	**L**
Anthreptes metallicus	Nile Valley Sunbird	U	H	U	**L**
Anthreptes neglectus	Uluguru Violet-backed Sunbird	U	U	L	**L**
Anthreptes orientalis	Kenya Violet-backed Sunbird	U	H	U	**L**
Anthreptes pallidigaster	Amani Sunbird	H	H	H	**H**
Anthreptes platurus	Pygmy Sunbird	U	H	H	**L**
Anthreptes rectirostris	Green Sunbird	H	H	L	**L**
Anthreptes reichenowi	Plain-backed Sunbird	U	L	L	**L**
Anthreptes rhodolaemus	Red-throated Sunbird	H	H	H	**H**
Anthreptes rubritorques	Banded Sunbird	H	H	H	**H**
Anthreptes simplex	Plain Sunbird	U	H	U	**L**
Anthreptes singalensis	Ruby-cheeked Sunbird	U	H	U	**L**
Anthus antarcticus	South Georgia Pipit	H	L	H	**L**
Anthus berthelotii	Berthelot's Pipit	H	L	H	**L**
Anthus bogotensis	Paramo Pipit	U	U	H	**L**
Anthus brachyurus	Short-tailed Pipit	U	L	U	**L**
Anthus caffer	Bush Pipit	U	L	U	**L**
Anthus campestris	Tawny Pipit	H	L	L	**L**
Anthus cervinus	Red-throated Pipit	L	H	H	**L**
Anthus chacoensis	Chaco Pipit	H	U	H	**L**
Anthus chloris	Yellow-breasted Pipit	H	L	L	**L**
Anthus correndera	Correndera Pipit	U	L	L	**L**
Anthus crenatus	Yellow-tufted Pipit	H	H	H	**H**
Anthus furcatus	Short-billed Pipit	H	U	L	**L**
Anthus godlewskii	Blyth's Pipit	U	L	U	**L**
Anthus gustavi	Pechora Pipit	H	H	H	**H**
Anthus gutturalis	Alpine Pipit	H	U	H	**L**
Anthus hellmayri	Hellmayr's Pipit	U	U	L	**L**
Anthus hodgsoni	Olive-backed Pipit	U	L	H	**L**
Anthus hoeschi	Mountain Pipit	H	H	L	**L**
Anthus leucophrys	Plain-backed Pipit	U	L	U	**L**
Anthus lineiventris	Striped Pipit	U	L	U	**L**
Anthus longicaudatus	Long-tailed Pipit	H	U	H	**L**
Anthus lutescens	Yellowish Pipit	U	L	L	**L**
Anthus melindae	Malindi Pipit	H	L	L	**L**
Anthus nattereri	Ochre-breasted Pipit	H	L	L	**L**
Anthus nilghiriensis	Nilgiri Pipit	U	L	H	**L**

Anthus novaeseelandiae	Australasian Pipit	H	L	L	**L**
Anthus pallidiventris	Long-legged Pipit	U	U	U	**L**
Anthus petrosus	Rock Pipit	H	L	H	**L**
Anthus pratensis	Meadow Pipit	H	L	H	**L**
Anthus richardi	Richard's Pipit	U	L	U	**L**
Anthus roseatus	Rosy Pipit	U	L	U	**L**
Anthus rubescens	American Pipit	H	H	H	**H**
Anthus rufulus	Paddyfield Pipit	U	L	U	**L**
Anthus similis	Long-billed Pipit	U	L	U	**L**
Anthus sokokensis	Sokoke Pipit	H	U	H	**L**
Anthus spinoletta	Water Pipit	H	L	L	**L**
Anthus spragueii	Sprague's Pipit	H	L	H	**L**
Anthus sylvanus	Upland Pipit	U	L	U	**L**
Anthus trivialis	Tree Pipit	H	L	H	**L**
Anthus vaalensis	Buffy Pipit	U	L	U	**L**
Antilophia bokermanni	Araripe Manakin	H	H	L	**L**
Antilophia galeata	Helmeted Manakin	H	H	L	**L**
Anumbius annumbi	Firewood-gatherer	H	L	L	**L**
Anurolimnas castaneiceps	Chestnut-headed Crake	H	U	H	**L**
Anurolimnas fasciatus	Black-banded Crake	H	U	L	**L**
Anurolimnas viridis	Russet-crowned Crake	U	H	H	**L**
Anurophasis monorthonyx	Snow Mountain Quail	H	H	H	**H**
Apalharpactes mackloti	Sumatran Trogon	H	H	U	**L**
Apalharpactes reinwardtii	Javan Trogon	H	H	H	**H**
Apalis argentea	Kungwe Apalis	H	H	L	**L**
Apalis bamendae	Bamenda Apalis	H	H	L	**L**
Apalis binotata	Masked Apalis	U	H	H	**L**
Apalis chapini	Chapin's Apalis	H	H	L	**L**
Apalis chariessa	White-winged Apalis	H	H	L	**L**
Apalis chirindensis	Chirinda Apalis	H	H	L	**L**
Apalis cinerea	Grey Apalis	U	L	U	**L**
Apalis flavida	Yellow-breasted Apalis	U	L	U	**L**
Apalis flavigularis	Yellow-throated Apalis	H	H	L	**L**
Apalis fuscigularis	Taita Apalis	H	H	H	**H**
Apalis goslingi	Gosling's Apalis	H	U	H	**L**
Apalis jacksoni	Black-throated Apalis	U	H	L	**L**
Apalis karamojae	Karamoja Apalis	H	U	L	**L**
Apalis lynesi	Namuli Apalis	L	H	L	**L**
Apalis melanocephala	Black-headed Apalis	U	L	L	**L**
Apalis nigriceps	Black-capped Apalis	U	U	L	**L**
Apalis personata	Black-faced Apalis	U	U	L	**L**
Apalis porphyrolaema	Chestnut-throated Apalis	H	H	U	**L**
Apalis pulchra	Black-collared Apalis	H	H	H	**H**
Apalis ruddi	Rudd's Apalis	H	H	L	**L**
Apalis rufogularis	Buff-throated Apalis	H	H	H	**H**

Apalis ruwenzorii	Collared Apalis	U	H	L	**L**
Apalis sharpii	Sharpe's Apalis	H	H	L	**L**
Apalis thoracica	Bar-throated Apalis	U	L	L	**L**
Apaloderma aequatoriale	Bare-cheeked Trogon	H	H	H	**H**
Apaloderma narina	Narina Trogon	H	H	L	**L**
Apaloderma vittatum	Bar-tailed Trogon	H	H	L	**L**
Apalopteron familiare	Bonin White-eye	H	H	U	**L**
Aphanotriccus audax	Black-billed Flycatcher	H	H	H	**H**
Aphanotriccus capitalis	Tawny-chested Flycatcher	H	U	H	**L**
Aphantochroa cirrochloris	Sombre Hummingbird	U	H	L	**L**
Aphelocephala leucopsis	Southern Whiteface	H	L	L	**L**
Aphelocephala nigricincta	Banded Whiteface	U	L	U	**L**
Aphelocephala pectoralis	Chestnut-breasted Whiteface	H	L	H	**L**
Aphelocoma californica	Western Scrub-jay	H	H	H	**H**
Aphelocoma coerulescens	Florida Scrub-jay	H	H	L	**L**
Aphelocoma insularis	Island Scrub-jay	H	H	U	**L**
Aphelocoma ultramarina	Mexican Jay	H	H	L	**L**
Aphelocoma unicolor	Unicoloured Jay	L	H	H	**L**
Aphrastura masafuerae	Masafuera Rayadito	H	H	U	**L**
Aphrastura spinicauda	Thorn-tailed Rayadito	H	L	H	**L**
Aphriza virgata	Surfbird	L	H	L	**L**
Aplonis atrifusca	Samoan Starling	H	U	U	**L**
Aplonis brunneicapillus	White-eyed Starling	H	U	H	**L**
Aplonis cantoroides	Singing Starling	U	L	U	**L**
Aplonis cinerascens	Rarotonga Starling	H	H	U	**L**
Aplonis crassa	Tanimbar Starling	H	U	H	**L**
Aplonis dichroa	San Cristobal Starling	H	U	H	**L**
Aplonis feadensis	Atoll Starling	H	H	U	**L**
Aplonis grandis	Brown-winged Starling	H	U	H	**L**
Aplonis insularis	Rennell Starling	H	H	H	**H**
Aplonis magna	Long-tailed Starling	H	U	H	**L**
Aplonis metallica	Metallic Starling	H	L	L	**L**
Aplonis minor	Short-tailed Starling	H	U	U	**L**
Aplonis mysolensis	Moluccan Starling	U	U	U	**L**
Aplonis mystacea	Yellow-eyed Starling	H	U	H	**L**
Aplonis opaca	Micronesian Starling	H	L	H	**L**
Aplonis panayensis	Asian Glossy Starling	H	L	L	**L**
Aplonis pelzelni	Pohnpei Starling	H	H	H	**H**
Aplonis santovestris	Santo Starling	H	H	L	**L**
Aplonis striata	Striated Starling	U	U	H	**L**
Aplonis tabuensis	Polynesian Starling	H	L	L	**L**
Aplonis zelandica	Rusty-winged Starling	H	U	H	**L**
Aplopelia larvata	Lemon Dove	U	H	L	**L**
Aprosmictus	Red-winged Parrot	U	H	L	**L**

erythropterus					
Aprosmictus jonquillaceus	Olive-shouldered Parrot	H	H	L	**L**
Aptenodytes forsteri	Emperor Penguin	H	H	L	**L**
Aptenodytes patagonicus	King Penguin	H	H	L	**L**
Apteryx australis	Southern Brown Kiwi	L	H	H	**L**
Apteryx haastii	Great Spotted Kiwi	L	H	H	**L**
Apteryx mantelli	Northern Brown Kiwi	H	H	H	**H**
Apteryx owenii	Little Spotted Kiwi	H	H	H	**H**
Apus acuticauda	Dark-rumped Swift	H	H	H	**H**
Apus affinis	Little Swift	U	H	L	**L**
Apus alexandri	Alexander's Swift	H	H	H	**H**
Apus apus	Common Swift	H	H	H	**H**
Apus balstoni	Madagascar Swift	H	H	L	**L**
Apus barbatus	African Black Swift	H	H	U	**L**
Apus batesi	Bates's Swift	H	H	H	**H**
Apus berliozi	Forbes-Watson's Swift	H	H	L	**L**
Apus bradfieldi	Bradfield's Swift	H	H	H	**H**
Apus caffer	White-rumped Swift	U	H	L	**L**
Apus horus	Horus Swift	H	H	L	**L**
Apus niansae	Nyanza Swift	H	H	L	**L**
Apus nipalensis	House Swift	U	H	U	**L**
Apus pacificus	Fork-tailed Swift	H	H	H	**H**
Apus pallidus	Pallid Swift	H	H	H	**H**
Apus sladeniae	Fernando Po Swift	H	H	L	**L**
Apus toulsoni	Loanda Swift	H	H	U	**L**
Apus unicolor	Plain Swift	H	H	H	**H**
Aquila adalberti	Spanish Imperial Eagle	H	H	H	**H**
Aquila audax	Wedge-tailed Eagle	H	H	L	**L**
Aquila chrysaetos	Golden Eagle	H	H	H	**H**
Aquila clanga	Greater Spotted Eagle	H	H	H	**H**
Aquila gurneyi	Gurney's Eagle	H	H	L	**L**
Aquila hastata	Indian Spotted Eagle	H	H	L	**L**
Aquila heliaca	Eastern Imperial Eagle	H	H	H	**H**
Aquila nipalensis	Steppe Eagle	H	H	L	**L**
Aquila pomarina	Lesser Spotted Eagle	H	H	L	**L**
Aquila rapax	Tawny Eagle	L	H	L	**L**
Aquila verreauxii	Verreaux's Eagle	H	H	L	**L**
Aquila wahlbergi	Wahlberg's Eagle	L	H	L	**L**
Ara ambiguus	Great Green Macaw	H	H	H	**H**
Ara ararauna	Blue-and-yellow Macaw	H	H	H	**H**
Ara chloropterus	Red-and-green Macaw	H	H	H	**H**
Ara glaucogularis	Blue-throated Macaw	H	H	L	**L**
Ara macao	Scarlet Macaw	H	H	H	**H**
Ara militaris	Military Macaw	H	H	H	**H**
Ara rubrogenys	Red-fronted Macaw	H	H	H	**H**

Ara severus	Chestnut-fronted Macaw	H	H	H	**H**
Arachnothera affinis	Grey-breasted Spiderhunter	U	H	U	**L**
Arachnothera chrysogenys	Yellow-eared Spiderhunter	U	H	U	**L**
Arachnothera clarae	Naked-faced Spiderhunter	U	U	U	**L**
Arachnothera crassirostris	Thick-billed Spiderhunter	U	U	U	**L**
Arachnothera everetti	Bornean Spiderhunter	U	H	U	**L**
Arachnothera flavigaster	Spectacled Spiderhunter	U	U	U	**L**
Arachnothera juliae	Whitehead's Spiderhunter	H	U	H	**L**
Arachnothera longirostra	Little Spiderhunter	U	L	U	**L**
Arachnothera magna	Streaked Spiderhunter	U	L	U	**L**
Arachnothera robusta	Long-billed Spiderhunter	U	H	L	**L**
Aramides axillaris	Rufous-necked Wood-rail	L	L	H	**L**
Aramides cajanea	Grey-necked Wood-rail	L	L	H	**L**
Aramides calopterus	Red-winged Wood-rail	H	U	H	**L**
Aramides mangle	Little Wood-rail	U	U	H	**L**
Aramides saracura	Slaty-breasted Wood-rail	H	L	L	**L**
Aramides wolfi	Brown Wood-rail	H	H	H	**H**
Aramides ypecaha	Giant Wood-rail	L	L	L	**L**
Aramidopsis plateni	Snoring Rail	H	U	H	**L**
Aramus guarauna	Limpkin	L	H	L	**L**
Aratinga acuticaudata	Blue-crowned Parakeet	U	H	L	**L**
Aratinga aurea	Peach-fronted Parakeet	U	H	L	**L**
Aratinga auricapillus	Golden-capped Parakeet	H	H	L	**L**
Aratinga brevipes	Socorro Parakeet	H	H	L	**L**
Aratinga cactorum	Cactus Parakeet	U	H	L	**L**
Aratinga canicularis	Orange-fronted Parakeet	L	H	L	**L**
Aratinga chloroptera	Hispaniolan Parakeet	H	H	H	**H**
Aratinga erythrogenys	Red-masked Parakeet	H	H	H	**H**
Aratinga euops	Cuban Parakeet	H	H	H	**H**
Aratinga finschi	Crimson-fronted Parakeet	U	H	H	**L**
Aratinga holochlora	Green Parakeet	U	H	U	**L**
Aratinga jandaya	Jandaya Parakeet	H	H	L	**L**
Aratinga leucophthalma	White-eyed Parakeet	U	H	L	**L**
Aratinga mitrata	Mitred Parakeet	H	H	H	**H**
Aratinga nana	Olive-throated Parakeet	L	H	H	**L**
Aratinga pertinax	Brown-throated Parakeet	H	H	H	**H**
Aratinga rubritorquis	Red-throated Parakeet	U	H	U	**L**
Aratinga solstitialis	Sun Parakeet	H	H	H	**H**
Aratinga wagleri	Scarlet-fronted Parakeet	U	H	H	**L**
Aratinga weddellii	Dusky-headed Parakeet	H	H	L	**L**
Arborophila ardens	Hainan Partridge	H	H	L	**L**
Arborophila atrogularis	White-cheeked Partridge	U	L	H	**L**
Arborophila brunneopectus	Bar-backed Partridge	H	U	U	**L**

Arborophila cambodiana	Chestnut-headed Partridge	H	H	H	**H**
Arborophila campbelli	Malaysian Partridge	H	U	U	**L**
Arborophila charltonii	Chestnut-necklaced Partridge	H	U	L	**L**
Arborophila chloropus	Scaly-breasted Partridge	H	L	U	**L**
Arborophila crudigularis	Taiwan Partridge	H	L	H	**L**
Arborophila davidi	Orange-necked Partridge	H	U	L	**L**
Arborophila gingica	White-necklaced Partridge	H	L	L	**L**
Arborophila hyperythra	Red-breasted Partridge	H	U	H	**L**
Arborophila javanica	Chestnut-bellied Partridge	H	L	H	**L**
Arborophila mandellii	Chestnut-breasted Partridge	H	L	H	**L**
Arborophila orientalis	White-faced Partridge	H	L	H	**L**
Arborophila rolli	Roll's Partridge	H	U	U	**L**
Arborophila rubrirostris	Red-billed Partridge	H	U	H	**L**
Arborophila rufipectus	Sichuan Partridge	H	L	H	**L**
Arborophila rufogularis	Rufous-throated Partridge	U	L	U	**L**
Arborophila sumatrana	Grey-breasted Partridge	H	U	U	**L**
Arborophila torqueola	Hill Partridge	U	L	U	**L**
Archboldia papuensis	Archbold's Bowerbird	H	H	H	**H**
Archilochus alexandri	Black-chinned Hummingbird	H	H	H	**H**
Archilochus colubris	Ruby-throated Hummingbird	H	H	L	**L**
Ardea cinerea	Grey Heron	L	H	H	**L**
Ardea cocoi	Cocoi Heron	U	H	U	**L**
Ardea goliath	Goliath Heron	L	H	L	**L**
Ardea herodias	Great Blue Heron	H	H	L	**L**
Ardea humbloti	Madagascar Heron	H	H	L	**L**
Ardea insignis	White-bellied Heron	H	H	H	**H**
Ardea melanocephala	Black-headed Heron	L	H	L	**L**
Ardea pacifica	White-necked Heron	U	H	U	**L**
Ardea picata	Pied Heron	U	H	U	**L**
Ardea purpurea	Purple Heron	L	H	L	**L**
Ardea sumatrana	Great-billed Heron	U	H	U	**L**
Ardeola bacchus	Chinese Pond-heron	U	H	U	**L**
Ardeola grayii	Indian Pond-heron	U	H	U	**L**
Ardeola idae	Madagascar Pond-heron	H	H	L	**L**
Ardeola ralloides	Squacco Heron	L	H	L	**L**
Ardeola rufiventris	Rufous-bellied Heron	L	H	L	**L**
Ardeola speciosa	Javan Pond-heron	U	H	U	**L**
Ardeotis arabs	Arabian Bustard	H	H	H	**H**
Ardeotis australis	Australian Bustard	H	H	L	**L**
Ardeotis kori	Kori Bustard	H	H	L	**L**
Ardeotis nigriceps	Great Indian Bustard	H	H	L	**L**
Arenaria interpres	Ruddy Turnstone	L	H	H	**L**
Arenaria melanocephala	Black Turnstone	L	H	H	**L**
Argusianus argus	Great Argus	H	H	L	**L**

Arremon abeillei	Black-capped Sparrow	H	H	H	**H**
Arremon aurantiirostris	Orange-billed Sparrow	L	H	H	**L**
Arremon brunneinucha	Chestnut-capped Brush-finch	L	H	L	**L**
Arremon castaneiceps	Olive Finch	H	H	L	**L**
Arremon crassirostris	Sooty-faced Finch	H	H	H	**H**
Arremon flavirostris	Saffron-billed Sparrow	U	U	L	**L**
Arremon franciscanus	Sao Francisco Sparrow	H	U	L	**L**
Arremon schlegeli	Golden-winged Sparrow	U	U	H	**L**
Arremon semitorquatus	Half-collared Sparrow	H	H	L	**L**
Arremon taciturnus	Pectoral Sparrow	H	H	H	**H**
Arremon torquatus	Stripe-headed Brush-finch	U	H	L	**L**
Arremon virenticeps	Green-striped Brush-finch	H	H	L	**L**
Arremonops chloronotus	Green-backed Sparrow	L	L	H	**L**
Arremonops conirostris	Black-striped Sparrow	U	H	H	**L**
Arremonops rufivirgatus	Olive Sparrow	L	L	H	**L**
Arremonops tocuyensis	Tocuyo Sparrow	H	L	H	**L**
Arses insularis	Rufous-collared Monarch	H	H	H	**H**
Arses kaupi	Pied Monarch	U	H	H	**L**
Arses telescophthalmus	Frilled Monarch	U	H	L	**L**
Artamella viridis	White-headed Vanga	U	L	U	**L**
Artamus cinereus	Black-faced Woodswallow	U	L	U	**L**
Artamus cyanopterus	Dusky Woodswallow	U	L	U	**L**
Artamus fuscus	Ashy Woodswallow	U	L	U	**L**
Artamus insignis	Bismarck Woodswallow	H	U	H	**L**
Artamus leucorynchus	White-breasted Woodswallow	U	L	U	**L**
Artamus maximus	Great Woodswallow	H	U	H	**L**
Artamus mentalis	Fiji Woodswallow	H	L	L	**L**
Artamus minor	Little Woodswallow	U	L	U	**L**
Artamus monachus	Ivory-backed Woodswallow	U	U	H	**L**
Artamus personatus	Masked Woodswallow	H	L	L	**L**
Artamus superciliosus	White-browed Woodswallow	U	L	U	**L**
Artisornis metopias	African Tailorbird	U	H	L	**L**
Artisornis moreaui	Long-billed Tailorbird	H	H	L	**L**
Arundinicola leucocephala	White-headed Marsh-tyrant	U	L	L	**L**
Ashbyia lovensis	Gibberbird	H	L	L	**L**
Asio abyssinicus	Abyssinian Owl	U	H	L	**L**
Asio capensis	Marsh Owl	U	H	L	**L**
Asio flammeus	Short-eared Owl	H	H	H	**H**
Asio madagascariensis	Madagascar Owl	U	H	U	**L**
Asio otus	Long-eared Owl	H	H	H	**H**
Asio stygius	Stygian Owl	L	H	L	**L**
Aspatha gularis	Blue-throated Motmot	H	L	H	**L**
Asthenes anthoides	Austral Canastero	U	U	H	**L**
Asthenes baeri	Short-billed Canastero	H	L	L	**L**

Asthenes berlepschi	Berlepsch's Canastero	U	U	H	**L**
Asthenes cactorum	Cactus Canastero	H	U	H	**L**
Asthenes dorbignyi	Creamy-breasted Canastero	H	H	H	**H**
Asthenes flammulata	Many-striped Canastero	H	U	H	**L**
Asthenes heterura	Maquis Canastero	H	U	H	**L**
Asthenes hudsoni	Hudson's Canastero	H	L	H	**L**
Asthenes humicola	Dusky-tailed Canastero	H	L	L	**L**
Asthenes humilis	Streak-throated Canastero	U	U	H	**L**
Asthenes luizae	Cipo Canastero	H	H	H	**H**
Asthenes maculicauda	Scribble-tailed Canastero	U	U	H	**L**
Asthenes modesta	Cordilleran Canastero	H	L	H	**L**
Asthenes ottonis	Rusty-fronted Canastero	U	U	H	**L**
Asthenes patagonica	Patagonian Canastero	H	L	H	**L**
Asthenes pudibunda	Canyon Canastero	H	U	H	**L**
Asthenes pyrrholeuca	Lesser Canastero	H	L	L	**L**
Asthenes sclateri	Puno Canastero	H	L	H	**L**
Asthenes steinbachi	Steinbach's Canastero	H	L	H	**L**
Asthenes urubambensis	Line-fronted Canastero	U	U	H	**L**
Asthenes virgata	Junin Canastero	H	U	H	**L**
Asthenes wyatti	Streak-backed Canastero	U	L	H	**L**
Astrapia mayeri	Ribbon-tailed Astrapia	H	H	H	**H**
Astrapia nigra	Arfak Astrapia	H	H	H	**H**
Astrapia rothschildi	Huon Astrapia	H	H	H	**H**
Astrapia splendidissima	Splendid Astrapia	U	H	H	**L**
Astrapia stephaniae	Stephanie's Astrapia	U	H	H	**L**
Atalotriccus pilaris	Pale-eyed Pygmy-tyrant	H	U	H	**L**
Atelornis crossleyi	Rufous-headed Ground-roller	H	H	H	**H**
Atelornis pittoides	Pitta-like Ground-roller	H	L	U	**L**
Athene brama	Spotted Owlet	U	L	U	**L**
Athene cunicularia	Burrowing Owl	L	L	L	**L**
Athene noctua	Little Owl	H	L	H	**L**
Atlantisia rogersi	Inaccessible Rail	H	H	U	**L**
Atlapetes albiceps	White-headed Brush-finch	H	U	H	**L**
Atlapetes albinucha	White-naped Brush-finch	L	L	L	**L**
Atlapetes albofrenatus	Moustached Brush-finch	U	U	H	**L**
Atlapetes canigenis	Grey Brush-finch	U	U	H	**L**
Atlapetes citrinellus	Yellow-striped Brush-finch	H	H	H	**H**
Atlapetes flaviceps	Yellow-headed Brush-finch	H	H	H	**H**
Atlapetes forbesi	Apurimac Brush-finch	U	U	H	**L**
Atlapetes fulviceps	Fulvous-headed Brush-finch	H	H	H	**H**
Atlapetes fuscoolivaceus	Dusky-headed Brush-finch	H	U	H	**L**
Atlapetes latinuchus	Yellow-breasted Brush-finch	U	U	H	**L**
Atlapetes leucopis	White-rimmed Brush-finch	U	U	H	**L**
Atlapetes leucopterus	White-winged Brush-finch	U	U	H	**L**

Atlapetes melanocephalus	Santa Marta Brush-finch	U	U	H	**L**
Atlapetes melanolaemus	Black-faced Brush-finch	U	U	H	**L**
Atlapetes melanopsis	Black-spectacled Brush-finch	H	U	H	**L**
Atlapetes nationi	Rusty-bellied Brush-finch	H	U	H	**L**
Atlapetes pallidiceps	Pale-headed Brush-finch	H	H	H	**H**
Atlapetes pallidinucha	Pale-naped Brush-finch	U	U	H	**L**
Atlapetes personatus	Tepui Brush-finch	U	U	H	**L**
Atlapetes pileatus	Rufous-capped Brush-finch	L	U	L	**L**
Atlapetes rufigenis	Rufous-eared Brush-finch	H	U	H	**L**
Atlapetes rufinucha	Rufous-naped Brush-finch	U	U	H	**L**
Atlapetes schistaceus	Slaty Brush-finch	U	U	H	**L**
Atlapetes seebohmi	Bay-crowned Brush-finch	H	U	H	**L**
Atlapetes semirufus	Ochre-breasted Brush-finch	U	U	H	**L**
Atlapetes terborghi	Vilcabamba Brush-finch	H	H	H	**H**
Atlapetes tricolor	Tricoloured Brush-finch	U	U	L	**L**
Atrichornis clamosus	Noisy Scrub-bird	H	H	H	**H**
Atrichornis rufescens	Rufous Scrub-bird	H	H	L	**L**
Attagis gayi	Rufous-bellied Seedsnipe	H	H	H	**H**
Attagis malouinus	White-bellied Seedsnipe	H	H	H	**H**
Atthis ellioti	Wine-throated Hummingbird	H	U	H	**L**
Atthis heloisa	Bumblebee Hummingbird	H	U	L	**L**
Atticora fasciata	White-banded Swallow	H	L	H	**L**
Atticora melanoleuca	Black-collared Swallow	H	L	H	**L**
Attila bolivianus	Dull-capped Attila	H	H	L	**L**
Attila cinnamomeus	Cinnamon Attila	H	L	H	**L**
Attila citriniventris	Citron-bellied Attila	H	H	H	**H**
Attila phoenicurus	Rufous-tailed Attila	U	U	L	**L**
Attila rufus	Grey-hooded Attila	U	U	H	**L**
Attila spadiceus	Bright-rumped Attila	H	L	H	**L**
Attila torridus	Ochraceous Attila	H	U	H	**L**
Augastes lumachella	Hooded Visorbearer	H	H	L	**L**
Augastes scutatus	Hyacinth Visorbearer	H	H	H	**H**
Aulacorhynchus coeruleicinctis	Blue-banded Toucanet	H	H	H	**H**
Aulacorhynchus derbianus	Chestnut-tipped Toucanet	H	H	L	**L**
Aulacorhynchus haematopygus	Crimson-rumped Toucanet	H	H	H	**H**
Aulacorhynchus huallagae	Yellow-browed Toucanet	H	H	H	**H**
Aulacorhynchus prasinus	Emerald Toucanet	H	H	L	**L**
Aulacorhynchus sulcatus	Groove-billed Toucanet	H	H	H	**H**
Auriparus flaviceps	Verdin	H	L	H	**L**
Automolus infuscatus	Olive-backed Foliage-gleaner	H	H	U	**L**
Automolus leucophthalmus	White-eyed Foliage-gleaner	H	H	L	**L**
Automolus melanopezus	Brown-rumped Foliage-	H	H	H	**H**

	gleaner				
Automolus ochrolaemus	Buff-throated Foliage-gleaner	H	L	H	**L**
Automolus roraimae	White-throated Foliage-gleaner	H	H	H	**H**
Automolus rubiginosus	Ruddy Foliage-gleaner	H	H	H	**H**
Automolus rufipileatus	Chestnut-crowned Foliage-gleaner	H	H	H	**H**
Aviceda cuculoides	African Baza	H	H	L	**L**
Aviceda jerdoni	Jerdon's Baza	H	H	L	**L**
Aviceda leuphotes	Black Baza	H	H	H	**H**
Aviceda madagascariensis	Madagascar Baza	H	H	L	**L**
Aviceda subcristata	Pacific Baza	H	H	L	**L**
Avocettula recurvirostris	Fiery-tailed Awlbill	H	H	H	**H**
Aythya affinis	Lesser Scaup	H	H	L	**L**
Aythya americana	Redhead	H	H	L	**L**
Aythya australis	Hardhead	U	H	U	**L**
Aythya baeri	Baer's Pochard	H	H	H	**H**
Aythya collaris	Ring-necked Duck	H	H	L	**L**
Aythya ferina	Common Pochard	H	H	H	**H**
Aythya fuligula	Tufted Duck	H	H	H	**H**
Aythya innotata	Madagascar Pochard	H	H	H	**H**
Aythya marila	Greater Scaup	H	H	L	**L**
Aythya novaeseelandiae	New Zealand Scaup	H	H	U	**L**
Aythya nyroca	Ferruginous Duck	H	H	H	**H**
Aythya valisineria	Canvasback	H	H	L	**L**
Babax koslowi	Tibetan Babax	H	U	H	**L**
Babax lanceolatus	Chinese Babax	U	L	U	**L**
Babax waddelli	Giant Babax	H	L	H	**L**
Baeolophus atricristatus	Black-crested Titmouse	H	L	H	**L**
Baeolophus bicolor	Tufted Titmouse	H	L	L	**L**
Baeolophus inornatus	Oak Titmouse	H	L	L	**L**
Baeolophus ridgwayi	Juniper Titmouse	H	L	H	**L**
Baeolophus wollweberi	Bridled Titmouse	H	L	L	**L**
Baeopogon clamans	White-tailed Greenbul	H	H	H	**H**
Baeopogon indicator	Honeyguide Greenbul	H	H	H	**H**
Balaeniceps rex	Shoebill	H	H	H	**H**
Balearica pavonina	Black Crowned-crane	L	H	H	**L**
Balearica regulorum	Grey Crowned-crane	L	H	H	**L**
Bambusicola fytchii	Mountain Bamboo-partridge	U	L	U	**L**
Bambusicola thoracicus	Chinese Bamboo-partridge	U	L	U	**L**
Bangsia arcaei	Blue-and-gold Tanager	U	U	H	**L**
Bangsia aureocincta	Gold-ringed Tanager	H	H	H	**H**
Bangsia edwardsi	Moss-backed Tanager	H	H	H	**H**
Bangsia melanochlamys	Black-and-gold Tanager	H	U	H	**L**

Bangsia rothschildi	Golden-chested Tanager	H	U	H	**L**
Barnardius zonarius	Ringneck Parrot	H	L	U	**L**
Bartramia longicauda	Upland Sandpiper	L	L	L	**L**
Baryphthengus martii	Rufous Motmot	H	U	L	**L**
Baryphthengus ruficapillus	Rufous-capped Motmot	H	L	L	**L**
Basileuterus basilicus	Santa Marta Warbler	H	U	H	**L**
Basileuterus belli	Golden-browed Warbler	H	H	L	**L**
Basileuterus bivittatus	Two-banded Warbler	H	H	H	**H**
Basileuterus chrysogaster	Golden-bellied Warbler	U	U	H	**L**
Basileuterus cinereicollis	Grey-throated Warbler	U	U	H	**L**
Basileuterus conspicillatus	White-lored Warbler	U	L	H	**L**
Basileuterus coronatus	Russet-crowned Warbler	H	U	L	**L**
Basileuterus culicivorus	Golden-crowned Warbler	L	L	L	**L**
Basileuterus flaveolus	Flavescent Warbler	U	L	L	**L**
Basileuterus fraseri	Grey-and-gold Warbler	U	U	H	**L**
Basileuterus griseiceps	Grey-headed Warbler	H	H	H	**H**
Basileuterus hypoleucus	White-bellied Warbler	H	U	L	**L**
Basileuterus ignotus	Pirre Warbler	H	H	H	**H**
Basileuterus leucoblepharus	White-browed Warbler	H	U	L	**L**
Basileuterus leucophrys	White-striped Warbler	U	U	L	**L**
Basileuterus luteoviridis	Citrine Warbler	H	U	L	**L**
Basileuterus melanogenys	Black-cheeked Warbler	H	H	H	**H**
Basileuterus nigrocristatus	Black-crested Warbler	U	U	H	**L**
Basileuterus rufifrons	Rufous-capped Warbler	L	L	L	**L**
Basileuterus signatus	Pale-legged Warbler	H	U	H	**L**
Basileuterus trifasciatus	Three-banded Warbler	U	U	H	**L**
Basileuterus tristriatus	Three-striped Warbler	U	H	L	**L**
Basilornis celebensis	Sulawesi Myna	U	U	H	**L**
Basilornis corythaix	Long-crested Myna	H	U	H	**L**
Basilornis galeatus	Helmeted Myna	H	U	L	**L**
Basilornis mirandus	Apo Myna	H	H	H	**H**
Batara cinerea	Giant Antshrike	H	L	L	**L**
Bathmocercus cerviniventris	Black-headed Rufous Warbler	H	U	L	**L**
Bathmocercus rufus	Black-faced Rufous Warbler	U	H	H	**L**
Bathmocercus winifredae	Mrs Moreau's Warbler	H	H	H	**H**
Batis capensis	Cape Batis	U	H	L	**L**
Batis crypta	Dark Batis	U	U	L	**L**
Batis diops	Ruwenzori Batis	U	U	L	**L**
Batis fratrum	Zululand Batis	U	L	L	**L**
Batis ituriensis	Ituri Batis	H	U	H	**L**
Batis margaritae	Boulton's Batis	U	H	H	**L**

Batis minima	Gabon Batis	H	U	H	**L**
Batis minor	Black-headed Batis	U	H	L	**L**
Batis minulla	Angola Batis	H	H	H	**H**
Batis mixta	Short-tailed Batis	U	H	U	**L**
Batis molitor	Chinspot Batis	U	H	L	**L**
Batis occulta	West African Batis	H	U	L	**L**
Batis orientalis	Grey-headed Batis	U	L	U	**L**
Batis perkeo	Pygmy Batis	U	U	U	**L**
Batis poensis	Fernando Po Batis	U	U	H	**L**
Batis pririt	Pririt Batis	H	H	H	**H**
Batis senegalensis	Senegal Batis	U	H	U	**L**
Batis soror	Pale Batis	U	H	U	**L**
Batrachostomus affinis	Blyth's Frogmouth	U	H	U	**L**
Batrachostomus auritus	Large Frogmouth	H	H	H	**H**
Batrachostomus cornutus	Sunda Frogmouth	U	H	U	**L**
Batrachostomus harterti	Dulit Frogmouth	H	H	H	**H**
Batrachostomus hodgsoni	Hodgson's Frogmouth	H	H	U	**L**
Batrachostomus javensis	Javan Frogmouth	U	H	L	**L**
Batrachostomus mixtus	Bornean Frogmouth	H	H	H	**H**
Batrachostomus moniliger	Sri Lanka Frogmouth	H	H	U	**L**
Batrachostomus poliolophus	Short-tailed Frogmouth	H	H	H	**H**
Batrachostomus septimus	Philippine Frogmouth	H	H	U	**L**
Batrachostomus stellatus	Gould's Frogmouth	H	H	H	**H**
Berlepschia rikeri	Point-tailed Palmcreeper	H	U	H	**L**
Bernieria apperti	Appert's Tetraka	H	H	L	**L**
Bernieria cinereiceps	Grey-crowned Tetraka	H	H	H	**H**
Bernieria madagascariensis	Common Tetraka	H	H	L	**L**
Bernieria tenebrosa	Dusky Tetraka	H	H	H	**H**
Bernieria zosterops	Spectacled Tetraka	H	H	U	**L**
Bias musicus	Black-and-white Shrike-flycatcher	H	H	L	**L**
Biatas nigropectus	White-bearded Antshrike	H	U	L	**L**
Biziura lobata	Musk Duck	H	H	H	**H**
Bleda canicapillus	Grey-headed Bristlebill	H	H	L	**L**
Bleda eximius	Green-tailed Bristlebill	H	H	L	**L**
Bleda notatus	Lesser Bristlebill	U	H	U	**L**
Bleda syndactylus	Common Bristlebill	H	H	L	**L**
Blythipicus pyrrhotis	Bay Woodpecker	H	L	H	**L**
Blythipicus rubiginosus	Maroon Woodpecker	H	U	U	**L**
Boissonneaua flavescens	Buff-tailed Coronet	H	H	H	**H**
Boissonneaua jardini	Velvet-purple Coronet	H	H	H	**H**
Boissonneaua matthewsii	Chestnut-breasted Coronet	H	H	L	**L**

Bolbopsittacus lunulatus	Guaiabero	U	U	U	**L**
Bolborhynchus ferrugineifrons	Rufous-fronted Parakeet	H	H	H	**H**
Bolborhynchus lineola	Barred Parakeet	H	H	H	**H**
Bolborhynchus orbygnesius	Andean Parakeet	U	H	H	**L**
Bombycilla cedrorum	Cedar Waxwing	H	U	L	**L**
Bombycilla garrulus	Bohemian Waxwing	H	L	H	**L**
Bombycilla japonica	Japanese Waxwing	H	U	H	**L**
Bonasa bonasia	Hazel Grouse	H	L	H	**L**
Bonasa sewerzowi	Chinese Grouse	H	L	H	**L**
Bonasa umbellus	Ruffed Grouse	H	L	L	**L**
Bostrychia bocagei	Dwarf Olive Ibis	H	H	L	**L**
Bostrychia carunculata	Wattled Ibis	H	H	L	**L**
Bostrychia hagedash	Hadada Ibis	L	H	L	**L**
Bostrychia olivacea	African Olive Ibis	H	H	H	**H**
Bostrychia rara	Spot-breasted Ibis	H	H	H	**H**
Botaurus lentiginosus	American Bittern	H	L	H	**L**
Botaurus pinnatus	Pinnated Bittern	H	L	L	**L**
Botaurus poiciloptilus	Australasian Bittern	H	L	H	**L**
Botaurus stellaris	Great Bittern	H	L	H	**L**
Bowdleria punctata	New Zealand Fernbird	U	H	U	**L**
Brachycope anomala	Bob-tailed Weaver	H	U	H	**L**
Brachygalba albogularis	White-throated Jacamar	H	H	L	**L**
Brachygalba goeringi	Pale-headed Jacamar	U	H	L	**L**
Brachygalba lugubris	Brown Jacamar	H	H	H	**H**
Brachygalba salmoni	Dusky-backed Jacamar	H	H	H	**H**
Brachypteracias leptosomus	Short-legged Ground-roller	H	H	H	**H**
Brachypteracias squamiger	Scaly Ground-roller	H	H	H	**H**
Brachypteryx hyperythra	Rusty-bellied Shortwing	H	U	H	**L**
Brachypteryx leucophrys	Lesser Shortwing	U	L	U	**L**
Brachypteryx major	White-bellied Shortwing	H	H	H	**H**
Brachypteryx montana	White-browed Shortwing	U	L	L	**L**
Brachypteryx stellata	Gould's Shortwing	U	U	U	**L**
Brachyramphus brevirostris	Kittlitz's Murrelet	H	H	L	**L**
Brachyramphus marmoratus	Marbled Murrelet	H	H	L	**L**
Brachyramphus perdix	Long-billed Murrelet	H	H	L	**L**
Bradornis infuscatus	Chat Flycatcher	H	L	H	**L**
Bradornis mariquensis	Marico Flycatcher	H	L	H	**L**
Bradornis microrhynchus	African Grey Flycatcher	U	L	U	**L**
Bradornis pallidus	Pale Flycatcher	U	L	U	**L**
Bradypterus accentor	Friendly Bush-warbler	H	H	H	**H**

Bradypterus alfredi	Bamboo Warbler	U	U	U	**L**
Bradypterus alishanensis	Taiwan Bush-warbler	U	U	H	**L**
Bradypterus baboecala	African Bush-warbler	H	L	L	**L**
Bradypterus bangwaensis	Bangwa Forest Warbler	H	L	L	**L**
Bradypterus barratti	African Scrub-warbler	H	H	L	**L**
Bradypterus carpalis	White-winged Scrub-warbler	H	U	H	**L**
Bradypterus castaneus	Chestnut-backed Bush-warbler	H	H	H	**H**
Bradypterus caudatus	Long-tailed Bush-warbler	H	H	H	**H**
Bradypterus cinnamomeus	Bracken Warbler	U	L	U	**L**
Bradypterus davidi	David's Bush-Warbler	U	U	U	**L**
Bradypterus grandis	Dja River Warbler	H	U	H	**L**
Bradypterus graueri	Grauer's Swamp-warbler	H	U	H	**L**
Bradypterus lopezi	Cameroon Scrub-warbler	U	L	U	**L**
Bradypterus luteoventris	Brown Bush-warbler	H	L	U	**L**
Bradypterus major	Long-billed Bush-warbler	H	U	H	**L**
Bradypterus mandelli	Russet Bush-warbler	U	U	U	**L**
Bradypterus montis	Javan Bush-warbler	U	U	U	**L**
Bradypterus palliseri	Sri Lanka Bush-warbler	U	H	H	**L**
Bradypterus seebohmi	Benguet Bush-warbler	H	U	U	**L**
Bradypterus sylvaticus	Knysna Warbler	H	H	L	**L**
Bradypterus tacsanowskius	Chinese Bush-warbler	U	U	U	**L**
Bradypterus thoracicus	Spotted Bush-warbler	U	L	U	**L**
Bradypterus timorensis	Timor Bush-warbler	H	H	H	**H**
Bradypterus victorini	Victorin's Scrub-warbler	H	H	H	**H**
Branta bernicla	Brent Goose	H	H	H	**H**
Branta canadensis	Canada Goose	H	H	L	**L**
Branta hutchinsii	Cackling Goose	U	H	U	**L**
Branta leucopsis	Barnacle Goose	H	H	H	**H**
Branta ruficollis	Red-breasted Goose	H	H	H	**H**
Branta sandvicensis	Hawaiian Goose	H	H	H	**H**
Brotogeris chiriri	Yellow-chevroned Parakeet	U	U	L	**L**
Brotogeris chrysoptera	Golden-winged Parakeet	H	L	H	**L**
Brotogeris cyanoptera	Cobalt-winged Parakeet	H	L	L	**L**
Brotogeris jugularis	Orange-chinned Parakeet	L	L	L	**L**
Brotogeris pyrrhoptera	Grey-cheeked Parakeet	H	L	H	**L**
Brotogeris sanctithomae	Tui Parakeet	H	U	H	**L**
Brotogeris tirica	Plain Parakeet	U	L	H	**L**
Brotogeris versicolurus	White-winged Parakeet	H	L	H	**L**
Bubalornis albirostris	White-billed Buffalo-weaver	U	U	H	**L**
Bubalornis niger	Red-billed Buffalo-weaver	U	U	U	**L**
Bubo africanus	Spotted Eagle-owl	U	H	L	**L**
Bubo ascalaphus	Pharaoh Eagle-owl	U	H	U	**L**

Bubo bengalensis	Rock Eagle-owl	U	H	U	**L**
Bubo bubo	Eurasian Eagle-owl	H	H	H	**H**
Bubo capensis	Cape Eagle-owl	U	H	U	**L**
Bubo coromandus	Dusky Eagle-owl	U	H	U	**L**
Bubo lacteus	Giant Eagle-owl	U	H	L	**L**
Bubo leucostictus	Akun Eagle-owl	H	H	L	**L**
Bubo nipalensis	Spot-bellied Eagle-owl	H	H	U	**L**
Bubo philippensis	Philippine Eagle-owl	H	H	L	**L**
Bubo poensis	Fraser's Eagle-owl	H	H	L	**L**
Bubo scandiaca	Snowy Owl	H	H	H	**H**
Bubo shelleyi	Shelley's Eagle-owl	H	H	L	**L**
Bubo sumatranus	Barred Eagle-owl	U	H	U	**L**
Bubo virginianus	Great Horned Owl	H	H	L	**L**
Bubo vosseleri	Usambara Eagle-owl	H	H	L	**L**
Bubulcus ibis	Cattle Egret	L	H	L	**L**
Bucanetes githagineus	Trumpeter Finch	H	L	H	**L**
Buccanodon duchaillui	Yellow-spotted Barbet	H	H	L	**L**
Bucco capensis	Collared Puffbird	H	U	H	**L**
Bucco macrodactylus	Chestnut-capped Puffbird	H	U	H	**L**
Bucco noanamae	Sooty-capped Puffbird	H	U	H	**L**
Bucco tamatia	Spotted Puffbird	H	H	H	**H**
Bucephala albeola	Bufflehead	H	H	L	**L**
Bucephala clangula	Common Goldeneye	H	H	H	**H**
Bucephala islandica	Barrow's Goldeneye	H	H	H	**H**
Buceros bicornis	Great Hornbill	H	H	L	**L**
Buceros hydrocorax	Rufous Hornbill	H	H	L	**L**
Buceros rhinoceros	Rhinoceros Hornbill	H	H	H	**H**
Bucorvus abyssinicus	Abyssinian Ground-hornbill	H	H	H	**H**
Bucorvus cafer	Southern Ground-hornbill	H	H	L	**L**
Buettikoferella bivittata	Buff-banded Grassbird	U	U	H	**L**
Bulweria bulwerii	Bulwer's Petrel	L	H	L	**L**
Bulweria fallax	Jouanin's Petrel	H	H	L	**L**
Buphagus africanus	Yellow-billed Oxpecker	U	L	H	**L**
Buphagus erythrorhynchus	Red-billed Oxpecker	U	L	U	**L**
Burhinus bistriatus	Double-striped Thick-knee	L	H	H	**L**
Burhinus capensis	Spotted Thick-knee	L	H	L	**L**
Burhinus grallarius	Bush Thick-knee	H	H	L	**L**
Burhinus oedicnemus	Eurasian Thick-knee	L	H	L	**L**
Burhinus senegalensis	Senegal Thick-knee	L	H	H	**L**
Burhinus superciliaris	Peruvian Thick-knee	H	H	H	**H**
Burhinus vermiculatus	Water Thick-knee	L	H	L	**L**
Busarellus nigricollis	Black-collared Hawk	L	H	L	**L**
Butastur indicus	Grey-faced Buzzard	L	H	H	**L**
Butastur liventer	Rufous-winged Buzzard	H	H	L	**L**

Butastur rufipennis	Grasshopper Buzzard	U	H	H	**L**
Butastur teesa	White-eyed Buzzard	L	H	L	**L**
Buteo albicaudatus	White-tailed Hawk	L	H	L	**L**
Buteo albigula	White-throated Hawk	H	H	L	**L**
Buteo albonotatus	Zone-tailed Hawk	L	H	L	**L**
Buteo augur	Augur Buzzard	H	H	U	**L**
Buteo auguralis	Red-necked Buzzard	H	H	H	**H**
Buteo brachypterus	Madagascar Buzzard	H	H	U	**L**
Buteo brachyurus	Short-tailed Hawk	L	H	L	**L**
Buteo buteo	Common Buzzard	H	H	H	**H**
Buteo galapagoensis	Galapagos Hawk	H	H	H	**H**
Buteo hemilasius	Upland Buzzard	H	H	H	**H**
Buteo jamaicensis	Red-tailed Hawk	H	H	L	**L**
Buteo lagopus	Rough-legged Hawk	H	H	H	**H**
Buteo leucorrhous	White-rumped Hawk	U	H	L	**L**
Buteo lineatus	Red-shouldered Hawk	H	H	L	**L**
Buteo magnirostris	Roadside Hawk	L	H	L	**L**
Buteo nitidus	Grey Hawk	L	H	H	**L**
Buteo oreophilus	Mountain Buzzard	H	H	L	**L**
Buteo platypterus	Broad-winged Hawk	L	H	L	**L**
Buteo poecilochrous	Puna Hawk	H	H	U	**L**
Buteo polyosoma	Red-backed Hawk	U	H	L	**L**
Buteo regalis	Ferruginous Hawk	H	H	H	**H**
Buteo ridgwayi	Ridgway's Hawk	H	H	H	**H**
Buteo rufinus	Long-legged Buzzard	H	H	H	**H**
Buteo rufofuscus	Jackal Buzzard	H	H	L	**L**
Buteo solitarius	Hawaiian Hawk	H	H	H	**H**
Buteo swainsoni	Swainson's Hawk	L	H	L	**L**
Buteo ventralis	Rufous-tailed Hawk	H	H	H	**H**
Buteogallus aequinoctialis	Rufous Crab-hawk	H	H	H	**H**
Buteogallus anthracinus	Common Black-hawk	L	H	H	**L**
Buteogallus gundlachii	Cuban Black Hawk	L	H	H	**L**
Buteogallus meridionalis	Savanna Hawk	L	H	L	**L**
Buteogallus subtilis	Mangrove Black-hawk	H	H	U	**L**
Buteogallus urubitinga	Great Black-hawk	L	H	L	**L**
Buthraupis aureodorsalis	Golden-backed Mountain-tanager	H	U	H	**L**
Buthraupis eximia	Black-chested Mountain-tanager	H	H	H	**H**
Buthraupis montana	Hooded Mountain-tanager	H	U	L	**L**
Buthraupis wetmorei	Masked Mountain-tanager	H	U	H	**L**
Butorides striata	Striated Heron	L	L	L	**L**
Butorides virescens	Green Heron	U	L	L	**L**
Bycanistes albotibialis	White-thighed Hornbill	H	H	H	**H**
Bycanistes brevis	Silvery-cheeked Hornbill	H	H	L	**L**

Bycanistes bucinator	Trumpeter Hornbill	H	H	L	**L**
Bycanistes cylindricus	Brown-cheeked Hornbill	H	H	L	**L**
Bycanistes fistulator	Piping Hornbill	H	H	H	**H**
Bycanistes subcylindricus	Black-and-white-casqued Hornbill	H	H	H	**H**
Cacatua alba	White Cockatoo	H	H	H	**H**
Cacatua ducorpsii	Solomons Cockatoo	H	H	H	**H**
Cacatua galerita	Sulphur-crested Cockatoo	H	H	L	**L**
Cacatua goffiniana	Tanimbar Cockatoo	H	H	L	**L**
Cacatua haematuropygia	Philippine Cockatoo	H	H	H	**H**
Cacatua leadbeateri	Major Mitchell's Cockatoo	H	H	U	**L**
Cacatua moluccensis	Salmon-crested Cockatoo	H	H	H	**H**
Cacatua ophthalmica	Blue-eyed Cockatoo	H	H	H	**H**
Cacatua pastinator	Western Corella	H	H	H	**H**
Cacatua roseicapilla	Galah	U	H	L	**L**
Cacatua sanguinea	Little Corella	H	H	L	**L**
Cacatua sulphurea	Yellow-crested Cockatoo	H	H	H	**H**
Cacatua tenuirostris	Long-billed Corella	H	H	L	**L**
Cacicus cela	Yellow-rumped Cacique	H	H	H	**H**
Cacicus chrysonotus	Mountain Cacique	H	H	U	**L**
Cacicus chrysopterus	Golden-winged Cacique	H	L	L	**L**
Cacicus haemorrhous	Red-rumped Cacique	H	H	H	**H**
Cacicus koepckeae	Selva Cacique	H	H	H	**H**
Cacicus melanicterus	Yellow-winged Cacique	L	L	H	**L**
Cacicus sclateri	Ecuadorian Cacique	H	U	H	**L**
Cacicus solitarius	Solitary Black Cacique	H	H	L	**L**
Cacicus uropygialis	Scarlet-rumped Cacique	H	H	H	**H**
Cacomantis castaneiventris	Chestnut-breasted Cuckoo	H	H	H	**H**
Cacomantis flabelliformis	Fan-tailed Cuckoo	U	H	H	**L**
Cacomantis heinrichi	Moluccan Cuckoo	H	H	H	**H**
Cacomantis merulinus	Plaintive Cuckoo	U	U	U	**L**
Cacomantis passerinus	Grey-bellied Cuckoo	U	U	U	**L**
Cacomantis sepulcralis	Rusty-breasted Cuckoo	U	U	L	**L**
Cacomantis sonneratii	Banded Bay Cuckoo	U	U	L	**L**
Cacomantis variolosus	Brush Cuckoo	U	H	L	**L**
Cairina moschata	Muscovy Duck	H	H	L	**L**
Cairina scutulata	White-winged Duck	H	H	L	**L**
Calamanthus campestris	Rufous Fieldwren	U	L	U	**L**
Calamanthus fuliginosus	Striated Fieldwren	U	H	U	**L**
Calamospiza melanocorys	Lark Bunting	H	L	H	**L**
Calandrella acutirostris	Hume's Lark	U	L	U	**L**
Calandrella brachydactyla	Greater Short-toed Lark	H	L	H	**L**
Calandrella cheleensis	Asian Short-toed Lark	H	U	H	**L**
Calandrella cinerea	Red-capped Lark	U	L	U	**L**

Calandrella raytal	Indian Short-toed Lark	U	L	U	**L**
Calandrella rufescens	Lesser Short-toed Lark	H	L	H	**L**
Calandrella somalica	Rufous Short-toed Lark	H	L	U	**L**
Calcarius lapponicus	Lapland Longspur	H	H	H	**H**
Calcarius mccownii	McCown's Longspur	H	L	H	**L**
Calcarius ornatus	Chestnut-collared Longspur	H	L	H	**L**
Calcarius pictus	Smith's Longspur	H	H	L	**L**
Calicalicus madagascariensis	Red-tailed Vanga	U	H	U	**L**
Calicalicus rufocarpalis	Red-shouldered Vanga	H	U	H	**L**
Calidris acuminata	Sharp-tailed Sandpiper	U	H	U	**L**
Calidris alba	Sanderling	L	H	L	**L**
Calidris alpina	Dunlin	H	H	H	**H**
Calidris bairdii	Baird's Sandpiper	U	H	U	**L**
Calidris canutus	Red Knot	L	H	H	**L**
Calidris ferruginea	Curlew Sandpiper	L	H	H	**L**
Calidris fuscicollis	White-rumped Sandpiper	U	H	U	**L**
Calidris himantopus	Stilt Sandpiper	L	H	L	**L**
Calidris maritima	Purple Sandpiper	H	H	H	**H**
Calidris mauri	Western Sandpiper	U	H	U	**L**
Calidris melanotos	Pectoral Sandpiper	U	H	U	**L**
Calidris minuta	Little Stint	L	H	H	**L**
Calidris minutilla	Least Sandpiper	U	H	U	**L**
Calidris ptilocnemis	Rock Sandpiper	U	H	U	**L**
Calidris pusilla	Semipalmated Sandpiper	U	H	U	**L**
Calidris ruficollis	Red-necked Stint	U	H	U	**L**
Calidris subminuta	Long-toed Stint	L	H	L	**L**
Calidris temminckii	Temminck's Stint	L	H	H	**L**
Calidris tenuirostris	Great Knot	L	H	L	**L**
Caliechthrus leucolophus	White-crowned Koel	H	U	L	**L**
Callacanthis burtoni	Spectacled Finch	U	U	H	**L**
Callaeas cinereus	Kokako	H	H	H	**H**
Callipepla californica	California Quail	U	L	U	**L**
Callipepla douglasii	Elegant Quail	L	L	H	**L**
Callipepla gambelii	Gambel's Quail	H	L	H	**L**
Callipepla squamata	Scaled Quail	H	L	H	**L**
Calliphlox amethystina	Amethyst Woodstar	U	H	H	**L**
Calliphlox bryantae	Magenta-throated Woodstar	U	U	H	**L**
Calliphlox evelynae	Bahama Woodstar	H	H	L	**L**
Calliphlox mitchellii	Purple-throated Woodstar	H	H	H	**H**
Callocephalon fimbriatum	Gang-gang Cockatoo	H	H	H	**H**
Callonetta leucophrys	Ringed Teal	H	H	L	**L**
Calochaetes coccineus	Vermilion Tanager	H	U	L	**L**
Calocitta colliei	Black-throated Magpie-jay	L	H	H	**L**
Calocitta formosa	White-throated Magpie-jay	L	H	L	**L**

Caloenas nicobarica	Nicobar Pigeon	H	H	L	**L**
Calonectris diomedea	Cory's Shearwater	L	H	L	**L**
Calonectris edwardsii	Cape Verde Shearwater	L	H	L	**L**
Calonectris leucomelas	Streaked Shearwater	H	H	L	**L**
Caloperdix oculeus	Ferruginous Partridge	H	L	H	**L**
Calorhamphus fuliginosus	Brown Barbet	H	H	U	**L**
Calothorax lucifer	Lucifer Hummingbird	L	H	H	**L**
Calothorax pulcher	Beautiful Hummingbird	L	H	L	**L**
Calypte anna	Anna's Hummingbird	L	H	H	**L**
Calypte costae	Costa's Hummingbird	H	H	H	**H**
Calyptocichla serina	Golden Greenbul	H	U	L	**L**
Calyptomena hosii	Hose's Broadbill	H	L	H	**L**
Calyptomena viridis	Asian Green Broadbill	H	H	H	**H**
Calyptomena whiteheadi	Whitehead's Broadbill	H	H	H	**H**
Calyptophilus frugivorus	Chat Tanager	H	U	H	**L**
Calyptorhynchus banksii	Red-tailed Black-cockatoo	H	H	L	**L**
Calyptorhynchus baudinii	Long-billed Black-cockatoo	H	H	H	**H**
Calyptorhynchus funereus	Yellow-tailed Black-cockatoo	H	H	U	**L**
Calyptorhynchus lathami	Glossy Black-cockatoo	H	H	L	**L**
Calyptorhynchus latirostris	Short-billed Black-cockatoo	H	H	H	**H**
Calyptura cristata	Kinglet Calyptura	H	U	H	**L**
Camarhynchus heliobates	Mangrove Finch	H	H	H	**H**
Camarhynchus pallidus	Woodpecker Finch	H	L	H	**L**
Camarhynchus parvulus	Small Tree-finch	U	L	U	**L**
Camarhynchus pauper	Medium Tree-finch	H	H	L	**L**
Camarhynchus psittacula	Large Tree-finch	H	H	U	**L**
Camaroptera brachyura	Green-backed Camaroptera	U	L	L	**L**
Camaroptera chloronota	Olive-green Camaroptera	H	H	L	**L**
Camaroptera fasciolata	Barred Wren-warbler	H	L	H	**L**
Camaroptera simplex	Grey Wren-warbler	U	L	U	**L**
Camaroptera superciliaris	Yellow-browed Camaroptera	H	L	H	**L**
Camaroptera undosa	Miombo Wren-warbler	H	L	U	**L**
Campephaga flava	Black Cuckooshrike	U	H	L	**L**
Campephaga lobata	Western Wattled Cuckooshrike	H	U	L	**L**
Campephaga oriolina	Eastern Wattled Cuckooshrike	H	U	H	**L**
Campephaga petiti	Petit's Cuckooshrike	U	U	L	**L**
Campephaga phoenicea	Red-shouldered Cuckooshrike	U	H	H	**L**
Campephaga quiscalina	Purple-throated Cuckooshrike	U	H	L	**L**
Campephilus gayaquilensis	Guayaquil Woodpecker	H	U	H	**L**
Campephilus	Pale-billed Woodpecker	H	H	H	**H**

guatemalensis					
Campephilus haematogaster	Crimson-bellied Woodpecker	H	U	H	**L**
Campephilus imperialis	Imperial Woodpecker	H	H	L	**L**
Campephilus leucopogon	Cream-backed Woodpecker	U	U	H	**L**
Campephilus magellanicus	Magellanic Woodpecker	H	H	H	**H**
Campephilus melanoleucos	Crimson-crested Woodpecker	U	L	H	**L**
Campephilus pollens	Powerful Woodpecker	H	U	H	**L**
Campephilus principalis	Ivory-billed Woodpecker	H	H	H	**H**
Campephilus robustus	Robust Woodpecker	H	U	L	**L**
Campephilus rubricollis	Red-necked Woodpecker	H	H	H	**H**
Campethera abingoni	Golden-tailed Woodpecker	U	L	L	**L**
Campethera bennettii	Bennett's Woodpecker	H	L	U	**L**
Campethera cailliautii	Green-backed Woodpecker	U	L	L	**L**
Campethera caroli	Brown-eared Woodpecker	H	L	L	**L**
Campethera maculosa	Little Green Woodpecker	H	U	L	**L**
Campethera mombassica	Mombasa Woodpecker	H	U	L	**L**
Campethera nivosa	Buff-spotted Woodpecker	H	H	L	**L**
Campethera notata	Knysna Woodpecker	H	H	L	**L**
Campethera nubica	Nubian Woodpecker	H	L	U	**L**
Campethera punctuligera	Fine-spotted Woodpecker	U	L	H	**L**
Campethera tullbergi	Tullberg's Woodpecker	H	U	H	**L**
Campochaera sloetii	Golden Cuckooshrike	H	H	H	**H**
Camptostoma imberbe	Northern Beardless-tyrannulet	L	L	H	**L**
Camptostoma obsoletum	Southern Beardless-tyrannulet	H	L	L	**L**
Campylopterus curvipennis	Wedge-tailed Sabrewing	H	U	H	**L**
Campylopterus duidae	Buff-breasted Sabrewing	U	U	H	**L**
Campylopterus ensipennis	White-tailed Sabrewing	H	H	H	**H**
Campylopterus excellens	Long-tailed Sabrewing	H	U	H	**L**
Campylopterus falcatus	Lazuline Sabrewing	U	U	H	**L**
Campylopterus hemileucurus	Violet Sabrewing	H	U	H	**L**
Campylopterus hyperythrus	Rufous-breasted Sabrewing	H	H	H	**H**
Campylopterus largipennis	Grey-breasted Sabrewing	H	H	H	**H**
Campylopterus phainopeplus	Santa Marta Sabrewing	H	U	H	**L**
Campylopterus rufus	Rufous Sabrewing	H	H	H	**H**
Campylopterus villaviscensio	Napo Sabrewing	H	U	H	**L**
Campylorhamphus	Black-billed Scythebill	H	H	L	**L**

falcularius					
Campylorhamphus procurvoides	Curve-billed Scythebill	H	H	H	**H**
Campylorhamphus pucherani	Greater Scythebill	H	H	H	**H**
Campylorhamphus pusillus	Brown-billed Scythebill	H	H	H	**H**
Campylorhamphus trochilirostris	Red-billed Scythebill	H	H	L	**L**
Campylorhynchus albobrunneus	White-headed Wren	H	H	L	**L**
Campylorhynchus brunneicapillus	Cactus Wren	H	L	H	**L**
Campylorhynchus chiapensis	Giant Wren	H	L	H	**L**
Campylorhynchus fasciatus	Fasciated Wren	H	L	H	**L**
Campylorhynchus griseus	Bicoloured Wren	U	L	H	**L**
Campylorhynchus gularis	Spotted Wren	L	L	H	**L**
Campylorhynchus jocosus	Boucard's Wren	L	L	L	**L**
Campylorhynchus megalopterus	Grey-barred Wren	H	H	L	**L**
Campylorhynchus nuchalis	Stripe-backed Wren	H	L	H	**L**
Campylorhynchus rufinucha	Rufous-naped Wren	L	L	L	**L**
Campylorhynchus turdinus	Thrush-like Wren	H	H	L	**L**
Campylorhynchus yucatanicus	Yucatan Wren	H	U	H	**L**
Campylorhynchus zonatus	Band-backed Wren	L	L	H	**L**
Canirallus kioloides	Madagascar Wood Rail	H	H	H	**H**
Canirallus oculeus	Grey-throated Rail	H	H	L	**L**
Capito auratus	Gilded Barbet	H	H	H	**H**
Capito aurovirens	Scarlet-crowned Barbet	H	H	L	**L**
Capito brunneipectus	Brown-chested Barbet	H	H	H	**H**
Capito dayi	Black-girdled Barbet	H	H	H	**H**
Capito hypoleucus	White-mantled Barbet	H	H	H	**H**
Capito maculicoronatus	Spot-crowned Barbet	H	H	L	**L**
Capito niger	Black-spotted Barbet	H	H	H	**H**
Capito quinticolor	Five-coloured Barbet	H	H	H	**H**
Capito squamatus	Orange-fronted Barbet	U	H	H	**L**
Capito wallacei	Scarlet-banded Barbet	H	H	H	**H**
Caprimulgus aegyptius	Egyptian Nightjar	H	H	H	**H**
Caprimulgus affinis	Savanna Nightjar	U	H	U	**L**
Caprimulgus anthonyi	Scrub Nightjar	H	H	H	**H**
Caprimulgus asiaticus	Indian Nightjar	U	U	U	**L**

Caprimulgus atripennis	Jerdon's Nightjar	U	U	H	**L**
Caprimulgus badius	Yucatan Nightjar	H	H	H	**H**
Caprimulgus batesi	Bates's Nightjar	H	H	H	**H**
Caprimulgus binotatus	Brown Nightjar	H	U	H	**L**
Caprimulgus carolinensis	Chuck-will's-widow	L	H	L	**L**
Caprimulgus cayennensis	White-tailed Nightjar	U	H	H	**L**
Caprimulgus celebensis	Sulawesi Nightjar	H	U	H	**L**
Caprimulgus centralasicus	Vaurie's Nightjar	H	U	H	**L**
Caprimulgus clarus	Slender-tailed Nightjar	H	H	L	**L**
Caprimulgus climacurus	Long-tailed Nightjar	U	H	H	**L**
Caprimulgus concretus	Sunda Nightjar	H	H	H	**H**
Caprimulgus cubanensis	Cuban Nightjar	H	H	U	**L**
Caprimulgus donaldsoni	Donaldson-Smith's Nightjar	H	H	L	**L**
Caprimulgus ekmani	Hispaniola Nightjar	H	H	H	**H**
Caprimulgus enarratus	Collared Nightjar	H	H	U	**L**
Caprimulgus europaeus	Eurasian Nightjar	H	H	H	**H**
Caprimulgus eximius	Golden Nightjar	H	H	H	**H**
Caprimulgus fossii	Square-tailed Nightjar	U	H	L	**L**
Caprimulgus fraenatus	Sombre Nightjar	H	H	H	**H**
Caprimulgus heterurus	Todd's Nightjar	U	U	L	**L**
Caprimulgus hirundinaceus	Pygmy Nightjar	H	U	L	**L**
Caprimulgus indicus	Grey Nightjar	U	H	H	**L**
Caprimulgus inornatus	Plain Nightjar	U	H	H	**L**
Caprimulgus longirostris	Band-winged Nightjar	U	H	L	**L**
Caprimulgus macrurus	Large-tailed Nightjar	U	H	U	**L**
Caprimulgus maculicaudus	Spot-tailed Nightjar	L	H	H	**L**
Caprimulgus maculosus	Cayenne Nightjar	H	U	U	**L**
Caprimulgus madagascariensis	Madagascar Nightjar	U	H	U	**L**
Caprimulgus mahrattensis	Sykes's Nightjar	U	U	U	**L**
Caprimulgus manillensis	Philippine Nightjar	H	H	L	**L**
Caprimulgus natalensis	Swamp Nightjar	U	H	L	**L**
Caprimulgus nigrescens	Blackish Nightjar	H	H	H	**H**
Caprimulgus nigriscapularis	Black-shouldered Nightjar	U	H	U	**L**
Caprimulgus noctitherus	Puerto Rican Nightjar	H	H	H	**H**
Caprimulgus nubicus	Nubian Nightjar	H	H	L	**L**
Caprimulgus parvulus	Little Nightjar	U	H	L	**L**
Caprimulgus pectoralis	Fiery-necked Nightjar	U	H	U	**L**
Caprimulgus poliocephalus	Montane Nightjar	U	H	L	**L**
Caprimulgus prigoginei	Itombwe Nightjar	H	U	L	**L**
Caprimulgus pulchellus	Salvadori's Nightjar	H	H	H	**H**
Caprimulgus ridgwayi	Buff-collared Nightjar	L	H	H	**L**

Caprimulgus ruficollis	Red-necked Nightjar	H	H	L	**L**
Caprimulgus rufigena	Rufous-cheeked Nightjar	H	H	L	**L**
Caprimulgus rufus	Rufous Nightjar	U	H	L	**L**
Caprimulgus ruwenzorii	Ruwenzori Nightjar	U	H	L	**L**
Caprimulgus salvini	Tawny-collared Nightjar	U	H	H	**L**
Caprimulgus saturatus	Dusky Nightjar	U	H	H	**L**
Caprimulgus sericocaudatus	Silky-tailed Nightjar	H	U	L	**L**
Caprimulgus solala	Nechisar Nightjar	H	H	H	**H**
Caprimulgus stellatus	Star-spotted Nightjar	H	U	H	**L**
Caprimulgus tristigma	Freckled Nightjar	U	H	L	**L**
Caprimulgus vociferus	Whip-poor-will	H	H	L	**L**
Caprimulgus whitelyi	Roraiman Nightjar	H	H	H	**H**
Capsiempis flaveola	Yellow Tyrannulet	H	H	H	**H**
Caracara cheriway	Crested Caracara	L	H	L	**L**
Caracara plancus	Southern Caracara	U	H	L	**L**
Cardellina rubrifrons	Red-faced Warbler	H	L	L	**L**
Cardinalis cardinalis	Northern Cardinal	H	L	H	**L**
Cardinalis phoeniceus	Vermilion Cardinal	H	U	H	**L**
Cardinalis sinuatus	Pyrrhuloxia	H	L	H	**L**
Carduelis ambigua	Black-headed Greenfinch	U	L	U	**L**
Carduelis atrata	Black Siskin	H	U	H	**L**
Carduelis atriceps	Black-capped Siskin	L	U	H	**L**
Carduelis barbata	Black-chinned Siskin	U	U	U	**L**
Carduelis cannabina	Eurasian Linnet	H	L	H	**L**
Carduelis carduelis	European Goldfinch	H	L	H	**L**
Carduelis chloris	European Greenfinch	H	L	H	**L**
Carduelis citrinella	Alpine Citril Finch	H	L	L	**L**
Carduelis corsicana	Mediterranean Citril Finch	H	U	H	**L**
Carduelis crassirostris	Thick-billed Siskin	H	U	H	**L**
Carduelis cucullata	Red Siskin	H	U	H	**L**
Carduelis dominicensis	Antillean Siskin	H	L	H	**L**
Carduelis flammea	Common Redpoll	H	H	H	**H**
Carduelis flavirostris	Twite	H	L	H	**L**
Carduelis hornemanni	Hoary Redpoll	H	H	H	**H**
Carduelis johannis	Warsangli Linnet	H	H	H	**H**
Carduelis lawrencei	Lawrence's Goldfinch	H	L	H	**L**
Carduelis magellanica	Hooded Siskin	U	U	L	**L**
Carduelis monguilloti	Vietnam Greenfinch	U	H	L	**L**
Carduelis notata	Black-headed Siskin	L	H	L	**L**
Carduelis olivacea	Olivaceous Siskin	U	U	H	**L**
Carduelis pinus	Pine Siskin	H	L	L	**L**
Carduelis psaltria	Lesser Goldfinch	H	L	H	**L**
Carduelis siemiradzkii	Saffron Siskin	H	U	H	**L**
Carduelis sinica	Grey-capped Greenfinch	U	L	U	**L**

Carduelis spinescens	Andean Siskin	U	U	H	**L**
Carduelis spinoides	Yellow-breasted Greenfinch	U	L	U	**L**
Carduelis spinus	Eurasian Siskin	H	L	H	**L**
Carduelis tristis	American Goldfinch	H	L	L	**L**
Carduelis uropygialis	Yellow-rumped Siskin	H	U	H	**L**
Carduelis xanthogastra	Yellow-bellied Siskin	U	L	L	**L**
Carduelis yarrellii	Yellow-faced Siskin	H	U	L	**L**
Carduelis yemenensis	Yemen Linnet	H	H	H	**H**
Cariama cristata	Red-legged Seriema	U	H	L	**L**
Caridonax fulgidus	White-rumped Kingfisher	H	H	H	**H**
Carpococcyx radiatus	Bornean Ground-cuckoo	H	U	H	**L**
Carpococcyx renauldi	Coral-billed Ground-cuckoo	U	L	L	**L**
Carpococcyx viridis	Sumatran Ground-cuckoo	H	U	H	**L**
Carpodacus cassinii	Cassin's Finch	H	L	L	**L**
Carpodacus edwardsii	Dark-rumped Rosefinch	U	U	U	**L**
Carpodacus eos	Pink-rumped Rosefinch	U	U	U	**L**
Carpodacus erythrinus	Common Rosefinch	H	L	H	**L**
Carpodacus mexicanus	House Finch	H	L	H	**L**
Carpodacus nipalensis	Dark-breasted Rosefinch	U	U	U	**L**
Carpodacus pulcherrimus	Beautiful Rosefinch	U	U	U	**L**
Carpodacus puniceus	Red-fronted Rosefinch	U	U	U	**L**
Carpodacus purpureus	Purple Finch	H	L	L	**L**
Carpodacus rhodochlamys	Red-mantled Rosefinch	U	U	U	**L**
Carpodacus roborowskii	Tibetan Rosefinch	H	U	L	**L**
Carpodacus rodochroa	Pink-browed Rosefinch	U	U	U	**L**
Carpodacus rodopeplus	Spot-winged Rosefinch	U	U	U	**L**
Carpodacus roseus	Pallas's Rosefinch	H	U	H	**L**
Carpodacus rubescens	Crimson Rosefinch	H	U	H	**L**
Carpodacus rubicilla	Great Rosefinch	H	L	H	**L**
Carpodacus rubicilloides	Streaked Rosefinch	U	U	U	**L**
Carpodacus synoicus	Pale Rosefinch	U	L	U	**L**
Carpodacus thura	White-browed Rosefinch	U	U	U	**L**
Carpodacus trifasciatus	Three-banded Rosefinch	U	U	U	**L**
Carpodacus vinaceus	Vinaceous Rosefinch	H	U	U	**L**
Carpodectes antoniae	Yellow-billed Cotinga	H	H	H	**H**
Carpodectes hopkei	Black-tipped Cotinga	H	U	H	**L**
Carpodectes nitidus	Snowy Cotinga	H	H	H	**H**
Carpornis cucullata	Hooded Berryeater	H	U	L	**L**
Carpornis melanocephala	Black-headed Berryeater	H	U	H	**L**
Caryothraustes canadensis	Yellow-green Grosbeak	H	H	H	**H**
Caryothraustes poliogaster	Black-faced Grosbeak	L	L	H	**L**
Casiornis fuscus	Ash-throated Casiornis	H	U	L	**L**
Casiornis rufus	Rufous Casiornis	H	U	L	**L**

Casmerodius albus	Great Egret	L	H	L	**L**
Casuarius bennetti	Dwarf Cassowary	H	H	H	**H**
Casuarius casuarius	Southern Cassowary	H	H	L	**L**
Casuarius unappendiculatus	Northern Cassowary	H	H	L	**L**
Catamblyrhynchus diadema	Plushcap	H	H	L	**L**
Catamenia analis	Band-tailed Seedeater	U	U	H	**L**
Catamenia homochroa	Paramo Seedeater	U	U	L	**L**
Catamenia inornata	Plain-coloured Seedeater	U	U	H	**L**
Cataponera turdoides	Sulawesi Thrush	H	H	H	**H**
Catharacta antarctica	Southern Skua	H	H	H	**H**
Catharacta chilensis	Chilean Skua	H	H	H	**H**
Catharacta lonnbergi	Brown Skua	H	H	L	**L**
Catharacta maccormicki	South Polar Skua	H	H	L	**L**
Catharacta skua	Great Skua	L	H	L	**L**
Catharopeza bishopi	Whistling Warbler	H	H	H	**H**
Cathartes aura	Turkey Vulture	L	H	L	**L**
Cathartes burrovianus	Lesser Yellow-headed Vulture	L	H	L	**L**
Cathartes melambrotus	Greater Yellow-headed Vulture	H	H	H	**H**
Catharus aurantiirostris	Orange-billed Nightingale-thrush	L	H	L	**L**
Catharus bicknelli	Bicknell's Thrush	H	H	H	**H**
Catharus dryas	Spotted Nightingale-thrush	H	H	L	**L**
Catharus frantzii	Ruddy-capped Nightingale-thrush	L	H	L	**L**
Catharus fuscater	Slaty-backed Nightingale-thrush	H	H	L	**L**
Catharus fuscescens	Veery	L	L	L	**L**
Catharus gracilirostris	Black-billed Nightingale-thrush	U	H	H	**L**
Catharus guttatus	Hermit Thrush	H	L	L	**L**
Catharus mexicanus	Black-headed Nightingale-thrush	H	H	H	**H**
Catharus minimus	Grey-cheeked Thrush	L	L	L	**L**
Catharus occidentalis	Russet Nightingale-thrush	H	H	L	**L**
Catharus ustulatus	Swainson's Thrush	H	L	L	**L**
Catherpes mexicanus	Canyon Wren	H	L	H	**L**
Catoptrophorus semipalmatus	Willet	L	H	H	**L**
Catreus wallichi	Cheer Pheasant	H	L	H	**L**
Celeus brachyurus	Rufous Woodpecker	H	L	U	**L**
Celeus castaneus	Chestnut-coloured Woodpecker	H	L	H	**L**
Celeus elegans	Chestnut Woodpecker	H	L	H	**L**

Celeus flavescens	Blond-crested Woodpecker	H	U	L	**L**
Celeus flavus	Cream-coloured Woodpecker	H	U	H	**L**
Celeus grammicus	Scaly-breasted Woodpecker	H	U	H	**L**
Celeus loricatus	Cinnamon Woodpecker	H	U	L	**L**
Celeus lugubris	Pale-crested Woodpecker	U	U	L	**L**
Celeus obrieni	Kaempfer's Woodpecker	H	U	L	**L**
Celeus spectabilis	Rufous-headed Woodpecker	H	H	H	**H**
Celeus torquatus	Ringed Woodpecker	H	U	H	**L**
Celeus undatus	Waved Woodpecker	H	U	H	**L**
Centrocercus minimus	Gunnison Sage-grouse	H	U	L	**L**
Centrocercus urophasianus	Greater Sage-grouse	H	L	H	**L**
Centropus andamanensis	Brown Coucal	H	L	L	**L**
Centropus anselli	Gabon Coucal	U	U	U	**L**
Centropus ateralbus	Pied Coucal	H	H	H	**H**
Centropus bengalensis	Lesser Coucal	U	L	U	**L**
Centropus bernsteini	Lesser Black Coucal	H	H	U	**L**
Centropus celebensis	Bay Coucal	U	U	H	**L**
Centropus chalybeus	Biak Coucal	H	U	H	**L**
Centropus chlororhynchus	Green-billed Coucal	H	L	H	**L**
Centropus cupreicaudus	Coppery-tailed Coucal	U	L	U	**L**
Centropus goliath	Goliath Coucal	H	U	H	**L**
Centropus grillii	Black Coucal	U	L	U	**L**
Centropus leucogaster	Black-throated Coucal	U	H	U	**L**
Centropus melanops	Black-faced Coucal	H	U	H	**L**
Centropus menbeki	Greater Black Coucal	U	U	L	**L**
Centropus milo	Buff-headed Coucal	H	U	H	**L**
Centropus monachus	Blue-headed Coucal	U	L	L	**L**
Centropus nigrorufus	Sunda Coucal	H	H	H	**H**
Centropus phasianinus	Pheasant Coucal	U	L	L	**L**
Centropus rectunguis	Short-toed Coucal	H	U	H	**L**
Centropus senegalensis	Senegal Coucal	U	L	H	**L**
Centropus sinensis	Greater Coucal	U	L	U	**L**
Centropus spilopterus	Kai Coucal	H	U	L	**L**
Centropus steerii	Black-hooded Coucal	H	U	L	**L**
Centropus superciliosus	White-browed Coucal	U	L	L	**L**
Centropus toulou	Madagascar Coucal	U	L	U	**L**
Centropus unirufus	Rufous Coucal	H	U	H	**L**
Centropus violaceus	Violaceous Coucal	H	L	H	**L**
Centropus viridis	Philippine Coucal	U	L	U	**L**
Cephalopterus glabricollis	Bare-necked Umbrellabird	H	H	H	**H**
Cephalopterus ornatus	Amazonian Umbrellabird	H	H	H	**H**
Cephalopterus penduliger	Long-wattled Umbrellabird	H	H	H	**H**
Cephalopyrus flammiceps	Fire-capped Tit	H	L	U	**L**

Cepphus carbo	Spectacled Guillemot	H	H	L	**L**
Cepphus columba	Pigeon Guillemot	H	H	L	**L**
Cepphus grylle	Black Guillemot	H	H	H	**H**
Ceratogymna atrata	Black-casqued Hornbill	H	H	L	**L**
Ceratogymna elata	Yellow-casqued Hornbill	H	H	L	**L**
Cercibis oxycerca	Sharp-tailed Ibis	H	H	H	**H**
Cercococcyx mechowi	Dusky Long-tailed Cuckoo	H	U	H	**L**
Cercococcyx montanus	Barred Long-tailed Cuckoo	U	U	L	**L**
Cercococcyx olivinus	Olive Long-tailed Cuckoo	U	U	U	**L**
Cercomacra brasiliana	Rio de Janeiro Antbird	U	H	L	**L**
Cercomacra carbonaria	Rio Branco Antbird	H	H	H	**H**
Cercomacra cinerascens	Grey Antbird	H	H	H	**H**
Cercomacra ferdinandi	Bananal Antbird	H	H	L	**L**
Cercomacra laeta	Laeta Antbird	H	H	H	**H**
Cercomacra manu	Manu Antbird	H	H	H	**H**
Cercomacra melanaria	Mato Grosso Antbird	H	H	L	**L**
Cercomacra nigrescens	Blackish Antbird	H	H	L	**L**
Cercomacra nigricans	Jet Antbird	H	H	L	**L**
Cercomacra parkeri	Parker's Antbird	U	H	H	**L**
Cercomacra serva	Black Antbird	H	H	H	**H**
Cercomacra tyrannina	Dusky Antbird	H	H	H	**H**
Cercomela dubia	Sombre Chat	H	U	L	**L**
Cercomela familiaris	Familiar Chat	U	L	U	**L**
Cercomela fusca	Indian Chat	H	U	U	**L**
Cercomela melanura	Blackstart	U	L	U	**L**
Cercomela schlegelii	Karoo Chat	H	H	L	**L**
Cercomela scotocerca	Brown-tailed Chat	U	U	U	**L**
Cercomela sinuata	Sicklewing Chat	H	H	L	**L**
Cercomela sordida	Moorland Chat	U	L	U	**L**
Cercomela tractrac	Tractrac Chat	H	H	H	**H**
Cercotrichas podobe	Black Scrub-robin	H	L	H	**L**
Cereopsis novaehollandiae	Cape Barren Goose	H	H	U	**L**
Cerorhinca monocerata	Rhinoceros Auklet	H	H	H	**H**
Certhia americana	American Treecreeper	H	L	L	**L**
Certhia brachydactyla	Short-toed Treecreeper	H	L	L	**L**
Certhia discolor	Brown-throated Treecreeper	H	H	U	**L**
Certhia familiaris	Eurasian Treecreeper	H	H	H	**H**
Certhia himalayana	Bar-tailed Treecreeper	H	H	H	**H**
Certhia hodgsoni	Hodgson's Treecreeper	H	H	H	**H**
Certhia manipurensis	Manipur Treecreeper	H	H	H	**H**
Certhia nipalensis	Rusty-flanked Treecreeper	H	H	H	**H**
Certhia tianquanensis	Sichuan Treecreeper	H	H	L	**L**
Certhiaxis cinnamomeus	Yellow-chinned Spinetail	U	L	L	**L**
Certhiaxis mustelinus	Red-and-white Spinetail	H	U	H	**L**

Certhidea olivacea	Warbler Finch	U	L	U	**L**
Certhilauda albescens	Karoo Lark	H	H	L	**L**
Certhilauda barlowi	Barlow's Lark	H	L	H	**L**
Certhilauda benguelensis	Benguela Long-billed Lark	H	U	H	**L**
Certhilauda brevirostris	Agulhas Long-billed Lark	H	L	H	**L**
Certhilauda burra	Red Lark	H	H	L	**L**
Certhilauda chuana	Short-clawed Lark	H	H	H	**H**
Certhilauda curvirostris	Long-billed Lark	H	H	H	**H**
Certhilauda erythrochlamys	Dune Lark	H	H	H	**H**
Certhilauda semitorquata	Eastern Long-billed Lark	H	U	H	**L**
Certhilauda subcoronata	Karoo Long-billed Lark	H	L	H	**L**
Certhionyx niger	Black Honeyeater	U	H	U	**L**
Certhionyx pectoralis	Banded Honeyeater	U	H	L	**L**
Certhionyx variegatus	Pied Honeyeater	U	L	U	**L**
Ceryle rudis	Pied Kingfisher	U	L	L	**L**
Cettia acanthizoides	Yellowish-bellied Bush-warbler	U	L	U	**L**
Cettia annae	Palau Bush-warbler	H	H	H	**H**
Cettia brunnifrons	Grey-sided Bush-warbler	U	L	U	**L**
Cettia canturians	Manchurian Bush-warbler	U	U	U	**L**
Cettia carolinae	Tanimbar Bush-warbler	H	U	L	**L**
Cettia cetti	Cetti's Warbler	H	L	L	**L**
Cettia diphone	Japanese Bush-warbler	U	L	H	**L**
Cettia flavolivacea	Aberrant Bush-warbler	U	L	U	**L**
Cettia fortipes	Brownish-flanked Bush-warbler	U	L	U	**L**
Cettia haddeni	Bougainville Bush-warbler	H	H	H	**H**
Cettia major	Chestnut-crowned Bush-warbler	H	H	U	**L**
Cettia pallidipes	Pale-footed Bush-warbler	U	L	U	**L**
Cettia parens	Shade Warbler	H	H	L	**L**
Cettia ruficapilla	Fiji Bush-warbler	H	H	H	**H**
Cettia seebohmi	Philippine Bush-warbler	H	U	H	**L**
Cettia vulcania	Sunda Bush-warbler	U	U	U	**L**
Ceuthmochares aereus	Yellowbill	U	U	L	**L**
Ceyx erithaca	Black-backed Kingfisher	U	L	L	**L**
Ceyx fallax	Sulawesi Kingfisher	H	U	H	**L**
Ceyx lecontei	African Dwarf-kingfisher	H	L	U	**L**
Ceyx lepidus	Variable Kingfisher	U	H	L	**L**
Ceyx madagascariensis	Madagascar Pygmy-kingfisher	U	L	U	**L**
Ceyx melanurus	Philippine Dwarf-kingfisher	H	U	L	**L**
Ceyx pictus	African Pygmy-kingfisher	U	L	L	**L**
Ceyx rufidorsa	Rufous-backed Kingfisher	H	L	L	**L**
Chaetocercus astreans	Santa Marta Woodstar	H	U	H	**L**

Chaetocercus berlepschi	Esmeraldas Woodstar	H	U	H	**L**
Chaetocercus bombus	Little Woodstar	H	U	H	**L**
Chaetocercus heliodor	Gorgeted Woodstar	U	U	H	**L**
Chaetocercus jourdanii	Rufous-shafted Woodstar	U	U	H	**L**
Chaetocercus mulsant	White-bellied Woodstar	U	U	L	**L**
Chaetops aurantius	Drakensberg Rockjumper	H	H	L	**L**
Chaetops frenatus	Cape Rock-jumper	H	H	H	**H**
Chaetorhynchus papuensis	Pygmy Drongo	H	U	L	**L**
Chaetornis striata	Bristled Grassbird	H	L	H	**L**
Chaetura brachyura	Short-tailed Swift	H	L	H	**L**
Chaetura chapmani	Chapman's Swift	H	H	H	**H**
Chaetura cinereiventris	Grey-rumped Swift	H	L	H	**L**
Chaetura egregia	Pale-rumped Swift	H	U	H	**L**
Chaetura fumosa	Costa Rican Swift	H	U	H	**L**
Chaetura martinica	Lesser Antillean Swift	H	L	H	**L**
Chaetura meridionalis	Sick's Swift	U	L	L	**L**
Chaetura pelagica	Chimney Swift	L	L	L	**L**
Chaetura spinicaudus	Band-rumped Swift	H	U	H	**L**
Chaetura vauxi	Vaux's Swift	L	L	H	**L**
Chaetura viridipennis	Amazonian Swift	H	U	L	**L**
Chaimarrornis leucocephalus	White-capped Water-redstart	U	L	H	**L**
Chalcophaps indica	Emerald Dove	U	H	L	**L**
Chalcophaps stephani	Stephan's Dove	U	H	L	**L**
Chalcopsitta atra	Black Lory	H	H	H	**H**
Chalcopsitta cardinalis	Cardinal Lory	H	H	H	**H**
Chalcopsitta duivenbodei	Brown Lory	H	H	H	**H**
Chalcopsitta sintillata	Yellow-streaked Lory	U	H	U	**L**
Chalcostigma herrani	Rainbow-bearded Thornbill	H	H	H	**H**
Chalcostigma heteropogon	Bronze-tailed Thornbill	U	H	H	**L**
Chalcostigma olivaceum	Olivaceous Thornbill	H	H	H	**H**
Chalcostigma ruficeps	Rufous-capped Thornbill	H	H	H	**H**
Chalcostigma stanleyi	Blue-mantled Thornbill	U	H	H	**L**
Chalybura buffonii	White-vented Plumeleteer	U	U	H	**L**
Chalybura urochrysia	Bronze-tailed Plumeleteer	H	U	H	**L**
Chamaea fasciata	Wrentit	H	L	L	**L**
Chamaepetes goudotii	Sickle-winged Guan	H	U	H	**L**
Chamaepetes unicolor	Black Guan	H	L	H	**L**
Chamaeza campanisona	Short-tailed Antthrush	H	H	L	**L**
Chamaeza meruloides	Such's Antthrush	U	U	L	**L**
Chamaeza mollissima	Barred Antthrush	H	H	L	**L**
Chamaeza nobilis	Striated Antthrush	H	H	L	**L**
Chamaeza ruficauda	Rufous-tailed Antthrush	U	U	L	**L**

Chamaeza turdina	Schwartz's Antthrush	H	U	H	**L**
Charadrius alexandrinus	Kentish Plover	L	L	L	**L**
Charadrius alticola	Puna Plover	H	U	H	**L**
Charadrius asiaticus	Caspian Plover	H	L	L	**L**
Charadrius australis	Inland Dotterel	H	L	L	**L**
Charadrius bicinctus	Double-banded Plover	U	L	U	**L**
Charadrius collaris	Collared Plover	H	H	H	**H**
Charadrius dubius	Little Ringed Plover	L	L	H	**L**
Charadrius falklandicus	Two-banded Plover	L	L	L	**L**
Charadrius forbesi	Forbes's Plover	L	L	H	**L**
Charadrius hiaticula	Common Ringed Plover	H	H	H	**H**
Charadrius javanicus	Javan Plover	H	U	H	**L**
Charadrius leschenaultii	Greater Sand Plover	L	L	L	**L**
Charadrius marginatus	White-fronted Plover	L	H	L	**L**
Charadrius melodus	Piping Plover	H	L	L	**L**
Charadrius modestus	Rufous-chested Plover	L	H	H	**L**
Charadrius mongolus	Lesser Sand Plover	L	L	H	**L**
Charadrius montanus	Mountain Plover	H	H	H	**H**
Charadrius obscurus	New Zealand Dotterel	H	L	H	**L**
Charadrius pallidus	Chestnut-banded Plover	H	H	L	**L**
Charadrius pecuarius	Kittlitz's Plover	L	H	L	**L**
Charadrius peronii	Malaysian Plover	H	L	H	**L**
Charadrius placidus	Long-billed Plover	H	L	U	**L**
Charadrius ruficapillus	Red-capped Plover	U	H	U	**L**
Charadrius sanctaehelenae	St Helena Plover	H	H	U	**L**
Charadrius semipalmatus	Semipalmated Plover	L	H	H	**L**
Charadrius thoracicus	Madagascar Plover	H	H	H	**H**
Charadrius tricollaris	Three-banded Plover	L	H	L	**L**
Charadrius veredus	Oriental Plover	L	U	L	**L**
Charadrius vociferus	Killdeer	H	L	L	**L**
Charadrius wilsonia	Wilson's Plover	H	L	H	**L**
Charitospiza eucosma	Coal-crested Finch	H	U	L	**L**
Charmosyna amabilis	Red-throated Lorikeet	H	H	H	**H**
Charmosyna diadema	New Caledonian Lorikeet	H	U	H	**L**
Charmosyna josefinae	Josephine's Lorikeet	U	U	U	**L**
Charmosyna margarethae	Duchess Lorikeet	H	U	H	**L**
Charmosyna meeki	Meek's Lorikeet	H	U	H	**L**
Charmosyna multistriata	Striated Lorikeet	H	U	H	**L**
Charmosyna palmarum	Palm Lorikeet	H	H	H	**H**
Charmosyna papou	Papuan Lorikeet	U	H	H	**L**
Charmosyna placentis	Red-flanked Lorikeet	U	H	U	**L**
Charmosyna pulchella	Fairy Lorikeet	U	H	U	**L**
Charmosyna rubrigularis	Red-chinned Lorikeet	H	H	H	**H**
Charmosyna rubronotata	Red-fronted Lorikeet	U	U	U	**L**

Charmosyna toxopei	Blue-fronted Lorikeet	H	H	H	**H**
Charmosyna wilhelminae	Pygmy Lorikeet	U	U	U	**L**
Chasiempis sandwichensis	Elepaio	L	U	H	**L**
Chauna chavaria	Northern Screamer	H	L	H	**L**
Chauna torquata	Southern Screamer	H	L	L	**L**
Chelictinia riocourii	African Swallow-tailed Kite	H	H	H	**H**
Chelidoptera tenebrosa	Swallow-wing	U	H	H	**L**
Chen caerulescens	Snow Goose	H	H	L	**L**
Chen canagica	Emperor Goose	H	H	H	**H**
Chen rossii	Ross's Goose	H	H	H	**H**
Chenonetta jubata	Maned Duck	H	H	U	**L**
Cheramoeca leucosterna	White-backed Swallow	H	L	L	**L**
Chersomanes albofasciata	Spike-heeled Lark	H	L	L	**L**
Chersophilus duponti	Dupont's Lark	H	L	L	**L**
Chilia melanura	Crag Chilia	H	L	H	**L**
Chionis albus	Snowy Sheathbill	H	H	H	**H**
Chionis minor	Black-faced Sheathbill	H	H	H	**H**
Chiroxiphia boliviana	Yungas Manakin	H	H	H	**H**
Chiroxiphia caudata	Swallow-tailed Manakin	H	H	L	**L**
Chiroxiphia lanceolata	Lance-tailed Manakin	U	H	L	**L**
Chiroxiphia linearis	Long-tailed Manakin	H	H	H	**H**
Chiroxiphia pareola	Blue-backed Manakin	H	H	H	**H**
Chlamydera cerviniventris	Fawn-breasted Bowerbird	U	H	U	**L**
Chlamydera guttata	Western Bowerbird	U	H	U	**L**
Chlamydera lauterbachi	Yellow-breasted Bowerbird	U	H	U	**L**
Chlamydera maculata	Spotted Bowerbird	H	H	L	**L**
Chlamydera nuchalis	Great Bowerbird	U	H	L	**L**
Chlamydochaera jefferyi	Fruit-hunter	H	H	H	**H**
Chlamydotis undulata	Houbara Bustard	H	H	H	**H**
Chlidonias hybrida	Whiskered Tern	L	H	L	**L**
Chlidonias leucopterus	White-winged Tern	L	H	L	**L**
Chlidonias niger	Black Tern	L	H	H	**L**
Chloephaga hybrida	Kelp Goose	L	H	H	**L**
Chloephaga melanoptera	Andean Goose	H	H	H	**H**
Chloephaga picta	Upland Goose	H	H	L	**L**
Chloephaga poliocephala	Ashy-headed Goose	H	H	L	**L**
Chloephaga rubidiceps	Ruddy-headed Goose	H	H	L	**L**
Chlorestes notata	Blue-chinned Sapphire	H	H	H	**H**
Chloroceryle aenea	American Pygmy Kingfisher	H	L	H	**L**
Chloroceryle amazona	Amazon Kingfisher	L	L	L	**L**
Chloroceryle americana	Green Kingfisher	L	L	L	**L**
Chloroceryle inda	Green-and-rufous Kingfisher	H	L	H	**L**
Chlorocharis emiliae	Mountain Blackeye	H	H	H	**H**
Chlorochrysa calliparaea	Orange-eared Tanager	H	U	U	**L**
Chlorochrysa nitidissima	Multicoloured Tanager	H	H	H	**H**

Chlorochrysa phoenicotis	Glistening-green Tanager	H	H	H	**H**
Chlorocichla falkensteini	Yellow-necked Greenbul	H	H	H	**H**
Chlorocichla flavicollis	Yellow-throated Greenbul	U	H	H	**L**
Chlorocichla flaviventris	Yellow-bellied Greenbul	U	H	L	**L**
Chlorocichla laetissima	Joyful Greenbul	U	U	H	**L**
Chlorocichla prigoginei	Prigogine's Greenbul	H	H	L	**L**
Chlorocichla simplex	Simple Greenbul	H	H	H	**H**
Chloropeta gracilirostris	Papyrus Yellow Warbler	H	L	H	**L**
Chloropeta natalensis	Yellow Flycatcher-warbler	U	H	U	**L**
Chloropeta similis	Mountain Flycatcher-warbler	U	H	U	**L**
Chlorophanes spiza	Green Honeycreeper	L	H	H	**L**
Chlorophonia callophrys	Golden-browed Chlorophonia	H	L	H	**L**
Chlorophonia cyanea	Blue-naped Chlorophonia	U	L	L	**L**
Chlorophonia flavirostris	Yellow-collared Chlorophonia	H	U	H	**L**
Chlorophonia occipitalis	Blue-crowned Chlorophonia	L	L	H	**L**
Chlorophonia pyrrhophrys	Chestnut-breasted Chlorophonia	H	U	H	**L**
Chloropsis aurifrons	Golden-fronted Leafbird	U	L	H	**L**
Chloropsis cochinchinensis	Blue-winged Leafbird	U	L	U	**L**
Chloropsis cyanopogon	Lesser Green Leafbird	H	U	H	**L**
Chloropsis flavipennis	Philippine Leafbird	H	U	H	**L**
Chloropsis hardwickii	Orange-bellied Leafbird	U	L	H	**L**
Chloropsis jerdoni	Jerdon's Leafbird	U	U	L	**L**
Chloropsis kinabaluensis	Bornean Leafbird	H	H	H	**H**
Chloropsis media	Sumatran Leafbird	U	U	H	**L**
Chloropsis palawanensis	Yellow-throated Leafbird	H	H	H	**H**
Chloropsis sonnerati	Greater Green Leafbird	H	U	H	**L**
Chloropsis venusta	Blue-masked Leafbird	U	U	H	**L**
Chlorornis riefferii	Grass-green Tanager	H	H	L	**L**
Chlorospingus canigularis	Ashy-throated Bush-tanager	U	U	U	**L**
Chlorospingus flavigularis	Yellow-throated Bush-tanager	U	U	U	**L**
Chlorospingus flavovirens	Yellow-green Bush-tanager	H	U	H	**L**
Chlorospingus inornatus	Pirre Bush-tanager	U	H	U	**L**
Chlorospingus ophthalmicus	Common Bush-tanager	U	L	U	**L**
Chlorospingus parvirostris	Yellow-whiskered Bush-tanager	U	U	U	**L**
Chlorospingus pileatus	Sooty-capped Bush-tanager	U	H	U	**L**
Chlorospingus semifuscus	Dusky Bush-tanager	H	U	U	**L**
Chlorospingus tacarcunae	Tacarcuna Bush-tanager	H	H	U	**L**
Chlorostilbon alice	Green-tailed Emerald	U	H	H	**L**
Chlorostilbon assimilis	Garden Emerald	H	H	L	**L**
Chlorostilbon auriceps	Golden-crowned Emerald	L	H	H	**L**

Chlorostilbon canivetii	Fork-tailed Emerald	L	H	H	**L**
Chlorostilbon forficatus	Cozumel Emerald	L	H	H	**L**
Chlorostilbon gibsoni	Red-billed Emerald	U	H	H	**L**
Chlorostilbon lucidus	Glittering-bellied Emerald	U	H	L	**L**
Chlorostilbon maugaeus	Puerto Rican Emerald	H	H	H	**H**
Chlorostilbon melanorhynchus	Western Emerald	H	U	H	**L**
Chlorostilbon mellisugus	Blue-tailed Emerald	U	H	L	**L**
Chlorostilbon olivaresi	Chiribiquete Emerald	H	U	H	**L**
Chlorostilbon poortmani	Short-tailed Emerald	H	H	H	**H**
Chlorostilbon ricordii	Cuban Emerald	U	H	H	**L**
Chlorostilbon russatus	Coppery Emerald	U	H	H	**L**
Chlorostilbon stenurus	Narrow-tailed Emerald	U	H	H	**L**
Chlorostilbon swainsonii	Hispaniolan Emerald	H	H	H	**H**
Chlorothraupis carmioli	Olive Tanager	U	H	U	**L**
Chlorothraupis olivacea	Lemon-spectacled Tanager	U	U	U	**L**
Chlorothraupis stolzmanni	Ochre-breasted Tanager	U	H	U	**L**
Chondestes grammacus	Lark Sparrow	H	L	H	**L**
Chondrohierax uncinatus	Hook-billed Kite	H	H	L	**L**
Chondrohierax wilsonii	Cuban Kite	H	H	H	**H**
Chordeiles acutipennis	Lesser Nighthawk	L	H	L	**L**
Chordeiles gundlachii	Antillean Nighthawk	L	H	L	**L**
Chordeiles minor	Common Nighthawk	L	H	L	**L**
Chordeiles pusillus	Least Nighthawk	U	H	L	**L**
Chordeiles rupestris	Sand-coloured Nighthawk	H	H	L	**L**
Chrysococcyx basalis	Horsfield's Bronze-cuckoo	H	H	L	**L**
Chrysococcyx caprius	Didric Cuckoo	U	U	L	**L**
Chrysococcyx crassirostris	Pied Bronze-cuckoo	H	U	L	**L**
Chrysococcyx cupreus	African Emerald Cuckoo	U	U	L	**L**
Chrysococcyx flavigularis	Yellow-throated Cuckoo	H	U	U	**L**
Chrysococcyx klaas	Klaas's Cuckoo	U	U	L	**L**
Chrysococcyx lucidus	Shining Bronze-cuckoo	U	H	U	**L**
Chrysococcyx maculatus	Asian Emerald Cuckoo	U	U	H	**L**
Chrysococcyx meyeri	White-eared Bronze-cuckoo	U	U	L	**L**
Chrysococcyx minutillus	Little Bronze-cuckoo	U	H	U	**L**
Chrysococcyx osculans	Black-eared Cuckoo	U	H	U	**L**
Chrysococcyx ruficollis	Rufous-throated Bronze-cuckoo	H	U	H	**L**
Chrysococcyx russatus	Gould's Bronze-cuckoo	U	H	L	**L**
Chrysococcyx xanthorhynchus	Violet Cuckoo	U	U	U	**L**
Chrysocolaptes festivus	White-naped Woodpecker	U	H	U	**L**
Chrysocolaptes lucidus	Greater Flameback	U	L	L	**L**
Chrysolampis mosquitus	Ruby-topaz Hummingbird	U	H	H	**L**
Chrysolophus amherstiae	Lady Amherst's Pheasant	L	L	H	**L**

Chrysolophus pictus	Golden Pheasant	U	L	H	**L**
Chrysomma altirostre	Jerdon's Babbler	H	L	H	**L**
Chrysomma poecilotis	Rufous-tailed Babbler	U	U	U	**L**
Chrysomma sinense	Yellow-eyed Babbler	U	L	U	**L**
Chrysomus icterocephalus	Yellow-hooded Blackbird	U	L	H	**L**
Chrysomus ruficapillus	Chestnut-capped Blackbird	U	L	L	**L**
Chrysothlypis chrysomelas	Black-and-yellow Tanager	H	U	H	**L**
Chrysothlypis salmoni	Scarlet-and-white Tanager	U	U	H	**L**
Chrysuronia oenone	Golden-tailed Sapphire	U	U	H	**L**
Chthonicola sagittatus	Speckled Warbler	H	H	L	**L**
Chunga burmeisteri	Black-legged Seriema	H	H	L	**L**
Cichladusa arquata	Collared Palm-thrush	U	L	L	**L**
Cichladusa guttata	Spotted Morning-thrush	H	L	L	**L**
Cichladusa ruficauda	Rufous-tailed Palm-thrush	U	L	L	**L**
Cichlherminia lherminieri	Forest Thrush	H	H	H	**H**
Cichlocolaptes leucophrus	Pale-browed Treehunter	H	H	L	**L**
Cichlopsis leucogenys	Rufous-brown Solitaire	H	H	H	**H**
Cicinnurus magnificus	Magnificent Bird-of-paradise	H	H	U	**L**
Cicinnurus regius	King Bird-of-paradise	H	H	L	**L**
Cicinnurus respublica	Wilson's Bird-of-paradise	H	H	H	**H**
Ciconia abdimii	Abdim's Stork	L	H	L	**L**
Ciconia boyciana	Oriental Stork	H	H	H	**H**
Ciconia ciconia	White Stork	H	H	H	**H**
Ciconia episcopus	Woolly-necked Stork	L	H	L	**L**
Ciconia maguari	Maguari Stork	U	H	U	**L**
Ciconia nigra	Black Stork	H	H	H	**H**
Ciconia stormi	Storm's Stork	H	H	H	**H**
Cinclidium diana	Sunda Robin	H	H	H	**H**
Cinclidium frontale	Blue-fronted Robin	U	U	U	**L**
Cinclidium leucurum	White-tailed Robin	U	L	H	**L**
Cinclocerthia gutturalis	Grey Trembler	H	H	H	**H**
Cinclocerthia ruficauda	Brown Trembler	H	H	H	**H**
Cinclodes antarcticus	Blackish Cinclodes	H	H	H	**H**
Cinclodes aricomae	Royal Cinclodes	H	H	H	**H**
Cinclodes atacamensis	White-winged Cinclodes	H	H	H	**H**
Cinclodes comechingonus	Cordoba Cinclodes	H	H	H	**H**
Cinclodes excelsior	Stout-billed Cinclodes	H	H	H	**H**
Cinclodes fuscus	Bar-winged Cinclodes	H	L	L	**L**
Cinclodes nigrofumosus	Seaside Cinclodes	U	L	H	**L**
Cinclodes olrogi	Olrog's Cinclodes	H	H	H	**H**
Cinclodes oustaleti	Grey-flanked Cinclodes	H	L	L	**L**
Cinclodes pabsti	Long-tailed Cinclodes	H	U	H	**L**
Cinclodes palliatus	White-bellied Cinclodes	H	H	H	**H**
Cinclodes patagonicus	Dark-bellied Cinclodes	U	L	H	**L**
Cinclodes taczanowskii	Surf Cinclodes	H	U	H	**L**

Cincloramphus cruralis	Brown Songlark	H	L	L	**L**
Cincloramphus mathewsi	Rufous Songlark	U	L	U	**L**
Cinclosoma ajax	Painted Quail-thrush	H	U	L	**L**
Cinclosoma castaneothorax	Chestnut-breasted Quail-thrush	U	L	U	**L**
Cinclosoma castanotum	Chestnut Quail-thrush	U	H	U	**L**
Cinclosoma cinnamomeum	Cinnamon Quail-thrush	H	L	L	**L**
Cinclosoma punctatum	Spotted Quail-thrush	H	H	L	**L**
Cinclus cinclus	White-throated Dipper	H	L	H	**L**
Cinclus leucocephalus	White-capped Dipper	H	H	L	**L**
Cinclus mexicanus	American Dipper	H	L	H	**L**
Cinclus pallasii	Brown Dipper	H	L	U	**L**
Cinclus schulzi	Rufous-throated Dipper	H	H	H	**H**
Cinnycerthia fulva	Fulvous Wren	H	H	H	**H**
Cinnycerthia olivascens	Sharpe's Wren	H	H	H	**H**
Cinnycerthia peruana	Peruvian Wren	H	H	H	**H**
Cinnycerthia unirufa	Rufous Wren	H	H	H	**H**
Cinnyricinclus femoralis	Abbott's Starling	H	U	H	**L**
Cinnyricinclus leucogaster	Violet-backed Starling	U	L	L	**L**
Cinnyricinclus sharpii	Sharpe's Starling	H	L	H	**L**
Circaetus beaudouini	Beaudouin's Snake-eagle	H	H	H	**H**
Circaetus cinerascens	Banded Snake-eagle	U	H	L	**L**
Circaetus cinereus	Brown Snake-eagle	L	H	L	**L**
Circaetus fasciolatus	Southern Banded Snake-eagle	H	H	L	**L**
Circaetus gallicus	Short-toed Snake-eagle	H	H	L	**L**
Circaetus pectoralis	Black-chested Snake-eagle	U	H	L	**L**
Circus aeruginosus	Western Marsh-harrier	H	H	H	**H**
Circus approximans	Swamp Harrier	L	H	L	**L**
Circus assimilis	Spotted Harrier	H	H	L	**L**
Circus buffoni	Long-winged Harrier	H	H	L	**L**
Circus cinereus	Cinereous Harrier	H	H	U	**L**
Circus cyaneus	Northern Harrier	H	H	H	**H**
Circus macrosceles	Madagascar Harrier	H	H	L	**L**
Circus macrourus	Pallid Harrier	L	H	H	**L**
Circus maillardi	Reunion Harrier	H	H	H	**H**
Circus maurus	Black Harrier	H	H	L	**L**
Circus melanoleucos	Pied Harrier	H	H	H	**H**
Circus pygargus	Montagu's Harrier	H	H	H	**H**
Circus ranivorus	African Marsh-harrier	U	H	L	**L**
Circus spilonotus	Eastern Marsh-harrier	U	H	H	**L**
Cissa chinensis	Green Magpie	U	H	H	**L**
Cissa hypoleuca	Yellow-breasted Magpie	H	H	U	**L**
Cissa thalassina	Short-tailed Magpie	H	H	U	**L**

Cissopis leverianus	Magpie Tanager	U	U	L	**L**
Cisticola aberdare	Aberdare Cisticola	H	H	H	**H**
Cisticola aberrans	Lazy Cisticola	U	L	U	**L**
Cisticola angusticauda	Tabora Cisticola	H	L	U	**L**
Cisticola anonymus	Chattering Cisticola	H	L	H	**L**
Cisticola aridulus	Desert Cisticola	U	L	U	**L**
Cisticola ayresii	Wing-snapping Cisticola	U	L	U	**L**
Cisticola bodessa	Boran Cisticola	H	L	L	**L**
Cisticola brachypterus	Siffling Cisticola	U	L	L	**L**
Cisticola brunnescens	Pectoral-patch Cisticola	U	L	U	**L**
Cisticola bulliens	Bubbling Cisticola	U	L	L	**L**
Cisticola cantans	Singing Cisticola	U	L	H	**L**
Cisticola carruthersi	Carruthers's Cisticola	H	L	H	**L**
Cisticola cherina	Madagascar Cisticola	U	L	U	**L**
Cisticola chiniana	Rattling Cisticola	U	L	U	**L**
Cisticola chubbi	Chubb's Cisticola	U	L	L	**L**
Cisticola cinereolus	Ashy Cisticola	U	L	U	**L**
Cisticola dambo	Black-tailed Cisticola	H	H	U	**L**
Cisticola discolor	Brown-backed Cisticola	U	L	L	**L**
Cisticola erythrops	Red-faced Cisticola	U	L	U	**L**
Cisticola exilis	Golden-headed Cisticola	U	L	L	**L**
Cisticola eximius	Black-necked Cisticola	U	L	U	**L**
Cisticola fulvicapilla	Piping Cisticola	U	L	L	**L**
Cisticola galactotes	Winding Cisticola	U	L	U	**L**
Cisticola guinea	Dorst's Cisticola	U	U	U	**L**
Cisticola haesitatus	Island Cisticola	H	H	H	**H**
Cisticola hunteri	Hunter's Cisticola	U	H	H	**L**
Cisticola juncidis	Zitting Cisticola	U	L	L	**L**
Cisticola lais	Wailing Cisticola	U	H	U	**L**
Cisticola lateralis	Whistling Cisticola	U	L	U	**L**
Cisticola melanurus	Slender-tailed Cisticola	H	U	H	**L**
Cisticola nanus	Tiny Cisticola	U	L	U	**L**
Cisticola natalensis	Croaking Cisticola	U	L	L	**L**
Cisticola nigriloris	Black-lored Cisticola	U	H	L	**L**
Cisticola njombe	Churring Cisticola	U	L	L	**L**
Cisticola pipiens	Chirping Cisticola	U	L	U	**L**
Cisticola restrictus	Tana River Cisticola	H	U	H	**L**
Cisticola robustus	Stout Cisticola	U	L	U	**L**
Cisticola ruficeps	Red-pate Cisticola	U	L	U	**L**
Cisticola rufilatus	Tinkling Cisticola	U	L	L	**L**
Cisticola rufus	Rufous Cisticola	U	U	U	**L**
Cisticola subruficapilla	Grey-backed Cisticola	H	H	L	**L**
Cisticola textrix	Tink-tink Cisticola	U	H	U	**L**
Cisticola tinniens	Levaillant's Cisticola	U	L	U	**L**
Cisticola troglodytes	Foxy Cisticola	U	U	H	**L**

Cisticola woosnami	Trilling Cisticola	U	L	U	**L**
Cistothorus apolinari	Apolinar's Wren	H	U	H	**L**
Cistothorus meridae	Merida Wren	H	U	H	**L**
Cistothorus palustris	Marsh Wren	H	L	L	**L**
Cistothorus platensis	Sedge Wren	H	L	L	**L**
Cittura cyanotis	Lilac-cheeked Kingfisher	H	U	H	**L**
Cladorhynchus leucocephalus	Banded Stilt	H	H	L	**L**
Clamator coromandus	Chestnut-winged Cuckoo	U	U	L	**L**
Clamator glandarius	Great Spotted Cuckoo	L	L	L	**L**
Clamator jacobinus	Pied Cuckoo	U	U	L	**L**
Clamator levaillantii	Levaillant's Cuckoo	U	U	L	**L**
Clangula hyemalis	Long-tailed Duck	H	H	L	**L**
Claravis godefrida	Purple-winged Ground-dove	H	H	L	**L**
Claravis mondetoura	Maroon-chested Ground-dove	H	U	H	**L**
Claravis pretiosa	Blue Ground-dove	L	H	H	**L**
Cleptornis marchei	Golden White-eye	H	H	H	**H**
Clibanornis dendrocolaptoides	Canebrake Groundcreeper	H	U	L	**L**
Climacteris affinis	White-browed Treecreeper	H	H	U	**L**
Climacteris erythrops	Red-browed Treecreeper	H	H	L	**L**
Climacteris melanurus	Black-tailed Treecreeper	H	H	L	**L**
Climacteris picumnus	Brown Treecreeper	H	H	L	**L**
Climacteris rufus	Rufous Treecreeper	H	H	H	**H**
Clypicterus oseryi	Casqued Oropendola	H	H	H	**H**
Clytoceyx rex	Shovel-billed Kookaburra	H	U	H	**L**
Clytoctantes alixii	Recurve-billed Bushbird	H	H	H	**H**
Clytoctantes atrogularis	Rondonia Bushbird	H	H	H	**H**
Clytolaema rubricauda	Brazilian Ruby	U	H	L	**L**
Clytomyias insignis	Orange-crowned Fairywren	H	H	H	**H**
Clytorhynchus hamlini	Rennell Shrikebill	H	H	H	**H**
Clytorhynchus nigrogularis	Black-throated Shrikebill	H	H	L	**L**
Clytorhynchus pachycephaloides	Southern Shrikebill	U	U	L	**L**
Clytorhynchus sanctaecrucis	Santa Cruz Shrikebill	H	H	H	**H**
Clytorhynchus vitiensis	Fiji Shrikebill	H	H	L	**L**
Clytospiza monteiri	Brown Twinspot	U	L	U	**L**
Cnemarchus erythropygius	Red-rumped Bush-tyrant	H	U	H	**L**
Cnemophilus loriae	Loria's Bird-of-paradise	U	H	H	**L**
Cnemophilus macgregorii	Crested Bird-of-paradise	U	H	H	**L**
Cnemoscopus rubrirostris	Grey-hooded Bush-tanager	H	U	H	**L**
Cnemotriccus fuscatus	Fuscous Flycatcher	U	L	H	**L**

Cnipodectes subbrunneus	Brownish Twistwing	H	H	L	**L**
Coccothraustes abeillei	Hooded Grosbeak	L	U	L	**L**
Coccothraustes coccothraustes	Hawfinch	H	L	H	**L**
Coccothraustes vespertinus	Evening Grosbeak	H	L	H	**L**
Coccycolius iris	Emerald Starling	H	L	H	**L**
Coccycua cinerea	Ash-coloured Cuckoo	U	L	L	**L**
Coccycua minuta	Little Cuckoo	H	H	H	**H**
Coccycua pumila	Dwarf Cuckoo	U	L	H	**L**
Coccyzus americanus	Yellow-billed Cuckoo	L	L	L	**L**
Coccyzus erythropthalmus	Black-billed Cuckoo	L	L	L	**L**
Coccyzus euleri	Pearly-breasted Cuckoo	U	H	H	**L**
Coccyzus ferrugineus	Cocos Cuckoo	H	H	U	**L**
Coccyzus lansbergi	Grey-capped Cuckoo	U	L	H	**L**
Coccyzus longirostris	Hispaniolan Lizard-cuckoo	U	L	H	**L**
Coccyzus melacoryphus	Dark-billed Cuckoo	U	L	U	**L**
Coccyzus merlini	Great Lizard-cuckoo	U	L	H	**L**
Coccyzus minor	Mangrove Cuckoo	L	H	H	**L**
Coccyzus pluvialis	Chestnut-bellied Cuckoo	H	L	H	**L**
Coccyzus rufigularis	Bay-breasted Cuckoo	H	H	H	**H**
Coccyzus vetula	Jamaican Lizard-cuckoo	H	U	H	**L**
Coccyzus vieilloti	Puerto Rican Lizard-cuckoo	H	L	H	**L**
Cochlearius cochlearius	Boat-billed Heron	L	H	H	**L**
Cochoa azurea	Javan Cochoa	H	H	H	**H**
Cochoa beccarii	Sumatran Cochoa	H	H	H	**H**
Cochoa purpurea	Purple Cochoa	H	H	U	**L**
Cochoa viridis	Green Cochoa	H	H	H	**H**
Coeligena bonapartei	Golden-bellied Starfrontlet	H	U	H	**L**
Coeligena coeligena	Bronzy Inca	U	H	L	**L**
Coeligena helianthea	Blue-throated Starfrontlet	U	U	H	**L**
Coeligena iris	Rainbow Starfrontlet	H	U	H	**L**
Coeligena lutetiae	Buff-winged Starfrontlet	H	H	H	**H**
Coeligena orina	Dusky Starfrontlet	H	H	H	**H**
Coeligena phalerata	White-tailed Starfrontlet	H	U	H	**L**
Coeligena prunellei	Black Inca	H	U	H	**L**
Coeligena torquata	Collared Inca	H	H	L	**L**
Coeligena violifer	Violet-throated Starfrontlet	H	U	H	**L**
Coeligena wilsoni	Brown Inca	H	H	H	**H**
Coenocorypha aucklandica	New Zealand Snipe	H	H	H	**H**
Coenocorypha pusilla	Chatham Snipe	H	H	U	**L**
Coereba flaveola	Bananaquit	L	U	H	**L**
Colaptes atricollis	Black-necked Woodpecker	H	U	H	**L**
Colaptes auratus	Northern Flicker	H	L	L	**L**

Colaptes auricularis	Grey-crowned Woodpecker	H	U	L	**L**
Colaptes campestris	Campo Flicker	U	L	L	**L**
Colaptes chrysoides	Gilded Flicker	H	L	H	**L**
Colaptes fernandinae	Fernandina's Flicker	H	L	H	**L**
Colaptes melanochloros	Green-barred Woodpecker	H	L	L	**L**
Colaptes pitius	Chilean Flicker	U	L	L	**L**
Colaptes punctigula	Spot-breasted Woodpecker	H	U	L	**L**
Colaptes rivolii	Crimson-mantled Woodpecker	H	U	L	**L**
Colaptes rubiginosus	Golden-olive Woodpecker	L	L	L	**L**
Colaptes rupicola	Andean Flicker	H	L	H	**L**
Colibri coruscans	Sparkling Violet-ear	U	U	L	**L**
Colibri delphinae	Brown Violet-ear	H	U	H	**L**
Colibri serrirostris	White-vented Violet-ear	U	H	L	**L**
Colibri thalassinus	Green Violet-ear	H	H	L	**L**
Colinus cristatus	Crested Bobwhite	L	L	H	**L**
Colinus nigrogularis	Black-throated Bobwhite	L	U	H	**L**
Colinus virginianus	Northern Bobwhite	H	L	L	**L**
Colius castanotus	Red-backed Mousebird	U	L	L	**L**
Colius colius	White-backed Mousebird	H	H	L	**L**
Colius leucocephalus	White-headed Mousebird	U	L	U	**L**
Colius striatus	Speckled Mousebird	U	L	L	**L**
Collocalia amelis	Grey Swiftlet	H	H	L	**L**
Collocalia bartschi	Guam Swiftlet	H	U	H	**L**
Collocalia brevirostris	Himalayan Swiftlet	H	U	U	**L**
Collocalia elaphra	Seychelles Swiftlet	H	H	L	**L**
Collocalia esculenta	Glossy Swiftlet	H	H	U	**L**
Collocalia francica	Mascarene Swiftlet	H	H	H	**H**
Collocalia fuciphaga	Edible-nest Swiftlet	H	U	H	**L**
Collocalia germani	German's Swiftlet	H	H	U	**L**
Collocalia hirundinacea	Mountain Swiftlet	H	H	U	**L**
Collocalia infuscata	Moluccan Swiftlet	H	U	H	**L**
Collocalia inquieta	Micronesian Swiftlet	H	H	U	**L**
Collocalia leucophaea	Tahiti Swiftlet	H	H	U	**L**
Collocalia linchi	Cave Swiftlet	H	U	L	**L**
Collocalia maxima	Black-nest Swiftlet	H	U	U	**L**
Collocalia mearnsi	Philippine Swiftlet	H	U	U	**L**
Collocalia nuditarsus	Bare-legged Swiftlet	H	U	H	**L**
Collocalia ocista	Marquesan Swiftlet	H	H	U	**L**
Collocalia orientalis	Mayr's Swiftlet	H	H	H	**H**
Collocalia palawanensis	Palawan Swiftlet	H	H	H	**H**
Collocalia papuensis	Papuan Swiftlet	H	U	H	**L**
Collocalia pelewensis	Palau Swiftlet	H	H	H	**H**
Collocalia rogersi	Indochinese Swiftlet	H	H	U	**L**
Collocalia salangana	Mossy-nest Swiftlet	H	H	L	**L**

Collocalia sawtelli	Atiu Swiftlet	H	H	U	**L**
Collocalia spodiopygia	White-rumped Swiftlet	H	H	H	**H**
Collocalia troglodytes	Pygmy Swiftlet	H	U	L	**L**
Collocalia unicolor	Indian Swiftlet	H	U	U	**L**
Collocalia vanikorensis	Uniform Swiftlet	H	H	L	**L**
Collocalia vulcanorum	Volcano Swiftlet	H	H	H	**H**
Collocalia whiteheadi	Whitehead's Swiftlet	H	H	H	**H**
Colluricincla boweri	Bower's Shrike-thrush	U	H	H	**L**
Colluricincla harmonica	Grey Shrike-thrush	H	H	L	**L**
Colluricincla megarhyncha	Little Shrike-thrush	H	H	U	**L**
Colluricincla sanghirensis	Sangihe Shrike-thrush	H	H	H	**H**
Colluricincla tenebrosa	Morningbird	H	H	H	**H**
Colluricincla umbrina	Sooty Shrike-thrush	H	H	H	**H**
Colluricincla woodwardi	Sandstone Shrike-thrush	U	H	U	**L**
Colonia colonus	Long-tailed Tyrant	H	L	L	**L**
Colorhamphus parvirostris	Patagonian Tyrant	U	L	H	**L**
Columba albinucha	White-naped Pigeon	H	H	H	**H**
Columba albitorques	White-collared Pigeon	U	H	U	**L**
Columba argentina	Silvery Wood-pigeon	H	H	L	**L**
Columba arquatrix	African Olive-pigeon	U	H	L	**L**
Columba bollii	Dark-tailed Laurel Pigeon	H	H	H	**H**
Columba delegorguei	Eastern Bronze-naped Pigeon	U	H	L	**L**
Columba elphinstonii	Nilgiri Wood-pigeon	H	H	H	**H**
Columba eversmanni	Pale-backed Pigeon	H	H	H	**H**
Columba guinea	Speckled Pigeon	U	H	H	**L**
Columba hodgsonii	Speckled Wood-pigeon	U	H	H	**L**
Columba iriditorques	Western Bronze-naped Pigeon	H	H	H	**H**
Columba janthina	Japanese Wood-pigeon	H	H	H	**H**
Columba junoniae	White-tailed Laurel Pigeon	H	H	H	**H**
Columba leucomela	White-headed Pigeon	H	H	L	**L**
Columba leuconota	Snow Pigeon	H	H	H	**H**
Columba livia	Rock Pigeon	H	H	H	**H**
Columba malherbii	Sao Tome Bronze-naped Pigeon	H	U	L	**L**
Columba oenas	Stock Dove	H	H	H	**H**
Columba oliviae	Somali Pigeon	H	H	L	**L**
Columba pallidiceps	Yellow-legged Pigeon	H	U	L	**L**
Columba palumboides	Andaman Wood-pigeon	H	U	L	**L**
Columba palumbus	Common Wood-pigeon	H	H	H	**H**
Columba pollenii	Comoro Olive-pigeon	H	H	H	**H**
Columba pulchricollis	Ashy Wood-pigeon	U	H	H	**L**
Columba punicea	Pale-capped Pigeon	H	H	H	**H**
Columba rupestris	Hill Pigeon	U	H	U	**L**

Columba sjostedti	Cameroon Olive-pigeon	H	H	L	**L**
Columba thomensis	Maroon Pigeon	H	H	H	**H**
Columba torringtoniae	Sri Lanka Wood-pigeon	H	H	H	**H**
Columba trocaz	Madeira Laurel Pigeon	H	H	H	**H**
Columba unicincta	Afep Pigeon	H	H	L	**L**
Columba vitiensis	White-throated Pigeon	U	H	L	**L**
Columbina buckleyi	Ecuadorian Ground-dove	H	U	H	**L**
Columbina cruziana	Croaking Ground-dove	U	H	H	**L**
Columbina cyanopis	Blue-eyed Ground-dove	H	U	L	**L**
Columbina inca	Inca Dove	L	H	H	**L**
Columbina minuta	Plain-breasted Ground-dove	L	H	L	**L**
Columbina passerina	Common Ground-dove	L	H	L	**L**
Columbina picui	Picui Ground-dove	U	H	L	**L**
Columbina squammata	Scaled Dove	U	H	L	**L**
Columbina talpacoti	Ruddy Ground-dove	U	H	L	**L**
Compsothraupis loricata	Scarlet-throated Tanager	U	U	L	**L**
Conioptilon mcilhennyi	Black-faced Cotinga	H	U	H	**L**
Conirostrum albifrons	Capped Conebill	H	U	L	**L**
Conirostrum bicolor	Bicoloured Conebill	H	H	H	**H**
Conirostrum cinereum	Cinereous Conebill	H	U	H	**L**
Conirostrum ferrugineiventre	White-browed Conebill	U	U	H	**L**
Conirostrum leucogenys	White-eared Conebill	U	U	L	**L**
Conirostrum margaritae	Pearly-breasted Conebill	H	H	H	**H**
Conirostrum rufum	Rufous-browed Conebill	H	U	H	**L**
Conirostrum sitticolor	Blue-backed Conebill	H	U	H	**L**
Conirostrum speciosum	Chestnut-vented Conebill	U	U	L	**L**
Conirostrum tamarugense	Tamarugo Conebill	H	U	H	**L**
Conopias albovittatus	White-ringed Flycatcher	U	H	U	**L**
Conopias cinchoneti	Lemon-browed Flycatcher	U	U	H	**L**
Conopias parvus	Yellow-throated Flycatcher	U	H	U	**L**
Conopias trivirgatus	Three-striped Flycatcher	H	H	L	**L**
Conopophaga ardesiaca	Slaty Gnateater	U	U	H	**L**
Conopophaga aurita	Chestnut-belted Gnateater	H	H	H	**H**
Conopophaga castaneiceps	Chestnut-crowned Gnateater	H	U	H	**L**
Conopophaga lineata	Rufous Gnateater	U	H	L	**L**
Conopophaga melanogaster	Black-bellied Gnateater	H	U	H	**L**
Conopophaga melanops	Black-cheeked Gnateater	U	H	H	**L**
Conopophaga peruviana	Ash-throated Gnateater	H	H	H	**H**
Conopophaga roberti	Hooded Gnateater	H	H	L	**L**
Conopophila albogularis	Rufous-banded Honeyeater	U	H	L	**L**
Conopophila rufogularis	Rufous-throated Honeyeater	U	L	U	**L**
Conopophila whitei	Grey Honeyeater	U	H	U	**L**

Conostoma oemodium	Great Parrotbill	U	L	U	**L**
Conothraupis mesoleuca	Cone-billed Tanager	H	H	H	**H**
Conothraupis speculigera	Black-and-white Tanager	U	U	H	**L**
Contopus albogularis	White-throated Pewee	H	H	H	**H**
Contopus caribaeus	Greater Antillean Pewee	U	L	L	**L**
Contopus cinereus	Tropical Pewee	L	L	L	**L**
Contopus cooperi	Olive-sided Flycatcher	H	L	L	**L**
Contopus fumigatus	Smoke-coloured Pewee	U	U	L	**L**
Contopus hispaniolensis	Hispaniolan Pewee	U	L	H	**L**
Contopus latirostris	Lesser Antillean Pewee	H	H	H	**H**
Contopus lugubris	Dark Pewee	U	U	H	**L**
Contopus nigrescens	Blackish Pewee	H	H	H	**H**
Contopus ochraceus	Ochraceous Pewee	U	U	H	**L**
Contopus pallidus	Jamaican Pewee	H	H	H	**H**
Contopus pertinax	Greater Pewee	H	L	L	**L**
Contopus sordidulus	Western Wood-pewee	L	L	L	**L**
Contopus virens	Eastern Wood-pewee	L	L	L	**L**
Copsychus albospecularis	Madagascar Magpie-robin	U	L	U	**L**
Copsychus cebuensis	Black Shama	H	L	H	**L**
Copsychus luzoniensis	White-browed Shama	H	H	U	**L**
Copsychus malabaricus	White-rumped Shama	U	L	U	**L**
Copsychus niger	White-vented Shama	H	U	H	**L**
Copsychus saularis	Oriental Magpie-robin	U	L	L	**L**
Copsychus sechellarum	Seychelles Magpie-robin	H	H	L	**L**
Coracias abyssinicus	Abyssinian Roller	U	L	H	**L**
Coracias benghalensis	Indian Roller	U	L	H	**L**
Coracias caudatus	Lilac-breasted Roller	H	L	L	**L**
Coracias cyanogaster	Blue-bellied Roller	H	U	U	**L**
Coracias garrulus	European Roller	H	L	H	**L**
Coracias naevia	Rufous-crowned Roller	U	L	U	**L**
Coracias spatulatus	Racket-tailed Roller	H	L	L	**L**
Coracias temminckii	Purple-winged Roller	H	L	H	**L**
Coracina abbotti	Pygmy Cuckooshrike	H	H	H	**H**
Coracina analis	New Caledonian Cuckooshrike	H	H	H	**H**
Coracina atriceps	Moluccan Cuckooshrike	H	U	H	**L**
Coracina azurea	Blue Cuckooshrike	H	U	L	**L**
Coracina bicolor	Pied Cuckooshrike	H	H	H	**H**
Coracina boyeri	Boyer's Cuckooshrike	H	H	L	**L**
Coracina caeruleogrisea	Stout-billed Cuckooshrike	U	H	L	**L**
Coracina caesia	Grey Cuckooshrike	U	H	L	**L**
Coracina caledonica	Melanesian Cuckooshrike	U	U	L	**L**
Coracina ceramensis	Pale Cicadabird	H	U	H	**L**
Coracina cinerea	Ashy Cuckooshrike	U	H	U	**L**
Coracina coerulescens	Blackish Cuckooshrike	U	U	L	**L**

Coracina dispar	Kai Cicadabird	H	H	L	**L**
Coracina dohertyi	Sumba Cicadabird	H	H	H	**H**
Coracina fimbriata	Lesser Cuckooshrike	H	H	H	**H**
Coracina fortis	Buru Cuckooshrike	U	U	H	**L**
Coracina graueri	Grauer's Cuckooshrike	H	H	H	**H**
Coracina holopolia	Solomons Cuckooshrike	H	H	H	**H**
Coracina incerta	Black-shouldered Cicadabird	U	U	L	**L**
Coracina javensis	Javan Cuckooshrike	U	H	H	**L**
Coracina larvata	Sunda Cuckooshrike	H	H	H	**H**
Coracina leucopygia	White-rumped Cuckooshrike	U	U	U	**L**
Coracina lineata	Yellow-eyed Cuckooshrike	U	H	L	**L**
Coracina longicauda	Hooded Cuckooshrike	H	H	H	**H**
Coracina macei	Large Cuckooshrike	U	L	H	**L**
Coracina maxima	Ground Cuckooshrike	U	L	U	**L**
Coracina mcgregori	McGregor's Cuckooshrike	H	H	H	**H**
Coracina melanoptera	Black-headed Cuckooshrike	U	L	L	**L**
Coracina melas	New Guinea Cuckooshrike	H	U	L	**L**
Coracina melaschistos	Black-winged Cuckooshrike	U	L	H	**L**
Coracina mindanensis	Black-bibbed Cicadabird	H	U	L	**L**
Coracina montana	Black-bellied Cuckooshrike	U	U	H	**L**
Coracina morio	Sulawesi Cicadabird	U	U	H	**L**
Coracina newtoni	Reunion Cuckooshrike	H	H	H	**H**
Coracina novaehollandiae	Black-faced Cuckooshrike	U	L	L	**L**
Coracina ostenta	White-winged Cuckooshrike	H	H	H	**H**
Coracina papuensis	White-bellied Cuckooshrike	U	L	U	**L**
Coracina parvula	Halmahera Cuckooshrike	H	H	H	**H**
Coracina pectoralis	White-breasted Cuckooshrike	U	H	U	**L**
Coracina personata	Wallacean Cuckooshrike	H	U	L	**L**
Coracina polioptera	Indochinese Cuckooshrike	U	L	L	**L**
Coracina schistacea	Slaty Cuckooshrike	H	U	L	**L**
Coracina schisticeps	Grey-headed Cuckooshrike	H	H	L	**L**
Coracina striata	Bar-bellied Cuckooshrike	H	U	L	**L**
Coracina sula	Sula Cicadabird	H	U	L	**L**
Coracina temminckii	Cerulean Cuckooshrike	U	U	H	**L**
Coracina tenuirostris	Slender-billed Cicadabird	U	H	L	**L**
Coracina typica	Mauritius Cuckooshrike	H	H	H	**H**
Coracopsis nigra	Black Parrot	H	H	U	**L**
Coracopsis vasa	Vasa Parrot	H	H	L	**L**
Coracornis raveni	Maroon-backed Whistler	H	H	H	**H**
Coragyps atratus	Black Vulture	L	H	L	**L**
Corapipo altera	White-ruffed Manakin	H	H	H	**H**
Corapipo gutturalis	White-throated Manakin	H	H	H	**H**
Corapipo leucorrhoa	White-bibbed Manakin	H	H	H	**H**
Corcorax	White-winged Chough	H	H	L	**L**

melanorhamphos					
Cormobates leucophaea	White-throated Treecreeper	H	H	L	**L**
Cormobates placens	Papuan Treecreeper	H	H	H	**H**
Corvinella corvina	Yellow-billed Shrike	U	L	H	**L**
Corvus albicollis	White-necked Raven	U	H	L	**L**
Corvus albus	Pied Crow	U	H	L	**L**
Corvus bennetti	Little Crow	U	H	U	**L**
Corvus brachyrhynchos	American Crow	H	H	L	**L**
Corvus capensis	Cape Crow	U	H	U	**L**
Corvus caurinus	North-western Crow	L	H	H	**L**
Corvus corax	Common Raven	H	H	H	**H**
Corvus corone	Carrion Crow	H	H	H	**H**
Corvus coronoides	Australian Raven	U	H	U	**L**
Corvus crassirostris	Thick-billed Raven	U	H	L	**L**
Corvus cryptoleucus	Chihuahuan Raven	H	H	H	**H**
Corvus dauuricus	Daurian Jackdaw	U	H	H	**L**
Corvus enca	Slender-billed Crow	U	H	U	**L**
Corvus florensis	Flores Crow	H	H	H	**H**
Corvus frugilegus	Rook	H	H	H	**H**
Corvus fuscicapillus	Brown-headed Crow	H	H	H	**H**
Corvus hawaiiensis	Hawaiian Crow	H	H	U	**L**
Corvus imparatus	Tamaulipas Crow	L	H	H	**L**
Corvus jamaicensis	Jamaican Crow	H	H	H	**H**
Corvus kubaryi	Mariana Crow	H	H	H	**H**
Corvus leucognaphalus	White-necked Crow	H	H	H	**H**
Corvus levaillantii	Jungle Crow	U	H	U	**L**
Corvus macrorhynchos	Large-billed Crow	U	H	H	**L**
Corvus meeki	Bougainville Crow	H	H	H	**H**
Corvus mellori	Little Raven	U	H	U	**L**
Corvus monedula	Eurasian Jackdaw	H	H	H	**H**
Corvus moneduloides	New Caledonian Crow	U	H	H	**L**
Corvus nasicus	Cuban Crow	U	H	H	**L**
Corvus orru	Torresian Crow	U	H	U	**L**
Corvus ossifragus	Fish Crow	H	H	L	**L**
Corvus palmarum	Palm Crow	U	H	H	**L**
Corvus rhipidurus	Fan-tailed Raven	U	H	U	**L**
Corvus ruficollis	Brown-necked Raven	H	H	H	**H**
Corvus sinaloae	Sinaloa Crow	L	H	H	**L**
Corvus splendens	House Crow	U	H	L	**L**
Corvus tasmanicus	Forest Raven	U	H	U	**L**
Corvus torquatus	Collared Crow	L	H	H	**L**
Corvus tristis	Grey Crow	U	H	U	**L**
Corvus typicus	Piping Crow	U	H	U	**L**
Corvus unicolor	Banggai Crow	H	H	H	**H**
Corvus validus	Long-billed Crow	H	H	H	**H**

Corvus woodfordi	White-billed Crow	H	H	H	**H**
Corydon sumatranus	Dusky Broadbill	U	L	L	**L**
Coryphaspiza melanotis	Black-masked Finch	H	U	L	**L**
Coryphistera alaudina	Lark-like Brushrunner	H	L	L	**L**
Coryphospingus cucullatus	Red-crested Finch	U	U	L	**L**
Coryphospingus pileatus	Pileated Finch	U	U	L	**L**
Corythaeola cristata	Great Blue Turaco	H	H	H	**H**
Corythaixoides concolor	Grey Go-away-bird	U	L	L	**L**
Corythaixoides leucogaster	White-bellied Go-away-bird	H	L	U	**L**
Corythaixoides personatus	Bare-faced Go-away-bird	U	L	U	**L**
Corythopis delalandi	Southern Antpipit	H	H	L	**L**
Corythopis torquatus	Ringed Antpipit	H	H	H	**H**
Coscoroba coscoroba	Coscoroba Swan	H	H	L	**L**
Cosmopsarus regius	Golden-breasted Starling	U	L	U	**L**
Cosmopsarus unicolor	Ashy Starling	U	L	U	**L**
Cossypha albicapilla	White-crowned Robin-chat	U	H	U	**L**
Cossypha anomala	Olive-flanked Robin-chat	U	H	L	**L**
Cossypha archeri	Archer's Robin-chat	H	H	L	**L**
Cossypha caffra	Cape Robin-chat	H	L	L	**L**
Cossypha cyanocampter	Blue-shouldered Robin-chat	U	H	L	**L**
Cossypha dichroa	Chorister Robin-chat	H	H	L	**L**
Cossypha heinrichi	White-headed Robin-chat	H	H	H	**H**
Cossypha heuglini	White-browed Robin-chat	U	H	L	**L**
Cossypha humeralis	White-throated Robin-chat	H	L	L	**L**
Cossypha isabellae	Mountain Robin-chat	H	H	L	**L**
Cossypha natalensis	Red-capped Robin-chat	U	L	L	**L**
Cossypha niveicapilla	Snowy-crowned Robin-chat	U	L	H	**L**
Cossypha polioptera	Grey-winged Robin-chat	H	U	H	**L**
Cossypha semirufa	Rueppell's Robin-chat	U	L	L	**L**
Cossyphicula roberti	White-bellied Robin-chat	H	H	L	**L**
Cotinga amabilis	Lovely Cotinga	H	U	H	**L**
Cotinga cayana	Spangled Cotinga	H	U	H	**L**
Cotinga cotinga	Purple-breasted Cotinga	H	U	H	**L**
Cotinga maculata	Banded Cotinga	H	U	H	**L**
Cotinga maynana	Plum-throated Cotinga	H	U	L	**L**
Cotinga nattererii	Blue Cotinga	H	H	H	**H**
Cotinga ridgwayi	Turquoise Cotinga	H	H	H	**H**
Coturnicops exquisitus	Swinhoe's Rail	H	L	H	**L**
Coturnicops notatus	Speckled Rail	H	U	L	**L**
Coturnicops noveboracensis	Yellow Rail	H	L	H	**L**
Coturnix chinensis	Blue Quail	U	L	U	**L**

Coturnix coromandelica	Rain Quail	U	L	U	**L**
Coturnix coturnix	Common Quail	H	L	H	**L**
Coturnix delegorguei	Harlequin Quail	U	L	U	**L**
Coturnix japonica	Japanese Quail	U	L	U	**L**
Coturnix pectoralis	Stubble Quail	U	L	U	**L**
Coturnix ypsilophora	Brown Quail	U	L	U	**L**
Coua caerulea	Blue Coua	U	H	U	**L**
Coua coquereli	Coquerel's Coua	H	H	L	**L**
Coua cristata	Crested Coua	U	H	U	**L**
Coua cursor	Running Coua	H	H	L	**L**
Coua gigas	Giant Coua	U	L	L	**L**
Coua reynaudii	Red-fronted Coua	U	H	U	**L**
Coua ruficeps	Red-capped Coua	U	H	U	**L**
Coua serriana	Red-breasted Coua	H	H	H	**H**
Coua verreauxi	Verreaux's Coua	H	U	L	**L**
Cracticus cassicus	Hooded Butcherbird	U	H	L	**L**
Cracticus louisiadensis	Tagula Butcherbird	H	H	H	**H**
Cracticus mentalis	Black-backed Butcherbird	U	H	U	**L**
Cracticus nigrogularis	Pied Butcherbird	H	H	L	**L**
Cracticus quoyi	Black Butcherbird	U	H	U	**L**
Cracticus torquatus	Grey Butcherbird	U	H	U	**L**
Cranioleuca albicapilla	Creamy-crested Spinetail	U	U	H	**L**
Cranioleuca albiceps	Light-crowned Spinetail	H	H	H	**H**
Cranioleuca antisiensis	Line-cheeked Spinetail	H	U	H	**L**
Cranioleuca baroni	Baron's Spinetail	H	H	H	**H**
Cranioleuca curtata	Ash-browed Spinetail	H	H	L	**L**
Cranioleuca demissa	Tepui Spinetail	H	H	H	**H**
Cranioleuca erythrops	Red-faced Spinetail	H	H	H	**H**
Cranioleuca gutturata	Speckled Spinetail	H	H	H	**H**
Cranioleuca hellmayri	Streak-capped Spinetail	H	H	H	**H**
Cranioleuca henricae	Bolivian Spinetail	H	U	H	**L**
Cranioleuca marcapatae	Marcapata Spinetail	H	H	H	**H**
Cranioleuca muelleri	Scaled Spinetail	H	H	H	**H**
Cranioleuca obsoleta	Olive Spinetail	H	U	L	**L**
Cranioleuca pallida	Pallid Spinetail	H	H	L	**L**
Cranioleuca pyrrhophia	Stripe-crowned Spinetail	H	L	L	**L**
Cranioleuca semicinerea	Grey-headed Spinetail	H	H	L	**L**
Cranioleuca subcristata	Crested Spinetail	U	U	H	**L**
Cranioleuca sulphurifera	Sulphur-throated Spinetail	H	L	H	**L**
Cranioleuca vulpecula	Parker's Spinetail	H	U	H	**L**
Cranioleuca vulpina	Rusty-backed Spinetail	H	L	L	**L**
Crateroscelis murina	Rusty Mouse-warbler	H	H	L	**L**
Crateroscelis nigrorufa	Bicoloured Mouse-warbler	H	H	H	**H**
Crateroscelis robusta	Mountain Mouse-warbler	H	U	H	**L**
Crax alberti	Blue-billed Curassow	H	H	H	**H**

Crax alector	Black Curassow	H	H	H	**H**
Crax blumenbachii	Red-billed Curassow	H	H	H	**H**
Crax daubentoni	Yellow-knobbed Curassow	H	H	H	**H**
Crax fasciolata	Bare-faced Curassow	H	H	L	**L**
Crax globulosa	Wattled Curassow	H	H	L	**L**
Crax rubra	Great Curassow	H	H	H	**H**
Creagrus furcatus	Swallow-tailed Gull	L	H	H	**L**
Creatophora cinerea	Wattled Starling	H	L	L	**L**
Crecopsis egregia	African Crake	U	L	L	**L**
Creurgops dentatus	Slaty Tanager	H	H	H	**H**
Creurgops verticalis	Rufous-crested Tanager	H	H	H	**H**
Crex crex	Corncrake	H	L	H	**L**
Crinifer piscator	Western Grey Plantain-eater	U	L	U	**L**
Crinifer zonurus	Eastern Grey Plantain-eater	U	L	U	**L**
Criniger barbatus	Bearded Bulbul	H	U	L	**L**
Criniger calurus	Red-tailed Bulbul	H	H	L	**L**
Criniger chloronotus	Green-backed Bulbul	H	H	H	**H**
Criniger ndussumensis	White-bearded Bulbul	H	H	H	**H**
Criniger olivaceus	Yellow-bearded Greenbul	H	U	L	**L**
Crocias albonotatus	Spotted Crocias	H	H	H	**H**
Crocias langbianis	Grey-crowned Crocias	H	H	H	**H**
Crossleyia xanthophrys	Madagascar Yellowbrow	H	H	H	**H**
Crossoptilon auritum	Blue Eared-pheasant	H	L	U	**L**
Crossoptilon crossoptilon	White Eared-pheasant	H	L	H	**L**
Crossoptilon harmani	Tibetan Eared-pheasant	H	L	H	**L**
Crossoptilon mantchuricum	Brown Eared-pheasant	H	L	L	**L**
Crotophaga ani	Smooth-billed Ani	L	U	L	**L**
Crotophaga major	Greater Ani	U	L	H	**L**
Crotophaga sulcirostris	Groove-billed Ani	L	L	L	**L**
Crypsirina cucullata	Hooded Treepie	U	H	H	**L**
Crypsirina temia	Racket-tailed Treepie	U	H	U	**L**
Cryptophaps poecilorrhoa	Sombre Pigeon	H	H	H	**H**
Cryptospiza jacksoni	Dusky Crimson-wing	U	U	L	**L**
Cryptospiza reichenovii	Red-faced Crimson-wing	H	U	U	**L**
Cryptospiza salvadorii	Abyssinian Crimson-wing	U	L	U	**L**
Cryptospiza shelleyi	Shelley's Crimson-wing	H	U	U	**L**
Cryptosylvicola randrianasoloi	Cryptic Warbler	H	H	H	**H**
Crypturellus atrocapillus	Black-capped Tinamou	H	H	H	**H**
Crypturellus bartletti	Bartlett's Tinamou	H	H	L	**L**
Crypturellus berlepschi	Berlepsch's Tinamou	H	H	H	**H**
Crypturellus boucardi	Slaty-breasted Tinamou	H	H	H	**H**
Crypturellus brevirostris	Rusty Tinamou	H	H	H	**H**
Crypturellus casiquiare	Barred Tinamou	H	H	H	**H**

Crypturellus cinereus	Cinereous Tinamou	H	H	H	**H**
Crypturellus cinnamomeus	Thicket Tinamou	L	H	H	**L**
Crypturellus duidae	Grey-legged Tinamou	H	H	H	**H**
Crypturellus erythropus	Red-legged Tinamou	H	H	H	**H**
Crypturellus kerriae	Choco Tinamou	H	H	L	**L**
Crypturellus noctivagus	Yellow-legged Tinamou	U	H	L	**L**
Crypturellus obsoletus	Brown Tinamou	H	H	H	**H**
Crypturellus parvirostris	Small-billed Tinamou	U	H	L	**L**
Crypturellus ptaritepui	Tepui Tinamou	H	H	H	**H**
Crypturellus soui	Little Tinamou	L	H	H	**L**
Crypturellus strigulosus	Brazilian Tinamou	H	H	L	**L**
Crypturellus tataupa	Tataupa Tinamou	H	H	L	**L**
Crypturellus transfasciatus	Pale-browed Tinamou	H	H	H	**H**
Crypturellus undulatus	Undulated Tinamou	H	H	H	**H**
Crypturellus variegatus	Variegated Tinamou	H	H	H	**H**
Cuculus canorus	Common Cuckoo	H	H	H	**H**
Cuculus clamosus	Black Cuckoo	U	H	L	**L**
Cuculus crassirostris	Sulawesi Hawk-cuckoo	H	H	H	**H**
Cuculus fugax	Hodgson's Hawk-cuckoo	U	H	L	**L**
Cuculus gularis	African Cuckoo	U	H	U	**L**
Cuculus lepidus	Sunda Cuckoo	H	H	L	**L**
Cuculus micropterus	Indian Cuckoo	U	H	H	**L**
Cuculus optatus	Oriental Cuckoo	U	H	H	**L**
Cuculus pallidus	Pallid Cuckoo	U	H	L	**L**
Cuculus poliocephalus	Lesser Cuckoo	U	H	L	**L**
Cuculus rochii	Madagascar Cuckoo	U	H	U	**L**
Cuculus saturatus	Himalayan Cuckoo	U	H	U	**L**
Cuculus solitarius	Red-chested Cuckoo	U	H	L	**L**
Cuculus sparverioides	Large Hawk-cuckoo	U	H	L	**L**
Cuculus vagans	Moustached Hawk-cuckoo	H	H	H	**H**
Cuculus varius	Common Hawk-cuckoo	U	H	L	**L**
Culicicapa ceylonensis	Grey-headed Canary-flycatcher	U	L	U	**L**
Culicicapa helianthea	Citrine Canary-flycatcher	U	U	U	**L**
Culicivora caudacuta	Sharp-tailed Tyrant	L	U	L	**L**
Curaeus curaeus	Austral Blackbird	U	L	H	**L**
Curaeus forbesi	Forbes's Blackbird	H	H	H	**H**
Cursorius coromandelicus	Indian Courser	U	H	U	**L**
Cursorius cursor	Cream-coloured Courser	H	H	H	**H**
Cursorius rufus	Burchell's Courser	H	H	H	**H**
Cursorius temminckii	Temminck's Courser	U	H	U	**L**
Cutia legalleni	Vietnamese Cutia	H	H	L	**L**
Cutia nipalensis	Himalayan Cutia	H	H	U	**L**

Cyanerpes caeruleus	Purple Honeycreeper	H	U	H	**L**
Cyanerpes cyaneus	Red-legged Honeycreeper	H	H	H	**H**
Cyanerpes lucidus	Shining Honeycreeper	L	H	H	**L**
Cyanerpes nitidus	Short-billed Honeycreeper	H	U	H	**L**
Cyanicterus cyanicterus	Blue-backed Tanager	H	U	H	**L**
Cyanochen cyanoptera	Blue-winged Goose	H	H	H	**H**
Cyanocitta cristata	Blue Jay	H	H	L	**L**
Cyanocitta stelleri	Steller's Jay	H	H	H	**H**
Cyanocompsa brissonii	Ultramarine Grosbeak	U	U	L	**L**
Cyanocompsa cyanoides	Blue-black Grosbeak	H	H	H	**H**
Cyanocompsa parellina	Blue Bunting	L	H	H	**L**
Cyanocorax affinis	Black-chested Jay	U	H	H	**L**
Cyanocorax beecheii	Purplish-backed Jay	L	H	H	**L**
Cyanocorax caeruleus	Azure Jay	H	H	L	**L**
Cyanocorax cayanus	Cayenne Jay	H	H	H	**H**
Cyanocorax chrysops	Plush-crested Jay	U	H	L	**L**
Cyanocorax cristatellus	Curl-crested Jay	U	H	L	**L**
Cyanocorax cyanomelas	Purplish Jay	U	H	L	**L**
Cyanocorax cyanopogon	White-naped Jay	H	H	L	**L**
Cyanocorax dickeyi	Tufted Jay	H	H	L	**L**
Cyanocorax heilprini	Azure-naped Jay	H	H	H	**H**
Cyanocorax melanocyaneus	Bushy-crested Jay	U	H	H	**L**
Cyanocorax morio	Brown Jay	L	H	H	**L**
Cyanocorax mystacalis	White-tailed Jay	U	H	H	**L**
Cyanocorax sanblasianus	San Blas Jay	L	H	H	**L**
Cyanocorax violaceus	Violaceous Jay	H	H	H	**H**
Cyanocorax yncas	Green Jay	L	H	L	**L**
Cyanocorax yucatanicus	Yucatan Jay	L	H	H	**L**
Cyanolanius madagascarinus	Blue Vanga	U	U	L	**L**
Cyanolimnas cerverai	Zapata Rail	H	L	H	**L**
Cyanoliseus patagonus	Burrowing Parakeet	H	H	H	**H**
Cyanoloxia glaucocaerulea	Glaucous-blue Grosbeak	H	U	L	**L**
Cyanolyca argentigula	Silvery-throated Jay	H	H	H	**H**
Cyanolyca armillata	Black-collared Jay	H	H	H	**H**
Cyanolyca cucullata	Azure-hooded Jay	H	H	H	**H**
Cyanolyca mirabilis	White-throated Jay	H	H	L	**L**
Cyanolyca nana	Dwarf Jay	H	H	H	**H**
Cyanolyca pulchra	Beautiful Jay	H	H	H	**H**
Cyanolyca pumilo	Black-throated Jay	H	H	H	**H**
Cyanolyca turcosa	Turquoise Jay	U	H	H	**L**
Cyanolyca viridicyanus	White-collared Jay	U	H	H	**L**
Cyanophaia bicolor	Blue-headed Hummingbird	H	H	H	**H**

Cyanopica cyanus	Azure-winged Magpie	H	H	H	**H**
Cyanopsitta spixii	Spix's Macaw	H	H	H	**H**
Cyanoptila cyanomelana	Blue-and-white Flycatcher	U	L	H	**L**
Cyanoramphus auriceps	Yellow-crowned Parakeet	H	L	H	**L**
Cyanoramphus cookii	Norfolk Island Parakeet	H	H	H	**H**
Cyanoramphus forbesi	Chatham Parakeet	H	H	U	**L**
Cyanoramphus malherbi	Malherbe's Parakeet	H	L	H	**L**
Cyanoramphus novaezelandiae	Red-fronted Parakeet	L	L	H	**L**
Cyanoramphus saisetti	New Caledonian Parakeet	U	L	H	**L**
Cyanoramphus unicolor	Antipodes Parakeet	H	H	U	**L**
Cyclarhis gujanensis	Rufous-browed Peppershrike	L	L	L	**L**
Cyclarhis nigrirostris	Black-billed Peppershrike	H	H	H	**H**
Cyclopsitta diophthalma	Double-eyed Fig-parrot	U	L	L	**L**
Cyclopsitta gulielmitertii	Orange-breasted Fig-parrot	U	U	U	**L**
Cygnus atratus	Black Swan	L	H	L	**L**
Cygnus buccinator	Trumpeter Swan	L	H	H	**L**
Cygnus columbianus	Tundra Swan	H	H	H	**H**
Cygnus cygnus	Whooper Swan	H	H	H	**H**
Cygnus melancoryphus	Black-necked Swan	L	H	L	**L**
Cygnus olor	Mute Swan	H	H	H	**H**
Cymbilaimus lineatus	Fasciated Antshrike	H	H	H	**H**
Cymbilaimus sanctaemariae	Bamboo Antshrike	H	U	L	**L**
Cymbirhynchus macrorhynchos	Black-and-red Broadbill	H	L	H	**L**
Cynanthus latirostris	Broad-billed Hummingbird	L	H	H	**L**
Cynanthus sordidus	Dusky Hummingbird	L	H	L	**L**
Cyornis banyumas	Hill Blue-flycatcher	U	L	U	**L**
Cyornis caerulatus	Large-billed Blue-flycatcher	H	H	H	**H**
Cyornis concretus	White-tailed Flycatcher	U	H	U	**L**
Cyornis hainanus	Hainan Blue-flycatcher	H	H	U	**L**
Cyornis herioti	Blue-breasted Flycatcher	U	H	H	**L**
Cyornis hoevelli	Blue-fronted Flycatcher	H	H	H	**H**
Cyornis hyacinthinus	Timor Blue-flycatcher	H	U	L	**L**
Cyornis lemprieri	Palawan Blue-flycatcher	H	H	H	**H**
Cyornis omissus	Sulawesi Blue-flycatcher	U	U	U	**L**
Cyornis pallipes	White-bellied Blue-flycatcher	U	L	H	**L**
Cyornis poliogenys	Pale-chinned Blue-flycatcher	U	L	U	**L**
Cyornis rubeculoides	Blue-throated Flycatcher	U	L	U	**L**
Cyornis ruckii	Rueck's Blue-flycatcher	H	U	H	**L**
Cyornis rufigastra	Mangrove Blue-flycatcher	U	H	U	**L**
Cyornis sanfordi	Matinan Flycatcher	H	H	H	**H**
Cyornis superbus	Bornean Blue-flycatcher	H	H	U	**L**
Cyornis tickelliae	Tickell's Blue-flycatcher	U	L	U	**L**

Cyornis turcosus	Malaysian Blue-flycatcher	H	U	H	**L**
Cyornis unicolor	Pale Blue-flycatcher	U	L	U	**L**
Cyphorhinus arada	Musician Wren	H	H	H	**H**
Cyphorhinus phaeocephalus	Song Wren	H	H	L	**L**
Cyphorhinus thoracicus	Chestnut-breasted Wren	H	H	H	**H**
Cypseloides cherriei	Spot-fronted Swift	H	H	H	**H**
Cypseloides cryptus	White-chinned Swift	H	H	H	**H**
Cypseloides fumigatus	Sooty Swift	H	H	L	**L**
Cypseloides lemosi	White-chested Swift	H	H	H	**H**
Cypseloides niger	American Black Swift	H	H	L	**L**
Cypseloides rothschildi	Rothschild's Swift	H	H	H	**H**
Cypseloides senex	Great Dusky Swift	H	H	L	**L**
Cypseloides storeri	White-fronted Swift	H	H	L	**L**
Cypsiurus balasiensis	Asian Palm-swift	U	H	U	**L**
Cypsiurus parvus	African Palm-swift	U	H	L	**L**
Cypsnagra hirundinacea	White-rumped Tanager	U	L	L	**L**
Cyrtonyx montezumae	Montezuma Quail	H	L	L	**L**
Cyrtonyx ocellatus	Ocellated Quail	H	U	H	**L**
Dacelo gaudichaud	Rufous-bellied Kookaburra	H	H	L	**L**
Dacelo leachii	Blue-winged Kookaburra	U	L	L	**L**
Dacelo novaeguineae	Laughing Kookaburra	H	L	L	**L**
Dacelo tyro	Spangled Kookaburra	H	U	L	**L**
Dacnis albiventris	White-bellied Dacnis	H	U	H	**L**
Dacnis berlepschi	Scarlet-breasted Dacnis	H	U	H	**L**
Dacnis cayana	Blue Dacnis	U	L	H	**L**
Dacnis flaviventer	Yellow-bellied Dacnis	H	U	H	**L**
Dacnis hartlaubi	Turquoise Dacnis	H	H	H	**H**
Dacnis lineata	Black-faced Dacnis	H	U	H	**L**
Dacnis nigripes	Black-legged Dacnis	H	U	L	**L**
Dacnis venusta	Scarlet-thighed Dacnis	U	H	L	**L**
Dacnis viguieri	Viridian Dacnis	H	U	L	**L**
Dactylortyx thoracicus	Singing Quail	H	L	H	**L**
Damophila julie	Violet-bellied Hummingbird	H	H	L	**L**
Daphoenositta chrysoptera	Varied Sittella	U	L	L	**L**
Daphoenositta miranda	Black Sittella	U	U	H	**L**
Daption capense	Cape Petrel	H	H	L	**L**
Daptrius ater	Black Caracara	H	H	H	**H**
Dasycrotapha speciosa	Flame-templed Babbler	H	H	H	**H**
Dasyornis brachypterus	Eastern Bristlebird	H	H	H	**H**
Dasyornis broadbenti	Rufous Bristlebird	H	H	L	**L**
Dasyornis longirostris	Western Bristlebird	H	H	H	**H**
Deconychura longicauda	Long-tailed Woodcreeper	H	H	H	**H**
Deconychura stictolaema	Spot-throated Woodcreeper	H	U	H	**L**

Delichon dasypus	Asian House-martin	U	L	U	**L**
Delichon nipalense	Nepal House-martin	U	L	U	**L**
Delichon urbicum	Northern House-martin	H	L	H	**L**
Delothraupis castaneoventris	Chestnut-bellied Mountain-tanager	H	U	H	**L**
Deltarhynchus flammulatus	Flammulated Flycatcher	L	L	H	**L**
Dendragapus canadensis	Spruce Grouse	H	L	L	**L**
Dendragapus falcipennis	Siberian Grouse	H	L	H	**L**
Dendragapus fuliginosus	Sooty Grouse	U	U	H	**L**
Dendragapus obscurus	Dusky Grouse	H	L	H	**L**
Dendrexetastes rufigula	Cinnamon-throated Woodcreeper	H	L	H	**L**
Dendrocincla anabatina	Tawny-winged Woodcreeper	H	H	H	**H**
Dendrocincla fuliginosa	Plain-brown Woodcreeper	H	H	H	**H**
Dendrocincla homochroa	Ruddy Woodcreeper	H	L	H	**L**
Dendrocincla merula	White-chinned Woodcreeper	H	U	H	**L**
Dendrocincla tyrannina	Tyrannine Woodcreeper	H	H	H	**H**
Dendrocitta bayleyi	Andaman Treepie	H	H	L	**L**
Dendrocitta formosae	Grey Treepie	U	H	U	**L**
Dendrocitta frontalis	Collared Treepie	H	H	U	**L**
Dendrocitta leucogastra	White-bellied Treepie	U	H	H	**L**
Dendrocitta occipitalis	Sunda Treepie	U	H	U	**L**
Dendrocitta vagabunda	Rufous Treepie	U	H	U	**L**
Dendrocolaptes certhia	Amazonian Barred Woodcreeper	H	H	H	**H**
Dendrocolaptes hoffmannsi	Hoffmanns's Woodcreeper	H	H	H	**H**
Dendrocolaptes picumnus	Black-banded Woodcreeper	H	H	H	**H**
Dendrocolaptes platyrostris	Planalto Woodcreeper	H	U	L	**L**
Dendrocolaptes sanctithomae	Northern Barred Woodcreeper	H	H	H	**H**
Dendrocopos assimilis	Sind Woodpecker	U	L	U	**L**
Dendrocopos atratus	Stripe-breasted Woodpecker	H	L	U	**L**
Dendrocopos auriceps	Brown-fronted Woodpecker	H	L	U	**L**
Dendrocopos canicapillus	Grey-capped Woodpecker	U	L	U	**L**
Dendrocopos cathpharius	Crimson-breasted Woodpecker	H	L	U	**L**
Dendrocopos darjellensis	Darjeeling Woodpecker	H	L	U	**L**
Dendrocopos dorae	Arabian Woodpecker	H	U	H	**L**
Dendrocopos himalayensis	Himalayan Woodpecker	H	L	U	**L**
Dendrocopos hyperythrus	Rufous-bellied Woodpecker	H	L	U	**L**
Dendrocopos kizuki	Pygmy Woodpecker	H	L	H	**L**
Dendrocopos leucopterus	White-winged Woodpecker	H	L	H	**L**
Dendrocopos leucotos	White-backed Woodpecker	H	L	U	**L**

Dendrocopos macei	Fulvous-breasted Woodpecker	U	L	U	**L**
Dendrocopos maculatus	Philippine Woodpecker	U	U	U	**L**
Dendrocopos mahrattensis	Yellow-crowned Woodpecker	H	L	U	**L**
Dendrocopos major	Great Spotted Woodpecker	H	L	H	**L**
Dendrocopos medius	Middle Spotted Woodpecker	H	L	L	**L**
Dendrocopos minor	Lesser Spotted Woodpecker	H	L	H	**L**
Dendrocopos moluccensis	Sunda Woodpecker	H	L	U	**L**
Dendrocopos nanus	Brown-capped Woodpecker	H	L	U	**L**
Dendrocopos noguchii	Okinawa Woodpecker	H	H	H	**H**
Dendrocopos obsoletus	Brown-backed Woodpecker	U	H	H	**L**
Dendrocopos ramsayi	Sulu Woodpecker	H	U	L	**L**
Dendrocopos syriacus	Syrian Woodpecker	U	L	U	**L**
Dendrocopos temminckii	Sulawesi Woodpecker	H	U	U	**L**
Dendrocygna arborea	West Indian Whistling-duck	H	L	L	**L**
Dendrocygna arcuata	Wandering Whistling-duck	U	L	U	**L**
Dendrocygna autumnalis	Black-bellied Whistling-duck	L	L	L	**L**
Dendrocygna bicolor	Fulvous Whistling-duck	L	L	L	**L**
Dendrocygna eytoni	Plumed Whistling-duck	U	L	U	**L**
Dendrocygna guttata	Spotted Whistling-duck	H	L	U	**L**
Dendrocygna javanica	Lesser Whistling-duck	H	L	U	**L**
Dendrocygna viduata	White-faced Whistling-duck	H	L	L	**L**
Dendroica adelaidae	Adelaide's Warbler	H	L	H	**L**
Dendroica angelae	Elfin-woods Warbler	H	H	H	**H**
Dendroica caerulescens	Black-throated Blue Warbler	H	L	L	**L**
Dendroica castanea	Bay-breasted Warbler	H	L	L	**L**
Dendroica cerulea	Cerulean Warbler	L	L	L	**L**
Dendroica chrysoparia	Golden-cheeked Warbler	L	L	H	**L**
Dendroica coronata	Yellow-rumped Warbler	H	L	L	**L**
Dendroica delicata	St Lucia Warbler	H	H	H	**H**
Dendroica discolor	Prairie Warbler	H	L	L	**L**
Dendroica dominica	Yellow-throated Warbler	L	L	H	**L**
Dendroica fusca	Blackburnian Warbler	L	L	L	**L**
Dendroica graciae	Grace's Warbler	H	L	L	**L**
Dendroica kirtlandii	Kirtland's Warbler	H	L	L	**L**
Dendroica magnolia	Magnolia Warbler	H	L	L	**L**
Dendroica nigrescens	Black-throated Grey Warbler	H	L	H	**L**
Dendroica occidentalis	Hermit Warbler	L	L	L	**L**
Dendroica palmarum	Palm Warbler	H	L	H	**L**
Dendroica pensylvanica	Chestnut-sided Warbler	H	L	L	**L**
Dendroica petechia	Yellow Warbler	L	L	L	**L**
Dendroica pharetra	Arrowhead Warbler	H	H	H	**H**
Dendroica pinus	Pine Warbler	H	L	L	**L**
Dendroica pityophila	Olive-capped Warbler	U	H	L	**L**

Dendroica plumbea	Plumbeous Warbler	H	L	H	**L**
Dendroica striata	Blackpoll Warbler	L	L	L	**L**
Dendroica subita	Barbuda Warbler	H	H	H	**H**
Dendroica tigrina	Cape May Warbler	H	L	L	**L**
Dendroica townsendi	Townsend's Warbler	H	L	H	**L**
Dendroica virens	Black-throated Green Warbler	L	L	L	**L**
Dendroica vitellina	Vitelline Warbler	H	H	L	**L**
Dendronanthus indicus	Forest Wagtail	U	L	U	**L**
Dendropicos abyssinicus	Abyssinian Woodpecker	U	U	L	**L**
Dendropicos elachus	Sahelian Woodpecker	H	U	U	**L**
Dendropicos fuscescens	Cardinal Woodpecker	H	L	L	**L**
Dendropicos gabonensis	Gabon Woodpecker	H	U	U	**L**
Dendropicos poecilolaemus	Speckle-breasted Woodpecker	H	U	H	**L**
Dendropicos stierlingi	Stierling's Woodpecker	H	H	L	**L**
Dendrortyx barbatus	Bearded Wood-partridge	H	L	H	**L**
Dendrortyx leucophrys	Buffy-crowned Wood-partridge	H	L	H	**L**
Dendrortyx macroura	Long-tailed Wood-partridge	H	L	L	**L**
Deroptyus accipitrinus	Red-fan Parrot	H	H	H	**H**
Dicaeum aeneum	Midget Flowerpecker	H	U	H	**L**
Dicaeum aeruginosum	Striped Flowerpecker	U	U	L	**L**
Dicaeum agile	Thick-billed Flowerpecker	U	L	L	**L**
Dicaeum annae	Golden-rumped Flowerpecker	H	U	H	**L**
Dicaeum anthonyi	Flame-crowned Flowerpecker	H	H	L	**L**
Dicaeum aureolimbatum	Yellow-sided Flowerpecker	U	U	U	**L**
Dicaeum australe	Red-striped Flowerpecker	U	U	U	**L**
Dicaeum bicolor	Bicoloured Flowerpecker	H	U	L	**L**
Dicaeum celebicum	Grey-sided Flowerpecker	U	L	H	**L**
Dicaeum chrysorrheum	Yellow-vented Flowerpecker	U	L	L	**L**
Dicaeum concolor	Plain Flowerpecker	U	L	H	**L**
Dicaeum cruentatum	Scarlet-backed Flowerpecker	U	L	L	**L**
Dicaeum erythrorhynchos	Pale-billed Flowerpecker	U	H	L	**L**
Dicaeum erythrothorax	Flame-breasted Flowerpecker	H	U	H	**L**
Dicaeum everetti	Brown-backed Flowerpecker	H	U	H	**L**
Dicaeum eximium	Red-banded Flowerpecker	H	U	H	**L**
Dicaeum geelvinkianum	Red-capped Flowerpecker	H	L	L	**L**
Dicaeum haematostictum	Visayan Flowerpecker	H	H	H	**H**
Dicaeum hirundinaceum	Mistletoebird	H	L	L	**L**
Dicaeum hypoleucum	Buzzing Flowerpecker	H	U	L	**L**
Dicaeum igniferum	Black-fronted Flowerpecker	H	U	L	**L**
Dicaeum ignipectus	Fire-breasted Flowerpecker	U	L	H	**L**

Dicaeum maugei	Red-chested Flowerpecker	H	H	L	**L**
Dicaeum melanoxanthum	Yellow-bellied Flowerpecker	U	L	H	**L**
Dicaeum monticolum	Black-sided Flowerpecker	U	U	H	**L**
Dicaeum nehrkorni	Crimson-crowned Flowerpecker	U	U	H	**L**
Dicaeum nigrilore	Olive-capped Flowerpecker	H	H	H	**H**
Dicaeum nitidum	Louisiade Flowerpecker	H	U	H	**L**
Dicaeum pectorale	Olive-crowned Flowerpecker	H	U	H	**L**
Dicaeum proprium	Whiskered Flowerpecker	H	U	H	**L**
Dicaeum pygmaeum	Pygmy Flowerpecker	H	U	L	**L**
Dicaeum quadricolor	Cebu Flowerpecker	H	H	L	**L**
Dicaeum retrocinctum	Scarlet-collared Flowerpecker	H	U	L	**L**
Dicaeum sanguinolentum	Blood-breasted Flowerpecker	U	U	H	**L**
Dicaeum trigonostigma	Orange-bellied Flowerpecker	U	L	L	**L**
Dicaeum tristrami	Mottled Flowerpecker	H	U	H	**L**
Dicaeum trochileum	Scarlet-headed Flowerpecker	U	U	U	**L**
Dicaeum vincens	White-throated Flowerpecker	H	H	H	**H**
Dicaeum vulneratum	Ashy Flowerpecker	H	U	H	**L**
Dichropogon poecilinotus	Scale-backed Antbird	H	H	U	**L**
Dichrozona cincta	Banded Antbird	H	H	L	**L**
Dicrurus adsimilis	Fork-tailed Drongo	U	L	U	**L**
Dicrurus aeneus	Bronzed Drongo	U	L	L	**L**
Dicrurus aldabranus	Aldabra Drongo	H	H	U	**L**
Dicrurus andamanensis	Andaman Drongo	H	L	L	**L**
Dicrurus annectans	Crow-billed Drongo	U	L	H	**L**
Dicrurus atripennis	Shining Drongo	H	H	L	**L**
Dicrurus balicassius	Balicassiao	H	U	L	**L**
Dicrurus bracteatus	Spangled Drongo	U	L	U	**L**
Dicrurus caerulescens	White-bellied Drongo	U	L	L	**L**
Dicrurus densus	Wallacean Drongo	H	U	L	**L**
Dicrurus forficatus	Crested Drongo	U	L	L	**L**
Dicrurus fuscipennis	Grand Comoro Drongo	H	H	H	**H**
Dicrurus hottentottus	Hair-crested Drongo	U	L	H	**L**
Dicrurus leucophaeus	Ashy Drongo	U	L	U	**L**
Dicrurus ludwigii	Square-tailed Drongo	U	L	L	**L**
Dicrurus macrocercus	Black Drongo	U	L	U	**L**
Dicrurus megarhynchus	Ribbon-tailed Drongo	H	U	U	**L**
Dicrurus montanus	Sulawesi Drongo	H	U	H	**L**
Dicrurus paradiseus	Greater Racket-tailed Drongo	U	L	L	**L**
Dicrurus remifer	Lesser Racket-tailed Drongo	U	L	H	**L**
Dicrurus sumatranus	Sumatran Drongo	H	U	H	**L**
Dicrurus waldenii	Mayotte Drongo	H	U	U	**L**

Didunculus strigirostris	Tooth-billed Pigeon	H	H	U	**L**
Diglossa albilatera	White-sided Flowerpiercer	U	H	H	**L**
Diglossa baritula	Cinnamon-bellied Flowerpiercer	L	H	L	**L**
Diglossa brunneiventris	Black-throated Flowerpiercer	U	U	H	**L**
Diglossa caerulescens	Bluish Flowerpiercer	U	U	L	**L**
Diglossa carbonaria	Grey-bellied Flowerpiercer	U	U	H	**L**
Diglossa cyanea	Masked Flowerpiercer	U	U	L	**L**
Diglossa duidae	Scaled Flowerpiercer	U	U	H	**L**
Diglossa glauca	Deep-blue Flowerpiercer	H	U	H	**L**
Diglossa gloriosa	Merida Flowerpiercer	H	U	H	**L**
Diglossa gloriosissima	Chestnut-bellied Flowerpiercer	H	H	H	**H**
Diglossa humeralis	Black Flowerpiercer	U	H	H	**L**
Diglossa indigotica	Indigo Flowerpiercer	U	U	H	**L**
Diglossa lafresnayii	Glossy Flowerpiercer	U	U	H	**L**
Diglossa major	Greater Flowerpiercer	H	U	H	**L**
Diglossa mystacalis	Moustached Flowerpiercer	U	H	H	**L**
Diglossa plumbea	Slaty Flowerpiercer	U	H	H	**L**
Diglossa sittoides	Rusty Flowerpiercer	U	H	L	**L**
Diglossa venezuelensis	Venezuelan Flowerpiercer	H	H	H	**H**
Dinemellia dinemelli	White-headed Buffalo-weaver	H	U	L	**L**
Dinopium benghalense	Black-rumped Flameback	U	L	L	**L**
Dinopium javanense	Common Flameback	U	L	U	**L**
Dinopium rafflesii	Olive-backed Woodpecker	H	U	H	**L**
Dinopium shorii	Himalayan Flameback	H	L	U	**L**
Diomedea amsterdamensis	Amsterdam Albatross	H	H	U	**L**
Diomedea antipodensis	Antipodean Albatross	H	H	H	**H**
Diomedea dabbenena	Tristan Albatross	H	H	L	**L**
Diomedea epomophora	Southern Royal Albatross	H	H	H	**H**
Diomedea exulans	Wandering Albatross	H	H	H	**H**
Diomedea sanfordi	Northern Royal Albatross	H	H	H	**H**
Diopsittaca nobilis	Red-shouldered Macaw	U	H	L	**L**
Dioptrornis brunneus	Angola Slaty Flycatcher	U	U	L	**L**
Dioptrornis chocolatinus	Abyssinian Slaty Flycatcher	U	L	L	**L**
Dioptrornis fischeri	White-eyed Slaty Flycatcher	U	L	L	**L**
Discosura conversii	Green Thorntail	H	U	L	**L**
Discosura langsdorffi	Black-bellied Thorntail	H	H	L	**L**
Discosura letitiae	Coppery Thorntail	H	U	U	**L**
Discosura longicaudus	Racket-tailed Coquette	H	H	H	**H**
Discosura popelairii	Wire-crested Thorntail	H	U	H	**L**
Diuca diuca	Common Diuca-finch	H	U	L	**L**
Diuca speculifera	White-winged Diuca-finch	H	U	H	**L**

Dives atroviolaceus	Cuban Blackbird	U	L	H	**L**
Dives dives	Melodious Blackbird	L	L	H	**L**
Dives warszewiczi	Scrub Blackbird	U	U	H	**L**
Dolichonyx oryzivorus	Bobolink	L	L	L	**L**
Doliornis remseni	Chestnut-bellied Cotinga	H	U	H	**L**
Doliornis sclateri	Bay-vented Cotinga	H	U	H	**L**
Dolospingus fringilloides	White-naped Seedeater	H	U	H	**L**
Donacobius atricapilla	Black-capped Donacobius	U	L	L	**L**
Donacospiza albifrons	Long-tailed Reed-finch	H	U	L	**L**
Doricha eliza	Mexican Sheartail	H	H	L	**L**
Doricha enicura	Slender Sheartail	L	U	H	**L**
Doryfera johannae	Blue-fronted Lancebill	H	H	H	**H**
Doryfera ludovicae	Green-fronted Lancebill	H	H	L	**L**
Drepanoptila holosericea	Cloven-feathered Dove	U	H	H	**L**
Dromaeocercus brunneus	Brown Emu-tail	H	H	H	**H**
Dromaius novaehollandiae	Emu	H	H	L	**L**
Dromas ardeola	Crab Plover	L	H	H	**L**
Dromococcyx pavoninus	Pavonine Cuckoo	H	U	L	**L**
Dromococcyx phasianellus	Pheasant Cuckoo	L	U	H	**L**
Drymocichla incana	Red-winged Grey Warbler	U	U	U	**L**
Drymodes brunneopygia	Southern Scrub-robin	U	H	U	**L**
Drymodes superciliaris	Northern Scrub-robin	U	H	L	**L**
Drymophila caudata	Long-tailed Antbird	H	U	L	**L**
Drymophila devillei	Striated Antbird	H	H	L	**L**
Drymophila ferruginea	Ferruginous Antbird	H	H	L	**L**
Drymophila genei	Rufous-tailed Antbird	H	H	H	**H**
Drymophila malura	Dusky-tailed Antbird	H	H	L	**L**
Drymophila ochropyga	Ochre-rumped Antbird	H	H	L	**L**
Drymophila rubricollis	Bertoni's Antbird	H	H	L	**L**
Drymophila squamata	Scaled Antbird	U	H	H	**L**
Drymornis bridgesii	Scimitar-billed Woodcreeper	H	L	L	**L**
Dryocopus galeatus	Helmeted Woodpecker	H	H	L	**L**
Dryocopus hodgei	Andaman Woodpecker	H	H	L	**L**
Dryocopus javensis	White-bellied Woodpecker	H	H	U	**L**
Dryocopus lineatus	Lineated Woodpecker	L	H	L	**L**
Dryocopus martius	Black Woodpecker	H	H	H	**H**
Dryocopus pileatus	Pileated Woodpecker	H	H	L	**L**
Dryocopus schulzi	Black-bodied Woodpecker	H	H	L	**L**
Dryolimnas cuvieri	White-throated Rail	H	L	L	**L**
Dryoscopus angolensis	Pink-footed Puffback	H	U	H	**L**
Dryoscopus cubla	Black-backed Puffback	U	H	L	**L**
Dryoscopus gambensis	Northern Puffback	U	H	H	**L**
Dryoscopus pringlii	Pringle's Puffback	U	H	U	**L**
Dryoscopus sabini	Large-billed Puffback	H	U	L	**L**

Dryoscopus senegalensis	Red-eyed Puffback	H	H	H	**H**
Dryotriorchis spectabilis	Congo Serpent-eagle	H	H	H	**H**
Dubusia taeniata	Buff-breasted Mountain-tanager	H	U	H	**L**
Ducula aenea	Green Imperial-pigeon	U	H	L	**L**
Ducula aurorae	Polynesian Imperial-pigeon	H	H	U	**L**
Ducula badia	Mountain Imperial-pigeon	H	H	L	**L**
Ducula bakeri	Vanuatu Imperial-pigeon	H	H	H	**H**
Ducula basilica	Cinnamon-bellied Imperial-pigeon	H	H	H	**H**
Ducula bicolor	Pied Imperial-pigeon	H	H	H	**H**
Ducula brenchleyi	Chestnut-bellied Imperial-pigeon	H	H	H	**H**
Ducula carola	Spotted Imperial-pigeon	H	H	L	**L**
Ducula chalconota	Shining Imperial-pigeon	H	H	H	**H**
Ducula cineracea	Timor Imperial-pigeon	H	H	H	**H**
Ducula concinna	Elegant Imperial-pigeon	H	H	L	**L**
Ducula finschii	Finsch's Imperial-pigeon	H	H	H	**H**
Ducula forsteni	White-bellied Imperial-pigeon	H	H	H	**H**
Ducula galeata	Marquesan Imperial-pigeon	H	H	U	**L**
Ducula goliath	New Caledonian Imperial-pigeon	H	H	H	**H**
Ducula lacernulata	Dark-backed Imperial-pigeon	U	H	H	**L**
Ducula latrans	Peale's Imperial-pigeon	H	H	L	**L**
Ducula luctuosa	White Imperial-pigeon	H	H	H	**H**
Ducula melanochroa	Bismarck Imperial-pigeon	H	H	H	**H**
Ducula mindorensis	Mindoro Imperial-pigeon	H	H	L	**L**
Ducula mullerii	Collared Imperial-pigeon	H	H	L	**L**
Ducula myristicivora	Spice Imperial-pigeon	H	H	H	**H**
Ducula oceanica	Micronesian Imperial-pigeon	H	H	H	**H**
Ducula pacifica	Pacific Imperial-pigeon	U	H	L	**L**
Ducula perspicillata	White-eyed Imperial-pigeon	H	H	H	**H**
Ducula pickeringii	Grey Imperial-pigeon	H	H	H	**H**
Ducula pinon	Pinon Imperial-pigeon	H	H	L	**L**
Ducula pistrinaria	Island Imperial-pigeon	H	H	L	**L**
Ducula poliocephala	Pink-bellied Imperial-pigeon	H	H	L	**L**
Ducula radiata	Grey-headed Imperial-pigeon	H	H	H	**H**
Ducula rosacea	Pink-headed Imperial-pigeon	H	H	L	**L**
Ducula rubricera	Red-knobbed Imperial-pigeon	H	H	H	**H**
Ducula rufigaster	Purple-tailed Imperial-pigeon	H	H	L	**L**
Ducula spilorrhoa	Torresian Imperial-pigeon	U	H	U	**L**
Ducula subflavescens	Yellowish Imperial-pigeon	H	H	H	**H**

Ducula whartoni	Christmas Imperial-pigeon	H	H	U	**L**
Ducula zoeae	Banded Imperial-pigeon	H	H	L	**L**
Dulus dominicus	Palmchat	U	L	H	**L**
Dumetella carolinensis	Grey Catbird	H	H	L	**L**
Dumetia hyperythra	Tawny-bellied Babbler	U	L	U	**L**
Dysithamnus leucostictus	White-spotted Antvireo	H	H	U	**L**
Dysithamnus mentalis	Plain Antvireo	L	H	L	**L**
Dysithamnus occidentalis	Bicoloured Antvireo	H	H	H	**H**
Dysithamnus plumbeus	Plumbeous Antvireo	H	H	H	**H**
Dysithamnus puncticeps	Spot-crowned Antvireo	H	H	L	**L**
Dysithamnus stictothorax	Spot-breasted Antvireo	U	U	L	**L**
Dysithamnus striaticeps	Streak-crowned Antvireo	H	H	H	**H**
Dysithamnus xanthopterus	Rufous-backed Antvireo	H	H	L	**L**
Eclectus roratus	Eclectus Parrot	U	H	L	**L**
Egretta ardesiaca	Black Heron	L	H	L	**L**
Egretta caerulea	Little Blue Heron	L	H	H	**L**
Egretta dimorpha	Dimorphic Egret	U	H	U	**L**
Egretta eulophotes	Chinese Egret	H	H	H	**H**
Egretta garzetta	Little Egret	L	H	L	**L**
Egretta gularis	Western Reef-egret	L	H	L	**L**
Egretta novaehollandiae	White-faced Heron	L	H	L	**L**
Egretta rufescens	Reddish Egret	L	H	H	**L**
Egretta sacra	Pacific Reef-egret	U	H	U	**L**
Egretta thula	Snowy Egret	L	H	L	**L**
Egretta tricolor	Tricoloured Heron	L	H	H	**L**
Egretta vinaceigula	Slaty Egret	H	H	L	**L**
Elaenia albiceps	White-crested Elaenia	U	L	L	**L**
Elaenia chiriquensis	Lesser Elaenia	U	H	H	**L**
Elaenia cristata	Plain-crested Elaenia	U	H	H	**L**
Elaenia dayi	Great Elaenia	U	U	H	**L**
Elaenia fallax	Greater Antillean Elaenia	U	H	H	**L**
Elaenia flavogaster	Yellow-bellied Elaenia	L	H	L	**L**
Elaenia frantzii	Mountain Elaenia	U	H	H	**L**
Elaenia gigas	Mottle-backed Elaenia	U	U	H	**L**
Elaenia martinica	Caribbean Elaenia	H	L	H	**L**
Elaenia mesoleuca	Olivaceous Elaenia	H	L	L	**L**
Elaenia obscura	Highland Elaenia	H	H	L	**L**
Elaenia pallatangae	Sierran Elaenia	U	H	L	**L**
Elaenia parvirostris	Small-billed Elaenia	U	L	H	**L**
Elaenia pelzelni	Brownish Elaenia	H	H	H	**H**
Elaenia ridleyana	Noronha Elaenia	H	H	U	**L**
Elaenia ruficeps	Rufous-crowned Elaenia	H	H	H	**H**
Elaenia spectabilis	Large Elaenia	U	H	L	**L**
Elaenia strepera	Slaty Elaenia	U	H	H	**L**

Elanoides forficatus	American Swallow-tailed Kite	L	H	L	**L**
Elanus axillaris	Black-shouldered Kite	U	H	U	**L**
Elanus caeruleus	Black-winged Kite	L	H	L	**L**
Elanus leucurus	White-tailed Kite	L	H	L	**L**
Elanus scriptus	Letter-winged Kite	H	H	L	**L**
Electron carinatum	Keel-billed Motmot	H	U	H	**L**
Electron platyrhynchum	Broad-billed Motmot	H	L	L	**L**
Eleothreptus anomalus	Sickle-winged Nightjar	H	U	H	**L**
Eleothreptus candicans	White-winged Nightjar	H	H	L	**L**
Elminia albicauda	White-tailed Blue-flycatcher	U	H	L	**L**
Elminia albiventris	White-bellied Crested-flycatcher	U	H	L	**L**
Elminia albonotata	White-tailed Crested-flycatcher	U	H	L	**L**
Elminia longicauda	African Blue-flycatcher	U	H	H	**L**
Elminia nigromitrata	Dusky Crested-flycatcher	H	H	L	**L**
Elseyornis melanops	Black-fronted Dotterel	H	H	U	**L**
Elvira chionura	White-tailed Emerald	H	U	H	**L**
Elvira cupreiceps	Coppery-headed Emerald	U	H	H	**L**
Emberiza affinis	Brown-rumped Bunting	U	U	U	**L**
Emberiza aureola	Yellow-breasted Bunting	H	L	H	**L**
Emberiza bruniceps	Red-headed Bunting	H	L	L	**L**
Emberiza buchanani	Grey-necked Bunting	H	L	H	**L**
Emberiza cabanisi	Cabanis's Bunting	U	L	L	**L**
Emberiza caesia	Cretzschmar's Bunting	H	L	L	**L**
Emberiza capensis	Cape Bunting	U	L	U	**L**
Emberiza chrysophrys	Yellow-browed Bunting	H	L	H	**L**
Emberiza cia	Rock Bunting	H	L	L	**L**
Emberiza cineracea	Cinereous Bunting	H	L	H	**L**
Emberiza cioides	Meadow Bunting	U	L	U	**L**
Emberiza cirlus	Cirl Bunting	H	L	L	**L**
Emberiza citrinella	Yellowhammer	H	L	H	**L**
Emberiza elegans	Yellow-throated Bunting	U	L	U	**L**
Emberiza flaviventris	African Golden-breasted Bunting	U	L	L	**L**
Emberiza fucata	Chestnut-eared Bunting	U	L	H	**L**
Emberiza godlewskii	Godlewski's Bunting	U	L	U	**L**
Emberiza hortulana	Ortolan Bunting	H	L	H	**L**
Emberiza impetuani	Lark-like Bunting	H	L	H	**L**
Emberiza jankowskii	Rufous-backed Bunting	H	L	H	**L**
Emberiza koslowi	Tibetan Bunting	H	U	H	**L**
Emberiza leucocephalos	Pine Bunting	H	L	H	**L**
Emberiza melanocephala	Black-headed Bunting	H	L	L	**L**
Emberiza pallasi	Pallas's Bunting	H	L	H	**L**
Emberiza poliopleura	Somali Golden-breasted	H	L	L	**L**

	Bunting				
Emberiza pusilla	Little Bunting	H	H	H	**H**
Emberiza rustica	Rustic Bunting	H	L	H	**L**
Emberiza rutila	Chestnut Bunting	H	L	H	**L**
Emberiza schoeniclus	Reed Bunting	H	L	H	**L**
Emberiza socotrana	Socotra Bunting	H	H	H	**H**
Emberiza spodocephala	Black-faced Bunting	U	L	H	**L**
Emberiza stewarti	Chestnut-breasted Bunting	U	L	U	**L**
Emberiza striolata	House Bunting	H	L	H	**L**
Emberiza sulphurata	Yellow Bunting	H	H	H	**H**
Emberiza tahapisi	Cinnamon-breasted Bunting	U	L	U	**L**
Emberiza tristrami	Tristram's Bunting	H	L	H	**L**
Emberiza variabilis	Grey Bunting	U	L	H	**L**
Emberiza yessoensis	Ochre-rumped Bunting	H	L	H	**L**
Emberizoides duidae	Duida Grass-finch	H	H	H	**H**
Emberizoides herbicola	Wedge-tailed Grass-finch	U	H	L	**L**
Emberizoides ypiranganus	Grey-cheeked Grass-finch	H	U	L	**L**
Embernagra longicauda	Pale-throated Pampa-finch	U	U	L	**L**
Embernagra platensis	Great Pampa-finch	H	U	L	**L**
Emblema pictum	Painted Firetail	H	L	L	**L**
Eminia lepida	Grey-capped Warbler	U	L	H	**L**
Empidonax affinis	Pine Flycatcher	H	H	L	**L**
Empidonax albigularis	White-throated Flycatcher	L	L	H	**L**
Empidonax alnorum	Alder Flycatcher	L	L	L	**L**
Empidonax atriceps	Black-capped Flycatcher	U	H	H	**L**
Empidonax difficilis	Pacific-slope Flycatcher	L	L	H	**L**
Empidonax flavescens	Yellowish Flycatcher	L	L	H	**L**
Empidonax flaviventris	Yellow-bellied Flycatcher	H	L	L	**L**
Empidonax fulvifrons	Buff-breasted Flycatcher	L	L	L	**L**
Empidonax hammondii	Hammond's Flycatcher	H	L	H	**L**
Empidonax minimus	Least Flycatcher	H	L	L	**L**
Empidonax oberholseri	American Dusky Flycatcher	H	L	H	**L**
Empidonax occidentalis	Cordilleran Flycatcher	H	L	H	**L**
Empidonax traillii	Willow Flycatcher	L	L	H	**L**
Empidonax virescens	Acadian Flycatcher	L	L	L	**L**
Empidonax wrightii	Grey Flycatcher	H	L	H	**L**
Empidonomus aurantioatrocristatus	Crowned Slaty Flycatcher	U	L	L	**L**
Empidonomus varius	Variegated Flycatcher	U	L	L	**L**
Empidornis semipartitus	Silverbird	U	H	U	**L**
Enicognathus ferrugineus	Austral Parakeet	H	H	U	**L**
Enicognathus leptorhynchus	Slender-billed Parakeet	H	H	L	**L**
Enicurus immaculatus	Black-backed Forktail	U	L	U	**L**
Enicurus leschenaulti	White-crowned Forktail	U	L	H	**L**

Enicurus maculatus	Spotted Forktail	U	L	U	**L**
Enicurus ruficapillus	Chestnut-naped Forktail	H	H	H	**H**
Enicurus schistaceus	Slaty-backed Forktail	H	L	U	**L**
Enicurus scouleri	Little Forktail	U	L	U	**L**
Enicurus velatus	Sunda Forktail	U	U	H	**L**
Enodes erythrophris	Fiery-browed Myna	H	U	U	**L**
Ensifera ensifera	Sword-billed Hummingbird	H	U	L	**L**
Entomodestes coracinus	Black Solitaire	H	H	H	**H**
Entomodestes leucotis	White-eared Solitaire	H	H	H	**H**
Entomyzon cyanotis	Blue-faced Honeyeater	U	L	L	**L**
Eophona migratoria	Yellow-billed Grosbeak	U	U	H	**L**
Eophona personata	Japanese Grosbeak	U	U	H	**L**
Eopsaltria australis	Eastern Yellow Robin	H	H	L	**L**
Eopsaltria flaviventris	Yellow-bellied Robin	H	H	H	**H**
Eopsaltria georgiana	White-breasted Robin	H	H	H	**H**
Eopsaltria griseogularis	Western Yellow Robin	U	H	U	**L**
Eopsaltria pulverulenta	Mangrove Robin	U	H	H	**L**
Eos bornea	Red Lory	H	H	H	**H**
Eos cyanogenia	Black-winged Lory	H	H	H	**H**
Eos histrio	Red-and-blue Lory	H	H	H	**H**
Eos reticulata	Blue-streaked Lory	H	H	L	**L**
Eos semilarvata	Blue-eared Lory	H	H	H	**H**
Eos squamata	Violet-necked Lory	H	H	H	**H**
Ephippiorhynchus asiaticus	Black-necked Stork	L	H	L	**L**
Ephippiorhynchus senegalensis	Saddle-billed Stork	H	H	L	**L**
Epimachus albertisi	Black-billed Sicklebill	H	H	U	**L**
Epimachus bruijnii	Pale-billed Sicklebill	H	H	H	**H**
Epimachus fastuosus	Black Sicklebill	H	H	H	**H**
Epimachus meyeri	Brown Sicklebill	U	H	H	**L**
Epinecrophylla erythrura	Rufous-tailed Antwren	H	H	L	**L**
Epinecrophylla fjeldsaai	Brown-backed Antwren	H	H	H	**H**
Epinecrophylla fulviventris	Checker-throated Antwren	H	H	H	**H**
Epinecrophylla haematonota	Stipple-throated Antwren	H	H	H	**H**
Epinecrophylla leucophthalma	White-eyed Antwren	H	H	H	**H**
Epinecrophylla ornata	Ornate Antwren	H	H	L	**L**
Epinecrophylla spodionota	Foothill Antwren	H	H	H	**H**
Epthianura albifrons	White-fronted Chat	U	L	U	**L**
Epthianura aurifrons	Orange Chat	U	L	U	**L**
Epthianura crocea	Yellow Chat	U	L	U	**L**
Epthianura tricolor	Crimson Chat	H	L	L	**L**
Eremalauda dunni	Dunn's Lark	H	L	H	**L**

Eremalauda starki	Stark's Lark	H	L	H	**L**
Eremiornis carteri	Spinifexbird	H	H	L	**L**
Eremobius phoenicurus	Band-tailed Earthcreeper	H	L	H	**L**
Eremomela atricollis	Black-necked Eremomela	U	H	H	**L**
Eremomela badiceps	Rufous-crowned Eremomela	H	U	L	**L**
Eremomela flavicrissalis	Yellow-vented Eremomela	U	H	U	**L**
Eremomela gregalis	Yellow-rumped Eremomela	H	H	L	**L**
Eremomela icteropygialis	Yellow-bellied Eremomela	U	H	L	**L**
Eremomela pusilla	Senegal Eremomela	U	H	U	**L**
Eremomela scotops	Greencap Eremomela	U	H	U	**L**
Eremomela turneri	Turner's Eremomela	H	U	H	**L**
Eremomela usticollis	Burnt-neck Eremomela	U	L	U	**L**
Eremophila alpestris	Horned Lark	H	L	H	**L**
Eremophila bilopha	Temminck's Lark	H	L	H	**L**
Eremopterix australis	Black-eared Sparrow-lark	H	H	H	**H**
Eremopterix griseus	Ashy-crowned Sparrow-lark	U	L	U	**L**
Eremopterix leucopareia	Fischer's Sparrow-lark	U	L	H	**L**
Eremopterix leucotis	Chestnut-backed Sparrow-lark	U	H	H	**L**
Eremopterix nigriceps	Black-crowned Sparrow-lark	H	L	H	**L**
Eremopterix signatus	Chestnut-headed Sparrow-lark	H	L	L	**L**
Eremopterix verticalis	Grey-backed Sparrow-lark	H	L	L	**L**
Ergaticus ruber	Red Warbler	H	H	L	**L**
Ergaticus versicolor	Pink-headed Warbler	H	H	H	**H**
Eriocnemis alinae	Emerald-bellied Puffleg	H	U	H	**L**
Eriocnemis cupreoventris	Coppery-bellied Puffleg	U	H	H	**L**
Eriocnemis derbyi	Black-thighed Puffleg	H	H	H	**H**
Eriocnemis glaucopoides	Blue-capped Puffleg	U	H	H	**L**
Eriocnemis godini	Turquoise-throated Puffleg	H	H	H	**H**
Eriocnemis luciani	Sapphire-vented Puffleg	H	H	H	**H**
Eriocnemis mirabilis	Colourful Puffleg	H	H	H	**H**
Eriocnemis mosquera	Golden-breasted Puffleg	H	H	H	**H**
Eriocnemis nigrivestis	Black-breasted Puffleg	H	H	H	**H**
Eriocnemis vestita	Glowing Puffleg	U	H	H	**L**
Erithacus akahige	Japanese Robin	U	H	L	**L**
Erithacus komadori	Ryukyu Robin	H	H	H	**H**
Erithacus rubecula	European Robin	H	H	H	**H**
Erpornis zantholeuca	White-bellied Yuhina	U	L	U	**L**
Erythrocercus holochlorus	Yellow Flycatcher	H	U	L	**L**
Erythrocercus livingstonei	Livingstone's Flycatcher	U	L	L	**L**
Erythrocercus mccallii	Chestnut-capped Flycatcher	H	U	L	**L**
Erythrogonys cinctus	Red-kneed Dotterel	U	L	U	**L**
Erythropygia barbata	Miombo Scrub-robin	U	L	U	**L**
Erythropygia coryphaeus	Karoo Scrub-robin	H	H	L	**L**

Erythropygia galactotes	Rufous-tailed Scrub-robin	H	L	H	**L**
Erythropygia hartlaubi	Brown-backed Scrub-robin	U	L	U	**L**
Erythropygia leucophrys	Red-backed Scrub-robin	U	L	L	**L**
Erythropygia leucosticta	Forest Scrub-robin	U	U	L	**L**
Erythropygia paena	Kalahari Scrub-robin	H	L	H	**L**
Erythropygia quadrivirgata	Bearded Scrub-robin	U	L	L	**L**
Erythropygia signata	Brown Scrub-robin	H	L	L	**L**
Erythrotriorchis buergersi	Chestnut-shouldered Goshawk	H	H	H	**H**
Erythrotriorchis radiatus	Red Goshawk	H	H	L	**L**
Erythrura coloria	Red-eared Parrotfinch	H	H	H	**H**
Erythrura cyaneovirens	Red-headed Parrotfinch	H	L	U	**L**
Erythrura gouldiae	Gouldian Finch	H	L	L	**L**
Erythrura hyperythra	Tawny-breasted Parrotfinch	U	L	U	**L**
Erythrura kleinschmidti	Pink-billed Parrotfinch	H	H	H	**H**
Erythrura papuana	Papuan Parrotfinch	U	U	U	**L**
Erythrura pealii	Fiji Parrotfinch	H	L	L	**L**
Erythrura prasina	Pin-tailed Parrotfinch	U	L	U	**L**
Erythrura psittacea	Red-throated Parrotfinch	U	L	H	**L**
Erythrura regia	Royal Parrotfinch	H	H	H	**H**
Erythrura trichroa	Blue-faced Parrotfinch	L	L	L	**L**
Erythrura tricolor	Tricoloured Parrotfinch	H	U	L	**L**
Erythrura viridifacies	Green-faced Parrotfinch	H	L	H	**L**
Esacus giganteus	Beach Thick-knee	H	H	H	**H**
Esacus recurvirostris	Great Thick-knee	H	H	U	**L**
Estrilda astrild	Common Waxbill	U	L	L	**L**
Estrilda atricapilla	Black-headed Waxbill	U	H	U	**L**
Estrilda caerulescens	Lavender Waxbill	U	U	U	**L**
Estrilda charmosyna	Red-rumped Waxbill	U	L	U	**L**
Estrilda erythronotos	Black-cheeked Waxbill	U	L	U	**L**
Estrilda melanotis	Swee Waxbill	U	U	U	**L**
Estrilda melpoda	Orange-cheeked Waxbill	U	L	H	**L**
Estrilda nigriloris	Black-lored Waxbill	U	U	H	**L**
Estrilda nonnula	Black-crowned Waxbill	U	U	H	**L**
Estrilda paludicola	Fawn-breasted Waxbill	U	L	U	**L**
Estrilda perreini	Black-tailed Waxbill	U	L	L	**L**
Estrilda poliopareia	Anambra Waxbill	H	U	L	**L**
Estrilda rhodopyga	Crimson-rumped Waxbill	U	U	L	**L**
Estrilda rufibarba	Arabian Waxbill	H	U	H	**L**
Estrilda thomensis	Cinderella Waxbill	U	U	H	**L**
Estrilda troglodytes	Black-rumped Waxbill	U	L	H	**L**
Eubucco bourcierii	Red-headed Barbet	H	H	L	**L**
Eubucco richardsoni	Lemon-throated Barbet	H	H	L	**L**
Eubucco tucinkae	Scarlet-hooded Barbet	H	H	H	**H**

Eubucco versicolor	Versicoloured Barbet	H	H	H	**H**
Eucometis penicillata	Grey-headed Tanager	H	H	H	**H**
Eudocimus albus	White Ibis	L	H	L	**L**
Eudocimus ruber	Scarlet Ibis	H	H	H	**H**
Eudromia elegans	Elegant Crested-tinamou	H	H	H	**H**
Eudromia formosa	Quebracho Crested-tinamou	H	H	L	**L**
Eudromias morinellus	Eurasian Dotterel	H	H	H	**H**
Eudynamys cyanocephalus	Australian Koel	U	H	L	**L**
Eudynamys melanorhynchus	Black-billed Koel	U	U	H	**L**
Eudynamys scolopaceus	Asian Koel	U	H	L	**L**
Eudynamys taitensis	Long-tailed Koel	U	H	H	**L**
Eudyptes chrysocome	Southern Rockhopper Penguin	H	H	H	**H**
Eudyptes chrysolophus	Macaroni Penguin	H	H	L	**L**
Eudyptes moseleyi	Northern Rockhopper Penguin	H	H	U	**L**
Eudyptes pachyrhynchus	Fiordland Penguin	H	H	H	**H**
Eudyptes robustus	Snares Penguin	H	H	H	**H**
Eudyptes schlegeli	Royal Penguin	H	H	H	**H**
Eudyptes sclateri	Erect-crested Penguin	H	H	H	**H**
Eudyptula minor	Little Penguin	H	H	U	**L**
Eugenes fulgens	Magnificent Hummingbird	H	H	L	**L**
Eugerygone rubra	Garnet Robin	U	U	U	**L**
Eugralla paradoxa	Ochre-flanked Tapaculo	H	H	L	**L**
Eulabeornis castaneoventris	Chestnut Rail	U	L	H	**L**
Eulacestoma nigropectus	Wattled Ploughbill	U	U	H	**L**
Eulampis holosericeus	Green-throated Carib	H	H	H	**H**
Eulampis jugularis	Purple-throated Carib	H	H	H	**H**
Eulidia yarrellii	Chilean Woodstar	H	U	H	**L**
Eulipoa wallacei	Moluccan Megapode	H	U	H	**L**
Eumomota superciliosa	Turquoise-browed Motmot	L	L	H	**L**
Eumyias albicaudatus	Nilgiri Flycatcher	U	L	H	**L**
Eumyias indigo	Indigo Flycatcher	H	H	U	**L**
Eumyias panayensis	Island Flycatcher	U	U	U	**L**
Eumyias sordidus	Dull-blue Flycatcher	U	U	H	**L**
Eumyias thalassinus	Verditer Flycatcher	U	L	U	**L**
Euneornis campestris	Orangequit	H	H	H	**H**
Eunymphicus cornutus	Horned Parakeet	H	H	H	**H**
Eunymphicus uvaeensis	Uvea Parakeet	H	H	U	**L**
Eupetes macrocerus	Rail-babbler	H	H	H	**H**
Eupetomena macroura	Swallow-tailed Hummingbird	U	H	L	**L**
Euphagus carolinus	Rusty Blackbird	H	L	L	**L**
Euphagus cyanocephalus	Brewer's Blackbird	H	L	H	**L**

Eupherusa cyanophrys	Blue-capped Hummingbird	H	H	H	**H**
Eupherusa eximia	Stripe-tailed Hummingbird	H	U	H	**L**
Eupherusa nigriventris	Black-bellied Hummingbird	H	H	H	**H**
Eupherusa poliocerca	White-tailed Hummingbird	H	U	L	**L**
Euphonia affinis	Scrub Euphonia	U	L	U	**L**
Euphonia anneae	Tawny-capped Euphonia	U	U	U	**L**
Euphonia cayennensis	Golden-sided Euphonia	U	L	U	**L**
Euphonia chalybea	Green-throated Euphonia	H	U	L	**L**
Euphonia chlorotica	Purple-throated Euphonia	U	L	U	**L**
Euphonia chrysopasta	White-lored Euphonia	U	U	U	**L**
Euphonia concinna	Velvet-fronted Euphonia	U	U	U	**L**
Euphonia cyanocephala	Golden-rumped Euphonia	U	L	U	**L**
Euphonia elegantissima	Blue-rumped Euphonia	U	L	U	**L**
Euphonia finschi	Finsch's Euphonia	U	U	U	**L**
Euphonia fulvicrissa	Fulvous-vented Euphonia	H	U	U	**L**
Euphonia gouldi	Olive-backed Euphonia	H	H	U	**L**
Euphonia hirundinacea	Yellow-throated Euphonia	U	L	U	**L**
Euphonia imitans	Spot-crowned Euphonia	H	L	U	**L**
Euphonia jamaica	Jamaican Euphonia	U	L	U	**L**
Euphonia laniirostris	Thick-billed Euphonia	U	L	L	**L**
Euphonia luteicapilla	Yellow-crowned Euphonia	U	L	U	**L**
Euphonia mesochrysa	Bronze-green Euphonia	U	U	U	**L**
Euphonia minuta	White-vented Euphonia	U	L	U	**L**
Euphonia musica	Antillean Euphonia	U	L	U	**L**
Euphonia pectoralis	Chestnut-bellied Euphonia	H	L	U	**L**
Euphonia plumbea	Plumbeous Euphonia	U	U	U	**L**
Euphonia rufiventris	Rufous-bellied Euphonia	U	U	U	**L**
Euphonia saturata	Orange-crowned Euphonia	U	U	U	**L**
Euphonia trinitatis	Trinidad Euphonia	U	L	U	**L**
Euphonia violacea	Violaceous Euphonia	U	L	U	**L**
Euphonia xanthogaster	Orange-bellied Euphonia	U	L	U	**L**
Euplectes afer	Yellow-crowned Bishop	U	U	U	**L**
Euplectes albonotatus	White-winged Widowbird	U	U	U	**L**
Euplectes ardens	Red-collared Widowbird	U	U	L	**L**
Euplectes aureus	Golden-backed Bishop	U	U	H	**L**
Euplectes axillaris	Fan-tailed Widowbird	U	U	U	**L**
Euplectes capensis	Yellow Bishop	U	U	U	**L**
Euplectes diadematus	Fire-fronted Bishop	U	U	U	**L**
Euplectes franciscanus	Orange Bishop	U	L	U	**L**
Euplectes gierowii	Black Bishop	U	U	U	**L**
Euplectes hartlaubi	Marsh Widowbird	U	U	U	**L**
Euplectes hordeaceus	Black-winged Bishop	U	U	L	**L**
Euplectes jacksoni	Jackson's Widowbird	H	U	H	**L**
Euplectes macroura	Yellow-shouldered Widowbird	U	U	U	**L**

Euplectes nigroventris	Zanzibar Bishop	U	U	U	**L**
Euplectes orix	Red Bishop	U	U	L	**L**
Euplectes progne	Long-tailed Widowbird	U	U	U	**L**
Euplectes psammocromius	Buff-shouldered Widowbird	U	U	L	**L**
Eupodotis afra	Black Bustard	H	H	H	**H**
Eupodotis afraoides	White-quilled Bustard	H	H	H	**H**
Eupodotis caerulescens	Blue Bustard	H	H	H	**H**
Eupodotis gindiana	Buff-crested Bustard	U	H	U	**L**
Eupodotis hartlaubii	Hartlaub's Bustard	U	H	U	**L**
Eupodotis humilis	Little Brown Bustard	H	H	L	**L**
Eupodotis melanogaster	Black-bellied Bustard	U	H	U	**L**
Eupodotis rueppellii	Rueppell's Bustard	H	H	H	**H**
Eupodotis ruficrista	Red-crested Bustard	U	H	U	**L**
Eupodotis savilei	Savile's Bustard	U	H	U	**L**
Eupodotis senegalensis	White-bellied Bustard	U	H	H	**L**
Eupodotis vigorsii	Karoo Bustard	H	H	L	**L**
Euptilotis neoxenus	Eared Quetzal	H	H	L	**L**
Eurocephalus anguitimens	White-crowned Shrike	H	L	L	**L**
Eurocephalus rueppelli	White-rumped Shrike	U	L	U	**L**
Eurochelidon sirintarae	White-eyed River-martin	H	U	H	**L**
Eurostopodus archboldi	Mountain Eared-nightjar	H	H	H	**H**
Eurostopodus argus	Spotted Eared-nightjar	H	H	L	**L**
Eurostopodus diabolicus	Sulawesi Eared-nightjar	H	H	H	**H**
Eurostopodus macrotis	Great Eared-nightjar	U	H	L	**L**
Eurostopodus mystacalis	White-throated Eared-nightjar	U	H	L	**L**
Eurostopodus papuensis	Papuan Eared-nightjar	H	H	H	**H**
Eurostopodus temminckii	Malaysian Eared-nightjar	U	H	U	**L**
Euryceros prevostii	Helmet Vanga	H	H	H	**H**
Eurylaimus javanicus	Banded Broadbill	U	L	L	**L**
Eurylaimus ochromalus	Black-and-yellow Broadbill	H	L	H	**L**
Eurylaimus samarensis	Visayan Broadbill	H	U	L	**L**
Eurylaimus steerii	Mindanao Broadbill	H	H	H	**H**
Eurynorhynchus pygmeus	Spoon-billed Sandpiper	H	H	H	**H**
Euryptila subcinnamomea	Kopje Warbler	H	H	L	**L**
Eurypyga helias	Sunbittern	H	H	H	**H**
Eurystomus azureus	Purple Dollarbird	H	U	H	**L**
Eurystomus glaucurus	Broad-billed Roller	H	L	L	**L**
Eurystomus gularis	Blue-throated Roller	H	L	L	**L**
Eurystomus orientalis	Asian Dollarbird	H	L	H	**L**
Euscarthmus meloryphus	Tawny-crowned Pygmy-tyrant	U	H	L	**L**
Euscarthmus rufomarginatus	Rufous-sided Pygmy-tyrant	U	U	L	**L**

Euschistospiza cinereovinacea	Dusky Twinspot	U	L	U	**L**
Euschistospiza dybowskii	Dybowski's Twinspot	U	L	U	**L**
Euthlypis lachrymosa	Fan-tailed Warbler	H	H	H	**H**
Eutoxeres aquila	White-tipped Sicklebill	H	H	H	**H**
Eutoxeres condamini	Buff-tailed Sicklebill	H	H	H	**H**
Eutrichomyias rowleyi	Caerulean Paradise-flycatcher	H	H	H	**H**
Eutriorchis astur	Madagascar Serpent-eagle	H	H	H	**H**
Falco alopex	Fox Kestrel	H	H	U	**L**
Falco amurensis	Amur Falcon	L	H	H	**L**
Falco araea	Seychelles Kestrel	H	H	L	**L**
Falco ardosiaceus	Grey Kestrel	U	H	U	**L**
Falco berigora	Brown Falcon	U	H	U	**L**
Falco biarmicus	Lanner Falcon	H	H	L	**L**
Falco cenchroides	Nankeen Kestrel	H	H	L	**L**
Falco cherrug	Saker Falcon	H	H	H	**H**
Falco chicquera	Red-necked Falcon	L	H	H	**L**
Falco columbarius	Merlin	H	L	H	**L**
Falco concolor	Sooty Falcon	H	H	L	**L**
Falco cuvierii	African Hobby	H	H	H	**H**
Falco deiroleucus	Orange-breasted Falcon	H	H	H	**H**
Falco dickinsoni	Dickinson's Kestrel	H	H	L	**L**
Falco eleonorae	Eleonora's Falcon	H	H	L	**L**
Falco fasciinucha	Taita Falcon	H	H	H	**H**
Falco femoralis	Aplomado Falcon	L	H	L	**L**
Falco hypoleucos	Grey Falcon	H	H	L	**L**
Falco jugger	Laggar Falcon	L	H	L	**L**
Falco longipennis	Australian Hobby	L	H	L	**L**
Falco mexicanus	Prairie Falcon	H	H	H	**H**
Falco moluccensis	Spotted Kestrel	U	H	U	**L**
Falco naumanni	Lesser Kestrel	L	L	L	**L**
Falco newtoni	Madagascar Kestrel	U	H	U	**L**
Falco novaeseelandiae	New Zealand Falcon	H	H	H	**H**
Falco pelegrinoides	Barbary Falcon	H	H	H	**H**
Falco peregrinus	Peregrine Falcon	H	H	H	**H**
Falco punctatus	Mauritius Kestrel	H	H	H	**H**
Falco rufigularis	Bat Falcon	H	H	L	**L**
Falco rupicoloides	Greater Kestrel	H	H	L	**L**
Falco rusticolus	Gyr Falcon	H	H	H	**H**
Falco severus	Oriental Hobby	H	H	L	**L**
Falco sparverius	American Kestrel	L	L	L	**L**
Falco subbuteo	Eurasian Hobby	H	H	H	**H**
Falco subniger	Black Falcon	H	H	U	**L**
Falco tinnunculus	Common Kestrel	H	L	H	**L**

Falco vespertinus	Red-footed Falcon	H	L	H	**L**
Falco zoniventris	Banded Kestrel	H	H	U	**L**
Falculea palliata	Sickle-billed Vanga	H	L	U	**L**
Falcunculus frontatus	Crested Shrike-tit	H	L	L	**L**
Ferminia cerverai	Zapata Wren	H	H	H	**H**
Ficedula albicollis	Collared Flycatcher	H	L	H	**L**
Ficedula basilanica	Little Slaty Flycatcher	H	H	H	**H**
Ficedula bonthaina	Lompobatang Flycatcher	H	H	H	**H**
Ficedula buruensis	Cinnamon-chested Flycatcher	H	L	H	**L**
Ficedula crypta	Cryptic Flycatcher	U	U	U	**L**
Ficedula disposita	Furtive Flycatcher	H	U	H	**L**
Ficedula dumetoria	Rufous-chested Flycatcher	U	H	H	**L**
Ficedula harterti	Sumba Flycatcher	H	L	H	**L**
Ficedula henrici	Damar Flycatcher	H	H	L	**L**
Ficedula hodgsonii	Slaty-backed Flycatcher	U	L	U	**L**
Ficedula hyperythra	Snowy-browed Flycatcher	U	L	U	**L**
Ficedula hypoleuca	European Pied Flycatcher	H	L	H	**L**
Ficedula monileger	White-gorgeted Flycatcher	U	L	U	**L**
Ficedula mugimaki	Mugimaki Flycatcher	U	L	U	**L**
Ficedula narcissina	Narcissus Flycatcher	U	L	U	**L**
Ficedula nigrorufa	Black-and-rufous Flycatcher	U	H	H	**L**
Ficedula parva	Red-breasted Flycatcher	H	L	H	**L**
Ficedula platenae	Palawan Flycatcher	H	H	H	**H**
Ficedula rufigula	Rufous-throated Flycatcher	H	H	H	**H**
Ficedula sapphira	Sapphire Flycatcher	U	L	U	**L**
Ficedula semitorquata	Semi-collared Flycatcher	L	L	H	**L**
Ficedula solitaris	Rufous-browed Flycatcher	U	L	U	**L**
Ficedula strophiata	Rufous-gorgeted Flycatcher	U	L	U	**L**
Ficedula subrubra	Kashmir Flycatcher	H	L	H	**L**
Ficedula superciliaris	Ultramarine Flycatcher	U	L	U	**L**
Ficedula timorensis	Black-banded Flycatcher	U	L	H	**L**
Ficedula tricolor	Slaty-blue Flycatcher	U	L	U	**L**
Ficedula westermanni	Little Pied Flycatcher	U	H	U	**L**
Ficedula zanthopygia	Yellow-rumped Flycatcher	U	U	U	**L**
Florisuga fusca	Black Jacobin	U	H	L	**L**
Florisuga mellivora	White-necked Jacobin	H	U	H	**L**
Fluvicola albiventer	Black-backed Water-tyrant	U	L	L	**L**
Fluvicola nengeta	Masked Water-tyrant	U	U	L	**L**
Fluvicola pica	Pied Water-tyrant	U	L	H	**L**
Formicarius analis	Black-faced Antthrush	H	H	H	**H**
Formicarius colma	Rufous-capped Antthrush	H	H	H	**H**
Formicarius nigricapillus	Black-headed Antthrush	H	H	H	**H**
Formicarius rufifrons	Rufous-fronted Antthrush	H	U	H	**L**
Formicarius rufipectus	Rufous-breasted Antthrush	U	U	L	**L**

Formicivora erythronotos	Black-hooded Antwren	H	H	H	**H**
Formicivora grisea	White-fringed Antwren	H	H	H	**H**
Formicivora iheringi	Narrow-billed Antwren	H	H	L	**L**
Formicivora littoralis	Restinga Antwren	H	H	L	**L**
Formicivora melanogaster	Black-bellied Antwren	U	U	L	**L**
Formicivora rufa	Rusty-backed Antwren	U	H	L	**L**
Formicivora serrana	Serra Antwren	U	U	H	**L**
Forpus coelestis	Pacific Parrotlet	U	L	H	**L**
Forpus conspicillatus	Spectacled Parrotlet	U	L	H	**L**
Forpus cyanopygius	Mexican Parrotlet	L	L	H	**L**
Forpus modestus	Dusky-billed Parrotlet	H	U	H	**L**
Forpus passerinus	Green-rumped Parrotlet	H	L	H	**L**
Forpus xanthops	Yellow-faced Parrotlet	H	L	H	**L**
Forpus xanthopterygius	Blue-winged Parrotlet	U	L	L	**L**
Foudia eminentissima	Red-headed Fody	U	L	H	**L**
Foudia flavicans	Rodrigues Fody	H	H	H	**H**
Foudia madagascariensis	Madagascar Red Fody	U	L	L	**L**
Foudia omissa	Forest Fody	U	U	U	**L**
Foudia rubra	Mauritius Fody	H	H	H	**H**
Foudia sechellarum	Seychelles Fody	H	H	U	**L**
Foulehaio carunculatus	Wattled Honeyeater	H	H	L	**L**
Francolinus adspersus	Red-billed Francolin	H	L	H	**L**
Francolinus afer	Red-necked Spurfowl	U	L	L	**L**
Francolinus africanus	Grey-winged Francolin	H	H	L	**L**
Francolinus ahantensis	Ahanta Francolin	H	L	L	**L**
Francolinus albogularis	White-throated Francolin	H	L	U	**L**
Francolinus bicalcaratus	Double-spurred Francolin	U	L	H	**L**
Francolinus camerunensis	Mount Cameroon Francolin	H	U	H	**L**
Francolinus capensis	Cape Francolin	H	H	H	**H**
Francolinus castaneicollis	Chestnut-naped Francolin	U	L	L	**L**
Francolinus clappertoni	Clapperton's Francolin	U	U	H	**L**
Francolinus coqui	Coqui Francolin	U	L	U	**L**
Francolinus erckelii	Erckel's Francolin	U	L	L	**L**
Francolinus finschi	Finsch's Francolin	U	L	L	**L**
Francolinus francolinus	Black Francolin	U	L	H	**L**
Francolinus griseostriatus	Grey-striped Francolin	H	U	H	**L**
Francolinus gularis	Swamp Francolin	H	L	H	**L**
Francolinus hartlaubi	Hartlaub's Francolin	H	L	L	**L**
Francolinus harwoodi	Harwood's Francolin	H	U	H	**L**
Francolinus hildebrandti	Hildebrandt's Francolin	U	L	U	**L**
Francolinus icterorhynchus	Heuglin's Francolin	U	L	U	**L**
Francolinus jacksoni	Jackson's Francolin	H	L	H	**L**
Francolinus lathami	Forest Francolin	H	H	L	**L**
Francolinus leucoscepus	Yellow-necked Spurfowl	U	L	U	**L**

Francolinus levaillantii	Red-winged Francolin	U	L	U	**L**
Francolinus levaillantoides	Orange River Francolin	H	L	H	**L**
Francolinus nahani	Nahan's Francolin	H	L	H	**L**
Francolinus natalensis	Natal Francolin	U	L	H	**L**
Francolinus nobilis	Handsome Francolin	U	U	H	**L**
Francolinus ochropectus	Djibouti Francolin	H	H	H	**H**
Francolinus pictus	Painted Francolin	U	L	U	**L**
Francolinus pintadeanus	Chinese Francolin	U	L	H	**L**
Francolinus pondicerianus	Grey Francolin	U	L	U	**L**
Francolinus psilolaemus	Moorland Francolin	U	U	H	**L**
Francolinus rufopictus	Grey-breasted Spurfowl	U	L	H	**L**
Francolinus schlegelii	Schlegel's Francolin	H	L	U	**L**
Francolinus sephaena	Crested Francolin	U	L	L	**L**
Francolinus shelleyi	Shelley's Francolin	U	L	L	**L**
Francolinus squamatus	Scaly Francolin	H	L	H	**L**
Francolinus streptophorus	Ring-necked Francolin	H	L	H	**L**
Francolinus swainsonii	Swainson's Spurfowl	H	L	L	**L**
Francolinus swierstrai	Swierstra's Francolin	H	H	L	**L**
Fraseria cinerascens	White-browed Forest Flycatcher	H	H	H	**H**
Fraseria ocreata	African Forest Flycatcher	H	L	H	**L**
Fratercula arctica	Atlantic Puffin	H	H	L	**L**
Fratercula cirrhata	Tufted Puffin	H	H	L	**L**
Fratercula corniculata	Horned Puffin	H	H	L	**L**
Frederickena unduligera	Undulated Antshrike	H	H	L	**L**
Frederickena viridis	Black-throated Antshrike	H	H	H	**H**
Fregata andrewsi	Christmas Frigatebird	H	H	H	**H**
Fregata aquila	Ascension Frigatebird	L	H	U	**L**
Fregata ariel	Lesser Frigatebird	L	H	L	**L**
Fregata magnificens	Magnificent Frigatebird	L	H	L	**L**
Fregata minor	Greater Frigatebird	L	H	L	**L**
Fregetta grallaria	White-bellied Storm-petrel	L	H	L	**L**
Fregetta tropica	Black-bellied Storm-petrel	L	H	L	**L**
Fringilla coelebs	Eurasian Chaffinch	H	L	H	**L**
Fringilla montifringilla	Brambling	H	L	H	**L**
Fringilla teydea	Blue Chaffinch	H	H	H	**H**
Fulica alai	Hawaiian Coot	H	H	H	**H**
Fulica americana	American Coot	H	H	L	**L**
Fulica ardesiaca	Slate-coloured Coot	H	H	H	**H**
Fulica armillata	Red-gartered Coot	U	H	U	**L**
Fulica atra	Common Coot	H	H	H	**H**
Fulica caribaea	Caribbean Coot	U	H	H	**L**
Fulica cornuta	Horned Coot	H	H	H	**H**
Fulica cristata	Red-knobbed Coot	L	H	L	**L**

Fulica gigantea	Giant Coot	H	H	H	**H**
Fulica leucoptera	White-winged Coot	U	H	U	**L**
Fulica rufifrons	Red-fronted Coot	H	H	U	**L**
Fulmarus glacialis	Northern Fulmar	H	H	L	**L**
Fulmarus glacialoides	Southern Fulmar	H	H	L	**L**
Furnarius cristatus	Crested Hornero	H	L	L	**L**
Furnarius figulus	Wing-banded Hornero	H	H	L	**L**
Furnarius leucopus	Pale-legged Hornero	U	H	L	**L**
Furnarius minor	Lesser Hornero	H	L	H	**L**
Furnarius rufus	Rufous Hornero	U	L	L	**L**
Furnarius torridus	Pale-billed Hornero	H	H	H	**H**
Galbalcyrhynchus leucotis	White-eared Jacamar	H	H	H	**H**
Galbalcyrhynchus purusianus	Chestnut Jacamar	H	H	L	**L**
Galbula albirostris	Yellow-billed Jacamar	H	H	H	**H**
Galbula chalcothorax	Purplish Jacamar	H	H	H	**H**
Galbula cyanescens	Bluish-fronted Jacamar	H	H	H	**H**
Galbula cyanicollis	Blue-necked Jacamar	H	H	H	**H**
Galbula dea	Paradise Jacamar	H	H	H	**H**
Galbula galbula	Green-tailed Jacamar	H	H	H	**H**
Galbula leucogastra	Bronzy Jacamar	H	H	H	**H**
Galbula pastazae	Coppery-chested Jacamar	H	H	H	**H**
Galbula ruficauda	Rufous-tailed Jacamar	H	H	L	**L**
Galbula tombacea	White-chinned Jacamar	H	H	H	**H**
Galerida cristata	Crested Lark	H	L	H	**L**
Galerida deva	Tawny Lark	U	L	U	**L**
Galerida magnirostris	Large-billed Lark	H	H	H	**H**
Galerida malabarica	Malabar Lark	U	L	U	**L**
Galerida modesta	Sun Lark	U	H	H	**L**
Galerida theklae	Thekla Lark	H	L	L	**L**
Gallicolumba beccarii	Bronze Ground-dove	H	H	H	**H**
Gallicolumba canifrons	Palau Ground-dove	H	H	H	**H**
Gallicolumba crinigera	Mindanao Bleeding-heart	H	H	H	**H**
Gallicolumba erythroptera	Polynesian Ground-dove	H	H	U	**L**
Gallicolumba hoedtii	Wetar Ground-dove	H	H	H	**H**
Gallicolumba jobiensis	White-bibbed Ground-dove	U	H	H	**L**
Gallicolumba keayi	Negros Bleeding-heart	H	H	H	**H**
Gallicolumba kubaryi	Caroline Islands Ground-dove	H	H	H	**H**
Gallicolumba luzonica	Luzon Bleeding-heart	H	H	H	**H**
Gallicolumba menagei	Sulu Bleeding-heart	H	H	H	**H**
Gallicolumba platenae	Mindoro Bleeding-heart	H	H	L	**L**
Gallicolumba rubescens	Marquesan Ground-dove	H	H	U	**L**
Gallicolumba rufigula	Cinnamon Ground-dove	H	H	L	**L**

Gallicolumba sanctaecrucis	Santa Cruz Ground-dove	H	H	L	**L**
Gallicolumba stairi	Shy Ground-dove	H	H	L	**L**
Gallicolumba tristigmata	Sulawesi Ground-dove	H	H	H	**H**
Gallicolumba xanthonura	White-throated Ground-dove	H	H	H	**H**
Gallicrex cinerea	Watercock	U	L	H	**L**
Gallinago andina	Puna Snipe	H	L	H	**L**
Gallinago gallinago	Common Snipe	H	L	H	**L**
Gallinago hardwickii	Latham's Snipe	U	L	U	**L**
Gallinago imperialis	Imperial Snipe	H	U	H	**L**
Gallinago jamesoni	Andean Snipe	U	H	L	**L**
Gallinago macrodactyla	Madagascar Snipe	H	L	L	**L**
Gallinago media	Great Snipe	H	L	H	**L**
Gallinago megala	Swinhoe's Snipe	L	L	L	**L**
Gallinago nemoricola	Wood Snipe	H	U	H	**L**
Gallinago nigripennis	African Snipe	L	L	L	**L**
Gallinago nobilis	Noble Snipe	U	H	H	**L**
Gallinago paraguaiae	South American Snipe	U	L	U	**L**
Gallinago solitaria	Solitary Snipe	U	L	U	**L**
Gallinago stenura	Pintail Snipe	L	H	H	**L**
Gallinago stricklandii	Fuegian Snipe	H	H	H	**H**
Gallinago undulata	Giant Snipe	U	L	H	**L**
Gallinula angulata	Lesser Moorhen	L	L	L	**L**
Gallinula chloropus	Common Moorhen	L	L	L	**L**
Gallinula comeri	Gough Moorhen	H	H	U	**L**
Gallinula melanops	Spot-flanked Gallinule	H	L	L	**L**
Gallinula mortierii	Tasmanian Native-hen	H	L	L	**L**
Gallinula pacifica	Samoan Moorhen	H	H	U	**L**
Gallinula silvestris	Makira Moorhen	H	H	L	**L**
Gallinula tenebrosa	Dusky Moorhen	U	L	U	**L**
Gallinula ventralis	Black-tailed Native-hen	U	L	U	**L**
Gallirallus australis	Weka	L	L	H	**L**
Gallirallus calayanensis	Calayan Rail	H	H	U	**L**
Gallirallus insignis	New Britain Rail	H	U	H	**L**
Gallirallus lafresnayanus	New Caledonian Rail	H	U	H	**L**
Gallirallus okinawae	Okinawa Rail	H	H	H	**H**
Gallirallus owstoni	Guam Rail	H	L	H	**L**
Gallirallus philippensis	Buff-banded Rail	L	L	L	**L**
Gallirallus rovianae	Roviana Rail	H	L	H	**L**
Gallirallus sharpei	Sharpe's Rail	U	U	U	**L**
Gallirallus striatus	Slaty-breasted Rail	U	L	U	**L**
Gallirallus sylvestris	Lord Howe Woodhen	H	H	U	**L**
Gallirallus torquatus	Barred Rail	U	L	U	**L**
Galloperdix bicalcarata	Sri Lanka Spurfowl	H	H	H	**H**

Galloperdix lunulata	Painted Spurfowl	U	L	U	**L**
Galloperdix spadicea	Red Spurfowl	U	L	U	**L**
Gallus gallus	Red Junglefowl	U	L	L	**L**
Gallus lafayetii	Sri Lanka Junglefowl	U	H	U	**L**
Gallus sonneratii	Grey Junglefowl	U	L	U	**L**
Gallus varius	Green Junglefowl	U	L	U	**L**
Gampsonyx swainsonii	Pearl Kite	H	H	L	**L**
Gampsorhynchus rufulus	White-hooded Babbler	H	L	U	**L**
Gampsorhynchus torquatus	Collared Babbler	H	U	U	**L**
Garrodia nereis	Grey-backed Storm-petrel	H	H	H	**H**
Garrulax affinis	Black-faced Laughingthrush	U	L	U	**L**
Garrulax albogularis	White-throated Laughingthrush	H	H	U	**L**
Garrulax annamensis	Orange-breasted Laughingthrush	U	U	U	**L**
Garrulax austeni	Brown-capped Laughingthrush	H	L	H	**L**
Garrulax berthemyi	Buffy Laughingthrush	H	H	U	**L**
Garrulax bicolor	Sumatran Laughingthrush	H	H	H	**H**
Garrulax bieti	White-speckled Laughingthrush	H	H	H	**H**
Garrulax cachinnans	Rufous-breasted Laughingthrush	H	H	H	**H**
Garrulax caerulatus	Grey-sided Laughingthrush	U	L	U	**L**
Garrulax calvus	Bare-headed Laughingthrush	H	H	H	**H**
Garrulax canorus	Chinese Hwamei	U	L	U	**L**
Garrulax castanotis	Rufous-cheeked Laughingthrush	H	H	U	**L**
Garrulax chinensis	Black-throated Laughingthrush	U	L	U	**L**
Garrulax chrysopterus	Assam Laughingthrush	H	H	U	**L**
Garrulax cineraceus	Moustached Laughingthrush	U	L	U	**L**
Garrulax cinereifrons	Ashy-headed Laughingthrush	H	H	H	**H**
Garrulax courtoisi	Blue-crowned Laughingthrush	H	U	H	**L**
Garrulax davidi	Plain Laughingthrush	U	U	U	**L**
Garrulax delesserti	Wynaad Laughingthrush	H	H	H	**H**
Garrulax elliotii	Elliot's Laughingthrush	U	U	U	**L**
Garrulax erythrocephalus	Chestnut-crowned Laughingthrush	H	H	U	**L**
Garrulax ferrarius	Cambodian Laughingthrush	H	H	H	**H**
Garrulax formosus	Red-winged Laughingthrush	U	U	H	**L**
Garrulax galbanus	Yellow-throated Laughingthrush	U	L	U	**L**
Garrulax gularis	Rufous-vented Laughingthrush	H	H	U	**L**

Garrulax henrici	Brown-cheeked Laughingthrush	U	L	U	**L**
Garrulax imbricatus	Bhutan Laughingthrush	U	U	U	**L**
Garrulax jerdoni	Grey-breasted Laughingthrush	U	H	H	**L**
Garrulax konkakinhensis	Chestnut-eared Laughingthrush	H	H	L	**L**
Garrulax leucolophus	White-crested Laughingthrush	U	L	U	**L**
Garrulax lineatus	Streaked Laughingthrush	U	L	U	**L**
Garrulax lugubris	Black Laughingthrush	U	H	H	**L**
Garrulax lunulatus	Barred Laughingthrush	U	U	H	**L**
Garrulax maesi	Grey Laughingthrush	H	H	U	**L**
Garrulax maximus	Giant Laughingthrush	U	U	U	**L**
Garrulax melanostigma	Silver-eared Laughingthrush	U	U	U	**L**
Garrulax merulinus	Spot-breasted Laughingthrush	U	L	U	**L**
Garrulax milleti	Black-hooded Laughingthrush	H	H	L	**L**
Garrulax milnei	Red-tailed Laughingthrush	U	L	U	**L**
Garrulax mitratus	Chestnut-capped Laughingthrush	U	H	U	**L**
Garrulax monileger	Lesser Necklaced Laughingthrush	U	L	U	**L**
Garrulax morrisonianus	White-whiskered Laughingthrush	U	H	H	**L**
Garrulax ngoclinhensis	Golden-winged Laughingthrush	H	H	H	**H**
Garrulax nuchalis	Chestnut-backed Laughingthrush	U	L	H	**L**
Garrulax ocellatus	Spotted Laughingthrush	U	H	U	**L**
Garrulax palliatus	Sunda Laughingthrush	H	H	H	**H**
Garrulax pectoralis	Greater Necklaced Laughingthrush	U	L	H	**L**
Garrulax peninsulae	Malayan Laughingthrush	U	U	U	**L**
Garrulax perspicillatus	Masked Laughingthrush	U	L	U	**L**
Garrulax poecilorhynchus	Rusty Laughingthrush	H	H	H	**H**
Garrulax ruficeps	Rufous-crowned Laughingthrush	U	U	U	**L**
Garrulax ruficollis	Rufous-necked Laughingthrush	U	L	U	**L**
Garrulax rufifrons	Rufous-fronted Laughingthrush	H	H	H	**H**
Garrulax rufogularis	Rufous-chinned Laughingthrush	U	L	U	**L**
Garrulax sannio	White-browed Laughingthrush	U	L	U	**L**
Garrulax squamatus	Blue-winged Laughingthrush	U	L	U	**L**
Garrulax strepitans	White-necked	H	H	U	**L**

	Laughingthrush				
Garrulax striatus	Striated Laughingthrush	U	H	U	**L**
Garrulax subunicolor	Scaly Laughingthrush	U	L	U	**L**
Garrulax sukatschewi	Snowy-cheeked Laughingthrush	H	U	H	**L**
Garrulax taewanus	Taiwan Hwamei	L	U	H	**L**
Garrulax variegatus	Variegated Laughingthrush	U	L	U	**L**
Garrulax vassali	White-cheeked Laughingthrush	U	H	L	**L**
Garrulax virgatus	Striped Laughingthrush	U	L	H	**L**
Garrulax yersini	Collared Laughingthrush	H	H	H	**H**
Garrulus glandarius	Eurasian Jay	H	H	H	**H**
Garrulus lanceolatus	Black-headed Jay	U	H	U	**L**
Garrulus lidthi	Amami Jay	H	H	H	**H**
Gavia adamsii	Yellow-billed Loon	H	H	H	**H**
Gavia arctica	Arctic Loon	H	H	L	**L**
Gavia immer	Common Loon	H	H	L	**L**
Gavia pacifica	Pacific Loon	H	H	H	**H**
Gavia stellata	Red-throated Loon	H	H	H	**H**
Gecinulus grantia	Pale-headed Woodpecker	H	L	U	**L**
Gecinulus viridis	Bamboo Woodpecker	H	U	U	**L**
Geococcyx californianus	Greater Roadrunner	H	L	H	**L**
Geococcyx velox	Lesser Roadrunner	L	L	L	**L**
Geocolaptes olivaceus	Ground Woodpecker	H	H	L	**L**
Geoffroyus geoffroyi	Red-cheeked Parrot	H	L	L	**L**
Geoffroyus heteroclitus	Singing Parrot	H	U	L	**L**
Geoffroyus simplex	Blue-collared Parrot	H	U	U	**L**
Geomalia heinrichi	Geomalia	H	H	H	**H**
Geopelia cuneata	Diamond Dove	H	H	L	**L**
Geopelia humeralis	Bar-shouldered Dove	U	H	U	**L**
Geopelia maugeus	Barred Dove	H	U	L	**L**
Geopelia placida	Peaceful Dove	U	H	U	**L**
Geopelia striata	Zebra Dove	U	H	U	**L**
Geophaps plumifera	Spinifex Pigeon	H	H	L	**L**
Geophaps scripta	Squatter Pigeon	H	H	U	**L**
Geophaps smithii	Partridge Pigeon	L	H	L	**L**
Geositta antarctica	Short-billed Miner	H	H	H	**H**
Geositta crassirostris	Thick-billed Miner	H	U	H	**L**
Geositta cunicularia	Common Miner	H	L	L	**L**
Geositta isabellina	Creamy-rumped Miner	H	L	H	**L**
Geositta maritima	Greyish Miner	H	U	H	**L**
Geositta peruviana	Coastal Miner	H	H	H	**H**
Geositta poeciloptera	Campo Miner	H	L	L	**L**
Geositta punensis	Puna Miner	H	L	H	**L**
Geositta rufipennis	Rufous-banded Miner	H	L	L	**L**

Geositta saxicolina	Dark-winged Miner	H	U	H	**L**
Geositta tenuirostris	Slender-billed Miner	H	H	H	**H**
Geospiza conirostris	Large Cactus-finch	U	H	U	**L**
Geospiza difficilis	Sharp-beaked Ground-finch	U	L	U	**L**
Geospiza fortis	Medium Ground-finch	U	H	U	**L**
Geospiza fuliginosa	Small Ground-finch	H	L	H	**L**
Geospiza magnirostris	Large Ground-finch	H	L	H	**L**
Geospiza scandens	Common Cactus-finch	U	H	U	**L**
Geothlypis aequinoctialis	Masked Yellowthroat	U	H	L	**L**
Geothlypis beldingi	Belding's Yellowthroat	H	H	H	**H**
Geothlypis flavovelata	Altamira Yellowthroat	H	U	H	**L**
Geothlypis nelsoni	Hooded Yellowthroat	H	U	L	**L**
Geothlypis poliocephala	Grey-crowned Yellowthroat	L	L	H	**L**
Geothlypis rostrata	Bahama Yellowthroat	U	H	L	**L**
Geothlypis semiflava	Olive-crowned Yellowthroat	U	H	L	**L**
Geothlypis speciosa	Black-polled Yellowthroat	H	H	H	**H**
Geothlypis trichas	Common Yellowthroat	H	L	L	**L**
Geotrygon albifacies	White-faced Quail-dove	H	H	H	**H**
Geotrygon caniceps	Grey-headed Quail-dove	H	H	H	**H**
Geotrygon carrikeri	Tuxtla Quail-dove	H	H	H	**H**
Geotrygon chiriquensis	Rufous-breasted Quail-dove	H	H	H	**H**
Geotrygon chrysia	Key West Quail-dove	U	H	H	**L**
Geotrygon costaricensis	Buff-fronted Quail-dove	H	U	H	**L**
Geotrygon frenata	White-throated Quail-dove	U	H	L	**L**
Geotrygon goldmani	Russet-crowned Quail-dove	H	U	L	**L**
Geotrygon lawrencii	Purplish-backed Quail-dove	H	H	H	**H**
Geotrygon linearis	Lined Quail-dove	H	H	H	**H**
Geotrygon montana	Ruddy Quail-dove	L	H	L	**L**
Geotrygon mystacea	Bridled Quail-dove	H	H	H	**H**
Geotrygon saphirina	Sapphire Quail-dove	H	U	H	**L**
Geotrygon veraguensis	Olive-backed Quail-dove	H	H	L	**L**
Geotrygon versicolor	Crested Quail-dove	H	H	H	**H**
Geotrygon violacea	Violaceous Quail-dove	U	H	L	**L**
Geranoaetus melanoleucus	Black-chested Buzzard-eagle	H	H	L	**L**
Geranospiza caerulescens	Crane Hawk	L	H	L	**L**
Geronticus calvus	Southern Bald Ibis	H	H	L	**L**
Geronticus eremita	Northern Bald Ibis	H	H	H	**H**
Gerygone albofrontata	Chatham Gerygone	H	H	U	**L**
Gerygone chloronota	Green-backed Gerygone	U	L	L	**L**
Gerygone chrysogaster	Yellow-bellied Gerygone	H	L	L	**L**
Gerygone cinerea	Mountain Gerygone	H	U	H	**L**
Gerygone dorsalis	Rufous-sided Gerygone	H	L	L	**L**
Gerygone flavolateralis	Fan-tailed Gerygone	U	L	L	**L**
Gerygone fusca	Western Gerygone	H	H	L	**L**

Gerygone igata	Grey Gerygone	U	L	U	**L**
Gerygone inornata	Plain Gerygone	H	L	L	**L**
Gerygone levigaster	Mangrove Gerygone	U	L	L	**L**
Gerygone magnirostris	Large-billed Gerygone	H	H	H	**H**
Gerygone modesta	Norfolk Island Gerygone	H	H	U	**L**
Gerygone mouki	Brown Gerygone	U	L	H	**L**
Gerygone olivacea	White-throated Gerygone	U	L	L	**L**
Gerygone palpebrosa	Fairy Gerygone	U	H	U	**L**
Gerygone ruficollis	Brown-breasted Gerygone	U	U	H	**L**
Gerygone sulphurea	Golden-bellied Gerygone	U	L	L	**L**
Gerygone tenebrosa	Dusky Gerygone	H	L	H	**L**
Glareola cinerea	Grey Pratincole	H	H	H	**H**
Glareola lactea	Small Pratincole	U	H	U	**L**
Glareola maldivarum	Oriental Pratincole	U	H	U	**L**
Glareola nordmanni	Black-winged Pratincole	H	H	H	**H**
Glareola nuchalis	Rock Pratincole	H	H	L	**L**
Glareola ocularis	Madagascar Pratincole	H	H	L	**L**
Glareola pratincola	Collared Pratincole	L	H	H	**L**
Glaucidium albertinum	Albertine Owlet	H	U	L	**L**
Glaucidium bolivianum	Yungas Pygmy-owl	H	U	H	**L**
Glaucidium brasilianum	Ferruginous Pygmy-owl	L	L	L	**L**
Glaucidium brodiei	Collared Owlet	U	L	U	**L**
Glaucidium capense	African Barred Owlet	U	L	L	**L**
Glaucidium castaneum	Chestnut Owlet	H	U	H	**L**
Glaucidium castanonotum	Chestnut-backed Owlet	H	U	H	**L**
Glaucidium castanopterum	Javan Owlet	H	L	U	**L**
Glaucidium costaricanum	Costa Rican Pygmy-owl	U	L	H	**L**
Glaucidium cuculoides	Asian Barred Owlet	U	L	U	**L**
Glaucidium gnoma	Northern Pygmy-owl	H	L	H	**L**
Glaucidium griseiceps	Central American Pygmy-owl	L	L	H	**L**
Glaucidium hardyi	Amazonian Pygmy-owl	H	U	H	**L**
Glaucidium jardinii	Andean Pygmy-owl	U	U	H	**L**
Glaucidium minutissimum	Least Pygmy-owl	H	U	L	**L**
Glaucidium mooreorum	Pernambuco Pygmy-owl	H	H	H	**H**
Glaucidium nanum	Austral Pygmy-owl	U	L	H	**L**
Glaucidium nubicola	Cloud-forest Pygmy-owl	H	U	H	**L**
Glaucidium palmarum	Colima Pygmy-owl	L	L	H	**L**
Glaucidium parkeri	Subtropical Pygmy-owl	H	U	L	**L**
Glaucidium passerinum	Eurasian Pygmy-owl	H	L	H	**L**
Glaucidium perlatum	Pearl-spotted Owlet	U	L	L	**L**
Glaucidium peruanum	Peruvian Pygmy-owl	H	U	H	**L**
Glaucidium radiatum	Jungle Owlet	U	L	U	**L**
Glaucidium sanchezi	Tamaulipas Pygmy-owl	H	L	H	**L**
Glaucidium siju	Cuban Pygmy-owl	U	L	H	**L**

Glaucidium sjostedti	Sjostedt's Owlet	H	H	H	**H**
Glaucidium tephronotum	Red-chested Owlet	H	L	L	**L**
Glaucis aeneus	Bronzy Hermit	H	H	H	**H**
Glaucis dohrnii	Hook-billed Hermit	H	H	H	**H**
Glaucis hirsutus	Rufous-breasted Hermit	H	H	H	**H**
Glossopsitta concinna	Musk Lorikeet	H	H	L	**L**
Glossopsitta porphyrocephala	Purple-crowned Lorikeet	H	H	H	**H**
Glossopsitta pusilla	Little Lorikeet	U	L	U	**L**
Glycichaera fallax	Green-backed Honeyeater	U	L	L	**L**
Glyphorynchus spirurus	Wedge-billed Woodcreeper	H	H	H	**H**
Gnorimopsar chopi	Chopi Blackbird	U	L	L	**L**
Goethalsia bella	Rufous-cheeked Hummingbird	H	U	L	**L**
Goldmania violiceps	Violet-capped Hummingbird	H	U	L	**L**
Gorsachius goisagi	Japanese Night-heron	H	H	L	**L**
Gorsachius leuconotus	White-backed Night-heron	L	H	L	**L**
Gorsachius magnificus	White-eared Night-heron	H	H	L	**L**
Gorsachius melanolophus	Malaysian Night-heron	H	H	U	**L**
Goura cristata	Western Crowned-pigeon	H	H	H	**H**
Goura scheepmakeri	Southern Crowned-pigeon	H	H	L	**L**
Goura victoria	Victoria Crowned-pigeon	H	H	H	**H**
Gracula ptilogenys	Sri Lanka Myna	H	H	H	**H**
Gracula religiosa	Hill Myna	U	H	L	**L**
Grafisia torquata	White-collared Starling	U	U	U	**L**
Grallaria albigula	White-throated Antpitta	H	H	H	**H**
Grallaria alleni	Moustached Antpitta	H	H	H	**H**
Grallaria andicolus	Stripe-headed Antpitta	H	U	H	**L**
Grallaria bangsi	Santa Marta Antpitta	H	H	H	**H**
Grallaria blakei	Chestnut Antpitta	H	H	H	**H**
Grallaria capitalis	Bay Antpitta	H	U	H	**L**
Grallaria carrikeri	Pale-billed Antpitta	H	H	H	**H**
Grallaria chthonia	Tachira Antpitta	H	H	H	**H**
Grallaria dignissima	Ochre-striped Antpitta	H	H	H	**H**
Grallaria eludens	Elusive Antpitta	H	H	H	**H**
Grallaria erythroleuca	Red-and-white Antpitta	H	U	H	**L**
Grallaria erythrotis	Rufous-faced Antpitta	U	H	H	**L**
Grallaria excelsa	Great Antpitta	H	H	H	**H**
Grallaria flavotincta	Yellow-breasted Antpitta	U	U	H	**L**
Grallaria gigantea	Giant Antpitta	H	U	H	**L**
Grallaria griseonucha	Grey-naped Antpitta	H	U	H	**L**
Grallaria guatimalensis	Scaled Antpitta	L	H	L	**L**
Grallaria haplonota	Plain-backed Antpitta	H	H	L	**L**
Grallaria hypoleuca	White-bellied Antpitta	H	U	H	**L**
Grallaria kaestneri	Cundinamarca Antpitta	H	H	H	**H**

Grallaria milleri	Brown-banded Antpitta	H	H	H	**H**
Grallaria nuchalis	Chestnut-naped Antpitta	H	H	H	**H**
Grallaria przewalskii	Rusty-tinged Antpitta	H	H	L	**L**
Grallaria quitensis	Tawny Antpitta	U	U	H	**L**
Grallaria ridgelyi	Jocotoco Antpitta	H	H	H	**H**
Grallaria ruficapilla	Chestnut-crowned Antpitta	U	H	H	**L**
Grallaria rufocinerea	Bicoloured Antpitta	H	H	H	**H**
Grallaria rufula	Rufous Antpitta	H	H	H	**H**
Grallaria squamigera	Undulated Antpitta	U	U	L	**L**
Grallaria varia	Variegated Antpitta	H	H	H	**H**
Grallaria watkinsi	Watkins's Antpitta	H	H	H	**H**
Grallaricula cucullata	Hooded Antpitta	H	H	H	**H**
Grallaricula ferrugineipectus	Rusty-breasted Antpitta	H	H	L	**L**
Grallaricula flavirostris	Ochre-breasted Antpitta	H	H	H	**H**
Grallaricula lineifrons	Crescent-faced Antpitta	H	H	H	**H**
Grallaricula loricata	Scallop-breasted Antpitta	H	H	H	**H**
Grallaricula nana	Slate-crowned Antpitta	H	H	H	**H**
Grallaricula ochraceifrons	Ochre-fronted Antpitta	H	H	H	**H**
Grallaricula peruviana	Peruvian Antpitta	H	H	H	**H**
Grallina bruijni	Torrent-lark	U	H	H	**L**
Grallina cyanoleuca	Magpie-lark	H	L	L	**L**
Graminicola bengalensis	Rufous-rumped Grassbird	L	L	H	**L**
Granatellus pelzelni	Rose-breasted Chat	U	U	U	**L**
Granatellus sallaei	Grey-throated Chat	U	U	U	**L**
Granatellus venustus	Red-breasted Chat	H	H	U	**L**
Grandala coelicolor	Grandala	U	H	U	**L**
Grantiella picta	Painted Honeyeater	H	H	L	**L**
Graueria vittata	Grauer's Warbler	H	H	L	**L**
Graydidascalus brachyurus	Short-tailed Parrot	H	U	H	**L**
Grus americana	Whooping Crane	H	H	H	**H**
Grus antigone	Sarus Crane	L	H	H	**L**
Grus canadensis	Sandhill Crane	H	H	L	**L**
Grus carunculatus	Wattled Crane	H	H	L	**L**
Grus grus	Common Crane	H	H	H	**H**
Grus japonensis	Red-crowned Crane	H	H	H	**H**
Grus leucogeranus	Siberian Crane	H	H	H	**H**
Grus monacha	Hooded Crane	H	H	H	**H**
Grus nigricollis	Black-necked Crane	H	H	H	**H**
Grus paradisea	Blue Crane	H	H	H	**H**
Grus rubicunda	Brolga	H	H	L	**L**
Grus vipio	White-naped Crane	H	H	H	**H**
Grus virgo	Demoiselle Crane	H	H	H	**H**
Guadalcanaria	Guadalcanal Honeyeater	H	H	H	**H**

inexpectata					
Guaruba guarouba	Golden Parakeet	H	H	H	**H**
Gubernatrix cristata	Yellow Cardinal	H	U	L	**L**
Gubernetes yetapa	Streamer-tailed Tyrant	U	U	L	**L**
Guira guira	Guira Cuckoo	U	U	L	**L**
Guttera plumifera	Plumed Guineafowl	H	L	H	**L**
Guttera pucherani	Crested Guineafowl	L	L	L	**L**
Gyalophylax hellmayri	Red-shouldered Spinetail	H	U	H	**L**
Gygis alba	Common White Tern	L	H	L	**L**
Gygis microrhyncha	Little White Tern	H	H	U	**L**
Gymnobucco bonapartei	Grey-throated Barbet	H	H	H	**H**
Gymnobucco calvus	Naked-faced Barbet	H	H	H	**H**
Gymnobucco peli	Bristle-nosed Barbet	H	H	L	**L**
Gymnobucco sladeni	Sladen's Barbet	H	H	H	**H**
Gymnocichla nudiceps	Bare-crowned Antbird	H	H	H	**H**
Gymnocrex plumbeiventris	Bare-eyed Rail	U	H	U	**L**
Gymnocrex rosenbergii	Blue-faced Rail	H	U	H	**L**
Gymnocrex talaudensis	Talaud Rail	H	H	H	**H**
Gymnoderus foetidus	Bare-necked Fruitcrow	H	H	H	**H**
Gymnoglaux lawrencii	Bare-legged Owl	U	H	H	**L**
Gymnogyps californianus	California Condor	H	H	L	**L**
Gymnomystax mexicanus	Oriole Blackbird	H	L	H	**L**
Gymnomyza aubryana	Crow Honeyeater	H	U	H	**L**
Gymnomyza samoensis	Mao	H	U	U	**L**
Gymnomyza viridis	Giant Honeyeater	H	H	H	**H**
Gymnophaps albertisii	Papuan Mountain-pigeon	H	H	H	**H**
Gymnophaps mada	Long-tailed Mountain-pigeon	H	H	H	**H**
Gymnophaps solomonensis	Pale Mountain-pigeon	H	H	H	**H**
Gymnopithys leucaspis	Bicoloured Antbird	H	H	H	**H**
Gymnopithys lunulatus	Lunulated Antbird	H	H	H	**H**
Gymnopithys rufigula	Rufous-throated Antbird	H	H	H	**H**
Gymnopithys salvini	White-throated Antbird	H	H	L	**L**
Gymnorhina tibicen	Australian Magpie	H	H	L	**L**
Gymnorhinus cyanocephalus	Pinyon Jay	H	H	H	**H**
Gypaetus barbatus	Lammergeier	H	H	H	**H**
Gypohierax angolensis	Palm-nut Vulture	L	H	L	**L**
Gypopsitta aurantiocephala	Bald Parrot	H	H	U	**L**
Gypopsitta barrabandi	Orange-cheeked Parrot	H	H	H	**H**
Gypopsitta caica	Caica Parrot	H	H	H	**H**
Gypopsitta haematotis	Brown-hooded Parrot	H	H	H	**H**
Gypopsitta pulchra	Rose-faced Parrot	U	H	H	**L**

Gypopsitta pyrilia	Saffron-headed Parrot	H	H	U	**L**
Gypopsitta vulturina	Vulturine Parrot	H	H	U	**L**
Gyps africanus	White-backed Vulture	L	H	L	**L**
Gyps bengalensis	White-rumped Vulture	H	H	L	**L**
Gyps coprotheres	Cape Vulture	H	H	L	**L**
Gyps fulvus	Griffon Vulture	H	H	H	**H**
Gyps himalayensis	Himalayan Vulture	H	H	H	**H**
Gyps indicus	Indian Vulture	H	H	L	**L**
Gyps rueppellii	Rueppell's Vulture	L	H	H	**L**
Gyps tenuirostris	Slender-billed Vulture	H	H	H	**H**
Gypsophila crispifrons	Limestone Wren-babbler	H	H	U	**L**
Habia atrimaxillaris	Black-cheeked Ant-tanager	H	U	H	**L**
Habia cristata	Crested Ant-tanager	U	U	U	**L**
Habia fuscicauda	Red-throated Ant-tanager	U	L	U	**L**
Habia gutturalis	Sooty Ant-tanager	U	H	H	**L**
Habia rubica	Red-crowned Ant-tanager	H	H	U	**L**
Habroptila wallacii	Invisible Rail	H	U	H	**L**
Haematoderus militaris	Crimson Fruitcrow	H	U	H	**L**
Haematopus ater	Blackish Oystercatcher	L	H	H	**L**
Haematopus bachmani	Black Oystercatcher	H	H	H	**H**
Haematopus chathamensis	Chatham Oystercatcher	H	H	H	**H**
Haematopus finschi	South Island Oystercatcher	L	H	H	**L**
Haematopus fuliginosus	Sooty Oystercatcher	H	H	H	**H**
Haematopus leucopodus	Magellanic Oystercatcher	U	H	U	**L**
Haematopus longirostris	Pied Oystercatcher	H	H	H	**H**
Haematopus moquini	African Oystercatcher	H	H	H	**H**
Haematopus ostralegus	Eurasian Oystercatcher	H	H	H	**H**
Haematopus palliatus	American Oystercatcher	L	H	H	**L**
Haematopus unicolor	Variable Oystercatcher	H	H	H	**H**
Haematortyx sanguiniceps	Crimson-headed Partridge	H	L	H	**L**
Haematospiza sipahi	Scarlet Finch	U	L	H	**L**
Halcyon albiventris	Brown-hooded Kingfisher	U	L	L	**L**
Halcyon badia	Chocolate-backed Kingfisher	H	L	H	**L**
Halcyon chelicuti	Striped Kingfisher	H	L	L	**L**
Halcyon coromanda	Ruddy Kingfisher	U	L	L	**L**
Halcyon cyanoventris	Javan Kingfisher	U	L	U	**L**
Halcyon leucocephala	Grey-headed Kingfisher	U	L	L	**L**
Halcyon malimbica	Blue-breasted Kingfisher	H	L	H	**L**
Halcyon pileata	Black-capped Kingfisher	U	L	H	**L**
Halcyon senegalensis	Woodland Kingfisher	H	L	L	**L**
Halcyon senegaloides	Mangrove Kingfisher	H	L	U	**L**
Halcyon smyrnensis	White-throated Kingfisher	U	L	L	**L**
Haliaeetus albicilla	White-tailed Eagle	H	H	H	**H**

Haliaeetus leucocephalus	Bald Eagle	H	H	L	**L**
Haliaeetus leucogaster	White-bellied Sea-eagle	H	H	L	**L**
Haliaeetus leucoryphus	Pallas's Fish-eagle	H	H	L	**L**
Haliaeetus pelagicus	Steller's Sea-eagle	H	H	H	**H**
Haliaeetus sanfordi	Sanford's Sea-eagle	H	H	H	**H**
Haliaeetus vocifer	African Fish-eagle	L	H	L	**L**
Haliaeetus vociferoides	Madagascar Fish-eagle	H	H	L	**L**
Haliastur indus	Brahminy Kite	L	H	L	**L**
Haliastur sphenurus	Whistling Kite	L	H	L	**L**
Halobaena caerulea	Blue Petrel	H	H	L	**L**
Halocyptena microsoma	Least Storm-petrel	L	H	L	**L**
Hamirostra melanosternon	Black-breasted Buzzard	H	H	L	**L**
Hapalopsittaca amazonina	Rusty-faced Parrot	H	H	H	**H**
Hapalopsittaca fuertesi	Indigo-winged Parrot	H	H	H	**H**
Hapalopsittaca melanotis	Black-winged Parrot	H	H	H	**H**
Hapalopsittaca pyrrhops	Red-faced Parrot	H	H	H	**H**
Hapaloptila castanea	White-faced Nunbird	U	H	H	**L**
Haplochelidon andecola	Andean Swallow	H	U	H	**L**
Haplophaedia assimilis	Buff-thighed Puffleg	H	U	H	**L**
Haplophaedia aureliae	Greenish Puffleg	H	H	L	**L**
Haplophaedia lugens	Hoary Puffleg	H	H	H	**H**
Haplospiza rustica	Slaty Finch	H	U	L	**L**
Haplospiza unicolor	Uniform Finch	H	H	L	**L**
Harpactes ardens	Philippine Trogon	H	H	U	**L**
Harpactes diardii	Diard's Trogon	H	H	H	**H**
Harpactes duvaucelii	Scarlet-rumped Trogon	H	H	H	**H**
Harpactes erythrocephalus	Red-headed Trogon	H	H	H	**H**
Harpactes fasciatus	Malabar Trogon	H	H	U	**L**
Harpactes kasumba	Red-naped Trogon	H	H	H	**H**
Harpactes oreskios	Orange-breasted Trogon	H	H	L	**L**
Harpactes orrhophaeus	Cinnamon-rumped Trogon	H	H	H	**H**
Harpactes wardi	Ward's Trogon	H	H	H	**H**
Harpactes whiteheadi	Whitehead's Trogon	H	H	H	**H**
Harpagus bidentatus	Double-toothed Kite	H	H	H	**H**
Harpagus diodon	Rufous-thighed Kite	H	H	L	**L**
Harpia harpyja	Harpy Eagle	H	H	H	**H**
Harpyhaliaetus coronatus	Crowned Eagle	H	H	L	**L**
Harpyhaliaetus solitarius	Solitary Eagle	H	H	L	**L**
Harpyopsis novaeguineae	New Guinea Eagle	H	H	L	**L**
Heinrichia calligyna	Great Shortwing	H	H	H	**H**
Heleia crassirostris	Thick-billed White-eye	H	H	H	**H**
Heleia muelleri	Spot-breasted White-eye	H	H	H	**H**

Heliactin bilophus	Horned Sungem	U	H	L	**L**
Heliangelus amethysticollis	Amethyst-throated Sunangel	H	H	L	**L**
Heliangelus exortis	Tourmaline Sunangel	U	H	H	**L**
Heliangelus mavors	Orange-throated Sunangel	H	H	H	**H**
Heliangelus micraster	Little Sunangel	H	H	H	**H**
Heliangelus regalis	Royal Sunangel	H	H	L	**L**
Heliangelus strophianus	Gorgeted Sunangel	H	H	H	**H**
Heliangelus viola	Purple-throated Sunangel	H	H	H	**H**
Heliangelus zusii	Bogota Sunangel	U	U	H	**L**
Helicolestes hamatus	Slender-billed Kite	H	H	H	**H**
Heliobletus contaminatus	Sharp-billed Treehunter	H	H	L	**L**
Heliodoxa aurescens	Gould's Jewelfront	H	H	L	**L**
Heliodoxa branickii	Rufous-webbed Brilliant	H	U	H	**L**
Heliodoxa gularis	Pink-throated Brilliant	H	U	H	**L**
Heliodoxa imperatrix	Empress Brilliant	H	U	H	**L**
Heliodoxa jacula	Green-crowned Brilliant	H	U	L	**L**
Heliodoxa leadbeateri	Violet-fronted Brilliant	U	H	L	**L**
Heliodoxa rubinoides	Fawn-breasted Brilliant	U	H	H	**L**
Heliodoxa schreibersii	Black-throated Brilliant	U	H	H	**L**
Heliodoxa xanthogonys	Velvet-browed Brilliant	H	H	H	**H**
Heliolais erythropterus	Red-winged Warbler	U	L	H	**L**
Heliomaster constantii	Plain-capped Starthroat	L	U	H	**L**
Heliomaster furcifer	Blue-tufted Starthroat	U	H	L	**L**
Heliomaster longirostris	Long-billed Starthroat	H	H	H	**H**
Heliomaster squamosus	Stripe-breasted Starthroat	U	H	L	**L**
Heliopais personatus	Masked Finfoot	H	H	L	**L**
Heliornis fulica	Sungrebe	H	H	H	**H**
Heliothryx auritus	Black-eared Fairy	H	H	H	**H**
Heliothryx barroti	Purple-crowned Fairy	H	H	H	**H**
Hellmayrea gularis	White-browed Spinetail	H	U	H	**L**
Helmitheros vermivorum	Worm-eating Warbler	L	L	H	**L**
Hemicircus canente	Heart-spotted Woodpecker	H	L	U	**L**
Hemicircus concretus	Grey-and-buff Woodpecker	H	U	U	**L**
Hemignathus flavus	Oahu Amakihi	H	H	H	**H**
Hemignathus kauaiensis	Kauai Amakihi	H	H	H	**H**
Hemignathus lucidus	Nukupuu	H	H	H	**H**
Hemignathus munroi	Akiapolaau	H	H	H	**H**
Hemignathus parvus	Anianiau	H	H	H	**H**
Hemignathus virens	Common Amakihi	U	U	H	**L**
Hemiphaga novaeseelandiae	New Zealand Pigeon	U	H	H	**L**
Hemiprocne comata	Whiskered Treeswift	H	H	L	**L**
Hemiprocne coronata	Crested Treeswift	U	H	L	**L**
Hemiprocne longipennis	Grey-rumped Treeswift	H	H	H	**H**

Hemiprocne mystacea	Moustached Treeswift	U	H	L	**L**
Hemipus hirundinaceus	Black-winged Flycatcher-shrike	H	H	H	**H**
Hemipus picatus	Bar-winged Flycatcher-shrike	U	L	L	**L**
Hemispingus atropileus	Black-capped Hemispingus	H	U	H	**L**
Hemispingus calophrys	Orange-browed Hemispingus	H	U	H	**L**
Hemispingus frontalis	Oleaginous Hemispingus	H	U	H	**L**
Hemispingus goeringi	Slaty-backed Hemispingus	H	U	H	**L**
Hemispingus melanotis	Black-eared Hemispingus	H	U	U	**L**
Hemispingus parodii	Parodi's Hemispingus	H	U	H	**L**
Hemispingus reyi	Grey-capped Hemispingus	H	U	H	**L**
Hemispingus rufosuperciliaris	Rufous-browed Hemispingus	H	H	H	**H**
Hemispingus superciliaris	Superciliaried Hemispingus	H	U	L	**L**
Hemispingus trifasciatus	Three-striped Hemispingus	H	U	H	**L**
Hemispingus verticalis	Black-headed Hemispingus	H	U	H	**L**
Hemispingus xanthophthalmus	Drab Hemispingus	H	H	H	**H**
Hemitesia neumanni	Neumann's Warbler	H	H	H	**H**
Hemithraupis flavicollis	Yellow-backed Tanager	H	U	H	**L**
Hemithraupis guira	Guira Tanager	U	U	L	**L**
Hemithraupis ruficapilla	Rufous-headed Tanager	U	U	L	**L**
Hemitriccus cinnamomeipectus	Cinnamon-breasted Tody-tyrant	H	H	L	**L**
Hemitriccus diops	Drab-breasted Bamboo-tyrant	H	H	L	**L**
Hemitriccus flammulatus	Flammulated Bamboo-tyrant	H	H	H	**H**
Hemitriccus furcatus	Fork-tailed Pygmy-tyrant	H	U	L	**L**
Hemitriccus granadensis	Black-throated Tody-tyrant	U	H	L	**L**
Hemitriccus griseipectus	White-bellied Tody-tyrant	H	H	L	**L**
Hemitriccus inornatus	Pelzeln's Tody-tyrant	H	U	H	**L**
Hemitriccus iohannis	Johannes's Tody-tyrant	H	U	L	**L**
Hemitriccus josephinae	Boat-billed Tody-tyrant	H	H	H	**H**
Hemitriccus kaempferi	Kaempfer's Tody-tyrant	H	H	L	**L**
Hemitriccus margaritaceiventer	Pearly-vented Tody-tyrant	U	H	L	**L**
Hemitriccus minimus	Zimmer's Tody-tyrant	H	U	U	**L**
Hemitriccus minor	Snethlage's Tody-tyrant	H	H	H	**H**
Hemitriccus mirandae	Buff-breasted Tody-tyrant	H	H	L	**L**
Hemitriccus nidipendulus	Hangnest Tody-tyrant	U	H	L	**L**
Hemitriccus obsoletus	Brown-breasted Bamboo-tyrant	H	H	L	**L**
Hemitriccus orbitatus	Eye-ringed Tody-tyrant	H	H	L	**L**
Hemitriccus rufigularis	Buff-throated Tody-tyrant	H	H	H	**H**
Hemitriccus spodiops	Yungas Tody-tyrant	H	H	H	**H**
Hemitriccus striaticollis	Stripe-necked Tody-tyrant	H	H	L	**L**

Hemitriccus zosterops	White-eyed Tody-tyrant	H	H	H	**H**
Hemixos castanonotus	Chestnut Bulbul	U	L	H	**L**
Hemixos flavala	Ashy Bulbul	U	L	U	**L**
Henicopernis infuscatus	Black Honey-buzzard	H	H	H	**H**
Henicopernis longicauda	Long-tailed Honey-buzzard	H	H	L	**L**
Henicophaps albifrons	New Guinea Bronzewing	U	U	L	**L**
Henicophaps foersteri	New Britain Bronzewing	H	H	H	**H**
Henicorhina leucophrys	Grey-breasted Wood-wren	H	H	L	**L**
Henicorhina leucoptera	Bar-winged Wood-wren	H	H	L	**L**
Henicorhina leucosticta	White-breasted Wood-wren	H	H	H	**H**
Henicorhina negreti	Munchique Wood-wren	H	H	H	**H**
Herpetotheres cachinnans	Laughing Falcon	L	H	H	**L**
Herpsilochmus atricapillus	Black-capped Antwren	H	H	L	**L**
Herpsilochmus axillaris	Yellow-breasted Antwren	H	H	H	**H**
Herpsilochmus dorsimaculatus	Spot-backed Antwren	H	U	H	**L**
Herpsilochmus dugandi	Dugand's Antwren	H	H	H	**H**
Herpsilochmus gentryi	Ancient Antwren	H	U	H	**L**
Herpsilochmus longirostris	Large-billed Antwren	H	H	L	**L**
Herpsilochmus motacilloides	Creamy-bellied Antwren	H	H	H	**H**
Herpsilochmus parkeri	Ash-throated Antwren	H	H	H	**H**
Herpsilochmus pectoralis	Pectoral Antwren	H	U	L	**L**
Herpsilochmus pileatus	Bahia Antwren	H	H	L	**L**
Herpsilochmus roraimae	Roraiman Antwren	U	U	H	**L**
Herpsilochmus rufimarginatus	Rufous-winged Antwren	H	U	L	**L**
Herpsilochmus sellowi	Caatinga Antwren	U	U	L	**L**
Herpsilochmus stictocephalus	Todd's Antwren	H	H	H	**H**
Herpsilochmus sticturus	Spot-tailed Antwren	H	H	H	**H**
Heterocercus aurantiivertex	Orange-crested Manakin	H	H	H	**H**
Heterocercus flavivertex	Yellow-crested Manakin	H	U	H	**L**
Heterocercus linteatus	Flame-crested Manakin	H	H	H	**H**
Heteroglaux blewitti	Forest Owlet	H	H	L	**L**
Heteromirafra archeri	Archer's Lark	H	H	H	**H**
Heteromirafra ruddi	Rudd's Lark	H	H	L	**L**
Heteromirafra sidamoensis	Sidamo Lark	H	H	H	**H**
Heteromunia pectoralis	Pictorella Munia	U	L	U	**L**
Heteromyias albispecularis	Grey-headed Robin	H	H	U	**L**
Heteronetta atricapilla	Black-headed Duck	H	H	L	**L**
Heterophasia annectens	Rufous-backed Sibia	H	H	U	**L**

Heterophasia auricularis	White-eared Sibia	H	H	H	**H**
Heterophasia capistrata	Rufous Sibia	U	L	U	**L**
Heterophasia desgodinsi	Black-headed Sibia	H	H	U	**L**
Heterophasia gracilis	Grey Sibia	H	H	H	**H**
Heterophasia melanoleuca	Dark-backed Sibia	H	H	U	**L**
Heterophasia picaoides	Long-tailed Sibia	U	L	U	**L**
Heterophasia pulchella	Beautiful Sibia	H	H	H	**H**
Heteroscelus brevipes	Grey-tailed Tattler	U	H	U	**L**
Heteroscelus incanus	Wandering Tattler	H	H	L	**L**
Heterospingus rubrifrons	Sulphur-rumped Tanager	H	U	H	**L**
Heterospingus xanthopygius	Scarlet-browed Tanager	H	U	H	**L**
Hieraaetus ayresii	Ayres's Hawk-eagle	H	H	L	**L**
Hieraaetus fasciatus	Bonelli's Eagle	H	H	L	**L**
Hieraaetus kienerii	Rufous-bellied Eagle	H	H	L	**L**
Hieraaetus morphnoides	Little Eagle	U	H	U	**L**
Hieraaetus pennatus	Booted Eagle	H	H	L	**L**
Hieraaetus spilogaster	African Hawk-eagle	L	H	L	**L**
Hieraaetus weiskei	New Guinea Hawk-eagle	H	H	L	**L**
Himantopus himantopus	Black-winged Stilt	L	H	L	**L**
Himantopus leucocephalus	White-headed Stilt	U	H	U	**L**
Himantopus mexicanus	Black-necked Stilt	L	H	L	**L**
Himantopus novaezelandiae	Black Stilt	H	H	H	**H**
Himantornis haematopus	Nkulengu Rail	H	L	L	**L**
Himatione sanguinea	Apapane	H	H	H	**H**
Hippolais caligata	Booted Warbler	H	L	H	**L**
Hippolais icterina	Icterine Warbler	L	L	H	**L**
Hippolais languida	Upcher's Warbler	H	L	H	**L**
Hippolais olivetorum	Olive-tree Warbler	L	L	L	**L**
Hippolais opaca	Western Olivaceous Warbler	U	U	H	**L**
Hippolais pallida	Eastern Olivaceous Warbler	U	L	H	**L**
Hippolais polyglotta	Melodious Warbler	L	L	H	**L**
Hippolais rama	Sykes's Warbler	U	U	U	**L**
Hirundapus caudacutus	White-throated Needletail	H	H	H	**H**
Hirundapus celebensis	Purple Needletail	U	H	U	**L**
Hirundapus cochinchinensis	Silver-backed Needletail	H	H	L	**L**
Hirundapus giganteus	Brown-backed Needletail	H	H	L	**L**
Hirundinea ferruginea	Cliff Flycatcher	H	H	L	**L**
Hirundo abyssinica	Lesser Striped-swallow	U	L	U	**L**
Hirundo aethiopica	Ethiopian Swallow	U	L	U	**L**
Hirundo albigularis	White-throated Swallow	U	L	U	**L**
Hirundo angolensis	Angola Swallow	U	L	L	**L**

Hirundo ariel	Fairy Martin	U	L	U	**L**
Hirundo atrocaerulea	Blue Swallow	H	L	L	**L**
Hirundo concolor	Dusky Crag-martin	U	L	U	**L**
Hirundo cucullata	Greater Striped-swallow	U	L	L	**L**
Hirundo daurica	Red-rumped Swallow	L	L	H	**L**
Hirundo dimidiata	Pearl-breasted Swallow	U	L	U	**L**
Hirundo domicola	Hill Swallow	U	L	U	**L**
Hirundo fluvicola	Streak-throated Swallow	U	L	U	**L**
Hirundo fuliginosa	Forest Swallow	H	L	H	**L**
Hirundo fuligula	Rock Martin	U	L	L	**L**
Hirundo leucosoma	Pied-winged Swallow	U	L	H	**L**
Hirundo lucida	Red-chested Swallow	U	L	H	**L**
Hirundo megaensis	White-tailed Swallow	H	U	H	**L**
Hirundo neoxena	Welcome Swallow	H	L	L	**L**
Hirundo nigricans	Tree Martin	U	L	L	**L**
Hirundo nigrita	White-throated Blue Swallow	H	L	L	**L**
Hirundo nigrorufa	Black-and-rufous Swallow	U	L	U	**L**
Hirundo obsoleta	Pale Crag-martin	H	L	H	**L**
Hirundo perdita	Red Sea Swallow	H	U	H	**L**
Hirundo preussi	Preuss's Swallow	U	L	U	**L**
Hirundo rufigula	Red-throated Swallow	U	H	U	**L**
Hirundo rupestris	Eurasian Crag-martin	H	L	H	**L**
Hirundo rustica	Barn Swallow	L	L	H	**L**
Hirundo semirufa	Rufous-chested Swallow	U	L	U	**L**
Hirundo senegalensis	Mosque Swallow	U	L	L	**L**
Hirundo smithii	Wire-tailed Swallow	U	L	L	**L**
Hirundo spilodera	South African Swallow	U	L	L	**L**
Hirundo striolata	Striated Swallow	U	L	U	**L**
Hirundo tahitica	Pacific Swallow	U	L	U	**L**
Histrionicus histrionicus	Harlequin Duck	H	H	H	**H**
Histurgops ruficaudus	Rufous-tailed Weaver	H	U	H	**L**
Hodgsonius phaenicuroides	White-bellied Redstart	U	L	U	**L**
Horizorhinus dohrni	Principe Thrush-babbler	H	H	L	**L**
Houbaropsis bengalensis	Bengal Florican	H	H	H	**H**
Humblotia flavirostris	Grand Comoro Flycatcher	H	H	H	**H**
Hydrobates pelagicus	European Storm-petrel	H	H	H	**H**
Hydrochous gigas	Waterfall Swift	H	H	L	**L**
Hydrophasianus chirurgus	Pheasant-tailed Jacana	L	L	L	**L**
Hydropsalis climacocerca	Ladder-tailed Nightjar	H	H	H	**H**
Hydropsalis torquata	Scissor-tailed Nightjar	U	H	L	**L**
Hylacola cauta	Shy Heathwren	U	H	U	**L**
Hylacola pyrrhopygia	Chestnut-rumped Heathwren	U	H	U	**L**

Hylexetastes brigidai	Mato Grosso Woodcreeper	H	H	H	**H**
Hylexetastes perrotii	Red-billed Woodcreeper	H	H	H	**H**
Hylexetastes stresemanni	Bar-bellied Woodcreeper	H	H	L	**L**
Hylexetastes uniformis	Uniform Woodcreeper	H	H	H	**H**
Hylia prasina	Green Hylia	U	H	L	**L**
Hyliota australis	Southern Hyliota	U	L	U	**L**
Hyliota flavigaster	Yellow-bellied Hyliota	U	H	U	**L**
Hyliota usambara	Usambara Hyliota	H	U	H	**L**
Hyliota violacea	Violet-backed Hyliota	H	U	H	**L**
Hylocharis chrysura	Gilded Hummingbird	U	H	L	**L**
Hylocharis cyanus	White-chinned Sapphire	U	H	H	**L**
Hylocharis eliciae	Blue-throated Goldentail	L	H	H	**L**
Hylocharis grayi	Blue-headed Sapphire	U	U	H	**L**
Hylocharis humboldtii	Humboldt's Sapphire	H	U	H	**L**
Hylocharis leucotis	White-eared Hummingbird	H	H	L	**L**
Hylocharis pyropygia	Flame-rumped Sapphire	U	U	U	**L**
Hylocharis sapphirina	Rufous-throated Sapphire	H	H	H	**H**
Hylocharis xantusii	Xantus's Hummingbird	H	H	H	**H**
Hylocichla mustelina	Wood Thrush	H	L	H	**L**
Hylocitrea bonensis	Olive-flanked Whistler	H	H	H	**H**
Hylocryptus erythrocephalus	Henna-hooded Foliage-gleaner	H	U	H	**L**
Hylocryptus rectirostris	Chestnut-capped Foliage-gleaner	H	H	L	**L**
Hyloctistes subulatus	Striped Woodhaunter	H	H	H	**H**
Hylomanes momotula	Tody Motmot	H	U	H	**L**
Hylonympha macrocerca	Scissor-tailed Hummingbird	H	H	H	**H**
Hylopezus auricularis	Masked Antpitta	H	H	L	**L**
Hylopezus berlepschi	Amazonian Antpitta	H	U	L	**L**
Hylopezus dives	Thicket Antpitta	H	U	H	**L**
Hylopezus fulviventris	White-lored Antpitta	H	U	H	**L**
Hylopezus macularius	Spotted Antpitta	H	H	H	**H**
Hylopezus nattereri	Speckle-breasted Antpitta	H	U	L	**L**
Hylopezus ochroleucus	White-browed Antpitta	H	U	L	**L**
Hylopezus perspicillatus	Spectacled Antpitta	H	H	H	**H**
Hylophilus amaurocephalus	Grey-eyed Greenlet	U	U	L	**L**
Hylophilus aurantiifrons	Golden-fronted Greenlet	U	U	H	**L**
Hylophilus brunneiceps	Brown-headed Greenlet	H	U	H	**L**
Hylophilus decurtatus	Lesser Greenlet	L	H	H	**L**
Hylophilus flavipes	Scrub Greenlet	U	L	L	**L**
Hylophilus hypoxanthus	Dusky-capped Greenlet	H	H	L	**L**
Hylophilus muscicapinus	Buff-cheeked Greenlet	H	H	H	**H**
Hylophilus ochraceiceps	Tawny-crowned Greenlet	H	H	H	**H**
Hylophilus olivaceus	Olivaceous Greenlet	U	U	H	**L**

Hylophilus pectoralis	Ashy-headed Greenlet	H	U	H	**L**
Hylophilus poicilotis	Rufous-crowned Greenlet	H	U	L	**L**
Hylophilus sclateri	Tepui Greenlet	U	U	H	**L**
Hylophilus semibrunneus	Rufous-naped Greenlet	U	U	H	**L**
Hylophilus semicinereus	Grey-chested Greenlet	H	U	H	**L**
Hylophilus thoracicus	Lemon-chested Greenlet	H	U	H	**L**
Hylophylax naevioides	Spotted Antbird	H	H	L	**L**
Hylophylax naevius	Spot-backed Antbird	H	H	H	**H**
Hylophylax punctulatus	Dot-backed Antbird	H	H	H	**H**
Hylorchilus navai	Nava's Wren	H	H	L	**L**
Hylorchilus sumichrasti	Sumichrast's Wren	H	H	H	**H**
Hymenolaimus malacorhynchos	Blue Duck	H	H	H	**H**
Hymenops perspicillatus	Spectacled Tyrant	H	L	L	**L**
Hypargos margaritatus	Pink-throated Twinspot	H	L	L	**L**
Hypargos niveoguttatus	Peters's Twinspot	U	L	U	**L**
Hypergerus atriceps	Oriole Warbler	U	L	H	**L**
Hypnelus ruficollis	Russet-throated Puffbird	H	L	H	**L**
Hypocnemis cantator	Warbling Antbird	H	H	U	**L**
Hypocnemis hypoxantha	Yellow-browed Antbird	H	U	H	**L**
Hypocnemoides maculicauda	Band-tailed Antbird	H	H	L	**L**
Hypocnemoides melanopogon	Black-chinned Antbird	H	H	H	**H**
Hypocolius ampelinus	Grey Hypocolius	H	L	H	**L**
Hypocryptadius cinnamomeus	Cinnamon Ibon	H	H	H	**H**
Hypoedaleus guttatus	Spot-backed Antshrike	H	H	L	**L**
Hypogramma hypogrammicum	Purple-naped Sunbird	U	L	U	**L**
Hypopyrrhus pyrohypogaster	Red-bellied Grackle	H	H	H	**H**
Hypositta corallirostris	Nuthatch Vanga	H	H	H	**H**
Hypositta perdita	Bluntschli's Vanga	U	U	U	**L**
Hypothymis azurea	Black-naped Monarch	H	L	L	**L**
Hypothymis coelestis	Celestial Monarch	H	U	L	**L**
Hypothymis helenae	Short-crested Monarch	H	H	H	**H**
Hypsipetes borbonicus	Olivaceous Bulbul	H	H	U	**L**
Hypsipetes crassirostris	Seychelles Bulbul	H	H	L	**L**
Hypsipetes leucocephalus	Asian Black Bulbul	U	L	H	**L**
Hypsipetes madagascariensis	Madagascar Black Bulbul	U	L	L	**L**
Hypsipetes mcclellandii	Mountain Bulbul	U	H	U	**L**
Hypsipetes nicobariensis	Nicobar Bulbul	H	H	H	**H**
Hypsipetes olivaceus	Mauritius Black Bulbul	H	H	H	**H**
Hypsipetes parvirostris	Comoro Bulbul	U	H	H	**L**

Hypsipetes thompsoni	White-headed Bulbul	U	L	U	**L**
Hypsipetes virescens	Green-winged Bulbul	U	U	H	**L**
Ibidorhyncha struthersii	Ibisbill	H	H	H	**H**
Ibycter americanus	Red-throated Caracara	H	H	H	**H**
Ichthyophaga humilis	Lesser Fish-eagle	H	H	L	**L**
Ichthyophaga ichthyaetus	Grey-headed Fish-eagle	H	H	L	**L**
Icteria virens	Yellow-breasted Chat	L	L	H	**L**
Icterus abeillei	Black-backed Oriole	L	U	H	**L**
Icterus auratus	Orange Oriole	H	U	H	**L**
Icterus auricapillus	Orange-crowned Oriole	U	U	L	**L**
Icterus bonana	Martinique Oriole	H	L	H	**L**
Icterus bullockii	Bullock's Oriole	H	L	H	**L**
Icterus cayanensis	Epaulet Oriole	U	L	L	**L**
Icterus chrysater	Yellow-backed Oriole	L	L	H	**L**
Icterus croconotus	Orange-backed Troupial	H	U	L	**L**
Icterus cucullatus	Hooded Oriole	L	L	H	**L**
Icterus dominicensis	Greater Antillean Oriole	U	L	H	**L**
Icterus galbula	Baltimore Oriole	L	L	L	**L**
Icterus graceannae	White-edged Oriole	H	H	H	**H**
Icterus graduacauda	Audubon's Oriole	L	L	L	**L**
Icterus gularis	Altamira Oriole	L	L	H	**L**
Icterus icterus	Venezuelan Troupial	H	L	H	**L**
Icterus jamacaii	Campo Troupial	H	U	L	**L**
Icterus laudabilis	St Lucia Oriole	H	H	H	**H**
Icterus leucopteryx	Jamaican Oriole	U	L	H	**L**
Icterus maculialatus	Bar-winged Oriole	L	U	H	**L**
Icterus mesomelas	Yellow-tailed Oriole	L	L	H	**L**
Icterus nigrogularis	Yellow Oriole	H	L	H	**L**
Icterus oberi	Montserrat Oriole	H	H	H	**H**
Icterus parisorum	Scott's Oriole	H	L	H	**L**
Icterus pectoralis	Spot-breasted Oriole	L	L	H	**L**
Icterus prosthemelas	Black-cowled Oriole	H	L	H	**L**
Icterus pustulatus	Streak-backed Oriole	L	L	L	**L**
Icterus spurius	Orchard Oriole	L	L	L	**L**
Icterus wagleri	Black-vented Oriole	L	L	L	**L**
Ictinaetus malayensis	Black Eagle	H	H	L	**L**
Ictinia mississippiensis	Mississippi Kite	L	H	L	**L**
Ictinia plumbea	Plumbeous Kite	L	H	L	**L**
Idiopsar brachyurus	Short-tailed Finch	H	U	H	**L**
Ifrita kowaldi	Ifrit	H	H	U	**L**
Ilicura militaris	Pin-tailed Manakin	H	H	L	**L**
Illadopsis albipectus	Scaly-breasted Illadopsis	H	H	L	**L**
Illadopsis cleaveri	Blackcap Illadopsis	H	H	L	**L**
Illadopsis fulvescens	Brown Illadopsis	H	H	L	**L**
Illadopsis puveli	Puvel's Illadopsis	H	H	L	**L**

Illadopsis pyrrhoptera	Mountain Illadopsis	H	H	L	**L**
Illadopsis rufescens	Rufous-winged Illadopsis	H	H	L	**L**
Illadopsis rufipennis	Pale-breasted Illadopsis	U	H	H	**L**
Incana incana	Socotra Warbler	H	U	H	**L**
Incaspiza laeta	Buff-bridled Inca-finch	H	U	H	**L**
Incaspiza ortizi	Grey-winged Inca-finch	H	U	H	**L**
Incaspiza personata	Rufous-backed Inca-finch	H	U	H	**L**
Incaspiza pulchra	Great Inca-finch	H	U	H	**L**
Incaspiza watkinsi	Little Inca-finch	H	U	H	**L**
Indicator archipelagicus	Malaysian Honeyguide	H	U	H	**L**
Indicator exilis	Least Honeyguide	H	U	L	**L**
Indicator indicator	Greater Honeyguide	H	U	L	**L**
Indicator maculatus	Spotted Honeyguide	H	U	H	**L**
Indicator meliphilus	Pallid Honeyguide	U	U	L	**L**
Indicator minor	Lesser Honeyguide	H	L	U	**L**
Indicator pumilio	Dwarf Honeyguide	H	U	L	**L**
Indicator variegatus	Scaly-throated Honeyguide	H	U	U	**L**
Indicator willcocksi	Willcocks's Honeyguide	H	U	H	**L**
Indicator xanthonotus	Yellow-rumped Honeyguide	H	U	H	**L**
Inezia caudata	Pale-tipped Tyrannulet	H	U	H	**L**
Inezia inornata	Plain Tyrannulet	U	U	L	**L**
Inezia subflava	Amazonian Tyrannulet	H	U	H	**L**
Inezia tenuirostris	Slender-billed Tyrannulet	U	U	H	**L**
Iodopleura fusca	Dusky Purpletuft	H	H	U	**L**
Iodopleura isabellae	White-browed Purpletuft	H	H	U	**L**
Iodopleura pipra	Buff-throated Purpletuft	H	H	H	**H**
Iole indica	Yellow-browed Bulbul	U	L	U	**L**
Iole olivacea	Buff-vented Bulbul	H	H	H	**H**
Iole propinqua	Grey-eyed Bulbul	U	U	U	**L**
Iole virescens	Olive Bulbul	H	H	U	**L**
Irania gutturalis	White-throated Robin	H	L	L	**L**
Irediparra gallinacea	Comb-crested Jacana	L	L	L	**L**
Irena cyanogastra	Philippine Fairy-bluebird	H	H	L	**L**
Irena puella	Asian Fairy-bluebird	H	H	L	**L**
Iridophanes pulcherrimus	Golden-collared Honeycreeper	U	H	H	**L**
Iridosornis analis	Yellow-throated Tanager	H	U	L	**L**
Iridosornis jelskii	Golden-collared Tanager	H	U	H	**L**
Iridosornis porphyrocephalus	Purplish-mantled Tanager	H	U	H	**L**
Iridosornis reinhardti	Yellow-scarfed Tanager	H	U	L	**L**
Iridosornis rufivertex	Golden-crowned Tanager	H	U	H	**L**
Ithaginis cruentus	Blood Pheasant	U	L	U	**L**
Ixobrychus cinnamomeus	Cinnamon Bittern	U	L	U	**L**
Ixobrychus eurhythmus	Schrenck's Bittern	H	L	H	**L**

Ixobrychus exilis	Least Bittern	L	L	L	**L**
Ixobrychus flavicollis	Black Bittern	L	L	L	**L**
Ixobrychus involucris	Stripe-backed Bittern	L	L	L	**L**
Ixobrychus minutus	Little Bittern	H	L	H	**L**
Ixobrychus sinensis	Yellow Bittern	U	L	U	**L**
Ixobrychus sturmii	Dwarf Bittern	L	L	L	**L**
Ixonotus guttatus	Spotted Greenbul	H	H	L	**L**
Ixos amaurotis	Brown-eared Bulbul	U	L	H	**L**
Ixos everetti	Yellowish Bulbul	H	U	H	**L**
Ixos malaccensis	Streaked Bulbul	H	H	H	**H**
Ixos palawanensis	Sulphur-bellied Bulbul	H	H	H	**H**
Ixos philippinus	Philippine Bulbul	U	L	U	**L**
Ixos rufigularis	Zamboanga Bulbul	H	H	H	**H**
Ixos siquijorensis	Streak-breasted Bulbul	H	H	H	**H**
Jabiru mycteria	Jabiru	H	H	L	**L**
Jabouilleia danjoui	Short-tailed Scimitar-babbler	H	H	L	**L**
Jacamaralcyon tridactyla	Three-toed Jacamar	H	H	L	**L**
Jacamerops aureus	Great Jacamar	H	H	H	**H**
Jacana jacana	Wattled Jacana	H	L	L	**L**
Jacana spinosa	Northern Jacana	H	L	H	**L**
Jubula lettii	Maned Owl	H	U	L	**L**
Junco hyemalis	Dark-eyed Junco	H	L	L	**L**
Junco phaeonotus	Yellow-eyed Junco	L	L	L	**L**
Junco vulcani	Volcano Junco	U	H	H	**L**
Jynx ruficollis	Rufous-necked Wryneck	H	L	L	**L**
Jynx torquilla	Eurasian Wryneck	H	L	H	**L**
Kakamega poliothorax	Grey-chested Babbler	H	H	L	**L**
Kaupifalco monogrammicus	Lizard Buzzard	L	H	L	**L**
Kenopia striata	Striped Wren-babbler	H	H	H	**H**
Ketupa blakistoni	Blakiston's Fish-owl	H	H	H	**H**
Ketupa flavipes	Tawny Fish-owl	U	H	U	**L**
Ketupa ketupu	Buffy Fish-owl	U	H	U	**L**
Ketupa zeylonensis	Brown Fish-owl	U	H	H	**L**
Klais guimeti	Violet-headed Hummingbird	H	H	H	**H**
Knipolegus aterrimus	White-winged Black-tyrant	U	L	U	**L**
Knipolegus cyanirostris	Blue-billed Black-tyrant	H	H	L	**L**
Knipolegus franciscanus	Brazilian Black-tyrant	H	U	H	**L**
Knipolegus hudsoni	Hudson's Black-tyrant	H	U	L	**L**
Knipolegus lophotes	Crested Black-tyrant	U	U	L	**L**
Knipolegus nigerrimus	Velvety Black-tyrant	U	U	L	**L**
Knipolegus orenocensis	Riverside Tyrant	H	U	H	**L**
Knipolegus poecilocercus	Amazonian Black-tyrant	H	U	H	**L**
Knipolegus poecilurus	Rufous-tailed Tyrant	U	U	L	**L**
Knipolegus signatus	Andean Tyrant	H	H	H	**H**

Knipolegus striaticeps	Cinereous Tyrant	H	H	L	**L**
Kupeornis chapini	Chapin's Mountain-babbler	H	H	H	**H**
Kupeornis gilberti	White-throated Mountain-babbler	H	H	H	**H**
Kupeornis rufocinctus	Red-collared Mountain-babbler	H	H	H	**H**
Lacedo pulchella	Banded Kingfisher	H	L	L	**L**
Lafresnaya lafresnayi	Mountain Velvetbreast	U	H	H	**L**
Lagonosticta landanae	Pale-billed Firefinch	U	U	U	**L**
Lagonosticta larvata	Black-throated Firefinch	U	U	U	**L**
Lagonosticta nitidula	Brown Firefinch	U	L	H	**L**
Lagonosticta rara	Black-bellied Firefinch	U	L	U	**L**
Lagonosticta rhodopareia	Jameson's Firefinch	U	U	U	**L**
Lagonosticta rubricata	African Firefinch	U	L	L	**L**
Lagonosticta rufopicta	Bar-breasted Firefinch	U	L	H	**L**
Lagonosticta sanguinodorsalis	Rock Firefinch	U	U	L	**L**
Lagonosticta senegala	Red-billed Firefinch	U	H	L	**L**
Lagonosticta virata	Mali Firefinch	U	L	H	**L**
Lagopus lagopus	Willow Ptarmigan	H	H	H	**H**
Lagopus leucura	White-tailed Ptarmigan	H	L	L	**L**
Lagopus muta	Rock Ptarmigan	H	H	L	**L**
Lalage atrovirens	Black-browed Triller	U	U	L	**L**
Lalage aurea	Rufous-bellied Triller	H	U	H	**L**
Lalage leucomela	Varied Triller	U	H	L	**L**
Lalage leucopyga	Long-tailed Triller	U	H	L	**L**
Lalage leucopygialis	White-rumped Triller	U	U	H	**L**
Lalage maculosa	Polynesian Triller	H	H	L	**L**
Lalage melanoleuca	Black-and-white Triller	H	H	L	**L**
Lalage moesta	White-browed Triller	H	U	L	**L**
Lalage nigra	Pied Triller	H	L	L	**L**
Lalage sharpei	Samoan Triller	H	H	U	**L**
Lalage sueurii	White-shouldered Triller	U	H	H	**L**
Lalage tricolor	White-winged Triller	H	H	L	**L**
Lampornis amethystinus	Amethyst-throated Hummingbird	H	H	H	**H**
Lampornis calolaemus	Purple-throated Mountain-gem	U	H	H	**L**
Lampornis castaneoventris	White-throated Mountain-gem	U	H	H	**L**
Lampornis clemenciae	Blue-throated Hummingbird	H	H	L	**L**
Lampornis hemileucus	White-bellied Mountain-gem	H	U	H	**L**
Lampornis sybillae	Green-breasted Mountain-gem	H	U	H	**L**
Lampornis viridipallens	Green-throated Mountain-gem	H	U	H	**L**

Lamprolaima rhami	Garnet-throated Hummingbird	H	H	H	**H**
Lamprolia victoriae	Silktail	H	H	L	**L**
Lampropsar tanagrinus	Velvet-fronted Grackle	H	U	L	**L**
Lamprospiza melanoleuca	Red-billed Pied Tanager	H	H	H	**H**
Lamprotornis acuticaudus	Sharp-tailed Glossy-starling	U	L	U	**L**
Lamprotornis australis	Burchell's Glossy-starling	H	L	L	**L**
Lamprotornis caudatus	Long-tailed Glossy-starling	U	L	U	**L**
Lamprotornis chalcurus	Bronze-tailed Glossy-starling	U	L	U	**L**
Lamprotornis chalybaeus	Greater Blue-eared Glossy-starling	U	L	U	**L**
Lamprotornis chloropterus	Lesser Blue-eared Glossy-starling	U	L	U	**L**
Lamprotornis corruscus	Black-bellied Glossy-starling	H	L	L	**L**
Lamprotornis cupreocauda	Copper-tailed Glossy-starling	H	L	L	**L**
Lamprotornis elisabeth	Southern Blue-eared Glossy-starling	U	L	U	**L**
Lamprotornis hildebrandti	Hildebrandt's Starling	U	L	U	**L**
Lamprotornis mevesii	Meves's Glossy-starling	U	L	H	**L**
Lamprotornis nitens	Red-shouldered Glossy-starling	H	L	L	**L**
Lamprotornis ornatus	Principe Glossy-starling	H	H	L	**L**
Lamprotornis pulcher	Chestnut-bellied Starling	U	L	U	**L**
Lamprotornis purpureiceps	Purple-headed Glossy-starling	H	L	H	**L**
Lamprotornis purpureus	Purple Glossy-starling	U	L	U	**L**
Lamprotornis purpuroptera	Rueppell's Glossy-starling	U	L	U	**L**
Lamprotornis shelleyi	Shelley's Starling	U	L	U	**L**
Lamprotornis splendidus	Splendid Glossy-starling	U	H	L	**L**
Lamprotornis superbus	Superb Starling	H	L	L	**L**
Laniarius aethiopicus	Tropical Boubou	U	L	L	**L**
Laniarius amboimensis	Gabela Bush-shrike	H	H	H	**H**
Laniarius atrococcineus	Crimson-breasted Gonolek	H	L	L	**L**
Laniarius atroflavus	Yellow-breasted Boubou	U	U	H	**L**
Laniarius barbarus	Common Gonolek	U	H	U	**L**
Laniarius bicolor	Gabon Boubou	U	H	U	**L**
Laniarius brauni	Orange-breasted Bush-shrike	H	H	H	**H**
Laniarius erythrogaster	Black-headed Gonolek	U	H	U	**L**
Laniarius ferrugineus	Southern Boubou	H	H	L	**L**
Laniarius fuelleborni	Fuelleborn's Boubou	H	H	L	**L**
Laniarius funebris	Slate-coloured Boubou	U	H	U	**L**
Laniarius leucorhynchus	Sooty Boubou	U	H	U	**L**
Laniarius liberatus	Bulo Burti Boubou	H	H	U	**L**
Laniarius luehderi	Luehder's Bush-shrike	U	H	H	**L**

Laniarius mufumbiri	Papyrus Gonolek	H	U	H	**L**
Laniarius poensis	Mountain Boubou	H	H	L	**L**
Laniarius ruficeps	Red-naped Bush-shrike	U	L	U	**L**
Laniarius turatii	Turati's Boubou	U	H	U	**L**
Laniisoma elegans	Shrike-like Cotinga	H	H	L	**L**
Lanio aurantius	Black-throated Shrike-tanager	H	H	H	**H**
Lanio fulvus	Fulvous Shrike-tanager	H	U	H	**L**
Lanio leucothorax	White-throated Shrike-tanager	H	H	H	**H**
Lanio versicolor	White-winged Shrike-tanager	H	H	L	**L**
Laniocera hypopyrra	Cinereous Mourner	U	H	U	**L**
Laniocera rufescens	Speckled Mourner	H	H	U	**L**
Lanioturdus torquatus	Chatshrike	H	H	L	**L**
Lanius bucephalus	Bull-headed Shrike	U	L	H	**L**
Lanius cabanisi	Long-tailed Fiscal	U	L	U	**L**
Lanius collaris	Common Fiscal	U	L	L	**L**
Lanius collurio	Red-backed Shrike	H	L	H	**L**
Lanius collurioides	Burmese Shrike	U	L	U	**L**
Lanius cristatus	Brown Shrike	U	L	U	**L**
Lanius dorsalis	Taita Fiscal	U	L	U	**L**
Lanius excubitor	Great Grey Shrike	H	L	H	**L**
Lanius excubitoroides	Grey-backed Fiscal	U	L	U	**L**
Lanius gubernator	Emin's Shrike	U	U	U	**L**
Lanius isabellinus	Rufous-tailed Shrike	U	L	U	**L**
Lanius ludovicianus	Loggerhead Shrike	H	H	H	**H**
Lanius mackinnoni	Mackinnon's Shrike	U	L	L	**L**
Lanius marwitzi	Uhehe Fiscal	U	L	L	**L**
Lanius minor	Lesser Grey Shrike	H	L	H	**L**
Lanius newtoni	Sao Tome Fiscal	H	H	L	**L**
Lanius nubicus	Masked Shrike	H	L	H	**L**
Lanius schach	Long-tailed Shrike	U	L	H	**L**
Lanius senator	Woodchat Shrike	L	L	H	**L**
Lanius somalicus	Somali Fiscal	U	L	U	**L**
Lanius souzae	Souza's Shrike	H	L	U	**L**
Lanius sphenocercus	Chinese Grey Shrike	U	L	U	**L**
Lanius tephronotus	Grey-backed Shrike	U	L	U	**L**
Lanius tigrinus	Tiger Shrike	U	L	H	**L**
Lanius validirostris	Mountain Shrike	U	H	H	**L**
Lanius vittatus	Bay-backed Shrike	U	L	U	**L**
Larosterna inca	Inca Tern	H	H	H	**H**
Larus argentatus	Herring Gull	H	H	H	**H**
Larus armenicus	Armenian Gull	H	H	H	**H**
Larus atlanticus	Olrog's Gull	H	H	H	**H**

Larus atricilla	Laughing Gull	L	H	H	**L**
Larus audouinii	Audouin's Gull	H	H	H	**H**
Larus belcheri	Band-tailed Gull	H	H	H	**H**
Larus brunnicephalus	Brown-headed Gull	L	H	H	**L**
Larus bulleri	Black-billed Gull	L	H	H	**L**
Larus cachinnans	Yellow-legged Gull	H	H	L	**L**
Larus californicus	California Gull	L	H	H	**L**
Larus canus	Mew Gull	H	H	L	**L**
Larus cirrocephalus	Grey-headed Gull	L	H	L	**L**
Larus crassirostris	Black-tailed Gull	L	H	H	**L**
Larus delawarensis	Ring-billed Gull	H	H	L	**L**
Larus dominicanus	Kelp Gull	L	H	H	**L**
Larus fuliginosus	Lava Gull	H	H	H	**H**
Larus fuscus	Lesser Black-backed Gull	L	H	L	**L**
Larus genei	Slender-billed Gull	H	H	H	**H**
Larus glaucescens	Glaucous-winged Gull	H	H	L	**L**
Larus glaucoides	Iceland Gull	H	H	H	**H**
Larus hartlaubii	King Gull	H	H	H	**H**
Larus heermanni	Heermann's Gull	L	H	H	**L**
Larus hemprichii	Sooty Gull	H	H	H	**H**
Larus hyperboreus	Glaucous Gull	H	H	H	**H**
Larus ichthyaetus	Pallas's Gull	H	H	L	**L**
Larus leucophthalmus	White-eyed Gull	H	H	H	**H**
Larus livens	Yellow-footed Gull	H	H	H	**H**
Larus maculipennis	Brown-hooded Gull	L	H	L	**L**
Larus marinus	Great Black-backed Gull	H	H	H	**H**
Larus melanocephalus	Mediterranean Gull	H	H	H	**H**
Larus minutus	Little Gull	H	H	H	**H**
Larus modestus	Grey Gull	L	H	H	**L**
Larus novaehollandiae	Silver Gull	L	H	L	**L**
Larus occidentalis	Western Gull	L	H	H	**L**
Larus pacificus	Pacific Gull	H	H	H	**H**
Larus philadelphia	Bonaparte's Gull	H	H	L	**L**
Larus pipixcan	Franklin's Gull	L	H	H	**L**
Larus relictus	Relict Gull	H	H	H	**H**
Larus ridibundus	Black-headed Gull	H	H	L	**L**
Larus saundersi	Saunders's Gull	H	H	H	**H**
Larus schistisagus	Slaty-backed Gull	H	H	L	**L**
Larus scopulinus	Red-billed Gull	L	H	H	**L**
Larus serranus	Andean Gull	H	H	H	**H**
Larus thayeri	Thayer's Gull	H	H	H	**H**
Laterallus albigularis	White-throated Crake	H	L	L	**L**
Laterallus exilis	Grey-breasted Crake	U	L	H	**L**
Laterallus jamaicensis	Black Rail	L	L	H	**L**
Laterallus leucopyrrhus	Red-and-white Crake	H	L	H	**L**

Laterallus levraudi	Rusty-flanked Crake	H	U	H	**L**
Laterallus melanophaius	Rufous-sided Crake	H	L	L	**L**
Laterallus ruber	Ruddy Crake	H	L	H	**L**
Laterallus spilonotus	Galapagos Rail	H	H	L	**L**
Laterallus tuerosi	Junin Rail	H	H	H	**H**
Laterallus xenopterus	Rufous-faced Crake	H	U	L	**L**
Lathamus discolor	Swift Parrot	H	L	L	**L**
Lathrotriccus euleri	Euler's Flycatcher	U	L	L	**L**
Lathrotriccus griseipectus	Grey-breasted Flycatcher	H	H	H	**H**
Latoucheornis siemsseni	Slaty Bunting	H	U	H	**L**
Legatus leucophaius	Piratic Flycatcher	L	L	H	**L**
Leiothrix argentauris	Silver-eared Mesia	U	L	H	**L**
Leiothrix lutea	Red-billed Leiothrix	U	L	H	**L**
Leipoa ocellata	Malleefowl	H	H	L	**L**
Leonardina woodi	Bagobo Babbler	H	H	H	**H**
Lepidocolaptes affinis	Spot-crowned Woodcreeper	H	L	H	**L**
Lepidocolaptes albolineatus	Lineated Woodcreeper	H	U	H	**L**
Lepidocolaptes angustirostris	Narrow-billed Woodcreeper	H	L	L	**L**
Lepidocolaptes falcinellus	Scalloped Woodcreeper	H	U	L	**L**
Lepidocolaptes lacrymiger	Montane Woodcreeper	H	U	L	**L**
Lepidocolaptes leucogaster	White-striped Woodcreeper	H	H	L	**L**
Lepidocolaptes souleyetii	Streak-headed Woodcreeper	H	H	H	**H**
Lepidocolaptes squamatus	Scaled Woodcreeper	U	U	L	**L**
Lepidopyga coeruleogularis	Sapphire-throated Hummingbird	H	H	L	**L**
Lepidopyga goudoti	Shining-green Hummingbird	U	U	H	**L**
Lepidopyga lilliae	Sapphire-bellied Hummingbird	H	H	H	**H**
Lepidothrix coeruleocapilla	Cerulean-capped Manakin	U	U	H	**L**
Lepidothrix coronata	Blue-crowned Manakin	H	H	L	**L**
Lepidothrix iris	Opal-crowned Manakin	H	H	H	**H**
Lepidothrix isidorei	Blue-rumped Manakin	H	H	H	**H**
Lepidothrix nattereri	Snow-capped Manakin	H	H	H	**H**
Lepidothrix serena	White-fronted Manakin	H	H	H	**H**
Lepidothrix suavissima	Orange-bellied Manakin	H	H	H	**H**
Lepidothrix vilasboasi	Golden-crowned Manakin	H	H	H	**H**
Leptasthenura aegithaloides	Plain-mantled Tit-spinetail	H	L	H	**L**
Leptasthenura andicola	Andean Tit-spinetail	U	H	H	**L**
Leptasthenura fuliginiceps	Brown-capped Tit-spinetail	H	L	H	**L**
Leptasthenura pileata	Rusty-crowned Tit-spinetail	H	U	H	**L**

Leptasthenura platensis	Tufted Tit-spinetail	H	L	L	**L**
Leptasthenura setaria	Araucaria Tit-spinetail	H	U	L	**L**
Leptasthenura striata	Streaked Tit-spinetail	H	U	H	**L**
Leptasthenura striolata	Striolated Tit-spinetail	H	U	L	**L**
Leptasthenura xenothorax	White-browed Tit-spinetail	H	U	H	**L**
Leptasthenura yanacensis	Tawny Tit-spinetail	H	U	H	**L**
Leptodon cayanensis	Grey-headed Kite	H	H	H	**H**
Leptodon forbesi	White-collared Kite	H	H	H	**H**
Leptopoecile elegans	Crested Tit-warbler	H	U	L	**L**
Leptopoecile sophiae	White-browed Tit-warbler	H	U	H	**L**
Leptopogon amaurocephalus	Sepia-capped Flycatcher	L	L	L	**L**
Leptopogon rufipectus	Rufous-breasted Flycatcher	H	H	H	**H**
Leptopogon superciliaris	Slaty-capped Flycatcher	H	H	L	**L**
Leptopogon taczanowskii	Inca Flycatcher	H	H	H	**H**
Leptopterus chabert	Chabert's Vanga	U	L	U	**L**
Leptoptilos crumeniferus	Marabou Stork	L	H	L	**L**
Leptoptilos dubius	Greater Adjutant	H	H	H	**H**
Leptoptilos javanicus	Lesser Adjutant	H	H	L	**L**
Leptosittaca branickii	Golden-plumed Parakeet	H	H	L	**L**
Leptosomus discolor	Cuckoo-roller	H	L	L	**L**
Leptotila battyi	Brown-backed Dove	H	U	H	**L**
Leptotila cassini	Grey-chested Dove	L	H	H	**L**
Leptotila conoveri	Tolima Dove	H	H	H	**H**
Leptotila jamaicensis	Caribbean Dove	L	H	H	**L**
Leptotila megalura	White-faced Dove	U	H	H	**L**
Leptotila ochraceiventris	Ochre-bellied Dove	H	U	H	**L**
Leptotila pallida	Pallid Dove	H	U	H	**L**
Leptotila plumbeiceps	Grey-headed Dove	U	U	H	**L**
Leptotila rufaxilla	Grey-fronted Dove	L	H	H	**L**
Leptotila verreauxi	White-tipped Dove	L	H	L	**L**
Leptotila wellsi	Grenada Dove	H	H	H	**H**
Lerwa lerwa	Snow Partridge	U	L	U	**L**
Lesbia nuna	Green-tailed Trainbearer	U	H	H	**L**
Lesbia victoriae	Black-tailed Trainbearer	H	U	H	**L**
Lessonia oreas	Andean Negrito	H	L	H	**L**
Lessonia rufa	Patagonian Negrito	U	L	L	**L**
Leucippus baeri	Tumbes Hummingbird	H	U	H	**L**
Leucippus chlorocercus	Olive-spotted Hummingbird	H	H	H	**H**
Leucippus fallax	Buffy Hummingbird	U	H	H	**L**
Leucippus taczanowskii	Spot-throated Hummingbird	H	U	H	**L**
Leucochloris albicollis	White-throated Hummingbird	H	H	L	**L**
Leucopeza semperi	Semper's Warbler	H	H	H	**H**
Leucophaeus scoresbii	Dolphin Gull	H	H	H	**H**

Leucopsar rothschildi	Bali Starling	H	H	H	**H**
Leucopternis albicollis	White Hawk	H	H	H	**H**
Leucopternis kuhli	White-browed Hawk	H	H	H	**H**
Leucopternis lacernulatus	White-necked Hawk	H	H	H	**H**
Leucopternis melanops	Black-faced Hawk	H	H	H	**H**
Leucopternis occidentalis	Grey-backed Hawk	H	H	H	**H**
Leucopternis plumbeus	Plumbeous Hawk	H	H	L	**L**
Leucopternis polionotus	Mantled Hawk	H	H	L	**L**
Leucopternis princeps	Barred Hawk	H	H	L	**L**
Leucopternis schistaceus	Slate-coloured Hawk	H	H	H	**H**
Leucopternis semiplumbeus	Semiplumbeous Hawk	H	H	H	**H**
Leucosarcia melanoleuca	Wonga Pigeon	H	H	L	**L**
Leucosticte arctoa	Asian Rosy-finch	H	U	H	**L**
Leucosticte atrata	Black Rosy-finch	H	L	H	**L**
Leucosticte australis	Brown-capped Rosy-finch	H	L	L	**L**
Leucosticte brandti	Black-headed Mountain-finch	U	U	U	**L**
Leucosticte nemoricola	Plain Mountain-finch	U	L	U	**L**
Leucosticte sillemi	Sillem's Mountain-finch	H	U	H	**L**
Leucosticte tephrocotis	Gray-crowned Rosy-finch	H	L	H	**L**
Lewinia mirifica	Brown-banded Rail	U	U	H	**L**
Lewinia muelleri	Auckland Islands Rail	H	H	H	**H**
Lewinia pectoralis	Lewin's Rail	U	L	U	**L**
Lichenostomus chrysops	Yellow-faced Honeyeater	H	H	L	**L**
Lichenostomus cratitius	Purple-gaped Honeyeater	U	H	U	**L**
Lichenostomus fasciogularis	Mangrove Honeyeater	U	H	H	**L**
Lichenostomus flavescens	Yellow-tinted Honeyeater	U	H	L	**L**
Lichenostomus flavicollis	Yellow-throated Honeyeater	H	L	H	**L**
Lichenostomus flavus	Yellow Honeyeater	U	H	L	**L**
Lichenostomus frenatus	Bridled Honeyeater	H	H	L	**L**
Lichenostomus fuscus	Fuscous Honeyeater	H	H	L	**L**
Lichenostomus hindwoodi	Eungella Honeyeater	U	H	H	**L**
Lichenostomus keartlandi	Grey-headed Honeyeater	U	H	U	**L**
Lichenostomus leucotis	White-eared Honeyeater	H	H	L	**L**
Lichenostomus melanops	Yellow-tufted Honeyeater	H	H	L	**L**
Lichenostomus obscurus	Obscure Honeyeater	U	H	L	**L**
Lichenostomus ornatus	Yellow-plumed Honeyeater	H	H	L	**L**
Lichenostomus penicillatus	White-plumed Honeyeater	H	L	L	**L**
Lichenostomus plumulus	Grey-fronted Honeyeater	U	H	U	**L**
Lichenostomus subfrenatus	Black-throated Honeyeater	U	U	H	**L**
Lichenostomus unicolor	White-gaped Honeyeater	U	H	L	**L**
Lichenostomus versicolor	Varied Honeyeater	H	H	L	**L**

Lichenostomus virescens	Singing Honeyeater	U	H	U	**L**
Lichmera alboauricularis	Silver-eared Honeyeater	U	H	U	**L**
Lichmera argentauris	Olive Honeyeater	H	H	H	**H**
Lichmera deningeri	Buru Honeyeater	H	H	H	**H**
Lichmera flavicans	Yellow-eared Honeyeater	U	H	H	**L**
Lichmera incana	Dark-brown Honeyeater	U	H	H	**L**
Lichmera indistincta	Brown Honeyeater	U	H	L	**L**
Lichmera limbata	Indonesian Honeyeater	H	H	L	**L**
Lichmera lombokia	Scaly-crowned Honeyeater	H	H	H	**H**
Lichmera monticola	Seram Honeyeater	H	H	H	**H**
Lichmera notabilis	Black-chested Honeyeater	H	H	L	**L**
Lichmera squamata	White-tufted Honeyeater	H	H	L	**L**
Limicola falcinellus	Broad-billed Sandpiper	L	H	H	**L**
Limnoctites rectirostris	Straight-billed Reedhaunter	H	L	H	**L**
Limnodromus griseus	Short-billed Dowitcher	L	L	L	**L**
Limnodromus scolopaceus	Long-billed Dowitcher	U	H	U	**L**
Limnodromus semipalmatus	Asian Dowitcher	H	H	H	**H**
Limnornis curvirostris	Curve-billed Reedhaunter	H	H	H	**H**
Limnothlypis swainsonii	Swainson's Warbler	H	L	H	**L**
Limosa fedoa	Marbled Godwit	L	H	L	**L**
Limosa haemastica	Hudsonian Godwit	L	H	L	**L**
Limosa lapponica	Bar-tailed Godwit	L	H	H	**L**
Limosa limosa	Black-tailed Godwit	L	H	H	**L**
Linurgus olivaceus	Oriole Finch	H	U	L	**L**
Liocichla bugunorum	Bugun Liocichla	H	H	L	**L**
Liocichla omeiensis	Omei Shan Liocichla	H	L	H	**L**
Liocichla phoenicea	Red-faced Liocichla	H	H	U	**L**
Liocichla steerii	Taiwan Liocichla	U	L	H	**L**
Lioptilus nigricapillus	Bush Blackcap	H	H	L	**L**
Liosceles thoracicus	Rusty-belted Tapaculo	H	H	L	**L**
Lipaugus fuscocinereus	Dusky Piha	H	U	H	**L**
Lipaugus lanioides	Cinnamon-vented Piha	H	H	L	**L**
Lipaugus streptophorus	Rose-collared Piha	H	U	H	**L**
Lipaugus unirufus	Rufous Piha	L	H	H	**L**
Lipaugus uropygialis	Scimitar-winged Piha	H	U	H	**L**
Lipaugus vociferans	Screaming Piha	H	U	H	**L**
Lipaugus weberi	Chestnut-capped Piha	H	U	L	**L**
Loboparadisea sericea	Yellow-breasted Bird-of-paradise	H	H	H	**H**
Lochmias nematura	Sharp-tailed Streamcreeper	H	H	L	**L**
Locustella certhiola	Pallas's Grasshopper-warbler	U	U	U	**L**
Locustella fasciolata	Gray's Grasshopper-warbler	U	L	U	**L**
Locustella fluviatilis	Eurasian River Warbler	H	L	H	**L**
Locustella lanceolata	Lanceolated Warbler	U	L	H	**L**

Locustella luscinioides	Savi's Warbler	H	L	H	**L**
Locustella naevia	Common Grasshopper-warbler	H	L	H	**L**
Locustella ochotensis	Middendorff's Grasshopper-warbler	U	L	U	**L**
Locustella pleskei	Pleske's Grasshopper-warbler	H	L	H	**L**
Locustella pryeri	Marsh Grassbird	H	L	H	**L**
Loddigesia mirabilis	Marvellous Spatuletail	H	H	H	**H**
Lonchura atricapilla	Chestnut Munia	U	U	L	**L**
Lonchura bicolor	Black-and-white Munia	H	L	L	**L**
Lonchura caniceps	Grey-headed Munia	U	U	U	**L**
Lonchura cantans	African Silverbill	H	L	H	**L**
Lonchura castaneothorax	Chestnut-breasted Munia	U	L	U	**L**
Lonchura cucullata	Bronze Munia	U	L	L	**L**
Lonchura ferruginosa	White-capped Munia	U	U	U	**L**
Lonchura flaviprymna	Yellow-rumped Munia	L	L	H	**L**
Lonchura forbesi	New Ireland Munia	H	U	H	**L**
Lonchura fringilloides	Magpie Munia	U	L	L	**L**
Lonchura fuscans	Dusky Munia	U	L	U	**L**
Lonchura grandis	Grand Munia	U	L	U	**L**
Lonchura griseicapilla	Grey-headed Silverbill	U	L	U	**L**
Lonchura hunsteini	Mottled Munia	H	U	H	**L**
Lonchura kelaarti	Black-throated Munia	U	L	U	**L**
Lonchura leucogastra	White-bellied Munia	U	L	U	**L**
Lonchura leucogastroides	Javan Munia	U	L	U	**L**
Lonchura leucosticta	White-spotted Munia	U	U	U	**L**
Lonchura maja	White-headed Munia	H	L	L	**L**
Lonchura malabarica	White-throated Munia	U	L	U	**L**
Lonchura malacca	Tricoloured Munia	U	L	L	**L**
Lonchura melaena	Bismarck Munia	H	L	H	**L**
Lonchura molucca	Black-faced Munia	U	L	U	**L**
Lonchura montana	Snow Mountain Munia	U	U	H	**L**
Lonchura monticola	Alpine Munia	H	H	H	**H**
Lonchura nana	Madagascar Munia	U	L	U	**L**
Lonchura nevermanni	Grey-crowned Munia	H	L	L	**L**
Lonchura nigerrima	New Hanover Munia	H	U	H	**L**
Lonchura nigriceps	Brown-backed Munia	U	U	L	**L**
Lonchura pallida	Pale-headed Munia	U	L	U	**L**
Lonchura punctulata	Scaly-breasted Munia	U	L	L	**L**
Lonchura quinticolor	Five-coloured Munia	U	U	U	**L**
Lonchura spectabilis	Hooded Munia	U	L	U	**L**
Lonchura striata	White-rumped Munia	U	L	U	**L**
Lonchura stygia	Black Munia	H	L	L	**L**
Lonchura teerinki	Black-breasted Munia	U	U	H	**L**

Lonchura tristissima	Streak-headed Munia	U	U	U	**L**
Lonchura vana	Grey-banded Munia	H	H	H	**H**
Lophaetus occipitalis	Long-crested Eagle	L	H	L	**L**
Lophodytes cucullatus	Hooded Merganser	H	H	L	**L**
Lophoictinia isura	Square-tailed Kite	H	H	L	**L**
Lopholaimus antarcticus	Topknot Pigeon	H	H	L	**L**
Lophonetta specularioides	Crested Duck	H	H	L	**L**
Lophophorus impejanus	Himalayan Monal	H	L	H	**L**
Lophophorus lhuysii	Chinese Monal	H	L	L	**L**
Lophophorus sclateri	Sclater's Monal	H	L	H	**L**
Lophorina superba	Superb Bird-of-paradise	U	H	H	**L**
Lophornis adorabilis	White-crested Coquette	U	H	H	**L**
Lophornis brachylophus	Short-crested Coquette	H	H	L	**L**
Lophornis chalybeus	Festive Coquette	U	H	H	**L**
Lophornis delattrei	Rufous-crested Coquette	U	U	H	**L**
Lophornis gouldii	Dot-eared Coquette	H	H	H	**H**
Lophornis helenae	Black-crested Coquette	L	U	H	**L**
Lophornis magnificus	Frilled Coquette	U	H	L	**L**
Lophornis ornatus	Tufted Coquette	H	H	H	**H**
Lophornis pavoninus	Peacock Coquette	H	H	H	**H**
Lophornis stictolophus	Spangled Coquette	U	U	H	**L**
Lophospingus griseocristatus	Grey-crested Finch	U	U	H	**L**
Lophospingus pusillus	Black-crested Finch	H	U	L	**L**
Lophostrix cristata	Crested Owl	H	U	H	**L**
Lophotibis cristata	Madagascar Crested Ibis	H	H	L	**L**
Lophotriccus eulophotes	Long-crested Pygmy-tyrant	H	U	L	**L**
Lophotriccus galeatus	Helmeted Pygmy-tyrant	H	L	H	**L**
Lophotriccus pileatus	Scale-crested Pygmy-tyrant	H	H	L	**L**
Lophotriccus vitiosus	Double-banded Pygmy-tyrant	H	H	H	**H**
Lophozosterops dohertyi	Crested White-eye	H	H	H	**H**
Lophozosterops goodfellowi	Black-masked White-eye	H	H	H	**H**
Lophozosterops javanicus	Javan Grey-throated White-eye	H	H	H	**H**
Lophozosterops pinaiae	Grey-hooded White-eye	H	H	H	**H**
Lophozosterops squamiceps	Streaky-headed White-eye	H	H	H	**H**
Lophozosterops superciliaris	Yellow-browed White-eye	H	H	H	**H**
Lophura bulweri	Wattled Pheasant	H	H	H	**H**
Lophura diardi	Siamese Fireback	H	L	H	**L**
Lophura edwardsi	Edwards's Pheasant	H	L	H	**L**
Lophura erythrophthalma	Crestless Fireback	H	L	H	**L**
Lophura hatinhensis	Vietnamese Pheasant	H	L	H	**L**

Lophura hoogerwerfi	Aceh Pheasant	H	H	H	**H**
Lophura ignita	Crested Fireback	H	L	H	**L**
Lophura inornata	Salvadori's Pheasant	H	H	H	**H**
Lophura leucomelanos	Kalij Pheasant	U	L	U	**L**
Lophura nycthemera	Silver Pheasant	U	L	H	**L**
Lophura swinhoii	Swinhoe's Pheasant	H	L	H	**L**
Loriculus amabilis	Moluccan Hanging-parrot	U	U	U	**L**
Loriculus aurantiifrons	Orange-fronted Hanging-parrot	H	L	U	**L**
Loriculus beryllinus	Sri Lanka Hanging-parrot	U	L	H	**L**
Loriculus catamene	Sangihe Hanging-parrot	H	H	H	**H**
Loriculus exilis	Red-billed Hanging-parrot	U	U	H	**L**
Loriculus flosculus	Flores Hanging-parrot	H	H	H	**H**
Loriculus galgulus	Blue-crowned Hanging-parrot	H	L	H	**L**
Loriculus philippensis	Philippine Hanging-parrot	U	L	U	**L**
Loriculus pusillus	Yellow-throated Hanging-parrot	H	H	H	**H**
Loriculus sclateri	Sula Hanging-parrot	H	U	L	**L**
Loriculus stigmatus	Sulawesi Hanging-parrot	U	U	U	**L**
Loriculus tener	Green-fronted Hanging-parrot	H	U	H	**L**
Loriculus vernalis	Vernal Hanging-parrot	U	L	H	**L**
Lorius albidinucha	White-naped Lory	H	H	H	**H**
Lorius chlorocercus	Yellow-bibbed Lory	H	H	H	**H**
Lorius domicella	Purple-naped Lory	H	H	H	**H**
Lorius garrulus	Chattering Lory	H	H	H	**H**
Lorius hypoinochrous	Purple-bellied Lory	U	H	U	**L**
Lorius lory	Black-capped Lory	H	H	L	**L**
Loxia curvirostra	Red Crossbill	H	L	L	**L**
Loxia leucoptera	White-winged Crossbill	H	L	H	**L**
Loxia megaplaga	Hispaniolan Crossbill	H	H	H	**H**
Loxia pytyopsittacus	Parrot Crossbill	H	L	H	**L**
Loxia scotica	Scottish Crossbill	H	L	H	**L**
Loxigilla barbadensis	Barbados Bullfinch	H	H	H	**H**
Loxigilla noctis	Lesser Antillean Bullfinch	H	L	H	**L**
Loxigilla portoricensis	Puerto Rican Bullfinch	H	L	H	**L**
Loxigilla violacea	Greater Antillean Bullfinch	U	L	L	**L**
Loxioides bailleui	Palila	H	H	U	**L**
Loxipasser anoxanthus	Yellow-shouldered Grassquit	H	L	H	**L**
Loxops caeruleirostris	Akekee	H	H	H	**H**
Loxops coccineus	Akepa	H	H	H	**H**
Lugensa brevirostris	Kerguelen Petrel	H	H	L	**L**
Lullula arborea	Wood Lark	H	L	H	**L**
Lurocalis rufiventris	Rufous-bellied Nighthawk	H	U	H	**L**

Lurocalis semitorquatus	Short-tailed Nighthawk	H	H	H	**H**
Luscinia brunnea	Indian Blue Robin	U	L	U	**L**
Luscinia calliope	Siberian Rubythroat	U	L	H	**L**
Luscinia cyane	Siberian Blue Robin	U	U	U	**L**
Luscinia luscinia	Thrush Nightingale	H	L	H	**L**
Luscinia megarhynchos	Common Nightingale	L	L	L	**L**
Luscinia obscura	Black-throated Blue Robin	H	L	H	**L**
Luscinia pectardens	Firethroat	H	U	H	**L**
Luscinia pectoralis	White-tailed Rubythroat	U	L	U	**L**
Luscinia ruficeps	Rufous-headed Robin	H	H	H	**H**
Luscinia sibilans	Rufous-tailed Robin	U	U	U	**L**
Luscinia svecica	Bluethroat	H	L	H	**L**
Lybius bidentatus	Double-toothed Barbet	H	H	H	**H**
Lybius chaplini	Zambian Barbet	H	H	L	**L**
Lybius dubius	Bearded Barbet	H	H	H	**H**
Lybius guifsobalito	Black-billed Barbet	U	H	L	**L**
Lybius leucocephalus	White-headed Barbet	H	H	U	**L**
Lybius melanopterus	Brown-breasted Barbet	U	H	L	**L**
Lybius minor	Black-backed Barbet	U	H	L	**L**
Lybius rolleti	Black-breasted Barbet	U	H	U	**L**
Lybius rubrifacies	Red-faced Barbet	H	H	L	**L**
Lybius torquatus	Black-collared Barbet	H	H	L	**L**
Lybius undatus	Banded Barbet	U	H	L	**L**
Lybius vieilloti	Vieillot's Barbet	H	H	H	**H**
Lycocorax pyrrhopterus	Paradise-crow	H	H	H	**H**
Lymnocryptes minimus	Jack Snipe	H	H	H	**H**
Macgregoria pulchra	Ochre-winged Honeyeater	H	H	H	**H**
Machaerirhynchus flaviventer	Yellow-breasted Boatbill	U	H	L	**L**
Machaerirhynchus nigripectus	Black-breasted Boatbill	U	H	H	**L**
Machaeropterus deliciosus	Club-winged Manakin	H	H	H	**H**
Machaeropterus pyrocephalus	Fiery-capped Manakin	H	H	L	**L**
Machaeropterus regulus	Striped Manakin	H	H	H	**H**
Macheiramphus alcinus	Bat Hawk	H	H	L	**L**
Machetornis rixosa	Cattle Tyrant	U	L	L	**L**
Mackenziaena leachii	Large-tailed Antshrike	H	U	L	**L**
Mackenziaena severa	Tufted Antshrike	H	U	L	**L**
Macroagelaius imthurni	Golden-tufted Grackle	H	U	H	**L**
Macroagelaius subalaris	Mountain Grackle	H	H	H	**H**
Macrocephalon maleo	Maleo	H	H	H	**H**
Macrodipteryx longipennis	Standard-winged Nightjar	U	H	H	**L**
Macrodipteryx vexillarius	Pennant-winged Nightjar	U	H	L	**L**

Macronectes giganteus	Southern Giant-petrel	H	H	L	**L**
Macronectes halli	Northern Giant-petrel	H	H	L	**L**
Macronous bornensis	Bold-striped Tit-babbler	U	L	U	**L**
Macronous flavicollis	Grey-cheeked Tit-babbler	H	H	H	**H**
Macronous gularis	Pin-striped Tit-babbler	U	L	U	**L**
Macronous kelleyi	Grey-faced Tit-babbler	H	H	L	**L**
Macronous ptilosus	Fluffy-backed Tit-babbler	H	L	H	**L**
Macronous striaticeps	Brown Tit-babbler	H	H	U	**L**
Macronyx ameliae	Rosy-throated Longclaw	U	L	U	**L**
Macronyx aurantiigula	Pangani Longclaw	U	L	U	**L**
Macronyx capensis	Cape Longclaw	H	L	L	**L**
Macronyx croceus	Yellow-throated Longclaw	U	L	U	**L**
Macronyx flavicollis	Abyssinian Longclaw	H	L	L	**L**
Macronyx fuellebornii	Fuelleborn's Longclaw	U	L	U	**L**
Macronyx grimwoodi	Grimwood's Longclaw	H	U	H	**L**
Macronyx sharpei	Sharpe's Longclaw	H	L	H	**L**
Macropsalis forcipata	Long-trained Nightjar	H	U	H	**L**
Macropygia amboinensis	Brown Cuckoo-dove	U	H	U	**L**
Macropygia emiliana	Ruddy Cuckoo-dove	U	H	L	**L**
Macropygia mackinlayi	Mackinlay's Cuckoo-dove	U	H	U	**L**
Macropygia magna	Dusky Cuckoo-dove	H	U	L	**L**
Macropygia nigrirostris	Black-billed Cuckoo-dove	H	H	H	**H**
Macropygia ruficeps	Little Cuckoo-dove	U	H	L	**L**
Macropygia rufipennis	Andaman Cuckoo-dove	H	U	L	**L**
Macropygia tenuirostris	Philippine Cuckoo-dove	H	H	L	**L**
Macropygia unchall	Barred Cuckoo-dove	U	H	H	**L**
Macrosphenus concolor	Grey Longbill	H	U	H	**L**
Macrosphenus flavicans	Yellow Longbill	H	H	H	**H**
Macrosphenus kempi	Kemp's Longbill	H	U	L	**L**
Macrosphenus kretschmeri	Kretschmer's Longbill	H	H	H	**H**
Macrosphenus pulitzeri	Pulitzer's Longbill	H	H	H	**H**
Madanga ruficollis	Rufous-throated White-eye	H	H	H	**H**
Malacocincla abbotti	Abbott's Babbler	U	L	U	**L**
Malacocincla cinereiceps	Ashy-headed Babbler	H	H	H	**H**
Malacocincla malaccensis	Short-tailed Babbler	H	L	H	**L**
Malacocincla perspicillata	Black-browed Babbler	H	H	H	**H**
Malacocincla sepiaria	Horsfield's Babbler	H	H	U	**L**
Malaconotus alius	Uluguru Bush-shrike	H	H	H	**H**
Malaconotus blanchoti	Grey-headed Bush-shrike	U	L	L	**L**
Malaconotus bocagei	Grey-green Bush-shrike	U	H	U	**L**
Malaconotus cruentus	Fiery-breasted Bush-shrike	H	L	L	**L**
Malaconotus dohertyi	Doherty's Bush-shrike	H	L	H	**L**
Malaconotus gladiator	Green-breasted Bush-shrike	H	U	H	**L**
Malaconotus kupeensis	Mount Kupe Bush-shrike	H	H	H	**H**

Malaconotus lagdeni	Lagden's Bush-shrike	U	H	H	**L**
Malaconotus monteiri	Monteiro's Bush-shrike	U	U	H	**L**
Malaconotus multicolor	Many-coloured Bush-shrike	U	U	U	**L**
Malaconotus olivaceus	Olive Bush-shrike	H	H	L	**L**
Malaconotus sulfureopectus	Sulphur-breasted Bush-shrike	U	H	U	**L**
Malaconotus zeylonus	Bokmakierie Bush-shrike	H	L	L	**L**
Malacopteron affine	Sooty-capped Babbler	H	H	H	**H**
Malacopteron albogulare	Grey-breasted Babbler	H	H	H	**H**
Malacopteron cinereum	Scaly-crowned Babbler	H	H	U	**L**
Malacopteron magnirostre	Moustached Babbler	U	H	U	**L**
Malacopteron magnum	Rufous-crowned Babbler	H	H	H	**H**
Malacopteron palawanense	Melodious Babbler	H	H	H	**H**
Malacoptila fulvogularis	Black-streaked Puffbird	H	U	H	**L**
Malacoptila fusca	White-chested Puffbird	H	U	H	**L**
Malacoptila mystacalis	Moustached Puffbird	H	U	H	**L**
Malacoptila panamensis	White-whiskered Puffbird	H	L	H	**L**
Malacoptila rufa	Rufous-necked Puffbird	H	U	H	**L**
Malacoptila semicincta	Semicollared Puffbird	H	U	L	**L**
Malacoptila striata	Crescent-chested Puffbird	H	U	L	**L**
Malacorhynchus membranaceus	Pink-eared Duck	H	H	L	**L**
Malcorus pectoralis	Rufous-eared Warbler	H	H	H	**H**
Malia grata	Malia	H	H	H	**H**
Malimbus ballmanni	Gola Malimbe	H	U	L	**L**
Malimbus cassini	Black-throated Malimbe	H	U	H	**L**
Malimbus coronatus	Red-crowned Malimbe	H	H	L	**L**
Malimbus erythrogaster	Red-bellied Malimbe	H	H	H	**H**
Malimbus ibadanensis	Ibadan Malimbe	H	U	H	**L**
Malimbus malimbicus	Crested Malimbe	H	U	L	**L**
Malimbus nitens	Gray's Malimbe	H	U	L	**L**
Malimbus racheliae	Rachel's Malimbe	H	H	H	**H**
Malimbus rubricollis	Red-headed Malimbe	H	U	L	**L**
Malimbus scutatus	Red-vented Malimbe	H	U	H	**L**
Malurus alboscapulatus	White-shouldered Fairywren	U	H	U	**L**
Malurus amabilis	Lovely Fairywren	U	H	L	**L**
Malurus coronatus	Purple-crowned Fairywren	H	H	U	**L**
Malurus cyaneus	Superb Fairywren	H	H	L	**L**
Malurus cyanocephalus	Emperor Fairywren	H	H	L	**L**
Malurus elegans	Red-winged Fairywren	H	H	H	**H**
Malurus grayi	Broad-billed Fairywren	H	H	U	**L**
Malurus lamberti	Variegated Fairywren	H	H	L	**L**
Malurus leucopterus	White-winged Fairywren	U	H	U	**L**

Malurus melanocephalus	Red-backed Fairywren	U	H	L	**L**
Malurus pulcherrimus	Blue-breasted Fairywren	U	H	U	**L**
Malurus splendens	Splendid Fairywren	H	H	L	**L**
Manacus aurantiacus	Orange-collared Manakin	U	H	H	**L**
Manacus candei	White-collared Manakin	L	H	H	**L**
Manacus manacus	White-bearded Manakin	U	H	H	**L**
Manacus vitellinus	Golden-collared Manakin	H	H	L	**L**
Mandingoa nitidula	Green-backed Twinspot	U	L	L	**L**
Manorina flavigula	Yellow-throated Miner	H	H	L	**L**
Manorina melanocephala	Noisy Miner	H	H	L	**L**
Manorina melanophrys	Bell Miner	H	H	L	**L**
Manorina melanotis	Black-eared Miner	H	H	H	**H**
Manucodia ater	Glossy-mantled Manucode	U	H	U	**L**
Manucodia chalybatus	Crinkle-collared Manucode	H	H	U	**L**
Manucodia comrii	Curl-crested Manucode	H	H	L	**L**
Manucodia jobiensis	Jobi Manucode	H	H	U	**L**
Manucodia keraudrenii	Trumpet Manucode	H	H	H	**H**
Margaroperdix madagascariensis	Madagascar Partridge	L	L	L	**L**
Margarops fuscatus	Pearly-eyed Thrasher	U	L	H	**L**
Margarops fuscus	Scaly-breasted Thrasher	H	H	H	**H**
Margarornis bellulus	Beautiful Treerunner	H	H	H	**H**
Margarornis rubiginosus	Ruddy Treerunner	H	H	H	**H**
Margarornis squamiger	Pearled Treerunner	H	H	L	**L**
Margarornis stellatus	Fulvous-dotted Treerunner	H	H	H	**H**
Marmaronetta angustirostris	Marbled Teal	H	H	H	**H**
Masius chrysopterus	Golden-winged Manakin	H	H	H	**H**
Mayrornis lessoni	Slaty Monarch	H	H	L	**L**
Mayrornis schistaceus	Vanikoro Monarch	H	H	H	**H**
Mayrornis versicolor	Ogea Monarch	H	H	U	**L**
Mearnsia novaeguineae	Papuan Needletail	H	H	L	**L**
Mearnsia picina	Philippine Needletail	H	H	H	**H**
Mecocerculus calopterus	Rufous-winged Tyrannulet	U	U	H	**L**
Mecocerculus hellmayri	Buff-banded Tyrannulet	H	H	H	**H**
Mecocerculus leucophrys	White-throated Tyrannulet	U	H	L	**L**
Mecocerculus minor	Sulphur-bellied Tyrannulet	H	H	H	**H**
Mecocerculus poecilocercus	White-tailed Tyrannulet	H	H	L	**L**
Mecocerculus stictopterus	White-banded Tyrannulet	U	U	L	**L**
Megabyas flammulatus	African Shrike-flycatcher	H	U	L	**L**
Megaceryle alcyon	Belted Kingfisher	H	L	L	**L**
Megaceryle lugubris	Crested Kingfisher	U	L	H	**L**
Megaceryle maxima	Giant Kingfisher	U	L	L	**L**
Megaceryle torquata	Ringed Kingfisher	L	L	L	**L**

Megacrex inepta	New Guinea Flightless Rail	H	U	H	**L**
Megadyptes antipodes	Yellow-eyed Penguin	H	H	H	**H**
Megalaima armillaris	Flame-fronted Barbet	U	H	H	**L**
Megalaima asiatica	Blue-throated Barbet	H	H	U	**L**
Megalaima australis	Blue-eared Barbet	H	H	U	**L**
Megalaima chrysopogon	Gold-whiskered Barbet	H	H	U	**L**
Megalaima corvina	Brown-throated Barbet	H	H	H	**H**
Megalaima eximia	Bornean Barbet	H	H	H	**H**
Megalaima faber	Chinese Barbet	U	H	U	**L**
Megalaima faiostricta	Green-eared Barbet	H	H	U	**L**
Megalaima flavifrons	Yellow-fronted Barbet	U	H	H	**L**
Megalaima franklinii	Golden-throated Barbet	H	H	U	**L**
Megalaima haemacephala	Coppersmith Barbet	H	H	L	**L**
Megalaima henricii	Yellow-crowned Barbet	H	H	H	**H**
Megalaima incognita	Moustached Barbet	H	H	U	**L**
Megalaima javensis	Black-banded Barbet	H	H	H	**H**
Megalaima lagrandieri	Red-vented Barbet	H	H	U	**L**
Megalaima lineata	Lineated Barbet	H	H	U	**L**
Megalaima monticola	Mountain Barbet	H	H	H	**H**
Megalaima mystacophanos	Red-throated Barbet	H	H	H	**H**
Megalaima nuchalis	Taiwan Barbet	U	H	U	**L**
Megalaima oorti	Black-browed Barbet	U	H	U	**L**
Megalaima pulcherrima	Golden-naped Barbet	H	H	H	**H**
Megalaima rafflesii	Red-crowned Barbet	H	H	H	**H**
Megalaima rubricapillus	Crimson-fronted Barbet	U	H	U	**L**
Megalaima virens	Great Barbet	H	H	H	**H**
Megalaima viridis	White-cheeked Barbet	H	H	U	**L**
Megalaima zeylanica	Brown-headed Barbet	U	H	U	**L**
Megalurulus grosvenori	Bismarck Thicketbird	H	H	H	**H**
Megalurulus llaneae	Bougainville Thicketbird	H	H	H	**H**
Megalurulus mariei	New Caledonian Grassbird	H	U	H	**L**
Megalurulus rubiginosus	Rusty Thicketbird	H	H	H	**H**
Megalurulus whitneyi	Guadalcanal Thicketbird	H	H	H	**H**
Megalurus albolimbatus	Fly River Grassbird	H	L	L	**L**
Megalurus gramineus	Little Grassbird	H	L	L	**L**
Megalurus palustris	Striated Grassbird	U	L	U	**L**
Megalurus timoriensis	Tawny Grassbird	U	L	U	**L**
Megapodius affinis	New Guinea Megapode	H	U	H	**L**
Megapodius bernsteinii	Sula Megapode	H	H	L	**L**
Megapodius cumingii	Tabon Megapode	H	U	H	**L**
Megapodius eremita	Melanesian Megapode	H	U	L	**L**
Megapodius freycinet	Dusky Megapode	H	U	U	**L**
Megapodius	Biak Megapode	H	U	H	**L**

geelvinkianus					
Megapodius laperouse	Micronesian Megapode	H	H	H	**H**
Megapodius layardi	Vanuatu Megapode	H	U	H	**L**
Megapodius nicobariensis	Nicobar Megapode	H	U	H	**L**
Megapodius pritchardii	Polynesian Megapode	H	H	H	**H**
Megapodius reinwardt	Orange-footed Megapode	U	L	U	**L**
Megapodius tenimberensis	Tanimbar Megapode	H	U	L	**L**
Megarynchus pitangua	Boat-billed Flycatcher	L	L	H	**L**
Megascops albogularis	White-throated Screech-owl	H	H	H	**H**
Megascops asio	Eastern Screech-owl	H	H	L	**L**
Megascops atricapilla	Black-capped Screech-owl	U	H	L	**L**
Megascops barbarus	Bearded Screech-owl	H	H	H	**H**
Megascops choliba	Tropical Screech-owl	U	H	L	**L**
Megascops clarkii	Bare-shanked Screech-owl	H	H	H	**H**
Megascops colombianus	Colombian Screech-owl	H	H	H	**H**
Megascops cooperi	Pacific Screech-owl	L	H	H	**L**
Megascops guatemalae	Vermiculated Screech-owl	H	H	L	**L**
Megascops hoyi	Montane Forest Screech-owl	H	H	H	**H**
Megascops ingens	Rufescent Screech-owl	H	H	U	**L**
Megascops kennicottii	Western Screech-owl	H	H	H	**H**
Megascops koepckeae	Koepcke's Screech-owl	H	H	U	**L**
Megascops marshalli	Cloud-forest Screech-owl	H	H	H	**H**
Megascops nudipes	Puerto Rican Screech-owl	H	H	H	**H**
Megascops petersoni	Cinnamon Screech-owl	H	H	H	**H**
Megascops roboratus	West Peruvian Screech-owl	U	H	H	**L**
Megascops sanctaecatarinae	Long-tufted Screech-owl	H	H	L	**L**
Megascops seductus	Balsas Screech-owl	L	H	H	**L**
Megascops trichopsis	Whiskered Screech-owl	H	H	L	**L**
Megascops watsonii	Tawny-bellied Screech-owl	H	H	H	**H**
Megastictus margaritatus	Pearly Antshrike	H	H	H	**H**
Megatriorchis doriae	Doria's Goshawk	H	H	L	**L**
Megaxenops parnaguae	Great Xenops	U	U	L	**L**
Megazosterops palauensis	Giant White-eye	H	H	H	**H**
Meiglyptes jugularis	Black-and-buff Woodpecker	H	U	U	**L**
Meiglyptes tristis	Buff-rumped Woodpecker	U	H	U	**L**
Meiglyptes tukki	Buff-necked Woodpecker	H	H	H	**H**
Melaenornis annamarulae	Nimba Flycatcher	H	U	L	**L**
Melaenornis ardesiacus	Yellow-eyed Black Flycatcher	U	H	L	**L**
Melaenornis edolioides	Northern Black Flycatcher	U	L	H	**L**
Melaenornis pammelaina	Southern Black Flycatcher	U	L	U	**L**
Melampitta gigantea	Greater Melampitta	H	U	H	**L**

Melampitta lugubris	Lesser Melampitta	U	H	U	**L**
Melamprosops phaeosoma	Poo-uli	H	H	H	**H**
Melanerpes aurifrons	Golden-fronted Woodpecker	L	L	H	**L**
Melanerpes cactorum	White-fronted Woodpecker	H	U	L	**L**
Melanerpes candidus	White Woodpecker	U	L	L	**L**
Melanerpes carolinus	Red-bellied Woodpecker	H	L	L	**L**
Melanerpes chrysauchen	Golden-naped Woodpecker	H	L	H	**L**
Melanerpes chrysogenys	Golden-cheeked Woodpecker	L	U	H	**L**
Melanerpes cruentatus	Yellow-tufted Woodpecker	H	U	H	**L**
Melanerpes erythrocephalus	Red-headed Woodpecker	H	L	L	**L**
Melanerpes flavifrons	Yellow-fronted Woodpecker	H	U	L	**L**
Melanerpes formicivorus	Acorn Woodpecker	H	L	L	**L**
Melanerpes herminieri	Guadeloupe Woodpecker	H	U	H	**L**
Melanerpes hoffmannii	Hoffmann's Woodpecker	H	L	H	**L**
Melanerpes hypopolius	Grey-breasted Woodpecker	L	U	L	**L**
Melanerpes lewis	Lewis's Woodpecker	H	L	H	**L**
Melanerpes portoricensis	Puerto Rican Woodpecker	H	U	H	**L**
Melanerpes pucherani	Black-cheeked Woodpecker	H	L	H	**L**
Melanerpes pulcher	Beautiful Woodpecker	H	U	H	**L**
Melanerpes pygmaeus	Yucatan Woodpecker	U	U	H	**L**
Melanerpes radiolatus	Jamaican Woodpecker	H	L	H	**L**
Melanerpes rubricapillus	Red-crowned Woodpecker	H	L	L	**L**
Melanerpes striatus	Hispaniolan Woodpecker	U	L	H	**L**
Melanerpes superciliaris	West Indian Woodpecker	H	L	L	**L**
Melanerpes uropygialis	Gila Woodpecker	H	L	H	**L**
Melanitta fusca	White-winged Scoter	H	H	L	**L**
Melanitta nigra	Black Scoter	H	H	L	**L**
Melanitta perspicillata	Surf Scoter	L	H	L	**L**
Melanocharis arfakiana	Obscure Berrypecker	H	U	H	**L**
Melanocharis crassirostris	Spotted Berrypecker	U	U	H	**L**
Melanocharis longicauda	Lemon-breasted Berrypecker	U	U	L	**L**
Melanocharis nigra	Black Berrypecker	U	H	L	**L**
Melanocharis striativentris	Streaked Berrypecker	U	U	H	**L**
Melanocharis versteri	Fan-tailed Berrypecker	U	U	H	**L**
Melanochlora sultanea	Sultan Tit	H	L	H	**L**
Melanocorypha bimaculata	Bimaculated Lark	H	L	L	**L**
Melanocorypha calandra	Calandra Lark	H	L	L	**L**
Melanocorypha leucoptera	White-winged Lark	U	L	U	**L**
Melanocorypha maxima	Tibetan Lark	U	L	U	**L**
Melanocorypha	Mongolian Lark	U	L	U	**L**

mongolica					
Melanocorypha yeltoniensis	Black Lark	H	L	L	**L**
Melanodera melanodera	Canary-winged Finch	H	H	H	**H**
Melanodera xanthogramma	Yellow-bridled Finch	U	U	H	**L**
Melanodryas cucullata	Hooded Robin	U	H	U	**L**
Melanodryas vittata	Dusky Robin	H	L	H	**L**
Melanopareia elegans	Elegant Crescentchest	U	U	H	**L**
Melanopareia maranonica	Maranon Crescentchest	H	U	H	**L**
Melanopareia maximiliani	Olive-crowned Crescentchest	U	U	H	**L**
Melanopareia torquata	Collared Crescentchest	U	U	L	**L**
Melanoperdix niger	Black Partridge	H	L	H	**L**
Melanoptila glabrirostris	Black Catbird	L	H	H	**L**
Melanospiza richardsoni	St Lucia Black Finch	H	H	H	**H**
Melanotis caerulescens	Blue Mockingbird	L	H	H	**L**
Melanotis hypoleucus	Blue-and-white Mockingbird	L	L	H	**L**
Meleagris gallopavo	Wild Turkey	H	H	H	**H**
Meleagris ocellata	Ocellated Turkey	L	H	H	**L**
Melichneutes robustus	Lyre-tailed Honeyguide	H	U	H	**L**
Melidectes belfordi	Belford's Melidectes	U	U	H	**L**
Melidectes foersteri	Huon Melidectes	H	U	H	**L**
Melidectes fuscus	Sooty Melidectes	H	H	H	**H**
Melidectes leucostephes	Vogelkop Melidectes	H	U	H	**L**
Melidectes nouhuysi	Short-bearded Melidectes	U	H	H	**L**
Melidectes ochromelas	Cinnamon-browed Melidectes	H	U	H	**L**
Melidectes princeps	Long-bearded Melidectes	H	H	H	**H**
Melidectes rufocrissalis	Yellow-browed Melidectes	U	U	H	**L**
Melidectes sclateri	San Cristobal Melidectes	H	U	L	**L**
Melidectes torquatus	Ornate Melidectes	U	U	H	**L**
Melidectes whitemanensis	Bismarck Melidectes	H	H	H	**H**
Melidora macrorrhina	Hook-billed Kingfisher	H	L	L	**L**
Melierax canorus	Pale Chanting-goshawk	H	H	H	**H**
Melierax gabar	Gabar Goshawk	L	H	L	**L**
Melierax metabates	Dark Chanting-goshawk	L	H	L	**L**
Melierax poliopterus	Eastern Chanting-goshawk	H	H	L	**L**
Melignomon eisentrauti	Yellow-footed Honeyguide	H	U	H	**L**
Melignomon zenkeri	Zenker's Honeyguide	H	U	H	**L**
Melilestes megarhynchus	Long-billed Honeyeater	U	U	L	**L**
Meliphaga albilineata	White-lined Honeyeater	U	H	L	**L**
Meliphaga albonotata	Scrub Honeyeater	U	H	U	**L**
Meliphaga analoga	Mimic Honeyeater	U	U	L	**L**
Meliphaga aruensis	Puff-backed Honeyeater	U	H	L	**L**

Meliphaga flavirictus	Yellow-gaped Honeyeater	U	U	L	**L**
Meliphaga gracilis	Graceful Honeyeater	U	H	L	**L**
Meliphaga lewinii	Lewin's Honeyeater	H	L	L	**L**
Meliphaga mimikae	Mottle-breasted Honeyeater	H	U	H	**L**
Meliphaga montana	Forest Honeyeater	U	U	H	**L**
Meliphaga notata	Yellow-spotted Honeyeater	U	L	L	**L**
Meliphaga orientalis	Hill-forest Honeyeater	U	U	H	**L**
Meliphaga reticulata	Streaky-breasted Honeyeater	U	U	H	**L**
Meliphaga vicina	Tagula Honeyeater	H	U	H	**L**
Melipotes ater	Spangled Honeyeater	H	U	H	**L**
Melipotes fumigatus	Smoky Honeyeater	U	H	H	**L**
Melipotes gymnops	Arfak Honeyeater	H	U	H	**L**
Melithreptus affinis	Black-headed Honeyeater	U	L	U	**L**
Melithreptus albogularis	White-throated Honeyeater	U	H	L	**L**
Melithreptus brevirostris	Brown-headed Honeyeater	H	L	L	**L**
Melithreptus gularis	Black-chinned Honeyeater	U	H	U	**L**
Melithreptus lunatus	White-naped Honeyeater	H	L	L	**L**
Melithreptus validirostris	Strong-billed Honeyeater	H	L	H	**L**
Melitograis gilolensis	White-streaked Friarbird	H	U	H	**L**
Mellisuga helenae	Bee Hummingbird	U	H	H	**L**
Mellisuga minima	Vervain Hummingbird	U	H	H	**L**
Melocichla mentalis	Moustached Grass-warbler	U	L	L	**L**
Melophus lathami	Crested Bunting	U	L	U	**L**
Melopsittacus undulatus	Budgerigar	H	L	L	**L**
Melopyrrha nigra	Cuban Bullfinch	U	L	H	**L**
Melospiza georgiana	Swamp Sparrow	H	L	L	**L**
Melospiza lincolnii	Lincoln's Sparrow	H	L	L	**L**
Melospiza melodia	Song Sparrow	H	L	L	**L**
Melozone biarcuata	Prevost's Ground-sparrow	L	L	H	**L**
Melozone kieneri	Rusty-crowned Ground-sparrow	L	L	H	**L**
Melozone leucotis	White-eared Ground-sparrow	L	H	H	**L**
Menura alberti	Albert's Lyrebird	H	H	L	**L**
Menura novaehollandiae	Superb Lyrebird	H	H	H	**H**
Merganetta armata	Torrent Duck	H	H	L	**L**
Mergellus albellus	Smew	H	H	H	**H**
Mergus merganser	Common Merganser	H	H	H	**H**
Mergus octosetaceus	Brazilian Merganser	H	H	L	**L**
Mergus serrator	Red-breasted Merganser	H	H	L	**L**
Mergus squamatus	Scaly-sided Merganser	H	H	H	**H**
Meropogon forsteni	Purple-bearded Bee-eater	H	H	H	**H**
Merops albicollis	White-throated Bee-eater	U	H	U	**L**
Merops apiaster	European Bee-eater	H	H	H	**H**

Merops boehmi	Boehm's Bee-eater	U	H	U	**L**
Merops breweri	Black-headed Bee-eater	H	H	H	**H**
Merops bullockoides	White-fronted Bee-eater	U	H	L	**L**
Merops bulocki	Red-throated Bee-eater	U	H	U	**L**
Merops gularis	Black Bee-eater	H	H	L	**L**
Merops hirundineus	Swallow-tailed Bee-eater	U	H	L	**L**
Merops leschenaulti	Chestnut-headed Bee-eater	U	H	L	**L**
Merops malimbicus	Rosy Bee-eater	H	H	H	**H**
Merops muelleri	Blue-headed Bee-eater	H	H	H	**H**
Merops nubicoides	Southern Carmine Bee-eater	U	H	L	**L**
Merops nubicus	Northern Carmine Bee-eater	U	H	H	**L**
Merops oreobates	Cinnamon-chested Bee-eater	U	H	U	**L**
Merops orientalis	Little Green Bee-eater	U	H	U	**L**
Merops ornatus	Rainbow Bee-eater	L	H	L	**L**
Merops persicus	Blue-cheeked Bee-eater	U	H	L	**L**
Merops philippinus	Blue-tailed Bee-eater	U	H	U	**L**
Merops pusillus	Little Bee-eater	U	H	U	**L**
Merops revoilii	Somali Bee-eater	U	H	U	**L**
Merops superciliosus	Madagascar Bee-eater	U	H	U	**L**
Merops variegatus	Blue-breasted Bee-eater	U	H	U	**L**
Merops viridis	Blue-throated Bee-eater	U	H	U	**L**
Merulaxis ater	Slaty Bristlefront	H	H	H	**H**
Merulaxis stresemanni	Stresemann's Bristlefront	H	H	H	**H**
Mesembrinibis cayennensis	Green Ibis	L	H	H	**L**
Mesitornis unicolor	Brown Mesite	H	H	H	**H**
Mesitornis variegatus	White-breasted Mesite	H	H	L	**L**
Mesophoyx intermedia	Intermediate Egret	L	L	L	**L**
Mesopicos elliotii	Elliot's Woodpecker	H	U	H	**L**
Mesopicos goertae	Grey Woodpecker	H	L	H	**L**
Mesopicos griseocephalus	Olive Woodpecker	H	L	L	**L**
Metabolus rugensis	Chuuk Monarch	H	H	U	**L**
Metallura aeneocauda	Scaled Metaltail	H	H	H	**H**
Metallura baroni	Violet-throated Metaltail	H	H	H	**H**
Metallura eupogon	Fire-throated Metaltail	H	H	H	**H**
Metallura iracunda	Perija Metaltail	L	H	H	**L**
Metallura odomae	Neblina Metaltail	H	H	H	**H**
Metallura phoebe	Black Metaltail	H	H	H	**H**
Metallura theresiae	Coppery Metaltail	H	H	H	**H**
Metallura tyrianthina	Tyrian Metaltail	H	H	L	**L**
Metallura williami	Viridian Metaltail	H	H	H	**H**
Metopidius indicus	Bronze-winged Jacana	U	L	U	**L**
Metopothrix aurantiaca	Orange-fronted Plushcrown	H	U	L	**L**
Metriopelia aymara	Golden-spotted Ground-dove	H	H	H	**H**

Metriopelia ceciliae	Bare-faced Ground-dove	H	U	H	**L**
Metriopelia melanoptera	Black-winged Ground-dove	H	H	H	**H**
Metriopelia morenoi	Bare-eyed Ground-dove	H	H	H	**H**
Micrastur buckleyi	Buckley's Forest-falcon	H	H	H	**H**
Micrastur gilvicollis	Lined Forest-falcon	H	H	H	**H**
Micrastur mintoni	Cryptic Forest-falcon	H	H	H	**H**
Micrastur mirandollei	Slaty-backed Forest-falcon	H	H	H	**H**
Micrastur plumbeus	Plumbeous Forest-falcon	H	H	H	**H**
Micrastur ruficollis	Barred Forest-falcon	L	H	H	**L**
Micrastur semitorquatus	Collared Forest-falcon	L	H	L	**L**
Micrathene whitneyi	Elf Owl	H	L	H	**L**
Microbates cinereiventris	Tawny-faced Gnatwren	H	H	H	**H**
Microbates collaris	Collared Gnatwren	H	H	H	**H**
Microcerculus bambla	Wing-banded Wren	H	H	H	**H**
Microcerculus marginatus	Southern Nightingale-wren	H	H	L	**L**
Microcerculus philomela	Northern Nightingale-wren	H	H	H	**H**
Microcerculus ustulatus	Fluting Wren	H	H	H	**H**
Microchera albocoronata	Snowcap	H	U	H	**L**
Microdynamis parva	Dwarf Koel	H	U	L	**L**
Microeca fascinans	Jacky-winter	H	H	L	**L**
Microeca flavigaster	Lemon-bellied Flyrobin	U	H	U	**L**
Microeca flavovirescens	Olive Flyrobin	U	H	U	**L**
Microeca griseoceps	Yellow-legged Flyrobin	U	H	U	**L**
Microeca hemixantha	Golden-bellied Flyrobin	H	U	H	**L**
Microeca papuana	Canary Flyrobin	U	H	U	**L**
Microhierax caerulescens	Collared Falconet	L	H	H	**L**
Microhierax erythrogenys	Philippine Falconet	H	H	L	**L**
Microhierax fringillarius	Black-thighed Falconet	H	H	H	**H**
Microhierax latifrons	White-fronted Falconet	H	H	H	**H**
Microhierax melanoleucos	Pied Falconet	H	H	H	**H**
Microligea palustris	Green-tailed Warbler	U	H	H	**L**
Micromacronus leytensis	Visayan Miniature-babbler	H	H	H	**H**
Micromacronus sordidus	Mindanao Miniature-babbler	H	H	H	**H**
Micromonacha lanceolata	Lanceolated Monklet	U	H	H	**L**
Microparra capensis	Lesser Jacana	L	L	L	**L**
Micropsitta bruijnii	Red-breasted Pygmy-parrot	U	U	H	**L**
Micropsitta finschii	Finsch's Pygmy-parrot	H	U	L	**L**
Micropsitta geelvinkiana	Geelvink Pygmy-parrot	H	U	H	**L**
Micropsitta keiensis	Yellow-capped Pygmy-parrot	H	U	U	**L**
Micropsitta meeki	Meek's Pygmy-parrot	H	U	H	**L**
Micropsitta pusio	Buff-faced Pygmy-parrot	H	L	H	**L**
Micropygia schomburgkii	Ocellated Crake	U	H	L	**L**
Microrhopias quixensis	Dot-winged Antwren	H	H	H	**H**
Microstilbon burmeisteri	Slender-tailed Woodstar	U	U	H	**L**
Miliaria calandra	Corn Bunting	H	L	L	**L**

Milvago chimachima	Yellow-headed Caracara	L	H	L	**L**
Milvago chimango	Chimango Caracara	L	H	L	**L**
Milvus lineatus	Black-eared Kite	U	H	H	**L**
Milvus migrans	Black Kite	L	H	H	**L**
Milvus milvus	Red Kite	H	H	L	**L**
Mimizuku gurneyi	Giant Scops-owl	H	H	H	**H**
Mimus dorsalis	Brown-backed Mockingbird	H	U	H	**L**
Mimus gilvus	Tropical Mockingbird	L	L	H	**L**
Mimus graysoni	Socorro Mockingbird	H	H	L	**L**
Mimus gundlachii	Bahama Mockingbird	H	H	L	**L**
Mimus longicaudatus	Long-tailed Mockingbird	H	U	H	**L**
Mimus macdonaldi	Espanola Mockingbird	H	H	U	**L**
Mimus melanotis	San Cristobal Mockingbird	H	L	H	**L**
Mimus parvulus	Galapagos Mockingbird	H	L	H	**L**
Mimus patagonicus	Patagonian Mockingbird	U	U	U	**L**
Mimus polyglottos	Northern Mockingbird	H	L	H	**L**
Mimus saturninus	Chalk-browed Mockingbird	U	L	L	**L**
Mimus thenca	Chilean Mockingbird	U	L	L	**L**
Mimus trifasciatus	Floreana Mockingbird	H	H	H	**H**
Mimus triurus	White-banded Mockingbird	H	L	L	**L**
Minla cyanouroptera	Blue-winged Minla	U	L	H	**L**
Minla ignotincta	Red-tailed Minla	U	L	U	**L**
Minla strigula	Chestnut-tailed Minla	U	L	H	**L**
Mino anais	Golden Myna	H	H	L	**L**
Mino dumontii	Yellow-faced Myna	H	H	L	**L**
Mionectes macconnelli	MacConnell's Flycatcher	H	L	H	**L**
Mionectes oleagineus	Ochre-bellied Flycatcher	H	L	H	**L**
Mionectes olivaceus	Olive-striped Flycatcher	U	L	L	**L**
Mionectes rufiventris	Grey-hooded Flycatcher	H	H	L	**L**
Mionectes striaticollis	Streak-necked Flycatcher	U	L	L	**L**
Mirafra affinis	Jerdon's Bushlark	U	U	U	**L**
Mirafra africana	Rufous-naped Lark	U	L	U	**L**
Mirafra africanoides	Fawn-coloured Lark	H	L	L	**L**
Mirafra albicauda	White-tailed Lark	U	H	H	**L**
Mirafra alopex	Abyssinian Lark	U	L	U	**L**
Mirafra angolensis	Angola Lark	U	L	H	**L**
Mirafra apiata	Clapper Lark	H	L	H	**L**
Mirafra ashi	Ash's Lark	H	U	L	**L**
Mirafra assamica	Rufous-winged Lark	U	L	U	**L**
Mirafra cantillans	Singing Bushlark	U	L	H	**L**
Mirafra cheniana	Melodious Lark	H	L	H	**L**
Mirafra collaris	Collared Lark	H	L	L	**L**
Mirafra cordofanica	Kordofan Lark	H	U	H	**L**
Mirafra degodiensis	Degodi Lark	H	H	U	**L**
Mirafra erythrocephala	Indochinese Bushlark	U	U	U	**L**

Mirafra erythroptera	Indian Lark	U	L	U	**L**
Mirafra gilletti	Gillett's Lark	U	L	U	**L**
Mirafra hova	Madagascar Lark	U	H	U	**L**
Mirafra hypermetra	Red-winged Lark	H	L	L	**L**
Mirafra javanica	Australasian Lark	U	L	L	**L**
Mirafra microptera	Burmese Bushlark	U	U	U	**L**
Mirafra passerina	Monotonous Lark	H	L	L	**L**
Mirafra poecilosterna	Pink-breasted Lark	H	H	H	**H**
Mirafra pulpa	Friedmann's Lark	H	U	H	**L**
Mirafra rufa	Rusty Lark	H	U	H	**L**
Mirafra rufocinnamomea	Flappet Lark	L	L	L	**L**
Mirafra sabota	Sabota Lark	H	L	H	**L**
Mirafra somalica	Somali Lark	H	L	L	**L**
Mirafra williamsi	Williams's Lark	H	U	H	**L**
Mitrephanes olivaceus	Olive Flycatcher	H	H	H	**H**
Mitrephanes phaeocercus	Tufted Flycatcher	L	H	L	**L**
Mitrospingus cassinii	Dusky-faced Tanager	U	H	U	**L**
Mitrospingus oleagineus	Olive-backed Tanager	U	U	U	**L**
Mitu mitu	Alagoas Curassow	H	H	H	**H**
Mitu salvini	Salvin's Curassow	H	H	H	**H**
Mitu tomentosum	Crestless Curassow	H	H	H	**H**
Mitu tuberosum	Razor-billed Curassow	H	H	L	**L**
Mniotilta varia	Black-and-white Warbler	H	L	L	**L**
Modulatrix orostruthus	Dapple-throat	H	H	L	**L**
Modulatrix stictigula	Spot-throat	H	H	L	**L**
Mohoua albicilla	Whitehead	H	H	H	**H**
Mohoua novaeseelandiae	Pipipi	H	H	H	**H**
Mohoua ochrocephala	Yellowhead	H	H	H	**H**
Molothrus aeneus	Bronzed Cowbird	L	U	H	**L**
Molothrus ater	Brown-headed Cowbird	H	L	H	**L**
Molothrus bonariensis	Shiny Cowbird	L	L	L	**L**
Molothrus oryzivorus	Giant Cowbird	L	U	H	**L**
Molothrus rufoaxillaris	Screaming Cowbird	H	U	L	**L**
Momotus mexicanus	Russet-crowned Motmot	L	L	H	**L**
Momotus momota	Blue-crowned Motmot	L	L	H	**L**
Monachella muelleriana	Torrent Robin	U	H	U	**L**
Monarcha axillaris	Black Monarch	H	H	L	**L**
Monarcha barbatus	Black-and-white Monarch	U	H	U	**L**
Monarcha boanensis	Black-chinned Monarch	H	H	H	**H**
Monarcha brehmii	Biak Monarch	H	U	H	**L**
Monarcha browni	Kolombangara Monarch	H	U	H	**L**
Monarcha castaneiventris	Chestnut-bellied Monarch	H	L	H	**L**
Monarcha castus	Loetoe Monarch	H	U	L	**L**
Monarcha chrysomela	Golden Monarch	U	U	L	**L**
Monarcha cinerascens	Island Monarch	H	H	L	**L**

Monarcha erythrostictus	Bougainville Monarch	H	U	H	**L**
Monarcha everetti	White-tipped Monarch	H	H	H	**H**
Monarcha frater	Black-winged Monarch	U	L	H	**L**
Monarcha godeffroyi	Yap Monarch	H	H	U	**L**
Monarcha guttula	Spot-winged Monarch	U	H	L	**L**
Monarcha infelix	Manus Monarch	U	U	U	**L**
Monarcha julianae	Black-backed Monarch	H	H	H	**H**
Monarcha leucotis	White-eared Monarch	U	L	U	**L**
Monarcha leucurus	White-tailed Monarch	H	H	L	**L**
Monarcha loricatus	Black-tipped Monarch	U	U	H	**L**
Monarcha manadensis	Hooded Monarch	H	H	L	**L**
Monarcha melanopsis	Black-faced Monarch	U	L	L	**L**
Monarcha menckei	White-breasted Monarch	H	U	H	**L**
Monarcha mundus	Black-bibbed Monarch	H	H	H	**H**
Monarcha pileatus	White-naped Monarch	H	U	H	**L**
Monarcha richardsii	White-capped Monarch	H	H	H	**H**
Monarcha rubiensis	Rufous Monarch	H	H	L	**L**
Monarcha sacerdotum	Flores Monarch	H	H	H	**H**
Monarcha takatsukasae	Tinian Monarch	H	H	U	**L**
Monarcha trivirgatus	Spectacled Monarch	U	L	L	**L**
Monarcha verticalis	Black-tailed Monarch	H	H	H	**H**
Monarcha viduus	White-collared Monarch	H	H	H	**H**
Monasa atra	Black Nunbird	H	U	H	**L**
Monasa flavirostris	Yellow-billed Nunbird	H	U	H	**L**
Monasa morphoeus	White-fronted Nunbird	H	L	H	**L**
Monasa nigrifrons	Black-fronted Nunbird	H	L	L	**L**
Monias benschi	Subdesert Mesite	H	H	H	**H**
Monticola angolensis	Miombo Rock-thrush	U	L	L	**L**
Monticola brevipes	Short-toed Rock-thrush	H	L	H	**L**
Monticola cinclorhynchus	Blue-capped Rock-thrush	U	L	U	**L**
Monticola erythronotus	Amber Mountain Rock-thrush	H	H	L	**L**
Monticola explorator	Sentinel Rock-thrush	H	H	L	**L**
Monticola gularis	White-throated Rock-thrush	U	L	U	**L**
Monticola imerinus	Littoral Rock-thrush	H	H	L	**L**
Monticola rufiventris	Chestnut-bellied Rock-thrush	U	L	U	**L**
Monticola rufocinereus	Little Rock-thrush	H	L	L	**L**
Monticola rupestris	Cape Rock-thrush	H	H	L	**L**
Monticola saxatilis	Rufous-tailed Rock-thrush	H	L	L	**L**
Monticola sharpei	Forest Rock-thrush	U	U	U	**L**
Monticola solitarius	Blue Rock-thrush	L	L	H	**L**
Montifringilla adamsi	Black-winged Snowfinch	H	U	U	**L**
Montifringilla blanfordi	Plain-backed Snowfinch	U	U	U	**L**
Montifringilla davidiana	Small Snowfinch	U	U	U	**L**
Montifringilla nivalis	White-winged Snowfinch	H	L	H	**L**

Montifringilla ruficollis	Rufous-necked Snowfinch	U	U	U	**L**
Montifringilla taczanowskii	White-rumped Snowfinch	U	U	U	**L**
Montifringilla theresae	Afghan Snowfinch	H	U	L	**L**
Morococcyx erythropygus	Lesser Ground-cuckoo	L	H	L	**L**
Morphnus guianensis	Crested Eagle	H	H	H	**H**
Morus bassanus	Northern Gannet	H	H	H	**H**
Morus capensis	Cape Gannet	L	H	L	**L**
Morus serrator	Australasian Gannet	H	H	L	**L**
Motacilla aguimp	African Pied Wagtail	U	L	U	**L**
Motacilla alba	White Wagtail	H	L	H	**L**
Motacilla capensis	Cape Wagtail	U	L	U	**L**
Motacilla cinerea	Grey Wagtail	L	L	H	**L**
Motacilla citreola	Citrine Wagtail	H	L	H	**L**
Motacilla clara	Mountain Wagtail	U	H	U	**L**
Motacilla flava	Yellow Wagtail	L	L	H	**L**
Motacilla flaviventris	Madagascar Wagtail	U	H	U	**L**
Motacilla grandis	Japanese Wagtail	U	L	U	**L**
Motacilla madaraspatensis	White-browed Wagtail	U	L	U	**L**
Motacilla samveasnae	Mekong Wagtail	H	U	H	**L**
Mulleripicus fulvus	Ashy Woodpecker	H	L	U	**L**
Mulleripicus funebris	Sooty Woodpecker	H	U	U	**L**
Mulleripicus pulverulentus	Great Slaty Woodpecker	H	L	L	**L**
Muscicapa adusta	African Dusky Flycatcher	U	L	L	**L**
Muscicapa aquatica	Swamp Flycatcher	U	L	U	**L**
Muscicapa boehmi	Boehm's Flycatcher	H	L	U	**L**
Muscicapa caerulescens	Ashy Flycatcher	U	L	L	**L**
Muscicapa cassini	Cassin's Grey Flycatcher	H	H	L	**L**
Muscicapa comitata	Dusky-blue Flycatcher	H	H	H	**H**
Muscicapa dauurica	Asian Brown Flycatcher	U	L	U	**L**
Muscicapa epulata	Little Grey Flycatcher	H	H	H	**H**
Muscicapa ferruginea	Ferruginous Flycatcher	U	L	U	**L**
Muscicapa gambagae	Gambaga Flycatcher	U	H	L	**L**
Muscicapa griseisticta	Grey-streaked Flycatcher	U	L	H	**L**
Muscicapa infuscata	Sooty Flycatcher	H	L	H	**L**
Muscicapa lendu	Chapin's Flycatcher	H	H	L	**L**
Muscicapa muttui	Brown-breasted Flycatcher	H	L	U	**L**
Muscicapa olivascens	Olivaceous Flycatcher	H	H	L	**L**
Muscicapa randi	Ashy-breasted Flycatcher	H	H	H	**H**
Muscicapa ruficauda	Rusty-tailed Flycatcher	U	L	U	**L**
Muscicapa segregata	Sumba Brown Flycatcher	H	U	H	**L**
Muscicapa sethsmithi	Yellow-footed Flycatcher	H	H	H	**H**
Muscicapa sibirica	Dark-sided Flycatcher	U	L	U	**L**
Muscicapa striata	Spotted Flycatcher	H	L	H	**L**

Muscicapa tessmanni	Tessmann's Flycatcher	H	U	L	**L**
Muscicapa ussheri	Ussher's Flycatcher	H	U	L	**L**
Muscicapella hodgsoni	Pygmy Blue-flycatcher	U	U	U	**L**
Muscigralla brevicauda	Short-tailed Field-tyrant	H	L	H	**L**
Muscipipra vetula	Shear-tailed Grey-tyrant	H	L	L	**L**
Muscisaxicola albifrons	White-fronted Ground-tyrant	H	U	H	**L**
Muscisaxicola albilora	White-browed Ground-tyrant	U	L	H	**L**
Muscisaxicola alpinus	Plain-capped Ground-tyrant	H	U	H	**L**
Muscisaxicola capistratus	Cinnamon-bellied Ground-tyrant	H	L	H	**L**
Muscisaxicola cinereus	Cinereous Ground-tyrant	H	H	H	**H**
Muscisaxicola flavinucha	Ochre-naped Ground-tyrant	H	L	H	**L**
Muscisaxicola fluviatilis	Little Ground-tyrant	H	H	H	**H**
Muscisaxicola frontalis	Black-fronted Ground-tyrant	H	U	H	**L**
Muscisaxicola griseus	Taczanowski's Ground-tyrant	H	L	H	**L**
Muscisaxicola juninensis	Puna Ground-tyrant	H	U	H	**L**
Muscisaxicola maclovianus	Dark-faced Ground-tyrant	U	L	L	**L**
Muscisaxicola maculirostris	Spot-billed Ground-tyrant	H	L	H	**L**
Muscisaxicola rufivertex	Rufous-naped Ground-tyrant	H	L	H	**L**
Musophaga rossae	Ross's Turaco	U	H	L	**L**
Musophaga violacea	Violet Turaco	U	H	H	**L**
Myadestes coloratus	Varied Solitaire	H	H	H	**H**
Myadestes elisabeth	Cuban Solitaire	H	H	L	**L**
Myadestes genibarbis	Rufous-throated Solitaire	H	H	H	**H**
Myadestes lanaiensis	Olomao	H	H	H	**H**
Myadestes melanops	Black-faced Solitaire	H	H	H	**H**
Myadestes obscurus	Omao	H	H	H	**H**
Myadestes occidentalis	Brown-backed Solitaire	H	H	L	**L**
Myadestes palmeri	Puaiohi	H	H	H	**H**
Myadestes ralloides	Andean Solitaire	H	H	L	**L**
Myadestes townsendi	Townsend's Solitaire	H	L	H	**L**
Myadestes unicolor	Slate-coloured Solitaire	H	H	H	**H**
Mycerobas affinis	Collared Grosbeak	H	U	H	**L**
Mycerobas carnipes	White-winged Grosbeak	U	L	U	**L**
Mycerobas icterioides	Black-and-yellow Grosbeak	U	U	H	**L**
Mycerobas melanozanthos	Spot-winged Grosbeak	H	H	H	**H**
Mycteria americana	Wood Stork	L	H	L	**L**
Mycteria cinerea	Milky Stork	H	H	L	**L**
Mycteria ibis	Yellow-billed Stork	L	H	L	**L**
Mycteria leucocephala	Painted Stork	L	H	L	**L**
Myiagra albiventris	Samoan Flycatcher	H	U	U	**L**

Myiagra alecto	Shining Flycatcher	U	L	L	**L**
Myiagra atra	Biak Flycatcher	H	U	H	**L**
Myiagra azureocapilla	Blue-crested Flycatcher	H	H	H	**H**
Myiagra caledonica	Melanesian Flycatcher	U	U	L	**L**
Myiagra cervinicauda	Makira Flycatcher	H	H	U	**L**
Myiagra cyanoleuca	Satin Flycatcher	U	L	L	**L**
Myiagra erythrops	Mangrove Flycatcher	H	H	H	**H**
Myiagra ferrocyanea	Steel-blue Flycatcher	H	U	H	**L**
Myiagra galeata	Dark-grey Flycatcher	H	U	H	**L**
Myiagra hebetior	Dull Flycatcher	H	H	H	**H**
Myiagra inquieta	Restless Flycatcher	H	L	L	**L**
Myiagra oceanica	Oceanic Flycatcher	H	U	U	**L**
Myiagra pluto	Pohnpei Flycatcher	H	U	H	**L**
Myiagra rubecula	Leaden Flycatcher	U	L	L	**L**
Myiagra ruficollis	Broad-billed Flycatcher	U	L	L	**L**
Myiagra vanikorensis	Vanikoro Flycatcher	U	H	U	**L**
Myiarchus antillarum	Puerto Rican Flycatcher	H	L	H	**L**
Myiarchus apicalis	Apical Flycatcher	H	U	H	**L**
Myiarchus barbirostris	Sad Flycatcher	H	H	H	**H**
Myiarchus cephalotes	Pale-edged Flycatcher	U	U	L	**L**
Myiarchus cinerascens	Ash-throated Flycatcher	H	L	H	**L**
Myiarchus crinitus	Great Crested Flycatcher	H	L	L	**L**
Myiarchus ferox	Short-crested Flycatcher	H	L	H	**L**
Myiarchus magnirostris	Large-billed Flycatcher	H	L	H	**L**
Myiarchus nugator	Grenada Flycatcher	H	H	H	**H**
Myiarchus nuttingi	Nutting's Flycatcher	L	L	H	**L**
Myiarchus oberi	Lesser Antillean Flycatcher	H	L	H	**L**
Myiarchus panamensis	Panama Flycatcher	H	L	H	**L**
Myiarchus phaeocephalus	Sooty-crowned Flycatcher	H	U	H	**L**
Myiarchus sagrae	La Sagra's Flycatcher	U	L	H	**L**
Myiarchus semirufus	Rufous Flycatcher	H	U	H	**L**
Myiarchus stolidus	Stolid Flycatcher	U	L	H	**L**
Myiarchus swainsoni	Swainson's Flycatcher	H	L	L	**L**
Myiarchus tuberculifer	Dusky-capped Flycatcher	H	L	L	**L**
Myiarchus tyrannulus	Brown-crested Flycatcher	H	H	L	**L**
Myiarchus validus	Rufous-tailed Flycatcher	H	L	H	**L**
Myiarchus venezuelensis	Venezuelan Flycatcher	U	U	H	**L**
Myiarchus yucatanensis	Yucatan Flycatcher	L	U	H	**L**
Myiobius atricaudus	Black-tailed Flycatcher	U	H	L	**L**
Myiobius barbatus	Bearded Flycatcher	H	H	H	**H**
Myiobius sulphureipygius	Sulphur-rumped Flycatcher	H	H	H	**H**
Myiobius villosus	Tawny-breasted Flycatcher	H	H	H	**H**
Myioborus albifacies	White-faced Redstart	H	H	H	**H**
Myioborus albifrons	White-fronted Redstart	H	H	H	**H**
Myioborus brunniceps	Brown-capped Redstart	U	U	H	**L**

Myioborus cardonai	Saffron-breasted Redstart	H	H	H	**H**
Myioborus castaneocapilla	Tepui Redstart	U	H	H	**L**
Myioborus flavivertex	Yellow-crowned Redstart	H	H	H	**H**
Myioborus melanocephalus	Spectacled Redstart	U	H	H	**L**
Myioborus miniatus	Slate-throated Redstart	L	H	L	**L**
Myioborus ornatus	Golden-fronted Redstart	H	H	H	**H**
Myioborus pariae	Paria Redstart	H	H	H	**H**
Myioborus pictus	Painted Redstart	H	L	L	**L**
Myioborus torquatus	Collared Redstart	U	L	H	**L**
Myiodynastes bairdii	Baird's Flycatcher	H	L	H	**L**
Myiodynastes chrysocephalus	Golden-crowned Flycatcher	H	H	L	**L**
Myiodynastes hemichrysus	Golden-bellied Flycatcher	U	L	H	**L**
Myiodynastes luteiventris	Sulphur-bellied Flycatcher	L	L	H	**L**
Myiodynastes maculatus	Streaked Flycatcher	L	L	L	**L**
Myiopagis caniceps	Grey Elaenia	H	H	L	**L**
Myiopagis cotta	Jamaican Elaenia	H	L	H	**L**
Myiopagis flavivertex	Yellow-crowned Elaenia	H	H	H	**H**
Myiopagis gaimardii	Forest Elaenia	H	H	H	**H**
Myiopagis olallai	Foothill Elaenia	H	H	L	**L**
Myiopagis subplacens	Pacific Elaenia	U	U	H	**L**
Myiopagis viridicata	Greenish Elaenia	L	H	L	**L**
Myioparus griseigularis	Grey-throated Tit-flycatcher	H	H	H	**H**
Myioparus plumbeus	Grey Tit-flycatcher	U	H	L	**L**
Myiophobus cryptoxanthus	Olive-chested Flycatcher	U	U	H	**L**
Myiophobus fasciatus	Bran-coloured Flycatcher	U	H	L	**L**
Myiophobus flavicans	Flavescent Flycatcher	H	H	H	**H**
Myiophobus inornatus	Unadorned Flycatcher	H	H	H	**H**
Myiophobus lintoni	Orange-banded Flycatcher	H	H	H	**H**
Myiophobus ochraceiventris	Ochraceous-breasted Flycatcher	U	U	H	**L**
Myiophobus phoenicomitra	Orange-crested Flycatcher	H	H	H	**H**
Myiophobus pulcher	Handsome Flycatcher	H	H	H	**H**
Myiophobus roraimae	Roraiman Flycatcher	H	H	H	**H**
Myiopsitta monachus	Monk Parakeet	H	H	L	**L**
Myiornis albiventris	White-bellied Pygmy-tyrant	H	H	H	**H**
Myiornis atricapillus	Black-capped Pygmy-tyrant	H	H	L	**L**
Myiornis auricularis	Eared Pygmy-tyrant	H	U	L	**L**
Myiornis ecaudatus	Short-tailed Pygmy-tyrant	H	H	H	**H**
Myiotheretes fumigatus	Smoky Bush-tyrant	U	U	H	**L**
Myiotheretes fuscorufus	Rufous-bellied Bush-tyrant	U	U	H	**L**

Myiotheretes pernix	Santa Marta Bush-tyrant	H	H	H	**H**
Myiotheretes striaticollis	Streak-throated Bush-tyrant	U	U	L	**L**
Myiotriccus ornatus	Ornate Flycatcher	H	H	H	**H**
Myiozetetes cayanensis	Rusty-margined Flycatcher	U	L	H	**L**
Myiozetetes granadensis	Grey-capped Flycatcher	H	L	L	**L**
Myiozetetes luteiventris	Dusky-chested Flycatcher	H	U	H	**L**
Myiozetetes similis	Social Flycatcher	L	L	H	**L**
Myophonus blighi	Sri Lanka Whistling-thrush	H	H	H	**H**
Myophonus borneensis	Bornean Whistling-thrush	H	H	U	**L**
Myophonus caeruleus	Blue Whistling-thrush	H	H	H	**H**
Myophonus castaneus	Chestnut-winged Whistling-thrush	H	H	H	**H**
Myophonus glaucinus	Javan Whistling Thrush	H	H	U	**L**
Myophonus horsfieldii	Malabar Whistling-thrush	H	L	U	**L**
Myophonus insularis	Taiwan Whistling-thrush	H	H	H	**H**
Myophonus melanurus	Shiny Whistling-thrush	H	H	H	**H**
Myophonus robinsoni	Malaysian Whistling-thrush	H	H	H	**H**
Myornis senilis	Ash-coloured Tapaculo	H	H	H	**H**
Myrmeciza atrothorax	Black-throated Antbird	H	U	H	**L**
Myrmeciza berlepschi	Stub-tailed Antbird	H	H	H	**H**
Myrmeciza castanea	Zimmer's Antbird	H	H	H	**H**
Myrmeciza disjuncta	Yapacana Antbird	H	U	H	**L**
Myrmeciza exsul	Chestnut-backed Antbird	H	H	L	**L**
Myrmeciza ferruginea	Ferruginous-backed Antbird	H	H	H	**H**
Myrmeciza fortis	Sooty Antbird	H	H	L	**L**
Myrmeciza goeldii	Goeldi's Antbird	H	H	H	**H**
Myrmeciza griseiceps	Grey-headed Antbird	H	H	H	**H**
Myrmeciza hemimelaena	Chestnut-tailed Antbird	H	H	L	**L**
Myrmeciza hyperythra	Plumbeous Antbird	H	H	L	**L**
Myrmeciza immaculata	Immaculate Antbird	H	U	H	**L**
Myrmeciza laemosticta	Dull-mantled Antbird	H	H	L	**L**
Myrmeciza longipes	White-bellied Antbird	U	H	H	**L**
Myrmeciza loricata	White-bibbed Antbird	H	H	H	**H**
Myrmeciza melanoceps	White-shouldered Antbird	H	H	L	**L**
Myrmeciza nigricauda	Esmeraldas Antbird	H	H	H	**H**
Myrmeciza pelzelni	Grey-bellied Antbird	H	U	H	**L**
Myrmeciza ruficauda	Scalloped Antbird	H	H	H	**H**
Myrmeciza squamosa	Squamate Antbird	H	U	L	**L**
Myrmecocichla aethiops	Northern Anteater-chat	U	U	U	**L**
Myrmecocichla albifrons	White-fronted Black-chat	U	L	U	**L**
Myrmecocichla arnoti	White-headed Black-chat	H	L	U	**L**
Myrmecocichla cinnamomeiventris	Mocking Cliff-chat	U	L	U	**L**
Myrmecocichla formicivora	Southern Anteater-chat	H	L	H	**L**

Myrmecocichla melaena	Rueppell's Chat	H	U	H	**L**
Myrmecocichla nigra	Sooty Chat	U	L	U	**L**
Myrmecocichla semirufa	White-winged Cliff-chat	U	L	L	**L**
Myrmecocichla tholloni	Congo Moor-chat	U	H	U	**L**
Myrmia micrura	Short-tailed Woodstar	H	H	H	**H**
Myrmoborus leucophrys	White-browed Antbird	H	H	H	**H**
Myrmoborus lugubris	Ash-breasted Antbird	H	H	H	**H**
Myrmoborus melanurus	Black-tailed Antbird	H	H	H	**H**
Myrmoborus myotherinus	Black-faced Antbird	H	H	H	**H**
Myrmochanes hemileucus	Black-and-white Antbird	H	U	L	**L**
Myrmorchilus strigilatus	Stripe-backed Antbird	H	H	L	**L**
Myrmornis torquata	Wing-banded Antbird	H	H	H	**H**
Myrmothera campanisona	Thrush-like Antpitta	H	H	H	**H**
Myrmothera simplex	Brown-breasted Antpitta	U	H	H	**L**
Myrmotherula ambigua	Yellow-throated Antwren	H	U	H	**L**
Myrmotherula assimilis	Leaden Antwren	H	U	L	**L**
Myrmotherula axillaris	White-flanked Antwren	H	H	H	**H**
Myrmotherula behni	Plain-winged Antwren	H	H	H	**H**
Myrmotherula brachyura	Pygmy Antwren	H	H	H	**H**
Myrmotherula cherriei	Cherrie's Antwren	H	U	H	**L**
Myrmotherula fluminensis	Rio de Janeiro Antwren	H	H	H	**H**
Myrmotherula grisea	Ashy Antwren	H	H	H	**H**
Myrmotherula gularis	Star-throated Antwren	H	H	L	**L**
Myrmotherula guttata	Rufous-bellied Antwren	H	H	H	**H**
Myrmotherula gutturalis	Brown-bellied Antwren	H	H	H	**H**
Myrmotherula hauxwelli	Plain-throated Antwren	H	H	L	**L**
Myrmotherula ignota	Moustached Antwren	H	H	L	**L**
Myrmotherula iheringi	Ihering's Antwren	H	H	L	**L**
Myrmotherula klagesi	Klages's Antwren	H	H	H	**H**
Myrmotherula longicauda	Stripe-chested Antwren	U	U	H	**L**
Myrmotherula longipennis	Long-winged Antwren	H	H	H	**H**
Myrmotherula menetriesii	Grey Antwren	H	U	H	**L**
Myrmotherula minor	Salvadori's Antwren	H	H	H	**H**
Myrmotherula multostriata	Amazonian Streaked Antwren	H	U	H	**L**
Myrmotherula pacifica	Pacific Antwren	U	H	H	**L**
Myrmotherula schisticolor	Slaty Antwren	H	H	L	**L**
Myrmotherula sclateri	Sclater's Antwren	H	H	L	**L**
Myrmotherula snowi	Alagoas Antwren	H	H	H	**H**
Myrmotherula sunensis	Rio Suno Antwren	H	H	H	**H**
Myrmotherula surinamensis	Guianan Streaked Antwren	H	H	H	**H**
Myrmotherula unicolor	Unicoloured Antwren	H	H	H	**H**

Myrmotherula urosticta	Band-tailed Antwren	H	H	H	**H**
Myrtis fanny	Purple-collared Woodstar	H	H	H	**H**
Mystacornis crossleyi	Crossley's Babbler	H	H	U	**L**
Myza celebensis	Dark-eared Myza	H	H	H	**H**
Myza sarasinorum	White-eared Myza	H	H	H	**H**
Myzomela adolphinae	Mountain Myzomela	U	H	L	**L**
Myzomela albigula	White-chinned Myzomela	H	H	H	**H**
Myzomela blasii	Drab Myzomela	H	H	H	**H**
Myzomela boiei	Banda Myzomela	H	H	L	**L**
Myzomela caledonica	New Caledonian Myzomela	U	H	H	**L**
Myzomela cardinalis	Cardinal Myzomela	H	H	L	**L**
Myzomela chermesina	Rotuma Myzomela	H	H	U	**L**
Myzomela chloroptera	Sulawesi Myzomela	H	H	H	**H**
Myzomela cruentata	Red Myzomela	U	H	L	**L**
Myzomela dammermani	Sumba Myzomela	H	H	L	**L**
Myzomela eichhorni	Yellow-vented Myzomela	H	H	H	**H**
Myzomela eques	Red-throated Myzomela	H	H	L	**L**
Myzomela erythrocephala	Red-headed Myzomela	U	H	L	**L**
Myzomela erythromelas	Black-bellied Myzomela	H	H	H	**H**
Myzomela jugularis	Orange-breasted Myzomela	H	H	L	**L**
Myzomela kuehni	Crimson-hooded Myzomela	H	H	L	**L**
Myzomela lafargei	Scarlet-naped Myzomela	H	H	H	**H**
Myzomela malaitae	Red-bellied Myzomela	U	H	U	**L**
Myzomela melanocephala	Black-headed Myzomela	H	H	H	**H**
Myzomela nigrita	Black Myzomela	U	H	L	**L**
Myzomela obscura	Dusky Myzomela	U	H	L	**L**
Myzomela pammelaena	Ebony Myzomela	H	H	H	**H**
Myzomela pulchella	Olive-yellow Myzomela	H	H	H	**H**
Myzomela rosenbergii	Red-collared Myzomela	U	H	H	**L**
Myzomela rubratra	Micronesian Myzomela	H	H	H	**H**
Myzomela sanguinolenta	Scarlet Myzomela	H	H	L	**L**
Myzomela sclateri	Scarlet-bibbed Myzomela	H	H	H	**H**
Myzomela tristrami	Sooty Myzomela	H	H	H	**H**
Myzomela vulnerata	Red-rumped Myzomela	U	H	H	**L**
Myzomela wakoloensis	Wakolo Myzomela	H	H	H	**H**
Myzornis pyrrhoura	Fire-tailed Myzornis	U	U	U	**L**
Namibornis herero	Herero Chat	H	H	H	**H**
Nandayus nenday	Nanday Parakeet	H	H	L	**L**
Nannopsittaca dachilleae	Amazonian Parrotlet	H	U	H	**L**
Nannopsittaca panychlora	Tepui Parrotlet	H	U	H	**L**
Napothera brevicaudata	Streaked Wren-babbler	H	H	U	**L**
Napothera crassa	Mountain Wren-babbler	H	H	H	**H**
Napothera epilepidota	Eyebrowed Wren-babbler	H	H	U	**L**
Nasica longirostris	Long-billed Woodcreeper	H	U	H	**L**

Neafrapus boehmi	Bat-like Spinetail	U	H	L	**L**
Neafrapus cassini	Cassin's Spinetail	H	H	L	**L**
Necrosyrtes monachus	Hooded Vulture	L	H	L	**L**
Nectarinia adelberti	Buff-throated Sunbird	H	L	H	**L**
Nectarinia afra	Greater Double-collared Sunbird	U	H	U	**L**
Nectarinia alinae	Blue-headed Sunbird	H	H	L	**L**
Nectarinia amethystina	Amethyst Sunbird	U	L	L	**L**
Nectarinia asiatica	Purple Sunbird	U	H	L	**L**
Nectarinia aspasia	Black Sunbird	U	L	U	**L**
Nectarinia balfouri	Socotra Sunbird	H	L	H	**L**
Nectarinia bannermani	Bannerman's Sunbird	H	H	H	**H**
Nectarinia batesi	Bates's Sunbird	H	H	H	**H**
Nectarinia bifasciata	Purple-banded Sunbird	U	H	L	**L**
Nectarinia bocagii	Bocage's Sunbird	U	H	H	**L**
Nectarinia bouvieri	Orange-tufted Sunbird	H	H	H	**H**
Nectarinia buettikoferi	Apricot-breasted Sunbird	H	U	H	**L**
Nectarinia calcostetha	Copper-throated Sunbird	U	H	U	**L**
Nectarinia chalcomelas	Violet-breasted Sunbird	H	U	U	**L**
Nectarinia chalybea	Southern Double-collared Sunbird	H	H	L	**L**
Nectarinia chloropygia	Olive-bellied Sunbird	H	H	L	**L**
Nectarinia coccinigaster	Splendid Sunbird	U	H	H	**L**
Nectarinia comorensis	Anjouan Sunbird	H	H	H	**H**
Nectarinia congensis	Congo Sunbird	H	U	H	**L**
Nectarinia coquerellii	Mayotte Sunbird	H	U	H	**L**
Nectarinia cuprea	Copper Sunbird	U	H	L	**L**
Nectarinia cyanolaema	Blue-throated Brown Sunbird	H	H	L	**L**
Nectarinia dussumieri	Seychelles Sunbird	H	H	L	**L**
Nectarinia erythrocerca	Red-chested Sunbird	U	H	L	**L**
Nectarinia famosa	Malachite Sunbird	H	H	L	**L**
Nectarinia fuliginosa	Carmelite Sunbird	H	H	L	**L**
Nectarinia fusca	Dusky Sunbird	U	L	U	**L**
Nectarinia habessinica	Shining Sunbird	U	H	U	**L**
Nectarinia hartlaubii	Principe Sunbird	H	H	L	**L**
Nectarinia humbloti	Humblot's Sunbird	U	H	H	**L**
Nectarinia hunteri	Hunter's Sunbird	U	H	U	**L**
Nectarinia johannae	Johanna's Sunbird	H	H	L	**L**
Nectarinia johnstoni	Red-tufted Sunbird	U	H	H	**L**
Nectarinia jugularis	Olive-backed Sunbird	U	H	L	**L**
Nectarinia kilimensis	Bronze Sunbird	U	H	L	**L**
Nectarinia lotenia	Long-billed Sunbird	U	L	U	**L**
Nectarinia loveridgei	Loveridge's Sunbird	L	H	H	**L**
Nectarinia manoensis	Miombo Double-collared	U	H	U	**L**

	Sunbird				
Nectarinia mariquensis	Mariqua Sunbird	U	H	L	**L**
Nectarinia mediocris	Eastern Double-collared Sunbird	H	H	H	**H**
Nectarinia minima	Crimson-backed Sunbird	U	H	H	**L**
Nectarinia minulla	Tiny Sunbird	H	H	L	**L**
Nectarinia moreaui	Moreau's Sunbird	H	H	L	**L**
Nectarinia nectarinioides	Black-bellied Sunbird	U	H	U	**L**
Nectarinia neergardi	Neergaard's Sunbird	H	H	L	**L**
Nectarinia newtonii	Newton's Sunbird	H	H	L	**L**
Nectarinia notata	Long-billed Green Sunbird	U	H	L	**L**
Nectarinia olivacea	Olive Sunbird	U	H	L	**L**
Nectarinia oritis	Cameroon Sunbird	U	H	L	**L**
Nectarinia osea	Palestine Sunbird	H	H	L	**L**
Nectarinia oustaleti	Oustalet's Sunbird	U	U	U	**L**
Nectarinia pembae	Pemba Sunbird	U	H	U	**L**
Nectarinia preussi	Northern Double-collared Sunbird	U	H	H	**L**
Nectarinia pulchella	Beautiful Sunbird	U	H	H	**L**
Nectarinia purpureiventris	Purple-breasted Sunbird	H	H	L	**L**
Nectarinia regia	Regal Sunbird	U	H	H	**L**
Nectarinia reichenbachii	Reichenbach's Sunbird	H	H	L	**L**
Nectarinia reichenowi	Golden-winged Sunbird	U	H	H	**L**
Nectarinia rockefelleri	Rockefeller's Sunbird	H	H	H	**H**
Nectarinia rubescens	Green-throated Sunbird	H	H	L	**L**
Nectarinia rufipennis	Rufous-winged Sunbird	H	H	H	**H**
Nectarinia seimundi	Little Green Sunbird	H	H	L	**L**
Nectarinia senegalensis	Scarlet-chested Sunbird	U	H	L	**L**
Nectarinia shelleyi	Shelley's Sunbird	U	H	U	**L**
Nectarinia solaris	Flame-breasted Sunbird	H	U	L	**L**
Nectarinia sovimanga	Souimanga Sunbird	U	H	U	**L**
Nectarinia sperata	Purple-throated Sunbird	U	H	U	**L**
Nectarinia superba	Superb Sunbird	H	H	L	**L**
Nectarinia tacazze	Tacazze Sunbird	U	H	L	**L**
Nectarinia talatala	White-breasted Sunbird	U	H	U	**L**
Nectarinia thomensis	Giant Sunbird	H	H	L	**L**
Nectarinia ursulae	Ursula's Sunbird	U	H	L	**L**
Nectarinia venusta	Variable Sunbird	U	H	L	**L**
Nectarinia veroxii	Mouse-coloured Sunbird	H	H	L	**L**
Nectarinia verticalis	Green-headed Sunbird	U	H	H	**L**
Nectarinia violacea	Orange-breasted Sunbird	H	H	U	**L**
Nectarinia zeylonica	Purple-rumped Sunbird	U	L	H	**L**
Nemosia pileata	Hooded Tanager	H	H	L	**L**
Nemosia rourei	Cherry-throated Tanager	H	H	H	**H**
Neochelidon tibialis	White-thighed Swallow	H	U	H	**L**

Neochen jubata	Orinoco Goose	H	H	H	**H**
Neochmia modesta	Plum-headed Finch	U	L	U	**L**
Neochmia phaeton	Crimson Finch	U	L	U	**L**
Neochmia ruficauda	Star Finch	L	L	L	**L**
Neochmia temporalis	Red-browed Finch	H	L	L	**L**
Neocichla gutturalis	Babbling Starling	H	H	U	**L**
Neocossyphus poensis	White-tailed Ant-thrush	H	U	L	**L**
Neocossyphus rufus	Red-tailed Ant-thrush	H	H	H	**H**
Neocrex colombiana	Colombian Crake	H	U	H	**L**
Neocrex erythrops	Paint-billed Crake	U	L	L	**L**
Neoctantes niger	Black Bushbird	H	H	H	**H**
Neodrepanis coruscans	Sunbird Asity	H	H	H	**H**
Neodrepanis hypoxantha	Yellow-bellied Asity	H	H	H	**H**
Neolalage banksiana	Buff-bellied Monarch	H	U	H	**L**
Neolestes torquatus	Black-collared Bulbul	U	H	U	**L**
Neomixis flavoviridis	Wedge-tailed Jery	H	H	H	**H**
Neomixis striatigula	Stripe-throated Jery	U	L	U	**L**
Neomixis tenella	Common Jery	U	H	U	**L**
Neomixis viridis	Green Jery	H	H	U	**L**
Neomorphus geoffroyi	Rufous-vented Ground-cuckoo	U	H	H	**L**
Neomorphus pucheranii	Red-billed Ground-cuckoo	H	U	L	**L**
Neomorphus radiolosus	Banded Ground-cuckoo	H	U	H	**L**
Neomorphus rufipennis	Rufous-winged Ground-cuckoo	H	U	H	**L**
Neomorphus squamiger	Scaled Ground-cuckoo	H	H	H	**H**
Neopelma aurifrons	Wied's Tyrant-manakin	H	H	H	**H**
Neopelma chrysocephalum	Saffron-crested Tyrant-manakin	H	U	H	**L**
Neopelma chrysolophum	Serra do Mar Tyrant-manakin	H	H	L	**L**
Neopelma pallescens	Pale-bellied Tyrant-manakin	H	H	L	**L**
Neopelma sulphureiventer	Sulphur-bellied Tyrant-manakin	H	H	L	**L**
Neophema chrysogaster	Orange-bellied Parrot	H	L	H	**L**
Neophema chrysostoma	Blue-winged Parrot	U	L	U	**L**
Neophema elegans	Elegant Parrot	U	H	U	**L**
Neophema petrophila	Rock Parrot	H	H	H	**H**
Neophema pulchella	Turquoise Parrot	U	H	U	**L**
Neophema splendida	Scarlet-chested Parrot	H	L	U	**L**
Neophron percnopterus	Egyptian Vulture	H	H	H	**H**
Neopipo cinnamomea	Cinnamon Manakin-tyrant	H	H	H	**H**
Neopsephotus bourkii	Bourke's Parrot	H	L	H	**L**
Neopsittacus musschenbroekii	Yellow-billed Lorikeet	U	H	U	**L**
Neopsittacus pullicauda	Orange-billed Lorikeet	H	H	U	**L**

Neospiza concolor	Sao Tome Grosbeak	H	H	L	**L**
Neothraupis fasciata	White-banded Tanager	H	L	L	**L**
Neotis denhami	Denham's Bustard	U	H	H	**L**
Neotis heuglinii	Heuglin's Bustard	U	H	U	**L**
Neotis ludwigii	Ludwig's Bustard	H	H	H	**H**
Neotis nuba	Nubian Bustard	H	H	H	**H**
Neoxolmis rufiventris	Chocolate-vented Tyrant	H	L	H	**L**
Nephelornis oneilli	Pardusco	H	U	H	**L**
Nesasio solomonensis	Fearful Owl	H	U	L	**L**
Nesillas brevicaudata	Grand Comoro Brush-warbler	U	H	H	**L**
Nesillas lantzii	Lantz's Brush-warbler	H	U	L	**L**
Nesillas longicaudata	Anjouan Brush-warbler	H	H	H	**H**
Nesillas mariae	Moheli Warbler	H	H	H	**H**
Nesillas typica	Madagascar Brush-warbler	U	H	U	**L**
Nesocharis ansorgei	White-collared Oliveback	H	L	L	**L**
Nesocharis capistrata	White-cheeked Oliveback	U	U	H	**L**
Nesocharis shelleyi	Fernando Po Oliveback	U	U	L	**L**
Nesocichla eremita	Tristan Thrush	H	L	U	**L**
Nesoclopeus woodfordi	Woodford's Rail	H	L	H	**L**
Nesoctites micromegas	Antillean Piculet	U	L	H	**L**
Nesoenas mayeri	Pink Pigeon	H	H	H	**H**
Nesoenas picturata	Madagascar Turtle-dove	U	H	U	**L**
Nesofregetta fuliginosa	White-throated Storm-petrel	H	H	L	**L**
Nesopsar nigerrimus	Jamaican Blackbird	H	H	H	**H**
Nesospingus speculiferus	Puerto Rican Tanager	U	H	U	**L**
Nesospiza acunhae	Tristan Bunting	H	H	U	**L**
Nesospiza wilkinsi	Grosbeak Bunting	H	H	U	**L**
Nesotriccus ridgwayi	Cocos Flycatcher	H	H	U	**L**
Nestor meridionalis	Kaka	H	H	H	**H**
Nestor notabilis	Kea	H	H	H	**H**
Netta erythrophthalma	Southern Pochard	L	H	L	**L**
Netta peposaca	Rosy-billed Pochard	U	H	U	**L**
Netta rufina	Red-crested Pochard	H	H	L	**L**
Nettapus auritus	African Pygmy-goose	H	H	L	**L**
Nettapus coromandelianus	Cotton Pygmy-goose	H	H	U	**L**
Nettapus pulchellus	Green Pygmy-goose	H	H	U	**L**
Newtonia amphichroa	Dark Newtonia	U	U	U	**L**
Newtonia archboldi	Archbold's Newtonia	H	U	L	**L**
Newtonia brunneicauda	Common Newtonia	U	U	U	**L**
Newtonia fanovanae	Red-tailed Newtonia	H	H	H	**H**
Nicator chloris	Yellow-spotted Nicator	H	H	L	**L**
Nicator gularis	Eastern Nicator	U	L	L	**L**
Nicator vireo	Yellow-throated Nicator	H	H	H	**H**

Nigrita bicolor	Chestnut-breasted Negrofinch	U	H	U	**L**
Nigrita canicapillus	Grey-headed Negrofinch	U	L	U	**L**
Nigrita fusconotus	White-breasted Negrofinch	H	L	L	**L**
Nigrita luteifrons	Pale-fronted Negrofinch	U	U	U	**L**
Nilaus afer	Brubru	U	H	L	**L**
Niltava davidi	Fujian Niltava	H	U	U	**L**
Niltava grandis	Large Niltava	U	L	U	**L**
Niltava macgrigoriae	Small Niltava	U	L	U	**L**
Niltava sumatrana	Rufous-vented Niltava	H	H	H	**H**
Niltava sundara	Rufous-bellied Niltava	U	L	H	**L**
Niltava vivida	Vivid Niltava	H	H	U	**L**
Ninox affinis	Andaman Hawk-owl	H	U	L	**L**
Ninox burhani	Togian Hawk-owl	H	H	H	**H**
Ninox connivens	Barking Owl	U	H	U	**L**
Ninox ios	Cinnabar Hawk-owl	H	H	H	**H**
Ninox jacquinoti	Solomons Hawk-owl	H	U	H	**L**
Ninox meeki	Manus Hawk-owl	H	U	H	**L**
Ninox natalis	Christmas Hawk-owl	H	H	U	**L**
Ninox novaeseelandiae	Southern Boobook	H	L	L	**L**
Ninox ochracea	Ochre-bellied Hawk-owl	H	U	H	**L**
Ninox odiosa	Russet Hawk-owl	H	U	H	**L**
Ninox philippensis	Philippine Hawk-owl	U	U	U	**L**
Ninox punctulata	Speckled Hawk-owl	U	U	U	**L**
Ninox rudolfi	Sumba Boobook	H	U	H	**L**
Ninox rufa	Rufous Owl	H	H	U	**L**
Ninox scutulata	Brown Hawk-owl	U	L	U	**L**
Ninox squamipila	Moluccan Hawk-owl	U	H	U	**L**
Ninox strenua	Powerful Owl	H	H	U	**L**
Ninox sumbaensis	Little Sumba Hawk-owl	H	U	H	**L**
Ninox superciliaris	White-browed Hawk-owl	U	L	U	**L**
Ninox theomacha	Jungle Hawk-owl	U	H	U	**L**
Ninox variegata	Bismarck Hawk-owl	H	U	H	**L**
Nipponia nippon	Asian Crested Ibis	H	H	H	**H**
Nomonyx dominicus	Masked Duck	L	H	L	**L**
Nonnula amaurocephala	Chestnut-headed Nunlet	H	U	H	**L**
Nonnula brunnea	Brown Nunlet	H	U	H	**L**
Nonnula frontalis	Grey-cheeked Nunlet	U	U	H	**L**
Nonnula rubecula	Rusty-breasted Nunlet	U	U	H	**L**
Nonnula ruficapilla	Rufous-capped Nunlet	H	U	L	**L**
Nonnula sclateri	Fulvous-chinned Nunlet	H	U	L	**L**
Northiella haematogaster	Bluebonnet	H	L	L	**L**
Notharchus hyperrhynchus	White-necked Puffbird	H	U	H	**L**
Notharchus	Guianan Puffbird	H	U	H	**L**

macrorhynchos					
Notharchus ordii	Brown-banded Puffbird	H	U	H	**L**
Notharchus pectoralis	Black-breasted Puffbird	H	L	H	**L**
Notharchus swainsoni	Buff-bellied Puffbird	H	U	L	**L**
Notharchus tectus	Pied Puffbird	H	H	H	**H**
Nothocercus bonapartei	Highland Tinamou	H	H	H	**H**
Nothocercus julius	Tawny-breasted Tinamou	H	H	H	**H**
Nothocercus nigrocapillus	Hooded Tinamou	H	H	H	**H**
Nothocrax urumutum	Nocturnal Curassow	H	H	H	**H**
Nothoprocta cinerascens	Brushland Tinamou	H	H	L	**L**
Nothoprocta curvirostris	Curve-billed Tinamou	H	H	H	**H**
Nothoprocta ornata	Ornate Tinamou	H	H	H	**H**
Nothoprocta pentlandii	Andean Tinamou	H	H	H	**H**
Nothoprocta perdicaria	Chilean Tinamou	U	H	L	**L**
Nothoprocta taczanowskii	Taczanowski's Tinamou	H	H	H	**H**
Nothura boraquira	White-bellied Nothura	U	H	L	**L**
Nothura chacoensis	Chaco Nothura	H	H	L	**L**
Nothura darwinii	Darwin's Nothura	H	H	H	**H**
Nothura maculosa	Spotted Nothura	U	H	L	**L**
Nothura minor	Lesser Nothura	H	H	L	**L**
Notiochelidon flavipes	Pale-footed Swallow	H	U	L	**L**
Notiochelidon murina	Brown-bellied Swallow	U	L	H	**L**
Notiochelidon pileata	Black-capped Swallow	L	L	H	**L**
Notiomystis cincta	Stitchbird	H	L	H	**L**
Nucifraga caryocatactes	Spotted Nutcracker	H	H	H	**H**
Nucifraga columbiana	Clark's Nutcracker	H	H	H	**H**
Numenius americanus	Long-billed Curlew	L	H	H	**L**
Numenius arquata	Eurasian Curlew	H	H	H	**H**
Numenius borealis	Eskimo Curlew	H	H	H	**H**
Numenius madagascariensis	Far Eastern Curlew	L	H	H	**L**
Numenius minutus	Little Curlew	H	H	L	**L**
Numenius phaeopus	Whimbrel	L	H	H	**L**
Numenius tahitiensis	Bristle-thighed Curlew	H	H	H	**H**
Numenius tenuirostris	Slender-billed Curlew	H	H	H	**H**
Numida meleagris	Helmeted Guineafowl	L	L	L	**L**
Nyctanassa violacea	Yellow-crowned Night-heron	L	L	L	**L**
Nyctibius aethereus	Long-tailed Potoo	H	H	H	**H**
Nyctibius bracteatus	Rufous Potoo	H	H	H	**H**
Nyctibius grandis	Great Potoo	H	H	H	**H**
Nyctibius griseus	Grey Potoo	U	H	L	**L**
Nyctibius jamaicensis	Northern Potoo	L	H	H	**L**
Nyctibius leucopterus	White-winged Potoo	H	H	H	**H**
Nyctibius maculosus	Andean Potoo	H	H	H	**H**
Nycticorax caledonicus	Rufous Night-heron	U	H	U	**L**

Nycticorax nycticorax	Black-crowned Night-heron	L	H	L	**L**
Nyctidromus albicollis	Common Pauraque	L	H	L	**L**
Nyctiphrynus mcleodii	Eared Poorwill	H	H	L	**L**
Nyctiphrynus ocellatus	Ocellated Poorwill	H	H	L	**L**
Nyctiphrynus rosenbergi	Choco Poorwill	H	U	H	**L**
Nyctiphrynus yucatanicus	Yucatan Poorwill	L	U	H	**L**
Nyctiprogne leucopyga	Band-tailed Nighthawk	H	U	H	**L**
Nyctiprogne vielliardi	Plain-tailed Nighthawk	H	U	H	**L**
Nyctyornis amictus	Red-bearded Bee-eater	H	H	H	**H**
Nyctyornis athertoni	Blue-bearded Bee-eater	H	H	H	**H**
Nymphicus hollandicus	Cockatiel	H	H	L	**L**
Nystalus chacuru	White-eared Puffbird	U	L	L	**L**
Nystalus maculatus	Spot-backed Puffbird	U	L	L	**L**
Nystalus radiatus	Barred Puffbird	U	U	H	**L**
Nystalus striolatus	Striolated Puffbird	H	L	L	**L**
Oceanites gracilis	White-vented Storm-petrel	U	H	H	**L**
Oceanites maorianus	New Zealand Storm-petrel	H	H	H	**H**
Oceanites oceanicus	Wilson's Storm-petrel	L	H	L	**L**
Oceanodroma castro	Madeiran Storm-petrel	L	H	L	**L**
Oceanodroma furcata	Fork-tailed Storm-petrel	H	H	L	**L**
Oceanodroma homochroa	Ashy Storm-petrel	H	H	H	**H**
Oceanodroma hornbyi	Ringed Storm-petrel	H	H	U	**L**
Oceanodroma leucorhoa	Leach's Storm-petrel	L	H	L	**L**
Oceanodroma macrodactyla	Guadalupe Storm-petrel	H	H	H	**H**
Oceanodroma markhami	Markham's Storm-petrel	H	H	U	**L**
Oceanodroma matsudairae	Matsudaira's Storm-petrel	L	H	L	**L**
Oceanodroma melania	Black Storm-petrel	L	H	L	**L**
Oceanodroma monorhis	Swinhoe's Storm-petrel	L	H	L	**L**
Oceanodroma tethys	Wedge-rumped Storm-petrel	L	H	H	**L**
Oceanodroma tristrami	Tristram's Storm-petrel	L	H	L	**L**
Ochthoeca cinnamomeiventris	Slaty-backed Chat-tyrant	H	U	L	**L**
Ochthoeca diadema	Yellow-bellied Chat-tyrant	U	L	H	**L**
Ochthoeca frontalis	Crowned Chat-tyrant	U	U	H	**L**
Ochthoeca fumicolor	Brown-backed Chat-tyrant	U	U	H	**L**
Ochthoeca jelskii	Jelski's Chat-tyrant	H	U	H	**L**
Ochthoeca leucophrys	White-browed Chat-tyrant	H	H	H	**H**
Ochthoeca oenanthoides	D'Orbigny's Chat-tyrant	H	U	H	**L**
Ochthoeca piurae	Piura Chat-tyrant	H	U	H	**L**
Ochthoeca pulchella	Golden-browed Chat-tyrant	U	U	H	**L**
Ochthoeca rufipectoralis	Rufous-breasted Chat-tyrant	H	U	L	**L**
Ochthornis littoralis	Drab Water-tyrant	H	L	H	**L**
Ocreatus underwoodii	Booted Racket-tail	H	H	L	**L**

Oculocincta squamifrons	Pygmy White-eye	H	H	H	**H**
Ocyalus latirostris	Band-tailed Oropendola	H	U	U	**L**
Ocyceros birostris	Indian Grey Hornbill	H	H	L	**L**
Ocyceros gingalensis	Sri Lanka Grey Hornbill	H	H	H	**H**
Ocyceros griseus	Malabar Grey Hornbill	H	H	H	**H**
Ocyphaps lophotes	Crested Pigeon	U	H	U	**L**
Odontophorus atrifrons	Black-fronted Wood-quail	H	U	H	**L**
Odontophorus balliviani	Stripe-faced Wood-quail	H	U	H	**L**
Odontophorus capueira	Spot-winged Wood-quail	H	L	L	**L**
Odontophorus columbianus	Venezuelan Wood-quail	H	L	H	**L**
Odontophorus dialeucos	Tacarcuna Wood-quail	H	H	H	**H**
Odontophorus erythrops	Rufous-fronted Wood-quail	H	U	H	**L**
Odontophorus gujanensis	Marbled Wood-quail	H	L	H	**L**
Odontophorus guttatus	Spotted Wood-quail	H	U	H	**L**
Odontophorus hyperythrus	Chestnut Wood-quail	H	U	H	**L**
Odontophorus leucolaemus	Black-breasted Wood-quail	H	L	H	**L**
Odontophorus melanonotus	Dark-backed Wood-quail	H	U	H	**L**
Odontophorus melanotis	Black-eared Wood-quail	H	L	H	**L**
Odontophorus speciosus	Rufous-breasted Wood-quail	H	U	H	**L**
Odontophorus stellatus	Starred Wood-quail	H	U	L	**L**
Odontophorus strophium	Gorgeted Wood-quail	H	U	H	**L**
Odontorchilus branickii	Grey-mantled Wren	H	H	H	**H**
Odontorchilus cinereus	Tooth-billed Wren	H	H	H	**H**
Oedistoma pygmaeum	Pygmy Longbill	H	U	L	**L**
Oena capensis	Namaqua Dove	U	H	L	**L**
Oenanthe albonigra	Hume's Wheatear	H	L	H	**L**
Oenanthe bifasciata	Buff-streaked Chat	H	H	L	**L**
Oenanthe bottae	Botta's Wheatear	U	L	U	**L**
Oenanthe chrysopygia	Red-tailed Wheatear	H	U	L	**L**
Oenanthe cypriaca	Cyprus Wheatear	H	L	H	**L**
Oenanthe deserti	Desert Wheatear	H	L	H	**L**
Oenanthe finschii	Finsch's Wheatear	H	L	H	**L**
Oenanthe hispanica	Black-eared Wheatear	H	L	H	**L**
Oenanthe isabellina	Isabelline Wheatear	H	L	H	**L**
Oenanthe leucopyga	White-tailed Wheatear	H	L	H	**L**
Oenanthe leucura	Black Wheatear	H	L	L	**L**
Oenanthe lugens	Mourning Wheatear	H	L	L	**L**
Oenanthe lugentoides	Arabian Wheatear	U	L	U	**L**
Oenanthe moesta	Red-rumped Wheatear	H	L	H	**L**
Oenanthe monacha	Hooded Wheatear	U	L	U	**L**
Oenanthe monticola	Mountain Wheatear	H	L	L	**L**

Oenanthe oenanthe	Northern Wheatear	H	L	H	**L**
Oenanthe phillipsi	Somali Wheatear	U	L	U	**L**
Oenanthe picata	Variable Wheatear	H	U	H	**L**
Oenanthe pileata	Capped Wheatear	U	L	U	**L**
Oenanthe pleschanka	Pied Wheatear	H	L	H	**L**
Oenanthe xanthoprymna	Red-rumped Wheatear	U	L	U	**L**
Ognorhynchus icterotis	Yellow-eared Parrot	H	H	H	**H**
Oncostoma cinereigulare	Northern Bentbill	L	H	H	**L**
Oncostoma olivaceum	Southern Bentbill	U	H	L	**L**
Onychognathus albirostris	White-billed Starling	H	L	U	**L**
Onychognathus blythii	Somali Starling	U	L	U	**L**
Onychognathus frater	Socotra Starling	H	U	H	**L**
Onychognathus fulgidus	Chestnut-winged Starling	H	U	L	**L**
Onychognathus morio	Red-winged Starling	U	L	U	**L**
Onychognathus nabouroup	Pale-winged Starling	H	L	H	**L**
Onychognathus salvadorii	Bristle-crowned Starling	H	U	U	**L**
Onychognathus tenuirostris	Slender-billed Starling	H	L	L	**L**
Onychognathus tristramii	Tristram's Starling	H	L	H	**L**
Onychognathus walleri	Waller's Starling	H	H	H	**H**
Onychorhynchus coronatus	Amazonian Royal Flycatcher	H	H	H	**H**
Onychorhynchus mexicanus	Northern Royal Flycatcher	H	H	U	**L**
Onychorhynchus occidentalis	Pacific Royal Flycatcher	H	H	H	**H**
Onychorhynchus swainsoni	Atlantic Royal Flycatcher	H	H	L	**L**
Ophrysia superciliosa	Himalayan Quail	H	U	H	**L**
Opisthocomus hoazin	Hoatzin	H	H	H	**H**
Opisthoprora euryptera	Mountain Avocetbill	H	U	H	**L**
Oporornis agilis	Connecticut Warbler	L	L	L	**L**
Oporornis formosus	Kentucky Warbler	L	L	L	**L**
Oporornis philadelphia	Mourning Warbler	H	L	L	**L**
Oporornis tolmiei	MacGillivray's Warbler	H	L	H	**L**
Orchesticus abeillei	Brown Tanager	U	H	L	**L**
Oreocharis arfaki	Tit Berrypecker	H	U	H	**L**
Oreoica gutturalis	Crested Bellbird	H	H	L	**L**
Oreomanes fraseri	Giant Conebill	H	H	H	**H**
Oreomystis bairdi	Akikiki	H	H	H	**H**
Oreomystis mana	Hawaii Creeper	H	H	U	**L**
Oreonympha nobilis	Bearded Mountaineer	U	U	H	**L**
Oreophasis derbianus	Horned Guan	H	H	H	**H**
Oreopholus ruficollis	Tawny-throated Dotterel	H	H	H	**H**
Oreophylax moreirae	Itatiaia Thistletail	H	H	H	**H**

Oreopsar bolivianus	Bolivian Blackbird	U	L	H	**L**
Oreopsittacus arfaki	Plum-faced Lorikeet	U	U	H	**L**
Oreornis chrysogenys	Orange-cheeked Honeyeater	H	H	H	**H**
Oreortyx pictus	Mountain Quail	L	L	L	**L**
Oreoscoptes montanus	Sage Thrasher	H	L	H	**L**
Oreoscopus gutturalis	Fernwren	H	H	L	**L**
Oreostruthus fuliginosus	Mountain Firetail	U	U	H	**L**
Oreothraupis arremonops	Tanager Finch	H	H	H	**H**
Oreotrochilus adela	Wedge-tailed Hillstar	H	U	H	**L**
Oreotrochilus chimborazo	Ecuadorian Hillstar	H	H	H	**H**
Oreotrochilus estella	Andean Hillstar	H	H	H	**H**
Oreotrochilus leucopleurus	White-sided Hillstar	H	H	L	**L**
Oreotrochilus melanogaster	Black-breasted Hillstar	H	H	H	**H**
Origma solitaria	Rockwarbler	H	H	L	**L**
Oriolia bernieri	Bernier's Vanga	H	H	H	**H**
Oriolus albiloris	White-lored Oriole	U	U	H	**L**
Oriolus auratus	African Golden Oriole	U	H	L	**L**
Oriolus bouroensis	Black-eared Oriole	U	U	H	**L**
Oriolus brachyrhynchus	Western Black-headed Oriole	H	H	L	**L**
Oriolus chinensis	Black-naped Oriole	U	H	U	**L**
Oriolus chlorocephalus	Green-headed Oriole	U	U	H	**L**
Oriolus crassirostris	Sao Tome Oriole	H	H	L	**L**
Oriolus cruentus	Black-and-crimson Oriole	H	H	U	**L**
Oriolus flavocinctus	Green Oriole	U	L	L	**L**
Oriolus forsteni	Grey-collared Oriole	H	H	H	**H**
Oriolus hosii	Black Oriole	H	H	H	**H**
Oriolus isabellae	Isabela Oriole	H	H	H	**H**
Oriolus larvatus	African Black-headed Oriole	U	H	L	**L**
Oriolus melanotis	Olive-brown Oriole	H	U	L	**L**
Oriolus mellianus	Silver Oriole	H	U	L	**L**
Oriolus monacha	Dark-headed Oriole	U	U	L	**L**
Oriolus nigripennis	Black-winged Oriole	H	U	L	**L**
Oriolus oriolus	Eurasian Golden Oriole	H	L	H	**L**
Oriolus percivali	Black-tailed Oriole	U	U	H	**L**
Oriolus phaeochromus	Dusky-brown Oriole	H	U	H	**L**
Oriolus sagittatus	Olive-backed Oriole	U	L	U	**L**
Oriolus steerii	Philippine Oriole	U	U	U	**L**
Oriolus szalayi	Brown Oriole	U	H	U	**L**
Oriolus tenuirostris	Slender-billed Oriole	U	L	U	**L**
Oriolus traillii	Maroon Oriole	H	L	H	**L**
Oriolus xanthonotus	Dark-throated Oriole	H	H	H	**H**
Oriolus xanthornus	Black-hooded Oriole	U	L	U	**L**

Oriturus superciliosus	Striped Sparrow	L	L	L	**L**
Ornithion brunneicapillus	Brown-capped Tyrannulet	U	U	L	**L**
Ornithion inerme	White-lored Tyrannulet	H	U	H	**L**
Ornithion semiflavum	Yellow-bellied Tyrannulet	L	U	H	**L**
Ortalis canicollis	Chaco Chachalaca	H	L	L	**L**
Ortalis cinereiceps	Grey-headed Chachalaca	U	L	H	**L**
Ortalis erythroptera	Rufous-headed Chachalaca	H	L	H	**L**
Ortalis garrula	Chestnut-winged Chachalaca	U	L	H	**L**
Ortalis guttata	Speckled Chachalaca	U	U	L	**L**
Ortalis leucogastra	White-bellied Chachalaca	L	L	H	**L**
Ortalis motmot	Little Chachalaca	H	L	H	**L**
Ortalis poliocephala	West Mexican Chachalaca	L	U	H	**L**
Ortalis ruficauda	Rufous-vented Chachalaca	U	L	H	**L**
Ortalis superciliaris	Buff-browed Chachalaca	H	L	H	**L**
Ortalis vetula	Plain Chachalaca	L	L	H	**L**
Ortalis wagleri	Rufous-bellied Chachalaca	L	L	H	**L**
Orthogonys chloricterus	Olive-green Tanager	H	U	L	**L**
Orthonyx novaeguineae	New Guinea Logrunner	H	H	H	**H**
Orthonyx spaldingii	Chowchilla	U	H	H	**L**
Orthonyx temminckii	Logrunner	H	H	L	**L**
Orthopsittaca manilata	Red-bellied Macaw	H	H	H	**H**
Orthorhyncus cristatus	Antillean Crested Hummingbird	H	H	H	**H**
Orthotomus atrogularis	Dark-necked Tailorbird	U	L	U	**L**
Orthotomus castaneiceps	Philippine Tailorbird	U	L	U	**L**
Orthotomus cinereiceps	White-eared Tailorbird	H	U	H	**L**
Orthotomus cuculatus	Mountain Tailorbird	U	L	U	**L**
Orthotomus derbianus	Grey-backed Tailorbird	H	U	L	**L**
Orthotomus frontalis	Rufous-fronted Tailorbird	U	L	U	**L**
Orthotomus heterolaemus	Rufous-headed Tailorbird	H	U	H	**L**
Orthotomus nigriceps	Black-headed Tailorbird	H	H	H	**H**
Orthotomus ruficeps	Ashy Tailorbird	U	L	U	**L**
Orthotomus samarensis	Yellow-breasted Tailorbird	H	H	H	**H**
Orthotomus sepium	Olive-backed Tailorbird	U	U	U	**L**
Orthotomus sericeus	Rufous-tailed Tailorbird	U	L	U	**L**
Orthotomus sutorius	Common Tailorbird	U	L	L	**L**
Ortygospiza atricollis	African Quailfinch	U	L	L	**L**
Ortygospiza gabonensis	Red-billed Quailfinch	U	U	U	**L**
Ortygospiza locustella	Locust Finch	U	L	L	**L**
Ortyxelos meiffrenii	Lark Buttonquail	H	H	H	**H**
Oryzoborus angolensis	Lesser Seed-finch	U	U	H	**L**
Oryzoborus atrirostris	Black-billed Seed-finch	U	U	H	**L**
Oryzoborus crassirostris	Large-billed Seed-finch	H	U	H	**L**
Oryzoborus funereus	Thick-billed Seed-finch	L	H	H	**L**

Oryzoborus maximiliani	Great-billed Seed-finch	U	U	H	**L**
Oryzoborus nuttingi	Nicaraguan Seed-finch	H	H	H	**H**
Otidiphaps nobilis	Pheasant Pigeon	H	H	L	**L**
Otis tarda	Great Bustard	H	H	H	**H**
Otus alfredi	Flores Scops-owl	H	H	H	**H**
Otus alius	Nicobar Scops-owl	H	U	U	**L**
Otus angelinae	Javan Scops-owl	H	H	H	**H**
Otus bakkamoena	Collared Scops-owl	U	L	U	**L**
Otus balli	Andaman Scops-owl	H	L	L	**L**
Otus beccarii	Biak Scops-owl	H	U	H	**L**
Otus brookii	Rajah Scops-owl	H	H	H	**H**
Otus brucei	Pallid Scops-owl	H	L	H	**L**
Otus capnodes	Anjouan Scops-owl	H	H	H	**H**
Otus collari	Sangihe Scops-owl	H	U	H	**L**
Otus elegans	Elegant Scops-owl	H	L	H	**L**
Otus enganensis	Enggano Scops-owl	H	U	H	**L**
Otus flammeolus	Flammulated Owl	H	L	L	**L**
Otus fuliginosus	Palawan Scops-owl	H	U	H	**L**
Otus hartlaubi	Sao Tome Scops-owl	H	H	L	**L**
Otus icterorhynchus	Sandy Scops-owl	H	U	L	**L**
Otus insularis	Seychelles Scops-owl	H	H	L	**L**
Otus ireneae	Sokoke Scops-owl	H	H	H	**H**
Otus leucotis	White-faced Scops-owl	U	L	L	**L**
Otus longicornis	Luzon Scops-owl	H	L	H	**L**
Otus madagascariensis	Torotoroka Scops-owl	U	U	U	**L**
Otus magicus	Moluccan Scops-owl	U	U	U	**L**
Otus manadensis	Sulawesi Scops-owl	H	U	U	**L**
Otus mantananensis	Mantanani Scops-owl	H	U	H	**L**
Otus mayottensis	Mayotte Scops-owl	H	U	H	**L**
Otus megalotis	Philippine Scops-owl	H	L	U	**L**
Otus mentawi	Mentawai Scops-owl	H	H	H	**H**
Otus mindorensis	Mindoro Scops-owl	H	U	H	**L**
Otus mirus	Mindanao Scops-owl	H	U	H	**L**
Otus moheliensis	Moheli Scops-owl	H	H	H	**H**
Otus pauliani	Grand Comoro Scops-owl	H	H	H	**H**
Otus pembaensis	Pemba Scops-owl	H	H	H	**H**
Otus podarginus	Palau Scops-owl	H	U	H	**L**
Otus rufescens	Reddish Scops-owl	H	U	H	**L**
Otus rutilus	Malagasy Scops-owl	U	L	U	**L**
Otus sagittatus	White-fronted Scops-owl	H	L	H	**L**
Otus scops	Common Scops-owl	H	L	H	**L**
Otus senegalensis	African Scops-owl	U	L	L	**L**
Otus siaoensis	Siau Scops-owl	H	H	H	**H**
Otus silvicola	Wallace's Scops-owl	H	U	H	**L**
Otus spilocephalus	Mountain Scops-owl	H	L	U	**L**

Otus sunia	Oriental Scops-owl	U	L	U	**L**
Otus thilohoffmanni	Serendib Scops-owl	H	H	H	**H**
Otus umbra	Simeulue Scops-owl	H	U	H	**L**
Oxylabes madagascariensis	White-throated Oxylabes	H	H	U	**L**
Oxypogon guerinii	Bearded Helmetcrest	H	H	H	**H**
Oxyruncus cristatus	Sharpbill	H	H	H	**H**
Oxyura australis	Blue-billed Duck	H	H	H	**H**
Oxyura jamaicensis	Ruddy Duck	H	H	L	**L**
Oxyura leucocephala	White-headed Duck	H	H	H	**H**
Oxyura maccoa	Maccoa Duck	H	H	L	**L**
Oxyura vittata	Lake Duck	H	H	L	**L**
Pachycare flavogriseum	Goldenface	U	H	U	**L**
Pachycephala albiventris	Green-backed Whistler	H	H	L	**L**
Pachycephala arctitorquis	Wallacean Whistler	U	H	U	**L**
Pachycephala aurea	Golden-backed Whistler	U	H	U	**L**
Pachycephala caledonica	New Caledonian Whistler	U	H	H	**L**
Pachycephala flavifrons	Samoan Whistler	H	H	U	**L**
Pachycephala griseonota	Drab Whistler	H	H	H	**H**
Pachycephala grisola	Mangrove Whistler	U	H	U	**L**
Pachycephala homeyeri	White-vented Whistler	H	H	U	**L**
Pachycephala hyperythra	Rusty Whistler	H	H	U	**L**
Pachycephala hypoxantha	Bornean Whistler	H	H	H	**H**
Pachycephala implicata	Hooded Whistler	H	H	H	**H**
Pachycephala inornata	Gilbert's Whistler	U	H	U	**L**
Pachycephala jacquinoti	Tongan Whistler	H	H	U	**L**
Pachycephala lanioides	White-breasted Whistler	H	H	H	**H**
Pachycephala leucogastra	White-bellied Whistler	H	H	L	**L**
Pachycephala lorentzi	Lorentz's Whistler	U	H	H	**L**
Pachycephala melanura	Black-tailed Whistler	U	H	U	**L**
Pachycephala meyeri	Vogelkop Whistler	H	H	H	**H**
Pachycephala modesta	Brown-backed Whistler	U	H	U	**L**
Pachycephala monacha	Black-headed Whistler	U	H	U	**L**
Pachycephala nudigula	Bare-throated Whistler	H	H	H	**H**
Pachycephala olivacea	Olive Whistler	U	H	U	**L**
Pachycephala orpheus	Fawn-breasted Whistler	U	H	H	**L**
Pachycephala pectoralis	Golden Whistler	U	H	L	**L**
Pachycephala phaionota	Island Whistler	H	H	H	**H**
Pachycephala philippinensis	Yellow-bellied Whistler	H	H	U	**L**
Pachycephala rufiventris	Rufous Whistler	U	H	U	**L**
Pachycephala rufogularis	Red-lored Whistler	H	H	L	**L**
Pachycephala schlegelii	Regent Whistler	H	H	U	**L**
Pachycephala simplex	Grey Whistler	U	H	L	**L**
Pachycephala soror	Sclater's Whistler	H	H	U	**L**

Pachycephala sulfuriventer	Sulphur-bellied Whistler	H	H	H	**H**
Pachycephalopsis hattamensis	Green-backed Robin	H	U	H	**L**
Pachycephalopsis poliosoma	White-eyed Robin	H	H	U	**L**
Pachycoccyx audeberti	Thick-billed Cuckoo	U	U	L	**L**
Pachyptila belcheri	Thin-billed Prion	H	H	H	**H**
Pachyptila crassirostris	Fulmar Prion	H	H	H	**H**
Pachyptila desolata	Antarctic Prion	H	H	L	**L**
Pachyptila salvini	Medium-billed Prion	H	H	H	**H**
Pachyptila turtur	Fairy Prion	H	H	H	**H**
Pachyptila vittata	Broad-billed Prion	H	H	L	**L**
Pachyramphus aglaiae	Rose-throated Becard	L	L	H	**L**
Pachyramphus albogriseus	Black-and-white Becard	H	H	L	**L**
Pachyramphus castaneus	Chestnut-crowned Becard	U	U	L	**L**
Pachyramphus cinnamomeus	Cinnamon Becard	H	L	H	**L**
Pachyramphus homochrous	One-coloured Becard	U	U	H	**L**
Pachyramphus major	Grey-collared Becard	L	U	H	**L**
Pachyramphus marginatus	Black-capped Becard	H	H	H	**H**
Pachyramphus minor	Pink-throated Becard	H	H	H	**H**
Pachyramphus niger	Jamaican Becard	H	L	H	**L**
Pachyramphus polychopterus	White-winged Becard	L	L	L	**L**
Pachyramphus rufus	Cinereous Becard	H	L	H	**L**
Pachyramphus spodiurus	Slaty Becard	H	U	H	**L**
Pachyramphus surinamus	Glossy-backed Becard	H	U	H	**L**
Pachyramphus validus	Crested Becard	H	L	L	**L**
Pachyramphus versicolor	Barred Becard	U	L	L	**L**
Pachyramphus viridis	Green-backed Becard	U	L	L	**L**
Padda fuscata	Timor Sparrow	H	L	U	**L**
Padda oryzivora	Java Sparrow	H	L	H	**L**
Pagodroma nivea	Snow Petrel	H	H	L	**L**
Pagophila eburnea	Ivory Gull	H	H	H	**H**
Palmeria dolei	Akohekohe	H	H	H	**H**
Pandion haliaetus	Osprey	L	H	H	**L**
Panterpe insignis	Fiery-throated Hummingbird	H	H	H	**H**
Panurus biarmicus	Bearded Parrotbill	H	L	H	**L**
Panyptila cayennensis	Lesser Swallow-tailed Swift	H	H	H	**H**
Panyptila sanctihieronymi	Great Swallow-tailed Swift	H	H	L	**L**
Papasula abbotti	Abbott's Booby	H	H	U	**L**
Parabuteo unicinctus	Harris's Hawk	L	H	L	**L**

Paradigalla brevicauda	Short-tailed Paradigalla	U	H	H	**L**
Paradigalla carunculata	Long-tailed Paradigalla	H	H	H	**H**
Paradisaea apoda	Greater Bird-of-paradise	H	H	H	**H**
Paradisaea decora	Goldie's Bird-of-paradise	H	H	U	**L**
Paradisaea guilielmi	Emperor Bird-of-paradise	H	H	H	**H**
Paradisaea minor	Lesser Bird-of-paradise	U	H	L	**L**
Paradisaea raggiana	Raggiana Bird-of-paradise	U	H	L	**L**
Paradisaea rubra	Red Bird-of-paradise	H	H	H	**H**
Paradisaea rudolphi	Blue Bird-of-paradise	H	H	H	**H**
Paradoxornis alphonsianus	Ashy-throated Parrotbill	U	L	U	**L**
Paradoxornis atrosuperciliaris	Black-browed Parrotbill	H	L	U	**L**
Paradoxornis brunneus	Brown-winged Parrotbill	U	L	H	**L**
Paradoxornis conspicillatus	Spectacled Parrotbill	U	U	U	**L**
Paradoxornis davidianus	Short-tailed Parrotbill	H	U	U	**L**
Paradoxornis flavirostris	Black-breasted Parrotbill	H	L	H	**L**
Paradoxornis fulvifrons	Fulvous Parrotbill	H	H	U	**L**
Paradoxornis gularis	Grey-headed Parrotbill	U	L	U	**L**
Paradoxornis guttaticollis	Spot-breasted Parrotbill	U	L	U	**L**
Paradoxornis heudei	Reed Parrotbill	U	L	H	**L**
Paradoxornis margaritae	Black-headed Parrotbill	H	U	L	**L**
Paradoxornis nipalensis	Black-throated Parrotbill	H	L	U	**L**
Paradoxornis paradoxus	Three-toed Parrotbill	H	U	H	**L**
Paradoxornis przewalskii	Rusty-throated Parrotbill	H	H	H	**H**
Paradoxornis ruficeps	Rufous-headed Parrotbill	H	L	U	**L**
Paradoxornis unicolor	Brown Parrotbill	H	U	U	**L**
Paradoxornis verreauxi	Golden Parrotbill	H	L	U	**L**
Paradoxornis webbianus	Vinous-throated Parrotbill	U	L	H	**L**
Paradoxornis zappeyi	Grey-hooded Parrotbill	H	H	L	**L**
Paramythia montium	Crested Berrypecker	U	H	H	**L**
Pardalotus punctatus	Spotted Pardalote	U	L	U	**L**
Pardalotus quadragintus	Forty-spotted Pardalote	H	L	L	**L**
Pardalotus rubricatus	Red-browed Pardalote	U	L	U	**L**
Pardalotus striatus	Striated Pardalote	H	L	L	**L**
Pardirallus maculatus	Spotted Rail	H	L	L	**L**
Pardirallus nigricans	Blackish Rail	H	L	L	**L**
Pardirallus sanguinolentus	Plumbeous Rail	H	L	U	**L**
Parkerthraustes humeralis	Yellow-shouldered Grosbeak	H	H	L	**L**
Parmoptila jamesoni	Jameson's Antpecker	U	U	U	**L**
Parmoptila rubrifrons	Red-fronted Antpecker	H	U	L	**L**
Parmoptila woodhousei	Woodhouse's Antpecker	H	L	U	**L**
Paroaria baeri	Crimson-fronted Cardinal	H	U	L	**L**

Paroaria capitata	Yellow-billed Cardinal	H	U	L	**L**
Paroaria coronata	Red-crested Cardinal	H	U	L	**L**
Paroaria dominicana	Red-cowled Cardinal	U	U	L	**L**
Paroaria gularis	Red-capped Cardinal	H	U	H	**L**
Parophasma galinieri	Abyssinian Catbird	H	H	L	**L**
Paroreomyza maculata	Oahu Alauahio	H	H	H	**H**
Paroreomyza montana	Maui Alauahio	H	H	H	**H**
Parotia carolae	Carola's Parotia	U	H	U	**L**
Parotia helenae	Eastern Parotia	U	H	H	**L**
Parotia lawesii	Lawes's Parotia	U	H	H	**L**
Parotia sefilata	Western Parotia	H	H	H	**H**
Parotia wahnesi	Wahnes's Parotia	H	H	H	**H**
Parula americana	Northern Parula	H	L	L	**L**
Parula gutturalis	Flame-throated Warbler	U	H	H	**L**
Parula pitiayumi	Tropical Parula	L	L	L	**L**
Parula superciliosa	Crescent-chested Warbler	H	H	L	**L**
Parus afer	Grey Tit	H	H	L	**L**
Parus albiventris	White-bellied Tit	H	L	L	**L**
Parus amabilis	Palawan Tit	H	U	H	**L**
Parus ater	Coal Tit	H	L	H	**L**
Parus atricapillus	Black-capped Chickadee	H	L	L	**L**
Parus bokharensis	Turkestan Tit	U	U	U	**L**
Parus caeruleus	Blue Tit	H	L	H	**L**
Parus carolinensis	Carolina Chickadee	H	L	L	**L**
Parus cinctus	Siberian Tit	H	L	H	**L**
Parus cinerascens	Ashy Tit	H	L	H	**L**
Parus cristatus	Crested Tit	H	L	H	**L**
Parus cyanus	Azure Tit	H	L	U	**L**
Parus davidi	Rusty-breasted Tit	H	U	L	**L**
Parus dichrous	Grey-crested Tit	H	L	U	**L**
Parus elegans	Elegant Tit	H	U	U	**L**
Parus fasciiventer	Stripe-breasted Tit	H	L	L	**L**
Parus flavipectus	Yellow-breasted Tit	H	L	U	**L**
Parus fringillinus	Red-throated Tit	H	L	U	**L**
Parus funereus	Dusky Tit	H	L	L	**L**
Parus gambeli	Mountain Chickadee	H	L	H	**L**
Parus griseiventris	Miombo Tit	H	L	L	**L**
Parus holsti	Yellow Tit	H	L	H	**L**
Parus hudsonicus	Boreal Chickadee	H	L	L	**L**
Parus leucomelas	White-winged Tit	H	L	L	**L**
Parus leuconotus	White-backed Tit	U	U	L	**L**
Parus lugubris	Sombre Tit	H	L	U	**L**
Parus major	Great Tit	H	L	H	**L**
Parus melanolophus	Black-crested Tit	H	L	U	**L**
Parus montanus	Willow Tit	H	L	U	**L**

Parus monticolus	Green-backed Tit	U	L	U	**L**
Parus niger	Black Tit	H	L	L	**L**
Parus nuchalis	White-naped Tit	H	U	H	**L**
Parus palustris	Marsh Tit	H	L	U	**L**
Parus rubidiventris	Rufous-vented Tit	U	L	U	**L**
Parus rufescens	Chestnut-backed Chickadee	H	L	H	**L**
Parus rufiventris	Rufous-bellied Tit	H	L	U	**L**
Parus rufonuchalis	Dark-grey Tit	H	L	U	**L**
Parus sclateri	Mexican Chickadee	H	L	L	**L**
Parus semilarvatus	White-fronted Tit	H	U	L	**L**
Parus spilonotus	Yellow-cheeked Tit	H	L	U	**L**
Parus superciliosus	White-browed Tit	U	U	U	**L**
Parus thruppi	Somali Tit	H	H	U	**L**
Parus varius	Varied Tit	H	L	H	**L**
Parus venustulus	Yellow-bellied Tit	H	L	U	**L**
Parus xanthogenys	Black-lored Tit	H	L	U	**L**
Passer ammodendri	Saxaul Sparrow	U	U	U	**L**
Passer castanopterus	Somali Sparrow	U	U	U	**L**
Passer diffusus	Southern Grey-headed Sparrow	U	U	U	**L**
Passer domesticus	House Sparrow	H	H	H	**H**
Passer eminibey	Chestnut Sparrow	U	U	U	**L**
Passer euchlorus	Arabian Golden Sparrow	U	U	U	**L**
Passer flaveolus	Plain-backed Sparrow	U	L	U	**L**
Passer gongonensis	Parrot-billed Sparrow	U	U	U	**L**
Passer griseus	Northern Grey-headed Sparrow	U	U	H	**L**
Passer hispaniolensis	Spanish Sparrow	H	L	H	**L**
Passer iagoensis	Iago Sparrow	H	U	H	**L**
Passer luteus	Sudan Golden Sparrow	U	L	U	**L**
Passer melanurus	Cape Sparrow	H	U	L	**L**
Passer moabiticus	Dead Sea Sparrow	H	L	H	**L**
Passer montanus	Eurasian Tree Sparrow	H	L	H	**L**
Passer motitensis	Southern Rufous-sparrow	U	U	U	**L**
Passer pyrrhonotus	Sind Sparrow	H	U	H	**L**
Passer rutilans	Russet Sparrow	U	L	U	**L**
Passer simplex	Desert Sparrow	H	L	H	**L**
Passer suahelicus	Swahili Sparrow	U	U	U	**L**
Passer swainsonii	Swainson's Sparrow	U	U	U	**L**
Passerculus sandwichensis	Savannah Sparrow	H	L	L	**L**
Passerella iliaca	Fox Sparrow	H	L	L	**L**
Passerina amoena	Lazuli Bunting	H	L	H	**L**
Passerina caerulea	Blue Grosbeak	L	L	H	**L**
Passerina ciris	Painted Bunting	L	L	H	**L**

Passerina cyanea	Indigo Bunting	H	L	L	**L**
Passerina leclancherii	Orange-breasted Bunting	L	U	H	**L**
Passerina rositae	Rose-bellied Bunting	H	H	L	**L**
Passerina versicolor	Varied Bunting	H	L	H	**L**
Patagioenas araucana	Chilean Pigeon	U	H	L	**L**
Patagioenas caribaea	Ring-tailed Pigeon	H	H	H	**H**
Patagioenas cayennensis	Pale-vented Pigeon	L	H	L	**L**
Patagioenas corensis	Bare-eyed Pigeon	U	H	H	**L**
Patagioenas fasciata	Band-tailed Pigeon	L	H	L	**L**
Patagioenas flavirostris	Red-billed Pigeon	L	H	H	**L**
Patagioenas goodsoni	Dusky Pigeon	H	H	H	**H**
Patagioenas inornata	Plain Pigeon	H	H	H	**H**
Patagioenas leucocephala	White-crowned Pigeon	H	H	H	**H**
Patagioenas maculosa	Spot-winged Pigeon	H	H	H	**H**
Patagioenas nigrirostris	Short-billed Pigeon	L	H	H	**L**
Patagioenas oenops	Peruvian Pigeon	H	H	H	**H**
Patagioenas picazuro	Picazuro Pigeon	U	H	L	**L**
Patagioenas plumbea	Plumbeous Pigeon	U	H	H	**L**
Patagioenas speciosa	Scaled Pigeon	L	H	H	**L**
Patagioenas squamosa	Scaly-naped Pigeon	U	H	H	**L**
Patagioenas subvinacea	Ruddy Pigeon	H	H	H	**H**
Patagona gigas	Giant Hummingbird	H	H	H	**H**
Pauxi pauxi	Helmeted Curassow	H	H	H	**H**
Pauxi unicornis	Horned Curassow	H	H	H	**H**
Pavo cristatus	Indian Peafowl	U	H	L	**L**
Pavo muticus	Green Peafowl	H	H	L	**L**
Pedionomus torquatus	Plains-wanderer	H	H	L	**L**
Pelagodroma marina	White-faced Storm-petrel	L	H	L	**L**
Pelargopsis amauroptera	Brown-winged Kingfisher	U	L	H	**L**
Pelargopsis capensis	Stork-billed Kingfisher	U	L	L	**L**
Pelargopsis melanorhyncha	Black-billed Kingfisher	U	U	U	**L**
Pelecanoides garnotii	Peruvian Diving-petrel	H	H	H	**H**
Pelecanoides georgicus	South Georgia Diving-petrel	H	H	H	**H**
Pelecanoides magellani	Magellanic Diving-petrel	H	H	L	**L**
Pelecanoides urinatrix	Common Diving-petrel	H	H	H	**H**
Pelecanus conspicillatus	Australian Pelican	U	H	U	**L**
Pelecanus crispus	Dalmatian Pelican	H	H	L	**L**
Pelecanus erythrorhynchos	American White Pelican	L	H	H	**L**
Pelecanus occidentalis	Brown Pelican	L	H	L	**L**
Pelecanus onocrotalus	Great White Pelican	H	H	L	**L**
Pelecanus philippensis	Spot-billed Pelican	H	H	L	**L**
Pelecanus rufescens	Pink-backed Pelican	L	H	L	**L**
Pelecanus thagus	Peruvian Pelican	H	H	H	**H**

Pellorneum albiventre	Spot-throated Babbler	U	L	U	**L**
Pellorneum capistratum	Black-capped Babbler	U	H	U	**L**
Pellorneum fuscocapillus	Brown-capped Babbler	U	H	H	**L**
Pellorneum palustre	Marsh Babbler	H	L	H	**L**
Pellorneum ruficeps	Puff-throated Babbler	U	L	U	**L**
Peltops blainvillii	Lowland Peltops	H	H	U	**L**
Peltops montanus	Mountain Peltops	H	H	U	**L**
Penelope albipennis	White-winged Guan	H	U	H	**L**
Penelope argyrotis	Band-tailed Guan	U	U	H	**L**
Penelope barbata	Bearded Guan	H	U	H	**L**
Penelope dabbenei	Red-faced Guan	H	L	H	**L**
Penelope jacquacu	Spix's Guan	H	H	H	**H**
Penelope jacucaca	White-browed Guan	H	U	L	**L**
Penelope marail	Marail Guan	H	L	H	**L**
Penelope montagnii	Andean Guan	H	H	L	**L**
Penelope obscura	Dusky-legged Guan	H	L	L	**L**
Penelope ochrogaster	Chestnut-bellied Guan	H	H	L	**L**
Penelope ortoni	Baudo Guan	H	U	H	**L**
Penelope perspicax	Cauca Guan	H	H	H	**H**
Penelope pileata	White-crested Guan	H	L	H	**L**
Penelope purpurascens	Crested Guan	H	L	L	**L**
Penelope superciliaris	Rusty-margined Guan	U	L	L	**L**
Penelopides affinis	Mindanao Hornbill	H	H	H	**H**
Penelopides exarhatus	Sulawesi Hornbill	H	H	H	**H**
Penelopides manillae	Luzon Hornbill	H	H	H	**H**
Penelopides mindorensis	Mindoro Hornbill	H	H	H	**H**
Penelopides panini	Visayan Hornbill	H	H	H	**H**
Penelopides samarensis	Samar Hornbill	H	H	L	**L**
Penelopina nigra	Highland Guan	H	H	H	**H**
Peneothello bimaculata	White-rumped Robin	H	U	U	**L**
Peneothello cryptoleuca	Smoky Robin	H	U	H	**L**
Peneothello cyanus	Blue-grey Robin	U	H	U	**L**
Peneothello sigillatus	White-winged Robin	U	H	U	**L**
Percnostola arenarum	Allpahuayo Antbird	H	H	H	**H**
Percnostola lophotes	White-lined Antbird	H	H	H	**H**
Percnostola rufifrons	Black-headed Antbird	H	H	H	**H**
Perdicula argoondah	Rock Bush-quail	U	L	U	**L**
Perdicula asiatica	Jungle Bush-quail	U	L	L	**L**
Perdicula erythrorhyncha	Painted Bush-quail	U	L	U	**L**
Perdicula manipurensis	Manipur Bush-quail	H	L	H	**L**
Perdix dauurica	Daurian Partridge	U	L	U	**L**
Perdix hodgsoniae	Tibetan Partridge	U	L	U	**L**
Perdix perdix	Grey Partridge	H	L	H	**L**
Pericrocotus brevirostris	Short-billed Minivet	U	U	H	**L**
Pericrocotus cantonensis	Brown-rumped Minivet	U	U	H	**L**

Pericrocotus cinnamomeus	Small Minivet	U	L	L	**L**
Pericrocotus divaricatus	Ashy Minivet	U	U	H	**L**
Pericrocotus erythropygius	White-bellied Minivet	U	L	U	**L**
Pericrocotus ethologus	Long-tailed Minivet	U	L	H	**L**
Pericrocotus flammeus	Scarlet Minivet	U	L	L	**L**
Pericrocotus igneus	Fiery Minivet	H	H	H	**H**
Pericrocotus lansbergei	Flores Minivet	H	H	H	**H**
Pericrocotus miniatus	Sunda Minivet	U	U	H	**L**
Pericrocotus roseus	Rosy Minivet	U	L	H	**L**
Pericrocotus solaris	Grey-chinned Minivet	U	L	L	**L**
Pericrocotus tegimae	Ryukyu Minivet	U	U	L	**L**
Periporphyrus erythromelas	Red-and-black Grosbeak	H	H	H	**H**
Perisoreus canadensis	Grey Jay	H	H	L	**L**
Perisoreus infaustus	Siberian Jay	H	H	H	**H**
Perisoreus internigrans	Sichuan Jay	H	H	H	**H**
Perissocephalus tricolor	Capuchinbird	H	H	H	**H**
Pernis apivorus	European Honey-buzzard	H	H	H	**H**
Pernis celebensis	Barred Honey-buzzard	H	H	L	**L**
Pernis ptilorhyncus	Oriental Honey-buzzard	L	H	H	**L**
Petrochelidon fulva	Cave Swallow	H	L	H	**L**
Petrochelidon pyrrhonota	Cliff Swallow	L	L	L	**L**
Petrochelidon rufocollaris	Chestnut-collared Swallow	U	U	H	**L**
Petroica archboldi	Snow Mountain Robin	H	H	H	**H**
Petroica australis	New Zealand Robin	U	H	H	**L**
Petroica bivittata	Alpine Robin	H	H	H	**H**
Petroica goodenovii	Red-capped Robin	U	H	U	**L**
Petroica macrocephala	Tomtit	U	H	U	**L**
Petroica multicolor	Scarlet Robin	U	H	U	**L**
Petroica phoenicea	Flame Robin	H	H	L	**L**
Petroica rodinogaster	Pink Robin	H	H	U	**L**
Petroica rosea	Rose Robin	U	H	U	**L**
Petroica traversi	Black Robin	H	H	U	**L**
Petronia brachydactyla	Pale Rock Sparrow	U	L	U	**L**
Petronia dentata	Bush Petronia	U	U	U	**L**
Petronia petronia	Rock Sparrow	H	L	H	**L**
Petronia pyrgita	Yellow-spotted Petronia	H	U	L	**L**
Petronia superciliaris	Yellow-throated Petronia	U	U	L	**L**
Petronia xanthocollis	Chestnut-shouldered Petronia	U	L	H	**L**
Petrophassa albipennis	White-quilled Rock-pigeon	U	H	H	**L**
Petrophassa rufipennis	Chestnut-quilled Rock-pigeon	U	H	L	**L**
Peucedramus taeniatus	Olive Warbler	H	L	L	**L**

Pezopetes capitalis	Large-footed Finch	U	H	H	**L**
Pezoporus occidentalis	Night Parrot	H	H	L	**L**
Pezoporus wallicus	Ground Parrot	U	H	U	**L**
Phacellodomus dorsalis	Chestnut-backed Thornbird	H	U	H	**L**
Phacellodomus erythrophthalmus	Red-eyed Thornbird	H	H	U	**L**
Phacellodomus ferrugineigula	Orange-eyed Thornbird	H	U	L	**L**
Phacellodomus maculipectus	Spot-breasted Thornbird	H	U	H	**L**
Phacellodomus ruber	Greater Thornbird	U	L	L	**L**
Phacellodomus rufifrons	Rufous-fronted Thornbird	U	L	L	**L**
Phacellodomus sibilatrix	Little Thornbird	H	L	L	**L**
Phacellodomus striaticeps	Streak-fronted Thornbird	H	L	H	**L**
Phacellodomus striaticollis	Freckle-breasted Thornbird	H	L	H	**L**
Phaenicophaeus calyorhynchus	Yellow-billed Malkoha	U	U	H	**L**
Phaenicophaeus chlorophaeus	Raffles's Malkoha	H	L	H	**L**
Phaenicophaeus cumingi	Scale-feathered Malkoha	H	U	L	**L**
Phaenicophaeus curvirostris	Chestnut-breasted Malkoha	H	L	H	**L**
Phaenicophaeus diardi	Black-bellied Malkoha	H	U	H	**L**
Phaenicophaeus javanicus	Red-billed Malkoha	H	U	H	**L**
Phaenicophaeus leschenaultii	Sirkeer Malkoha	U	L	L	**L**
Phaenicophaeus pyrrhocephalus	Red-faced Malkoha	H	L	H	**L**
Phaenicophaeus sumatranus	Chestnut-bellied Malkoha	H	H	H	**H**
Phaenicophaeus superciliosus	Red-crested Malkoha	H	U	L	**L**
Phaenicophaeus tristis	Green-billed Malkoha	U	L	H	**L**
Phaenicophaeus viridirostris	Blue-faced Malkoha	U	H	L	**L**
Phaenicophilus palmarum	Black-crowned Palm-tanager	U	L	H	**L**
Phaenicophilus poliocephalus	Grey-crowned Palm-tanager	U	L	H	**L**
Phaenostictus mcleannani	Ocellated Antbird	H	H	H	**H**
Phaeochroa cuvierii	Scaly-breasted Hummingbird	L	H	H	**L**
Phaeomyias murina	Mouse-coloured Tyrannulet	U	H	L	**L**
Phaeothlypis fulvicauda	Buff-rumped Warbler	H	H	L	**L**
Phaeothlypis rivularis	Neotropical River Warbler	H	U	H	**L**
Phaethon aethereus	Red-billed Tropicbird	H	H	L	**L**
Phaethon lepturus	White-tailed Tropicbird	L	H	L	**L**
Phaethon rubricauda	Red-tailed Tropicbird	L	H	L	**L**

Phaethornis anthophilus	Pale-bellied Hermit	U	H	L	**L**
Phaethornis atrimentalis	Black-throated Hermit	H	U	H	**L**
Phaethornis augusti	Sooty-capped Hermit	U	H	H	**L**
Phaethornis bourcieri	Straight-billed Hermit	H	H	H	**H**
Phaethornis eurynome	Scale-throated Hermit	H	H	L	**L**
Phaethornis griseogularis	Grey-chinned Hermit	H	U	L	**L**
Phaethornis guy	Green Hermit	U	H	L	**L**
Phaethornis hispidus	White-bearded Hermit	H	H	L	**L**
Phaethornis idaliae	Minute Hermit	H	H	H	**H**
Phaethornis koepckeae	Koepcke's Hermit	H	H	H	**H**
Phaethornis longirostris	Long-billed Hermit	H	H	H	**H**
Phaethornis longuemareus	Little Hermit	H	H	H	**H**
Phaethornis malaris	Great-billed Hermit	H	H	H	**H**
Phaethornis nattereri	Cinnamon-throated Hermit	H	U	L	**L**
Phaethornis philippii	Needle-billed Hermit	H	U	L	**L**
Phaethornis pretrei	Planalto Hermit	U	H	L	**L**
Phaethornis ruber	Reddish Hermit	U	H	H	**L**
Phaethornis rupurumii	Streak-throated Hermit	H	U	H	**L**
Phaethornis squalidus	Dusky-throated Hermit	H	H	L	**L**
Phaethornis striigularis	Stripe-throated Hermit	L	H	H	**L**
Phaethornis stuarti	White-browed Hermit	H	U	H	**L**
Phaethornis subochraceus	Buff-bellied Hermit	H	U	H	**L**
Phaethornis superciliosus	Long-tailed Hermit	H	H	H	**H**
Phaethornis syrmatophorus	Tawny-bellied Hermit	H	H	H	**H**
Phaethornis yaruqui	White-whiskered Hermit	H	H	H	**H**
Phaetusa simplex	Large-billed Tern	L	H	H	**L**
Phainopepla nitens	Phainopepla	H	L	H	**L**
Phainoptila melanoxantha	Black-and-yellow Silky-flycatcher	U	H	H	**L**
Phalacrocorax africanus	Reed Cormorant	L	H	L	**L**
Phalacrocorax aristotelis	European Shag	H	H	H	**H**
Phalacrocorax atriceps	Imperial Shag	L	H	H	**L**
Phalacrocorax auritus	Double-crested Cormorant	L	H	H	**L**
Phalacrocorax bougainvillii	Guanay Cormorant	L	H	H	**L**
Phalacrocorax brasilianus	Neotropic Cormorant	L	H	L	**L**
Phalacrocorax campbelli	Campbell Island Shag	H	H	H	**H**
Phalacrocorax capensis	Cape Cormorant	H	H	H	**H**
Phalacrocorax capillatus	Japanese Cormorant	L	H	H	**L**
Phalacrocorax carbo	Great Cormorant	L	H	H	**L**
Phalacrocorax carunculatus	New Zealand King Shag	H	H	H	**H**
Phalacrocorax chalconotus	Stewart Island Shag	H	H	H	**H**

Phalacrocorax colensoi	Auckland Islands Shag	H	H	H	**H**
Phalacrocorax coronatus	Crowned Cormorant	H	H	H	**H**
Phalacrocorax featherstoni	Pitt Island Shag	H	H	H	**H**
Phalacrocorax fuscescens	Black-faced Cormorant	H	H	H	**H**
Phalacrocorax fuscicollis	Indian Cormorant	U	H	U	**L**
Phalacrocorax gaimardi	Red-legged Cormorant	H	H	H	**H**
Phalacrocorax harrisi	Flightless Cormorant	H	H	H	**H**
Phalacrocorax magellanicus	Rock Shag	H	H	H	**H**
Phalacrocorax melanoleucos	Little Pied Cormorant	U	H	U	**L**
Phalacrocorax neglectus	Bank Cormorant	H	H	H	**H**
Phalacrocorax niger	Little Cormorant	U	H	U	**L**
Phalacrocorax nigrogularis	Socotra Cormorant	H	H	H	**H**
Phalacrocorax onslowi	Chatham Islands Shag	H	H	H	**H**
Phalacrocorax pelagicus	Pelagic Cormorant	H	H	H	**H**
Phalacrocorax penicillatus	Brandt's Cormorant	L	H	H	**L**
Phalacrocorax punctatus	Spotted Shag	L	H	H	**L**
Phalacrocorax pygmeus	Pygmy Cormorant	H	H	L	**L**
Phalacrocorax ranfurlyi	Bounty Islands Shag	H	H	H	**H**
Phalacrocorax sulcirostris	Little Black Cormorant	U	H	U	**L**
Phalacrocorax urile	Red-faced Cormorant	H	H	H	**H**
Phalacrocorax varius	Large Pied Cormorant	L	H	L	**L**
Phalaenoptilus nuttallii	Common Poorwill	H	H	H	**H**
Phalaropus fulicarius	Red Phalarope	L	H	L	**L**
Phalaropus lobatus	Red-necked Phalarope	H	H	L	**L**
Phalcoboenus albogularis	White-throated Caracara	H	H	H	**H**
Phalcoboenus australis	Striated Caracara	H	H	H	**H**
Phalcoboenus carunculatus	Carunculated Caracara	H	H	H	**H**
Phalcoboenus megalopterus	Mountain Caracara	H	H	H	**H**
Phapitreron amethystinus	Amethyst Brown-dove	H	H	L	**L**
Phapitreron brunneiceps	Mindanao Brown-dove	H	H	H	**H**
Phapitreron cinereiceps	Tawitawi Brown-dove	H	H	H	**H**
Phapitreron leucotis	White-eared Brown-dove	H	H	L	**L**
Phaps chalcoptera	Common Bronzewing	U	H	L	**L**
Phaps elegans	Brush Bronzewing	H	H	H	**H**
Phaps histrionica	Flock Bronzewing	U	H	U	**L**
Pharomachrus antisianus	Crested Quetzal	H	H	L	**L**
Pharomachrus auriceps	Golden-headed Quetzal	H	H	L	**L**
Pharomachrus fulgidus	White-tipped Quetzal	H	H	H	**H**
Pharomachrus mocinno	Resplendent Quetzal	H	H	H	**H**
Pharomachrus pavoninus	Pavonine Quetzal	H	H	H	**H**

Phasianus colchicus	Common Pheasant	H	L	H	**L**
Phedina borbonica	Mascarene Martin	U	L	U	**L**
Phedina brazzae	Brazza's Martin	H	L	H	**L**
Phegornis mitchellii	Diademed Plover	H	H	H	**H**
Phelpsia inornatus	White-bearded Flycatcher	H	H	H	**H**
Pheucticus aureoventris	Black-backed Grosbeak	U	U	H	**L**
Pheucticus chrysogaster	Golden-bellied Grosbeak	U	U	H	**L**
Pheucticus chrysopeplus	Yellow Grosbeak	L	U	H	**L**
Pheucticus ludovicianus	Rose-breasted Grosbeak	L	L	L	**L**
Pheucticus melanocephalus	Black-headed Grosbeak	H	L	H	**L**
Pheucticus tibialis	Black-thighed Grosbeak	U	H	H	**L**
Phibalura flavirostris	Swallow-tailed Cotinga	H	L	L	**L**
Phigys solitarius	Collared Lory	H	H	L	**L**
Philemon albitorques	White-naped Friarbird	H	U	H	**L**
Philemon argenticeps	Silver-crowned Friarbird	U	L	L	**L**
Philemon brassi	Brass's Friarbird	H	U	H	**L**
Philemon buceroides	Helmeted Friarbird	U	L	L	**L**
Philemon citreogularis	Little Friarbird	U	L	U	**L**
Philemon cockerelli	New Britain Friarbird	H	U	H	**L**
Philemon corniculatus	Noisy Friarbird	H	L	L	**L**
Philemon diemenensis	New Caledonian Friarbird	U	U	H	**L**
Philemon eichhorni	New Ireland Friarbird	H	U	H	**L**
Philemon fuscicapillus	Dusky Friarbird	H	U	H	**L**
Philemon inornatus	Plain Friarbird	U	U	H	**L**
Philemon meyeri	Meyer's Friarbird	U	U	H	**L**
Philemon moluccensis	Black-faced Friarbird	H	U	H	**L**
Philemon novaeguineae	New Guinea Friarbird	U	U	U	**L**
Philemon subcorniculatus	Grey-necked Friarbird	H	U	H	**L**
Philentoma pyrhoptera	Rufous-winged Philentoma	H	H	U	**L**
Philentoma velata	Maroon-breasted Philentoma	H	H	H	**H**
Philepitta castanea	Velvet Asity	H	L	H	**L**
Philepitta schlegeli	Schlegel's Asity	H	U	L	**L**
Philesturnus carunculatus	Saddleback	H	H	H	**H**
Philetairus socius	Sociable Weaver	H	U	H	**L**
Philomachus pugnax	Ruff	L	H	H	**L**
Philortyx fasciatus	Banded Quail	L	L	H	**L**
Philydor atricapillus	Black-capped Foliage-gleaner	H	H	L	**L**
Philydor erythrocercum	Rufous-rumped Foliage-gleaner	H	H	H	**H**
Philydor erythropterum	Chestnut-winged Foliage-gleaner	H	H	H	**H**
Philydor fuscipenne	Slaty-winged Foliage-gleaner	H	H	L	**L**
Philydor lichtensteini	Ochre-breasted Foliage-gleaner	H	H	L	**L**

Philydor novaesi	Alagoas Foliage-gleaner	H	H	H	**H**
Philydor pyrrhodes	Cinnamon-rumped Foliage-gleaner	H	H	H	**H**
Philydor ruficaudatum	Rufous-tailed Foliage-gleaner	H	H	H	**H**
Philydor rufum	Buff-fronted Foliage-gleaner	H	H	L	**L**
Phimosus infuscatus	Bare-faced Ibis	H	H	L	**L**
Phlegopsis erythroptera	Reddish-winged Bare-eye	H	H	H	**H**
Phlegopsis nigromaculata	Black-spotted Bare-eye	H	H	H	**H**
Phleocryptes melanops	Wren-like Rushbird	H	L	L	**L**
Phlogophilus harterti	Peruvian Piedtail	H	U	H	**L**
Phlogophilus hemileucurus	Ecuadorian Piedtail	H	H	H	**H**
Phodilus badius	Oriental Bay-owl	H	L	L	**L**
Phodilus prigoginei	Congo Bay-owl	H	H	H	**H**
Phoebastria albatrus	Short-tailed Albatross	H	H	L	**L**
Phoebastria immutabilis	Laysan Albatross	L	H	L	**L**
Phoebastria irrorata	Waved Albatross	L	H	H	**L**
Phoebastria nigripes	Black-footed Albatross	L	H	L	**L**
Phoebetria fusca	Sooty Albatross	H	H	H	**H**
Phoebetria palpebrata	Light-mantled Albatross	H	H	L	**L**
Phoenicircus carnifex	Guianan Red-cotinga	H	U	H	**L**
Phoenicircus nigricollis	Black-necked Red-cotinga	H	U	H	**L**
Phoeniconaias minor	Lesser Flamingo	L	H	L	**L**
Phoenicoparrus andinus	Andean Flamingo	H	H	H	**H**
Phoenicoparrus jamesi	Puna Flamingo	H	H	H	**H**
Phoenicopterus chilensis	Chilean Flamingo	H	H	L	**L**
Phoenicopterus roseus	Greater Flamingo	L	H	L	**L**
Phoenicopterus ruber	Caribbean Flamingo	H	H	H	**H**
Phoeniculus bollei	White-headed Woodhoopoe	H	H	H	**H**
Phoeniculus castaneiceps	Forest Woodhoopoe	H	H	L	**L**
Phoeniculus damarensis	Violet Woodhoopoe	H	H	U	**L**
Phoeniculus purpureus	Green Woodhoopoe	H	H	L	**L**
Phoeniculus somaliensis	Black-billed Woodhoopoe	H	H	L	**L**
Phoenicurus alaschanicus	Ala Shan Redstart	H	U	H	**L**
Phoenicurus auroreus	Daurian Redstart	U	L	U	**L**
Phoenicurus caeruleocephala	Blue-capped Redstart	U	U	U	**L**
Phoenicurus erythrogastrus	White-winged Redstart	H	L	L	**L**
Phoenicurus erythronotus	Rufous-backed Redstart	U	U	U	**L**
Phoenicurus frontalis	Blue-fronted Redstart	U	L	U	**L**
Phoenicurus hodgsoni	Hodgson's Redstart	U	U	U	**L**
Phoenicurus moussieri	Moussier's Redstart	H	L	H	**L**
Phoenicurus ochruros	Black Redstart	H	L	H	**L**
Phoenicurus phoenicurus	Common Redstart	H	L	H	**L**

Phoenicurus schisticeps	White-throated Redstart	U	L	U	**L**
Pholidornis rushiae	Tit-hylia	H	H	L	**L**
Phragmacia substriata	Namaqua Warbler	H	H	H	**H**
Phrygilus alaudinus	Band-tailed Sierra-finch	H	U	H	**L**
Phrygilus atriceps	Black-hooded Sierra-finch	H	U	H	**L**
Phrygilus carbonarius	Carbonated Sierra-finch	H	U	H	**L**
Phrygilus dorsalis	Red-backed Sierra-finch	H	U	H	**L**
Phrygilus erythronotus	White-throated Sierra-finch	H	U	H	**L**
Phrygilus fruticeti	Mourning Sierra-finch	H	U	H	**L**
Phrygilus gayi	Grey-hooded Sierra-finch	H	U	L	**L**
Phrygilus patagonicus	Patagonian Sierra-finch	U	U	H	**L**
Phrygilus plebejus	Ash-breasted Sierra-finch	H	U	H	**L**
Phrygilus punensis	Peruvian Sierra-finch	U	U	H	**L**
Phrygilus unicolor	Plumbeous Sierra-finch	H	U	H	**L**
Phylidonyris albifrons	White-fronted Honeyeater	H	H	L	**L**
Phylidonyris melanops	Tawny-crowned Honeyeater	U	H	U	**L**
Phylidonyris niger	White-cheeked Honeyeater	U	H	U	**L**
Phylidonyris notabilis	New Hebrides Honeyeater	H	H	L	**L**
Phylidonyris novaehollandiae	New Holland Honeyeater	H	H	L	**L**
Phylidonyris pyrrhopterus	Crescent Honeyeater	H	H	H	**H**
Phylidonyris undulatus	Barred Honeyeater	U	H	H	**L**
Phyllanthus atripennis	Capuchin Babbler	H	H	L	**L**
Phyllastrephus albigularis	White-throated Greenbul	H	H	L	**L**
Phyllastrephus baumanni	Baumann's Greenbul	H	U	L	**L**
Phyllastrephus cabanisi	Cabanis's Greenbul	U	H	U	**L**
Phyllastrephus cerviniventris	Grey-olive Greenbul	U	H	L	**L**
Phyllastrephus debilis	Tiny Greenbul	U	H	L	**L**
Phyllastrephus fischeri	Fischer's Greenbul	U	U	U	**L**
Phyllastrephus flavostriatus	Yellow-streaked Greenbul	U	H	L	**L**
Phyllastrephus fulviventris	Pale-olive Greenbul	U	U	L	**L**
Phyllastrephus hypochloris	Toro Olive Greenbul	H	U	H	**L**
Phyllastrephus icterinus	Icterine Greenbul	H	H	L	**L**
Phyllastrephus leucolepis	Liberian Greenbul	H	H	L	**L**
Phyllastrephus poensis	Cameroon Olive Greenbul	H	H	L	**L**
Phyllastrephus poliocephalus	Grey-headed Greenbul	H	H	H	**H**
Phyllastrephus strepitans	Northern Brownbul	H	U	L	**L**
Phyllastrephus terrestris	Terrestrial Brownbul	U	H	L	**L**
Phyllastrephus xavieri	Xavier's Greenbul	H	H	U	**L**
Phyllolais pulchella	Buff-bellied Warbler	U	L	L	**L**
Phyllomyias burmeisteri	Rough-legged Tyrannulet	H	U	L	**L**
Phyllomyias cinereiceps	Ashy-headed Tyrannulet	H	H	L	**L**

Phyllomyias fasciatus	Planalto Tyrannulet	H	H	L	**L**
Phyllomyias griseiceps	Sooty-headed Tyrannulet	U	U	L	**L**
Phyllomyias griseocapilla	Grey-capped Tyrannulet	U	U	L	**L**
Phyllomyias nigrocapillus	Black-capped Tyrannulet	U	U	H	**L**
Phyllomyias plumbeiceps	Plumbeous-crowned Tyrannulet	H	H	L	**L**
Phyllomyias reiseri	Reiser's Tyrannulet	H	H	L	**L**
Phyllomyias sclateri	Sclater's Tyrannulet	U	U	H	**L**
Phyllomyias urichi	Urich's Tyrannulet	H	H	H	**H**
Phyllomyias uropygialis	Tawny-rumped Tyrannulet	U	U	H	**L**
Phyllomyias virescens	Greenish Tyrannulet	H	H	L	**L**
Phylloscartes beckeri	Bahia Tyrannulet	H	H	H	**H**
Phylloscartes ceciliae	Alagoas Tyrannulet	H	H	H	**H**
Phylloscartes chapmani	Chapman's Tyrannulet	H	H	H	**H**
Phylloscartes difficilis	Serra Do Mar Tyrannulet	H	H	H	**H**
Phylloscartes eximius	Southern Bristle-tyrant	H	H	L	**L**
Phylloscartes flaviventris	Rufous-lored Tyrannulet	H	H	H	**H**
Phylloscartes flavovirens	Yellow-green Tyrannulet	H	H	L	**L**
Phylloscartes gualaquizae	Ecuadorian Tyrannulet	H	H	H	**H**
Phylloscartes kronei	Restinga Tyrannulet	H	U	H	**L**
Phylloscartes lanyoni	Antioquia Bristle-tyrant	H	H	H	**H**
Phylloscartes nigrifrons	Black-fronted Tyrannulet	H	H	H	**H**
Phylloscartes ophthalmicus	Marble-faced Bristle-tyrant	H	H	L	**L**
Phylloscartes orbitalis	Spectacled Bristle-tyrant	H	H	H	**H**
Phylloscartes oustaleti	Oustalet's Tyrannulet	H	H	H	**H**
Phylloscartes parkeri	Cinnamon-faced Tyrannulet	H	H	H	**H**
Phylloscartes paulista	Sao Paulo Tyrannulet	H	H	L	**L**
Phylloscartes poecilotis	Variegated Bristle-tyrant	H	H	L	**L**
Phylloscartes roquettei	Minas Gerais Tyrannulet	H	H	L	**L**
Phylloscartes superciliaris	Rufous-browed Tyrannulet	H	H	H	**H**
Phylloscartes sylviolus	Bay-ringed Tyrannulet	H	H	L	**L**
Phylloscartes venezuelanus	Venezuelan Bristle-tyrant	H	H	H	**H**
Phylloscartes ventralis	Mottle-cheeked Tyrannulet	H	H	H	**H**
Phylloscartes virescens	Olive-green Tyrannulet	H	H	H	**H**
Phylloscopus affinis	Tickell's Leaf-warbler	U	U	U	**L**
Phylloscopus amoenus	Sombre Leaf-warbler	H	H	H	**H**
Phylloscopus armandii	Yellow-streaked Warbler	U	U	U	**L**
Phylloscopus bonelli	Bonelli's Warbler	H	L	L	**L**
Phylloscopus borealis	Arctic Warbler	H	H	H	**H**
Phylloscopus borealoides	Sakhalin Leaf-warbler	H	U	U	**L**
Phylloscopus budongoensis	Uganda Woodland-warbler	H	H	L	**L**
Phylloscopus canariensis	Canary Islands Chiffchaff	H	L	H	**L**

Phylloscopus cantator	Yellow-vented Warbler	U	L	H	**L**
Phylloscopus cebuensis	Lemon-throated Leaf-warbler	H	H	U	**L**
Phylloscopus chloronotus	Pale-rumped Warbler	U	L	U	**L**
Phylloscopus claudiae	Claudia's Warbler	U	U	U	**L**
Phylloscopus collybita	Common Chiffchaff	H	L	H	**L**
Phylloscopus coronatus	Eastern Crowned Warbler	U	U	U	**L**
Phylloscopus davisoni	Davison's Leaf-warbler	U	U	U	**L**
Phylloscopus emeiensis	Emei Leaf-warbler	H	U	H	**L**
Phylloscopus forresti	Sichuan Leaf-warbler	U	U	U	**L**
Phylloscopus fuligiventer	Smoky Warbler	U	U	U	**L**
Phylloscopus fuscatus	Dusky Warbler	U	U	U	**L**
Phylloscopus goodsoni	Hartert's Warbler	U	U	U	**L**
Phylloscopus griseolus	Sulphur-bellied Warbler	U	U	U	**L**
Phylloscopus hainanus	Hainan Leaf-warbler	H	H	L	**L**
Phylloscopus herberti	Black-capped Woodland-warbler	H	H	L	**L**
Phylloscopus humei	Hume's Leaf-warbler	U	U	U	**L**
Phylloscopus ibericus	Iberian Chiffchaff	L	L	L	**L**
Phylloscopus ijimae	Izu Leaf-warbler	H	U	H	**L**
Phylloscopus inornatus	Inornate Warbler	U	L	U	**L**
Phylloscopus kansuensis	Gansu Leaf-warbler	H	U	U	**L**
Phylloscopus laetus	Red-faced Woodland-warbler	H	H	L	**L**
Phylloscopus laurae	Laura's Woodland-warbler	H	U	H	**L**
Phylloscopus maculipennis	Ashy-throated Warbler	U	U	U	**L**
Phylloscopus magnirostris	Large-billed Leaf-warbler	U	L	U	**L**
Phylloscopus makirensis	Makira Leaf-warbler	H	H	L	**L**
Phylloscopus neglectus	Plain Leaf-warbler	H	U	L	**L**
Phylloscopus occipitalis	Western Crowned Warbler	U	U	U	**L**
Phylloscopus ogilviegranti	Kloss's Leaf-warbler	U	U	U	**L**
Phylloscopus olivaceus	Philippine Leaf-warbler	H	H	U	**L**
Phylloscopus poliocephalus	Island Leaf-warbler	U	U	U	**L**
Phylloscopus presbytes	Timor Leaf-warbler	U	U	U	**L**
Phylloscopus proregulus	Lemon-rumped Warbler	U	U	U	**L**
Phylloscopus pulcher	Buff-barred Warbler	U	L	U	**L**
Phylloscopus reguloides	Southern Blyth's Leaf-warbler	U	L	U	**L**
Phylloscopus ricketti	Sulphur-breasted Warbler	U	U	U	**L**
Phylloscopus ruficapilla	Yellow-throated Woodland-warbler	H	H	L	**L**
Phylloscopus sarasinorum	Sulawesi Leaf-warbler	H	H	H	**H**
Phylloscopus schwarzi	Radde's Warbler	U	U	U	**L**
Phylloscopus sibilatrix	Wood Warbler	H	L	H	**L**

Phylloscopus sindianus	Mountain Chiffchaff	H	L	H	**L**
Phylloscopus subaffinis	Buff-throated Warbler	U	L	U	**L**
Phylloscopus subviridis	Brooks's Leaf-warbler	U	U	H	**L**
Phylloscopus tenellipes	Pale-legged Leaf-warbler	U	U	U	**L**
Phylloscopus trivirgatus	Mountain Leaf-warbler	H	H	U	**L**
Phylloscopus trochiloides	Greenish Warbler	H	L	H	**L**
Phylloscopus trochilus	Willow Warbler	H	L	H	**L**
Phylloscopus tytleri	Tytler's Leaf-warbler	H	U	H	**L**
Phylloscopus umbrovirens	Brown Woodland-warbler	U	L	L	**L**
Phylloscopus xanthoschistos	Grey-hooded Warbler	H	L	U	**L**
Phylloscopus yunnanensis	Chinese Leaf-warbler	U	U	U	**L**
Phytotoma raimondii	Peruvian Plantcutter	H	L	H	**L**
Phytotoma rara	Rufous-tailed Plantcutter	U	L	L	**L**
Phytotoma rutila	White-tipped Plantcutter	H	L	L	**L**
Piaya cayana	Squirrel Cuckoo	L	L	L	**L**
Piaya melanogaster	Black-bellied Cuckoo	H	U	H	**L**
Pica nuttalli	Yellow-billed Magpie	H	H	L	**L**
Pica pica	Black-billed Magpie	H	H	H	**H**
Picathartes gymnocephalus	White-necked Picathartes	H	H	L	**L**
Picathartes oreas	Grey-necked Picathartes	H	H	H	**H**
Picoides albolarvatus	White-headed Woodpecker	H	L	L	**L**
Picoides arcticus	Black-backed Woodpecker	H	L	L	**L**
Picoides arizonae	Arizona Woodpecker	H	L	H	**L**
Picoides borealis	Red-cockaded Woodpecker	H	H	L	**L**
Picoides dorsalis	American Three-toed Woodpecker	H	U	L	**L**
Picoides fumigatus	Smoky-brown Woodpecker	H	L	L	**L**
Picoides nuttallii	Nuttall's Woodpecker	H	L	L	**L**
Picoides pubescens	Downy Woodpecker	H	L	L	**L**
Picoides scalaris	Ladder-backed Woodpecker	H	L	H	**L**
Picoides stricklandi	Strickland's Woodpecker	H	L	L	**L**
Picoides tridactylus	Eurasian Three-toed Woodpecker	H	L	L	**L**
Picoides villosus	Hairy Woodpecker	H	L	H	**L**
Piculus aurulentus	White-browed Woodpecker	H	U	L	**L**
Piculus callopterus	Stripe-cheeked Woodpecker	H	U	L	**L**
Piculus chrysochloros	Golden-green Woodpecker	H	U	L	**L**
Piculus flavigula	Yellow-throated Woodpecker	H	U	H	**L**
Piculus leucolaemus	White-throated Woodpecker	H	U	H	**L**
Piculus litae	Lita Woodpecker	H	U	H	**L**
Piculus simplex	Rufous-winged Woodpecker	H	L	H	**L**
Picumnus albosquamatus	White-wedged Piculet	U	U	L	**L**
Picumnus aurifrons	Bar-breasted Piculet	H	U	L	**L**

Picumnus castelnau	Plain-breasted Piculet	H	U	H	**L**
Picumnus cinnamomeus	Chestnut Piculet	H	U	H	**L**
Picumnus cirratus	White-barred Piculet	U	L	L	**L**
Picumnus dorbignyanus	Ocellated Piculet	H	U	H	**L**
Picumnus exilis	Golden-spangled Piculet	H	U	H	**L**
Picumnus fulvescens	Tawny Piculet	U	U	L	**L**
Picumnus fuscus	Rusty-necked Piculet	H	U	U	**L**
Picumnus granadensis	Greyish Piculet	U	U	H	**L**
Picumnus innominatus	Speckled Piculet	H	L	U	**L**
Picumnus lafresnayi	Lafresnaye's Piculet	H	H	H	**H**
Picumnus limae	Ochraceous Piculet	H	U	L	**L**
Picumnus minutissimus	Guianan Piculet	H	L	H	**L**
Picumnus nebulosus	Mottled Piculet	H	U	L	**L**
Picumnus olivaceus	Olivaceous Piculet	U	L	L	**L**
Picumnus pumilus	Orinoco Piculet	H	U	H	**L**
Picumnus pygmaeus	Spotted Piculet	U	U	L	**L**
Picumnus rufiventris	Rufous-breasted Piculet	H	U	H	**L**
Picumnus sclateri	Ecuadorian Piculet	H	U	H	**L**
Picumnus spilogaster	White-bellied Piculet	H	U	H	**L**
Picumnus squamulatus	Scaled Piculet	U	U	L	**L**
Picumnus steindachneri	Speckle-chested Piculet	H	U	H	**L**
Picumnus subtilis	Fine-barred Piculet	H	U	H	**L**
Picumnus temminckii	Ochre-collared Piculet	H	U	L	**L**
Picumnus varzeae	Varzea Piculet	H	U	H	**L**
Picus awokera	Japanese Woodpecker	U	L	H	**L**
Picus canus	Grey-faced Woodpecker	H	L	H	**L**
Picus chlorolophus	Lesser Yellownape	H	L	U	**L**
Picus erythropygius	Black-headed Woodpecker	H	L	H	**L**
Picus flavinucha	Greater Yellownape	H	L	H	**L**
Picus mentalis	Checker-throated Woodpecker	H	L	U	**L**
Picus mineaceus	Banded Woodpecker	H	L	U	**L**
Picus puniceus	Crimson-winged Woodpecker	U	L	U	**L**
Picus rabieri	Red-collared Woodpecker	H	U	H	**L**
Picus squamatus	Scaly-bellied Woodpecker	U	L	U	**L**
Picus vaillantii	Levaillant's Woodpecker	H	L	H	**L**
Picus viridanus	Streak-breasted Woodpecker	H	L	U	**L**
Picus viridis	Eurasian Green Woodpecker	H	L	H	**L**
Picus vittatus	Laced Woodpecker	U	L	U	**L**
Picus xanthopygaeus	Streak-throated Woodpecker	H	L	L	**L**
Piezorhina cinerea	Cinereous Finch	H	U	H	**L**
Pilherodius pileatus	Capped Heron	H	H	H	**H**
Pinarocorys erythropygia	Rufous-rumped Lark	U	H	H	**L**

Pinarocorys nigricans	Dusky Lark	U	H	H	**L**
Pinaroloxias inornata	Cocos Finch	H	H	U	**L**
Pinarornis plumosus	Boulder Chat	U	L	U	**L**
Pinicola enucleator	Pine Grosbeak	H	L	H	**L**
Pinicola subhimachala	Crimson-browed Finch	U	U	U	**L**
Pionites leucogaster	White-bellied Parrot	H	H	L	**L**
Pionites melanocephalus	Black-headed Parrot	H	H	H	**H**
Pionopsitta pileata	Pileated Parrot	H	H	L	**L**
Pionus chalcopterus	Bronze-winged Parrot	U	H	H	**L**
Pionus fuscus	Dusky Parrot	H	H	H	**H**
Pionus maximiliani	Scaly-headed Parrot	U	H	L	**L**
Pionus menstruus	Blue-headed Parrot	H	H	H	**H**
Pionus senilis	White-crowned Parrot	L	H	H	**L**
Pionus sordidus	Red-billed Parrot	U	H	H	**L**
Pionus tumultuosus	Speckle-faced Parrot	H	H	L	**L**
Pipile cujubi	Red-throated Piping-guan	H	U	H	**L**
Pipile cumanensis	Blue-throated Piping-guan	U	L	U	**L**
Pipile jacutinga	Black-fronted Piping-guan	H	L	L	**L**
Pipile pipile	Trinidad Piping-guan	H	H	H	**H**
Pipilo aberti	Abert's Towhee	H	L	H	**L**
Pipilo albicollis	White-throated Towhee	L	L	L	**L**
Pipilo chlorurus	Green-tailed Towhee	H	L	H	**L**
Pipilo crissalis	California Towhee	H	L	L	**L**
Pipilo erythrophthalmus	Eastern Towhee	H	L	H	**L**
Pipilo fuscus	Canyon Towhee	H	L	H	**L**
Pipilo maculatus	Spotted Towhee	H	L	H	**L**
Pipilo ocai	Collared Towhee	L	L	L	**L**
Pipra aureola	Crimson-hooded Manakin	H	H	H	**H**
Pipra chloromeros	Round-tailed Manakin	H	H	H	**H**
Pipra cornuta	Scarlet-horned Manakin	H	H	H	**H**
Pipra erythrocephala	Golden-headed Manakin	H	H	H	**H**
Pipra fasciicauda	Band-tailed Manakin	H	H	L	**L**
Pipra filicauda	Wire-tailed Manakin	H	H	H	**H**
Pipra mentalis	Red-capped Manakin	H	H	H	**H**
Pipra pipra	White-crowned Manakin	H	H	H	**H**
Pipra rubrocapilla	Red-headed Manakin	H	H	H	**H**
Pipraeidea melanonota	Fawn-breasted Tanager	U	U	L	**L**
Pipreola arcuata	Barred Fruiteater	H	U	L	**L**
Pipreola aureopectus	Golden-breasted Fruiteater	H	U	H	**L**
Pipreola chlorolepidota	Fiery-throated Fruiteater	H	U	H	**L**
Pipreola formosa	Handsome Fruiteater	H	U	H	**L**
Pipreola frontalis	Scarlet-breasted Fruiteater	H	U	H	**L**
Pipreola intermedia	Band-tailed Fruiteater	H	U	H	**L**
Pipreola jucunda	Orange-breasted Fruiteater	H	H	H	**H**
Pipreola lubomirskii	Black-chested Fruiteater	H	U	H	**L**

Pipreola pulchra	Masked Fruiteater	H	U	H	**L**
Pipreola riefferii	Green-and-black Fruiteater	H	H	H	**H**
Pipreola whitelyi	Red-banded Fruiteater	H	U	H	**L**
Piprites chloris	Wing-barred Piprites	H	H	U	**L**
Piprites griseiceps	Grey-headed Piprites	H	H	U	**L**
Piprites pileata	Black-capped Piprites	H	H	L	**L**
Piranga bidentata	Flame-coloured Tanager	L	L	H	**L**
Piranga erythrocephala	Red-headed Tanager	L	U	L	**L**
Piranga flava	Hepatic Tanager	L	L	L	**L**
Piranga leucoptera	White-winged Tanager	L	U	L	**L**
Piranga ludoviciana	Western Tanager	H	L	H	**L**
Piranga olivacea	Scarlet Tanager	L	L	L	**L**
Piranga roseogularis	Rose-throated Tanager	L	U	H	**L**
Piranga rubra	Summer Tanager	L	L	L	**L**
Piranga rubriceps	Red-hooded Tanager	H	U	H	**L**
Pitangus lictor	Lesser Kiskadee	H	L	H	**L**
Pitangus sulphuratus	Great Kiskadee	L	L	L	**L**
Pithecophaga jefferyi	Philippine Eagle	H	H	L	**L**
Pithys albifrons	White-plumed Antbird	H	H	H	**H**
Pithys castaneus	White-masked Antbird	H	H	H	**H**
Pitohui cristatus	Crested Pitohui	H	H	L	**L**
Pitohui dichrous	Hooded Pitohui	U	H	H	**L**
Pitohui ferrugineus	Rusty Pitohui	U	H	L	**L**
Pitohui incertus	White-bellied Pitohui	H	H	H	**H**
Pitohui kirhocephalus	Variable Pitohui	U	H	L	**L**
Pitohui nigrescens	Black Pitohui	H	H	H	**H**
Pitta anerythra	Black-faced Pitta	H	U	L	**L**
Pitta angolensis	African Pitta	U	L	L	**L**
Pitta arcuata	Blue-banded Pitta	H	H	U	**L**
Pitta baudii	Blue-headed Pitta	H	H	H	**H**
Pitta brachyura	Indian Pitta	H	L	L	**L**
Pitta caerulea	Giant Pitta	H	H	H	**H**
Pitta cyanea	Blue Pitta	H	H	U	**L**
Pitta dohertyi	Sula Pitta	H	U	L	**L**
Pitta elegans	Elegant Pitta	U	L	U	**L**
Pitta elliotii	Bar-bellied Pitta	H	L	U	**L**
Pitta erythrogaster	Red-bellied Pitta	U	L	L	**L**
Pitta granatina	Garnet Pitta	H	H	H	**H**
Pitta guajana	Banded Pitta	H	L	H	**L**
Pitta gurneyi	Gurney's Pitta	H	L	H	**L**
Pitta iris	Rainbow Pitta	H	L	L	**L**
Pitta kochi	Whiskered Pitta	H	U	H	**L**
Pitta maxima	Ivory-breasted Pitta	H	H	H	**H**
Pitta megarhyncha	Mangrove Pitta	H	L	H	**L**
Pitta moluccensis	Blue-winged Pitta	U	L	L	**L**

Pitta nipalensis	Blue-naped Pitta	H	L	U	**L**
Pitta nympha	Fairy Pitta	H	L	L	**L**
Pitta oatesi	Rusty-naped Pitta	H	H	U	**L**
Pitta phayrei	Eared Pitta	H	H	U	**L**
Pitta reichenowi	Green-breasted Pitta	H	L	H	**L**
Pitta schneideri	Schneider's Pitta	H	H	H	**H**
Pitta sordida	Hooded Pitta	U	L	U	**L**
Pitta soror	Blue-rumped Pitta	U	L	U	**L**
Pitta steerii	Azure-breasted Pitta	H	H	H	**H**
Pitta superba	Superb Pitta	H	H	H	**H**
Pitta venusta	Graceful Pitta	H	H	H	**H**
Pitta versicolor	Noisy Pitta	U	L	L	**L**
Pittasoma michleri	Black-crowned Antpitta	H	H	L	**L**
Pittasoma rufopileatum	Rufous-crowned Antpitta	H	H	H	**H**
Pityriasis gymnocephala	Bornean Bristlehead	H	U	H	**L**
Platalea ajaja	Roseate Spoonbill	L	H	L	**L**
Platalea alba	African Spoonbill	L	H	L	**L**
Platalea flavipes	Yellow-billed Spoonbill	U	H	U	**L**
Platalea leucorodia	Eurasian Spoonbill	L	H	H	**L**
Platalea minor	Black-faced Spoonbill	H	H	H	**H**
Platalea regia	Royal Spoonbill	U	H	U	**L**
Platycercus adscitus	Pale-headed Rosella	U	H	U	**L**
Platycercus caledonicus	Green Rosella	H	L	H	**L**
Platycercus elegans	Crimson Rosella	H	H	L	**L**
Platycercus eximius	Eastern Rosella	U	L	U	**L**
Platycercus icterotis	Western Rosella	U	H	U	**L**
Platycercus venustus	Northern Rosella	U	L	U	**L**
Platylophus galericulatus	Crested Jay	H	H	H	**H**
Platyrinchus cancrominus	Stub-tailed Spadebill	H	H	H	**H**
Platyrinchus coronatus	Golden-crowned Spadebill	H	H	H	**H**
Platyrinchus flavigularis	Yellow-throated Spadebill	H	H	H	**H**
Platyrinchus leucoryphus	Russet-winged Spadebill	H	H	L	**L**
Platyrinchus mystaceus	White-throated Spadebill	U	H	L	**L**
Platyrinchus platyrhynchos	White-crested Spadebill	H	H	H	**H**
Platyrinchus saturatus	Cinnamon-crested Spadebill	H	H	H	**H**
Platysmurus leucopterus	Black Magpie	H	L	H	**L**
Platyspiza crassirostris	Vegetarian Finch	H	H	U	**L**
Platysteira albifrons	White-fronted Wattle-eye	U	H	L	**L**
Platysteira blissetti	Red-cheeked Wattle-eye	U	H	U	**L**
Platysteira castanea	Chestnut Wattle-eye	H	H	L	**L**
Platysteira chalybea	Black-necked Wattle-eye	H	H	L	**L**
Platysteira concreta	Yellow-bellied Wattle-eye	U	H	L	**L**
Platysteira cyanea	Brown-throated Wattle-eye	U	H	H	**L**
Platysteira jamesoni	Jameson's Wattle-eye	U	H	H	**L**

Platysteira laticincta	Banded Wattle-eye	H	H	H	**H**
Platysteira peltata	Black-throated Wattle-eye	U	H	L	**L**
Platysteira tonsa	White-spotted Wattle-eye	H	H	H	**H**
Plectorhyncha lanceolata	Striped Honeyeater	U	L	U	**L**
Plectrophenax hyperboreus	McKay's Bunting	H	H	H	**H**
Plectrophenax nivalis	Snow Bunting	H	H	H	**H**
Plectropterus gambensis	Spur-winged Goose	L	H	L	**L**
Plegadis chihi	White-faced Ibis	H	H	L	**L**
Plegadis falcinellus	Glossy Ibis	L	H	L	**L**
Plegadis ridgwayi	Puna Ibis	H	H	H	**H**
Plocepasser donaldsoni	Donaldson-Smith's Sparrow-weaver	H	U	H	**L**
Plocepasser mahali	White-browed Sparrow-weaver	U	U	U	**L**
Plocepasser rufoscapulatus	Chestnut-backed Sparrow-weaver	H	U	U	**L**
Plocepasser superciliosus	Chestnut-crowned Sparrow-weaver	U	U	H	**L**
Ploceus albinucha	Maxwell's Black Weaver	H	U	L	**L**
Ploceus alienus	Strange Weaver	H	U	L	**L**
Ploceus angolensis	Bar-winged Weaver	H	H	U	**L**
Ploceus aurantius	Orange Weaver	U	U	H	**L**
Ploceus aureonucha	Golden-naped Weaver	H	U	H	**L**
Ploceus badius	Cinnamon Weaver	U	L	U	**L**
Ploceus baglafecht	Baglafecht Weaver	U	U	L	**L**
Ploceus bannermani	Bannerman's Weaver	H	H	L	**L**
Ploceus batesi	Bates's Weaver	H	H	H	**H**
Ploceus benghalensis	Black-breasted Weaver	U	L	U	**L**
Ploceus bertrandi	Bertrand's Weaver	U	U	L	**L**
Ploceus bicolor	Forest Weaver	U	U	L	**L**
Ploceus bojeri	Golden Palm Weaver	U	U	U	**L**
Ploceus burnieri	Kilombero Weaver	H	U	H	**L**
Ploceus capensis	Cape Weaver	H	H	L	**L**
Ploceus castaneiceps	Taveta Golden Weaver	H	U	H	**L**
Ploceus castanops	Northern Brown-throated Weaver	H	U	H	**L**
Ploceus cucullatus	Village Weaver	U	L	L	**L**
Ploceus dichrocephalus	Salvadori's Weaver	H	U	L	**L**
Ploceus dorsomaculatus	Yellow-capped Weaver	H	U	H	**L**
Ploceus flavipes	Yellow-legged Weaver	H	H	H	**H**
Ploceus galbula	Rueppell's Weaver	U	U	U	**L**
Ploceus golandi	Clarke's Weaver	H	H	H	**H**
Ploceus grandis	Giant Weaver	H	H	L	**L**
Ploceus heuglini	Heuglin's Masked-weaver	U	U	H	**L**
Ploceus hypoxanthus	Asian Golden Weaver	H	L	H	**L**

Ploceus insignis	Brown-capped Weaver	U	U	L	**L**
Ploceus intermedius	Lesser Masked Weaver	U	U	L	**L**
Ploceus jacksoni	Golden-backed Weaver	U	U	H	**L**
Ploceus katangae	Katanga Masked-weaver	U	U	U	**L**
Ploceus luteolus	Little Weaver	U	U	U	**L**
Ploceus manyar	Streaked Weaver	U	L	U	**L**
Ploceus megarhynchus	Yellow Weaver	H	L	H	**L**
Ploceus melanocephalus	Black-headed Weaver	U	U	U	**L**
Ploceus melanogaster	Black-billed Weaver	H	U	L	**L**
Ploceus nelicourvi	Nelicourvi Weaver	H	L	U	**L**
Ploceus nicolli	Usambara Weaver	H	H	H	**H**
Ploceus nigerrimus	Vieillot's Black Weaver	U	U	L	**L**
Ploceus nigricollis	Black-necked Weaver	U	U	L	**L**
Ploceus nigrimentus	Black-chinned Weaver	L	U	L	**L**
Ploceus ocularis	Spectacled Weaver	U	U	L	**L**
Ploceus olivaceiceps	Olive-headed Weaver	H	U	L	**L**
Ploceus pelzelni	Slender-billed Weaver	U	U	L	**L**
Ploceus philippinus	Baya Weaver	U	L	L	**L**
Ploceus preussi	Preuss's Weaver	H	U	L	**L**
Ploceus princeps	Principe Golden Weaver	H	H	L	**L**
Ploceus reichardi	Tanzania Masked-weaver	U	U	H	**L**
Ploceus rubiginosus	Chestnut Weaver	H	U	U	**L**
Ploceus ruweti	Lake Lufira Weaver	U	U	H	**L**
Ploceus sakalava	Sakalava Weaver	U	L	U	**L**
Ploceus sanctithomae	Sao Tome Weaver	H	H	L	**L**
Ploceus spekei	Speke's Weaver	U	U	L	**L**
Ploceus spekeoides	Fox's Weaver	H	U	H	**L**
Ploceus subaureus	African Golden Weaver	U	U	U	**L**
Ploceus subpersonatus	Loango Weaver	H	U	H	**L**
Ploceus superciliosus	Compact Weaver	U	U	L	**L**
Ploceus taeniopterus	Northern Masked-weaver	U	U	U	**L**
Ploceus temporalis	Bocage's Weaver	U	L	U	**L**
Ploceus tricolor	Yellow-mantled Weaver	H	U	L	**L**
Ploceus velatus	Southern Masked-weaver	H	U	L	**L**
Ploceus vitellinus	Vitelline Masked-weaver	U	H	U	**L**
Ploceus weynsi	Weyns's Weaver	H	U	H	**L**
Ploceus xanthops	Holub's Golden Weaver	U	U	L	**L**
Ploceus xanthopterus	Southern Brown-throated Weaver	U	U	U	**L**
Pluvialis apricaria	Eurasian Golden Plover	H	H	H	**H**
Pluvialis dominica	American Golden Plover	L	H	L	**L**
Pluvialis fulva	Pacific Golden Plover	L	H	H	**L**
Pluvialis squatarola	Grey Plover	L	H	H	**L**
Pluvianellus socialis	Magellanic Plover	H	H	H	**H**
Pluvianus aegyptius	Egyptian Plover	L	H	H	**L**

Pnoepyga albiventer	Scaly-breasted Wren-babbler	U	L	U	**L**
Pnoepyga formosana	Taiwan Wren-babbler	H	H	U	**L**
Pnoepyga immaculata	Nepal Wren-babbler	H	H	H	**H**
Pnoepyga pusilla	Pygmy Wren-babbler	H	H	U	**L**
Podager nacunda	Nacunda Nighthawk	U	H	L	**L**
Podargus ocellatus	Marbled Frogmouth	U	H	U	**L**
Podargus papuensis	Papuan Frogmouth	U	H	U	**L**
Podargus strigoides	Tawny Frogmouth	H	H	L	**L**
Podica senegalensis	African Finfoot	L	H	L	**L**
Podiceps auritus	Horned Grebe	H	H	H	**H**
Podiceps cristatus	Great Crested Grebe	H	H	H	**H**
Podiceps gallardoi	Hooded Grebe	H	H	H	**H**
Podiceps grisegena	Red-necked Grebe	H	H	L	**L**
Podiceps major	Great Grebe	U	H	H	**L**
Podiceps nigricollis	Black-necked Grebe	H	H	H	**H**
Podiceps occipitalis	Silvery Grebe	H	H	L	**L**
Podiceps taczanowskii	Junin Grebe	H	H	H	**H**
Podilymbus podiceps	Pied-billed Grebe	L	L	L	**L**
Podoces biddulphi	Xinjiang Ground-jay	H	H	H	**H**
Podoces hendersoni	Mongolian Ground-jay	H	H	U	**L**
Podoces panderi	Turkestan Ground-jay	H	H	U	**L**
Podoces pleskei	Iranian Ground-jay	H	H	U	**L**
Poecilodryas albonotata	Black-throated Robin	U	U	U	**L**
Poecilodryas brachyura	Black-chinned Robin	H	U	U	**L**
Poecilodryas hypoleuca	Black-sided Robin	H	U	L	**L**
Poecilodryas placens	Olive-yellow Robin	H	U	U	**L**
Poecilodryas superciliosa	White-browed Robin	U	H	U	**L**
Poecilotriccus albifacies	White-cheeked Tody-tyrant	H	H	H	**H**
Poecilotriccus calopterus	Golden-winged Tody-flycatcher	U	U	H	**L**
Poecilotriccus capitalis	Black-and-white Tody-tyrant	H	U	H	**L**
Poecilotriccus fumifrons	Smoky-fronted Tody-flycatcher	H	U	H	**L**
Poecilotriccus latirostris	Rusty-fronted Tody-flycatcher	H	U	L	**L**
Poecilotriccus luluae	Lulu's Tody-tyrant	H	H	H	**H**
Poecilotriccus plumbeiceps	Ochre-faced Tody-flycatcher	H	L	L	**L**
Poecilotriccus pulchellus	Black-backed Tody-flycatcher	U	U	H	**L**
Poecilotriccus ruficeps	Rufous-crowned Tody-tyrant	H	U	H	**L**
Poecilotriccus russatus	Ruddy Tody-flycatcher	H	H	H	**H**
Poecilotriccus senex	Buff-cheeked Tody-flycatcher	H	H	H	**H**
Poecilotriccus sylvia	Slate-headed Tody-flycatcher	L	H	H	**L**
Poeoptera kenricki	Kenrick's Starling	H	U	L	**L**
Poeoptera lugubris	Narrow-tailed Starling	H	L	L	**L**

Poeoptera stuhlmanni	Stuhlmann's Starling	H	U	L	**L**
Poephila acuticauda	Long-tailed Finch	U	L	U	**L**
Poephila cincta	Black-throated Finch	U	L	L	**L**
Poephila personata	Masked Finch	U	L	U	**L**
Pogoniulus atroflavus	Red-rumped Tinkerbird	H	H	H	**H**
Pogoniulus bilineatus	Yellow-rumped Tinkerbird	H	H	L	**L**
Pogoniulus chrysoconus	Yellow-fronted Tinkerbird	H	H	L	**L**
Pogoniulus coryphaeus	Western Green-tinkerbird	U	H	L	**L**
Pogoniulus leucomystax	Moustached Green-tinkerbird	U	H	H	**L**
Pogoniulus makawai	White-chested Tinkerbird	H	H	H	**H**
Pogoniulus pusillus	Red-fronted Tinkerbird	H	H	L	**L**
Pogoniulus scolopaceus	Speckled Tinkerbird	H	H	H	**H**
Pogoniulus simplex	African Green-tinkerbird	U	H	L	**L**
Pogoniulus subsulphureus	Yellow-throated Tinkerbird	H	H	H	**H**
Pogonocichla stellata	White-starred Robin	U	L	L	**L**
Poicephalus crassus	Niam-niam Parrot	H	H	H	**H**
Poicephalus cryptoxanthus	Brown-headed Parrot	U	H	L	**L**
Poicephalus flavifrons	Yellow-fronted Parrot	U	H	L	**L**
Poicephalus gulielmi	Red-fronted Parrot	U	H	H	**L**
Poicephalus meyeri	Meyer's Parrot	U	H	U	**L**
Poicephalus robustus	Brown-necked Parrot	U	H	L	**L**
Poicephalus rueppellii	Rueppell's Parrot	H	H	L	**L**
Poicephalus rufiventris	Red-bellied Parrot	U	H	U	**L**
Poicephalus senegalus	Senegal Parrot	U	H	H	**L**
Polemaetus bellicosus	Martial Eagle	L	H	L	**L**
Polihierax insignis	White-rumped Falcon	U	H	L	**L**
Polihierax semitorquatus	Pygmy Falcon	H	H	L	**L**
Poliocephalus poliocephalus	Hoary-headed Grebe	H	L	L	**L**
Poliocephalus rufopectus	New Zealand Grebe	H	L	H	**L**
Poliolais lopezi	White-tailed Warbler	U	H	L	**L**
Polioptila albiloris	White-lored Gnatcatcher	L	L	L	**L**
Polioptila caerulea	Blue-grey Gnatcatcher	H	L	L	**L**
Polioptila californica	California Gnatcatcher	H	L	L	**L**
Polioptila clementsi	Iquitos Gnatcatcher	H	H	H	**H**
Polioptila dumicola	Masked Gnatcatcher	U	U	L	**L**
Polioptila guianensis	Guianan Gnatcatcher	H	H	H	**H**
Polioptila lactea	Creamy-bellied Gnatcatcher	H	U	L	**L**
Polioptila lembeyei	Cuban Gnatcatcher	H	L	H	**L**
Polioptila melanura	Black-tailed Gnatcatcher	H	L	H	**L**
Polioptila nigriceps	Black-capped Gnatcatcher	L	U	H	**L**
Polioptila plumbea	Tropical Gnatcatcher	L	L	H	**L**
Polioptila schistaceigula	Slate-throated Gnatcatcher	H	H	H	**H**

Polioxolmis rufipennis	Rufous-webbed Bush-tyrant	H	U	H	**L**
Polyboroides radiatus	Madagascar Harrier-hawk	H	H	L	**L**
Polyboroides typus	African Harrier-hawk	L	H	L	**L**
Polyonymus caroli	Bronze-tailed Comet	H	U	H	**L**
Polyplectron bicalcaratum	Grey Peacock-pheasant	H	H	U	**L**
Polyplectron chalcurum	Bronze-tailed Peacock-pheasant	H	H	H	**H**
Polyplectron germaini	Germain's Peacock-pheasant	H	H	L	**L**
Polyplectron inopinatum	Mountain Peacock-pheasant	H	H	H	**H**
Polyplectron malacense	Malayan Peacock-pheasant	H	H	L	**L**
Polyplectron napoleonis	Palawan Peacock-pheasant	H	H	H	**H**
Polyplectron schleiermacheri	Bornean Peacock-pheasant	H	H	H	**H**
Polysticta stelleri	Steller's Eider	H	H	H	**H**
Polystictus pectoralis	Bearded Tachuri	U	L	L	**L**
Polystictus superciliaris	Grey-backed Tachuri	U	H	L	**L**
Polytelis alexandrae	Princess Parrot	H	L	L	**L**
Polytelis anthopeplus	Regent Parrot	H	H	L	**L**
Polytelis swainsonii	Superb Parrot	H	L	L	**L**
Polytmus guainumbi	White-tailed Goldenthroat	U	H	L	**L**
Polytmus milleri	Tepui Goldenthroat	H	H	H	**H**
Polytmus theresiae	Green-tailed Goldenthroat	H	H	H	**H**
Pomarea dimidiata	Rarotonga Monarch	H	H	U	**L**
Pomarea iphis	Iphis Monarch	H	H	U	**L**
Pomarea mendozae	Marquesan Monarch	H	H	U	**L**
Pomarea nigra	Tahiti Monarch	H	H	U	**L**
Pomarea whitneyi	Fatuhiva Monarch	H	H	U	**L**
Pomatorhinus erythrocnemis	Spot-breasted Scimitar-babbler	U	L	U	**L**
Pomatorhinus erythrogenys	Rusty-cheeked Scimitar-babbler	U	L	H	**L**
Pomatorhinus ferruginosus	Coral-billed Scimitar-babbler	H	H	U	**L**
Pomatorhinus horsfieldii	Indian Scimitar-babbler	U	L	U	**L**
Pomatorhinus hypoleucos	Large Scimitar-babbler	U	L	L	**L**
Pomatorhinus montanus	Chestnut-backed Scimitar-babbler	U	L	U	**L**
Pomatorhinus musicus	Taiwan Scimitar-babbler	H	H	U	**L**
Pomatorhinus ochraceiceps	Red-billed Scimitar-babbler	H	H	U	**L**
Pomatorhinus ruficollis	Streak-breasted Scimitar-babbler	U	L	U	**L**
Pomatorhinus schisticeps	White-browed Scimitar-babbler	U	L	U	**L**
Pomatostomus halli	Hall's Babbler	U	H	U	**L**
Pomatostomus isidorei	New Guinea Babbler	U	H	U	**L**
Pomatostomus ruficeps	Chestnut-crowned Babbler	U	H	U	**L**

Pomatostomus superciliosus	White-browed Babbler	U	H	U	**L**
Pomatostomus temporalis	Grey-crowned Babbler	H	H	L	**L**
Pooecetes gramineus	Vesper Sparrow	H	L	H	**L**
Poospiza alticola	Plain-tailed Warbling-finch	H	U	H	**L**
Poospiza baeri	Tucuman Mountain-finch	H	U	H	**L**
Poospiza boliviana	Bolivian Warbling-finch	U	U	H	**L**
Poospiza caesar	Chestnut-breasted Mountain-finch	H	U	H	**L**
Poospiza cinerea	Cinereous Warbling-finch	H	H	L	**L**
Poospiza erythrophrys	Rusty-browed Warbling-finch	H	H	H	**H**
Poospiza garleppi	Cochabamba Mountain-finch	H	U	H	**L**
Poospiza hispaniolensis	Collared Warbling-finch	H	U	H	**L**
Poospiza hypochondria	Rufous-sided Warbling-finch	H	U	H	**L**
Poospiza lateralis	Red-rumped Warbling-finch	H	U	H	**L**
Poospiza melanoleuca	Black-capped Warbling-finch	H	U	L	**L**
Poospiza nigrorufa	Black-and-rufous Warbling-finch	H	U	L	**L**
Poospiza ornata	Cinnamon Warbling-finch	H	U	H	**L**
Poospiza rubecula	Rufous-breasted Warbling-finch	H	U	H	**L**
Poospiza thoracica	Bay-chested Warbling-finch	U	U	L	**L**
Poospiza torquata	Ringed Warbling-finch	H	U	L	**L**
Porphyrio alleni	Allen's Gallinule	L	L	L	**L**
Porphyrio flavirostris	Azure Gallinule	H	L	H	**L**
Porphyrio hochstetteri	Takahe	H	H	H	**H**
Porphyrio martinica	Yellow-legged Gallinule	H	L	L	**L**
Porphyrio porphyrio	Purple Swamphen	L	L	L	**L**
Porphyrolaema porphyrolaema	Purple-throated Cotinga	H	U	L	**L**
Porphyrospiza caerulescens	Blue Finch	U	U	L	**L**
Porzana albicollis	Ash-throated Crake	U	L	L	**L**
Porzana atra	Henderson Crake	H	H	U	**L**
Porzana carolina	Sora	H	L	L	**L**
Porzana cinerea	White-browed Crake	U	L	U	**L**
Porzana flaviventer	Yellow-breasted Crake	H	L	H	**L**
Porzana fluminea	Australian Crake	U	L	U	**L**
Porzana fusca	Ruddy-breasted Crake	U	L	U	**L**
Porzana parva	Little Crake	H	L	H	**L**
Porzana paykullii	Band-bellied Crake	U	L	H	**L**
Porzana porzana	Spotted Crake	H	L	H	**L**
Porzana pusilla	Baillon's Crake	L	L	H	**L**
Porzana spiloptera	Dot-winged Crake	H	U	H	**L**
Porzana tabuensis	Spotless Crake	U	L	U	**L**

Premnoplex brunnescens	Spotted Barbtail	H	H	L	**L**
Premnoplex tatei	White-throated Barbtail	H	H	H	**H**
Premnornis guttuligera	Rusty-winged Barbtail	H	H	L	**L**
Primolius auricollis	Yellow-collared Macaw	H	H	L	**L**
Primolius couloni	Blue-headed Macaw	H	H	H	**H**
Primolius maracana	Blue-winged Macaw	H	H	L	**L**
Prinia atrogularis	Hill Prinia	U	L	U	**L**
Prinia bairdii	Banded Prinia	H	L	U	**L**
Prinia buchanani	Rufous-fronted Prinia	U	U	U	**L**
Prinia burnesii	Rufous-vented Prinia	U	U	H	**L**
Prinia cinereocapilla	Grey-crowned Prinia	H	U	H	**L**
Prinia crinigera	Striated Prinia	U	L	U	**L**
Prinia familiaris	Bar-winged Prinia	U	U	L	**L**
Prinia flavicans	Black-chested Prinia	H	L	L	**L**
Prinia flaviventris	Yellow-bellied Prinia	U	L	U	**L**
Prinia fluviatilis	River Prinia	H	L	H	**L**
Prinia gracilis	Graceful Prinia	U	L	H	**L**
Prinia hodgsonii	Grey-breasted Prinia	U	L	H	**L**
Prinia hypoxantha	Saffron-breasted Prinia	H	H	L	**L**
Prinia inornata	Plain Prinia	U	L	U	**L**
Prinia leontica	White-eyed Prinia	H	H	L	**L**
Prinia leucopogon	White-chinned Prinia	U	H	H	**L**
Prinia maculosa	Karoo Prinia	H	H	L	**L**
Prinia molleri	Sao Tome Prinia	H	H	L	**L**
Prinia polychroa	Brown Prinia	U	L	U	**L**
Prinia robertsi	Briar Warbler	U	H	L	**L**
Prinia rufescens	Rufescent Prinia	U	L	H	**L**
Prinia socialis	Ashy Prinia	U	U	U	**L**
Prinia somalica	Pale Prinia	U	L	U	**L**
Prinia subflava	Tawny-flanked Prinia	U	L	L	**L**
Prinia sylvatica	Jungle Prinia	U	U	U	**L**
Prioniturus discurus	Blue-crowned Racquet-tail	H	L	L	**L**
Prioniturus flavicans	Yellowish-breasted Racquet-tail	H	U	H	**L**
Prioniturus luconensis	Green Racquet-tail	H	U	H	**L**
Prioniturus mada	Buru Racquet-tail	H	U	H	**L**
Prioniturus montanus	Luzon Racquet-tail	H	U	H	**L**
Prioniturus platenae	Blue-headed Racquet-tail	H	U	H	**L**
Prioniturus platurus	Golden-mantled Racquet-tail	U	U	U	**L**
Prioniturus verticalis	Blue-winged Racquet-tail	H	U	H	**L**
Prioniturus waterstradti	Mindanao Racquet-tail	H	H	H	**H**
Prionochilus maculatus	Yellow-breasted Flowerpecker	H	H	H	**H**
Prionochilus olivaceus	Olive-backed Flowerpecker	H	U	L	**L**
Prionochilus percussus	Crimson-breasted	H	H	H	**H**

	Flowerpecker				
Prionochilus plateni	Palawan Flowerpecker	H	U	H	**L**
Prionochilus thoracicus	Scarlet-breasted Flowerpecker	H	U	H	**L**
Prionochilus xanthopygius	Yellow-rumped Flowerpecker	U	U	H	**L**
Prionodura newtoniana	Golden Bowerbird	H	H	L	**L**
Prionops alberti	Yellow-crested Helmet-shrike	H	H	H	**H**
Prionops caniceps	Chestnut-bellied Helmet-shrike	U	H	U	**L**
Prionops gabela	Gabela Helmet-shrike	H	H	H	**H**
Prionops plumatus	White Helmet-shrike	U	H	L	**L**
Prionops poliolophus	Grey-crested Helmet-shrike	H	H	H	**H**
Prionops retzii	Retz's Helmet-shrike	U	H	L	**L**
Prionops scopifrons	Chestnut-fronted Helmet-shrike	U	H	L	**L**
Priotelus roseigaster	Hispaniolan Trogon	H	H	H	**H**
Priotelus temnurus	Cuban Trogon	H	H	H	**H**
Probosciger aterrimus	Palm Cockatoo	H	H	L	**L**
Procellaria aequinoctialis	White-chinned Petrel	H	H	H	**H**
Procellaria cinerea	Grey Petrel	H	H	H	**H**
Procellaria conspicillata	Spectacled Petrel	H	H	L	**L**
Procellaria parkinsoni	Parkinson's Petrel	H	H	L	**L**
Procellaria westlandica	Westland Petrel	H	H	H	**H**
Procelsterna cerulea	Blue Noddy	L	H	L	**L**
Procnias albus	White Bellbird	H	U	H	**L**
Procnias averano	Bearded Bellbird	H	H	L	**L**
Procnias nudicollis	Bare-throated Bellbird	H	H	L	**L**
Procnias tricarunculatus	Three-wattled Bellbird	H	U	H	**L**
Prodotiscus insignis	Cassin's Honeyguide	H	H	L	**L**
Prodotiscus regulus	Wahlberg's Honeyguide	U	L	U	**L**
Prodotiscus zambesiae	Green-backed Honeyguide	U	H	L	**L**
Progne chalybea	Grey-breasted Martin	L	L	L	**L**
Progne cryptoleuca	Cuban Martin	U	L	H	**L**
Progne dominicensis	Caribbean Martin	U	L	H	**L**
Progne elegans	Southern Martin	U	L	L	**L**
Progne modesta	Galapagos Martin	H	L	H	**L**
Progne murphyi	Peruvian Martin	H	U	H	**L**
Progne sinaloae	Sinaloa Martin	H	U	H	**L**
Progne subis	Purple Martin	L	U	L	**L**
Progne tapera	Brown-chested Martin	U	L	L	**L**
Promerops cafer	Cape Sugarbird	H	H	H	**H**
Promerops gurneyi	Gurney's Sugarbird	H	H	L	**L**
Prosobonia cancellata	Tuamotu Sandpiper	H	H	H	**H**
Prosopeia personata	Masked Shining-parrot	H	H	H	**H**

Prosopeia splendens	Crimson Shining-parrot	H	H	L	**L**
Prosopeia tabuensis	Red Shining-parrot	H	L	L	**L**
Prosthemadera novaeseelandiae	Tui	H	L	H	**L**
Protonotaria citrea	Prothonotary Warbler	L	L	L	**L**
Prunella atrogularis	Black-throated Accentor	H	L	H	**L**
Prunella collaris	Alpine Accentor	H	L	L	**L**
Prunella fagani	Yemen Accentor	H	H	H	**H**
Prunella fulvescens	Brown Accentor	U	U	U	**L**
Prunella himalayana	Rufous-streaked Accentor	U	U	U	**L**
Prunella immaculata	Maroon-backed Accentor	U	L	U	**L**
Prunella koslowi	Mongolian Accentor	H	U	H	**L**
Prunella modularis	Hedge Accentor	H	L	H	**L**
Prunella montanella	Siberian Accentor	H	H	H	**H**
Prunella ocularis	Radde's Accentor	H	L	L	**L**
Prunella rubeculoides	Robin Accentor	U	U	U	**L**
Prunella rubida	Japanese Accentor	U	L	H	**L**
Prunella strophiata	Rufous-breasted Accentor	U	L	U	**L**
Psalidoprocne albiceps	White-headed Saw-wing	U	L	U	**L**
Psalidoprocne fuliginosa	Cameroon Mountain Saw-wing	U	H	H	**L**
Psalidoprocne nitens	Square-tailed Saw-wing	H	H	H	**H**
Psalidoprocne obscura	Fanti Saw-wing	U	H	U	**L**
Psalidoprocne pristoptera	Blue Saw-wing	U	H	L	**L**
Psaltria exilis	Pygmy Tit	H	L	H	**L**
Psaltriparus minimus	Bushtit	H	L	H	**L**
Psarisomus dalhousiae	Long-tailed Broadbill	U	L	H	**L**
Psarocolius angustifrons	Russet-backed Oropendola	U	H	L	**L**
Psarocolius atrovirens	Dusky-green Oropendola	U	U	H	**L**
Psarocolius bifasciatus	Olive Oropendola	H	H	H	**H**
Psarocolius cassini	Baudo Oropendola	H	H	L	**L**
Psarocolius decumanus	Crested Oropendola	U	H	H	**L**
Psarocolius guatimozinus	Black Oropendola	U	U	H	**L**
Psarocolius montezuma	Montezuma Oropendola	L	H	H	**L**
Psarocolius viridis	Green Oropendola	H	H	H	**H**
Psarocolius wagleri	Chestnut-headed Oropendola	L	H	H	**L**
Pselliophorus luteoviridis	Yellow-green Finch	H	H	H	**H**
Pselliophorus tibialis	Yellow-thighed Finch	U	H	H	**L**
Psephotus chrysopterygius	Golden-shouldered Parrot	H	L	L	**L**
Psephotus dissimilis	Hooded Parrot	U	L	L	**L**
Psephotus haematonotus	Red-rumped Parrot	H	L	L	**L**
Psephotus varius	Mulga Parrot	H	L	L	**L**
Pseudalaemon fremantlii	Short-tailed Lark	H	L	L	**L**

Pseudelaenia leucospodia	Grey-and-white Tyrannulet	H	L	H	**L**
Pseudeos fuscata	Dusky Lory	U	H	U	**L**
Pseudhirundo griseopyga	Grey-rumped Swallow	U	L	U	**L**
Pseudibis davisoni	White-shouldered Ibis	H	H	L	**L**
Pseudibis papillosa	Red-naped Ibis	H	H	U	**L**
Pseudoalcippe abyssinica	African Hill Babbler	H	H	L	**L**
Pseudobias wardi	Ward's Flycatcher	H	H	U	**L**
Pseudobulweria aterrima	Mascarene Petrel	H	H	H	**H**
Pseudobulweria becki	Beck's Petrel	H	H	L	**L**
Pseudobulweria macgillivrayi	Fiji Petrel	H	H	L	**L**
Pseudobulweria rostrata	Tahiti Petrel	L	H	H	**L**
Pseudocalyptomena graueri	African Green Broadbill	H	H	H	**H**
Pseudochelidon eurystomina	African River-martin	H	L	H	**L**
Pseudocolaptes boissonneautii	Streaked Tuftedcheek	H	H	L	**L**
Pseudocolaptes lawrencii	Buffy Tuftedcheek	H	H	H	**H**
Pseudocolopteryx acutipennis	Subtropical Doradito	U	L	H	**L**
Pseudocolopteryx dinelliana	Dinelli's Doradito	H	L	L	**L**
Pseudocolopteryx flaviventris	Warbling Doradito	H	L	L	**L**
Pseudocolopteryx sclateri	Crested Doradito	H	H	L	**L**
Pseudoleistes guirahuro	Yellow-rumped Marshbird	U	L	L	**L**
Pseudoleistes virescens	Brown-and-yellow Marshbird	H	L	L	**L**
Pseudonestor xanthophrys	Maui Parrotbill	H	H	H	**H**
Pseudonigrita arnaudi	Grey-headed Social-weaver	U	U	U	**L**
Pseudonigrita cabanisi	Black-capped Social-weaver	H	U	U	**L**
Pseudopodoces humilis	Tibetan Ground-tit	U	L	U	**L**
Pseudoscops clamator	Striped Owl	L	L	L	**L**
Pseudoscops grammicus	Jamaican Owl	H	H	H	**H**
Pseudoseisura cristata	Caatinga Cacholote	U	U	L	**L**
Pseudoseisura gutturalis	White-throated Cacholote	H	L	H	**L**
Pseudoseisura lophotes	Brown Cacholote	H	L	L	**L**
Pseudoseisura unirufa	Rufous Cacholote	U	U	L	**L**
Pseudotriccus pelzelni	Bronze-olive Pygmy-tyrant	H	H	H	**H**
Pseudotriccus ruficeps	Rufous-headed Pygmy-tyrant	H	H	L	**L**
Pseudotriccus simplex	Hazel-fronted Pygmy-tyrant	H	H	H	**H**
Psilopogon pyrolophus	Fire-tufted Barbet	H	H	L	**L**
Psilopsiagon aurifrons	Mountain Parakeet	H	H	H	**H**
Psilopsiagon aymara	Grey-hooded Parakeet	H	H	H	**H**
Psilorhamphus guttatus	Spotted Bamboowren	H	U	L	**L**

Psittacella brehmii	Brehm's Tiger-parrot	H	U	U	**L**
Psittacella madaraszi	Madarasz's Tiger-parrot	U	U	U	**L**
Psittacella modesta	Modest Tiger-parrot	U	U	H	**L**
Psittacella picta	Painted Tiger-parrot	U	U	H	**L**
Psittacula alexandri	Red-breasted Parakeet	U	H	U	**L**
Psittacula calthropae	Emerald-collared Parakeet	U	H	H	**L**
Psittacula caniceps	Nicobar Parakeet	H	H	H	**H**
Psittacula columboides	Malabar Parakeet	U	H	U	**L**
Psittacula cyanocephala	Plum-headed Parakeet	U	H	L	**L**
Psittacula derbiana	Derbyan Parakeet	U	H	U	**L**
Psittacula eques	Mauritius Parakeet	H	H	H	**H**
Psittacula eupatria	Alexandrine Parakeet	U	H	U	**L**
Psittacula finschii	Grey-headed Parakeet	U	H	U	**L**
Psittacula himalayana	Slaty-headed Parakeet	H	H	U	**L**
Psittacula krameri	Rose-ringed Parakeet	U	H	H	**L**
Psittacula longicauda	Long-tailed Parakeet	H	H	H	**H**
Psittacula roseata	Blossom-headed Parakeet	U	H	U	**L**
Psittaculirostris desmarestii	Large Fig-parrot	H	U	U	**L**
Psittaculirostris edwardsii	Edwards's Fig-parrot	U	U	H	**L**
Psittaculirostris salvadorii	Salvadori's Fig-parrot	H	U	H	**L**
Psittacus erithacus	Grey Parrot	H	H	L	**L**
Psitteuteles goldiei	Goldie's Lorikeet	H	H	U	**L**
Psitteuteles iris	Iris Lorikeet	H	H	H	**H**
Psitteuteles versicolor	Varied Lorikeet	U	L	L	**L**
Psittinus cyanurus	Blue-rumped Parrot	H	L	H	**L**
Psittirostra psittacea	Ou	H	H	H	**H**
Psittrichas fulgidus	Pesquet's Parrot	H	H	H	**H**
Psophia crepitans	Grey-winged Trumpeter	H	H	H	**H**
Psophia leucoptera	Pale-winged Trumpeter	H	H	L	**L**
Psophia viridis	Dark-winged Trumpeter	H	H	H	**H**
Psophocichla litsitsirupa	Groundscraper Thrush	U	L	U	**L**
Psophodes cristatus	Chirruping Wedgebill	U	H	U	**L**
Psophodes nigrogularis	Western Whipbird	H	H	H	**H**
Psophodes occidentalis	Chiming Wedgebill	U	H	U	**L**
Psophodes olivaceus	Eastern Whipbird	H	H	L	**L**
Pteridophora alberti	King-of-Saxony Bird-of-paradise	H	H	H	**H**
Pterocles alchata	Pin-tailed Sandgrouse	H	L	H	**L**
Pterocles bicinctus	Double-banded Sandgrouse	H	L	L	**L**
Pterocles burchelli	Burchell's Sandgrouse	H	L	H	**L**
Pterocles coronatus	Crowned Sandgrouse	H	L	H	**L**
Pterocles decoratus	Black-faced Sandgrouse	U	L	U	**L**
Pterocles exustus	Chestnut-bellied Sandgrouse	U	L	U	**L**
Pterocles gutturalis	Yellow-throated Sandgrouse	U	L	U	**L**

Pterocles indicus	Painted Sandgrouse	U	L	U	**L**
Pterocles lichtensteinii	Lichtenstein's Sandgrouse	H	L	H	**L**
Pterocles namaqua	Namaqua Sandgrouse	H	L	H	**L**
Pterocles orientalis	Black-bellied Sandgrouse	H	L	H	**L**
Pterocles personatus	Madagascar Sandgrouse	U	L	U	**L**
Pterocles quadricinctus	Four-banded Sandgrouse	U	L	U	**L**
Pterocles senegallus	Spotted Sandgrouse	H	L	H	**L**
Pterocnemia pennata	Lesser Rhea	U	H	U	**L**
Pterodroma alba	Phoenix Petrel	L	H	L	**L**
Pterodroma arminjoniana	Trindade Petrel	H	H	U	**L**
Pterodroma atrata	Henderson Petrel	H	H	U	**L**
Pterodroma axillaris	Chatham Petrel	H	H	U	**L**
Pterodroma baraui	Barau's Petrel	H	H	H	**H**
Pterodroma brevipes	Collared Petrel	H	H	L	**L**
Pterodroma cahow	Bermuda Petrel	H	H	U	**L**
Pterodroma caribbaea	Jamaica Petrel	H	H	H	**H**
Pterodroma cervicalis	White-necked Petrel	L	H	L	**L**
Pterodroma cookii	Cook's Petrel	H	H	L	**L**
Pterodroma defilippiana	De Filippi's Petrel	H	H	H	**H**
Pterodroma externa	Juan Fernandez Petrel	H	H	H	**H**
Pterodroma feae	Fea's Petrel	H	H	H	**H**
Pterodroma hasitata	Black-capped Petrel	H	H	L	**L**
Pterodroma heraldica	Herald Petrel	U	H	H	**L**
Pterodroma hypoleuca	Bonin Petrel	L	H	L	**L**
Pterodroma incerta	Atlantic Petrel	H	H	L	**L**
Pterodroma inexpectata	Mottled Petrel	L	H	L	**L**
Pterodroma lessonii	White-headed Petrel	H	H	H	**H**
Pterodroma leucoptera	Gould's Petrel	H	H	L	**L**
Pterodroma longirostris	Stejneger's Petrel	H	H	H	**H**
Pterodroma macroptera	Great-winged Petrel	H	H	H	**H**
Pterodroma madeira	Zino's Petrel	H	H	H	**H**
Pterodroma magentae	Magenta Petrel	H	H	U	**L**
Pterodroma mollis	Soft-plumaged Petrel	H	H	H	**H**
Pterodroma neglecta	Kermadec Petrel	L	H	L	**L**
Pterodroma nigripennis	Black-winged Petrel	L	H	L	**L**
Pterodroma phaeopygia	Galapagos Petrel	H	H	H	**H**
Pterodroma pycrofti	Pycroft's Petrel	H	H	H	**H**
Pterodroma sandwichensis	Hawaiian Petrel	L	H	H	**L**
Pterodroma solandri	Providence Petrel	H	H	H	**H**
Pterodroma ultima	Murphy's Petrel	L	H	L	**L**
Pteroglossus aracari	Black-necked Aracari	H	H	H	**H**
Pteroglossus azara	Ivory-billed Aracari	H	H	H	**H**
Pteroglossus bailloni	Saffron Toucanet	H	H	L	**L**
Pteroglossus	Curl-crested Aracari	H	H	L	**L**

beauharnaesii					
Pteroglossus bitorquatus	Red-necked Aracari	H	H	H	**H**
Pteroglossus castanotis	Chestnut-eared Aracari	H	H	L	**L**
Pteroglossus frantzii	Fiery-billed Aracari	H	H	H	**H**
Pteroglossus inscriptus	Lettered Aracari	H	H	L	**L**
Pteroglossus pluricinctus	Many-banded Aracari	H	H	H	**H**
Pteroglossus torquatus	Collared Aracari	H	H	H	**H**
Pteroglossus viridis	Green Aracari	H	H	H	**H**
Pteronetta hartlaubii	Hartlaub's Duck	H	H	H	**H**
Pterophanes cyanopterus	Great Sapphirewing	U	H	H	**L**
Pteroptochos castaneus	Chestnut-throated Huet-huet	H	H	L	**L**
Pteroptochos megapodius	Moustached Turca	H	H	L	**L**
Pteroptochos tarnii	Black-throated Huet-huet	H	H	H	**H**
Pteruthius aenobarbus	Chestnut-fronted Shrike-babbler	H	H	U	**L**
Pteruthius flaviscapis	White-browed Shrike-babbler	H	H	U	**L**
Pteruthius melanotis	Black-eared Shrike-babbler	H	H	U	**L**
Pteruthius rufiventer	Black-headed Shrike-babbler	H	H	U	**L**
Pteruthius xanthochlorus	Green Shrike-babbler	H	H	U	**L**
Ptilinopus arcanus	Negros Fruit-dove	H	U	H	**L**
Ptilinopus aurantiifrons	Orange-fronted Fruit-dove	H	H	L	**L**
Ptilinopus bernsteinii	Scarlet-breasted Fruit-dove	H	H	H	**H**
Ptilinopus chalcurus	Makatea Fruit-dove	H	H	U	**L**
Ptilinopus cinctus	Banded Fruit-dove	H	H	U	**L**
Ptilinopus coralensis	Atoll Fruit-dove	U	H	U	**L**
Ptilinopus coronulatus	Coroneted Fruit-dove	U	H	L	**L**
Ptilinopus dohertyi	Red-naped Fruit-dove	H	H	H	**H**
Ptilinopus dupetithouarsii	White-capped Fruit-dove	U	H	U	**L**
Ptilinopus eugeniae	White-headed Fruit-dove	H	U	H	**L**
Ptilinopus fischeri	Red-eared Fruit-dove	U	H	H	**L**
Ptilinopus granulifrons	Carunculated Fruit-dove	H	U	H	**L**
Ptilinopus greyii	Red-bellied Fruit-dove	U	H	L	**L**
Ptilinopus huttoni	Rapa Fruit-dove	H	U	U	**L**
Ptilinopus hyogastrus	Grey-headed Fruit-dove	H	U	H	**L**
Ptilinopus insolitus	Knob-billed Fruit-dove	H	H	H	**H**
Ptilinopus insularis	Henderson Fruit-dove	H	H	U	**L**
Ptilinopus iozonus	Orange-bellied Fruit-dove	U	H	L	**L**
Ptilinopus jambu	Jambu Fruit-dove	H	H	H	**H**
Ptilinopus layardi	Whistling Dove	H	H	L	**L**
Ptilinopus leclancheri	Black-chinned Fruit-dove	H	H	L	**L**
Ptilinopus luteovirens	Golden Dove	H	H	H	**H**
Ptilinopus magnificus	Wompoo Fruit-dove	U	H	L	**L**
Ptilinopus marchei	Flame-breasted Fruit-dove	H	U	H	**L**

Ptilinopus melanospilus	Black-naped Fruit-dove	U	H	H	**L**
Ptilinopus merrilli	Cream-bellied Fruit-dove	H	U	L	**L**
Ptilinopus monacha	Blue-capped Fruit-dove	H	U	H	**L**
Ptilinopus naina	Dwarf Fruit-dove	H	U	L	**L**
Ptilinopus occipitalis	Yellow-breasted Fruit-dove	H	U	L	**L**
Ptilinopus ornatus	Ornate Fruit-dove	U	U	L	**L**
Ptilinopus pelewensis	Palau Fruit-dove	H	H	H	**H**
Ptilinopus perlatus	Pink-spotted Fruit-dove	H	H	L	**L**
Ptilinopus perousii	Many-coloured Fruit-dove	H	H	L	**L**
Ptilinopus porphyraceus	Purple-capped Fruit-dove	H	H	L	**L**
Ptilinopus porphyreus	Pink-headed Fruit-dove	U	U	H	**L**
Ptilinopus pulchellus	Beautiful Fruit-dove	U	H	L	**L**
Ptilinopus purpuratus	Grey-green Fruit-dove	H	H	U	**L**
Ptilinopus rarotongensis	Cook Islands Fruit-dove	H	U	U	**L**
Ptilinopus regina	Rose-crowned Fruit-dove	U	H	H	**L**
Ptilinopus richardsii	Silver-capped Fruit-dove	H	H	U	**L**
Ptilinopus rivoli	White-bibbed Fruit-dove	U	H	H	**L**
Ptilinopus roseicapilla	Mariana Fruit-dove	H	H	H	**H**
Ptilinopus solomonensis	Yellow-bibbed Fruit-dove	H	H	H	**H**
Ptilinopus subgularis	Maroon-chinned Fruit-dove	H	U	H	**L**
Ptilinopus superbus	Superb Fruit-dove	U	H	L	**L**
Ptilinopus tannensis	Tanna Fruit-dove	U	H	U	**L**
Ptilinopus victor	Orange Dove	H	H	H	**H**
Ptilinopus viridis	Claret-breasted Fruit-dove	H	H	L	**L**
Ptilinopus wallacii	Wallace's Fruit-dove	H	U	L	**L**
Ptilocichla falcata	Falcated Wren-babbler	H	H	H	**H**
Ptilocichla leucogrammica	Bornean Wren-babbler	H	H	H	**H**
Ptilocichla mindanensis	Striated Wren-babbler	H	H	H	**H**
Ptilogonys caudatus	Long-tailed Silky-flycatcher	U	H	H	**L**
Ptilogonys cinereus	Grey Silky-flycatcher	L	H	L	**L**
Ptilonorhynchus violaceus	Satin Bowerbird	H	H	L	**L**
Ptilopachus petrosus	Stone Partridge	U	L	H	**L**
Ptiloprora erythropleura	Rufous-sided Honeyeater	H	U	H	**L**
Ptiloprora guisei	Rufous-backed Honeyeater	U	U	H	**L**
Ptiloprora mayri	Mayr's Honeyeater	H	H	H	**H**
Ptiloprora meekiana	Olive-streaked Honeyeater	U	U	H	**L**
Ptiloprora perstriata	Black-backed Honeyeater	U	H	H	**L**
Ptiloprora plumbea	Leaden Honeyeater	U	U	H	**L**
Ptiloris intercedens	Eastern Riflebird	U	H	H	**L**
Ptiloris magnificus	Magnificent Riflebird	U	H	L	**L**
Ptiloris paradiseus	Paradise Riflebird	H	H	H	**H**
Ptiloris victoriae	Victoria's Riflebird	H	H	H	**H**
Ptilorrhoa caerulescens	Blue Jewel-babbler	H	H	L	**L**
Ptilorrhoa castanonota	Chestnut-backed Jewel-babbler	H	H	H	**H**

Ptilorrhoa leucosticta	Spotted Jewel-babbler	H	H	H	**H**
Ptilostomus afer	Piapiac	U	H	H	**L**
Ptychoramphus aleuticus	Cassin's Auklet	H	H	L	**L**
Ptyrticus turdinus	Thrush Babbler	H	U	H	**L**
Pucrasia macrolopha	Koklass Pheasant	H	U	U	**L**
Puffinus assimilis	Little Shearwater	H	H	L	**L**
Puffinus auricularis	Townsend's Shearwater	L	H	L	**L**
Puffinus bulleri	Buller's Shearwater	L	H	L	**L**
Puffinus carneipes	Flesh-footed Shearwater	L	H	L	**L**
Puffinus creatopus	Pink-footed Shearwater	L	H	H	**L**
Puffinus gavia	Fluttering Shearwater	H	H	H	**H**
Puffinus gravis	Great Shearwater	L	H	H	**L**
Puffinus griseus	Sooty Shearwater	U	H	L	**L**
Puffinus heinrothi	Heinroth's Shearwater	H	H	L	**L**
Puffinus huttoni	Hutton's Shearwater	H	H	L	**L**
Puffinus lherminieri	Audubon's Shearwater	L	H	L	**L**
Puffinus mauretanicus	Balearic Shearwater	H	H	H	**H**
Puffinus nativitatis	Christmas Shearwater	L	H	L	**L**
Puffinus newelli	Newell's Shearwater	L	H	H	**L**
Puffinus opisthomelas	Black-vented Shearwater	L	H	H	**L**
Puffinus pacificus	Wedge-tailed Shearwater	L	H	L	**L**
Puffinus puffinus	Manx Shearwater	L	H	H	**L**
Puffinus tenuirostris	Short-tailed Shearwater	L	H	L	**L**
Puffinus yelkouan	Yelkouan Shearwater	H	H	H	**H**
Pulsatrix koeniswaldiana	Tawny-browed Owl	H	H	L	**L**
Pulsatrix melanota	Band-bellied Owl	H	U	H	**L**
Pulsatrix perspicillata	Spectacled Owl	L	H	H	**L**
Purpureicephalus spurius	Red-capped Parrot	H	H	H	**H**
Pycnonotus atriceps	Black-headed Bulbul	U	L	U	**L**
Pycnonotus aurigaster	Sooty-headed Bulbul	U	L	U	**L**
Pycnonotus barbatus	Common Bulbul	U	L	L	**L**
Pycnonotus bimaculatus	Orange-spotted Bulbul	U	U	H	**L**
Pycnonotus blanfordi	Streak-eared Bulbul	U	L	U	**L**
Pycnonotus brunneus	Red-eyed Bulbul	U	H	U	**L**
Pycnonotus cafer	Red-vented Bulbul	U	L	U	**L**
Pycnonotus capensis	Cape Bulbul	H	H	H	**H**
Pycnonotus cyaniventris	Grey-bellied Bulbul	H	L	H	**L**
Pycnonotus erythropthalmos	Spectacled Bulbul	U	H	U	**L**
Pycnonotus eutilotus	Puff-backed Bulbul	H	H	H	**H**
Pycnonotus finlaysoni	Stripe-throated Bulbul	U	L	U	**L**
Pycnonotus flavescens	Flavescent Bulbul	U	L	U	**L**
Pycnonotus goiavier	Yellow-vented Bulbul	U	L	U	**L**
Pycnonotus jocosus	Red-whiskered Bulbul	U	L	H	**L**
Pycnonotus leucogenys	Himalayan Bulbul	U	L	U	**L**

Pycnonotus leucogrammicus	Cream-striped Bulbul	U	U	H	**L**
Pycnonotus leucotis	White-eared Bulbul	U	U	U	**L**
Pycnonotus luteolus	White-browed Bulbul	U	H	U	**L**
Pycnonotus melanicterus	Black-crested Bulbul	U	L	U	**L**
Pycnonotus melanoleucos	Black-and-white Bulbul	H	U	H	**L**
Pycnonotus nieuwenhuisii	Blue-wattled Bulbul	U	U	H	**L**
Pycnonotus nigricans	Black-fronted Bulbul	H	L	H	**L**
Pycnonotus penicillatus	Yellow-eared Bulbul	U	H	H	**L**
Pycnonotus plumosus	Olive-winged Bulbul	U	H	U	**L**
Pycnonotus priocephalus	Grey-headed Bulbul	U	H	H	**L**
Pycnonotus simplex	Cream-vented Bulbul	U	H	U	**L**
Pycnonotus sinensis	Light-vented Bulbul	U	L	U	**L**
Pycnonotus squamatus	Scaly-breasted Bulbul	U	H	H	**L**
Pycnonotus striatus	Striated Bulbul	H	H	U	**L**
Pycnonotus taivanus	Taiwan Bulbul	H	U	H	**L**
Pycnonotus tympanistrigus	Spot-necked Bulbul	U	U	U	**L**
Pycnonotus urostictus	Yellow-wattled Bulbul	U	L	U	**L**
Pycnonotus xantholaemus	Yellow-throated Bulbul	H	L	L	**L**
Pycnonotus xanthopygos	White-spectacled Bulbul	H	L	H	**L**
Pycnonotus xanthorrhous	Brown-breasted Bulbul	U	L	U	**L**
Pycnonotus zeylanicus	Straw-headed Bulbul	H	H	H	**H**
Pycnoptilus floccosus	Pilotbird	H	H	H	**H**
Pycnopygius cinereus	Marbled Honeyeater	U	U	H	**L**
Pycnopygius ixoides	Plain Honeyeater	U	U	L	**L**
Pycnopygius stictocephalus	Streak-headed Honeyeater	U	U	U	**L**
Pygarrhichas albogularis	White-throated Treerunner	H	H	H	**H**
Pygiptila stellaris	Spot-winged Antshrike	H	H	H	**H**
Pygochelidon cyanoleuca	Blue-and-white Swallow	L	L	L	**L**
Pygoscelis adeliae	Adelie Penguin	H	H	L	**L**
Pygoscelis antarcticus	Chinstrap Penguin	H	H	L	**L**
Pygoscelis papua	Gentoo Penguin	H	H	L	**L**
Pyrenestes minor	Lesser Seedcracker	U	U	L	**L**
Pyrenestes ostrinus	Black-bellied Seedcracker	U	L	U	**L**
Pyrenestes sanguineus	Crimson Seedcracker	U	L	U	**L**
Pyriglena atra	Fringe-backed Fire-eye	H	H	H	**H**
Pyriglena leuconota	White-backed Fire-eye	H	H	L	**L**
Pyriglena leucoptera	White-shouldered Fire-eye	H	H	L	**L**
Pyrocephalus rubinus	Vermilion Flycatcher	L	L	L	**L**
Pyroderus scutatus	Red-ruffed Fruitcrow	H	H	L	**L**
Pyrrhocoma ruficeps	Chestnut-headed Tanager	H	U	L	**L**
Pyrrhocorax graculus	Yellow-billed Chough	H	H	L	**L**
Pyrrhocorax pyrrhocorax	Red-billed Chough	H	H	H	**H**

Pyrrholaemus brunneus	Redthroat	U	L	U	**L**
Pyrrhomyias cinnamomeus	Cinnamon Flycatcher	U	H	L	**L**
Pyrrhoplectes epauletta	Gold-naped Finch	H	U	U	**L**
Pyrrhula aurantiaca	Orange Bullfinch	H	U	H	**L**
Pyrrhula erythaca	Grey-headed Bullfinch	U	U	H	**L**
Pyrrhula erythrocephala	Red-headed Bullfinch	U	U	L	**L**
Pyrrhula leucogenis	White-cheeked Bullfinch	H	H	H	**H**
Pyrrhula murina	Azores Bullfinch	H	H	H	**H**
Pyrrhula nipalensis	Brown Bullfinch	U	H	H	**L**
Pyrrhula pyrrhula	Eurasian Bullfinch	H	L	H	**L**
Pyrrhura albipectus	White-necked Parakeet	H	H	H	**H**
Pyrrhura calliptera	Flame-winged Parakeet	H	H	H	**H**
Pyrrhura cruentata	Blue-throated Parakeet	H	H	H	**H**
Pyrrhura devillei	Blaze-winged Parakeet	U	H	L	**L**
Pyrrhura egregia	Fiery-shouldered Parakeet	H	H	H	**H**
Pyrrhura frontalis	Maroon-bellied Parakeet	U	H	L	**L**
Pyrrhura griseipectus	Grey-breasted Parakeet	H	H	L	**L**
Pyrrhura hoematotis	Red-eared Parakeet	H	H	H	**H**
Pyrrhura hoffmanni	Sulphur-winged Parakeet	H	H	H	**H**
Pyrrhura lepida	Pearly Parakeet	H	H	H	**H**
Pyrrhura leucotis	Maroon-faced Parakeet	H	H	H	**H**
Pyrrhura melanura	Maroon-tailed Parakeet	U	H	H	**L**
Pyrrhura molinae	Green-cheeked Parakeet	H	H	H	**H**
Pyrrhura orcesi	El Oro Parakeet	H	H	H	**H**
Pyrrhura perlata	Crimson-bellied Parakeet	H	H	L	**L**
Pyrrhura pfrimeri	Pfrimer's Parakeet	H	H	H	**H**
Pyrrhura picta	Painted Parakeet	H	H	H	**H**
Pyrrhura rhodocephala	Rose-headed Parakeet	H	H	H	**H**
Pyrrhura rupicola	Black-capped Parakeet	H	H	H	**H**
Pyrrhura viridicata	Santa Marta Parakeet	H	H	H	**H**
Pyrrhurus scandens	Leaf-love	H	L	H	**L**
Pytilia afra	Orange-winged Pytilia	U	L	L	**L**
Pytilia hypogrammica	Red-faced Pytilia	U	L	U	**L**
Pytilia melba	Green-winged Pytilia	U	L	L	**L**
Pytilia phoenicoptera	Red-winged Pytilia	U	L	U	**L**
Quelea cardinalis	Cardinal Quelea	U	U	U	**L**
Quelea erythrops	Red-headed Quelea	U	U	U	**L**
Quelea quelea	Red-billed Quelea	U	U	L	**L**
Querula purpurata	Purple-throated Fruitcrow	H	H	H	**H**
Quiscalus lugubris	Carib Grackle	H	L	H	**L**
Quiscalus major	Boat-tailed Grackle	H	L	L	**L**
Quiscalus mexicanus	Great-tailed Grackle	L	L	H	**L**
Quiscalus nicaraguensis	Nicaraguan Grackle	H	L	H	**L**
Quiscalus niger	Greater Antillean Grackle	U	L	H	**L**

Quiscalus quiscula	Common Grackle	H	L	L	**L**
Rallina canningi	Andaman Crake	H	L	L	**L**
Rallina eurizonoides	Slaty-legged Crake	U	L	U	**L**
Rallina fasciata	Red-legged Crake	U	L	U	**L**
Rallina forbesi	Forbes's Forest-rail	H	L	U	**L**
Rallina leucospila	White-striped Forest-rail	H	H	H	**H**
Rallina mayri	Mayr's Forest-rail	H	H	H	**H**
Rallina rubra	Chestnut Forest-rail	H	H	H	**H**
Rallina tricolor	Red-necked Crake	U	L	U	**L**
Rallus antarcticus	Austral Rail	H	L	H	**L**
Rallus aquaticus	Water Rail	H	L	H	**L**
Rallus caerulescens	African Water Rail	U	L	L	**L**
Rallus elegans	King Rail	H	L	L	**L**
Rallus limicola	Virginia Rail	H	L	L	**L**
Rallus longirostris	Clapper Rail	H	H	H	**H**
Rallus madagascariensis	Madagascar Rail	H	U	H	**L**
Rallus semiplumbeus	Bogota Rail	H	H	H	**H**
Rallus wetmorei	Plain-flanked Rail	H	H	H	**H**
Ramphastos ambiguus	Black-mandibled Toucan	H	H	H	**H**
Ramphastos brevis	Choco Toucan	U	H	H	**L**
Ramphastos dicolorus	Red-breasted Toucan	H	H	L	**L**
Ramphastos sulfuratus	Keel-billed Toucan	H	H	H	**H**
Ramphastos swainsonii	Chestnut-mandibled Toucan	H	H	L	**L**
Ramphastos toco	Toco Toucan	U	H	L	**L**
Ramphastos tucanus	White-throated Toucan	H	H	H	**H**
Ramphastos vitellinus	Channel-billed Toucan	H	H	H	**H**
Ramphocaenus melanurus	Long-billed Gnatwren	H	H	H	**H**
Ramphocelus bresilius	Brazilian Tanager	U	L	H	**L**
Ramphocelus carbo	Silver-beaked Tanager	U	H	H	**L**
Ramphocelus costaricensis	Cherrie's Tanager	U	H	H	**L**
Ramphocelus dimidiatus	Crimson-backed Tanager	U	H	U	**L**
Ramphocelus flammigerus	Flame-rumped Tanager	U	H	H	**L**
Ramphocelus melanogaster	Huallaga Tanager	U	U	H	**L**
Ramphocelus nigrogularis	Masked Crimson Tanager	H	U	L	**L**
Ramphocelus passerinii	Scarlet-rumped Tanager	L	H	H	**L**
Ramphocelus sanguinolentus	Crimson-collared Tanager	L	L	H	**L**
Ramphocinclus brachyurus	White-breasted Thrasher	H	H	H	**H**
Ramphodon naevius	Saw-billed Hermit	H	U	L	**L**
Ramphomicron dorsale	Black-backed Thornbill	H	U	H	**L**
Ramphomicron	Purple-backed Thornbill	U	H	H	**L**

microrhynchum					
Ramphotrigon fuscicauda	Dusky-tailed Flatbill	H	H	H	**H**
Ramphotrigon megacephalum	Large-headed Flatbill	H	H	L	**L**
Ramphotrigon ruficauda	Rufous-tailed Flatbill	H	L	H	**L**
Ramsayornis fasciatus	Bar-breasted Honeyeater	U	H	L	**L**
Ramsayornis modestus	Brown-backed Honeyeater	U	H	U	**L**
Randia pseudozosterops	Rand's Warbler	H	H	U	**L**
Recurvirostra americana	American Avocet	L	H	H	**L**
Recurvirostra andina	Andean Avocet	H	H	H	**H**
Recurvirostra avosetta	Pied Avocet	H	H	H	**H**
Recurvirostra novaehollandiae	Red-necked Avocet	H	H	L	**L**
Regulus calendula	Ruby-crowned Kinglet	H	L	L	**L**
Regulus goodfellowi	Flamecrest	H	H	H	**H**
Regulus ignicapilla	Firecrest	H	L	L	**L**
Regulus madeirensis	Madeira Kinglet	H	U	H	**L**
Regulus regulus	Goldcrest	H	L	H	**L**
Regulus satrapa	Golden-crowned Kinglet	H	L	L	**L**
Reinwardtipicus validus	Orange-backed Woodpecker	H	H	H	**H**
Reinwardtoena browni	Pied Cuckoo-dove	H	U	H	**L**
Reinwardtoena crassirostris	Crested Cuckoo-dove	H	H	H	**H**
Reinwardtoena reinwardtsi	Great Cuckoo-dove	H	H	L	**L**
Remiz consobrinus	Chinese Penduline-tit	U	L	U	**L**
Remiz coronatus	White-crowned Penduline-tit	U	L	U	**L**
Remiz pendulinus	Eurasian Penduline-tit	H	L	H	**L**
Rhabdornis grandis	Long-billed Rhabdornis	H	H	H	**H**
Rhabdornis inornatus	Stripe-breasted Rhabdornis	H	H	H	**H**
Rhabdornis mystacalis	Stripe-sided Rhabdornis	H	H	U	**L**
Rhagologus leucostigma	Mottled Whistler	U	H	U	**L**
Rhamphocoris clotbey	Thick-billed Lark	H	L	H	**L**
Rhamphomantis megarhynchus	Long-billed Cuckoo	H	U	L	**L**
Rhaphidura leucopygialis	Silver-rumped Spinetail	H	H	H	**H**
Rhaphidura sabini	Sabine's Spinetail	H	H	L	**L**
Rhea americana	Greater Rhea	U	H	L	**L**
Rhegmatorhina berlepschi	Harlequin Antbird	H	H	H	**H**
Rhegmatorhina cristata	Chestnut-crested Antbird	H	H	H	**H**
Rhegmatorhina gymnops	Bare-eyed Antbird	H	H	H	**H**
Rhegmatorhina hoffmannsi	White-breasted Antbird	H	H	H	**H**
Rhegmatorhina melanosticta	Hairy-crested Antbird	H	H	L	**L**
Rheinardia ocellata	Crested Argus	H	H	L	**L**

Rhinocrypta lanceolata	Crested Gallito	H	H	H	**H**
Rhinomyias additus	Streaky-breasted Jungle-flycatcher	H	L	H	**L**
Rhinomyias albigularis	White-throated Jungle-flycatcher	H	H	H	**H**
Rhinomyias brunneatus	Brown-chested Jungle-flycatcher	H	L	L	**L**
Rhinomyias colonus	Henna-tailed Jungle-flycatcher	H	L	H	**L**
Rhinomyias goodfellowi	Slaty-backed Jungle-flycatcher	H	H	H	**H**
Rhinomyias gularis	Eyebrowed Jungle-flycatcher	H	H	H	**H**
Rhinomyias insignis	White-browed Jungle-flycatcher	H	H	H	**H**
Rhinomyias olivaceus	Fulvous-chested Jungle-flycatcher	U	L	U	**L**
Rhinomyias oscillans	Russet-backed Jungle-flycatcher	H	L	H	**L**
Rhinomyias ruficauda	Rufous-tailed Jungle-flycatcher	H	H	U	**L**
Rhinomyias umbratilis	Grey-chested Jungle-flycatcher	H	L	H	**L**
Rhinoplax vigil	Helmeted Hornbill	H	H	H	**H**
Rhinopomastus aterrimus	Black Scimitarbill	H	H	U	**L**
Rhinopomastus cyanomelas	Common Scimitarbill	H	H	L	**L**
Rhinopomastus minor	Abyssinian Scimitarbill	H	H	U	**L**
Rhinoptilus africanus	Double-banded Courser	U	H	U	**L**
Rhinoptilus bitorquatus	Jerdon's Courser	H	H	H	**H**
Rhinoptilus chalcopterus	Bronze-winged Courser	U	H	U	**L**
Rhinoptilus cinctus	Three-banded Courser	U	H	U	**L**
Rhipidura albicollis	White-throated Fantail	U	L	H	**L**
Rhipidura albolimbata	Friendly Fantail	U	U	U	**L**
Rhipidura atra	Black Fantail	U	H	U	**L**
Rhipidura aureola	White-browed Fantail	U	L	L	**L**
Rhipidura brachyrhyncha	Dimorphic Fantail	H	U	U	**L**
Rhipidura cockerelli	White-winged Fantail	U	U	U	**L**
Rhipidura cyaniceps	Blue-headed Fantail	U	H	U	**L**
Rhipidura dahli	Bismarck Fantail	H	U	H	**L**
Rhipidura dedemi	Streaky-breasted Fantail	H	U	H	**L**
Rhipidura diluta	Brown-capped Fantail	H	U	H	**L**
Rhipidura drownei	Brown Fantail	H	H	H	**H**
Rhipidura euryura	White-bellied Fantail	H	U	H	**L**
Rhipidura fuliginosa	Grey Fantail	H	H	L	**L**
Rhipidura fuscorufa	Cinnamon-tailed Fantail	H	U	H	**L**
Rhipidura hyperythra	Chestnut-bellied Fantail	H	H	U	**L**
Rhipidura hypoxantha	Yellow-bellied Fantail	H	L	U	**L**

Rhipidura javanica	Pied Fantail	U	H	U	**L**
Rhipidura kubaryi	Pohnpei Fantail	H	U	H	**L**
Rhipidura lepida	Palau Fantail	H	H	H	**H**
Rhipidura leucophrys	Willie-wagtail	U	L	L	**L**
Rhipidura leucothorax	White-bellied Thicket-fantail	U	H	U	**L**
Rhipidura maculipectus	Black Thicket-fantail	U	U	U	**L**
Rhipidura malaitae	Malaita Fantail	H	H	H	**H**
Rhipidura matthiae	Matthias Fantail	H	U	H	**L**
Rhipidura nebulosa	Samoan Fantail	H	H	U	**L**
Rhipidura nigrocinnamomea	Black-and-cinnamon Fantail	H	U	H	**L**
Rhipidura opistherythra	Long-tailed Fantail	H	U	H	**L**
Rhipidura perlata	Spotted Fantail	U	H	U	**L**
Rhipidura personata	Kadavu Fantail	H	U	L	**L**
Rhipidura phasiana	Mangrove Fantail	U	H	U	**L**
Rhipidura phoenicura	Rufous-tailed Fantail	H	U	H	**L**
Rhipidura rennelliana	Rennell Fantail	H	H	H	**H**
Rhipidura rufidorsa	Rufous-backed Fantail	H	H	U	**L**
Rhipidura rufifrons	Rufous Fantail	U	L	U	**L**
Rhipidura rufiventris	Northern Fantail	U	H	U	**L**
Rhipidura semirubra	Manus Fantail	H	U	H	**L**
Rhipidura superciliaris	Blue Fantail	H	U	U	**L**
Rhipidura superflua	Tawny-backed Fantail	H	U	H	**L**
Rhipidura tenebrosa	Dusky Fantail	H	H	H	**H**
Rhipidura teysmanni	Rusty-bellied Fantail	H	U	H	**L**
Rhipidura threnothorax	Sooty Thicket-fantail	H	U	U	**L**
Rhipidura verreauxi	Streaked Fantail	U	H	L	**L**
Rhizothera longirostris	Long-billed Partridge	H	L	H	**L**
Rhodinocichla rosea	Rosy Thrush-tanager	L	L	L	**L**
Rhodonessa caryophyllacea	Pink-headed Duck	H	H	H	**H**
Rhodopechys mongolicus	Mongolian Finch	H	L	H	**L**
Rhodopechys obsoletus	Desert Finch	H	L	H	**L**
Rhodopechys sanguineus	Crimson-winged Finch	H	L	L	**L**
Rhodophoneus cruentus	Rosy-patched Bush-shrike	U	L	U	**L**
Rhodopis vesper	Oasis Hummingbird	H	H	H	**H**
Rhodospingus cruentus	Crimson-breasted Finch	H	U	H	**L**
Rhodostethia rosea	Ross's Gull	H	H	L	**L**
Rhodothraupis celaeno	Crimson-collared Grosbeak	L	L	H	**L**
Rhopocichla atriceps	Dark-fronted Babbler	U	H	U	**L**
Rhopophilus pekinensis	White-browed Chinese Warbler	U	L	U	**L**
Rhopornis ardesiacus	Slender Antbird	H	H	L	**L**
Rhyacornis bicolor	Luzon Water-redstart	H	L	H	**L**
Rhyacornis fuliginosa	Plumbeous Water-redstart	U	L	U	**L**

Rhynchocyclus brevirostris	Eye-ringed Flatbill	H	H	H	**H**
Rhynchocyclus fulvipectus	Fulvous-breasted Flatbill	H	H	H	**H**
Rhynchocyclus olivaceus	Olivaceous Flatbill	H	H	H	**H**
Rhynchocyclus pacificus	Pacific Flatbill	H	H	H	**H**
Rhynchopsitta pachyrhyncha	Thick-billed Parrot	H	H	L	**L**
Rhynchopsitta terrisi	Maroon-fronted Parrot	H	H	H	**H**
Rhynchortyx cinctus	Tawny-faced Quail	H	U	H	**L**
Rhynchostruthus louisae	Somali Grosbeak	H	U	L	**L**
Rhynchostruthus percivali	Arabian Grosbeak	H	U	H	**L**
Rhynchostruthus socotranus	Socotra Grosbeak	H	H	H	**H**
Rhynchotus maculicollis	Huayco Tinamou	U	H	H	**L**
Rhynchotus rufescens	Red-winged Tinamou	U	H	L	**L**
Rhynochetos jubatus	Kagu	H	H	H	**H**
Rhytipterna holerythra	Rufous Mourner	H	H	H	**H**
Rhytipterna immunda	Pale-bellied Mourner	H	U	H	**L**
Rhytipterna simplex	Greyish Mourner	H	H	H	**H**
Rimator albostriatus	Sumatran Wren-babbler	H	H	U	**L**
Rimator malacoptilus	Long-billed Wren-babbler	H	H	U	**L**
Rimator pasquieri	White-throated Wren-babbler	H	H	U	**L**
Riparia cincta	Banded Martin	U	L	U	**L**
Riparia congica	Congo Martin	H	U	H	**L**
Riparia paludicola	Plain Martin	U	L	L	**L**
Riparia riparia	Sand Martin	L	L	H	**L**
Rissa brevirostris	Red-legged Kittiwake	H	H	L	**L**
Rissa tridactyla	Black-legged Kittiwake	H	H	L	**L**
Robsonius rabori	Rusty-faced Babbler	H	H	H	**H**
Rollandia microptera	Titicaca Grebe	H	H	H	**H**
Rollandia rolland	White-tufted Grebe	H	H	H	**H**
Rollulus rouloul	Crested Partridge	H	L	H	**L**
Roraimia adusta	Roraiman Barbtail	H	H	H	**H**
Rostratula benghalensis	Greater Painted-snipe	L	H	L	**L**
Rostratula semicollaris	South American Painted-snipe	H	H	L	**L**
Rostrhamus sociabilis	Snail Kite	L	H	L	**L**
Rougetius rougetii	Rouget's Rail	H	L	L	**L**
Rowettia goughensis	Gough Bunting	H	H	U	**L**
Rukia longirostra	Long-billed White-eye	H	H	H	**H**
Rukia ruki	Faichuk White-eye	H	H	U	**L**
Rupicola peruvianus	Andean Cock-of-the-rock	H	U	L	**L**
Rupicola rupicola	Guianan Cock-of-the-rock	H	H	H	**H**
Ruwenzorornis johnstoni	Ruwenzori Turaco	H	H	H	**H**
Rynchops albicollis	Indian Skimmer	H	H	H	**H**

Rynchops flavirostris	African Skimmer	L	H	L	**L**
Rynchops niger	Black Skimmer	L	H	L	**L**
Sagittarius serpentarius	Secretarybird	L	H	L	**L**
Sakesphorus bernardi	Collared Antshrike	U	L	H	**L**
Sakesphorus canadensis	Black-crested Antshrike	H	L	H	**L**
Sakesphorus cristatus	Silvery-cheeked Antshrike	U	U	L	**L**
Sakesphorus luctuosus	Glossy Antshrike	H	H	H	**H**
Sakesphorus melanonotus	Black-backed Antshrike	U	H	H	**L**
Sakesphorus melanothorax	Band-tailed Antshrike	H	U	H	**L**
Salpinctes obsoletus	Rock Wren	H	L	H	**L**
Salpornis spilonotus	Spotted Treecreeper	U	L	L	**L**
Saltator albicollis	Lesser Antillean Saltator	H	L	H	**L**
Saltator atriceps	Black-headed Saltator	L	H	H	**L**
Saltator atricollis	Black-throated Saltator	U	U	L	**L**
Saltator atripennis	Black-winged Saltator	U	U	H	**L**
Saltator aurantiirostris	Golden-billed Saltator	H	U	L	**L**
Saltator cinctus	Masked Saltator	H	H	H	**H**
Saltator coerulescens	Greyish Saltator	L	L	H	**L**
Saltator fuliginosus	Black-throated Grosbeak	H	H	L	**L**
Saltator grossus	Slate-coloured Grosbeak	H	U	H	**L**
Saltator maxillosus	Thick-billed Saltator	H	H	L	**L**
Saltator maximus	Buff-throated Saltator	H	H	H	**H**
Saltator nigriceps	Black-cowled Saltator	U	U	H	**L**
Saltator orenocensis	Orinoco Saltator	H	U	H	**L**
Saltator rufiventris	Rufous-bellied Saltator	H	U	H	**L**
Saltator similis	Green-winged Saltator	U	U	L	**L**
Saltator striatipectus	Streaked Saltator	U	H	H	**L**
Saltatricula multicolor	Many-coloured Chaco-finch	H	U	L	**L**
Salvadorina waigiuensis	Salvadori's Teal	H	H	H	**H**
Sapayoa aenigma	Broad-billed Sapayoa	H	U	L	**L**
Sappho sparganura	Red-tailed Comet	H	H	H	**H**
Sarcogyps calvus	Red-headed Vulture	H	H	L	**L**
Sarcops calvus	Coleto	U	U	U	**L**
Sarcoramphus papa	King Vulture	H	H	L	**L**
Sarkidiornis melanotos	Comb Duck	L	L	L	**L**
Saroglossa aurata	Madagascar Starling	U	U	U	**L**
Saroglossa spiloptera	Spot-winged Starling	U	L	U	**L**
Sarothrura affinis	Striped Flufftail	H	L	L	**L**
Sarothrura ayresi	White-winged Flufftail	H	L	H	**L**
Sarothrura boehmi	Streaky-breasted Flufftail	H	L	H	**L**
Sarothrura elegans	Buff-spotted Flufftail	U	L	L	**L**
Sarothrura insularis	Madagascar Flufftail	L	L	H	**L**
Sarothrura lugens	Chestnut-headed Flufftail	H	U	L	**L**
Sarothrura pulchra	White-spotted Flufftail	H	H	L	**L**

Sarothrura rufa	Red-chested Flufftail	U	L	L	**L**
Sarothrura watersi	Slender-billed Flufftail	H	U	H	**L**
Sasia abnormis	Rufous Piculet	H	L	H	**L**
Sasia africana	African Piculet	H	H	H	**H**
Sasia ochracea	White-browed Piculet	H	L	H	**L**
Satrapa icterophrys	Yellow-browed Tyrant	U	L	L	**L**
Saxicola caprata	Pied Bushchat	U	L	H	**L**
Saxicola dacotiae	Fuerteventura Stonechat	H	L	H	**L**
Saxicola ferreus	Grey Bushchat	U	L	U	**L**
Saxicola gutturalis	White-bellied Bushchat	H	U	L	**L**
Saxicola insignis	White-throated Bushchat	H	L	H	**L**
Saxicola jerdoni	Jerdon's Bushchat	U	L	U	**L**
Saxicola leucurus	White-tailed Stonechat	U	L	U	**L**
Saxicola macrorhynchus	White-browed Bushchat	H	U	H	**L**
Saxicola rubetra	Whinchat	H	L	H	**L**
Saxicola tectes	Reunion Stonechat	U	U	H	**L**
Saxicola torquatus	Common Stonechat	H	L	H	**L**
Saxicoloides fulicatus	Indian Robin	U	U	L	**L**
Sayornis nigricans	Black Phoebe	L	L	L	**L**
Sayornis phoebe	Eastern Phoebe	H	L	L	**L**
Sayornis saya	Say's Phoebe	H	L	H	**L**
Scelorchilus albicollis	White-throated Tapaculo	H	L	H	**L**
Scelorchilus rubecula	Chucao Tapaculo	H	H	L	**L**
Scenopoeetes dentirostris	Tooth-billed Bowerbird	H	H	H	**H**
Schetba rufa	Rufous Vanga	H	H	L	**L**
Schiffornis major	Greater Schiffornis	H	H	U	**L**
Schiffornis turdina	Thrush-like Schiffornis	H	H	H	**H**
Schiffornis virescens	Greenish Schiffornis	H	H	U	**L**
Schistes geoffroyi	Wedge-billed Hummingbird	H	H	L	**L**
Schistochlamys melanopis	Black-faced Tanager	U	H	L	**L**
Schistochlamys ruficapillus	Cinnamon Tanager	U	H	L	**L**
Schistocichla caurensis	Caura Antbird	H	H	H	**H**
Schistocichla leucostigma	Spot-winged Antbird	H	H	U	**L**
Schistocichla saturata	Roraiman Antbird	H	H	H	**H**
Schistocichla schistacea	Slate-coloured Antbird	H	H	L	**L**
Schizoeaca coryi	Ochre-browed Thistletail	H	H	H	**H**
Schizoeaca fuliginosa	White-chinned Thistletail	H	H	H	**H**
Schizoeaca griseomurina	Mouse-coloured Thistletail	H	U	H	**L**
Schizoeaca harterti	Black-throated Thistletail	H	H	H	**H**
Schizoeaca helleri	Puna Thistletail	H	U	H	**L**
Schizoeaca palpebralis	Eye-ringed Thistletail	H	U	H	**L**
Schizoeaca perijana	Perija Thistletail	H	H	H	**H**
Schizoeaca vilcabambae	Vilcabamba Thistletail	H	U	H	**L**
Schoenicola brevirostris	Fan-tailed Grassbird	U	L	U	**L**

Schoenicola platyurus	Broad-tailed Grassbird	H	L	H	**L**
Schoeniophylax phryganophilus	Chotoy Spinetail	H	L	L	**L**
Schoutedenapus myoptilus	Scarce Swift	H	U	H	**L**
Schoutedenapus schoutedeni	Schouteden's Swift	H	U	H	**L**
Scissirostrum dubium	Finch-billed Myna	H	H	U	**L**
Sclateria naevia	Silvered Antbird	H	H	H	**H**
Sclerurus albigularis	Grey-throated Leaftosser	H	H	H	**H**
Sclerurus caudacutus	Black-tailed Leaftosser	H	H	H	**H**
Sclerurus guatemalensis	Scaly-throated Leaftosser	H	H	H	**H**
Sclerurus mexicanus	Tawny-throated Leaftosser	H	H	H	**H**
Sclerurus rufigularis	Short-billed Leaftosser	H	H	H	**H**
Sclerurus scansor	Rufous-breasted Leaftosser	H	H	L	**L**
Scolopax bukidnonensis	Philippine Woodcock	H	H	U	**L**
Scolopax celebensis	Sulawesi Woodcock	H	H	H	**H**
Scolopax minor	American Woodcock	H	H	L	**L**
Scolopax mira	Ryukyu Woodcock	H	H	H	**H**
Scolopax rochussenii	Moluccan Woodcock	H	H	H	**H**
Scolopax rosenbergii	New Guinea Woodcock	U	H	U	**L**
Scolopax rusticola	Eurasian Woodcock	H	H	H	**H**
Scolopax saturata	Javan Woodcock	H	H	H	**H**
Scopus umbretta	Hamerkop	L	H	L	**L**
Scotocerca inquieta	Streaked Scrub-warbler	H	L	H	**L**
Scotopelia bouvieri	Vermiculated Fishing-owl	H	H	H	**H**
Scotopelia peli	Pel's Fishing-owl	U	H	L	**L**
Scotopelia ussheri	Rufous Fishing-owl	H	H	L	**L**
Scytalopus acutirostris	Tschudi's Tapaculo	H	H	H	**H**
Scytalopus affinis	Ancash Tapaculo	H	U	H	**L**
Scytalopus altirostris	Neblina Tapaculo	H	H	H	**H**
Scytalopus argentifrons	Silvery-fronted Tapaculo	U	H	H	**L**
Scytalopus atratus	White-crowned Tapaculo	H	H	H	**H**
Scytalopus bolivianus	Bolivian Tapaculo	H	H	H	**H**
Scytalopus canus	Paramo Tapaculo	H	H	H	**H**
Scytalopus caracae	Caracas Tapaculo	H	H	H	**H**
Scytalopus chocoensis	Choco Tapaculo	H	H	H	**H**
Scytalopus femoralis	Rufous-vented Tapaculo	H	H	H	**H**
Scytalopus fuscus	Dusky Tapaculo	H	H	L	**L**
Scytalopus griseicollis	Matorral Tapaculo	H	H	H	**H**
Scytalopus indigoticus	White-breasted Tapaculo	U	H	U	**L**
Scytalopus iraiensis	Marsh Tapaculo	H	H	H	**H**
Scytalopus latebricola	Brown-rumped Tapaculo	H	H	H	**H**
Scytalopus latrans	Blackish Tapaculo	H	U	H	**L**
Scytalopus macropus	Large-footed Tapaculo	H	H	H	**H**

Scytalopus magellanicus	Magellanic Tapaculo	U	L	H	**L**
Scytalopus meridanus	Merida Tapaculo	H	H	U	**L**
Scytalopus micropterus	Long-tailed Tapaculo	U	U	H	**L**
Scytalopus novacapitalis	Brasilia Tapaculo	H	H	U	**L**
Scytalopus pachecoi	Planalto Tapaculo	H	H	L	**L**
Scytalopus panamensis	Tacarcuna Tapaculo	H	H	H	**H**
Scytalopus parkeri	Chusquea Tapaculo	H	H	H	**H**
Scytalopus parvirostris	Trilling Tapaculo	H	H	H	**H**
Scytalopus psychopompus	Bahia Tapaculo	H	H	U	**L**
Scytalopus robbinsi	Ecuadorian Tapaculo	H	H	H	**H**
Scytalopus rodriguezi	Upper Magdalena Tapaculo	H	H	H	**H**
Scytalopus sanctaemartae	Santa Marta Tapaculo	H	H	H	**H**
Scytalopus schulenbergi	Diademed Tapaculo	H	H	H	**H**
Scytalopus simonsi	Puna Tapaculo	U	H	H	**L**
Scytalopus speluncae	Mouse-coloured Tapaculo	H	H	L	**L**
Scytalopus spillmanni	Spillmann's Tapaculo	H	H	H	**H**
Scytalopus stilesi	Stiles's Tapaculo	H	H	H	**H**
Scytalopus superciliaris	White-browed Tapaculo	H	H	H	**H**
Scytalopus unicolor	Unicoloured Tapaculo	H	H	H	**H**
Scytalopus urubambae	Vilcabamba Tapaculo	H	H	H	**H**
Scytalopus vicinior	Narino Tapaculo	H	H	H	**H**
Scytalopus zimmeri	Zimmer's Tapaculo	H	U	H	**L**
Scythrops novaehollandiae	Channel-billed Cuckoo	U	L	H	**L**
Seicercus affinis	White-spectacled Warbler	H	H	U	**L**
Seicercus burkii	Green-crowned Warbler	H	U	U	**L**
Seicercus castaniceps	Chestnut-crowned Warbler	H	H	U	**L**
Seicercus grammiceps	Sunda Warbler	H	H	H	**H**
Seicercus montis	Yellow-breasted Warbler	H	H	H	**H**
Seicercus omeiensis	Martens's Warbler	H	U	U	**L**
Seicercus poliogenys	Grey-cheeked Warbler	H	H	U	**L**
Seicercus soror	Alstrom's Warbler	H	U	U	**L**
Seicercus tephrocephalus	Grey-crowned Warbler	H	U	U	**L**
Seicercus valentini	Bianchi's Warbler	H	U	U	**L**
Seicercus whistleri	Whistler's Warbler	H	H	U	**L**
Seiurus aurocapilla	Ovenbird	H	L	L	**L**
Seiurus motacilla	Louisiana Waterthrush	L	L	L	**L**
Seiurus noveboracensis	Northern Waterthrush	H	L	L	**L**
Selasphorus ardens	Glow-throated Hummingbird	H	U	H	**L**
Selasphorus flammula	Volcano Hummingbird	U	U	H	**L**
Selasphorus platycercus	Broad-tailed Hummingbird	H	H	L	**L**
Selasphorus rufus	Rufous Hummingbird	H	H	H	**H**
Selasphorus sasin	Allen's Hummingbird	H	H	H	**H**
Selasphorus scintilla	Scintillant Hummingbird	U	U	H	**L**
Selenidera gouldii	Gould's Toucanet	H	H	H	**H**

Selenidera maculirostris	Spot-billed Toucanet	H	H	L	**L**
Selenidera nattereri	Tawny-tufted Toucanet	H	H	H	**H**
Selenidera piperivora	Guianan Toucanet	H	H	H	**H**
Selenidera reinwardtii	Golden-collared Toucanet	H	H	L	**L**
Selenidera spectabilis	Yellow-eared Toucanet	H	H	H	**H**
Seleucidis melanoleucus	Twelve-wired Bird-of-paradise	H	H	L	**L**
Semioptera wallacii	Standardwing	H	H	H	**H**
Semnornis frantzii	Prong-billed Barbet	H	H	H	**H**
Semnornis ramphastinus	Toucan Barbet	H	H	H	**H**
Sephanoides fernandensis	Juan Fernandez Firecrown	H	H	H	**H**
Sephanoides sephaniodes	Green-backed Firecrown	U	H	L	**L**
Sericornis arfakianus	Grey-green Scrubwren	U	H	H	**L**
Sericornis beccarii	Beccari's Scrubwren	U	H	H	**L**
Sericornis citreogularis	Yellow-throated Scrubwren	H	H	L	**L**
Sericornis frontalis	White-browed Scrubwren	H	H	L	**L**
Sericornis humilis	Tasmanian Scrubwren	H	H	H	**H**
Sericornis keri	Atherton Scrubwren	H	H	H	**H**
Sericornis magnirostra	Large-billed Scrubwren	H	H	L	**L**
Sericornis nouhuysi	Large Scrubwren	U	H	H	**L**
Sericornis papuensis	Papuan Scrubwren	U	H	H	**L**
Sericornis perspicillatus	Buff-faced Scrubwren	U	H	H	**L**
Sericornis rufescens	Vogelkop Scrubwren	H	H	H	**H**
Sericornis spilodera	Pale-billed Scrubwren	H	H	L	**L**
Sericornis virgatus	Perplexing Scrubwren	H	H	L	**L**
Sericossypha albocristata	White-capped Tanager	H	U	L	**L**
Sericulus aureus	Flame Bowerbird	U	H	U	**L**
Sericulus bakeri	Fire-maned Bowerbird	H	H	H	**H**
Sericulus chrysocephalus	Regent Bowerbird	H	H	H	**H**
Serilophus lunatus	Silver-breasted Broadbill	U	L	L	**L**
Serinus alario	Black-headed Canary	U	H	U	**L**
Serinus albogularis	White-throated Canary	H	U	L	**L**
Serinus ankoberensis	Ankober Serin	H	H	L	**L**
Serinus atrogularis	Black-throated Seedeater	U	U	U	**L**
Serinus buchanani	Kenya Grosbeak-canary	H	U	U	**L**
Serinus burtoni	Thick-billed Seedeater	U	U	L	**L**
Serinus canaria	Island Canary	H	L	H	**L**
Serinus canicollis	Cape Canary	U	U	U	**L**
Serinus capistratus	Black-faced Canary	U	U	L	**L**
Serinus citrinelloides	Abyssinian Citril	U	U	L	**L**
Serinus citrinipectus	Lemon-breasted Seedeater	U	U	L	**L**
Serinus donaldsoni	Ethiopian Grosbeak-canary	U	U	U	**L**
Serinus dorsostriatus	White-bellied Canary	H	U	L	**L**
Serinus estherae	Mountain Serin	U	U	H	**L**
Serinus flavigula	Yellow-throated Seedeater	H	U	H	**L**

Serinus flaviventris	Yellow Canary	H	U	L	**L**
Serinus frontalis	Yellow-browed Citril	U	U	U	**L**
Serinus gularis	Streaky-headed Seedeater	U	U	U	**L**
Serinus hypostictus	Grey-faced Citril	U	U	U	**L**
Serinus koliensis	Papyrus Canary	H	U	H	**L**
Serinus leucopterus	Protea Canary	H	H	H	**H**
Serinus leucopygius	White-rumped Seedeater	U	U	U	**L**
Serinus melanochrous	Kipengere Seedeater	U	H	L	**L**
Serinus menachensis	Yemen Serin	H	U	H	**L**
Serinus mennelli	Black-eared Seedeater	U	U	L	**L**
Serinus mozambicus	Yellow-fronted Canary	U	U	U	**L**
Serinus nigriceps	Ethiopian Siskin	U	H	U	**L**
Serinus pusillus	Fire-fronted Serin	H	L	L	**L**
Serinus reichardi	Reichard's Seedeater	U	U	U	**L**
Serinus rothschildi	Olive-rumped Serin	U	H	U	**L**
Serinus rufobrunneus	Principe Seedeater	H	U	L	**L**
Serinus scotops	Forest Canary	H	H	L	**L**
Serinus serinus	European Serin	H	L	L	**L**
Serinus striolatus	Streaky Seedeater	U	U	L	**L**
Serinus sulphuratus	Brimstone Canary	U	U	U	**L**
Serinus symonsi	Drakensberg Siskin	H	H	L	**L**
Serinus syriacus	Syrian Serin	H	L	H	**L**
Serinus thibetanus	Tibetan Serin	U	U	U	**L**
Serinus totta	Cape Siskin	H	H	H	**H**
Serinus tristriatus	Brown-rumped Seedeater	U	U	U	**L**
Serinus whytii	Yellow-browed Seedeater	H	H	L	**L**
Serinus xantholaemus	Salvadori's Serin	H	H	H	**H**
Serinus xanthopygius	Yellow-rumped Seedeater	H	U	U	**L**
Serpophaga cinerea	Torrent Tyrannulet	H	H	L	**L**
Serpophaga hypoleuca	River Tyrannulet	H	H	H	**H**
Serpophaga munda	White-bellied Tyrannulet	H	L	L	**L**
Serpophaga nigricans	Sooty Tyrannulet	H	L	L	**L**
Serpophaga subcristata	White-crested Tyrannulet	U	L	L	**L**
Setophaga ruticilla	American Redstart	L	L	L	**L**
Setornis criniger	Hook-billed Bulbul	H	U	H	**L**
Sheppardia aequatorialis	Equatorial Akalat	H	H	L	**L**
Sheppardia aurantiithorax	Rubeho Akalat	H	H	H	**H**
Sheppardia bocagei	Bocage's Akalat	U	H	U	**L**
Sheppardia cyornithopsis	Lowland Akalat	H	H	L	**L**
Sheppardia gabela	Gabela Akalat	H	H	H	**H**
Sheppardia gunningi	East Coast Akalat	H	U	L	**L**
Sheppardia lowei	Iringa Akalat	H	H	L	**L**
Sheppardia montana	Usambara Akalat	H	H	H	**H**
Sheppardia sharpei	Sharpe's Akalat	U	H	L	**L**
Sialia currucoides	Mountain Bluebird	H	L	H	**L**

Sialia mexicana	Western Bluebird	H	L	H	**L**
Sialia sialis	Eastern Bluebird	H	L	L	**L**
Sicalis auriventris	Greater Yellow-finch	H	U	H	**L**
Sicalis citrina	Stripe-tailed Yellow-finch	U	U	L	**L**
Sicalis columbiana	Orange-fronted Yellow-finch	H	U	H	**L**
Sicalis flaveola	Saffron Finch	U	L	L	**L**
Sicalis lebruni	Patagonian Yellow-finch	H	U	H	**L**
Sicalis lutea	Puna Yellow-finch	H	U	H	**L**
Sicalis luteiventris	Misto Yellow-finch	U	L	U	**L**
Sicalis luteocephala	Citron-headed Yellow-finch	H	U	H	**L**
Sicalis luteola	Grassland Yellow-finch	L	L	L	**L**
Sicalis olivascens	Greenish Yellow-finch	H	U	H	**L**
Sicalis raimondii	Raimondi's Yellow-finch	H	U	H	**L**
Sicalis taczanowskii	Sulphur-throated Finch	H	U	H	**L**
Sicalis uropygialis	Bright-rumped Yellow-finch	H	U	H	**L**
Sigelus silens	Fiscal Flycatcher	H	L	H	**L**
Simoxenops striatus	Bolivian Recurvebill	H	H	H	**H**
Simoxenops ucayalae	Peruvian Recurvebill	H	H	H	**H**
Siphonorhis americana	Jamaican Pauraque	H	U	H	**L**
Siphonorhis brewsteri	Least Pauraque	H	U	H	**L**
Sipodotus wallacii	Wallace's Fairywren	H	H	L	**L**
Siptornis striaticollis	Spectacled Prickletail	H	H	H	**H**
Siptornopsis hypochondriaca	Great Spinetail	H	U	H	**L**
Sirystes sibilator	Sirystes	H	U	H	**L**
Sitta azurea	Blue Nuthatch	H	H	U	**L**
Sitta canadensis	Red-breasted Nuthatch	H	L	L	**L**
Sitta carolinensis	White-breasted Nuthatch	H	L	L	**L**
Sitta cashmirensis	Kashmir Nuthatch	H	H	H	**H**
Sitta castanea	Chestnut-bellied Nuthatch	U	L	U	**L**
Sitta europaea	Wood Nuthatch	H	H	H	**H**
Sitta formosa	Beautiful Nuthatch	H	H	H	**H**
Sitta frontalis	Velvet-fronted Nuthatch	H	H	U	**L**
Sitta himalayensis	White-tailed Nuthatch	H	H	U	**L**
Sitta krueperi	Krueper's Nuthatch	H	H	L	**L**
Sitta ledanti	Algerian Nuthatch	H	H	H	**H**
Sitta leucopsis	White-cheeked Nuthatch	H	H	U	**L**
Sitta magna	Giant Nuthatch	H	H	H	**H**
Sitta nagaensis	Chestnut-vented Nuthatch	H	H	U	**L**
Sitta neumayer	Western Rock-nuthatch	H	L	L	**L**
Sitta oenochlamys	Sulphur-billed Nuthatch	H	H	U	**L**
Sitta pusilla	Brown-headed Nuthatch	H	L	L	**L**
Sitta pygmaea	Pygmy Nuthatch	H	H	L	**L**
Sitta solangiae	Yellow-billed Nuthatch	H	H	L	**L**
Sitta tephronota	Eastern Rock-nuthatch	H	L	H	**L**

Sitta victoriae	White-browed Nuthatch	H	H	H	**H**
Sitta villosa	Snowy-browed Nuthatch	H	H	U	**L**
Sitta whiteheadi	Corsican Nuthatch	H	H	L	**L**
Sitta yunnanensis	Yunnan Nuthatch	H	H	H	**H**
Sittasomus griseicapillus	Olivaceous Woodcreeper	H	L	L	**L**
Skutchia borbae	Pale-faced Antbird	H	H	H	**H**
Smicrornis brevirostris	Weebill	H	L	L	**L**
Smithornis capensis	African Broadbill	U	L	L	**L**
Smithornis rufolateralis	Rufous-sided Broadbill	H	H	L	**L**
Smithornis sharpei	Grey-headed Broadbill	H	H	H	**H**
Snowornis cryptolophus	Olivaceous Piha	H	U	H	**L**
Snowornis subalaris	Grey-tailed Piha	H	U	H	**L**
Somateria fischeri	Spectacled Eider	H	H	H	**H**
Somateria mollissima	Common Eider	H	H	H	**H**
Somateria spectabilis	King Eider	H	H	H	**H**
Spartonoica maluroides	Bay-capped Wren-spinetail	H	L	H	**L**
Speculanas specularis	Spectacled Duck	H	H	H	**H**
Speculipastor bicolor	Magpie Starling	U	L	U	**L**
Speirops brunneus	Fernando Po Speirops	H	H	H	**H**
Speirops leucophoeus	Principe Speirops	H	H	L	**L**
Speirops lugubris	Black-capped Speirops	H	H	L	**L**
Speirops melanocephalus	Mount Cameroon Speirops	H	H	H	**H**
Spelaeornis badeigularis	Rusty-throated Wren-babbler	H	H	H	**H**
Spelaeornis caudatus	Rufous-throated Wren-babbler	H	H	H	**H**
Spelaeornis chocolatinus	Long-tailed Wren-babbler	H	H	L	**L**
Spelaeornis formosus	Spotted Wren-babbler	H	H	U	**L**
Spelaeornis kinneari	Pale-throated Wren-babbler	H	H	U	**L**
Spelaeornis longicaudatus	Tawny-breasted Wren-babbler	H	H	H	**H**
Spelaeornis oatesi	Chin Hills Wren-babbler	H	H	U	**L**
Spelaeornis reptatus	Grey-bellied Wren-babbler	H	H	L	**L**
Spelaeornis troglodytoides	Bar-winged Wren-babbler	H	H	U	**L**
Spermophaga haematina	Western Bluebill	H	L	L	**L**
Spermophaga poliogenys	Grant's Bluebill	H	U	U	**L**
Spermophaga ruficapilla	Red-headed Bluebill	U	L	H	**L**
Sphecotheres hypoleucus	Wetar Figbird	H	U	L	**L**
Sphecotheres vieilloti	Australasian Figbird	U	H	L	**L**
Sphecotheres viridis	Timor Figbird	H	L	L	**L**
Spheniscus demersus	African Penguin	H	H	H	**H**
Spheniscus humboldti	Humboldt Penguin	H	H	H	**H**
Spheniscus magellanicus	Magellanic Penguin	H	H	H	**H**
Spheniscus mendiculus	Galapagos Penguin	H	H	H	**H**

Sphenocichla humei	Blackish-breasted Babbler	H	H	H	**H**
Sphenocichla roberti	Chevron-breasted Babbler	H	H	H	**H**
Sphenoeacus afer	Cape Grass-warbler	H	H	L	**L**
Sphyrapicus nuchalis	Red-naped Sapsucker	H	L	H	**L**
Sphyrapicus ruber	Red-breasted Sapsucker	H	L	L	**L**
Sphyrapicus thyroideus	Williamson's Sapsucker	H	H	L	**L**
Sphyrapicus varius	Yellow-bellied Sapsucker	H	H	L	**L**
Spiloptila clamans	Cricket Longtail	U	L	U	**L**
Spiloptila rufifrons	Red-faced Apalis	U	L	U	**L**
Spilornis cheela	Crested Serpent-eagle	U	H	L	**L**
Spilornis elgini	Andaman Serpent-eagle	H	H	H	**H**
Spilornis holospilus	Philippine Serpent-eagle	H	H	L	**L**
Spilornis kinabaluensis	Mountain Serpent-eagle	H	H	H	**H**
Spilornis klossi	South Nicobar Serpent-eagle	H	H	H	**H**
Spilornis rufipectus	Sulawesi Serpent-eagle	H	H	H	**H**
Spindalis dominicensis	Hispaniolan Spindalis	U	L	H	**L**
Spindalis nigricephala	Jamaican Spindalis	H	L	H	**L**
Spindalis portoricensis	Puerto Rican Spindalis	H	L	H	**L**
Spindalis zena	Western Spindalis	L	L	L	**L**
Spiza americana	Dickcissel	L	L	H	**L**
Spizaetus africanus	Cassin's Hawk-eagle	H	H	U	**L**
Spizaetus alboniger	Blyth's Hawk-eagle	H	H	H	**H**
Spizaetus bartelsi	Javan Hawk-eagle	H	H	H	**H**
Spizaetus cirrhatus	Changeable Hawk-eagle	U	H	U	**L**
Spizaetus floris	Flores Hawk-eagle	H	H	H	**H**
Spizaetus isidori	Black-and-chestnut Eagle	H	H	L	**L**
Spizaetus lanceolatus	Sulawesi Hawk-eagle	H	H	H	**H**
Spizaetus melanoleucus	Black-and-white Hawk-eagle	H	H	L	**L**
Spizaetus nanus	Wallace's Hawk-eagle	H	H	H	**H**
Spizaetus nipalensis	Mountain Hawk-eagle	H	H	H	**H**
Spizaetus ornatus	Ornate Hawk-eagle	H	H	H	**H**
Spizaetus philippensis	Philippine Hawk-eagle	H	H	L	**L**
Spizaetus tyrannus	Black Hawk-eagle	L	H	H	**L**
Spizella arborea	American Tree Sparrow	H	H	L	**L**
Spizella atrogularis	Black-chinned Sparrow	H	L	H	**L**
Spizella breweri	Brewer's Sparrow	H	L	H	**L**
Spizella pallida	Clay-coloured Sparrow	H	L	L	**L**
Spizella passerina	Chipping Sparrow	H	L	L	**L**
Spizella pusilla	Field Sparrow	H	L	L	**L**
Spizella wortheni	Worthen's Sparrow	H	H	H	**H**
Spiziapteryx circumcincta	Spot-winged Falconet	H	H	L	**L**
Spizixos canifrons	Crested Finchbill	U	L	H	**L**
Spizixos semitorques	Collared Finchbill	U	L	U	**L**
Spizocorys conirostris	Pink-billed Lark	H	L	H	**L**
Spizocorys fringillaris	Botha's Lark	H	H	H	**H**

Spizocorys obbiensis	Obbia Lark	H	L	L	**L**
Spizocorys personata	Masked Lark	H	U	H	**L**
Spizocorys sclateri	Sclater's Lark	H	H	H	**H**
Sporophila albogularis	White-throated Seedeater	U	U	L	**L**
Sporophila americana	Wing-barred Seedeater	H	U	H	**L**
Sporophila ardesiaca	Dubois's Seedeater	U	U	L	**L**
Sporophila bouvreuil	Capped Seedeater	U	U	L	**L**
Sporophila bouvronides	Lesson's Seedeater	H	U	H	**L**
Sporophila caerulescens	Double-collared Seedeater	U	U	L	**L**
Sporophila castaneiventris	Chestnut-bellied Seedeater	H	U	H	**L**
Sporophila cinnamomea	Chestnut Seedeater	H	U	L	**L**
Sporophila collaris	Rusty-collared Seedeater	U	U	L	**L**
Sporophila corvina	Variable Seedeater	U	U	H	**L**
Sporophila falcirostris	Temminck's Seedeater	H	U	H	**L**
Sporophila frontalis	Buffy-fronted Seedeater	H	U	L	**L**
Sporophila hypochroma	Rufous-rumped Seedeater	U	U	L	**L**
Sporophila hypoxantha	Tawny-bellied Seedeater	U	U	L	**L**
Sporophila intermedia	Grey Seedeater	U	U	H	**L**
Sporophila leucoptera	White-bellied Seedeater	U	U	L	**L**
Sporophila lineola	Lined Seedeater	U	U	L	**L**
Sporophila luctuosa	Black-and-white Seedeater	U	U	H	**L**
Sporophila melanogaster	Black-bellied Seedeater	U	U	L	**L**
Sporophila melanops	Hooded Seedeater	H	U	H	**L**
Sporophila minuta	Ruddy-breasted Seedeater	H	L	H	**L**
Sporophila murallae	Caqueta Seedeater	H	U	L	**L**
Sporophila nigricollis	Yellow-bellied Seedeater	U	L	L	**L**
Sporophila nigrorufa	Black-and-tawny Seedeater	H	U	L	**L**
Sporophila palustris	Marsh Seedeater	H	U	L	**L**
Sporophila peruviana	Parrot-billed Seedeater	H	U	H	**L**
Sporophila plumbea	Plumbeous Seedeater	U	U	L	**L**
Sporophila ruficollis	Dark-throated Seedeater	U	U	L	**L**
Sporophila schistacea	Slate-coloured Seedeater	H	U	H	**L**
Sporophila simplex	Drab Seedeater	H	U	H	**L**
Sporophila telasco	Chestnut-throated Seedeater	U	U	H	**L**
Sporophila torqueola	White-collared Seedeater	L	L	H	**L**
Sporophila zelichi	Entre Rios Seedeater	H	U	H	**L**
Sporopipes frontalis	Speckle-fronted Weaver	U	U	H	**L**
Sporopipes squamifrons	Scaly Weaver	H	L	H	**L**
Spreo albicapillus	White-crowned Starling	U	L	U	**L**
Spreo bicolor	African Pied Starling	H	H	H	**H**
Spreo fischeri	Fischer's Starling	U	L	U	**L**
Stachyris ambigua	Buff-chested Babbler	U	U	U	**L**
Stachyris capitalis	Rusty-crowned Babbler	H	H	H	**H**

Stachyris chrysaea	Golden Babbler	U	L	U	**L**
Stachyris dennistouni	Golden-crowned Babbler	H	U	L	**L**
Stachyris erythroptera	Chestnut-winged Babbler	H	H	U	**L**
Stachyris grammiceps	White-breasted Babbler	H	U	H	**L**
Stachyris herberti	Sooty Babbler	H	H	H	**H**
Stachyris hypogrammica	Palawan Striped-babbler	H	H	H	**H**
Stachyris latistriata	Panay Striped-babbler	H	H	H	**H**
Stachyris leucotis	White-necked Babbler	U	L	H	**L**
Stachyris maculata	Chestnut-rumped Babbler	H	H	H	**H**
Stachyris melanothorax	Crescent-chested Babbler	U	L	H	**L**
Stachyris nigriceps	Grey-throated Babbler	U	L	U	**L**
Stachyris nigricollis	Black-throated Babbler	H	H	H	**H**
Stachyris nigrocapitata	Black-crowned Babbler	H	H	U	**L**
Stachyris nigrorum	Negros Striped-babbler	H	H	H	**H**
Stachyris oglei	Snowy-throated Babbler	H	L	H	**L**
Stachyris plateni	Mindanao Pygmy-babbler	H	H	H	**H**
Stachyris poliocephala	Grey-headed Babbler	U	L	U	**L**
Stachyris pygmaea	Visayan Pygmy Babbler	H	H	L	**L**
Stachyris pyrrhops	Black-chinned Babbler	U	L	U	**L**
Stachyris ruficeps	Rufous-capped Babbler	H	H	H	**H**
Stachyris rufifrons	Rufous-fronted Babbler	U	L	U	**L**
Stachyris striata	Luzon Striped-babbler	H	U	L	**L**
Stachyris striolata	Spot-necked Babbler	U	L	U	**L**
Stachyris thoracica	White-bibbed Babbler	H	H	H	**H**
Stachyris whiteheadi	Chestnut-faced Babbler	U	U	H	**L**
Stactolaema anchietae	Anchieta's Barbet	H	H	H	**H**
Stactolaema leucotis	White-eared Barbet	H	H	L	**L**
Stactolaema olivacea	Green Barbet	H	H	L	**L**
Stactolaema whytii	Whyte's Barbet	H	H	U	**L**
Stagonopleura bella	Beautiful Firetail	H	H	U	**L**
Stagonopleura guttata	Diamond Firetail	H	L	L	**L**
Stagonopleura oculata	Red-eared Firetail	H	H	H	**H**
Starnoenas cyanocephala	Blue-headed Quail-dove	H	H	H	**H**
Steatornis caripensis	Oilbird	H	H	L	**L**
Steganopus tricolor	Wilson's Phalarope	U	L	U	**L**
Stelgidopteryx ruficollis	Southern Rough-winged Swallow	U	L	U	**L**
Stelgidopteryx serripennis	Northern Rough-winged Swallow	H	L	L	**L**
Stellula calliope	Calliope Hummingbird	H	H	H	**H**
Stenostira scita	Fairy Warbler	H	H	H	**H**
Stephanoaetus coronatus	Crowned Hawk-eagle	H	H	L	**L**
Stephanophorus diadematus	Diademed Tanager	H	L	L	**L**
Stephanoxis lalandi	Plovercrest	H	H	L	**L**

Stercorarius longicaudus	Long-tailed Jaeger	H	H	H	**H**
Stercorarius parasiticus	Parasitic Jaeger	H	H	L	**L**
Stercorarius pomarinus	Pomarine Jaeger	L	H	L	**L**
Sterna acuticauda	Black-bellied Tern	H	H	L	**L**
Sterna albifrons	Little Tern	L	H	L	**L**
Sterna albostriata	Black-fronted Tern	H	H	H	**H**
Sterna aleutica	Aleutian Tern	L	H	L	**L**
Sterna anaethetus	Bridled Tern	L	H	L	**L**
Sterna antillarum	Least Tern	L	H	H	**L**
Sterna aurantia	River Tern	L	H	L	**L**
Sterna balaenarum	Damara Tern	H	H	H	**H**
Sterna bengalensis	Lesser Crested Tern	L	H	L	**L**
Sterna bergii	Great Crested Tern	L	H	L	**L**
Sterna bernsteini	Chinese Crested Tern	H	H	H	**H**
Sterna caspia	Caspian Tern	L	H	L	**L**
Sterna dougallii	Roseate Tern	L	H	L	**L**
Sterna elegans	Elegant Tern	L	H	H	**L**
Sterna forsteri	Forster's Tern	L	H	L	**L**
Sterna fuscata	Sooty Tern	L	H	L	**L**
Sterna hirundinacea	South American Tern	L	H	H	**L**
Sterna hirundo	Common Tern	H	H	L	**L**
Sterna lorata	Peruvian Tern	H	H	H	**H**
Sterna lunata	Grey-backed Tern	L	H	L	**L**
Sterna maxima	Royal Tern	L	H	L	**L**
Sterna nereis	Fairy Tern	H	H	H	**H**
Sterna nilotica	Gull-billed Tern	L	H	L	**L**
Sterna paradisaea	Arctic Tern	H	H	L	**L**
Sterna repressa	White-cheeked Tern	H	H	H	**H**
Sterna sandvicensis	Sandwich Tern	L	H	H	**L**
Sterna saundersi	Saunders's Tern	L	H	H	**L**
Sterna striata	White-fronted Tern	L	H	H	**L**
Sterna sumatrana	Black-naped Tern	H	H	H	**H**
Sterna superciliaris	Yellow-billed Tern	L	H	H	**L**
Sterna trudeaui	Snowy-crowned Tern	H	H	H	**H**
Sterna virgata	Kerguelen Tern	H	H	U	**L**
Sterna vittata	Antarctic Tern	H	H	H	**H**
Sternoclyta cyanopectus	Violet-chested Hummingbird	H	H	H	**H**
Stictonetta naevosa	Freckled Duck	H	H	H	**H**
Stigmatopelia chinensis	Spotted Dove	U	L	L	**L**
Stigmatopelia senegalensis	Laughing Dove	U	H	H	**L**
Stigmatura budytoides	Greater Wagtail-tyrant	H	H	H	**H**
Stigmatura napensis	Lesser Wagtail-tyrant	H	U	H	**L**
Stiltia isabella	Australian Pratincole	L	H	L	**L**
Stiphrornis erythrothorax	Forest Robin	H	H	L	**L**

Stipiturus malachurus	Southern Emuwren	U	H	U	**L**
Stipiturus mallee	Mallee Emuwren	H	H	H	**H**
Stipiturus ruficeps	Rufous-crowned Emuwren	H	H	H	**H**
Stizorhina fraseri	Rufous Flycatcher-thrush	H	H	L	**L**
Strepera fuliginosa	Black Currawong	H	H	H	**H**
Strepera graculina	Pied Currawong	U	H	L	**L**
Strepera versicolor	Grey Currawong	U	H	U	**L**
Streptocitta albertinae	Bare-eyed Myna	H	U	L	**L**
Streptocitta albicollis	White-necked Myna	H	U	H	**L**
Streptopelia bitorquata	Island Collared-dove	H	H	L	**L**
Streptopelia capicola	Ring-necked Dove	U	H	U	**L**
Streptopelia decaocto	Eurasian Collared-dove	H	H	H	**H**
Streptopelia decipiens	Mourning Collared-dove	U	H	U	**L**
Streptopelia hypopyrrha	Adamawa Turtle-dove	U	H	U	**L**
Streptopelia lugens	Dusky Turtle-dove	U	H	L	**L**
Streptopelia orientalis	Oriental Turtle-dove	U	H	H	**L**
Streptopelia reichenowi	White-winged Collared-dove	H	H	L	**L**
Streptopelia roseogrisea	African Collared-dove	H	H	H	**H**
Streptopelia semitorquata	Red-eyed Dove	U	H	L	**L**
Streptopelia tranquebarica	Red Collared-dove	U	U	H	**L**
Streptopelia turtur	European Turtle-dove	H	H	H	**H**
Streptopelia vinacea	Vinaceous Dove	U	H	U	**L**
Streptoprocne biscutata	Biscutate Swift	H	H	L	**L**
Streptoprocne phelpsi	Tepui Swift	H	H	H	**H**
Streptoprocne rutila	Chestnut-collared Swift	H	H	L	**L**
Streptoprocne semicollaris	White-naped Swift	H	H	L	**L**
Streptoprocne zonaris	White-collared Swift	H	H	U	**L**
Stresemannia bougainvillei	Bougainville Honeyeater	H	H	H	**H**
Strigops habroptila	Kakapo	H	H	H	**H**
Strix albitarsis	Rufous-banded Owl	H	H	L	**L**
Strix aluco	Tawny Owl	H	H	H	**H**
Strix butleri	Hume's Owl	U	H	U	**L**
Strix chacoensis	Chaco Owl	H	H	L	**L**
Strix fulvescens	Fulvous Owl	H	H	H	**H**
Strix huhula	Black-banded Owl	U	H	H	**L**
Strix hylophila	Rusty-barred Owl	H	H	L	**L**
Strix leptogrammica	Brown Wood-owl	H	H	U	**L**
Strix nebulosa	Great Grey Owl	H	H	H	**H**
Strix nigrolineata	Black-and-white Owl	L	H	H	**L**
Strix occidentalis	Spotted Owl	H	H	L	**L**
Strix ocellata	Mottled Wood-owl	U	H	U	**L**
Strix rufipes	Rufous-legged Owl	H	H	H	**H**

Strix seloputo	Spotted Wood-owl	U	H	U	**L**
Strix uralensis	Ural Owl	H	H	H	**H**
Strix varia	Barred Owl	H	H	L	**L**
Strix virgata	Mottled Owl	L	H	H	**L**
Strix woodfordii	African Wood-owl	U	H	L	**L**
Struthidea cinerea	Apostlebird	H	L	L	**L**
Struthio camelus	Ostrich	H	H	L	**L**
Sturnella bellicosa	Peruvian Meadowlark	U	L	H	**L**
Sturnella defilippii	Pampas Meadowlark	H	U	H	**L**
Sturnella lilianae	Lilian's Meadowlark	H	U	U	**L**
Sturnella loyca	Long-tailed Meadowlark	U	L	U	**L**
Sturnella magna	Eastern Meadowlark	L	L	L	**L**
Sturnella militaris	Red-breasted Blackbird	H	L	H	**L**
Sturnella neglecta	Western Meadowlark	H	L	H	**L**
Sturnella superciliaris	White-browed Blackbird	U	L	L	**L**
Sturnus albofrontatus	White-faced Starling	H	H	H	**H**
Sturnus burmannicus	Vinous-breasted Starling	U	U	U	**L**
Sturnus cineraceus	White-cheeked Starling	U	L	U	**L**
Sturnus contra	Asian Pied Starling	U	L	U	**L**
Sturnus erythropygius	White-headed Starling	H	L	L	**L**
Sturnus malabaricus	Chestnut-tailed Starling	U	L	U	**L**
Sturnus melanopterus	Black-winged Starling	H	L	H	**L**
Sturnus nigricollis	Black-collared Starling	U	L	U	**L**
Sturnus pagodarum	Brahminy Starling	U	L	U	**L**
Sturnus philippensis	Chestnut-cheeked Starling	U	L	L	**L**
Sturnus roseus	Rosy Starling	H	L	L	**L**
Sturnus sericeus	Red-billed Starling	U	U	U	**L**
Sturnus sinensis	White-shouldered Starling	U	L	U	**L**
Sturnus sturninus	Purple-backed Starling	U	L	U	**L**
Sturnus unicolor	Spotless Starling	H	L	L	**L**
Sturnus vulgaris	Common Starling	H	L	H	**L**
Stymphalornis acutirostris	Parana Antwren	H	U	H	**L**
Sublegatus arenarum	Northern Scrub-flycatcher	H	H	H	**H**
Sublegatus modestus	Southern Scrub-flycatcher	U	H	L	**L**
Sublegatus obscurior	Todd's Scrub-flycatcher	H	L	H	**L**
Suiriri islerorum	Chapada Flycatcher	H	U	L	**L**
Suiriri suiriri	Suiriri Flycatcher	H	L	L	**L**
Sula dactylatra	Masked Booby	U	H	L	**L**
Sula granti	Nazca Booby	H	H	L	**L**
Sula leucogaster	Brown Booby	L	H	L	**L**
Sula nebouxii	Blue-footed Booby	L	H	H	**L**
Sula sula	Red-footed Booby	L	H	L	**L**
Sula variegata	Peruvian Booby	L	H	H	**L**
Surnia ulula	Northern Hawk Owl	H	L	H	**L**
Surniculus lugubris	Drongo Cuckoo	U	U	L	**L**

Swynnertonia swynnertoni	Swynnerton's Robin	H	H	L	**L**
Sylvia althaea	Hume's Whitethroat	U	U	U	**L**
Sylvia atricapilla	Blackcap	H	L	H	**L**
Sylvia boehmi	Banded Warbler	H	L	U	**L**
Sylvia borin	Garden Warbler	H	L	H	**L**
Sylvia buryi	Yemen Warbler	H	H	H	**H**
Sylvia cantillans	Subalpine Warbler	H	L	H	**L**
Sylvia communis	Common Whitethroat	H	L	H	**L**
Sylvia conspicillata	Spectacled Warbler	H	L	H	**L**
Sylvia curruca	Lesser Whitethroat	H	L	H	**L**
Sylvia deserticola	Tristram's Warbler	H	L	H	**L**
Sylvia hortensis	Orphean Warbler	H	L	L	**L**
Sylvia layardi	Layard's Warbler	H	H	L	**L**
Sylvia leucomelaena	Arabian Warbler	H	L	U	**L**
Sylvia lugens	Brown Warbler	U	H	L	**L**
Sylvia melanocephala	Sardinian Warbler	H	L	L	**L**
Sylvia melanothorax	Cyprus Warbler	H	L	H	**L**
Sylvia minula	Small Whitethroat	U	U	U	**L**
Sylvia mystacea	Menetries's Warbler	H	L	H	**L**
Sylvia nana	Desert Warbler	H	L	H	**L**
Sylvia nisoria	Barred Warbler	H	L	H	**L**
Sylvia rueppelli	Rueppell's Warbler	H	L	L	**L**
Sylvia sarda	Marmora's Warbler	H	L	H	**L**
Sylvia subcaerulea	Rufous-vented Warbler	U	L	U	**L**
Sylvia undata	Dartford Warbler	H	L	L	**L**
Sylvietta brachyura	Northern Crombec	U	H	H	**L**
Sylvietta denti	Lemon-bellied Crombec	H	H	L	**L**
Sylvietta isabellina	Somali Crombec	H	H	U	**L**
Sylvietta leucophrys	White-browed Crombec	U	H	U	**L**
Sylvietta philippae	Short-billed Crombec	H	U	L	**L**
Sylvietta rufescens	Cape Crombec	H	H	U	**L**
Sylvietta ruficapilla	Red-capped Crombec	U	H	U	**L**
Sylvietta virens	Green Crombec	H	H	L	**L**
Sylvietta whytii	Red-faced Crombec	U	H	U	**L**
Sylviorthorhynchus desmursii	Des Murs's Wiretail	H	L	H	**L**
Sylviparus modestus	Yellow-browed Tit	H	L	U	**L**
Syma megarhyncha	Mountain Kingfisher	H	H	H	**H**
Syma torotoro	Yellow-billed Kingfisher	H	L	L	**L**
Synallaxis albescens	Pale-breasted Spinetail	U	L	L	**L**
Synallaxis albigularis	Dark-breasted Spinetail	H	U	H	**L**
Synallaxis albilora	White-lored Spinetail	U	U	L	**L**
Synallaxis azarae	Azara's Spinetail	U	L	L	**L**
Synallaxis brachyura	Slaty Spinetail	U	L	L	**L**

Synallaxis cabanisi	Cabanis's Spinetail	H	U	H	**L**
Synallaxis candei	White-whiskered Spinetail	U	L	H	**L**
Synallaxis castanea	Black-throated Spinetail	H	H	H	**H**
Synallaxis cherriei	Chestnut-throated Spinetail	H	H	H	**H**
Synallaxis cinerascens	Grey-bellied Spinetail	H	H	L	**L**
Synallaxis cinnamomea	Stripe-breasted Spinetail	U	L	H	**L**
Synallaxis courseni	Apurimac Spinetail	H	H	H	**H**
Synallaxis erythrothorax	Rufous-breasted Spinetail	L	L	H	**L**
Synallaxis frontalis	Sooty-fronted Spinetail	U	L	L	**L**
Synallaxis fuscorufa	Rusty-headed Spinetail	H	U	H	**L**
Synallaxis gujanensis	Plain-crowned Spinetail	H	L	H	**L**
Synallaxis hypospodia	Cinereous-breasted Spinetail	U	U	L	**L**
Synallaxis infuscata	Pinto's Spinetail	H	H	H	**H**
Synallaxis kollari	Hoary-throated Spinetail	H	H	H	**H**
Synallaxis macconnelli	MacConnell's Spinetail	H	U	H	**L**
Synallaxis maranonica	Maranon Spinetail	H	U	H	**L**
Synallaxis moesta	Dusky Spinetail	U	U	H	**L**
Synallaxis propinqua	White-bellied Spinetail	H	U	L	**L**
Synallaxis ruficapilla	Rufous-capped Spinetail	H	L	L	**L**
Synallaxis rutilans	Ruddy Spinetail	H	H	H	**H**
Synallaxis scutata	Ochre-cheeked Spinetail	H	H	L	**L**
Synallaxis spixi	Spix's Spinetail	H	L	L	**L**
Synallaxis stictothorax	Necklaced Spinetail	H	U	H	**L**
Synallaxis subpudica	Silvery-throated Spinetail	U	U	H	**L**
Synallaxis tithys	Blackish-headed Spinetail	H	U	H	**L**
Synallaxis unirufa	Rufous Spinetail	H	U	H	**L**
Synallaxis whitneyi	Bahia Spinetail	H	H	H	**H**
Synallaxis zimmeri	Russet-bellied Spinetail	H	H	H	**H**
Syndactyla dimidiata	Russet-mantled Foliage-gleaner	H	H	L	**L**
Syndactyla guttulata	Guttulated Foliage-gleaner	H	H	H	**H**
Syndactyla ruficollis	Rufous-necked Foliage-gleaner	H	H	H	**H**
Syndactyla rufosuperciliata	Buff-browed Foliage-gleaner	H	L	L	**L**
Syndactyla subalaris	Lineated Foliage-gleaner	H	H	L	**L**
Synthliboramphus antiquus	Ancient Murrelet	H	H	L	**L**
Synthliboramphus craveri	Craveri's Murrelet	H	H	H	**H**
Synthliboramphus hypoleucus	Xantus's Murrelet	L	H	H	**L**
Synthliboramphus wumizusume	Japanese Murrelet	H	H	L	**L**
Sypheotides indicus	Lesser Florican	H	H	L	**L**
Syrigma sibilatrix	Whistling Heron	U	H	L	**L**
Syrmaticus ellioti	Elliot's Pheasant	H	L	H	**L**

Syrmaticus humiae	Hume's Pheasant	H	L	L	**L**
Syrmaticus mikado	Mikado Pheasant	H	L	H	**L**
Syrmaticus reevesii	Reeves's Pheasant	H	L	H	**L**
Syrmaticus soemmerringii	Copper Pheasant	H	L	H	**L**
Syrrhaptes paradoxus	Pallas's Sandgrouse	H	L	H	**L**
Syrrhaptes tibetanus	Tibetan Sandgrouse	H	L	U	**L**
Tachornis furcata	Pygmy Swift	U	H	H	**L**
Tachornis phoenicobia	Antillean Palm-swift	U	H	H	**L**
Tachornis squamata	Fork-tailed Palm-swift	H	H	H	**H**
Tachuris rubrigastra	Many-coloured Rush-tyrant	H	L	L	**L**
Tachybaptus dominicus	Least Grebe	L	L	L	**L**
Tachybaptus novaehollandiae	Australasian Grebe	L	L	L	**L**
Tachybaptus pelzelnii	Madagascar Grebe	H	L	L	**L**
Tachybaptus ruficollis	Little Grebe	L	L	H	**L**
Tachybaptus rufolavatus	Alaotra Grebe	H	H	H	**H**
Tachycineta albilinea	Mangrove Swallow	H	L	H	**L**
Tachycineta albiventer	White-winged Swallow	U	L	H	**L**
Tachycineta bicolor	Tree Swallow	H	L	H	**L**
Tachycineta cyaneoviridis	Bahama Swallow	H	H	L	**L**
Tachycineta euchrysea	Golden Swallow	H	L	H	**L**
Tachycineta leucorrhoa	White-rumped Swallow	U	L	L	**L**
Tachycineta meyeni	Chilean Swallow	U	L	L	**L**
Tachycineta stolzmanni	Tumbes Swallow	H	U	H	**L**
Tachycineta thalassina	Violet-green Swallow	H	L	H	**L**
Tachyeres brachypterus	Falkland Steamerduck	H	H	H	**H**
Tachyeres leucocephalus	White-headed Steamerduck	H	H	H	**H**
Tachyeres patachonicus	Flying Steamerduck	H	H	H	**H**
Tachyeres pteneres	Flightless Steamerduck	L	H	H	**L**
Tachymarptis aequatorialis	Mottled Swift	H	H	L	**L**
Tachymarptis melba	Alpine Swift	H	H	L	**L**
Tachyphonus coronatus	Ruby-crowned Tanager	U	L	L	**L**
Tachyphonus cristatus	Flame-crested Tanager	H	U	H	**L**
Tachyphonus delatrii	Tawny-crested Tanager	H	H	H	**H**
Tachyphonus luctuosus	White-shouldered Tanager	H	L	H	**L**
Tachyphonus phoenicius	Red-shouldered Tanager	H	H	H	**H**
Tachyphonus rufiventer	Yellow-crested Tanager	H	U	H	**L**
Tachyphonus rufus	White-lined Tanager	U	L	L	**L**
Tachyphonus surinamus	Fulvous-crested Tanager	H	U	H	**L**
Tadorna cana	South African Shelduck	H	H	L	**L**
Tadorna cristata	Crested Shelduck	H	H	H	**H**
Tadorna ferruginea	Ruddy Shelduck	H	H	H	**H**
Tadorna radjah	Radjah Shelduck	U	H	U	**L**
Tadorna tadorna	Common Shelduck	H	H	L	**L**

Tadorna tadornoides	Australian Shelduck	H	H	U	**L**
Tadorna variegata	Paradise Shelduck	U	H	U	**L**
Taeniopygia bichenovii	Double-barred Finch	U	L	U	**L**
Taeniopygia guttata	Zebra Finch	H	L	L	**L**
Taeniotriccus andrei	Black-chested Tyrant	H	H	H	**H**
Talegalla cuvieri	Red-billed Brush-turkey	H	H	H	**H**
Talegalla fuscirostris	Black-billed Brush-turkey	H	H	L	**L**
Talegalla jobiensis	Brown-collared Brush-turkey	H	H	H	**H**
Tangara argyrofenges	Straw-backed Tanager	H	U	H	**L**
Tangara arthus	Golden Tanager	U	H	L	**L**
Tangara cabanisi	Azure-rumped Tanager	H	U	H	**L**
Tangara callophrys	Opal-crowned Tanager	H	U	H	**L**
Tangara cayana	Burnished-buff Tanager	U	H	H	**L**
Tangara chilensis	Paradise Tanager	H	H	H	**H**
Tangara chrysotis	Golden-eared Tanager	H	U	H	**L**
Tangara cucullata	Lesser Antillean Tanager	H	H	H	**H**
Tangara cyanicollis	Blue-necked Tanager	U	H	L	**L**
Tangara cyanocephala	Red-necked Tanager	H	L	L	**L**
Tangara cyanoptera	Black-headed Tanager	U	U	H	**L**
Tangara cyanotis	Blue-browed Tanager	H	U	H	**L**
Tangara cyanoventris	Gilt-edged Tanager	U	U	L	**L**
Tangara desmaresti	Brassy-breasted Tanager	U	L	L	**L**
Tangara dowii	Spangle-cheeked Tanager	H	H	H	**H**
Tangara fastuosa	Seven-coloured Tanager	H	U	H	**L**
Tangara florida	Emerald Tanager	U	H	L	**L**
Tangara fucosa	Green-naped Tanager	H	H	H	**H**
Tangara guttata	Speckled Tanager	U	H	H	**L**
Tangara gyrola	Bay-headed Tanager	U	H	H	**L**
Tangara heinei	Black-capped Tanager	U	U	H	**L**
Tangara icterocephala	Silver-throated Tanager	U	H	L	**L**
Tangara inornata	Plain-coloured Tanager	H	H	H	**H**
Tangara johannae	Blue-whiskered Tanager	H	U	H	**L**
Tangara labradorides	Metallic-green Tanager	U	U	H	**L**
Tangara larvata	Golden-hooded Tanager	L	H	H	**L**
Tangara lavinia	Rufous-winged Tanager	U	L	H	**L**
Tangara mexicana	Turquoise Tanager	H	L	H	**L**
Tangara meyerdeschauenseei	Green-capped Tanager	H	H	H	**H**
Tangara nigrocincta	Masked Tanager	H	H	H	**H**
Tangara nigroviridis	Beryl-spangled Tanager	H	U	L	**L**
Tangara palmeri	Grey-and-gold Tanager	H	H	H	**H**
Tangara parzudakii	Flame-faced Tanager	H	U	L	**L**
Tangara peruviana	Black-backed Tanager	H	U	L	**L**
Tangara phillipsi	Sira Tanager	H	H	H	**H**
Tangara preciosa	Chestnut-backed Tanager	H	U	L	**L**

Tangara punctata	Spotted Tanager	H	U	H	**L**
Tangara ruficervix	Golden-naped Tanager	U	H	L	**L**
Tangara rufigenis	Rufous-cheeked Tanager	H	U	H	**L**
Tangara rufigula	Rufous-throated Tanager	H	U	H	**L**
Tangara schrankii	Green-and-gold Tanager	H	H	L	**L**
Tangara seledon	Green-headed Tanager	H	L	L	**L**
Tangara varia	Dotted Tanager	H	U	H	**L**
Tangara vassorii	Blue-and-black Tanager	H	U	L	**L**
Tangara velia	Opal-rumped Tanager	H	U	H	**L**
Tangara viridicollis	Silver-backed Tanager	U	U	L	**L**
Tangara vitriolina	Scrub Tanager	U	H	H	**L**
Tangara xanthocephala	Saffron-crowned Tanager	U	H	L	**L**
Tangara xanthogastra	Yellow-bellied Tanager	H	U	H	**L**
Tanygnathus gramineus	Black-lored Parrot	H	U	H	**L**
Tanygnathus lucionensis	Blue-naped Parrot	H	U	L	**L**
Tanygnathus megalorynchos	Great-billed Parrot	H	U	H	**L**
Tanygnathus sumatranus	Blue-backed Parrot	U	U	U	**L**
Tanysiptera carolinae	Numfor Paradise-kingfisher	H	H	H	**H**
Tanysiptera danae	Brown-headed Paradise-kingfisher	H	U	L	**L**
Tanysiptera ellioti	Kofiau Paradise-kingfisher	H	H	H	**H**
Tanysiptera galatea	Common Paradise-kingfisher	H	L	L	**L**
Tanysiptera hydrocharis	Little Paradise-kingfisher	H	U	L	**L**
Tanysiptera nympha	Red-breasted Paradise-kingfisher	H	H	H	**H**
Tanysiptera riedelii	Biak Paradise-kingfisher	H	U	H	**L**
Tanysiptera sylvia	Buff-breasted Paradise-kingfisher	H	L	L	**L**
Taoniscus nanus	Dwarf Tinamou	H	H	L	**L**
Tapera naevia	Striped Cuckoo	L	U	L	**L**
Taphrolesbia griseiventris	Grey-bellied Comet	H	U	H	**L**
Taphrospilus hypostictus	Many-spotted Hummingbird	H	H	H	**H**
Taraba major	Great Antshrike	L	L	L	**L**
Tarsiger chrysaeus	Golden Bush-robin	U	L	U	**L**
Tarsiger cyanurus	Orange-flanked Bush-robin	U	L	U	**L**
Tarsiger hyperythrus	Rufous-breasted Bush-robin	H	U	U	**L**
Tarsiger indicus	White-browed Bush-robin	U	L	U	**L**
Tarsiger johnstoniae	Collared Bush-robin	H	H	H	**H**
Tauraco bannermani	Bannerman's Turaco	H	H	H	**H**
Tauraco corythaix	Knysna Turaco	H	H	L	**L**
Tauraco erythrolophus	Red-crested Turaco	H	U	L	**L**
Tauraco fischeri	Fischer's Turaco	H	H	L	**L**
Tauraco hartlaubi	Hartlaub's Turaco	H	H	H	**H**
Tauraco leucolophus	White-crested Turaco	U	H	H	**L**

Tauraco leucotis	White-cheeked Turaco	U	H	L	**L**
Tauraco livingstonii	Livingstone's Turaco	H	H	L	**L**
Tauraco macrorhynchus	Yellow-billed Turaco	H	H	L	**L**
Tauraco persa	Guinea Turaco	U	H	H	**L**
Tauraco porphyreolophus	Purple-crested Turaco	U	L	L	**L**
Tauraco ruspolii	Prince Ruspoli's Turaco	H	U	H	**L**
Tauraco schalowi	Schalow's Turaco	U	H	L	**L**
Tauraco schuetti	Black-billed Turaco	H	H	H	**H**
Tchagra australis	Brown-crowned Tchagra	U	L	U	**L**
Tchagra jamesi	Three-streaked Tchagra	U	L	U	**L**
Tchagra minutus	Marsh Tchagra	U	L	U	**L**
Tchagra senegalus	Black-crowned Tchagra	U	L	L	**L**
Tchagra tchagra	Southern Tchagra	H	H	L	**L**
Telacanthura melanopygia	Black Spinetail	H	H	L	**L**
Telacanthura ussheri	Mottled Spinetail	H	H	H	**H**
Teledromas fuscus	Sandy Gallito	H	H	H	**H**
Telespiza cantans	Laysan Finch	H	H	U	**L**
Telespiza ultima	Nihoa Finch	H	H	U	**L**
Telophorus quadricolor	Four-coloured Bush-shrike	U	H	L	**L**
Telophorus viridis	Perrin's Bush-shrike	U	H	U	**L**
Temnurus temnurus	Ratchet-tailed Treepie	H	H	U	**L**
Tephrodornis gularis	Large Woodshrike	U	L	L	**L**
Tephrodornis pondicerianus	Common Woodshrike	U	L	H	**L**
Tephrozosterops stalkeri	Bicoloured White-eye	H	U	H	**L**
Terathopius ecaudatus	Bateleur	L	H	L	**L**
Terenotriccus erythrurus	Ruddy-tailed Flycatcher	H	H	H	**H**
Terenura callinota	Rufous-rumped Antwren	H	H	L	**L**
Terenura humeralis	Chestnut-shouldered Antwren	H	H	L	**L**
Terenura maculata	Streak-capped Antwren	H	H	L	**L**
Terenura sharpei	Yellow-rumped Antwren	H	H	H	**H**
Terenura sicki	Orange-bellied Antwren	H	H	H	**H**
Terenura spodioptila	Ash-winged Antwren	H	H	H	**H**
Teretistris fernandinae	Yellow-headed Warbler	U	L	H	**L**
Teretistris fornsi	Oriente Warbler	U	L	H	**L**
Terpsiphone atrocaudata	Japanese Paradise-flycatcher	U	L	L	**L**
Terpsiphone atrochalybeia	Sao Tome Paradise-flycatcher	H	H	L	**L**
Terpsiphone bedfordi	Bedford's Paradise-flycatcher	H	H	H	**H**
Terpsiphone bourbonnensis	Mascarene Paradise-flycatcher	U	U	H	**L**
Terpsiphone cinnamomea	Rufous Paradise-flycatcher	H	L	L	**L**
Terpsiphone corvina	Seychelles Paradise-flycatcher	H	H	U	**L**

Terpsiphone cyanescens	Blue Paradise-flycatcher	H	U	H	**L**
Terpsiphone mutata	Madagascar Paradise-flycatcher	U	L	U	**L**
Terpsiphone paradisi	Asian Paradise-flycatcher	U	L	H	**L**
Terpsiphone rufiventer	Black-headed Paradise-flycatcher	U	L	U	**L**
Terpsiphone rufocinerea	Rufous-vented Paradise-flycatcher	H	H	H	**H**
Terpsiphone viridis	African Paradise-flycatcher	U	L	L	**L**
Tersina viridis	Swallow Tanager	U	L	L	**L**
Tesia castaneocoronata	Chestnut-headed Tesia	H	H	U	**L**
Tesia cyaniventer	Grey-bellied Tesia	H	H	U	**L**
Tesia everetti	Russet-capped Tesia	U	U	H	**L**
Tesia olivea	Slaty-bellied Tesia	H	H	U	**L**
Tesia superciliaris	Javan Tesia	U	U	H	**L**
Tetrao mlokosiewiczi	Caucasian Grouse	H	H	H	**H**
Tetrao parvirostris	Black-billed Capercaillie	H	H	U	**L**
Tetrao tetrix	Black Grouse	H	H	H	**H**
Tetrao urogallus	Western Capercaillie	H	H	H	**H**
Tetraogallus altaicus	Altai Snowcock	U	U	U	**L**
Tetraogallus caspius	Caspian Snowcock	H	L	L	**L**
Tetraogallus caucasicus	Caucasian Snowcock	H	L	H	**L**
Tetraogallus himalayensis	Himalayan Snowcock	H	L	H	**L**
Tetraogallus tibetanus	Tibetan Snowcock	U	L	U	**L**
Tetraophasis obscurus	Chestnut-throated Partridge	U	U	U	**L**
Tetraophasis szechenyii	Buff-throated Partridge	U	U	U	**L**
Tetrax tetrax	Little Bustard	H	H	H	**H**
Thalassarche bulleri	Buller's Albatross	H	H	L	**L**
Thalassarche carteri	Indian Yellow-nosed Albatross	H	H	L	**L**
Thalassarche cauta	Shy Albatross	H	H	L	**L**
Thalassarche chlororhynchos	Atlantic Yellow-nosed Albatross	H	H	L	**L**
Thalassarche chrysostoma	Grey-headed Albatross	H	H	L	**L**
Thalassarche eremita	Chatham Albatross	L	H	L	**L**
Thalassarche impavida	Campbell Albatross	H	H	H	**H**
Thalassarche melanophrys	Black-browed Albatross	H	H	L	**L**
Thalassarche salvini	Salvin's Albatross	H	H	L	**L**
Thalassarche steadi	White-capped Albatross	H	H	L	**L**
Thalassoica antarctica	Antarctic Petrel	H	H	L	**L**
Thalassornis leuconotus	White-backed Duck	L	H	L	**L**
Thalurania colombica	Blue-crowned Woodnymph	U	U	H	**L**
Thalurania fannyi	Green-crowned Woodnymph	U	H	H	**L**
Thalurania furcata	Fork-tailed Woodnymph	U	U	L	**L**

Thalurania glaucopis	Violet-capped Woodnymph	H	H	L	**L**
Thalurania ridgwayi	Mexican Woodnymph	H	U	H	**L**
Thalurania watertonii	Long-tailed Woodnymph	H	H	H	**H**
Thamnistes anabatinus	Russet Antshrike	H	H	H	**H**
Thamnomanes ardesiacus	Dusky-throated Antshrike	H	H	H	**H**
Thamnomanes caesius	Cinereous Antshrike	H	H	H	**H**
Thamnomanes saturninus	Saturnine Antshrike	H	H	H	**H**
Thamnomanes schistogynus	Bluish-slate Antshrike	H	H	L	**L**
Thamnophilus aethiops	White-shouldered Antshrike	H	H	H	**H**
Thamnophilus amazonicus	Amazonian Antshrike	H	H	H	**H**
Thamnophilus ambiguus	Sooretama Slaty-antshrike	H	H	H	**H**
Thamnophilus aroyae	Upland Antshrike	H	U	H	**L**
Thamnophilus atrinucha	Western Slaty-antshrike	U	H	L	**L**
Thamnophilus bridgesi	Black-hooded Antshrike	U	L	H	**L**
Thamnophilus caerulescens	Variable Antshrike	U	L	L	**L**
Thamnophilus cryptoleucus	Castelnau's Antshrike	H	H	L	**L**
Thamnophilus divisorius	Acre Antshrike	H	H	U	**L**
Thamnophilus doliatus	Barred Antshrike	L	H	H	**L**
Thamnophilus insignis	Streak-backed Antshrike	U	U	H	**L**
Thamnophilus multistriatus	Bar-crested Antshrike	U	U	H	**L**
Thamnophilus murinus	Mouse-coloured Antshrike	H	H	H	**H**
Thamnophilus nigriceps	Black Antshrike	U	U	H	**L**
Thamnophilus nigrocinereus	Blackish-grey Antshrike	H	H	H	**H**
Thamnophilus palliatus	Chestnut-backed Antshrike	H	H	L	**L**
Thamnophilus pelzelni	Planalto Slaty-antshrike	H	H	L	**L**
Thamnophilus praecox	Cocha Antshrike	H	H	H	**H**
Thamnophilus punctatus	Northern Slaty-antshrike	H	H	H	**H**
Thamnophilus ruficapillus	Rufous-capped Antshrike	H	L	L	**L**
Thamnophilus schistaceus	Plain-winged Antshrike	H	H	L	**L**
Thamnophilus stictocephalus	Natterer's Slaty-antshrike	H	H	H	**H**
Thamnophilus sticturus	Bolivian Slaty-antshrike	H	H	L	**L**
Thamnophilus tenuepunctatus	Lined Antshrike	U	U	H	**L**
Thamnophilus torquatus	Rufous-winged Antshrike	U	L	L	**L**
Thamnophilus unicolor	Uniform Antshrike	H	H	H	**H**
Thamnophilus zarumae	Chapman's Antshrike	U	U	H	**L**
Thamnornis chloropetoides	Thamnornis Warbler	H	L	L	**L**
Thaumastura cora	Peruvian Sheartail	H	H	H	**H**
Thaumatibis gigantea	Giant Ibis	H	H	H	**H**

Theristicus caerulescens	Plumbeous Ibis	H	H	L	**L**
Theristicus caudatus	Buff-necked Ibis	U	H	U	**L**
Theristicus melanopis	Black-faced Ibis	H	H	H	**H**
Thescelocichla leucopleura	Swamp Greenbul	H	H	L	**L**
Thinocorus orbignyianus	Grey-breasted Seedsnipe	H	H	L	**L**
Thinocorus rumicivorus	Least Seedsnipe	H	H	L	**L**
Thinornis novaeseelandiae	Shore Plover	H	H	H	**H**
Thinornis rubricollis	Hooded Plover	H	H	L	**L**
Thlypopsis fulviceps	Fulvous-headed Tanager	U	U	H	**L**
Thlypopsis inornata	Buff-bellied Tanager	H	U	H	**L**
Thlypopsis ornata	Rufous-chested Tanager	H	U	H	**L**
Thlypopsis pectoralis	Brown-flanked Tanager	H	U	H	**L**
Thlypopsis ruficeps	Rust-and-yellow Tanager	H	U	H	**L**
Thlypopsis sordida	Orange-headed Tanager	U	L	L	**L**
Thraupis abbas	Yellow-winged Tanager	L	L	H	**L**
Thraupis bonariensis	Blue-and-yellow Tanager	H	L	L	**L**
Thraupis cyanocephala	Blue-capped Tanager	U	H	L	**L**
Thraupis cyanoptera	Azure-shouldered Tanager	U	H	H	**L**
Thraupis episcopus	Blue-grey Tanager	H	H	H	**H**
Thraupis glaucocolpa	Glaucous Tanager	H	U	H	**L**
Thraupis ornata	Golden-chevroned Tanager	U	L	H	**L**
Thraupis palmarum	Palm Tanager	U	H	H	**L**
Thraupis sayaca	Sayaca Tanager	U	L	L	**L**
Threnetes leucurus	Pale-tailed Barbthroat	H	H	H	**H**
Threnetes niger	Sooty Barbthroat	H	H	H	**H**
Threnetes ruckeri	Band-tailed Barbthroat	U	H	L	**L**
Threskiornis aethiopicus	African Sacred Ibis	L	H	L	**L**
Threskiornis bernieri	Madagascar Sacred Ibis	H	H	H	**H**
Threskiornis melanocephalus	Black-headed Ibis	L	H	L	**L**
Threskiornis molucca	Australian Sacred Ibis	H	H	L	**L**
Threskiornis spinicollis	Straw-necked Ibis	U	H	U	**L**
Thripadectes flammulatus	Flammulated Treehunter	H	H	H	**H**
Thripadectes holostictus	Striped Treehunter	H	U	L	**L**
Thripadectes ignobilis	Uniform Treehunter	H	H	H	**H**
Thripadectes melanorhynchus	Black-billed Treehunter	U	L	H	**L**
Thripadectes rufobrunneus	Streak-breasted Treehunter	H	H	H	**H**
Thripadectes scrutator	Buff-throated Treehunter	H	H	H	**H**
Thripadectes virgaticeps	Streak-capped Treehunter	U	H	L	**L**
Thripias namaquus	Bearded Woodpecker	H	L	L	**L**
Thripias pyrrhogaster	Fire-bellied Woodpecker	H	U	H	**L**
Thripias xantholophus	Golden-crowned	H	U	H	**L**

	Woodpecker				
Thripophaga berlepschi	Russet-mantled Softtail	H	U	H	**L**
Thripophaga cherriei	Orinoco Softtail	H	H	H	**H**
Thripophaga fusciceps	Plain Softtail	H	H	H	**H**
Thripophaga macroura	Striated Softtail	H	L	H	**L**
Thryomanes bewickii	Bewick's Wren	H	L	H	**L**
Thryorchilus browni	Timberline Wren	H	H	H	**H**
Thryothorus atrogularis	Black-throated Wren	U	U	H	**L**
Thryothorus coraya	Coraya Wren	H	H	H	**H**
Thryothorus eisenmanni	Inca Wren	H	U	H	**L**
Thryothorus euophrys	Plain-tailed Wren	H	U	H	**L**
Thryothorus fasciatoventris	Black-bellied Wren	H	U	L	**L**
Thryothorus felix	Happy Wren	L	L	H	**L**
Thryothorus genibarbis	Moustached Wren	H	U	L	**L**
Thryothorus griseus	Grey Wren	H	U	U	**L**
Thryothorus guarayanus	Fawn-breasted Wren	U	U	L	**L**
Thryothorus leucopogon	Stripe-throated Wren	H	U	L	**L**
Thryothorus leucotis	Buff-breasted Wren	H	L	H	**L**
Thryothorus longirostris	Long-billed Wren	U	U	L	**L**
Thryothorus ludovicianus	Carolina Wren	H	L	L	**L**
Thryothorus maculipectus	Spot-breasted Wren	L	L	H	**L**
Thryothorus modestus	Plain Wren	L	L	H	**L**
Thryothorus mystacalis	Whiskered Wren	U	U	H	**L**
Thryothorus nicefori	Niceforo's Wren	H	H	H	**H**
Thryothorus nigricapillus	Bay Wren	U	L	L	**L**
Thryothorus pleurostictus	Banded Wren	L	L	L	**L**
Thryothorus rufalbus	Rufous-and-white Wren	L	L	H	**L**
Thryothorus rutilus	Rufous-breasted Wren	U	L	H	**L**
Thryothorus sclateri	Speckle-breasted Wren	U	U	H	**L**
Thryothorus semibadius	Riverside Wren	H	H	H	**H**
Thryothorus sinaloa	Sinaloa Wren	H	L	H	**L**
Thryothorus spadix	Sooty-headed Wren	H	H	H	**H**
Thryothorus superciliaris	Superciliated Wren	U	L	H	**L**
Thryothorus thoracicus	Stripe-breasted Wren	H	L	H	**L**
Tiaris bicolor	Black-faced Grassquit	U	L	U	**L**
Tiaris canorus	Cuban Grassquit	U	L	U	**L**
Tiaris fuliginosus	Sooty Grassquit	U	U	U	**L**
Tiaris obscurus	Dull-coloured Grassquit	U	U	U	**L**
Tiaris olivaceus	Yellow-faced Grassquit	L	L	H	**L**
Tichodroma muraria	Wallcreeper	H	L	H	**L**
Tickellia hodgsoni	Broad-billed Warbler	H	L	H	**L**
Tigriornis leucolopha	White-crested Tiger-heron	H	H	H	**H**
Tigrisoma fasciatum	Fasciated Tiger-heron	H	H	L	**L**
Tigrisoma lineatum	Rufescent Tiger-heron	U	H	H	**L**

Tigrisoma mexicanum	Bare-throated Tiger-heron	H	H	H	**H**
Tijuca atra	Black-and-gold Cotinga	H	U	H	**L**
Tijuca condita	Grey-winged Cotinga	H	H	H	**H**
Tilmatura dupontii	Sparkling-tailed Hummingbird	H	U	L	**L**
Timalia pileata	Chestnut-capped Babbler	U	L	U	**L**
Timeliopsis fulvigula	Olive Straightbill	H	H	H	**H**
Timeliopsis griseigula	Tawny Straightbill	H	U	L	**L**
Tinamotis ingoufi	Patagonian Tinamou	H	H	H	**H**
Tinamotis pentlandii	Puna Tinamou	H	H	H	**H**
Tinamus guttatus	White-throated Tinamou	H	H	H	**H**
Tinamus major	Great Tinamou	H	H	H	**H**
Tinamus osgoodi	Black Tinamou	H	H	H	**H**
Tinamus solitarius	Solitary Tinamou	H	H	L	**L**
Tinamus tao	Grey Tinamou	H	H	L	**L**
Tityra cayana	Black-tailed Tityra	H	L	U	**L**
Tityra inquisitor	Black-crowned Tityra	H	L	U	**L**
Tityra semifasciata	Masked Tityra	H	L	L	**L**
Tmetothylacus tenellus	Golden Pipit	H	L	L	**L**
Tockus alboterminatus	Crowned Hornbill	H	H	L	**L**
Tockus bradfieldi	Bradfield's Hornbill	H	H	H	**H**
Tockus camurus	Red-billed Dwarf Hornbill	H	H	H	**H**
Tockus deckeni	Von der Decken's Hornbill	H	H	L	**L**
Tockus erythrorhynchus	Red-billed Hornbill	H	H	H	**H**
Tockus fasciatus	African Pied Hornbill	H	H	H	**H**
Tockus flavirostris	Eastern Yellow-billed Hornbill	H	H	L	**L**
Tockus hartlaubi	Black Dwarf Hornbill	H	H	H	**H**
Tockus hemprichii	Hemprich's Hornbill	H	H	L	**L**
Tockus jacksoni	Jackson's Hornbill	H	H	H	**H**
Tockus leucomelas	Southern Yellow-billed Hornbill	H	H	L	**L**
Tockus monteiri	Monteiro's Hornbill	H	H	H	**H**
Tockus nasutus	African Grey Hornbill	H	H	H	**H**
Tockus pallidirostris	Pale-billed Hornbill	H	H	L	**L**
Todiramphus albonotatus	New Britain Kingfisher	H	L	H	**L**
Todiramphus australasia	Cinnamon-banded Kingfisher	H	U	L	**L**
Todiramphus chloris	Collared Kingfisher	U	L	L	**L**
Todiramphus cinnamominus	Micronesian Kingfisher	H	H	L	**L**
Todiramphus diops	Blue-and-white Kingfisher	H	U	H	**L**
Todiramphus enigma	Talaud Kingfisher	H	U	H	**L**
Todiramphus farquhari	Chestnut-bellied Kingfisher	H	L	H	**L**
Todiramphus funebris	Sombre Kingfisher	H	U	H	**L**
Todiramphus gambieri	Tuamotu Kingfisher	H	H	U	**L**

Todiramphus godeffroyi	Marquesan Kingfisher	H	H	U	**L**
Todiramphus lazuli	Lazuli Kingfisher	H	L	H	**L**
Todiramphus leucopygius	Ultramarine Kingfisher	H	L	H	**L**
Todiramphus macleayii	Forest Kingfisher	H	L	L	**L**
Todiramphus nigrocyaneus	Blue-black Kingfisher	H	U	L	**L**
Todiramphus pyrrhopygius	Red-backed Kingfisher	U	L	U	**L**
Todiramphus recurvirostris	Flat-billed Kingfisher	H	L	U	**L**
Todiramphus ruficollaris	Mangaia Kingfisher	H	H	U	**L**
Todiramphus sanctus	Sacred Kingfisher	H	L	L	**L**
Todiramphus saurophaga	Beach Kingfisher	H	L	U	**L**
Todiramphus tutus	Chattering Kingfisher	H	U	U	**L**
Todiramphus veneratus	Tahiti Kingfisher	H	L	U	**L**
Todiramphus winchelli	Rufous-lored Kingfisher	H	U	H	**L**
Todirostrum chrysocrotaphum	Yellow-browed Tody-flycatcher	H	H	L	**L**
Todirostrum cinereum	Common Tody-flycatcher	L	L	L	**L**
Todirostrum maculatum	Spotted Tody-flycatcher	H	H	H	**H**
Todirostrum nigriceps	Black-headed Tody-flycatcher	U	H	H	**L**
Todirostrum pictum	Painted Tody-flycatcher	H	U	H	**L**
Todirostrum poliocephalum	Yellow-lored Tody-flycatcher	U	U	L	**L**
Todirostrum viridanum	Maracaibo Tody-flycatcher	H	U	H	**L**
Todus angustirostris	Narrow-billed Tody	H	L	H	**L**
Todus mexicanus	Puerto Rican Tody	H	L	H	**L**
Todus multicolor	Cuban Tody	U	L	H	**L**
Todus subulatus	Broad-billed Tody	U	L	H	**L**
Todus todus	Jamaican Tody	H	L	H	**L**
Tolmomyias assimilis	Yellow-margined Flycatcher	H	H	H	**H**
Tolmomyias flaviventris	Yellow-breasted Flycatcher	H	L	H	**L**
Tolmomyias poliocephalus	Grey-crowned Flycatcher	H	H	H	**H**
Tolmomyias sulphurescens	Yellow-olive Flycatcher	L	L	H	**L**
Tolmomyias traylori	Orange-eyed Flycatcher	H	H	H	**H**
Topaza pella	Crimson Topaz	H	H	H	**H**
Topaza pyra	Fiery Topaz	H	H	H	**H**
Torgos tracheliotos	Lappet-faced Vulture	H	H	L	**L**
Torreornis inexpectata	Cuban Sparrow	H	H	H	**H**
Touit batavicus	Lilac-tailed Parrotlet	H	L	H	**L**
Touit costaricensis	Red-fronted Parrotlet	H	L	H	**L**
Touit dilectissimus	Blue-fronted Parrotlet	H	U	H	**L**
Touit huetii	Scarlet-shouldered Parrotlet	H	U	H	**L**

Touit melanonotus	Brown-backed Parrotlet	H	U	H	**L**
Touit purpuratus	Sapphire-rumped Parrotlet	H	L	H	**L**
Touit stictopterus	Spot-winged Parrotlet	H	U	H	**L**
Touit surdus	Golden-tailed Parrotlet	H	U	H	**L**
Toxorhamphus iliolophus	Plumed Longbill	U	H	H	**L**
Toxorhamphus novaeguineae	Green-crowned Longbill	H	U	H	**L**
Toxorhamphus poliopterus	Grey-winged Longbill	U	H	H	**L**
Toxostoma bendirei	Bendire's Thrasher	H	L	H	**L**
Toxostoma cinereum	Grey Thrasher	H	L	H	**L**
Toxostoma crissale	Crissal Thrasher	H	L	H	**L**
Toxostoma curvirostre	Curve-billed Thrasher	H	L	H	**L**
Toxostoma guttatum	Cozumel Thrasher	H	H	H	**H**
Toxostoma lecontei	Le Conte's Thrasher	H	L	H	**L**
Toxostoma longirostre	Long-billed Thrasher	H	L	H	**L**
Toxostoma ocellatum	Ocellated Thrasher	H	H	H	**H**
Toxostoma redivivum	California Thrasher	H	L	L	**L**
Toxostoma rufum	Brown Thrasher	H	L	L	**L**
Trachyphonus darnaudii	D'Arnaud's Barbet	U	H	U	**L**
Trachyphonus erythrocephalus	Red-and-yellow Barbet	H	H	L	**L**
Trachyphonus margaritatus	Yellow-breasted Barbet	U	H	U	**L**
Trachyphonus purpuratus	Yellow-billed Barbet	H	H	H	**H**
Trachyphonus usambiro	Usambiro Barbet	H	H	L	**L**
Trachyphonus vaillantii	Crested Barbet	H	H	L	**L**
Tragopan blythii	Blyth's Tragopan	H	L	H	**L**
Tragopan caboti	Cabot's Tragopan	H	L	L	**L**
Tragopan melanocephalus	Western Tragopan	H	L	H	**L**
Tragopan satyra	Satyr Tragopan	H	L	L	**L**
Tragopan temminckii	Temminck's Tragopan	H	L	H	**L**
Tregellasia capito	Pale-yellow Robin	H	H	L	**L**
Tregellasia leucops	White-faced Robin	U	H	U	**L**
Treron apicauda	Pin-tailed Green-pigeon	H	H	H	**H**
Treron australis	Madagascar Green-pigeon	U	H	U	**L**
Treron bicinctus	Orange-breasted Green-pigeon	U	U	L	**L**
Treron calvus	African Green-pigeon	U	H	L	**L**
Treron capellei	Large Green-pigeon	H	U	H	**L**
Treron curvirostra	Thick-billed Green-pigeon	U	H	L	**L**
Treron floris	Flores Green-pigeon	H	U	H	**L**
Treron formosae	Whistling Green-pigeon	U	H	H	**L**
Treron fulvicollis	Cinnamon-headed Green-pigeon	H	H	H	**H**

Treron griseicauda	Grey-cheeked Green-pigeon	U	H	U	**L**
Treron olax	Little Green-pigeon	H	U	H	**L**
Treron oxyurus	Sumatran Green-pigeon	H	U	H	**L**
Treron pembaensis	Pemba Green-pigeon	H	H	H	**H**
Treron phoenicopterus	Yellow-footed Green-pigeon	U	H	L	**L**
Treron pompadora	Pompadour Green-pigeon	H	H	L	**L**
Treron psittaceus	Timor Green-pigeon	H	U	H	**L**
Treron sanctithomae	Sao Tome Green-pigeon	H	H	L	**L**
Treron seimundi	Yellow-vented Green-pigeon	H	U	L	**L**
Treron sieboldii	White-bellied Green-pigeon	U	H	L	**L**
Treron sphenurus	Wedge-tailed Green-pigeon	U	H	H	**L**
Treron teysmannii	Sumba Green-pigeon	H	U	H	**L**
Treron vernans	Pink-necked Green-pigeon	H	H	L	**L**
Treron waalia	Bruce's Green-pigeon	U	H	L	**L**
Trichastoma bicolor	Ferruginous Babbler	U	H	U	**L**
Trichastoma buettikoferi	Sumatran Babbler	U	U	H	**L**
Trichastoma celebense	Sulawesi Babbler	U	H	U	**L**
Trichastoma pyrrogenys	Temminck's Babbler	H	H	U	**L**
Trichastoma rostratum	White-chested Babbler	H	H	H	**H**
Trichastoma tickelli	Buff-breasted Babbler	U	L	U	**L**
Trichixos pyrropygus	Rufous-tailed Shama	H	H	H	**H**
Trichocichla rufa	Long-legged Thicketbird	H	H	H	**H**
Trichodere cockerelli	White-streaked Honeyeater	U	H	L	**L**
Trichoglossus chlorolepidotus	Scaly-breasted Lorikeet	U	L	U	**L**
Trichoglossus euteles	Olive-headed Lorikeet	U	H	U	**L**
Trichoglossus flavoviridis	Yellow-and-green Lorikeet	U	H	H	**L**
Trichoglossus haematodus	Rainbow Lorikeet	U	H	L	**L**
Trichoglossus johnstoniae	Mindanao Lorikeet	H	H	H	**H**
Trichoglossus ornatus	Ornate Lorikeet	U	H	U	**L**
Trichoglossus rubiginosus	Pohnpei Lorikeet	H	H	H	**H**
Tricholaema diademata	Red-fronted Barbet	U	H	U	**L**
Tricholaema frontata	Miombo Barbet	H	H	U	**L**
Tricholaema hirsuta	Hairy-breasted Barbet	H	H	L	**L**
Tricholaema lacrymosa	Spot-flanked Barbet	H	H	H	**H**
Tricholaema leucomelas	Pied Barbet	H	H	L	**L**
Tricholaema melanocephala	Black-throated Barbet	H	H	L	**L**
Tricholestes criniger	Hairy-backed Bulbul	H	H	U	**L**
Trichothraupis melanops	Black-goggled Tanager	U	L	L	**L**
Triclaria malachitacea	Blue-bellied Parrot	H	L	L	**L**
Trigonoceps occipitalis	White-headed Vulture	H	H	L	**L**
Tringa erythropus	Spotted Redshank	L	H	H	**L**
Tringa flavipes	Lesser Yellowlegs	U	H	U	**L**

Tringa glareola	Wood Sandpiper	L	H	H	**L**
Tringa guttifer	Spotted Greenshank	H	L	H	**L**
Tringa melanoleuca	Greater Yellowlegs	L	L	L	**L**
Tringa nebularia	Common Greenshank	L	H	H	**L**
Tringa ochropus	Green Sandpiper	L	L	H	**L**
Tringa solitaria	Solitary Sandpiper	L	L	L	**L**
Tringa stagnatilis	Marsh Sandpiper	L	L	H	**L**
Tringa totanus	Common Redshank	H	H	H	**H**
Trochilus polytmus	Red-billed Streamertail	U	H	H	**L**
Trochilus scitulus	Black-billed Streamertail	H	H	H	**H**
Trochocercus cyanomelas	African Crested-flycatcher	U	L	L	**L**
Trochocercus nitens	Blue-headed Crested-flycatcher	H	H	L	**L**
Troglodytes aedon	House Wren	L	L	L	**L**
Troglodytes cobbi	Cobb's Wren	H	H	H	**H**
Troglodytes monticola	Santa Marta Wren	H	U	H	**L**
Troglodytes ochraceus	Ochraceous Wren	H	H	H	**H**
Troglodytes rufociliatus	Rufous-browed Wren	H	H	H	**H**
Troglodytes rufulus	Tepui Wren	H	H	H	**H**
Troglodytes sissonii	Socorro Wren	H	H	L	**L**
Troglodytes solstitialis	Mountain Wren	H	H	L	**L**
Troglodytes tanneri	Clarion Wren	H	H	U	**L**
Troglodytes troglodytes	Winter Wren	H	L	H	**L**
Trogon aurantiiventris	Orange-bellied Trogon	H	H	H	**H**
Trogon bairdii	Baird's Trogon	H	H	H	**H**
Trogon citreolus	Citreoline Trogon	H	H	H	**H**
Trogon clathratus	Lattice-tailed Trogon	H	H	H	**H**
Trogon collaris	Collared Trogon	H	H	H	**H**
Trogon comptus	White-eyed Trogon	H	H	H	**H**
Trogon curucui	Blue-crowned Trogon	H	H	L	**L**
Trogon elegans	Elegant Trogon	H	H	H	**H**
Trogon massena	Slaty-tailed Trogon	H	H	H	**H**
Trogon melanocephalus	Black-headed Trogon	H	H	H	**H**
Trogon melanurus	Black-tailed Trogon	H	H	H	**H**
Trogon mexicanus	Mountain Trogon	H	H	L	**L**
Trogon personatus	Masked Trogon	H	H	L	**L**
Trogon rufus	Black-throated Trogon	H	H	H	**H**
Trogon surrucura	Surucua Trogon	H	H	L	**L**
Trogon violaceus	Violaceous Trogon	H	H	H	**H**
Trogon viridis	White-tailed Trogon	H	H	H	**H**
Tropicranus albocristatus	White-crested Hornbill	H	H	H	**H**
Trugon terrestris	Thick-billed Ground-pigeon	H	H	L	**L**
Tryngites subruficollis	Buff-breasted Sandpiper	L	H	L	**L**
Tumbezia salvini	Tumbes Tyrant	H	U	H	**L**
Turacoena manadensis	White-faced Cuckoo-dove	H	H	H	**H**

Turacoena modesta	Slaty Cuckoo-dove	H	U	H	**L**
Turdinus atrigularis	Black-throated Wren-babbler	H	H	H	**H**
Turdinus macrodactylus	Large Wren-babbler	H	H	H	**H**
Turdinus marmorata	Marbled Wren-babbler	H	H	L	**L**
Turdinus rufipectus	Rusty-breasted Wren-babbler	H	H	H	**H**
Turdoides affinis	Yellow-billed Babbler	U	H	U	**L**
Turdoides altirostris	Iraq Babbler	H	H	H	**H**
Turdoides aylmeri	Scaly Chatterer	U	H	U	**L**
Turdoides bicolor	Southern Pied-babbler	H	H	H	**H**
Turdoides caudata	Common Babbler	U	H	L	**L**
Turdoides earlei	Striated Babbler	U	H	U	**L**
Turdoides fulva	Fulvous Chatterer	H	H	H	**H**
Turdoides gularis	White-throated Babbler	U	H	H	**L**
Turdoides gymnogenys	Bare-cheeked Babbler	H	H	L	**L**
Turdoides hartlaubii	Angola Babbler	U	H	U	**L**
Turdoides hindei	Hinde's Pied-babbler	H	H	H	**H**
Turdoides hypoleuca	Northern Pied-babbler	U	H	H	**L**
Turdoides jardineii	Arrow-marked Babbler	U	H	U	**L**
Turdoides leucocephala	Cretschmar's Babbler	U	H	U	**L**
Turdoides leucopygia	White-rumped Babbler	U	H	U	**L**
Turdoides longirostris	Slender-billed Babbler	H	H	H	**H**
Turdoides malcolmi	Large Grey Babbler	U	H	U	**L**
Turdoides melanops	Black-lored Babbler	H	H	H	**H**
Turdoides nipalensis	Spiny Babbler	H	H	H	**H**
Turdoides plebejus	Brown Babbler	U	H	U	**L**
Turdoides reinwardii	Blackcap Babbler	U	H	U	**L**
Turdoides rubiginosa	Rufous Chatterer	U	H	U	**L**
Turdoides rufescens	Orange-billed Babbler	U	H	H	**L**
Turdoides sharpei	Sharpe's Pied-babbler	U	H	H	**L**
Turdoides squamiceps	Arabian Babbler	U	H	U	**L**
Turdoides squamulata	Scaly Babbler	U	H	U	**L**
Turdoides striata	Jungle Babbler	U	H	U	**L**
Turdoides subrufa	Rufous Babbler	U	H	H	**L**
Turdoides tenebrosa	Dusky Babbler	U	H	U	**L**
Turdus albicollis	White-necked Thrush	U	L	H	**L**
Turdus albocinctus	White-collared Blackbird	U	H	U	**L**
Turdus amaurochalinus	Creamy-bellied Thrush	U	L	L	**L**
Turdus assimilis	White-throated Thrush	L	H	L	**L**
Turdus aurantius	White-chinned Thrush	H	H	H	**H**
Turdus bewsheri	Comoro Thrush	H	H	H	**H**
Turdus boulboul	Grey-winged Blackbird	H	H	U	**L**
Turdus cardis	Japanese Thrush	U	H	L	**L**
Turdus celaenops	Izu Thrush	H	H	L	**L**

Turdus chiguanco	Chiguanco Thrush	H	H	H	**H**
Turdus chrysolaus	Brown-headed Thrush	U	H	U	**L**
Turdus dissimilis	Black-breasted Thrush	H	H	U	**L**
Turdus falcklandii	Austral Thrush	U	H	L	**L**
Turdus feae	Grey-sided Thrush	H	H	H	**H**
Turdus flavipes	Yellow-legged Thrush	U	U	H	**L**
Turdus fulviventris	Chestnut-bellied Thrush	U	H	H	**L**
Turdus fumigatus	Cocoa Thrush	H	L	H	**L**
Turdus fuscater	Great Thrush	U	H	H	**L**
Turdus grayi	Clay-coloured Thrush	L	H	H	**L**
Turdus haplochrous	Unicoloured Thrush	H	H	L	**L**
Turdus hauxwelli	Hauxwell's Thrush	H	H	L	**L**
Turdus helleri	Taita Thrush	H	H	H	**H**
Turdus hortulorum	Grey-backed Thrush	U	H	U	**L**
Turdus ignobilis	Black-billed Thrush	H	H	H	**H**
Turdus iliacus	Redwing	H	L	H	**L**
Turdus infuscatus	Black Thrush	H	H	H	**H**
Turdus jamaicensis	White-eyed Thrush	H	L	H	**L**
Turdus kessleri	White-backed Thrush	U	H	U	**L**
Turdus lawrencii	Lawrence's Thrush	H	H	H	**H**
Turdus leucomelas	Pale-breasted Thrush	U	L	H	**L**
Turdus leucops	Pale-eyed Thrush	H	H	L	**L**
Turdus libonyanus	Kurrichane Thrush	U	H	U	**L**
Turdus ludoviciae	Somali Thrush	H	H	H	**H**
Turdus maranonicus	Maranon Thrush	H	H	H	**H**
Turdus menachensis	Yemen Thrush	H	H	H	**H**
Turdus merula	Eurasian Blackbird	H	L	H	**L**
Turdus migratorius	American Robin	H	L	L	**L**
Turdus mupinensis	Chinese Thrush	U	H	U	**L**
Turdus naumanni	Dusky Thrush	U	H	U	**L**
Turdus nigrescens	Sooty Thrush	U	H	H	**L**
Turdus nigriceps	Slaty Thrush	U	H	H	**L**
Turdus nudigenis	Spectacled Thrush	U	L	U	**L**
Turdus obscurus	Eyebrowed Thrush	H	H	U	**L**
Turdus obsoletus	Pale-vented Thrush	U	H	L	**L**
Turdus olivaceofuscus	Sao Tome Thrush	H	H	L	**L**
Turdus olivaceus	Olive Thrush	U	L	U	**L**
Turdus olivater	Black-hooded Thrush	U	H	H	**L**
Turdus pallidus	Pale Thrush	U	H	U	**L**
Turdus pelios	African Thrush	U	H	U	**L**
Turdus philomelos	Song Thrush	H	L	H	**L**
Turdus pilaris	Fieldfare	H	L	H	**L**
Turdus plebejus	American Mountain Thrush	L	H	H	**L**
Turdus plumbeus	Red-legged Thrush	U	H	H	**L**
Turdus poliocephalus	Island Thrush	H	H	H	**H**

Turdus reevei	Plumbeous-backed Thrush	U	H	H	**L**
Turdus rubrocanus	Chestnut Thrush	H	H	U	**L**
Turdus ruficollis	Dark-throated Thrush	H	H	H	**H**
Turdus rufitorques	Rufous-collared Robin	L	H	H	**L**
Turdus rufiventris	Rufous-bellied Thrush	U	L	L	**L**
Turdus rufopalliatus	Rufous-backed Robin	U	H	H	**L**
Turdus serranus	Glossy-black Thrush	H	H	L	**L**
Turdus swalesi	La Selle Thrush	H	H	H	**H**
Turdus tephronotus	Bare-eyed Thrush	U	H	U	**L**
Turdus torquatus	Ring Ouzel	H	H	L	**L**
Turdus unicolor	Tickell's Thrush	U	H	U	**L**
Turdus viscivorus	Mistle Thrush	H	H	H	**H**
Turnix castanotus	Chestnut-backed Buttonquail	L	L	L	**L**
Turnix everetti	Sumba Buttonquail	H	L	H	**L**
Turnix hottentottus	Hottentot Buttonquail	U	L	U	**L**
Turnix maculosus	Red-backed Buttonquail	U	L	U	**L**
Turnix melanogaster	Black-breasted Buttonquail	H	L	L	**L**
Turnix nigricollis	Madagascar Buttonquail	U	L	U	**L**
Turnix ocellatus	Spotted Buttonquail	U	U	L	**L**
Turnix olivii	Buff-breasted Buttonquail	H	L	L	**L**
Turnix pyrrhothorax	Red-chested Buttonquail	U	L	U	**L**
Turnix suscitator	Barred Buttonquail	U	L	U	**L**
Turnix sylvaticus	Small Buttonquail	U	L	L	**L**
Turnix tanki	Yellow-legged Buttonquail	U	L	H	**L**
Turnix varius	Painted Buttonquail	U	L	U	**L**
Turnix velox	Little Buttonquail	U	L	U	**L**
Turnix worcesteri	Luzon Buttonquail	U	U	H	**L**
Turtur abyssinicus	Black-billed Wood-dove	U	H	H	**L**
Turtur afer	Blue-spotted Wood-dove	U	H	L	**L**
Turtur brehmeri	Blue-headed Wood-dove	H	H	H	**H**
Turtur chalcospilos	Emerald-spotted Wood-dove	U	H	L	**L**
Turtur tympanistria	Tambourine Dove	U	H	L	**L**
Tylas eduardi	Tylas Vanga	U	H	U	**L**
Tympanuchus cupido	Greater Prairie-chicken	H	L	L	**L**
Tympanuchus pallidicinctus	Lesser Prairie-chicken	H	H	H	**H**
Tympanuchus phasianellus	Sharp-tailed Grouse	H	L	H	**L**
Tyranneutes stolzmanni	Dwarf Tyrant-manakin	H	H	H	**H**
Tyranneutes virescens	Tiny Tyrant-manakin	H	H	H	**H**
Tyrannopsis sulphurea	Sulphury Flycatcher	H	H	H	**H**
Tyrannulus elatus	Yellow-crowned Tyrannulet	H	H	H	**H**
Tyrannus albogularis	White-throated Kingbird	H	U	H	**L**
Tyrannus caudifasciatus	Loggerhead Kingbird	U	L	H	**L**
Tyrannus couchii	Couch's Kingbird	L	L	H	**L**

Tyrannus crassirostris	Thick-billed Kingbird	L	L	H	**L**
Tyrannus cubensis	Giant Kingbird	H	L	H	**L**
Tyrannus dominicensis	Grey Kingbird	L	L	H	**L**
Tyrannus forficatus	Scissor-tailed Flycatcher	L	L	H	**L**
Tyrannus melancholicus	Tropical Kingbird	L	L	L	**L**
Tyrannus niveigularis	Snowy-throated Kingbird	U	L	H	**L**
Tyrannus savana	Fork-tailed Flycatcher	L	L	L	**L**
Tyrannus tyrannus	Eastern Kingbird	L	L	L	**L**
Tyrannus verticalis	Western Kingbird	H	L	H	**L**
Tyrannus vociferans	Cassin's Kingbird	H	L	H	**L**
Tyto alba	Barn Owl	L	H	L	**L**
Tyto aurantia	Bismarck Masked-owl	H	H	H	**H**
Tyto capensis	African Grass-owl	U	H	U	**L**
Tyto glaucops	Ashy-faced Owl	U	H	H	**L**
Tyto inexspectata	Sulawesi Golden Owl	H	H	H	**H**
Tyto longimembris	Eastern Grass-owl	U	H	U	**L**
Tyto manusi	Manus Masked-owl	H	H	H	**H**
Tyto nigrobrunnea	Taliabu Masked-owl	H	H	L	**L**
Tyto novaehollandiae	Australian Masked-owl	U	H	L	**L**
Tyto rosenbergii	Sulawesi Owl	U	H	U	**L**
Tyto sororcula	Lesser Masked-owl	H	H	H	**H**
Tyto soumagnei	Madagascar Red Owl	H	H	H	**H**
Tyto tenebricosa	Sooty Owl	U	H	U	**L**
Upucerthia albigula	White-throated Earthcreeper	H	U	H	**L**
Upucerthia andaecola	Rock Earthcreeper	H	H	H	**H**
Upucerthia certhioides	Chaco Earthcreeper	H	L	H	**L**
Upucerthia dumetaria	Scale-throated Earthcreeper	H	L	L	**L**
Upucerthia harterti	Bolivian Earthcreeper	H	U	H	**L**
Upucerthia jelskii	Plain-breasted Earthcreeper	H	U	H	**L**
Upucerthia ruficaudus	Straight-billed Earthcreeper	H	H	H	**H**
Upucerthia serrana	Striated Earthcreeper	H	H	H	**H**
Upucerthia validirostris	Buff-breasted Earthcreeper	H	H	H	**H**
Upupa epops	Eurasian Hoopoe	L	H	H	**L**
Upupa marginata	Madagascar Hoopoe	U	H	L	**L**
Uraeginthus angolensis	Blue-breasted Cordonbleu	U	L	U	**L**
Uraeginthus bengalus	Red-cheeked Cordonbleu	U	L	U	**L**
Uraeginthus cyanocephalus	Blue-capped Cordonbleu	U	U	U	**L**
Uraeginthus granatinus	Common Grenadier	U	L	U	**L**
Uraeginthus ianthinogaster	Purple Grenadier	H	U	L	**L**
Uragus sibiricus	Long-tailed Rosefinch	U	U	U	**L**
Uratelornis chimaera	Long-tailed Ground-roller	H	L	H	**L**
Uria aalge	Common Guillemot	H	H	L	**L**
Uria lomvia	Thick-billed Guillemot	H	H	L	**L**

Urochroa bougueri	White-tailed Hillstar	H	H	H	**H**
Urocissa caerulea	Taiwan Magpie	H	H	H	**H**
Urocissa erythrorhyncha	Blue Magpie	U	H	H	**L**
Urocissa flavirostris	Gold-billed Magpie	U	H	U	**L**
Urocissa ornata	Sri Lanka Magpie	H	H	H	**H**
Urocissa whiteheadi	White-winged Magpie	U	H	U	**L**
Urocolius indicus	Red-faced Mousebird	U	L	U	**L**
Urocolius macrourus	Blue-naped Mousebird	U	L	L	**L**
Urocynchramus pylzowi	Pink-tailed Bunting	U	U	U	**L**
Uroglaux dimorpha	Papuan Hawk-owl	U	U	L	**L**
Urolais epichlorus	Green Longtail	U	U	L	**L**
Urolestes melanoleucus	Magpie Shrike	U	L	L	**L**
Uropelia campestris	Long-tailed Ground-dove	U	U	L	**L**
Uropsalis lyra	Lyre-tailed Nightjar	H	U	L	**L**
Uropsalis segmentata	Swallow-tailed Nightjar	U	U	L	**L**
Uropsila leucogastra	White-bellied Wren	L	L	H	**L**
Urosphena squameiceps	Asian Stubtail	U	L	U	**L**
Urosphena subulata	Timor Stubtail	U	U	H	**L**
Urosphena whiteheadi	Bornean Stubtail	H	H	H	**H**
Urosticte benjamini	Purple-bibbed Whitetip	H	H	H	**H**
Urosticte ruficrissa	Rufous-vented Whitetip	H	H	H	**H**
Urothraupis stolzmanni	Black-backed Bush-finch	H	U	H	**L**
Urotriorchis macrourus	Long-tailed Hawk	H	H	U	**L**
Vanellus albiceps	White-headed Lapwing	L	H	H	**L**
Vanellus armatus	Blacksmith Lapwing	L	H	L	**L**
Vanellus cayanus	Pied Lapwing	H	H	H	**H**
Vanellus chilensis	Southern Lapwing	L	H	L	**L**
Vanellus cinereus	Grey-headed Lapwing	L	H	H	**L**
Vanellus coronatus	Crowned Lapwing	L	H	L	**L**
Vanellus crassirostris	Long-toed Lapwing	L	H	L	**L**
Vanellus duvaucelii	River Lapwing	H	H	U	**L**
Vanellus gregarius	Sociable Lapwing	H	H	H	**H**
Vanellus indicus	Red-wattled Lapwing	L	H	H	**L**
Vanellus leucurus	White-tailed Lapwing	H	H	H	**H**
Vanellus lugubris	Senegal Lapwing	L	H	L	**L**
Vanellus macropterus	Javan Lapwing	H	H	L	**L**
Vanellus malarbaricus	Yellow-wattled Lapwing	H	H	U	**L**
Vanellus melanocephalus	Spot-breasted Lapwing	H	H	L	**L**
Vanellus melanopterus	Black-winged Lapwing	L	H	L	**L**
Vanellus miles	Masked Lapwing	L	H	L	**L**
Vanellus resplendens	Andean Lapwing	H	H	H	**H**
Vanellus senegallus	Wattled Lapwing	L	H	L	**L**
Vanellus spinosus	Spur-winged Lapwing	L	H	H	**L**
Vanellus superciliosus	Brown-chested Lapwing	H	H	H	**H**
Vanellus tectus	Black-headed Lapwing	U	H	U	**L**

Vanellus tricolor	Banded Lapwing	U	H	U	**L**
Vanellus vanellus	Northern Lapwing	H	H	H	**H**
Vanga curvirostris	Hook-billed Vanga	U	L	L	**L**
Veniliornis affinis	Red-stained Woodpecker	H	U	L	**L**
Veniliornis callonotus	Scarlet-backed Woodpecker	U	U	H	**L**
Veniliornis cassini	Golden-collared Woodpecker	H	U	H	**L**
Veniliornis chocoensis	Choco Woodpecker	H	U	H	**L**
Veniliornis dignus	Yellow-vented Woodpecker	H	U	H	**L**
Veniliornis frontalis	Dot-fronted Woodpecker	H	U	H	**L**
Veniliornis kirkii	Red-rumped Woodpecker	U	L	L	**L**
Veniliornis lignarius	Striped Woodpecker	U	L	H	**L**
Veniliornis maculifrons	Yellow-eared Woodpecker	U	U	H	**L**
Veniliornis mixtus	Checkered Woodpecker	U	L	L	**L**
Veniliornis nigriceps	Bar-bellied Woodpecker	H	U	H	**L**
Veniliornis passerinus	Little Woodpecker	H	U	H	**L**
Veniliornis sanguineus	Blood-coloured Woodpecker	H	H	H	**H**
Veniliornis spilogaster	White-spotted Woodpecker	H	U	L	**L**
Vermivora bachmanii	Bachman's Warbler	H	L	L	**L**
Vermivora celata	Orange-crowned Warbler	H	L	L	**L**
Vermivora chrysoptera	Golden-winged Warbler	L	L	L	**L**
Vermivora crissalis	Colima Warbler	H	L	L	**L**
Vermivora luciae	Lucy's Warbler	H	L	H	**L**
Vermivora peregrina	Tennessee Warbler	H	L	L	**L**
Vermivora pinus	Blue-winged Warbler	H	L	L	**L**
Vermivora ruficapilla	Nashville Warbler	H	L	L	**L**
Vermivora virginiae	Virginia's Warbler	H	L	L	**L**
Vestiaria coccinea	Iiwi	H	H	H	**H**
Vidua chalybeata	Village Indigobird	U	U	U	**L**
Vidua codringtoni	Twinspot Indigobird	U	U	U	**L**
Vidua fischeri	Straw-tailed Whydah	U	U	U	**L**
Vidua funerea	Variable Indigobird	U	U	L	**L**
Vidua hypocherina	Steel-blue Whydah	U	U	U	**L**
Vidua interjecta	Long-tailed Paradise-whydah	U	U	U	**L**
Vidua larvaticola	Baka Indigobird	U	U	U	**L**
Vidua macroura	Pin-tailed Whydah	U	U	L	**L**
Vidua maryae	Jos Plateau Indigobird	U	U	L	**L**
Vidua obtusa	Broad-tailed Paradise-whydah	U	U	U	**L**
Vidua paradisaea	Eastern Paradise-whydah	U	U	L	**L**
Vidua purpurascens	Dusky Indigobird	U	U	U	**L**
Vidua raricola	Jambandu Indigobird	U	U	U	**L**
Vidua regia	Queen Whydah	H	U	L	**L**
Vidua togoensis	Togo Paradise-whydah	U	U	U	**L**
Vidua wilsoni	Pale-winged Indigobird	U	U	U	**L**

Vini australis	Blue-crowned Lorikeet	H	H	U	**L**
Vini kuhlii	Rimitara Lorikeet	H	H	U	**L**
Vini peruviana	Blue Lorikeet	H	H	U	**L**
Vini stepheni	Henderson Lorikeet	H	H	U	**L**
Vini ultramarina	Ultramarine Lorikeet	H	H	U	**L**
Vireo altiloquus	Black-whiskered Vireo	H	L	H	**L**
Vireo atricapilla	Black-capped Vireo	H	L	H	**L**
Vireo bairdi	Cozumel Vireo	H	H	H	**H**
Vireo bellii	Bell's Vireo	H	L	H	**L**
Vireo brevipennis	Slaty Vireo	L	U	L	**L**
Vireo caribaeus	San Andres Vireo	H	H	U	**L**
Vireo carmioli	Yellow-winged Vireo	H	H	H	**H**
Vireo cassinii	Cassin's Vireo	H	L	H	**L**
Vireo crassirostris	Thick-billed Vireo	H	L	L	**L**
Vireo flavifrons	Yellow-throated Vireo	L	L	L	**L**
Vireo flavoviridis	Yellow-green Vireo	L	L	H	**L**
Vireo gilvus	Warbling Vireo	H	L	L	**L**
Vireo gracilirostris	Noronha Vireo	H	H	U	**L**
Vireo griseus	White-eyed Vireo	H	L	L	**L**
Vireo gundlachii	Cuban Vireo	U	L	H	**L**
Vireo huttoni	Hutton's Vireo	H	H	L	**L**
Vireo hypochryseus	Golden Vireo	L	U	H	**L**
Vireo latimeri	Puerto Rican Vireo	H	L	H	**L**
Vireo leucophrys	Brown-capped Vireo	L	U	L	**L**
Vireo magister	Yucatan Vireo	H	H	L	**L**
Vireo masteri	Choco Vireo	H	H	H	**H**
Vireo modestus	Jamaican Vireo	H	L	H	**L**
Vireo nanus	Flat-billed Vireo	H	H	H	**H**
Vireo nelsoni	Dwarf Vireo	L	U	L	**L**
Vireo olivaceus	Red-eyed Vireo	H	L	L	**L**
Vireo osburni	Blue Mountain Vireo	H	U	H	**L**
Vireo pallens	Mangrove Vireo	L	L	H	**L**
Vireo philadelphicus	Philadelphia Vireo	H	L	L	**L**
Vireo plumbeus	Plumbeous Vireo	H	L	L	**L**
Vireo solitarius	Blue-headed Vireo	H	L	L	**L**
Vireo vicinior	Grey Vireo	H	L	H	**L**
Vireolanius eximius	Yellow-browed Shrike-vireo	H	H	H	**H**
Vireolanius leucotis	Slaty-capped Shrike-vireo	H	H	H	**H**
Vireolanius melitophrys	Chestnut-sided Shrike-vireo	H	H	L	**L**
Vireolanius pulchellus	Green Shrike-vireo	L	U	H	**L**
Volatinia jacarina	Blue-black Grassquit	L	L	L	**L**
Vultur gryphus	Andean Condor	H	H	L	**L**
Wetmorethraupis sterrhopteron	Orange-throated Tanager	H	H	H	**H**
Wilsonia canadensis	Canada Warbler	L	L	L	**L**

Wilsonia citrina	Hooded Warbler	L	L	H	**L**
Wilsonia pusilla	Wilson's Warbler	H	L	L	**L**
Woodfordia lacertosa	Sanford's White-eye	H	U	H	**L**
Woodfordia superciliosa	Bare-eyed White-eye	H	H	H	**H**
Xanthocephalus xanthocephalus	Yellow-headed Blackbird	H	L	L	**L**
Xanthomyza phrygia	Regent Honeyeater	H	H	L	**L**
Xanthopsar flavus	Saffron-cowled Blackbird	H	L	H	**L**
Xanthotis flaviventer	Tawny-breasted Honeyeater	H	H	L	**L**
Xanthotis macleayanus	Macleay's Honeyeater	U	H	H	**L**
Xanthotis polygrammus	Spotted Honeyeater	H	U	L	**L**
Xanthotis provocator	Kadavu Honeyeater	H	H	L	**L**
Xema sabini	Sabine's Gull	H	H	L	**L**
Xenerpestes minlosi	Double-banded Greytail	H	H	H	**H**
Xenerpestes singularis	Equatorial Greytail	H	H	L	**L**
Xenicus gilviventris	South Island Wren	H	L	H	**L**
Xenocopsychus ansorgei	Angola Cave-chat	U	H	L	**L**
Xenodacnis parina	Tit-like Dacnis	U	U	H	**L**
Xenoglaux loweryi	Long-whiskered Owlet	H	H	L	**L**
Xenoligea montana	White-winged Warbler	H	H	H	**H**
Xenoperdix udzungwensis	Udzungwa Forest-partridge	H	H	H	**H**
Xenopipo atronitens	Black Manakin	H	U	H	**L**
Xenopipo flavicapilla	Yellow-headed Manakin	H	H	H	**H**
Xenopipo holochlora	Green Manakin	H	H	H	**H**
Xenopipo unicolor	Jet Manakin	H	H	H	**H**
Xenopipo uniformis	Olive Manakin	H	H	H	**H**
Xenopirostris damii	Van Dam's Vanga	H	H	H	**H**
Xenopirostris polleni	Pollen's Vanga	H	H	H	**H**
Xenopirostris xenopirostris	Lafresnaye's Vanga	H	H	L	**L**
Xenops milleri	Rufous-tailed Xenops	H	H	H	**H**
Xenops minutus	Plain Xenops	H	H	H	**H**
Xenops rutilans	Streaked Xenops	H	H	L	**L**
Xenops tenuirostris	Slender-billed Xenops	H	H	H	**H**
Xenopsaris albinucha	White-naped Xenopsaris	H	L	U	**L**
Xenornis setifrons	Spiny-faced Antshrike	H	H	L	**L**
Xenospingus concolor	Slender-billed Finch	H	U	H	**L**
Xenospiza baileyi	Sierra Madre Sparrow	H	H	L	**L**
Xenotriccus callizonus	Belted Flycatcher	H	H	H	**H**
Xenotriccus mexicanus	Pileated Flycatcher	L	L	H	**L**
Xenus cinereus	Terek Sandpiper	H	H	H	**H**
Xiphidiopicus percussus	Cuban Green Woodpecker	H	L	H	**L**
Xiphirhynchus superciliaris	Slender-billed Scimitar-babbler	H	L	U	**L**
Xiphocolaptes albicollis	White-throated	H	H	L	**L**

	Woodcreeper				
Xiphocolaptes falcirostris	Moustached Woodcreeper	H	U	L	**L**
Xiphocolaptes major	Great Rufous Woodcreeper	H	L	L	**L**
Xiphocolaptes promeropirhynchus	Strong-billed Woodcreeper	L	U	L	**L**
Xipholena atropurpurea	White-winged Cotinga	H	H	H	**H**
Xipholena lamellipennis	White-tailed Cotinga	H	H	H	**H**
Xipholena punicea	Pompadour Cotinga	H	H	H	**H**
Xiphorhynchus elegans	Elegant Woodcreeper	H	H	L	**L**
Xiphorhynchus erythropygius	Spotted Woodcreeper	H	H	L	**L**
Xiphorhynchus flavigaster	Ivory-billed Woodcreeper	H	L	H	**L**
Xiphorhynchus fuscus	Lesser Woodcreeper	H	L	L	**L**
Xiphorhynchus guttatus	Buff-throated Woodcreeper	H	H	H	**H**
Xiphorhynchus kienerii	Zimmer's Woodcreeper	H	H	H	**H**
Xiphorhynchus lachrymosus	Black-striped Woodcreeper	H	H	L	**L**
Xiphorhynchus obsoletus	Striped Woodcreeper	H	H	H	**H**
Xiphorhynchus ocellatus	Ocellated Woodcreeper	H	U	L	**L**
Xiphorhynchus pardalotus	Chestnut-rumped Woodcreeper	H	H	H	**H**
Xiphorhynchus picus	Straight-billed Woodcreeper	H	H	H	**H**
Xiphorhynchus spixii	Spix's Woodcreeper	H	H	H	**H**
Xiphorhynchus susurrans	Cocoa Woodcreeper	U	H	H	**L**
Xiphorhynchus triangularis	Olive-backed Woodcreeper	H	H	L	**L**
Xolmis cinereus	Grey Monjita	U	L	L	**L**
Xolmis coronatus	Black-crowned Monjita	H	L	L	**L**
Xolmis dominicanus	Black-and-white Monjita	H	L	H	**L**
Xolmis irupero	White Monjita	H	L	L	**L**
Xolmis pyrope	Fire-eyed Diucon	U	L	U	**L**
Xolmis rubetra	Rusty-backed Monjita	H	H	H	**H**
Xolmis salinarum	Salinas Monjita	H	U	H	**L**
Xolmis velatus	White-rumped Monjita	U	U	L	**L**
Yuhina bakeri	White-naped Yuhina	H	H	H	**H**
Yuhina brunneiceps	Taiwan Yuhina	H	H	H	**H**
Yuhina castaniceps	Striated Yuhina	H	H	H	**H**
Yuhina diademata	White-collared Yuhina	H	H	U	**L**
Yuhina everetti	Chestnut-crested Yuhina	H	H	H	**H**
Yuhina flavicollis	Whiskered Yuhina	H	H	U	**L**
Yuhina gularis	Stripe-throated Yuhina	H	H	U	**L**
Yuhina humilis	Burmese Yuhina	H	H	L	**L**
Yuhina nigrimenta	Black-chinned Yuhina	H	H	U	**L**
Yuhina occipitalis	Rufous-vented Yuhina	H	H	U	**L**
Yuhina torqueola	Indochinese Yuhina	U	U	U	**L**

Zaratornis stresemanni	White-cheeked Cotinga	H	L	H	**L**
Zavattariornis stresemanni	Ethiopian Bush-crow	H	H	L	**L**
Zebrilus undulatus	Zigzag Heron	H	H	H	**H**
Zeledonia coronata	Wrenthrush	H	H	H	**H**
Zenaida asiatica	White-winged Dove	L	H	H	**L**
Zenaida auriculata	Eared Dove	U	H	U	**L**
Zenaida aurita	Zenaida Dove	L	H	L	**L**
Zenaida galapagoensis	Galapagos Dove	H	H	H	**H**
Zenaida graysoni	Socorro Dove	H	H	L	**L**
Zenaida macroura	Mourning Dove	H	H	L	**L**
Zenaida meloda	West Peruvian Dove	H	H	H	**H**
Zimmerius bolivianus	Bolivian Tyrannulet	H	H	H	**H**
Zimmerius chrysops	Golden-faced Tyrannulet	U	U	H	**L**
Zimmerius cinereicapilla	Red-billed Tyrannulet	H	H	H	**H**
Zimmerius gracilipes	Slender-footed Tyrannulet	H	H	U	**L**
Zimmerius improbus	Venezuelan Tyrannulet	H	H	H	**H**
Zimmerius vilissimus	Paltry Tyrannulet	L	H	H	**L**
Zimmerius villarejoi	Mishana Tyrannulet	H	H	H	**H**
Zimmerius viridiflavus	Peruvian Tyrannulet	H	H	H	**H**
Zonerodius heliosylus	Forest Bittern	H	H	L	**L**
Zonotrichia albicollis	White-throated Sparrow	H	L	L	**L**
Zonotrichia atricapilla	Golden-crowned Sparrow	H	L	H	**L**
Zonotrichia capensis	Rufous-collared Sparrow	L	L	L	**L**
Zonotrichia leucophrys	White-crowned Sparrow	H	L	L	**L**
Zonotrichia querula	Harris's Sparrow	H	H	L	**L**
Zoonavena grandidieri	Malagasy Spinetail	U	H	U	**L**
Zoonavena sylvatica	White-rumped Spinetail	H	H	H	**H**
Zoonavena thomensis	Sao Tome Spinetail	H	H	L	**L**
Zoothera andromedae	Sunda Thrush	H	H	U	**L**
Zoothera camaronensis	Black-eared Ground-thrush	H	H	H	**H**
Zoothera cinerea	Ashy Thrush	H	U	L	**L**
Zoothera citrina	Orange-headed Thrush	U	L	L	**L**
Zoothera crossleyi	Crossley's Ground-thrush	H	H	L	**L**
Zoothera dauma	Eurasian Scaly Thrush	U	L	H	**L**
Zoothera dixoni	Long-tailed Thrush	H	H	U	**L**
Zoothera dohertyi	Chestnut-backed Thrush	H	H	H	**H**
Zoothera dumasi	Buru Thrush	H	H	H	**H**
Zoothera erythronota	Red-backed Thrush	H	H	H	**H**
Zoothera everetti	Everett's Thrush	H	H	H	**H**
Zoothera gurneyi	Orange Ground-thrush	H	H	L	**L**
Zoothera guttata	Spotted Ground-thrush	H	L	L	**L**
Zoothera heinei	Russet-tailed Thrush	H	H	U	**L**
Zoothera imbricata	Sri Lanka Scaly Thrush	H	H	H	**H**
Zoothera interpres	Chestnut-capped Thrush	H	L	H	**L**

Zoothera joiceyi	Seram Thrush	H	H	H	**H**
Zoothera leucolaema	Enggano Thrush	H	H	H	**H**
Zoothera lunulata	Bassian Thrush	H	H	U	**L**
Zoothera machiki	Fawn-breasted Thrush	H	H	L	**L**
Zoothera margaretae	Makira Thrush	H	H	L	**L**
Zoothera marginata	Dark-sided Thrush	H	H	U	**L**
Zoothera mendeni	Red-and-black Thrush	H	H	L	**L**
Zoothera mollissima	Plain-backed Thrush	U	L	U	**L**
Zoothera monticola	Long-billed Thrush	H	H	U	**L**
Zoothera naevia	Varied Thrush	H	L	H	**L**
Zoothera oberlaenderi	Forest Ground-thrush	H	H	H	**H**
Zoothera peronii	Orange-banded Thrush	H	H	L	**L**
Zoothera piaggiae	Abyssinian Ground-thrush	H	H	U	**L**
Zoothera pinicola	Aztec Thrush	H	H	L	**L**
Zoothera princei	Grey Ground-thrush	H	H	L	**L**
Zoothera schistacea	Slaty-backed Thrush	H	H	L	**L**
Zoothera sibirica	Siberian Thrush	H	L	U	**L**
Zoothera spiloptera	Spot-winged Thrush	U	L	H	**L**
Zoothera talaseae	New Britain Thrush	H	H	H	**H**
Zoothera tanganjicae	Kivu Ground-thrush	H	H	L	**L**
Zoothera turipavae	Guadalcanal Thrush	H	H	H	**H**
Zoothera wardii	Pied Thrush	H	L	U	**L**
Zosterops abyssinicus	White-breasted White-eye	U	L	L	**L**
Zosterops albogularis	White-chested White-eye	H	H	U	**L**
Zosterops anomalus	Lemon-throated White-eye	U	U	H	**L**
Zosterops atricapilla	Black-capped White-eye	H	H	H	**H**
Zosterops atriceps	Creamy-throated White-eye	H	U	H	**L**
Zosterops atrifrons	Black-crowned White-eye	U	U	U	**L**
Zosterops borbonicus	Mascarene Grey White-eye	L	U	H	**L**
Zosterops buruensis	Buru Yellow White-eye	U	U	H	**L**
Zosterops ceylonensis	Sri Lanka White-eye	H	H	H	**H**
Zosterops chloris	Lemon-bellied White-eye	U	U	U	**L**
Zosterops chloronothus	Mauritius Olive White-eye	H	H	H	**H**
Zosterops cinereus	Grey-brown White-eye	H	U	H	**L**
Zosterops citrinella	Ashy-bellied White-eye	U	L	U	**L**
Zosterops consobrinorum	Pale-bellied White-eye	H	U	H	**L**
Zosterops conspicillatus	Bridled White-eye	H	H	H	**H**
Zosterops erythropleurus	Chestnut-flanked White-eye	H	U	U	**L**
Zosterops everetti	Everett's White-eye	U	U	U	**L**
Zosterops explorator	Layard's White-eye	H	H	L	**L**
Zosterops ficedulinus	Sao Tome White-eye	H	H	L	**L**
Zosterops finschii	Dusky White-eye	H	H	H	**H**
Zosterops flavifrons	Yellow-fronted White-eye	H	U	H	**L**
Zosterops flavus	Javan White-eye	H	U	H	**L**
Zosterops fuscicapilla	Capped White-eye	U	U	U	**L**

Zosterops grayi	Pearl-bellied White-eye	H	U	L	**L**
Zosterops griseotinctus	Louisiade White-eye	H	H	L	**L**
Zosterops griseovirescens	Annobon White-eye	H	H	U	**L**
Zosterops hypolais	Plain White-eye	H	H	U	**L**
Zosterops hypoxanthus	Black-headed White-eye	H	H	H	**H**
Zosterops inornatus	Large Lifu White-eye	H	H	H	**H**
Zosterops japonicus	Japanese White-eye	U	L	H	**L**
Zosterops kuehni	Ambon Yellow White-eye	H	U	H	**L**
Zosterops kulambangrae	Solomons White-eye	H	H	H	**H**
Zosterops lateralis	Silvereye	H	L	L	**L**
Zosterops luteirostris	Splendid White-eye	H	H	U	**L**
Zosterops luteus	Australian Yellow White-eye	H	L	H	**L**
Zosterops maderaspatanus	Madagascar White-eye	U	L	U	**L**
Zosterops mayottensis	Mayotte White-eye	H	H	H	**H**
Zosterops meeki	White-throated White-eye	H	H	H	**H**
Zosterops metcalfii	Yellow-throated White-eye	H	U	H	**L**
Zosterops meyeni	Lowland White-eye	U	U	U	**L**
Zosterops minor	Black-fronted White-eye	U	U	U	**L**
Zosterops minutus	Small Lifu White-eye	U	H	H	**L**
Zosterops modestus	Seychelles White-eye	H	H	L	**L**
Zosterops montanus	Mountain White-eye	H	U	H	**L**
Zosterops mouroniensis	Mount Karthala White-eye	H	H	H	**H**
Zosterops murphyi	Hermit White-eye	H	H	H	**H**
Zosterops mysorensis	Biak White-eye	H	H	H	**H**
Zosterops natalis	Christmas White-eye	H	H	U	**L**
Zosterops nehrkorni	Sangihe White-eye	H	H	H	**H**
Zosterops nigrorum	Golden-yellow White-eye	H	U	U	**L**
Zosterops novaeguineae	New Guinea White-eye	H	H	U	**L**
Zosterops oleagineus	Yap Olive White-eye	H	H	U	**L**
Zosterops olivaceus	Reunion Olive White-eye	H	H	H	**H**
Zosterops pallidus	Pale White-eye	H	L	H	**L**
Zosterops palpebrosus	Oriental White-eye	U	L	L	**L**
Zosterops poliogastrus	Montane White-eye	U	L	L	**L**
Zosterops rendovae	Grey-throated White-eye	H	H	H	**H**
Zosterops rennellianus	Rennell White-eye	H	H	H	**H**
Zosterops rotensis	Rota Bridled White-eye	H	H	U	**L**
Zosterops salvadorii	Enggano White-eye	H	H	H	**H**
Zosterops samoensis	Samoan White-eye	H	H	U	**L**
Zosterops sanctaecrucis	Santa Cruz White-eye	H	U	H	**L**
Zosterops semperi	Caroline Islands White-eye	H	U	H	**L**
Zosterops senegalensis	African Yellow White-eye	U	L	L	**L**
Zosterops splendidus	Ranongga White-eye	H	H	H	**H**
Zosterops stalkeri	Seram White-eye	H	H	U	**L**
Zosterops stresemanni	Malaita White-eye	H	U	H	**L**

Zosterops tenuirostris	Slender-billed White-eye	H	H	U	**L**
Zosterops uropygialis	Golden-bellied White-eye	H	U	L	**L**
Zosterops vaughani	Pemba White-eye	H	H	H	**H**
Zosterops vellalavella	Banded White-eye	H	H	H	**H**
Zosterops wallacei	Yellow-spectacled White-eye	H	U	H	**L**
Zosterops xanthochroa	Green-backed White-eye	H	H	H	**H**
