# Supplementary material for: Identifying the World's Most Climate Change Vulnerable Species: A Systematic Trait-Based Assessment of all Birds, Amphibians and Corals
Source: PLoS One. 2013 Jun 12;8(6):e65427. doi: 10.1371/journal.pone.0065427 (PMC3680427; doi:10.1371/journal.pone.0065427)
Supplement: Appendix B — Climate change vulnerability scores for amphibian species. (PDF) [file pone.0065427.s037.pdf]

Appendix B:
Climate change vulnerability assessments by species for **amphibians**

*Assessments of overall vulnerability are based on optimistic assumptions for missing trait information.

Species	SENSI-TIVITY	UN-ADAPTA-BILITY	EXPO-SURE	**OVERALL VULNERA-BILITY***
Acanthixalus sonjae	H	H	L	**L**
Acanthixalus spinosus	H	H	L	**L**
Acris crepitans	H	L	L	**L**
Acris gryllus	H	L	L	**L**
Adelastes hylonomos	H	H	H	**H**
Adelophryne adiastola	H	L	L	**L**
Adelophryne baturitensis	H	H	L	**L**
Adelophryne gutturosa	H	L	H	**L**
Adelophryne maranguapensis	H	L	L	**L**
Adelophryne pachydactyla	H	L	H	**L**
Adelotus brevis	H	L	L	**L**
Adelphobates castaneoticus	H	H	H	**H**
Adelphobates galactonotus	H	H	H	**H**
Adelphobates quinquevittatus	H	H	L	**L**
Adenomus dasi	H	H	H	**H**
Adenomus kandianus	H	H	U	**L**
Adenomus kelaartii	L	H	L	**L**
Afrixalus aureus	H	H	L	**L**
Afrixalus brachycnemis	H	H	L	**L**
Afrixalus clarkei	L	H	L	**L**
Afrixalus crotalus	H	H	L	**L**
Afrixalus delicatus	L	H	L	**L**
Afrixalus dorsalis	H	H	L	**L**
Afrixalus dorsimaculatus	H	H	L	**L**
Afrixalus enseticola	L	H	L	**L**
Afrixalus equatorialis	H	H	L	**L**
Afrixalus fornasini	L	H	L	**L**
Afrixalus fulvovittatus	H	H	L	**L**
Afrixalus knysnae	H	H	H	**H**
Afrixalus lacteus	L	H	L	**L**
Afrixalus laevis	H	H	L	**L**
Afrixalus leucostictus	H	H	H	**H**
Eleutherodactylus darlingtoni	H	H	H	**H**

Eleutherodactylus dennisi	L	H	H	**L**
Eleutherodactylus dilatus	H	H	L	**L**
Eleutherodactylus dimidiatus	L	H	L	**L**
Eleutherodactylus dixoni	H	H	L	**L**
Eleutherodactylus dolomedes	H	H	H	**H**
Eleutherodactylus eileenae	H	H	L	**L**
Austrochaperina yelaensis	H	L	L	**L**
Babina adenopleura	L	H	L	**L**
Babina chapaensis	L	L	L	**L**
Babina daunchina	L	L	H	**L**
Babina holsti	L	H	H	**L**
Babina lini	H	L	H	**L**
Babina okinavana	L	H	H	**L**
Babina pleuraden	H	H	L	**L**
Afrixalus lindholmi	H	H	H	**H**
Afrixalus morerei	H	H	L	**L**
Afrixalus nigeriensis	H	H	L	**L**
Afrixalus orophilus	H	H	L	**L**
Afrixalus osorioi	H	H	L	**L**
Afrixalus paradorsalis	H	H	L	**L**
Afrixalus quadrivittatus	H	H	L	**L**
Afrixalus schneideri	H	H	H	**H**
Afrixalus septentrionalis	H	H	H	**H**
Afrixalus spinifrons	H	H	L	**L**
Afrixalus stuhlmanni	H	H	L	**L**
Afrixalus sylvaticus	H	H	L	**L**
Afrixalus uluguruensis	L	H	H	**L**
Afrixalus upembae	H	L	H	**L**
Afrixalus vibekensis	H	H	L	**L**
Afrixalus vittiger	H	H	L	**L**
Afrixalus weidholzi	H	H	L	**L**
Afrixalus wittei	H	H	L	**L**
Agalychnis annae	H	L	H	**L**
Agalychnis callidryas	H	H	H	**H**
Agalychnis litodryas	H	L	H	**L**
Agalychnis moreletii	H	L	H	**L**
Agalychnis saltator	H	H	H	**H**
Agalychnis spurrelli	H	H	L	**L**
Aglyptodactylus laticeps	L	L	L	**L**
Aglyptodactylus madagascariensis	L	L	H	**L**
Aglyptodactylus securifer	L	L	L	**L**
Albericus brunhildae	L	H	L	**L**
Albericus darlingtoni	H	H	H	**H**
Albericus exclamitans	H	H	L	**L**
Albericus fafniri	H	H	H	**H**

Albericus gudrunae	H	L	H	**L**
Albericus gunnari	H	H	H	**H**
Albericus laurini	H	L	H	**L**
Albericus rhenaurum	H	H	H	**H**
Albericus sanguinopictus	H	H	H	**H**
Albericus siegfriedi	H	H	U	**L**
Albericus swanhildae	H	H	H	**H**
Albericus tuberculus	H	H	H	**H**
Albericus valkuriarum	H	H	H	**H**
Albericus variegatus	H	H	U	**L**
Alexteroon hypsiphonus	H	H	L	**L**
Alexteroon jynx	L	H	L	**L**
Alexteroon obstetricans	H	H	L	**L**
Allobates alessandroi	H	L	H	**L**
Allobates bromelicola	H	H	H	**H**
Allobates brunneus	H	H	H	**H**
Allobates caeruleodactylus	H	L	H	**L**
Allobates caribe	H	L	H	**L**
Allobates cepedai	H	L	H	**L**
Allobates chalcopis	H	H	H	**H**
Allobates conspicuus	H	L	L	**L**
Allobates crombiei	H	L	H	**L**
Allobates femoralis	H	H	H	**H**
Allobates fratisenescus	H	L	H	**L**
Allobates fuscellus	H	L	L	**L**
Allobates gasconi	H	L	L	**L**
Allobates goianus	H	L	L	**L**
Allobates granti	H	H	H	**H**
Allobates humilis	H	H	H	**H**
Allobates insperatus	H	H	H	**H**
Allobates juanii	H	H	H	**H**
Allobates kingsburyi	L	H	L	**L**
Allobates mandelorum	L	H	H	**L**
Allobates marchesianus	H	H	L	**L**
Allobates masniger	H	L	H	**L**
Allobates mcdiarmidi	L	H	H	**L**
Allobates melanolaemus	H	L	H	**L**
Allobates myersi	H	H	L	**L**
Allobates nidicola	H	H	H	**H**
Allobates niputidea	H	H	H	**H**
Allobates olfersioides	H	L	H	**L**
Allobates ornatus	H	L	H	**L**
Allobates picachos	H	H	L	**L**
Allobates pittieri	H	H	H	**H**
Allobates ranoides	H	H	H	**H**

Allobates rufulus	H	H	H	**H**
Allobates sanmartini	H	L	H	**L**
Allobates spumaponens	H	H	H	**H**
Allobates subfolionidificans	H	L	L	**L**
Allobates sumtuosus	H	L	H	**L**
Allobates talamancae	H	H	L	**L**
Allobates trilineatus	H	H	H	**H**
Allobates undulatus	H	L	H	**L**
Allobates vanzolinius	H	L	L	**L**
Allobates wayuu	H	H	H	**H**
Allobates zaparo	H	H	H	**H**
Allopaa hazarensis	H	L	H	**L**
Allophryne ruthveni	H	L	H	**L**
Alsodes australis	U	L	H	**L**
Alsodes barrioi	L	L	L	**L**
Alsodes gargola	H	L	L	**L**
Alsodes hugoi	U	L	L	**L**
Alsodes igneus	U	L	H	**L**
Alsodes kaweshkari	H	L	H	**L**
Alsodes laevis	H	L	U	**L**
Alsodes montanus	H	L	H	**L**
Alsodes monticola	H	L	H	**L**
Alsodes nodosus	H	L	L	**L**
Alsodes pehuenche	H	L	H	**L**
Alsodes tumultuosus	H	L	H	**L**
Alsodes valdiviensis	U	L	L	**L**
Alsodes vanzolinii	L	L	L	**L**
Alsodes verrucosus	U	L	H	**L**
Alsodes vittatus	H	L	H	**L**
Altigius alios	H	L	H	**L**
Altiphrynoides malcolmi	H	H	H	**H**
Altiphrynoides osgoodi	H	H	L	**L**
Alytes cisternasii	H	L	L	**L**
Alytes dickhilleni	H	H	L	**L**
Alytes maurus	H	L	H	**L**
Alytes muletensis	H	H	H	**H**
Alytes obstetricans	H	L	L	**L**
Ambystoma altamirani	L	L	L	**L**
Ambystoma amblycephalum	L	L	L	**L**
Ambystoma andersoni	H	H	L	**L**
Ambystoma annulatum	H	L	L	**L**
Ambystoma barbouri	H	L	L	**L**
Ambystoma bombypellum	L	L	L	**L**
Ambystoma californiense	H	L	L	**L**
Ambystoma cingulatum	H	L	L	**L**

Ambystoma dumerilii	H	H	L	**L**
Ambystoma flavipiperatum	H	H	L	**L**
Ambystoma gracile	L	L	L	**L**
Ambystoma granulosum	H	L	L	**L**
Ambystoma jeffersonianum	H	L	L	**L**
Ambystoma laterale	H	L	L	**L**
Ambystoma leorae	L	L	H	**L**
Babina psaltes	U	H	U	**L**
Babina subaspera	L	H	H	**L**
Balebreviceps hillmani	H	H	H	**H**
Barbourula busuangensis	H	H	L	**L**
Barbourula kalimantanensis	H	H	H	**H**
Barycholos pulcher	L	L	H	**L**
Barycholos ternetzi	U	L	L	**L**
Barygenys atra	H	H	L	**L**
Barygenys cheesmanae	H	L	H	**L**
Barygenys exsul	H	H	L	**L**
Barygenys flavigularis	H	L	H	**L**
Barygenys maculata	H	H	H	**H**
Barygenys nana	L	H	H	**L**
Barygenys parvula	H	H	H	**H**
Batrachophrynus brachydactylus	H	L	H	**L**
Batrachophrynus macrostomus	H	H	H	**H**
Batrachoseps attenuatus	L	H	L	**L**
Batrachoseps campi	H	H	H	**H**
Batrachoseps diabolicus	H	H	H	**H**
Batrachoseps gabrieli	H	L	L	**L**
Batrachoseps gavilanensis	H	H	L	**L**
Batrachoseps gregarius	H	H	H	**H**
Batrachoseps incognitus	H	L	L	**L**
Batrachoseps kawia	H	L	L	**L**
Ambystoma lermaense	H	H	L	**L**
Ambystoma mabeei	H	L	L	**L**
Ambystoma macrodactylum	H	L	H	**L**
Ambystoma maculatum	H	L	L	**L**
Ambystoma mexicanum	H	L	L	**L**
Ambystoma opacum	H	L	L	**L**
Ambystoma ordinarium	L	L	L	**L**
Ambystoma rivulare	U	L	L	**L**
Ambystoma rosaceum	L	L	L	**L**
Ambystoma silvense	U	L	L	**L**
Ambystoma talpoideum	H	L	L	**L**
Ambystoma taylori	H	H	L	**L**
Ambystoma texanum	H	L	L	**L**
Ambystoma tigrinum	H	L	L	**L**

Ambystoma velasci	H	L	H	**L**
Ameerega andina	U	L	H	**L**
Ameerega bassleri	H	H	H	**H**
Ameerega bilinguis	H	H	H	**H**
Ameerega boliviana	L	H	H	**L**
Ameerega braccata	U	H	L	**L**
Ameerega cainarachi	H	H	H	**H**
Ameerega erythromos	H	L	H	**L**
Ameerega flavopicta	U	H	L	**L**
Ameerega hahneli	H	L	H	**L**
Ameerega ingeri	H	H	H	**H**
Ameerega labialis	H	U	U	**L**
Ameerega macero	H	H	H	**H**
Ameerega maculata	H	L	U	**L**
Ameerega parvula	H	H	H	**H**
Ameerega petersi	H	H	H	**H**
Ameerega picta	L	H	L	**L**
Ameerega planipaleae	H	H	H	**H**
Ameerega pongoensis	H	L	H	**L**
Ameerega pulchripecta	H	L	H	**L**
Ameerega rubriventris	H	L	H	**L**
Ameerega silverstonei	H	L	H	**L**
Ameerega simulans	H	H	H	**H**
Ameerega smaragdina	H	L	H	**L**
Ameerega trivittata	H	H	H	**H**
Ameerega yungicola	H	H	H	**H**
Amietia amieti	H	L	H	**L**
Amietia angolensis	H	L	L	**L**
Amietia desaegeri	H	L	H	**L**
Amietia dracomontana	H	L	L	**L**
Amietia fuscigula	H	L	L	**L**
Amietia inyangae	H	H	H	**H**
Amietia johnstoni	L	H	L	**L**
Amietia lubrica	H	L	H	**L**
Amietia ruwenzorica	H	L	H	**L**
Amietia tenuoplicata	H	L	L	**L**
Amietia vandijki	H	L	H	**L**
Amietia vertebralis	H	L	L	**L**
Amietia viridireticulata	U	L	L	**L**
Amietia wittei	U	L	H	**L**
Amietophrynus asmarae	H	H	L	**L**
Amietophrynus blanfordii	H	H	L	**L**
Amietophrynus brauni	L	L	L	**L**
Amietophrynus buchneri	H	L	L	**L**
Amietophrynus camerunensis	H	L	L	**L**

Amietophrynus chudeaui	H	L	U	**L**
Amietophrynus cristiglans	H	H	L	**L**
Amietophrynus danielae	H	H	L	**L**
Amietophrynus djohongensis	H	L	L	**L**
Amietophrynus fuliginatus	L	L	L	**L**
Amietophrynus funereus	L	L	L	**L**
Amietophrynus garmani	H	H	L	**L**
Amietophrynus gracilipes	L	L	L	**L**
Amietophrynus gutturalis	L	L	L	**L**
Amietophrynus kassasii	H	L	L	**L**
Amietophrynus kerinyagae	H	H	L	**L**
Amietophrynus kisoloensis	L	L	H	**L**
Amietophrynus langanoensis	H	L	H	**L**
Amietophrynus latifrons	L	H	L	**L**
Amietophrynus lemairii	H	H	L	**L**
Amietophrynus maculatus	L	L	L	**L**
Amietophrynus pantherinus	H	H	H	**H**
Amietophrynus pardalis	H	H	L	**L**
Amietophrynus perreti	H	H	L	**L**
Amietophrynus poweri	H	H	L	**L**
Amietophrynus rangeri	H	H	L	**L**
Amietophrynus reesi	H	H	H	**H**
Amietophrynus regularis	H	L	L	**L**
Amietophrynus steindachneri	H	L	L	**L**
Amietophrynus superciliaris	H	H	L	**L**
Amietophrynus taiensis	H	L	L	**L**
Amietophrynus togoensis	H	L	L	**L**
Amietophrynus tuberosus	H	L	L	**L**
Amietophrynus turkanae	H	L	H	**L**
Amietophrynus urunguensis	U	L	H	**L**
Amietophrynus villiersi	L	L	L	**L**
Amietophrynus vittatus	H	H	L	**L**
Amietophrynus xeros	H	L	H	**L**
Amolops aniqiaoensis	H	L	H	**L**
Amolops archotaphus	L	L	L	**L**
Amolops bellulus	U	L	L	**L**
Amolops caelumnoctis	U	L	H	**L**
Amolops chakrataensis	H	L	H	**L**
Amolops chunganensis	L	L	H	**L**
Amolops compotrix	U	L	L	**L**
Amolops cremnobatus	L	L	H	**L**
Amolops cucae	U	L	H	**L**
Amolops daiyunensis	L	L	L	**L**
Amolops formosus	L	L	H	**L**
Amolops gerbillus	L	L	H	**L**

Amolops granulosus	L	L	H	**L**
Amolops hainanensis	L	H	L	**L**
Amolops hongkongensis	L	L	H	**L**
Amolops iriodes	U	L	H	**L**
Amolops jaunsari	H	L	H	**L**
Amolops jinjiangensis	L	L	H	**L**
Amolops kangtingensis	L	L	L	**L**
Amolops kaulbacki	H	L	H	**L**
Amolops larutensis	H	L	L	**L**
Amolops liangshanensis	U	L	H	**L**
Amolops lifanensis	L	L	H	**L**
Amolops loloensis	L	L	H	**L**
Amolops longimanus	H	L	L	**L**
Amolops mantzorum	L	L	H	**L**
Amolops marmoratus	L	L	H	**L**
Amolops medogensis	H	L	H	**L**
Amolops mengyangensis	L	L	H	**L**
Amolops monticola	L	L	H	**L**
Amolops nepalicus	U	L	H	**L**
Amolops panhai	L	L	L	**L**
Amolops ricketti	L	L	L	**L**
Amolops spinapectoralis	U	L	L	**L**
Amolops torrentis	L	H	L	**L**
Amolops tuberodepressus	L	L	L	**L**
Amolops viridimaculatus	L	L	L	**L**
Amolops vitreus	U	L	H	**L**
Amolops wuyiensis	L	L	L	**L**
Amphiuma means	H	L	L	**L**
Amphiuma pholeter	H	L	H	**L**
Amphiuma tridactylum	H	L	L	**L**
Anaxyrus americanus	H	H	L	**L**
Anaxyrus baxteri	H	H	L	**L**
Anaxyrus boreas	H	L	H	**L**
Anaxyrus californicus	H	L	L	**L**
Anaxyrus canorus	H	L	L	**L**
Anaxyrus cognatus	H	L	H	**L**
Anaxyrus compactilis	H	L	H	**L**
Anaxyrus debilis	H	L	H	**L**
Anaxyrus exsul	H	H	L	**L**
Anaxyrus fowleri	H	L	L	**L**
Anaxyrus hemiophrys	H	L	L	**L**
Anaxyrus houstonensis	H	H	H	**H**
Anaxyrus kelloggi	H	L	H	**L**
Anaxyrus mexicanus	L	L	L	**L**
Anaxyrus microscaphus	H	L	H	**L**

Anaxyrus nelsoni	H	H	H	**H**
Anaxyrus punctatus	H	L	H	**L**
Anaxyrus quercicus	H	H	L	**L**
Anaxyrus retiformis	H	L	H	**L**
Anaxyrus speciosus	H	L	L	**L**
Anaxyrus terrestris	H	L	L	**L**
Anaxyrus woodhousii	H	L	L	**L**
Andinophryne atelopoides	H	L	H	**L**
Andinophryne colomai	H	H	H	**H**
Andinophryne olallai	H	H	H	**H**
Andrias davidianus	L	L	H	**L**
Andrias japonicus	L	L	L	**L**
Aneides aeneus	H	H	L	**L**
Aneides ferreus	H	H	L	**L**
Aneides flavipunctatus	H	H	L	**L**
Aneides hardii	H	H	H	**H**
Aneides lugubris	L	H	L	**L**
Aneides vagrans	H	H	L	**L**
Anhydrophryne hewitti	H	H	L	**L**
Anhydrophryne rattrayi	H	H	L	**L**
Annandia delacouri	U	L	H	**L**
Anodonthyla boulengerii	L	H	H	**L**
Anodonthyla hutchisoni	H	H	H	**H**
Anodonthyla montana	H	H	L	**L**
Anodonthyla moramora	H	H	L	**L**
Anodonthyla nigrigularis	U	L	H	**L**
Anodonthyla rouxae	H	H	H	**H**
Anomaloglossus atopoglossus	H	L	H	**L**
Anomaloglossus ayarzaguenai	U	L	H	**L**
Anomaloglossus baeobatrachus	H	L	H	**L**
Anomaloglossus beebei	H	H	H	**H**
Anomaloglossus breweri	H	H	H	**H**
Anomaloglossus degranvillei	H	H	H	**H**
Anomaloglossus guanayensis	U	L	H	**L**
Anomaloglossus kaiei	H	H	H	**H**
Anomaloglossus lacrimosus	H	L	H	**L**
Anomaloglossus murisipanensis	H	H	H	**H**
Anomaloglossus parimae	H	L	H	**L**
Anomaloglossus parkerae	U	L	H	**L**
Anomaloglossus praderioi	H	L	H	**L**
Anomaloglossus roraima	H	L	H	**L**
Anomaloglossus shrevei	U	L	H	**L**
Anomaloglossus stepheni	H	H	L	**L**
Anomaloglossus tamacuarensis	H	L	H	**L**
Anomaloglossus tepuyensis	U	L	H	**L**

Anomaloglossus triunfo	H	L	H	**L**
Anomaloglossus wothuja	H	L	L	**L**
Anotheca spinosa	L	H	H	**L**
Ansonia albomaculata	L	H	H	**L**
Ansonia endauensis	H	L	L	**L**
Ansonia fuliginea	H	H	H	**H**
Ansonia glandulosa	H	L	H	**L**
Ansonia guibei	H	H	H	**H**
Ansonia hanitschi	L	H	H	**L**
Ansonia inthanon	U	L	L	**L**
Ansonia kraensis	H	L	H	**L**
Ansonia latidisca	H	H	L	**L**
Ansonia latirostra	U	L	L	**L**
Ansonia leptopus	H	H	H	**H**
Ansonia longidigita	L	H	H	**L**
Ansonia malayana	L	H	L	**L**
Ansonia mcgregori	L	H	L	**L**
Ansonia minuta	H	H	L	**L**
Ansonia muelleri	L	H	L	**L**
Ansonia ornata	L	H	H	**L**
Ansonia penangensis	H	H	H	**H**
Ansonia platysoma	L	H	H	**L**
Ansonia rubigina	L	H	H	**L**
Ansonia siamensis	H	H	H	**H**
Ansonia spinulifer	H	H	L	**L**
Ansonia tiomanica	H	H	U	**L**
Ansonia torrentis	H	H	H	**H**
Aparasphenodon bokermanni	H	L	H	**L**
Aparasphenodon brunoi	L	L	L	**L**
Aparasphenodon venezolanus	H	L	H	**L**
Aphantophryne minuta	H	L	H	**L**
Aphantophryne pansa	H	H	H	**H**
Aphantophryne sabini	H	H	H	**H**
Aplastodiscus albofrenatus	H	L	L	**L**
Aplastodiscus albosignatus	H	L	L	**L**
Aplastodiscus arildae	H	L	L	**L**
Aplastodiscus callipygius	L	L	L	**L**
Aplastodiscus cavicola	H	L	L	**L**
Aplastodiscus cochranae	H	L	L	**L**
Aplastodiscus ehrhardti	H	L	L	**L**
Aplastodiscus eugenioi	H	L	H	**L**
Aplastodiscus flumineus	H	L	H	**L**
Aplastodiscus ibirapitanga	H	L	H	**L**
Aplastodiscus leucopygius	H	L	L	**L**
Aplastodiscus musicus	U	L	H	**L**

Aplastodiscus perviridis	H	L	L	**L**
Aplastodiscus sibilatus	H	L	H	**L**
Aplastodiscus weygoldti	H	L	L	**L**
Arcovomer passarellii	L	H	L	**L**
Arenophryne rotunda	H	H	L	**L**
Argenteohyla siemersi	H	L	L	**L**
Arlequinus krebsi	H	H	L	**L**
Aromobates alboguttatus	H	H	H	**H**
Aromobates capurinensis	H	L	H	**L**
Aromobates duranti	H	H	H	**H**
Aromobates haydeeae	L	H	H	**L**
Aromobates leopardalis	H	H	H	**H**
Aromobates mayorgai	H	H	H	**H**
Aromobates meridensis	H	H	H	**H**
Aromobates molinarii	H	H	H	**H**
Aromobates nocturnus	H	H	H	**H**
Aromobates orostoma	H	H	H	**H**
Aromobates saltuensis	H	H	H	**H**
Aromobates serranus	H	H	H	**H**
Arthroleptella bicolor	H	H	H	**H**
Arthroleptella drewesii	H	L	H	**L**
Arthroleptella landdrosia	H	H	L	**L**
Arthroleptella lightfooti	H	H	L	**L**
Arthroleptella ngongoniensis	H	H	L	**L**
Arthroleptella subvoce	H	L	H	**L**
Arthroleptella villiersi	H	H	H	**H**
Arthroleptis adelphus	H	H	L	**L**
Arthroleptis adolfifriederici	H	H	L	**L**
Arthroleptis affinis	L	H	L	**L**
Arthroleptis bivittatus	U	H	U	**L**
Arthroleptis brevipes	U	L	U	**L**
Arthroleptis carquejai	H	H	H	**H**
Arthroleptis crusculum	H	H	L	**L**
Arthroleptis discodactylus	H	L	U	**L**
Arthroleptis francei	L	H	L	**L**
Arthroleptis hematogaster	H	H	L	**L**
Arthroleptis lameerei	H	H	U	**L**
Arthroleptis lonnbergi	H	L	L	**L**
Arthroleptis loveridgei	H	L	H	**L**
Arthroleptis milletihorsini	U	U	U	**L**
Arthroleptis mossoensis	H	L	L	**L**
Arthroleptis nikeae	H	H	L	**L**
Arthroleptis nimbaensis	H	L	L	**L**
Arthroleptis phrynoides	H	L	U	**L**
Arthroleptis poecilonotus	L	H	L	**L**

Arthroleptis pyrrhoscelis	H	H	L	**L**
Arthroleptis reichei	H	H	L	**L**
Arthroleptis schubotzi	H	H	L	**L**
Arthroleptis spinalis	H	L	H	**L**
Arthroleptis stenodactylus	L	H	L	**L**
Arthroleptis stridens	H	H	L	**L**
Arthroleptis sylvaticus	H	H	L	**L**
Arthroleptis taeniatus	H	H	L	**L**
Arthroleptis tanneri	L	H	L	**L**
Arthroleptis troglodytes	L	H	H	**L**
Arthroleptis tuberosus	H	L	L	**L**
Arthroleptis variabilis	H	H	L	**L**
Arthroleptis vercammeni	H	L	H	**L**
Arthroleptis wahlbergii	H	H	L	**L**
Arthroleptis xenochirus	L	H	L	**L**
Arthroleptis xenodactyloides	L	H	L	**L**
Arthroleptis xenodactylus	L	H	L	**L**
Arthroleptis zimmeri	H	H	L	**L**
Ascaphus montanus	H	H	H	**H**
Ascaphus truei	L	H	L	**L**
Assa darlingtoni	H	H	L	**L**
Asterophrys leucopus	H	H	H	**H**
Asterophrys turpicola	L	H	L	**L**
Astylosternus batesi	H	L	L	**L**
Astylosternus diadematus	L	L	L	**L**
Astylosternus fallax	H	L	L	**L**
Astylosternus laurenti	L	L	L	**L**
Astylosternus montanus	L	L	L	**L**
Astylosternus nganhanus	H	H	L	**L**
Astylosternus occidentalis	H	L	L	**L**
Astylosternus perreti	H	H	L	**L**
Astylosternus ranoides	L	H	H	**L**
Astylosternus rheophilus	L	L	L	**L**
Astylosternus schioetzi	H	L	H	**L**
Atelognathus ceii	H	L	H	**L**
Atelognathus grandisonae	H	H	H	**H**
Atelognathus jeinimenensis	H	H	H	**H**
Atelognathus nitoi	H	L	L	**L**
Atelognathus patagonicus	H	L	L	**L**
Atelognathus praebasalticus	H	L	L	**L**
Atelognathus reverberii	H	L	H	**L**
Atelognathus salai	H	L	H	**L**
Atelognathus solitarius	H	L	H	**L**
Atelopus andinus	H	L	H	**L**
Atelopus angelito	H	L	H	**L**

Atelopus arsyecue	H	H	H	**H**
Atelopus arthuri	H	L	H	**L**
Atelopus balios	H	L	H	**L**
Atelopus bomolochos	H	L	H	**L**
Atelopus boulengeri	H	L	H	**L**
Atelopus carauta	H	L	L	**L**
Atelopus carbonerensis	H	L	H	**L**
Atelopus carrikeri	H	H	H	**H**
Atelopus certus	H	L	L	**L**
Atelopus chiriquiensis	H	L	H	**L**
Atelopus chocoensis	H	H	H	**H**
Atelopus chrysocorallus	H	L	H	**L**
Atelopus coynei	H	L	H	**L**
Atelopus cruciger	H	L	H	**L**
Atelopus dimorphus	H	H	H	**H**
Atelopus ebenoides	H	L	H	**L**
Atelopus elegans	H	H	H	**H**
Atelopus epikeisthos	H	L	L	**L**
Atelopus erythropus	H	L	H	**L**
Atelopus eusebianus	H	L	H	**L**
Atelopus exiguus	H	L	H	**L**
Atelopus famelicus	H	L	L	**L**
Atelopus farci	H	L	H	**L**
Atelopus flavescens	H	L	H	**L**
Atelopus franciscus	H	L	H	**L**
Atelopus galactogaster	H	H	H	**H**
Atelopus glyphus	H	L	L	**L**
Atelopus guanujo	H	L	H	**L**
Atelopus guitarraensis	H	L	H	**L**
Atelopus halihelos	H	L	H	**L**
Atelopus ignescens	H	L	U	**L**
Atelopus laetissimus	H	H	H	**H**
Atelopus limosus	H	L	H	**L**
Atelopus longibrachius	H	L	H	**L**
Atelopus longirostris	H	L	U	**L**
Atelopus lozanoi	H	L	H	**L**
Atelopus lynchi	H	L	H	**L**
Atelopus mandingues	H	L	H	**L**
Atelopus mindoensis	H	L	H	**L**
Atelopus minutulus	H	L	H	**L**
Atelopus mittermeieri	L	L	H	**L**
Atelopus monohernandezii	H	L	H	**L**
Atelopus mucubajiensis	H	L	H	**L**
Atelopus muisca	H	L	H	**L**
Atelopus nahumae	H	H	H	**H**

Atelopus nanay	H	L	H	**L**
Atelopus nepiozomus	H	L	H	**L**
Atelopus nicefori	H	L	H	**L**
Atelopus onorei	H	L	H	**L**
Atelopus oxyrhynchus	H	L	H	**L**
Atelopus pachydermus	H	L	L	**L**
Atelopus palmatus	H	L	L	**L**
Atelopus pedimarmoratus	H	L	H	**L**
Atelopus peruensis	H	L	H	**L**
Atelopus petersi	H	L	H	**L**
Atelopus petriruizi	H	L	L	**L**
Atelopus pictiventris	H	L	L	**L**
Atelopus pinangoi	H	L	H	**L**
Atelopus planispina	H	L	L	**L**
Atelopus pulcher	H	L	H	**L**
Atelopus pyrodactylus	H	H	L	**L**
Atelopus quimbaya	H	L	H	**L**
Atelopus reticulatus	H	L	H	**L**
Atelopus sanjosei	H	L	H	**L**
Atelopus seminiferus	H	L	H	**L**
Atelopus senex	H	L	H	**L**
Atelopus sernai	H	L	H	**L**
Atelopus simulatus	H	L	H	**L**
Atelopus siranus	H	L	H	**L**
Atelopus sonsonensis	H	L	H	**L**
Atelopus sorianoi	H	L	H	**L**
Atelopus spumarius	H	L	H	**L**
Atelopus spurrelli	H	L	H	**L**
Atelopus subornatus	H	L	H	**L**
Atelopus tamaensis	H	L	H	**L**
Atelopus tricolor	H	L	H	**L**
Atelopus varius	H	L	H	**L**
Atelopus vogli	H	L	U	**L**
Atelopus walkeri	H	H	H	**H**
Atelopus zeteki	H	L	L	**L**
Atopophrynus syntomopus	H	L	H	**L**
Atretochoana eiselti	H	H	U	**L**
Atylodes genei	H	H	H	**H**
Atympanophrys giganticus	U	L	U	**L**
Atympanophrys shapingensis	U	L	U	**L**
Aubria masako	H	H	L	**L**
Aubria occidentalis	H	L	L	**L**
Aubria subsigillata	L	L	L	**L**
Austrochaperina adamantina	H	H	H	**H**
Austrochaperina adelphe	H	H	L	**L**

Austrochaperina aquilonia	H	H	H	**H**
Austrochaperina archboldi	H	H	L	**L**
Austrochaperina basipalmata	H	H	H	**H**
Austrochaperina blumi	H	H	H	**H**
Austrochaperina brevipes	H	H	H	**H**
Austrochaperina derongo	L	H	H	**L**
Austrochaperina fryi	H	H	L	**L**
Austrochaperina gracilipes	H	H	L	**L**
Austrochaperina guttata	H	H	H	**H**
Austrochaperina hooglandi	L	H	H	**L**
Austrochaperina kosarek	H	H	H	**H**
Austrochaperina macrorhyncha	H	H	H	**H**
Austrochaperina mehelyi	U	L	H	**L**
Austrochaperina novaebritanniae	L	H	H	**L**
Austrochaperina palmipes	L	H	L	**L**
Austrochaperina parkeri	H	L	L	**L**
Austrochaperina pluvialis	L	H	H	**L**
Austrochaperina polysticta	H	L	H	**L**
Austrochaperina rivularis	L	H	H	**L**
Austrochaperina robusta	L	H	H	**L**
Austrochaperina septentrionalis	H	L	H	**L**
Eleutherodactylus auriculatoides	H	H	H	**H**
Eleutherodactylus auriculatus	L	H	L	**L**
Eleutherodactylus bakeri	L	H	H	**L**
Eleutherodactylus barlagnei	H	H	H	**H**
Eleutherodactylus bartonsmithi	H	H	H	**H**
Eleutherodactylus blairhedgesi	L	H	L	**L**
Eleutherodactylus bresslerae	H	H	L	**L**
Eleutherodactylus brevirostris	L	H	H	**L**
Eleutherodactylus brittoni	L	H	H	**L**
Eleutherodactylus caribe	H	H	H	**H**
Eleutherodactylus casparii	L	H	H	**L**
Eleutherodactylus cavernicola	H	H	L	**L**
Eleutherodactylus chlorophenax	L	H	H	**L**
Eleutherodactylus cochranae	L	H	L	**L**
Eleutherodactylus cooki	H	H	H	**H**
Eleutherodactylus coqui	H	H	H	**H**
Eleutherodactylus corona	H	H	H	**H**
Eleutherodactylus counouspeus	L	H	H	**L**
Eleutherodactylus cubanus	L	H	L	**L**
Eleutherodactylus cundalli	L	H	L	**L**
Eleutherodactylus cuneatus	L	H	L	**L**
Eleutherodactylus cystignathoides	H	H	L	**L**
Batrachoseps luciae	H	H	L	**L**
Batrachoseps major	H	H	L	**L**

Batrachoseps minor	H	L	L	**L**
Batrachoseps nigriventris	H	H	L	**L**
Batrachoseps pacificus	H	H	H	**H**
Batrachoseps regius	H	H	H	**H**
Batrachoseps relictus	H	L	L	**L**
Batrachoseps robustus	H	H	L	**L**
Batrachoseps simatus	H	H	L	**L**
Batrachoseps stebbinsi	H	H	L	**L**
Batrachoseps wrighti	H	H	L	**L**
Batrachuperus cochranae	L	H	L	**L**
Batrachuperus karlschmidti	H	H	L	**L**
Batrachuperus londongensis	H	H	H	**H**
Batrachuperus pinchonii	H	H	L	**L**
Batrachuperus taibaiensis	H	L	L	**L**
Batrachuperus tibetanus	H	H	L	**L**
Batrachuperus yenyuanensis	H	H	L	**L**
Batrachyla antartandica	L	H	L	**L**
Batrachyla fitzroya	H	H	L	**L**
Batrachyla leptopus	L	H	L	**L**
Batrachyla nibaldoi	U	H	H	**L**
Batrachyla taeniata	L	H	L	**L**
Batrachylodes elegans	H	H	H	**H**
Batrachylodes gigas	H	H	H	**H**
Batrachylodes mediodiscus	H	H	H	**H**
Batrachylodes minutus	H	H	H	**H**
Batrachylodes montanus	H	H	L	**L**
Batrachylodes trossulus	H	H	L	**L**
Batrachylodes vertebralis	H	H	H	**H**
Batrachylodes wolfi	H	H	H	**H**
Blommersia blommersae	L	H	L	**L**
Blommersia domerguei	L	H	L	**L**
Blommersia grandisonae	L	H	H	**L**
Blommersia kely	L	H	L	**L**
Blommersia sarotra	U	H	H	**L**
Blommersia wittei	H	H	L	**L**
Boehmantis microtympanum	L	H	H	**L**
Bokermannohyla ahenea	U	H	U	**L**
Bokermannohyla alvarengai	H	L	L	**L**
Bokermannohyla astartea	H	H	L	**L**
Bokermannohyla caramaschii	H	L	L	**L**
Bokermannohyla carvalhoi	L	L	H	**L**
Bokermannohyla circumdata	H	L	L	**L**
Bokermannohyla claresignata	H	L	L	**L**
Bokermannohyla clepsydra	H	L	L	**L**
Bokermannohyla diamantina	H	L	H	**L**

Bokermannohyla feioi	U	L	L	**L**
Bokermannohyla gouveai	H	L	L	**L**
Bokermannohyla hylax	H	L	L	**L**
Bokermannohyla ibitiguara	H	L	L	**L**
Bokermannohyla ibitipoca	U	L	L	**L**
Bokermannohyla itapoty	H	L	H	**L**
Bokermannohyla izecksohni	H	H	L	**L**
Bokermannohyla langei	H	L	L	**L**
Bokermannohyla lucianae	H	L	H	**L**
Bokermannohyla luctuosa	H	L	L	**L**
Bokermannohyla martinsi	H	L	L	**L**
Bokermannohyla nanuzae	H	L	L	**L**
Bokermannohyla oxente	H	L	H	**L**
Bokermannohyla pseudopseudis	H	L	L	**L**
Bokermannohyla ravida	H	L	L	**L**
Bokermannohyla saxicola	H	L	L	**L**
Bokermannohyla sazimai	H	L	L	**L**
Bokermannohyla vulcaniae	H	L	L	**L**
Bolitoglossa adspersa	L	H	H	**L**
Bolitoglossa alberchi	L	H	L	**L**
Bolitoglossa altamazonica	H	H	L	**L**
Bolitoglossa alvaradoi	H	H	H	**H**
Bolitoglossa anthracina	H	L	H	**L**
Bolitoglossa biseriata	H	H	L	**L**
Bolitoglossa borburata	H	H	H	**H**
Bolitoglossa bramei	H	H	H	**H**
Bolitoglossa capitana	H	H	L	**L**
Bolitoglossa carri	H	H	H	**H**
Bolitoglossa celaque	H	H	H	**H**
Bolitoglossa cerroensis	H	H	H	**H**
Bolitoglossa chica	H	H	H	**H**
Bolitoglossa colonnea	L	H	H	**L**
Bolitoglossa compacta	H	H	H	**H**
Bolitoglossa conanti	H	H	H	**H**
Bolitoglossa copia	H	L	L	**L**
Bolitoglossa cuchumatana	H	H	H	**H**
Bolitoglossa cuna	H	L	H	**L**
Bolitoglossa decora	H	H	H	**H**
Bolitoglossa diaphora	H	H	H	**H**
Bolitoglossa digitigrada	H	H	H	**H**
Bolitoglossa diminuta	H	H	H	**H**
Bolitoglossa dofleini	H	H	H	**H**
Bolitoglossa dunni	H	H	H	**H**
Bolitoglossa engelhardti	H	H	H	**H**
Bolitoglossa epimela	U	L	H	**L**

Bolitoglossa equatoriana	H	H	H	**H**
Bolitoglossa flavimembris	H	H	H	**H**
Bolitoglossa flaviventris	L	H	H	**L**
Bolitoglossa franklini	H	H	H	**H**
Bolitoglossa gomezi	H	H	H	**H**
Bolitoglossa gracilis	H	H	H	**H**
Bolitoglossa guaramacalensis	H	H	H	**H**
Bolitoglossa hartwegi	L	H	H	**L**
Bolitoglossa heiroreias	H	H	H	**H**
Bolitoglossa helmrichi	L	H	H	**L**
Bolitoglossa hermosa	L	H	L	**L**
Bolitoglossa hiemalis	H	H	H	**H**
Bolitoglossa hypacra	H	H	H	**H**
Bolitoglossa jacksoni	H	L	H	**L**
Bolitoglossa lignicolor	H	H	H	**H**
Bolitoglossa lincolni	H	H	H	**H**
Bolitoglossa longissima	H	H	H	**H**
Bolitoglossa lozanoi	H	L	L	**L**
Bolitoglossa macrinii	L	H	L	**L**
Bolitoglossa magnifica	H	H	H	**H**
Bolitoglossa marmorea	L	H	H	**L**
Bolitoglossa medemi	H	H	H	**H**
Bolitoglossa meliana	H	H	H	**H**
Bolitoglossa mexicana	L	H	H	**L**
Bolitoglossa minutula	H	H	H	**H**
Bolitoglossa mombachoensis	H	H	H	**H**
Bolitoglossa morio	L	H	H	**L**
Bolitoglossa mulleri	L	H	H	**L**
Bolitoglossa nicefori	L	H	L	**L**
Bolitoglossa nigrescens	H	H	H	**H**
Bolitoglossa oaxacensis	U	L	H	**L**
Bolitoglossa obscura	H	H	H	**H**
Bolitoglossa occidentalis	L	H	L	**L**
Bolitoglossa odonnelli	L	H	H	**L**
Bolitoglossa oresbia	H	H	H	**H**
Bolitoglossa orestes	H	H	H	**H**
Bolitoglossa palmata	H	H	H	**H**
Bolitoglossa pandi	H	H	H	**H**
Bolitoglossa paraensis	H	L	H	**L**
Bolitoglossa peruviana	H	H	H	**H**
Bolitoglossa pesrubra	H	H	H	**H**
Bolitoglossa phalarosoma	H	L	L	**L**
Bolitoglossa platydactyla	L	H	H	**L**
Bolitoglossa porrasorum	L	H	H	**L**
Bolitoglossa ramosi	L	H	H	**L**

Bolitoglossa riletti	L	H	L	**L**
Bolitoglossa robusta	L	H	H	**L**
Bolitoglossa rostrata	H	H	H	**H**
Bolitoglossa rufescens	L	H	H	**L**
Bolitoglossa salvinii	L	H	H	**L**
Bolitoglossa savagei	H	H	H	**H**
Bolitoglossa schizodactyla	L	H	L	**L**
Bolitoglossa silverstonei	H	H	H	**H**
Bolitoglossa sima	H	H	H	**H**
Bolitoglossa sombra	H	H	H	**H**
Bolitoglossa sooyorum	H	H	H	**H**
Bolitoglossa spongai	H	H	H	**H**
Bolitoglossa striatula	H	H	H	**H**
Bolitoglossa stuarti	U	L	H	**L**
Bolitoglossa subpalmata	H	H	H	**H**
Bolitoglossa synoria	L	H	H	**L**
Bolitoglossa tatamae	H	H	H	**H**
Bolitoglossa taylori	H	L	L	**L**
Bolitoglossa vallecula	L	H	H	**L**
Bolitoglossa veracrucis	L	H	H	**L**
Bolitoglossa walkeri	H	H	L	**L**
Bolitoglossa yucatana	L	H	L	**L**
Bolitoglossa zapoteca	H	L	L	**L**
Bombina bombina	H	L	H	**L**
Bombina fortinuptialis	L	H	L	**L**
Bombina lichuanensis	L	H	L	**L**
Bombina maxima	U	H	U	**L**
Bombina microdeladigitora	U	H	U	**L**
Bombina orientalis	L	L	H	**L**
Bombina pachypus	H	L	L	**L**
Bombina variegata	H	L	L	**L**
Boophis albilabris	L	L	H	**L**
Boophis albipunctatus	L	L	L	**L**
Boophis andohahela	U	L	H	**L**
Boophis andreonei	L	L	H	**L**
Boophis anjanaharibeensis	U	L	H	**L**
Boophis ankaratra	L	L	L	**L**
Boophis axelmeyeri	L	L	H	**L**
Boophis blommersae	L	L	H	**L**
Boophis boehmei	L	L	L	**L**
Boophis bottae	L	L	L	**L**
Boophis brachychir	U	L	H	**L**
Boophis burgeri	U	L	L	**L**
Boophis doulioti	L	L	L	**L**
Boophis elenae	U	L	L	**L**

Boophis englaenderi	H	H	H	**H**
Boophis erythrodactylus	L	L	L	**L**
Boophis feonnyala	H	L	H	**L**
Boophis goudotii	L	L	L	**L**
Boophis guibei	L	L	L	**L**
Boophis haematopus	L	H	H	**L**
Boophis hillenii	U	H	H	**L**
Boophis idae	L	L	L	**L**
Boophis jaegeri	H	H	H	**H**
Boophis laurenti	H	L	L	**L**
Boophis liami	U	L	L	**L**
Boophis lichenoides	L	L	H	**L**
Boophis luteus	L	L	H	**L**
Boophis madagascariensis	L	L	H	**L**
Boophis majori	L	L	L	**L**
Boophis mandraka	U	L	L	**L**
Boophis marojezensis	L	L	H	**L**
Boophis microtympanum	L	H	L	**L**
Boophis miniatus	L	L	L	**L**
Boophis occidentalis	L	L	L	**L**
Boophis opisthodon	L	L	H	**L**
Boophis pauliani	L	L	L	**L**
Boophis periegetes	U	L	L	**L**
Boophis picturatus	L	L	L	**L**
Boophis pyrrhus	L	L	H	**L**
Boophis rappiodes	L	L	L	**L**
Boophis reticulatus	L	L	H	**L**
Boophis rhodoscelis	L	L	L	**L**
Boophis rufioculis	L	L	L	**L**
Boophis sambirano	L	L	H	**L**
Boophis schuboeae	U	L	L	**L**
Boophis septentrionalis	U	L	H	**L**
Boophis sibilans	U	L	L	**L**
Boophis solomaso	U	L	H	**L**
Boophis tasymena	L	L	H	**L**
Boophis tephraeomystax	L	L	H	**L**
Boophis viridis	L	L	H	**L**
Boophis vittatus	L	L	H	**L**
Boophis williamsi	L	H	H	**L**
Boophis xerophilus	H	H	L	**L**
Borneophrys edwardinae	H	H	H	**H**
Boulengerula boulengeri	H	H	L	**L**
Boulengerula changamwensis	U	H	L	**L**
Boulengerula denhardti	U	L	U	**L**
Boulengerula fischeri	H	L	L	**L**

Boulengerula niedeni	H	H	L	**L**
Boulengerula taitana	L	H	L	**L**
Boulengerula uluguruensis	L	H	H	**L**
Brachycephalus alipioi	H	L	L	**L**
Brachycephalus brunneus	H	H	L	**L**
Brachycephalus didactylus	H	H	L	**L**
Brachycephalus ephippium	L	H	H	**L**
Brachycephalus ferruginus	H	L	H	**L**
Brachycephalus hermogenesi	H	H	L	**L**
Brachycephalus izecksohni	H	H	L	**L**
Brachycephalus nodoterga	H	L	H	**L**
Brachycephalus pernix	H	H	L	**L**
Brachycephalus pombali	H	L	H	**L**
Brachycephalus vertebralis	H	L	L	**L**
Brachytarsophrys carinense	L	L	L	**L**
Brachytarsophrys chuannanensis	U	L	H	**L**
Brachytarsophrys feae	L	L	H	**L**
Brachytarsophrys intermedia	L	L	L	**L**
Brachytarsophrys platyparietus	L	L	H	**L**
Bradytriton silus	H	H	H	**H**
Brasilotyphlus braziliensis	H	H	H	**H**
Breviceps acutirostris	H	H	H	**H**
Breviceps adspersus	H	H	L	**L**
Breviceps bagginsi	H	L	L	**L**
Breviceps fichus	H	H	L	**L**
Breviceps fuscus	H	H	H	**H**
Breviceps gibbosus	H	H	H	**H**
Breviceps macrops	H	H	H	**H**
Breviceps montanus	H	H	H	**H**
Breviceps mossambicus	L	H	L	**L**
Breviceps namaquensis	H	H	L	**L**
Breviceps poweri	H	H	L	**L**
Breviceps rosei	H	H	H	**H**
Breviceps sopranus	H	H	H	**H**
Breviceps sylvestris	L	H	L	**L**
Breviceps verrucosus	H	H	L	**L**
Bromeliohyla bromeliacia	H	L	H	**L**
Bromeliohyla dendroscarta	H	L	H	**L**
Bryophryne bustamantei	L	H	H	**L**
Bryophryne cophites	L	H	H	**L**
Buergeria buergeri	L	L	L	**L**
Buergeria japonica	H	H	L	**L**
Buergeria oxycephala	L	H	L	**L**
Buergeria robusta	L	H	H	**L**
Bufo ailaoanus	U	L	H	**L**

Bufo andrewsi	H	L	H	**L**
Bufo arabicus	H	H	H	**H**
Bufo aspinius	U	L	L	**L**
Bufo atukoralei	H	H	L	**L**
Bufo bankorensis	H	H	H	**H**
Bufo beddomii	L	L	H	**L**
Bufo brevirostris	H	L	L	**L**
Bufo bufo	H	L	H	**L**
Bufo cryptotympanicus	H	L	L	**L**
Bufo dhufarensis	H	L	L	**L**
Bufo dodsoni	H	L	L	**L**
Bufo gargarizans	H	L	H	**L**
Bufo hololius	H	L	L	**L**
Bufo japonicus	L	H	L	**L**
Bufo kabischi	U	L	H	**L**
Bufo kotagamai	H	H	L	**L**
Bufo koynayensis	H	L	H	**L**
Bufo mauritanicus	H	H	H	**H**
Bufo minshanicus	H	L	L	**L**
Bufo olivaceus	H	L	H	**L**
Bufo pageoti	L	L	L	**L**
Bufo parietalis	H	L	H	**L**
Bufo pentoni	H	L	L	**L**
Bufo scaber	H	L	L	**L**
Bufo scorteccii	H	H	L	**L**
Bufo silentvalleyensis	H	L	H	**L**
Bufo stejnegeri	L	L	H	**L**
Bufo stomaticus	H	L	L	**L**
Bufo stuarti	H	L	H	**L**
Bufo sumatranus	H	L	H	**L**
Bufo tibetanus	H	H	H	**H**
Bufo tihamicus	H	L	H	**L**
Bufo torrenticola	L	H	L	**L**
Bufo tuberculatus	H	H	H	**H**
Bufo valhallae	H	L	U	**L**
Bufo verrucosissimus	H	L	H	**L**
Bufo wolongensis	U	L	H	**L**
Bufoides meghalayanus	L	H	H	**L**
Cacosternum boettgeri	H	L	L	**L**
Cacosternum capense	H	H	H	**H**
Cacosternum karooicum	H	L	L	**L**
Cacosternum leleupi	H	L	L	**L**
Cacosternum namaquense	H	H	L	**L**
Cacosternum nanum	H	H	L	**L**
Cacosternum parvum	H	H	L	**L**

Cacosternum platys	H	H	H	**H**
Cacosternum plimptoni	H	H	L	**L**
Cacosternum poyntoni	H	L	H	**L**
Cacosternum striatum	H	L	L	**L**
Caecilia abitaguae	H	H	H	**H**
Caecilia albiventris	U	H	U	**L**
Caecilia antioquiaensis	H	H	H	**H**
Caecilia armata	H	H	U	**L**
Caecilia attenuata	H	H	H	**H**
Caecilia bokermanni	H	H	H	**H**
Caecilia caribea	H	H	H	**H**
Caecilia corpulenta	U	H	H	**L**
Caecilia crassisquama	H	H	H	**H**
Caecilia degenerata	U	H	H	**L**
Caecilia disossea	H	H	H	**H**
Caecilia dunni	U	H	H	**L**
Caecilia flavopunctata	H	L	H	**L**
Caecilia gracilis	H	L	H	**L**
Caecilia guntheri	H	L	H	**L**
Caecilia inca	H	H	H	**H**
Caecilia isthmica	H	H	U	**L**
Caecilia leucocephala	H	H	H	**H**
Caecilia marcusi	H	H	H	**H**
Caecilia mertensi	H	U	U	**L**
Caecilia nigricans	H	H	L	**L**
Caecilia occidentalis	U	L	H	**L**
Caecilia orientalis	L	L	H	**L**
Caecilia pachynema	U	L	L	**L**
Caecilia perdita	H	H	H	**H**
Caecilia pressula	H	H	H	**H**
Caecilia subdermalis	L	H	L	**L**
Caecilia subnigricans	L	H	L	**L**
Caecilia subterminalis	H	H	U	**L**
Caecilia tentaculata	H	H	H	**H**
Caecilia tenuissima	H	L	H	**L**
Caecilia thompsoni	U	L	H	**L**
Caecilia volcani	U	L	L	**L**
Calamita melanorabdotus	H	L	U	**L**
Calamita quadrilineatus	H	U	U	**L**
Callixalus pictus	H	H	L	**L**
Calluella brooksii	H	L	H	**L**
Calluella flava	H	L	H	**L**
Calluella guttulata	L	L	L	**L**
Calluella minuta	H	L	L	**L**
Calluella smithi	H	L	H	**L**

Calluella volzi	H	L	L	**L**
Calluella yunnanensis	L	L	H	**L**
Callulina kisiwamsitu	H	L	L	**L**
Callulina kreffti	L	L	H	**L**
Callulops boettgeri	H	L	U	**L**
Callulops comptus	L	H	H	**L**
Callulops doriae	L	H	L	**L**
Callulops dubius	H	L	H	**L**
Callulops eurydactylus	U	L	H	**L**
Callulops fuscus	U	L	H	**L**
Callulops glandulosus	H	L	H	**L**
Callulops humicola	H	H	H	**H**
Callulops kopsteini	H	H	H	**H**
Callulops marmoratus	H	L	L	**L**
Callulops personatus	H	H	H	**H**
Callulops pullifer	H	L	H	**L**
Callulops robustus	L	H	L	**L**
Callulops sagittatus	H	L	H	**L**
Callulops slateri	L	H	H	**L**
Callulops stictogaster	H	H	H	**H**
Callulops wilhelmanus	L	H	H	**L**
Calotriton arnoldi	H	L	H	**L**
Calotriton asper	H	H	H	**H**
Calyptocephalella gayi	H	L	L	**L**
Capensibufo rosei	H	H	H	**H**
Capensibufo tradouwi	H	H	H	**H**
Cardioglossa alsco	H	H	L	**L**
Cardioglossa aureoli	H	H	L	**L**
Cardioglossa cyaneospila	U	L	U	**L**
Cardioglossa elegans	L	H	L	**L**
Cardioglossa escalerae	H	H	L	**L**
Cardioglossa gracilis	H	H	L	**L**
Cardioglossa gratiosa	H	H	L	**L**
Cardioglossa leucomystax	H	H	L	**L**
Cardioglossa melanogaster	L	H	L	**L**
Cardioglossa nigromaculata	L	H	H	**L**
Cardioglossa oreas	L	H	H	**L**
Cardioglossa pulchra	L	H	L	**L**
Cardioglossa schioetzi	L	H	L	**L**
Cardioglossa trifasciata	L	H	L	**L**
Cardioglossa venusta	L	H	L	**L**
Caudacaecilia asplenia	H	U	H	**L**
Caudacaecilia larutensis	H	U	H	**L**
Caudacaecilia nigroflava	U	U	U	**L**
Caudacaecilia paucidentula	H	U	H	**L**

Caudacaecilia weberi	H	U	U	**L**
Centrolene acanthidiocephalum	U	L	H	**L**
Centrolene altitudinale	H	L	H	**L**
Centrolene andinum	H	H	H	**H**
Centrolene antioquiense	L	H	H	**L**
Centrolene audax	H	H	H	**H**
Centrolene azulae	H	H	H	**H**
Centrolene bacatum	U	L	H	**L**
Centrolene balionotum	H	H	H	**H**
Centrolene ballux	H	H	H	**H**
Centrolene buckleyi	H	H	H	**H**
Centrolene callistommum	U	L	H	**L**
Centrolene durrellorum	L	H	H	**L**
Centrolene fernandoi	H	H	H	**H**
Centrolene geckoideum	H	H	H	**H**
Centrolene gemmatum	H	H	H	**H**
Centrolene gorzulae	U	L	H	**L**
Centrolene grandisonae	L	H	H	**L**
Centrolene guanacarum	H	L	H	**L**
Centrolene heloderma	H	H	H	**H**
Centrolene hesperium	H	H	H	**H**
Centrolene huilense	H	L	H	**L**
Centrolene hybrida	L	H	H	**L**
Centrolene ilex	H	H	L	**L**
Centrolene lema	U	L	H	**L**
Centrolene lemniscatum	H	L	L	**L**
Centrolene litorale	H	L	H	**L**
Centrolene lynchi	H	H	H	**H**
Centrolene mariaelenae	H	H	L	**L**
Centrolene medemi	H	L	H	**L**
Centrolene muelleri	H	L	L	**L**
Centrolene notostictum	L	H	H	**L**
Centrolene ocellifera	H	L	H	**L**
Centrolene paezorum	H	L	H	**L**
Centrolene papillahallicum	H	L	H	**L**
Centrolene peristictum	H	H	L	**L**
Centrolene petrophilum	L	H	H	**L**
Centrolene pipilatum	H	H	H	**H**
Centrolene prosoblepon	H	H	L	**L**
Centrolene quindianum	L	H	H	**L**
Centrolene robledoi	H	H	H	**H**
Centrolene sanchezi	U	L	L	**L**
Centrolene scirtetes	H	L	H	**L**
Centrolene tayrona	H	H	H	**H**
Centrolene venezuelense	H	H	H	**H**

Ceratobatrachus guentheri	H	H	H	**H**
Ceratophrys aurita	L	H	L	**L**
Ceratophrys calcarata	H	L	L	**L**
Ceratophrys cornuta	H	H	H	**H**
Ceratophrys cranwelli	H	L	L	**L**
Ceratophrys joazeirensis	H	H	L	**L**
Ceratophrys ornata	H	L	H	**L**
Ceratophrys stolzmanni	H	L	H	**L**
Ceratophrys testudo	H	H	U	**L**
Chacophrys pierottii	H	L	L	**L**
Chaperina fusca	L	H	L	**L**
Charadrahyla altipotens	H	L	H	**L**
Charadrahyla chaneque	H	L	L	**L**
Charadrahyla nephila	H	L	L	**L**
Charadrahyla taeniopus	L	L	H	**L**
Charadrahyla trux	H	L	L	**L**
Chiasmocleis alagoanus	H	L	H	**L**
Chiasmocleis albopunctata	H	H	L	**L**
Chiasmocleis anatipes	H	H	H	**H**
Chiasmocleis atlantica	L	H	L	**L**
Chiasmocleis bassleri	H	L	L	**L**
Chiasmocleis capixaba	H	L	L	**L**
Chiasmocleis carvalhoi	H	H	H	**H**
Chiasmocleis centralis	H	L	H	**L**
Chiasmocleis cordeiroi	H	L	H	**L**
Chiasmocleis crucis	H	H	H	**H**
Chiasmocleis gnoma	H	L	H	**L**
Chiasmocleis hudsoni	H	L	H	**L**
Chiasmocleis jimi	H	H	H	**H**
Chiasmocleis leucosticta	L	H	L	**L**
Chiasmocleis magnova	H	L	H	**L**
Chiasmocleis mantiqueira	U	L	H	**L**
Chiasmocleis mehelyi	H	L	L	**L**
Chiasmocleis panamensis	H	L	H	**L**
Chiasmocleis sapiranga	H	H	H	**H**
Chiasmocleis schubarti	H	L	H	**L**
Chiasmocleis shudikarensis	H	L	H	**L**
Chiasmocleis ventrimaculata	H	H	H	**H**
Chioglossa lusitanica	H	H	L	**L**
Chiromantis cherrapunjiae	H	L	H	**L**
Chiromantis doriae	L	H	H	**L**
Chiromantis dudhwaensis	U	H	H	**L**
Chiromantis hansenae	H	H	L	**L**
Chiromantis kelleri	H	L	L	**L**
Chiromantis laevis	H	H	L	**L**

Chiromantis nongkhorensis	L	H	L	**L**
Chiromantis petersii	H	L	L	**L**
Chiromantis punctatus	H	L	L	**L**
Chiromantis rufescens	H	L	L	**L**
Chiromantis samkosensis	H	L	H	**L**
Chiromantis shyamrupus	U	L	H	**L**
Chiromantis simus	L	H	H	**L**
Chiromantis vittatus	L	H	H	**L**
Chiromantis xerampelina	L	L	L	**L**
Chiropterotriton arboreus	H	H	H	**H**
Chiropterotriton chiropterus	H	H	H	**H**
Chiropterotriton chondrostega	H	H	H	**H**
Chiropterotriton cracens	H	H	H	**H**
Chiropterotriton dimidiatus	H	H	L	**L**
Chiropterotriton lavae	H	H	L	**L**
Chiropterotriton magnipes	L	H	H	**L**
Chiropterotriton mosaueri	H	L	H	**L**
Chiropterotriton multidentatus	H	H	L	**L**
Chiropterotriton orculus	H	H	L	**L**
Chiropterotriton priscus	H	H	L	**L**
Chiropterotriton terrestris	H	H	H	**H**
Chlorolius koehleri	H	L	L	**L**
Choerophryne allisoni	H	L	L	**L**
Choerophryne burtoni	H	L	L	**L**
Choerophryne longirostris	H	H	H	**H**
Choerophryne proboscidea	H	H	H	**H**
Choerophryne rostellifer	L	H	H	**L**
Chrysobatrachus cupreonitens	H	H	H	**H**
Chrysopaa sternosignata	H	L	H	**L**
Chthonerpeton arii	H	H	L	**L**
Chthonerpeton braestrupi	H	H	U	**L**
Chthonerpeton exile	H	H	U	**L**
Chthonerpeton indistinctum	H	H	L	**L**
Chthonerpeton noctinectes	U	H	H	**L**
Chthonerpeton onorei	H	H	L	**L**
Chthonerpeton perissodus	H	H	U	**L**
Chthonerpeton viviparum	H	H	L	**L**
Churamiti maridadi	H	H	H	**H**
Clinotarsus alticola	L	L	H	**L**
Clinotarsus curtipes	L	L	H	**L**
Cochranella adenocheira	H	L	L	**L**
Cochranella adiazeta	L	H	H	**L**
Cochranella albomaculata	H	H	H	**H**
Cochranella amelie	H	L	H	**L**
Cochranella antisthenesi	H	H	H	**H**

Cochranella castroviejoi	H	L	H	**L**
Cochranella croceopodes	H	L	H	**L**
Cochranella daidalea	L	H	L	**L**
Cochranella duidaeana	U	L	H	**L**
Cochranella erminea	H	L	H	**L**
Cochranella euhystrix	H	L	H	**L**
Cochranella euknemos	H	H	L	**L**
Cochranella flavopunctata	H	H	H	**H**
Cochranella geijskesi	H	L	H	**L**
Cochranella granulosa	H	H	H	**H**
Cochranella helenae	H	L	H	**L**
Cochranella mache	H	H	H	**H**
Cochranella mariae	H	H	H	**H**
Cochranella megistra	H	H	H	**H**
Cochranella midas	H	H	L	**L**
Cochranella nola	L	H	H	**L**
Cochranella ocellata	L	H	H	**L**
Cochranella orejuela	H	L	L	**L**
Cochranella oyampiensis	H	H	H	**H**
Cochranella phryxa	U	L	H	**L**
Cochranella pulverata	H	H	H	**H**
Cochranella punctulata	L	H	H	**L**
Cochranella puyoensis	H	H	H	**H**
Cochranella ramirezi	H	L	L	**L**
Cochranella resplendens	H	H	H	**H**
Cochranella revocata	H	H	H	**H**
Cochranella ritae	H	L	L	**L**
Cochranella riveroi	H	H	H	**H**
Cochranella savagei	L	H	L	**L**
Cochranella saxiscandens	H	H	H	**H**
Cochranella solitaria	H	L	H	**L**
Cochranella spiculata	L	H	H	**L**
Cochranella spinosa	H	H	L	**L**
Cochranella susatamai	H	H	H	**H**
Cochranella tangarana	H	L	H	**L**
Cochranella vozmedianoi	H	L	H	**L**
Cochranella xanthocheridia	H	H	H	**H**
Colostethus agilis	L	H	H	**L**
Colostethus alacris	H	L	H	**L**
Colostethus brachistriatus	H	L	H	**L**
Colostethus dysprosium	H	L	H	**L**
Colostethus fraterdanieli	L	H	H	**L**
Colostethus fugax	H	L	H	**L**
Colostethus furviventris	H	L	H	**L**
Colostethus imbricolus	H	L	H	**L**

Colostethus inguinalis	H	H	L	**L**
Colostethus jacobuspetersi	L	H	H	**L**
Colostethus latinasus	H	L	L	**L**
Colostethus lynchi	H	L	U	**L**
Colostethus mertensi	L	H	H	**L**
Colostethus panamansis	H	H	L	**L**
Colostethus poecilonotus	H	L	L	**L**
Colostethus pratti	L	H	L	**L**
Colostethus ramirezi	H	L	H	**L**
Colostethus ruthveni	L	H	H	**L**
Colostethus thorntoni	H	L	L	**L**
Colostethus ucumari	L	H	H	**L**
Colostethus yaguara	H	L	L	**L**
Conraua alleni	H	L	L	**L**
Conraua beccarii	L	L	L	**L**
Conraua crassipes	L	L	L	**L**
Conraua derooi	H	L	L	**L**
Conraua goliath	L	L	L	**L**
Conraua robusta	L	L	L	**L**
Cophixalus aenigma	L	H	L	**L**
Cophixalus aimbensis	H	L	L	**L**
Cophixalus ateles	H	L	U	**L**
Cophixalus balbus	H	L	H	**L**
Cophixalus bewaniensis	H	L	H	**L**
Cophixalus biroi	L	H	H	**L**
Cophixalus bombiens	L	H	L	**L**
Cophixalus cheesmanae	L	H	L	**L**
Cophixalus concinnus	L	H	H	**L**
Cophixalus crepitans	H	H	L	**L**
Cophixalus cryptotympanum	H	L	H	**L**
Cophixalus daymani	H	L	H	**L**
Cophixalus exiguus	H	H	L	**L**
Cophixalus hosmeri	L	H	L	**L**
Cophixalus humicola	H	H	L	**L**
Cophixalus infacetus	H	H	H	**H**
Cophixalus kaindiensis	H	L	H	**L**
Cophixalus mcdonaldi	H	H	L	**L**
Cophixalus misimae	H	L	L	**L**
Cophixalus montanus	H	L	U	**L**
Cophixalus monticola	H	H	L	**L**
Cophixalus neglectus	L	H	H	**L**
Cophixalus nubicola	H	H	H	**H**
Cophixalus ornatus	L	H	H	**L**
Cophixalus parkeri	H	H	H	**H**
Cophixalus peninsularis	H	L	L	**L**

Cophixalus pipilans	H	H	L	**L**
Cophixalus pulchellus	H	L	H	**L**
Cophixalus riparius	H	H	H	**H**
Cophixalus saxatilis	L	H	L	**L**
Cophixalus shellyi	L	H	H	**L**
Cophixalus sisyphus	U	L	H	**L**
Cophixalus sphagnicola	L	H	H	**L**
Cophixalus tagulensis	H	L	L	**L**
Cophixalus tetzlaffi	H	L	L	**L**
Cophixalus timidus	H	H	H	**H**
Cophixalus tridactylus	H	H	H	**H**
Cophixalus variabilis	L	H	L	**L**
Cophixalus verecundus	H	H	H	**H**
Cophixalus verrucosus	L	H	L	**L**
Cophixalus zweifeli	H	H	L	**L**
Cophyla berara	H	H	H	**H**
Cophyla occultans	U	H	H	**L**
Cophyla phyllodactyla	L	H	H	**L**
Copiula exspectata	H	H	H	**H**
Copiula fistulans	H	H	L	**L**
Copiula major	H	H	H	**H**
Copiula minor	H	H	L	**L**
Copiula obsti	H	H	H	**H**
Copiula oxyrhina	L	H	L	**L**
Copiula pipiens	H	H	L	**L**
Copiula tyleri	H	H	L	**L**
Corythomantis greeningi	H	L	L	**L**
Craugastor adamastus	H	L	H	**L**
Craugastor alfredi	H	L	H	**L**
Craugastor amniscola	H	L	L	**L**
Craugastor anciano	H	L	H	**L**
Craugastor andi	H	L	H	**L**
Craugastor angelicus	H	L	H	**L**
Craugastor aphanus	H	L	H	**L**
Craugastor augusti	L	L	H	**L**
Craugastor aurilegulus	H	L	H	**L**
Craugastor azueroensis	H	L	L	**L**
Craugastor batrachylus	U	L	H	**L**
Craugastor berkenbuschii	H	L	H	**L**
Craugastor bocourti	H	L	H	**L**
Craugastor bransfordii	H	L	H	**L**
Craugastor brocchi	H	L	H	**L**
Craugastor campbelli	H	L	H	**L**
Craugastor catalinae	H	L	H	**L**
Craugastor chac	L	L	H	**L**

Craugastor charadra	H	L	H	**L**
Craugastor chingopetaca	H	L	H	**L**
Craugastor chrysozetetes	H	L	U	**L**
Craugastor coffeus	L	L	H	**L**
Craugastor crassidigitus	L	L	H	**L**
Craugastor cruzi	H	L	H	**L**
Craugastor cuaquero	H	L	H	**L**
Craugastor cyanochthebius	H	H	H	**H**
Craugastor daryi	H	L	H	**L**
Craugastor decoratus	H	H	H	**H**
Craugastor emcelae	H	H	H	**H**
Craugastor emleni	H	H	H	**H**
Craugastor epochthidius	H	L	H	**L**
Craugastor escoces	H	L	U	**L**
Craugastor fecundus	H	L	H	**L**
Craugastor fitzingeri	H	H	H	**H**
Craugastor fleischmanni	H	L	H	**L**
Craugastor glaucus	H	H	H	**H**
Craugastor gollmeri	L	L	H	**L**
Craugastor greggi	H	L	H	**L**
Craugastor guerreroensis	H	L	L	**L**
Craugastor gulosus	H	H	H	**H**
Craugastor hobartsmithi	L	L	L	**L**
Craugastor inachus	H	L	H	**L**
Craugastor jota	H	L	H	**L**
Craugastor laevissimus	H	H	H	**H**
Craugastor laticeps	H	H	H	**H**
Craugastor lauraster	H	H	H	**H**
Craugastor lineatus	H	L	H	**L**
Craugastor loki	L	L	H	**L**
Craugastor longirostris	L	L	H	**L**
Craugastor matudai	H	L	H	**L**
Craugastor megacephalus	L	H	H	**L**
Craugastor megalotympanum	L	L	H	**L**
Craugastor melanostictus	H	L	H	**L**
Craugastor merendonensis	H	L	H	**L**
Craugastor mexicanus	H	L	L	**L**
Craugastor milesi	H	L	H	**L**
Craugastor mimus	H	L	H	**L**
Craugastor monnichorum	U	L	H	**L**
Craugastor montanus	H	L	H	**L**
Craugastor myllomyllon	U	L	H	**L**
Craugastor nefrens	H	H	H	**H**
Craugastor noblei	H	H	H	**H**
Craugastor obesus	H	L	H	**L**

Craugastor occidentalis	U	L	U	**L**
Craugastor olanchano	H	H	H	**H**
Craugastor omiltemanus	H	H	L	**L**
Craugastor omoaensis	H	L	H	**L**
Craugastor opimus	L	H	L	**L**
Craugastor palenque	H	L	H	**L**
Craugastor pechorum	H	H	H	**H**
Craugastor pelorus	U	L	H	**L**
Craugastor persimilis	L	H	H	**L**
Craugastor phasma	H	L	H	**L**
Craugastor podiciferus	H	H	H	**H**
Craugastor polymniae	H	L	H	**L**
Craugastor polyptychus	H	L	H	**L**
Craugastor pozo	L	L	L	**L**
Craugastor psephosypharus	L	H	H	**L**
Craugastor punctariolus	H	L	L	**L**
Craugastor pygmaeus	L	L	L	**L**
Craugastor raniformis	L	H	L	**L**
Craugastor ranoides	H	L	H	**L**
Craugastor rayo	H	L	H	**L**
Craugastor rhodopis	H	H	L	**L**
Craugastor rhyacobatrachus	H	L	H	**L**
Craugastor rivulus	L	H	H	**L**
Craugastor rostralis	L	H	H	**L**
Craugastor rugosus	L	H	H	**L**
Craugastor rugulosus	H	L	L	**L**
Craugastor rupinius	H	L	H	**L**
Craugastor sabrinus	H	L	H	**L**
Craugastor saltuarius	H	H	H	**H**
Craugastor sandersoni	H	H	H	**H**
Craugastor silvicola	H	L	L	**L**
Craugastor spatulatus	H	H	H	**H**
Craugastor stadelmani	H	L	H	**L**
Craugastor stejnegerianus	H	L	H	**L**
Craugastor stuarti	H	L	H	**L**
Craugastor tabasarae	H	L	L	**L**
Craugastor talamancae	L	L	H	**L**
Craugastor tarahumaraensis	H	H	L	**L**
Craugastor taurus	H	L	H	**L**
Craugastor taylori	U	H	U	**L**
Craugastor trachydermus	H	L	H	**L**
Craugastor underwoodi	H	H	H	**H**
Craugastor uno	H	H	L	**L**
Craugastor vocalis	L	L	H	**L**
Craugastor vulcani	L	H	H	**L**

Craugastor xucanebi	L	H	H	**L**
Craugastor yucatanensis	H	H	L	**L**
Crepidophryne chompipe	H	H	H	**H**
Crepidophryne epiotica	H	L	H	**L**
Crepidophryne guanacaste	H	H	H	**H**
Crinia bilingua	H	H	L	**L**
Crinia deserticola	H	H	L	**L**
Crinia georgiana	H	H	H	**H**
Crinia glauerti	L	H	H	**L**
Crinia insignifera	H	L	L	**L**
Crinia nimbus	H	H	H	**H**
Crinia parinsignifera	H	H	L	**L**
Crinia pseudinsignifera	H	H	H	**H**
Crinia remota	H	H	L	**L**
Crinia riparia	H	H	L	**L**
Crinia signifera	H	L	L	**L**
Crinia sloanei	H	L	L	**L**
Crinia subinsignifera	H	H	H	**H**
Crinia tasmaniensis	H	H	H	**H**
Crinia tinnula	H	H	L	**L**
Crossodactylodes bokermanni	H	H	L	**L**
Crossodactylodes izecksohni	H	L	L	**L**
Crossodactylodes pintoi	U	H	U	**L**
Crossodactylus aeneus	H	L	L	**L**
Crossodactylus bokermanni	H	L	L	**L**
Crossodactylus caramaschii	H	L	L	**L**
Crossodactylus cyclospinus	H	L	L	**L**
Crossodactylus dantei	H	L	H	**L**
Crossodactylus dispar	H	L	L	**L**
Crossodactylus gaudichaudii	H	L	L	**L**
Crossodactylus grandis	H	L	L	**L**
Crossodactylus lutzorum	H	L	H	**L**
Crossodactylus schmidti	H	L	L	**L**
Crossodactylus trachystomus	H	L	H	**L**
Crotaphatrema bornmuelleri	U	U	U	**L**
Crotaphatrema lamottei	H	U	H	**L**
Crotaphatrema tchabalmbaboensis	H	U	L	**L**
Cruziohyla calcarifer	L	H	H	**L**
Cruziohyla craspedopus	H	H	L	**L**
Cryptobatrachus boulengeri	L	H	H	**L**
Cryptobatrachus fuhrmanni	L	L	H	**L**
Cryptobatrachus nicefori	H	L	H	**L**
Cryptobranchus alleganiensis	H	L	L	**L**
Cryptothylax greshoffii	H	H	L	**L**
Cryptothylax minutus	H	L	L	**L**

Cryptotriton adelos	H	H	L	**L**
Cryptotriton alvarezdeltoroi	H	H	H	**H**
Cryptotriton monzoni	H	H	H	**H**
Cryptotriton nasalis	H	H	H	**H**
Cryptotriton veraepacis	H	H	H	**H**
Cryptotriton wakei	H	H	H	**H**
Ctenophryne geayi	H	H	H	**H**
Ctenophryne minor	H	H	H	**H**
Cycloramphus acangatan	H	L	L	**L**
Cycloramphus asper	H	L	L	**L**
Cycloramphus bandeirensis	H	L	H	**L**
Cycloramphus bolitoglossus	H	H	L	**L**
Cycloramphus boraceiensis	H	L	L	**L**
Cycloramphus brasiliensis	H	L	L	**L**
Cycloramphus carvalhoi	H	H	H	**H**
Cycloramphus catarinensis	H	L	L	**L**
Cycloramphus cedrensis	H	L	L	**L**
Cycloramphus diringshofeni	H	H	L	**L**
Cycloramphus dubius	H	L	H	**L**
Cycloramphus duseni	H	L	L	**L**
Cycloramphus eleutherodactylus	H	H	L	**L**
Cycloramphus faustoi	L	H	H	**L**
Cycloramphus fuliginosus	H	L	H	**L**
Cycloramphus granulosus	H	L	L	**L**
Cycloramphus izecksohni	H	L	H	**L**
Cycloramphus jordanensis	H	H	L	**L**
Cycloramphus juimirim	H	L	H	**L**
Cycloramphus lutzorum	H	L	L	**L**
Cycloramphus migueli	H	H	H	**H**
Cycloramphus mirandaribeiroi	H	L	L	**L**
Cycloramphus ohausi	H	L	H	**L**
Cycloramphus rhyakonastes	H	H	L	**L**
Cycloramphus semipalmatus	H	L	H	**L**
Cycloramphus stejnegeri	H	H	H	**H**
Cycloramphus valae	H	L	H	**L**
Cynops chenggongensis	H	L	H	**L**
Cynops cyanurus	L	H	L	**L**
Cynops ensicauda	L	H	H	**L**
Cynops orientalis	L	H	H	**L**
Cynops orphicus	L	H	L	**L**
Cynops pyrrhogaster	L	H	L	**L**
Cynops wolterstorffi	H	H	U	**L**
Dasypops schirchi	H	H	L	**L**
Dendrobates auratus	H	L	L	**L**
Dendrobates leucomelas	H	L	H	**L**

Dendrobates nubeculosus	H	H	H	**H**
Dendrobates tinctorius	H	H	H	**H**
Dendrobates truncatus	L	H	H	**L**
Dendrophryniscus berthalutzae	H	L	L	**L**
Dendrophryniscus bokermanni	H	H	H	**H**
Dendrophryniscus brevipollicatus	H	H	L	**L**
Dendrophryniscus carvalhoi	H	H	L	**L**
Dendrophryniscus leucomystax	L	H	L	**L**
Dendrophryniscus minutus	H	L	H	**L**
Dendrophryniscus stawiarskyi	H	H	L	**L**
Dendropsophus acreanus	H	H	L	**L**
Dendropsophus allenorum	H	H	H	**H**
Dendropsophus amicorum	H	H	H	**H**
Dendropsophus anataliasiasi	H	H	L	**L**
Dendropsophus anceps	H	L	H	**L**
Dendropsophus aperomeus	H	L	L	**L**
Dendropsophus araguaya	H	H	L	**L**
Dendropsophus baileyi	U	H	L	**L**
Dendropsophus battersbyi	H	L	H	**L**
Dendropsophus berthalutzae	L	H	L	**L**
Dendropsophus bifurcus	H	L	L	**L**
Dendropsophus bipunctatus	H	L	H	**L**
Dendropsophus bogerti	L	L	H	**L**
Dendropsophus bokermanni	H	L	L	**L**
Dendropsophus branneri	H	L	L	**L**
Dendropsophus brevifrons	H	H	H	**H**
Dendropsophus cachimbo	H	H	H	**H**
Dendropsophus carnifex	L	L	H	**L**
Dendropsophus cerradensis	H	L	L	**L**
Dendropsophus coffeus	H	H	H	**H**
Dendropsophus columbianus	H	L	H	**L**
Dendropsophus cruzi	H	L	L	**L**
Dendropsophus decipiens	L	L	L	**L**
Dendropsophus delarivai	L	H	H	**L**
Dendropsophus dutrai	H	L	L	**L**
Dendropsophus ebraccatus	L	L	L	**L**
Dendropsophus elegans	L	L	L	**L**
Dendropsophus elianeae	H	L	L	**L**
Dendropsophus garagoensis	H	H	L	**L**
Dendropsophus gaucheri	H	L	H	**L**
Dendropsophus giesleri	L	H	L	**L**
Dendropsophus grandisonae	U	L	U	**L**
Dendropsophus gryllatus	H	H	H	**H**
Dendropsophus haddadi	H	L	H	**L**
Dendropsophus haraldschultzi	H	L	L	**L**

Dendropsophus jimi	H	L	L	**L**
Dendropsophus joannae	H	H	L	**L**
Dendropsophus juliani	H	L	L	**L**
Dendropsophus koechlini	H	L	L	**L**
Dendropsophus labialis	H	H	H	**H**
Dendropsophus leali	H	H	L	**L**
Dendropsophus leucophyllatus	H	H	H	**H**
Dendropsophus limai	H	H	H	**H**
Dendropsophus luteoocellatus	L	H	H	**L**
Dendropsophus marmoratus	H	H	H	**H**
Dendropsophus mathiassoni	H	H	H	**H**
Dendropsophus melanargyreus	H	L	H	**L**
Dendropsophus meridensis	H	H	H	**H**
Dendropsophus meridianus	H	L	L	**L**
Dendropsophus microcephalus	H	H	H	**H**
Dendropsophus microps	H	L	L	**L**
Dendropsophus minimus	H	L	H	**L**
Dendropsophus minusculus	H	L	H	**L**
Dendropsophus minutus	L	H	L	**L**
Dendropsophus miyatai	H	L	L	**L**
Dendropsophus nahdereri	H	L	L	**L**
Dendropsophus nanus	L	L	L	**L**
Dendropsophus novaisi	H	L	L	**L**
Dendropsophus oliveirai	H	H	L	**L**
Dendropsophus padreluna	H	H	H	**H**
Dendropsophus parviceps	H	L	L	**L**
Dendropsophus pauiniensis	H	L	L	**L**
Dendropsophus pelidna	L	L	H	**L**
Dendropsophus phlebodes	H	L	H	**L**
Dendropsophus praestans	H	L	H	**L**
Dendropsophus pseudomeridianus	H	L	L	**L**
Dendropsophus rhea	H	H	L	**L**
Dendropsophus rhodopeplus	H	L	H	**L**
Dendropsophus riveroi	H	H	L	**L**
Dendropsophus robertmertensi	L	L	H	**L**
Dendropsophus rossalleni	H	H	L	**L**
Dendropsophus rubicundulus	H	H	L	**L**
Dendropsophus ruschii	H	L	L	**L**
Dendropsophus sanborni	H	H	L	**L**
Dendropsophus sarayacuensis	H	H	L	**L**
Dendropsophus sartori	L	H	H	**L**
Dendropsophus schubarti	H	H	L	**L**
Dendropsophus seniculus	L	H	L	**L**
Dendropsophus soaresi	H	H	L	**L**
Dendropsophus stingi	H	L	H	**L**

Dendropsophus studerae	H	L	H	**L**
Dendropsophus subocularis	L	H	H	**L**
Dendropsophus timbeba	H	H	H	**H**
Dendropsophus tintinnabulum	H	L	H	**L**
Dendropsophus triangulum	H	L	L	**L**
Dendropsophus tritaeniatus	H	L	L	**L**
Dendropsophus virolinensis	H	L	L	**L**
Dendropsophus walfordi	H	L	H	**L**
Dendropsophus werneri	H	L	L	**L**
Dendropsophus xapuriensis	H	H	L	**L**
Dendropsophus yaracuyanus	H	L	H	**L**
Dendrotriton bromeliacius	H	H	H	**H**
Dendrotriton cuchumatanus	H	H	H	**H**
Dendrotriton megarhinus	H	H	L	**L**
Dendrotriton rabbi	H	H	H	**H**
Dendrotriton sanctibarbarus	H	H	H	**H**
Dendrotriton xolocalcae	H	H	H	**H**
Dermatonotus muelleri	H	L	L	**L**
Dermophis costaricensis	H	H	H	**H**
Dermophis glandulosus	U	H	H	**L**
Dermophis gracilior	U	H	H	**L**
Dermophis mexicanus	L	H	H	**L**
Dermophis oaxacae	H	H	L	**L**
Dermophis occidentalis	H	H	H	**H**
Dermophis parviceps	L	H	H	**L**
Desmognathus abditus	H	H	L	**L**
Desmognathus aeneus	H	H	L	**L**
Desmognathus apalachicolae	H	H	H	**H**
Desmognathus auriculatus	H	H	L	**L**
Desmognathus brimleyorum	H	H	L	**L**
Desmognathus carolinensis	H	H	L	**L**
Desmognathus folkertsi	H	L	H	**L**
Desmognathus fuscus	H	H	L	**L**
Desmognathus imitator	H	H	H	**H**
Desmognathus marmoratus	H	H	L	**L**
Desmognathus monticola	H	H	L	**L**
Desmognathus ochrophaeus	H	H	L	**L**
Desmognathus ocoee	H	H	L	**L**
Desmognathus orestes	H	H	L	**L**
Desmognathus quadramaculatus	H	H	H	**H**
Desmognathus santeetlah	H	H	H	**H**
Desmognathus welteri	H	H	L	**L**
Desmognathus wrighti	H	H	L	**L**
Diaglena spatulata	L	L	L	**L**
Diasporus anthrax	U	H	H	**L**

Diasporus diastema	H	H	H	**H**
Diasporus gularis	H	H	H	**H**
Diasporus hylaeformis	H	H	H	**H**
Diasporus quidditus	H	H	L	**L**
Diasporus tigrillo	H	L	H	**L**
Diasporus tinker	H	H	H	**H**
Diasporus vocator	L	H	L	**L**
Dicamptodon aterrimus	H	L	H	**L**
Dicamptodon copei	L	L	H	**L**
Dicamptodon ensatus	L	H	L	**L**
Dicamptodon tenebrosus	L	L	L	**L**
Didynamipus sjostedti	L	H	H	**L**
Dischidodactylus colonnelloi	H	H	H	**H**
Dischidodactylus duidensis	H	H	H	**H**
Discodeles bufoniformis	H	H	H	**H**
Discodeles guppyi	H	H	L	**L**
Discodeles malukuna	H	L	H	**L**
Discodeles opisthodon	H	L	L	**L**
Discodeles vogti	H	H	H	**H**
Discoglossus galganoi	H	L	L	**L**
Discoglossus jeanneae	H	L	L	**L**
Discoglossus montalentii	H	H	L	**L**
Discoglossus nigriventer	H	H	U	**L**
Discoglossus pictus	H	H	H	**H**
Discoglossus sardus	H	H	L	**L**
Discoglossus scovazzi	H	L	H	**L**
Duellmanohyla chamulae	H	L	L	**L**
Duellmanohyla ignicolor	H	L	L	**L**
Duellmanohyla lythrodes	H	L	H	**L**
Duellmanohyla rufioculis	H	L	H	**L**
Duellmanohyla salvavida	H	L	H	**L**
Duellmanohyla schmidtorum	H	L	L	**L**
Duellmanohyla soralia	H	L	H	**L**
Duellmanohyla uranochroa	H	L	H	**L**
Duttaphrynus crocus	H	L	H	**L**
Duttaphrynus himalayanus	L	L	H	**L**
Duttaphrynus melanostictus	H	L	L	**L**
Duttaphrynus microtympanum	L	H	H	**L**
Duttaphrynus noellerti	L	H	H	**L**
Dyscophus antongilii	L	L	H	**L**
Dyscophus guineti	L	L	H	**L**
Dyscophus insularis	L	L	L	**L**
Echinotriton andersoni	L	H	H	**L**
Echinotriton chinhaiensis	H	H	L	**L**
Ecnomiohyla echinata	H	L	H	**L**

Ecnomiohyla fimbrimembra	H	H	H	**H**
Ecnomiohyla miliaria	L	H	H	**L**
Ecnomiohyla minera	H	H	H	**H**
Ecnomiohyla miotympanum	L	L	H	**L**
Ecnomiohyla phantasmagoria	H	L	H	**L**
Ecnomiohyla salvaje	L	H	H	**L**
Ecnomiohyla thysanota	H	L	H	**L**
Ecnomiohyla tuberculosa	H	L	H	**L**
Ecnomiohyla valancifer	H	L	H	**L**
Edalorhina nasuta	U	L	H	**L**
Edalorhina perezi	H	H	L	**L**
Elachistocleis bicolor	H	L	L	**L**
Elachistocleis erythrogaster	H	L	H	**L**
Elachistocleis ovalis	L	H	L	**L**
Elachistocleis piauiensis	H	H	L	**L**
Elachistocleis skotogaster	U	L	H	**L**
Elachistocleis surinamensis	H	H	H	**H**
Eleutherodactylus abbotti	L	H	H	**L**
Eleutherodactylus acmonis	L	H	H	**L**
Eleutherodactylus adelus	H	H	L	**L**
Eleutherodactylus albipes	H	H	L	**L**
Eleutherodactylus alcoae	L	H	L	**L**
Eleutherodactylus alticola	H	H	H	**H**
Eleutherodactylus amadeus	H	H	H	**H**
Eleutherodactylus amplinympha	H	H	H	**H**
Eleutherodactylus andrewsi	L	H	H	**L**
Eleutherodactylus angustidigitorum	H	H	L	**L**
Eleutherodactylus antillensis	L	H	L	**L**
Eleutherodactylus apostates	L	H	H	**L**
Eleutherodactylus armstrongi	L	H	H	**L**
Eleutherodactylus atkinsi	L	H	L	**L**
Eleutherodactylus audanti	L	H	H	**L**
Eleutherodactylus emiliae	H	H	H	**H**
Eleutherodactylus eneidae	H	H	H	**H**
Eleutherodactylus etheridgei	L	H	L	**L**
Eleutherodactylus eunaster	L	H	H	**L**
Eleutherodactylus flavescens	H	H	H	**H**
Eleutherodactylus fowleri	L	H	H	**L**
Eleutherodactylus furcyensis	L	H	H	**L**
Eleutherodactylus fuscus	H	H	H	**H**
Eleutherodactylus glamyrus	L	H	H	**L**
Eleutherodactylus glandulifer	L	H	H	**L**
Eleutherodactylus glanduliferoides	H	H	H	**H**
Eleutherodactylus glaphycompus	L	H	H	**L**

Eleutherodactylus glaucoreius	L	H	H	**L**
Eleutherodactylus goini	L	H	L	**L**
Eleutherodactylus gossei	L	H	H	**L**
Eleutherodactylus grabhami	H	H	H	**H**
Eleutherodactylus grahami	H	H	H	**H**
Eleutherodactylus grandis	H	H	L	**L**
Eleutherodactylus greyi	L	H	H	**L**
Eleutherodactylus griphus	H	H	H	**H**
Eleutherodactylus gryllus	H	H	H	**H**
Eleutherodactylus guanahacabibes	L	H	L	**L**
Eleutherodactylus guantanamera	L	H	H	**L**
Eleutherodactylus gundlachi	L	H	H	**L**
Eleutherodactylus guttilatus	H	H	H	**H**
Eleutherodactylus haitianus	H	H	H	**H**
Eleutherodactylus hedricki	H	H	H	**H**
Eleutherodactylus heminota	L	H	H	**L**
Eleutherodactylus hypostenor	L	H	H	**L**
Eleutherodactylus iberia	H	H	L	**L**
Eleutherodactylus inoptatus	L	H	H	**L**
Eleutherodactylus intermedius	L	H	H	**L**
Eleutherodactylus interorbitalis	H	L	H	**L**
Eleutherodactylus ionthus	L	H	H	**L**
Eleutherodactylus jamaicensis	L	H	H	**L**
Eleutherodactylus jasperi	H	H	H	**H**
Eleutherodactylus jaumei	H	H	H	**H**
Eleutherodactylus johnstonei	H	H	H	**H**
Eleutherodactylus juanriveroi	H	H	U	**L**
Eleutherodactylus jugans	H	H	H	**H**
Eleutherodactylus junori	H	H	L	**L**
Eleutherodactylus karlschmidti	H	H	H	**H**
Eleutherodactylus klinikowskii	L	H	L	**L**
Eleutherodactylus lamprotes	L	H	H	**L**
Eleutherodactylus leberi	H	H	H	**H**
Eleutherodactylus lentus	H	H	L	**L**
Eleutherodactylus leoncei	H	H	H	**H**
Eleutherodactylus leprus	H	H	H	**H**
Eleutherodactylus limbatus	L	H	L	**L**
Eleutherodactylus locustus	H	H	H	**H**
Eleutherodactylus longipes	H	H	H	**H**
Eleutherodactylus lucioi	H	H	H	**H**
Eleutherodactylus luteolus	H	H	H	**H**
Eleutherodactylus maestrensis	H	L	H	**L**
Eleutherodactylus mariposa	H	H	H	**H**
Eleutherodactylus marnockii	H	H	L	**L**
Eleutherodactylus martinicensis	L	H	H	**L**

Eleutherodactylus maurus	H	L	L	**L**
Eleutherodactylus melacara	L	H	L	**L**
Eleutherodactylus michaelschmidi	L	H	H	**L**
Eleutherodactylus minutus	H	H	H	**H**
Eleutherodactylus modestus	H	H	L	**L**
Eleutherodactylus monensis	H	H	U	**L**
Eleutherodactylus montanus	H	H	H	**H**
Eleutherodactylus nitidus	H	H	L	**L**
Eleutherodactylus nivicolimae	L	H	H	**L**
Eleutherodactylus nortoni	L	H	H	**L**
Eleutherodactylus nubicola	H	H	H	**H**
Eleutherodactylus orcutti	H	H	H	**H**
Eleutherodactylus orientalis	H	H	L	**L**
Eleutherodactylus oxyrhyncus	L	H	H	**L**
Eleutherodactylus pallidus	U	L	H	**L**
Eleutherodactylus pantoni	L	H	H	**L**
Eleutherodactylus parabates	H	H	H	**H**
Eleutherodactylus parapelates	L	H	H	**L**
Eleutherodactylus patriciae	H	H	H	**H**
Eleutherodactylus paulsoni	L	H	H	**L**
Eleutherodactylus pentasyringos	L	H	H	**L**
Eleutherodactylus pezopetrus	L	H	H	**L**
Eleutherodactylus pictissimus	L	H	H	**L**
Eleutherodactylus pinarensis	L	H	L	**L**
Eleutherodactylus pinchoni	L	H	H	**L**
Eleutherodactylus pipilans	L	H	L	**L**
Eleutherodactylus pituinus	H	H	H	**H**
Eleutherodactylus planirostris	L	H	L	**L**
Eleutherodactylus poolei	H	H	H	**H**
Eleutherodactylus portoricensis	H	H	H	**H**
Eleutherodactylus principalis	H	H	H	**H**
Eleutherodactylus probolaeus	H	H	L	**L**
Eleutherodactylus rhodesi	H	H	H	**H**
Eleutherodactylus richmondi	H	H	H	**H**
Eleutherodactylus ricordii	L	H	H	**L**
Eleutherodactylus riparius	H	H	L	**L**
Eleutherodactylus rivularis	L	H	L	**L**
Eleutherodactylus rogersi	H	L	L	**L**
Eleutherodactylus ronaldi	L	H	H	**L**
Eleutherodactylus rubrimaculatus	H	H	H	**H**
Eleutherodactylus rufescens	H	H	H	**H**
Eleutherodactylus rufifemoralis	L	H	H	**L**
Eleutherodactylus ruthae	H	H	H	**H**
Eleutherodactylus saxatilis	L	H	L	**L**
Eleutherodactylus schmidti	H	H	H	**H**

Eleutherodactylus schwartzi	H	H	L	**L**
Eleutherodactylus sciagraphus	H	H	H	**H**
Eleutherodactylus semipalmatus	H	H	H	**H**
Eleutherodactylus simulans	H	H	H	**H**
Eleutherodactylus sisyphodemus	H	H	L	**L**
Eleutherodactylus symingtoni	H	H	L	**L**
Eleutherodactylus syristes	H	H	L	**L**
Eleutherodactylus teretistes	H	L	H	**L**
Eleutherodactylus tetajulia	H	H	L	**L**
Eleutherodactylus thomasi	L	H	L	**L**
Eleutherodactylus thorectes	H	H	H	**H**
Eleutherodactylus toa	H	H	H	**H**
Eleutherodactylus tonyi	H	H	L	**L**
Eleutherodactylus turquinensis	H	H	L	**L**
Eleutherodactylus unicolor	H	H	H	**H**
Eleutherodactylus varians	H	H	L	**L**
Eleutherodactylus varleyi	L	H	L	**L**
Eleutherodactylus ventrilineatus	H	H	H	**H**
Eleutherodactylus verrucipes	L	H	H	**L**
Eleutherodactylus verruculatus	H	H	H	**H**
Eleutherodactylus warreni	H	H	L	**L**
Eleutherodactylus weinlandi	H	H	H	**H**
Eleutherodactylus wetmorei	L	H	H	**L**
Eleutherodactylus wightmanae	H	H	H	**H**
Eleutherodactylus zeus	L	H	L	**L**
Eleutherodactylus zugi	H	H	L	**L**
Engystomops coloradorum	U	H	H	**L**
Engystomops guayaco	H	L	H	**L**
Engystomops montubio	H	L	H	**L**
Engystomops petersi	U	L	U	**L**
Engystomops pustulatus	H	L	H	**L**
Engystomops pustulosus	L	L	L	**L**
Engystomops randi	H	L	H	**L**
Ensatina eschscholtzii	L	H	L	**L**
Epicrionops bicolor	L	H	H	**L**
Epicrionops columbianus	H	L	L	**L**
Epicrionops lativittatus	U	L	U	**L**
Epicrionops marmoratus	H	L	H	**L**
Epicrionops niger	L	H	H	**L**
Epicrionops parkeri	H	L	L	**L**
Epicrionops peruvianus	H	L	H	**L**
Epicrionops petersi	L	H	H	**L**
Epidalea calamita	H	L	L	**L**
Epipedobates anthonyi	H	H	H	**H**
Epipedobates boulengeri	H	H	H	**H**

Epipedobates espinosai	H	L	H	**L**
Epipedobates machalilla	H	H	H	**H**
Epipedobates tricolor	H	H	H	**H**
Ericabatrachus baleensis	H	H	H	**H**
Euparkerella brasiliensis	H	L	H	**L**
Euparkerella cochranae	H	H	H	**H**
Euparkerella robusta	H	H	L	**L**
Euparkerella tridactyla	H	H	L	**L**
Eupemphix nattereri	H	L	L	**L**
Euphlyctis cyanophlyctis	L	L	L	**L**
Euphlyctis ehrenbergii	H	L	L	**L**
Euphlyctis ghoshi	H	L	L	**L**
Euphlyctis hexadactylus	L	L	L	**L**
Euproctus montanus	H	H	L	**L**
Euproctus platycephalus	H	H	H	**H**
Eupsophus calcaratus	L	L	L	**L**
Eupsophus contulmoensis	L	H	L	**L**
Eupsophus emiliopugini	L	L	L	**L**
Eupsophus insularis	L	H	U	**L**
Eupsophus migueli	L	H	L	**L**
Eupsophus nahuelbutensis	L	H	L	**L**
Eupsophus queulensis	L	L	L	**L**
Eupsophus roseus	L	L	L	**L**
Eupsophus septentrionalis	H	L	L	**L**
Eupsophus vertebralis	L	L	L	**L**
Eurycea bislineata	H	H	L	**L**
Eurycea chamberlaini	U	L	U	**L**
Eurycea chisholmensis	H	H	L	**L**
Eurycea cirrigera	H	H	L	**L**
Eurycea guttolineata	H	H	L	**L**
Eurycea junaluska	H	H	L	**L**
Eurycea latitans	H	H	L	**L**
Eurycea longicauda	H	H	L	**L**
Eurycea lucifuga	H	H	L	**L**
Eurycea multiplicata	H	H	L	**L**
Eurycea nana	H	H	L	**L**
Eurycea naufragia	H	H	L	**L**
Eurycea neotenes	H	H	L	**L**
Eurycea pterophila	H	H	L	**L**
Eurycea quadridigitata	H	H	L	**L**
Eurycea rathbuni	H	H	L	**L**
Eurycea robusta	H	H	L	**L**
Eurycea sosorum	H	H	L	**L**
Eurycea spelaea	H	H	L	**L**
Eurycea tonkawae	H	H	L	**L**

Eurycea tridentifera	H	H	L	**L**
Eurycea troglodytes	H	L	L	**L**
Eurycea tynerensis	H	H	L	**L**
Eurycea wallacei	H	H	H	**H**
Eurycea waterlooensis	H	H	L	**L**
Eurycea wilderae	H	H	L	**L**
Excitobates captivus	H	U	H	**L**
Excitobates mysteriosus	H	H	L	**L**
Exerodonta abdivita	H	L	H	**L**
Exerodonta bivocata	U	L	L	**L**
Exerodonta catracha	L	L	H	**L**
Exerodonta chimalapa	L	L	L	**L**
Exerodonta juanitae	H	L	L	**L**
Exerodonta melanomma	H	L	L	**L**
Exerodonta perkinsi	H	L	H	**L**
Exerodonta pinorum	H	L	L	**L**
Exerodonta smaragdina	H	L	H	**L**
Exerodonta sumichrasti	H	L	L	**L**
Exerodonta xera	H	L	L	**L**
Feihyla palpebralis	L	H	L	**L**
Fejervarya altilabris	H	L	U	**L**
Fejervarya andamanensis	H	H	L	**L**
Fejervarya assimilis	H	L	U	**L**
Fejervarya brama	H	U	U	**L**
Fejervarya brevipalmata	U	H	H	**L**
Fejervarya cancrivora	L	H	L	**L**
Fejervarya frithi	H	H	L	**L**
Fejervarya greenii	L	H	H	**L**
Fejervarya iskandari	H	H	H	**H**
Fejervarya keralensis	L	L	H	**L**
Fejervarya kirtisinghei	L	H	H	**L**
Fejervarya limnocharis	L	L	L	**L**
Fejervarya moodiei	H	H	U	**L**
Fejervarya multistriata	H	L	H	**L**
Fejervarya murthii	H	H	H	**H**
Fejervarya mysorensis	U	L	H	**L**
Fejervarya nepalensis	L	L	H	**L**
Fejervarya nicobariensis	H	L	L	**L**
Fejervarya nilagirica	L	L	H	**L**
Fejervarya orissaensis	H	L	L	**L**
Fejervarya parambikulamana	H	H	H	**H**
Fejervarya pierrei	H	L	H	**L**
Fejervarya pulla	H	L	H	**L**
Fejervarya raja	H	L	L	**L**
Fejervarya rufescens	L	L	H	**L**

Fejervarya sahyadris	L	L	H	**L**
Fejervarya sauriceps	H	L	U	**L**
Fejervarya schlueteri	H	U	U	**L**
Fejervarya syhadrensis	H	H	L	**L**
Fejervarya teraiensis	H	L	H	**L**
Fejervarya triora	U	L	L	**L**
Fejervarya verruculosa	H	H	L	**L**
Fejervarya vittigera	H	L	L	**L**
Flectonotus fissilis	L	H	L	**L**
Flectonotus fitzgeraldi	H	H	H	**H**
Flectonotus goeldii	L	H	L	**L**
Flectonotus ohausi	H	H	L	**L**
Flectonotus pygmaeus	L	H	H	**L**
Frostius erythrophthalmus	H	L	H	**L**
Frostius pernambucensis	H	L	H	**L**
Gastrophryne carolinensis	H	L	L	**L**
Gastrophryne elegans	L	L	H	**L**
Gastrophryne olivacea	H	L	L	**L**
Gastrophryne pictiventris	H	L	H	**L**
Gastrophryne usta	L	H	L	**L**
Gastrophrynoides borneensis	H	H	H	**H**
Gastrotheca abdita	H	H	H	**H**
Gastrotheca albolineata	L	H	L	**L**
Gastrotheca andaquiensis	H	H	H	**H**
Gastrotheca angustifrons	H	H	H	**H**
Gastrotheca antomia	H	H	H	**H**
Gastrotheca argenteovirens	L	H	H	**L**
Gastrotheca atympana	H	L	H	**L**
Gastrotheca aureomaculata	H	H	H	**H**
Gastrotheca bufona	H	H	L	**L**
Gastrotheca carinaceps	H	L	H	**L**
Gastrotheca christiani	L	H	H	**L**
Gastrotheca chrysosticta	H	H	H	**H**
Gastrotheca cornuta	H	L	L	**L**
Gastrotheca dendronastes	H	H	H	**H**
Gastrotheca dunni	L	H	H	**L**
Gastrotheca ernestoi	H	L	L	**L**
Gastrotheca espeletia	H	H	H	**H**
Gastrotheca excubitor	L	H	H	**L**
Gastrotheca fissipes	H	H	H	**H**
Gastrotheca fulvorufa	H	H	H	**H**
Gastrotheca galeata	H	H	H	**H**
Gastrotheca gracilis	H	H	H	**H**
Gastrotheca griswoldi	H	H	H	**H**
Gastrotheca guentheri	H	H	H	**H**

Gastrotheca helenae	H	H	H	**H**
Gastrotheca lateonota	H	H	H	**H**
Gastrotheca lauzuricae	H	H	H	**H**
Gastrotheca litonedis	H	H	H	**H**
Gastrotheca longipes	L	H	H	**L**
Gastrotheca marsupiata	L	H	H	**L**
Gastrotheca microdiscus	L	H	L	**L**
Gastrotheca monticola	H	H	H	**H**
Gastrotheca nicefori	L	H	H	**L**
Gastrotheca ochoai	H	H	H	**H**
Gastrotheca orophylax	H	H	H	**H**
Gastrotheca ossilaginis	H	H	H	**H**
Gastrotheca ovifera	H	H	H	**H**
Gastrotheca pacchamama	H	H	L	**L**
Gastrotheca peruana	H	H	H	**H**
Gastrotheca phalarosa	H	H	H	**H**
Gastrotheca piperata	H	H	H	**H**
Gastrotheca plumbea	H	H	H	**H**
Gastrotheca pseustes	H	H	H	**H**
Gastrotheca psychrophila	H	H	H	**H**
Gastrotheca pulchra	H	L	U	**L**
Gastrotheca rebeccae	H	H	H	**H**
Gastrotheca riobambae	H	H	H	**H**
Gastrotheca ruizi	H	H	H	**H**
Gastrotheca splendens	H	H	H	**H**
Gastrotheca stictopleura	H	H	L	**L**
Gastrotheca testudinea	L	H	H	**L**
Gastrotheca trachyceps	H	H	H	**H**
Gastrotheca walkeri	H	H	H	**H**
Gastrotheca weinlandii	H	H	H	**H**
Gastrotheca williamsoni	H	H	H	**H**
Gastrotheca zeugocystis	H	H	H	**H**
Gegeneophis carnosus	H	L	H	**L**
Gegeneophis danieli	H	L	H	**L**
Gegeneophis fulleri	U	L	H	**L**
Gegeneophis goaensis	H	H	H	**H**
Gegeneophis krishni	H	H	H	**H**
Gegeneophis madhavai	H	L	H	**L**
Gegeneophis mhadeiensis	U	H	H	**L**
Gegeneophis nadkarnii	H	H	H	**H**
Gegeneophis ramaswamii	H	H	H	**H**
Gegeneophis seshachari	H	H	U	**L**
Genyophryne thomsoni	H	H	L	**L**
Geobatrachus walkeri	H	H	H	**H**
Geocrinia alba	H	H	H	**H**

Geocrinia laevis	H	H	L	**L**
Geocrinia leai	H	H	H	**H**
Geocrinia lutea	L	H	H	**L**
Geocrinia rosea	H	H	H	**H**
Geocrinia victoriana	H	H	H	**H**
Geocrinia vitellina	H	H	H	**H**
Geotrypetes angeli	U	H	U	**L**
Geotrypetes pseudoangeli	H	H	U	**L**
Geotrypetes seraphini	L	H	L	**L**
Gephyromantis ambohitra	L	H	H	**L**
Gephyromantis asper	L	H	H	**L**
Gephyromantis azzurrae	L	L	L	**L**
Gephyromantis blanci	L	H	L	**L**
Gephyromantis boulengeri	L	H	H	**L**
Gephyromantis cornutus	U	L	L	**L**
Gephyromantis corvus	L	H	L	**L**
Gephyromantis decaryi	H	H	L	**L**
Gephyromantis eiselti	U	L	L	**L**
Gephyromantis enki	U	L	L	**L**
Gephyromantis granulatus	L	H	H	**L**
Gephyromantis horridus	L	H	H	**L**
Gephyromantis klemmeri	H	H	H	**H**
Gephyromantis leucocephalus	L	H	H	**L**
Gephyromantis leucomaculatus	H	H	H	**H**
Gephyromantis luteus	H	H	H	**H**
Gephyromantis malagasius	L	H	H	**L**
Gephyromantis moseri	H	H	H	**H**
Gephyromantis plicifer	H	H	L	**L**
Gephyromantis pseudoasper	L	H	H	**L**
Gephyromantis redimitus	H	H	H	**H**
Gephyromantis rivicola	H	H	H	**H**
Gephyromantis runewsweeki	L	H	L	**L**
Gephyromantis salegy	H	H	H	**H**
Gephyromantis schilfi	H	H	H	**H**
Gephyromantis sculpturatus	L	H	L	**L**
Gephyromantis silvanus	L	H	H	**L**
Gephyromantis spiniferus	H	H	H	**H**
Gephyromantis striatus	L	H	H	**L**
Gephyromantis tandroka	L	H	H	**L**
Gephyromantis thelenae	U	H	H	**L**
Gephyromantis tschenki	U	L	L	**L**
Gephyromantis ventrimaculatus	H	H	L	**L**
Gephyromantis webbi	L	H	H	**L**
Gephyromantis zavona	H	H	H	**H**
Glandirana emeljanovi	L	L	H	**L**

Glandirana minima	H	L	L	**L**
Glandirana rugosa	L	L	L	**L**
Glandirana tientaiensis	L	L	L	**L**
Glyphoglossus molossus	L	H	H	**L**
Gracixalus gracilipes	L	H	H	**L**
Gracixalus supercornutus	U	L	L	**L**
Grandisonia alternans	H	H	L	**L**
Grandisonia brevis	H	H	L	**L**
Grandisonia larvata	H	H	L	**L**
Grandisonia sechellensis	H	H	L	**L**
Guibemantis albolineatus	U	H	H	**L**
Guibemantis bicalcaratus	L	H	H	**L**
Guibemantis depressiceps	L	H	H	**L**
Guibemantis flavobrunneus	H	H	H	**H**
Guibemantis kathrinae	U	L	H	**L**
Guibemantis liber	L	H	H	**L**
Guibemantis pulcher	L	H	H	**L**
Guibemantis punctatus	U	H	H	**L**
Guibemantis timidus	L	H	H	**L**
Guibemantis tornieri	L	H	H	**L**
Gymnopis multiplicata	L	H	H	**L**
Gymnopis syntrema	U	H	H	**L**
Gyrinophilus gulolineatus	H	H	H	**H**
Gyrinophilus palleucus	H	H	L	**L**
Gyrinophilus porphyriticus	H	H	L	**L**
Gyrinophilus subterraneus	H	H	L	**L**
Haddadus binotatus	L	H	L	**L**
Haddadus plicifer	H	L	H	**L**
Hamptophryne boliviana	H	H	H	**H**
Heleioporus albopunctatus	H	L	L	**L**
Heleioporus australiacus	H	L	L	**L**
Heleioporus barycragus	H	H	H	**H**
Heleioporus eyrei	H	H	L	**L**
Heleioporus inornatus	H	H	H	**H**
Heleioporus psammophilus	H	H	H	**H**
Heleophryne hewitti	H	H	H	**H**
Heleophryne natalensis	H	L	L	**L**
Heleophryne orientalis	H	H	L	**L**
Heleophryne purcelli	H	L	H	**L**
Heleophryne regis	H	L	H	**L**
Heleophryne rosei	H	H	H	**H**
Hemidactylium scutatum	H	H	L	**L**
Hemiphractus bubalus	L	H	H	**L**
Hemiphractus fasciatus	L	H	L	**L**
Hemiphractus helioi	L	H	H	**L**

Hemiphractus johnsoni	H	H	L	**L**
Hemiphractus proboscideus	H	H	H	**H**
Hemiphractus scutatus	H	H	L	**L**
Hemisus barotseensis	H	H	L	**L**
Hemisus brachydactylus	H	H	L	**L**
Hemisus guineensis	H	L	L	**L**
Hemisus guttatus	H	L	L	**L**
Hemisus marmoratus	H	L	L	**L**
Hemisus microscaphus	H	L	L	**L**
Hemisus olivaceus	H	L	L	**L**
Hemisus perreti	H	H	L	**L**
Hemisus wittei	H	H	L	**L**
Herpele multiplicata	H	U	H	**L**
Herpele squalostoma	H	L	L	**L**
Heterixalus alboguttatus	H	H	L	**L**
Heterixalus andrakata	H	H	H	**H**
Heterixalus betsileo	H	H	L	**L**
Heterixalus boettgeri	L	H	H	**L**
Heterixalus carbonei	L	H	L	**L**
Heterixalus luteostriatus	L	H	L	**L**
Heterixalus madagascariensis	L	L	H	**L**
Heterixalus punctatus	H	L	H	**L**
Heterixalus rutenbergi	H	L	L	**L**
Heterixalus tricolor	H	L	H	**L**
Heterixalus variabilis	H	H	H	**H**
Hildebrandtia macrotympanum	H	L	L	**L**
Hildebrandtia ornata	H	L	L	**L**
Hildebrandtia ornatissima	U	L	U	**L**
Holoaden bradei	U	H	H	**L**
Holoaden luederwaldti	U	H	L	**L**
Hoplobatrachus crassus	L	L	L	**L**
Hoplobatrachus occipitalis	L	L	L	**L**
Hoplobatrachus rugulosus	H	H	L	**L**
Hoplobatrachus tigerinus	L	L	L	**L**
Hoplophryne rogersi	H	H	L	**L**
Hoplophryne uluguruensis	H	H	H	**H**
Huia cavitympanum	L	L	H	**L**
Huia masonii	L	L	H	**L**
Huia modiglianii	H	L	H	**L**
Huia sumatrana	L	L	L	**L**
Humerana humeralis	L	H	H	**L**
Humerana miopus	H	L	L	**L**
Humerana oatesii	H	L	L	**L**
Hyalinobatrachium aureoguttatum	H	H	H	**H**
Hyalinobatrachium bergeri	H	H	H	**H**

Hyalinobatrachium cardiacalyptum	H	H	H	**H**
Hyalinobatrachium chirripoi	H	H	L	**L**
Hyalinobatrachium colymbiphyllum	H	H	L	**L**
Hyalinobatrachium crurifasciatum	H	H	H	**H**
Hyalinobatrachium crybetes	H	H	H	**H**
Hyalinobatrachium duranti	H	L	H	**L**
Hyalinobatrachium eccentricum	H	L	H	**L**
Hyalinobatrachium esmeralda	L	H	H	**L**
Hyalinobatrachium eurygnathum	H	H	L	**L**
Hyalinobatrachium fleischmanni	H	H	H	**H**
Hyalinobatrachium fragile	H	H	H	**H**
Hyalinobatrachium guairarepanense	H	H	H	**H**
Hyalinobatrachium iaspidiense	H	L	H	**L**
Hyalinobatrachium ibama	L	H	H	**L**
Hyalinobatrachium ignioculus	H	L	H	**L**
Hyalinobatrachium lemur	H	L	H	**L**
Hyalinobatrachium mondolfii	H	H	H	**H**
Hyalinobatrachium munozorum	H	H	H	**H**
Hyalinobatrachium nouraguense	H	H	H	**H**
Hyalinobatrachium orientale	H	H	H	**H**
Hyalinobatrachium pallidum	L	H	H	**L**
Hyalinobatrachium parvulum	H	L	L	**L**
Hyalinobatrachium pellucidum	L	H	H	**L**
Hyalinobatrachium ruedai	H	H	H	**H**
Hyalinobatrachium talamancae	H	H	H	**H**
Hyalinobatrachium tatayoi	H	H	H	**H**
Hyalinobatrachium taylori	H	H	H	**H**
Hyalinobatrachium uranoscopum	H	H	L	**L**
Hyalinobatrachium valerioi	H	H	L	**L**
Hyalinobatrachium vireovittatum	H	L	H	**L**
Hydrolaetare caparu	H	H	L	**L**
Hydrolaetare dantasi	H	L	H	**L**
Hydrolaetare schmidti	H	L	H	**L**
Hydromantes brunus	L	H	H	**L**
Hydromantes platycephalus	H	H	L	**L**
Hydromantes shastae	L	H	H	**L**
Hyla andersonii	H	L	L	**L**
Hyla annectans	H	L	H	**L**
Hyla antoniiochoai	H	L	H	**L**
Hyla arborea	H	L	L	**L**
Hyla arboricola	U	L	H	**L**
Hyla arenicolor	H	L	H	**L**
Hyla auraria	H	U	U	**L**
Hyla avivoca	H	L	L	**L**

Hyla bocourti	H	L	H	**L**
Hyla chinensis	H	H	L	**L**
Hyla chrysoscelis	H	H	L	**L**
Hyla cinerea	H	H	L	**L**
Hyla euphorbiacea	L	L	L	**L**
Hyla eximia	L	L	L	**L**
Hyla femoralis	H	L	L	**L**
Hyla gratiosa	H	H	L	**L**
Hyla hallowellii	H	H	H	**H**
Hyla heinzsteinitzi	H	H	H	**H**
Hyla helenae	H	L	U	**L**
Hyla imitator	H	L	H	**L**
Hyla immaculata	L	L	H	**L**
Hyla inframaculata	H	L	H	**L**
Hyla intermedia	H	L	L	**L**
Hyla japonica	L	L	H	**L**
Hyla javana	H	U	U	**L**
Hyla meridionalis	H	L	L	**L**
Hyla molitor	H	U	U	**L**
Hyla plicata	H	L	L	**L**
Hyla sanchiangensis	H	L	L	**L**
Hyla sarda	H	H	L	**L**
Hyla savignyi	H	L	L	**L**
Hyla simplex	H	L	L	**L**
Hyla squirella	H	L	L	**L**
Hyla surinamensis	H	U	U	**L**
Hyla suweonensis	H	H	H	**H**
Hyla tsinlingensis	H	L	H	**L**
Hyla versicolor	H	L	L	**L**
Hyla walkeri	H	L	H	**L**
Hyla warreni	H	L	H	**L**
Hyla wrightorum	H	L	L	**L**
Hyla zhaopingensis	H	H	L	**L**
Hylambates dorsalis	H	U	U	**L**
Hylarana albolabris	L	L	L	**L**
Hylarana albotuberculata	H	L	L	**L**
Hylarana amnicola	H	L	L	**L**
Hylarana arfaki	L	L	L	**L**
Hylarana asperrima	L	L	L	**L**
Hylarana attigua	L	L	L	**L**
Hylarana aurantiaca	L	L	H	**L**
Hylarana aurata	H	L	H	**L**
Hylarana banjarana	L	L	L	**L**
Hylarana baramica	H	H	L	**L**
Hylarana celebensis	H	H	H	**H**

Hylarana chalconota	H	H	L	**L**
Hylarana chitwanensis	L	H	H	**L**
Hylarana crassiovis	U	L	H	**L**
Hylarana cubitalis	L	L	H	**L**
Hylarana daemeli	L	L	L	**L**
Hylarana darlingi	L	L	L	**L**
Hylarana debussyi	H	L	L	**L**
Hylarana elberti	L	H	L	**L**
Hylarana erythraea	L	L	L	**L**
Hylarana everetti	U	L	L	**L**
Hylarana faber	L	L	H	**L**
Hylarana florensis	H	H	L	**L**
Hylarana fonensis	H	H	L	**L**
Hylarana galamensis	H	L	L	**L**
Hylarana garoensis	L	H	H	**L**
Hylarana garritor	L	L	L	**L**
Hylarana glandulosa	H	L	L	**L**
Hylarana gracilis	H	H	L	**L**
Hylarana grandocula	L	L	L	**L**
Hylarana grisea	H	L	H	**L**
Hylarana guentheri	H	L	H	**L**
Hylarana igorota	L	H	H	**L**
Hylarana jimiensis	H	L	H	**L**
Hylarana kampeni	L	L	L	**L**
Hylarana kreffti	H	H	L	**L**
Hylarana laterimaculata	H	L	L	**L**
Hylarana latouchii	L	L	L	**L**
Hylarana lemairei	H	L	L	**L**
Hylarana leptoglossa	L	L	L	**L**
Hylarana lepus	H	L	L	**L**
Hylarana longipes	H	L	L	**L**
Hylarana luctuosa	L	L	L	**L**
Hylarana luzonensis	L	H	L	**L**
Hylarana macrodactyla	L	L	L	**L**
Hylarana macrops	H	L	H	**L**
Hylarana malabarica	L	H	L	**L**
Hylarana mangyanum	L	H	H	**L**
Hylarana maosonensis	L	L	H	**L**
Hylarana margariana	H	L	L	**L**
Hylarana melanomenta	H	L	H	**L**
Hylarana milleti	L	L	L	**L**
Hylarana milneana	L	L	L	**L**
Hylarana mocquardii	L	L	H	**L**
Hylarana moellendorffi	H	H	L	**L**
Hylarana moluccana	H	H	H	**H**

Hylarana montivaga	L	L	L	**L**
Hylarana mortenseni	L	L	H	**L**
Hylarana nigrovittata	L	L	L	**L**
Hylarana novaeguineae	H	H	L	**L**
Hylarana occidentalis	H	L	L	**L**
Hylarana papua	L	H	L	**L**
Hylarana parkeriana	U	H	L	**L**
Hylarana persimilis	H	H	L	**L**
Hylarana picturata	L	L	L	**L**
Hylarana raniceps	H	L	L	**L**
Hylarana scutigera	H	L	H	**L**
Hylarana siberu	L	L	L	**L**
Hylarana signata	L	L	L	**L**
Hylarana similis	L	L	L	**L**
Hylarana spinulosa	L	H	L	**L**
Hylarana supragrisea	L	L	L	**L**
Hylarana taipehensis	L	L	L	**L**
Hylarana temporalis	L	L	H	**L**
Hylarana tipanan	L	H	L	**L**
Hylarana tytleri	L	L	H	**L**
Hylarana volkerjane	H	L	H	**L**
Hylarana waliesa	H	L	L	**L**
Hylodes amnicola	H	L	L	**L**
Hylodes asper	H	L	L	**L**
Hylodes babax	U	L	H	**L**
Hylodes charadranaetes	H	L	H	**L**
Hylodes dactylocinus	H	L	H	**L**
Hylodes fredi	U	L	L	**L**
Hylodes glaber	U	L	H	**L**
Hylodes heyeri	H	L	L	**L**
Hylodes lateristrigatus	H	L	L	**L**
Hylodes magalhaesi	U	L	L	**L**
Hylodes meridionalis	H	L	H	**L**
Hylodes mertensi	H	L	H	**L**
Hylodes nasus	H	L	L	**L**
Hylodes ornatus	H	L	L	**L**
Hylodes otavioi	U	L	L	**L**
Hylodes perplicatus	H	L	L	**L**
Hylodes phyllodes	H	L	L	**L**
Hylodes pipilans	U	L	L	**L**
Hylodes regius	U	L	L	**L**
Hylodes sazimai	H	L	L	**L**
Hylodes uai	H	L	L	**L**
Hylodes vanzolinii	H	L	H	**L**
Hylomantis aspera	H	H	H	**H**

Hylomantis buckleyi	H	H	H	**H**
Hylomantis danieli	U	L	H	**L**
Hylomantis granulosa	H	L	L	**L**
Hylomantis hulli	H	L	H	**L**
Hylomantis lemur	H	L	L	**L**
Hylomantis medinae	H	L	H	**L**
Hylomantis psilopygion	H	L	H	**L**
Hylophorbus nigrinus	H	H	L	**L**
Hylophorbus picoides	H	L	L	**L**
Hylophorbus rainerguentheri	H	H	H	**H**
Hylophorbus richardsi	H	H	H	**H**
Hylophorbus rufescens	L	H	L	**L**
Hylophorbus sextus	H	H	H	**H**
Hylophorbus tetraphonus	H	H	L	**L**
Hylophorbus wondiwoi	H	H	H	**H**
Hylorina sylvatica	L	H	L	**L**
Hyloscirtus albopunctulatus	H	L	H	**L**
Hyloscirtus alytolylax	H	L	H	**L**
Hyloscirtus armatus	H	L	H	**L**
Hyloscirtus bogotensis	L	L	H	**L**
Hyloscirtus callipeza	L	L	H	**L**
Hyloscirtus caucanus	H	L	H	**L**
Hyloscirtus charazani	H	L	H	**L**
Hyloscirtus chlorosteus	H	L	H	**L**
Hyloscirtus colymba	H	L	L	**L**
Hyloscirtus denticulentus	L	L	H	**L**
Hyloscirtus estevesi	H	L	U	**L**
Hyloscirtus jahni	H	L	H	**L**
Hyloscirtus larinopygion	L	L	H	**L**
Hyloscirtus lascinius	H	L	H	**L**
Hyloscirtus lindae	H	L	H	**L**
Hyloscirtus lynchi	L	L	H	**L**
Hyloscirtus pacha	H	L	H	**L**
Hyloscirtus palmeri	H	L	L	**L**
Hyloscirtus pantostictus	H	L	H	**L**
Hyloscirtus phyllognathus	H	L	H	**L**
Hyloscirtus piceigularis	L	L	L	**L**
Hyloscirtus platydactylus	H	L	H	**L**
Hyloscirtus psarolaimus	H	L	H	**L**
Hyloscirtus ptychodactylus	H	L	H	**L**
Hyloscirtus sarampiona	H	L	H	**L**
Hyloscirtus simmonsi	L	L	H	**L**
Hyloscirtus staufferorum	H	L	L	**L**
Hyloscirtus tapichalaca	H	L	H	**L**
Hyloscirtus torrenticola	H	L	H	**L**

Hyloxalus abditaurantius	L	H	L	**L**
Hyloxalus anthracinus	H	H	H	**H**
Hyloxalus argyrogaster	H	H	H	**H**
Hyloxalus awa	H	H	H	**H**
Hyloxalus azureiventris	H	H	H	**H**
Hyloxalus betancuri	U	L	H	**L**
Hyloxalus bocagei	H	H	L	**L**
Hyloxalus borjai	H	L	L	**L**
Hyloxalus breviquartus	H	L	L	**L**
Hyloxalus cevallosi	H	H	H	**H**
Hyloxalus chlorocraspedus	H	H	H	**H**
Hyloxalus chocoensis	H	L	L	**L**
Hyloxalus delatorreae	H	H	H	**H**
Hyloxalus edwardsi	H	H	H	**H**
Hyloxalus elachyhistus	H	H	H	**H**
Hyloxalus exasperatus	H	L	H	**L**
Hyloxalus excisus	H	L	L	**L**
Hyloxalus faciopunctulatus	H	H	L	**L**
Hyloxalus fallax	U	L	H	**L**
Hyloxalus fascianigrus	L	H	L	**L**
Hyloxalus fuliginosus	H	L	H	**L**
Hyloxalus idiomelus	H	L	H	**L**
Hyloxalus infraguttatus	H	H	H	**H**
Hyloxalus lehmanni	H	H	H	**H**
Hyloxalus littoralis	H	H	H	**H**
Hyloxalus maculosus	H	H	H	**H**
Hyloxalus maquipucuna	H	L	H	**L**
Hyloxalus marmoreoventris	H	L	H	**L**
Hyloxalus mittermeieri	H	L	H	**L**
Hyloxalus mystax	H	L	H	**L**
Hyloxalus nexipus	H	H	H	**H**
Hyloxalus parcus	H	H	L	**L**
Hyloxalus patitae	H	L	H	**L**
Hyloxalus peculiaris	H	L	H	**L**
Hyloxalus peruvianus	H	H	L	**L**
Hyloxalus pinguis	H	L	H	**L**
Hyloxalus pulchellus	H	H	H	**H**
Hyloxalus pumilus	H	L	H	**L**
Hyloxalus ramosi	U	L	H	**L**
Hyloxalus ruizi	H	H	H	**H**
Hyloxalus saltuarius	H	H	L	**L**
Hyloxalus sauli	H	H	H	**H**
Hyloxalus shuar	L	H	L	**L**
Hyloxalus subpunctatus	H	H	H	**H**
Hyloxalus sylvaticus	H	L	H	**L**

Hyloxalus toachi	H	H	H	**H**
Hyloxalus utcubambensis	H	L	H	**L**
Hyloxalus vergeli	H	H	L	**L**
Hyloxalus vertebralis	H	H	H	**H**
Hyloxalus whymperi	H	L	H	**L**
Hymenochirus boettgeri	H	L	L	**L**
Hymenochirus boulengeri	H	L	U	**L**
Hymenochirus curtipes	H	L	L	**L**
Hymenochirus feae	H	L	H	**L**
Hynobius abei	H	H	H	**H**
Hynobius amjiensis	H	H	L	**L**
Hynobius arisanensis	L	H	H	**L**
Hynobius boulengeri	L	H	H	**L**
Hynobius chinensis	L	H	H	**L**
Hynobius dunni	L	H	H	**L**
Hynobius formosanus	L	H	H	**L**
Hynobius guabangshanensis	H	L	H	**L**
Hynobius hidamontanus	L	H	L	**L**
Hynobius katoi	U	L	L	**L**
Hynobius kimurae	L	H	L	**L**
Hynobius leechii	L	H	H	**L**
Hynobius lichenatus	H	H	L	**L**
Hynobius maoershanensis	L	L	L	**L**
Hynobius naevius	U	H	H	**L**
Hynobius nebulosus	L	H	L	**L**
Hynobius nigrescens	H	H	L	**L**
Hynobius okiensis	H	H	H	**H**
Hynobius quelpaertensis	U	L	H	**L**
Hynobius retardatus	H	L	H	**L**
Hynobius sonani	L	H	H	**L**
Hynobius stejnegeri	L	H	H	**L**
Hynobius takedai	H	H	H	**H**
Hynobius tokyoensis	L	H	H	**L**
Hynobius tsuensis	L	H	H	**L**
Hynobius turkestanicus	H	H	H	**H**
Hynobius yiwuensis	L	H	L	**L**
Hyophryne histrio	H	L	H	**L**
Hyperolius acuticephalus	H	L	U	**L**
Hyperolius acuticeps	H	L	L	**L**
Hyperolius acutirostris	H	L	H	**L**
Hyperolius ademetzi	H	L	L	**L**
Hyperolius adspersus	H	L	L	**L**
Hyperolius albofrenatus	H	L	U	**L**
Hyperolius argus	H	H	L	**L**
Hyperolius atrigularis	U	L	U	**L**

Hyperolius balfouri	H	L	L	**L**
Hyperolius baumanni	L	H	L	**L**
Hyperolius benguellensis	H	L	L	**L**
Hyperolius bicolor	H	L	L	**L**
Hyperolius bobirensis	H	H	L	**L**
Hyperolius bolifambae	H	L	L	**L**
Hyperolius bopeleti	H	H	H	**H**
Hyperolius brachiofasciatus	H	L	L	**L**
Hyperolius camerunensis	H	L	L	**L**
Hyperolius castaneus	L	H	L	**L**
Hyperolius chlorosteus	H	L	L	**L**
Hyperolius chrysogaster	H	H	L	**L**
Hyperolius cinereus	H	L	L	**L**
Hyperolius cinnamomeoventris	L	H	L	**L**
Hyperolius concolor	H	H	L	**L**
Hyperolius cystocandicans	H	H	H	**H**
Hyperolius diaphanus	U	H	H	**L**
Hyperolius dintelmanni	H	L	L	**L**
Hyperolius discodactylus	H	H	L	**L**
Hyperolius endjami	L	H	H	**L**
Hyperolius fasciatus	H	L	L	**L**
Hyperolius ferreirai	H	L	L	**L**
Hyperolius ferrugineus	H	L	H	**L**
Hyperolius frontalis	H	H	L	**L**
Hyperolius fuscigula	H	L	H	**L**
Hyperolius fusciventris	H	L	L	**L**
Hyperolius ghesquieri	H	L	U	**L**
Hyperolius glandicolor	H	L	L	**L**
Hyperolius gularis	H	L	L	**L**
Hyperolius guttulatus	H	H	L	**L**
Hyperolius horstockii	H	H	H	**H**
Hyperolius houyi	H	L	U	**L**
Hyperolius hutsebauti	H	H	L	**L**
Hyperolius igbettensis	H	H	L	**L**
Hyperolius inornatus	H	L	L	**L**
Hyperolius kachalolae	H	H	L	**L**
Hyperolius kibarae	U	H	U	**L**
Hyperolius kihangensis	L	H	L	**L**
Hyperolius kivuensis	L	H	L	**L**
Hyperolius kuligae	H	H	L	**L**
Hyperolius lamottei	H	L	L	**L**
Hyperolius langi	H	H	L	**L**
Hyperolius lateralis	L	L	L	**L**
Hyperolius laticeps	H	L	U	**L**
Hyperolius laurenti	H	L	L	**L**

Hyperolius leleupi	H	H	H	**H**
Hyperolius leucotaenius	H	L	L	**L**
Hyperolius lucani	H	L	U	**L**
Hyperolius maestus	H	L	U	**L**
Hyperolius major	L	L	L	**L**
Hyperolius marginatus	H	L	L	**L**
Hyperolius mariae	H	L	L	**L**
Hyperolius marmoratus	H	L	L	**L**
Hyperolius minutissimus	H	L	L	**L**
Hyperolius mitchelli	L	H	L	**L**
Hyperolius molleri	H	H	L	**L**
Hyperolius montanus	H	H	H	**H**
Hyperolius mosaicus	H	H	L	**L**
Hyperolius nasicus	H	L	H	**L**
Hyperolius nasutus	H	L	L	**L**
Hyperolius nienokouensis	H	H	L	**L**
Hyperolius nimbae	H	H	L	**L**
Hyperolius nitidulus	H	L	L	**L**
Hyperolius obscurus	H	L	H	**L**
Hyperolius occidentalis	L	H	H	**L**
Hyperolius ocellatus	H	L	L	**L**
Hyperolius orkarkarri	H	L	L	**L**
Hyperolius parallelus	H	L	L	**L**
Hyperolius pardalis	H	L	L	**L**
Hyperolius parkeri	L	H	L	**L**
Hyperolius phantasticus	H	H	L	**L**
Hyperolius pickersgilli	H	H	L	**L**
Hyperolius picturatus	H	L	L	**L**
Hyperolius pictus	H	H	L	**L**
Hyperolius platyceps	H	L	L	**L**
Hyperolius polli	H	L	U	**L**
Hyperolius polystictus	H	L	L	**L**
Hyperolius poweri	H	H	L	**L**
Hyperolius protchei	H	L	U	**L**
Hyperolius pseudargus	H	H	L	**L**
Hyperolius puncticulatus	H	H	H	**H**
Hyperolius punctulatus	H	H	L	**L**
Hyperolius pusillus	H	H	L	**L**
Hyperolius pustulifer	H	L	H	**L**
Hyperolius pyrrhodictyon	H	L	L	**L**
Hyperolius quadratomaculatus	H	L	H	**L**
Hyperolius quinquevittatus	H	L	L	**L**
Hyperolius raveni	H	U	U	**L**
Hyperolius reesi	L	L	H	**L**
Hyperolius rhizophilus	H	H	L	**L**

Hyperolius rhodesianus	H	L	L	**L**
Hyperolius riggenbachi	H	L	L	**L**
Hyperolius robustus	H	H	L	**L**
Hyperolius rubrovermiculatus	H	H	L	**L**
Hyperolius sankuruensis	H	L	L	**L**
Hyperolius schoutedeni	H	L	L	**L**
Hyperolius seabrai	H	L	L	**L**
Hyperolius semidiscus	H	L	L	**L**
Hyperolius sheldricki	H	L	H	**L**
Hyperolius soror	H	H	U	**L**
Hyperolius spinigularis	L	H	L	**L**
Hyperolius steindachneri	U	L	H	**L**
Hyperolius stenodactylus	H	L	H	**L**
Hyperolius substriatus	L	L	L	**L**
Hyperolius swynnertoni	H	L	L	**L**
Hyperolius sylvaticus	H	H	L	**L**
Hyperolius tannerorum	H	L	H	**L**
Hyperolius thomensis	L	H	H	**L**
Hyperolius thoracotuberculatus	H	U	U	**L**
Hyperolius tornieri	H	L	U	**L**
Hyperolius torrentis	L	L	L	**L**
Hyperolius tuberculatus	H	L	L	**L**
Hyperolius tuberilinguis	H	L	L	**L**
Hyperolius vilhenai	H	L	L	**L**
Hyperolius viridiflavus	L	L	L	**L**
Hyperolius viridigulosus	H	H	L	**L**
Hyperolius viridis	H	L	L	**L**
Hyperolius watsonae	H	H	L	**L**
Hyperolius wermuthi	H	H	L	**L**
Hyperolius xenorhinus	H	L	H	**L**
Hyperolius zonatus	H	H	L	**L**
Hypodactylus adercus	H	H	L	**L**
Hypodactylus araiodactylus	H	H	H	**H**
Hypodactylus babax	L	H	H	**L**
Hypodactylus brunneus	H	H	H	**H**
Hypodactylus dolops	H	H	H	**H**
Hypodactylus elassodiscus	H	H	H	**H**
Hypodactylus fallaciosus	H	L	H	**L**
Hypodactylus latens	L	H	H	**L**
Hypodactylus lucida	H	H	L	**L**
Hypodactylus lundbergi	H	L	L	**L**
Hypodactylus mantipus	L	H	H	**L**
Hypodactylus nigrovittatus	H	H	H	**H**
Hypodactylus peraccai	H	H	H	**H**
Hypogeophis rostratus	H	H	L	**L**

Hypopachus barberi	L	L	H	**L**
Hypopachus variolosus	L	L	L	**L**
Hypsiboas albomarginatus	H	L	L	**L**
Hypsiboas alboniger	H	L	H	**L**
Hypsiboas albopunctatus	H	L	L	**L**
Hypsiboas albovittatus	H	L	U	**L**
Hypsiboas alemani	H	L	H	**L**
Hypsiboas andinus	H	L	H	**L**
Hypsiboas atlanticus	H	H	H	**H**
Hypsiboas balzani	H	L	H	**L**
Hypsiboas beckeri	U	L	L	**L**
Hypsiboas benitezi	H	L	H	**L**
Hypsiboas bischoffi	H	L	L	**L**
Hypsiboas boans	H	L	H	**L**
Hypsiboas buriti	H	L	L	**L**
Hypsiboas caingua	H	L	L	**L**
Hypsiboas calcaratus	H	L	H	**L**
Hypsiboas callipleura	L	L	H	**L**
Hypsiboas cinerascens	H	L	H	**L**
Hypsiboas cipoensis	L	L	L	**L**
Hypsiboas cordobae	H	L	H	**L**
Hypsiboas crepitans	H	H	L	**L**
Hypsiboas curupi	H	H	L	**L**
Hypsiboas cymbalum	H	L	L	**L**
Hypsiboas dentei	H	L	H	**L**
Hypsiboas ericae	H	L	L	**L**
Hypsiboas exastis	H	L	H	**L**
Hypsiboas faber	H	L	L	**L**
Hypsiboas fasciatus	H	H	H	**H**
Hypsiboas freicanecae	H	L	L	**L**
Hypsiboas fuentei	H	L	H	**L**
Hypsiboas geographicus	H	L	L	**L**
Hypsiboas goianus	H	L	L	**L**
Hypsiboas guentheri	H	H	L	**L**
Hypsiboas heilprini	H	L	H	**L**
Hypsiboas hobbsi	H	L	L	**L**
Hypsiboas hutchinsi	H	L	L	**L**
Hypsiboas hypselops	H	L	U	**L**
Hypsiboas jimenezi	H	H	H	**H**
Hypsiboas joaquini	H	L	H	**L**
Hypsiboas lanciformis	H	L	L	**L**
Hypsiboas latistriatus	U	L	L	**L**
Hypsiboas lemai	H	L	H	**L**
Hypsiboas leptolineatus	H	L	L	**L**
Hypsiboas leucocheilus	H	L	H	**L**

Hypsiboas liliae	H	U	H	**L**
Hypsiboas lundii	H	L	L	**L**
Hypsiboas marginatus	H	L	H	**L**
Hypsiboas marianitae	H	L	H	**L**
Hypsiboas melanopleura	H	L	L	**L**
Hypsiboas microderma	H	L	L	**L**
Hypsiboas multifasciatus	H	L	H	**L**
Hypsiboas nympha	H	H	H	**H**
Hypsiboas ornatissimus	H	H	H	**H**
Hypsiboas palaestes	U	L	H	**L**
Hypsiboas palliatus	H	H	U	**L**
Hypsiboas pardalis	H	L	L	**L**
Hypsiboas pellucens	H	H	H	**H**
Hypsiboas phaeopleura	H	L	L	**L**
Hypsiboas picturatus	H	L	H	**L**
Hypsiboas polytaenius	H	L	L	**L**
Hypsiboas pombali	H	L	H	**L**
Hypsiboas prasinus	H	L	L	**L**
Hypsiboas pugnax	H	L	H	**L**
Hypsiboas pulchellus	H	H	L	**L**
Hypsiboas pulidoi	H	L	H	**L**
Hypsiboas punctatus	H	H	L	**L**
Hypsiboas raniceps	H	L	L	**L**
Hypsiboas rhythmicus	U	L	H	**L**
Hypsiboas riojanus	H	L	H	**L**
Hypsiboas roeschmanni	H	L	U	**L**
Hypsiboas roraima	U	L	H	**L**
Hypsiboas rosenbergi	H	L	L	**L**
Hypsiboas rubracylus	H	L	H	**L**
Hypsiboas rufitelus	H	H	H	**H**
Hypsiboas secedens	U	L	U	**L**
Hypsiboas semiguttatus	H	L	L	**L**
Hypsiboas semilineatus	H	L	L	**L**
Hypsiboas sibleszi	H	L	H	**L**
Hypsiboas stenocephalus	H	L	L	**L**
Hypsiboas varelae	H	L	L	**L**
Hypsiboas wavrini	H	L	H	**L**
Ichthyophis acuminatus	U	U	H	**L**
Ichthyophis atricollaris	U	U	U	**L**
Ichthyophis bannanicus	L	H	H	**L**
Ichthyophis beddomei	L	H	H	**L**
Ichthyophis bernisi	U	U	U	**L**
Ichthyophis biangularis	H	U	H	**L**
Ichthyophis billitonensis	H	U	H	**L**
Ichthyophis bombayensis	U	U	H	**L**

Ichthyophis dulitensis	H	U	H	**L**
Ichthyophis elongatus	U	U	L	**L**
Ichthyophis garoensis	U	U	H	**L**
Ichthyophis glandulosus	H	U	H	**L**
Ichthyophis glutinosus	L	H	L	**L**
Ichthyophis humphreyi	H	U	U	**L**
Ichthyophis husaini	U	U	H	**L**
Ichthyophis hypocyaneus	H	U	H	**L**
Ichthyophis javanicus	U	U	U	**L**
Ichthyophis kodaguensis	H	U	H	**L**
Ichthyophis kohtaoensis	L	H	L	**L**
Ichthyophis laosensis	U	U	U	**L**
Ichthyophis longicephalus	H	U	H	**L**
Ichthyophis malabarensis	U	U	U	**L**
Ichthyophis mindanaoensis	U	U	L	**L**
Ichthyophis monochrous	H	U	U	**L**
Ichthyophis orthoplicatus	L	H	H	**L**
Ichthyophis paucisulcus	U	U	L	**L**
Ichthyophis peninsularis	U	U	U	**L**
Ichthyophis pseudangularis	L	H	H	**L**
Ichthyophis sikkimensis	U	U	H	**L**
Ichthyophis singaporensis	H	U	L	**L**
Ichthyophis subterrestris	U	U	U	**L**
Ichthyophis sumatranus	H	U	H	**L**
Ichthyophis supachaii	H	U	L	**L**
Ichthyophis tricolor	L	H	H	**L**
Ichthyophis youngorum	H	U	H	**L**
Idiocranium russeli	U	U	U	**L**
Incilius alvarius	H	L	H	**L**
Incilius aucoinae	H	L	H	**L**
Incilius bocourti	L	L	H	**L**
Incilius campbelli	H	L	H	**L**
Incilius canaliferus	H	L	H	**L**
Incilius cavifrons	H	L	H	**L**
Incilius coccifer	H	L	H	**L**
Incilius coniferus	H	L	H	**L**
Incilius cristatus	L	L	H	**L**
Incilius cycladen	H	H	U	**L**
Incilius fastidiosus	H	H	H	**H**
Incilius gemmifer	H	L	H	**L**
Incilius holdridgei	H	L	U	**L**
Incilius ibarrai	L	L	H	**L**
Incilius intermedius	H	U	U	**L**
Incilius leucomyos	H	L	H	**L**
Incilius luetkenii	H	H	H	**H**

Incilius macrocristatus	L	L	L	**L**
Incilius marmoreus	H	L	H	**L**
Incilius mazatlanensis	H	L	H	**L**
Incilius melanochlorus	H	L	H	**L**
Incilius nebulifer	H	L	H	**L**
Incilius occidentalis	L	L	L	**L**
Incilius periglenes	H	L	U	**L**
Incilius peripatetes	H	L	H	**L**
Incilius perplexus	H	L	H	**L**
Incilius pisinnus	H	L	H	**L**
Incilius porteri	H	L	H	**L**
Incilius signifer	H	L	L	**L**
Incilius spiculatus	H	L	H	**L**
Incilius tacanensis	H	L	H	**L**
Incilius tutelarius	H	L	L	**L**
Incilius valliceps	H	H	H	**H**
Indirana beddomii	L	L	H	**L**
Indirana brachytarsus	L	L	L	**L**
Indirana diplosticta	L	L	H	**L**
Indirana gundia	H	L	H	**L**
Indirana leithii	L	L	H	**L**
Indirana leptodactyla	L	L	L	**L**
Indirana longicrus	U	L	H	**L**
Indirana phrynoderma	H	L	H	**L**
Indirana semipalmata	L	L	H	**L**
Indirana tenuilingua	U	L	H	**L**
Indotyphlus battersbyi	U	U	H	**L**
Indotyphlus maharashtraensis	H	U	H	**L**
Ingerana alpina	H	L	H	**L**
Ingerana baluensis	H	H	H	**H**
Ingerana charlesdarwini	H	H	L	**L**
Ingerana liui	L	H	H	**L**
Ingerana mariae	H	L	L	**L**
Ingerana medogensis	H	L	H	**L**
Ingerana reticulata	H	L	H	**L**
Ingerana tasanae	L	H	L	**L**
Ingerana tenasserimensis	H	H	H	**H**
Ingerana xizangensis	H	L	H	**L**
Ingerophrynus biporcatus	L	L	L	**L**
Ingerophrynus celebensis	L	L	H	**L**
Ingerophrynus claviger	H	L	H	**L**
Ingerophrynus divergens	L	L	H	**L**
Ingerophrynus galeatus	L	L	L	**L**
Ingerophrynus gollum	H	L	L	**L**
Ingerophrynus kumquat	H	L	L	**L**

Ingerophrynus macrotis	L	L	H	**L**
Ingerophrynus parvus	L	L	L	**L**
Ingerophrynus philippinicus	H	L	H	**L**
Ingerophrynus quadriporcatus	H	H	L	**L**
Insuetophrynus acarpicus	L	L	L	**L**
Ischnocnema bilineata	H	H	H	**H**
Ischnocnema bolbodactyla	H	H	H	**H**
Ischnocnema epipeda	H	H	L	**L**
Ischnocnema erythromera	U	H	H	**L**
Ischnocnema gehrti	H	H	H	**H**
Ischnocnema gualteri	L	H	H	**L**
Ischnocnema guentheri	L	H	L	**L**
Ischnocnema henselii	H	H	L	**L**
Ischnocnema hoehnei	L	H	H	**L**
Ischnocnema holti	H	L	H	**L**
Ischnocnema izecksohni	U	L	H	**L**
Ischnocnema juipoca	L	H	L	**L**
Ischnocnema lactea	L	H	L	**L**
Ischnocnema manezinho	H	H	L	**L**
Ischnocnema nasuta	L	H	L	**L**
Ischnocnema nigriventris	H	H	H	**H**
Ischnocnema octavioi	L	H	L	**L**
Ischnocnema oea	H	H	L	**L**
Ischnocnema paranaensis	H	L	L	**L**
Ischnocnema parva	H	H	L	**L**
Ischnocnema paulodutrai	H	H	H	**H**
Ischnocnema penaxavantinho	U	H	L	**L**
Ischnocnema pusilla	U	H	L	**L**
Ischnocnema ramagii	L	H	H	**L**
Ischnocnema randorum	H	H	H	**H**
Ischnocnema sambaqui	H	L	H	**L**
Ischnocnema spanios	H	L	H	**L**
Ischnocnema venancioi	L	H	H	**L**
Ischnocnema verrucosa	H	L	L	**L**
Ischnocnema vinhai	H	H	H	**H**
Isthmohyla angustilineata	H	L	H	**L**
Isthmohyla calypsa	H	H	H	**H**
Isthmohyla debilis	H	L	H	**L**
Isthmohyla graceae	H	L	H	**L**
Isthmohyla infucata	H	H	H	**H**
Isthmohyla insolita	L	L	H	**L**
Isthmohyla lancasteri	H	L	H	**L**
Isthmohyla melacaena	H	H	H	**H**
Isthmohyla picadoi	H	L	H	**L**
Isthmohyla pictipes	H	L	H	**L**

Isthmohyla pseudopuma	H	L	H	**L**
Isthmohyla rivularis	H	L	H	**L**
Isthmohyla tica	H	L	H	**L**
Isthmohyla xanthosticta	H	L	H	**L**
Isthmohyla zeteki	H	H	H	**H**
Itapotihyla langsdorffii	L	H	L	**L**
Kalophrynus baluensis	H	H	H	**H**
Kalophrynus bunguranus	H	H	L	**L**
Kalophrynus eok	H	H	H	**H**
Kalophrynus heterochirus	L	H	H	**L**
Kalophrynus interlineatus	L	H	L	**L**
Kalophrynus intermedius	H	H	H	**H**
Kalophrynus menglienicus	H	H	H	**H**
Kalophrynus minusculus	H	H	H	**H**
Kalophrynus nubicola	H	H	H	**H**
Kalophrynus orangensis	H	H	H	**H**
Kalophrynus palmatissimus	H	H	L	**L**
Kalophrynus pleurostigma	L	H	L	**L**
Kalophrynus punctatus	H	H	L	**L**
Kalophrynus robinsoni	H	H	U	**L**
Kalophrynus subterrestris	H	H	H	**H**
Kaloula assamensis	U	H	H	**L**
Kaloula aureata	H	H	U	**L**
Kaloula baleata	L	L	L	**L**
Kaloula borealis	L	H	H	**L**
Kaloula conjuncta	H	L	L	**L**
Kaloula kalingensis	L	H	L	**L**
Kaloula kokacii	H	H	L	**L**
Kaloula macrocephala	U	H	U	**L**
Kaloula mediolineata	L	H	L	**L**
Kaloula picta	H	L	L	**L**
Kaloula pulchra	L	L	L	**L**
Kaloula rigida	L	L	L	**L**
Kaloula rugifera	H	H	H	**H**
Kaloula taprobanica	L	L	L	**L**
Kaloula verrucosa	H	H	L	**L**
Kaloula walteri	H	L	L	**L**
Karsenia koreana	H	L	H	**L**
Kassina arboricola	H	H	L	**L**
Kassina cassinoides	H	L	H	**L**
Kassina cochranae	H	L	L	**L**
Kassina decorata	H	H	L	**L**
Kassina fusca	H	H	H	**H**
Kassina jozani	H	H	L	**L**
Kassina kuvangensis	H	L	L	**L**

Kassina lamottei	H	L	L	**L**
Kassina maculata	H	H	L	**L**
Kassina maculifer	H	L	L	**L**
Kassina maculosa	H	L	L	**L**
Kassina mertensi	H	L	L	**L**
Kassina schioetzi	H	H	L	**L**
Kassina senegalensis	H	L	L	**L**
Kassina somalica	H	L	L	**L**
Kassina wazae	H	H	L	**L**
Kassinula wittei	H	L	L	**L**
Kurixalus ananjevae	H	L	H	**L**
Kurixalus baliogaster	L	L	L	**L**
Kurixalus bisacculus	L	L	L	**L**
Kurixalus carinensis	H	L	L	**L**
Kurixalus eiffingeri	L	H	H	**L**
Kurixalus hainanus	H	H	L	**L**
Kurixalus idiootocus	H	H	H	**H**
Kurixalus naso	U	H	H	**L**
Kurixalus verrucosus	L	H	L	**L**
Laliostoma labrosum	H	H	L	**L**
Lankanectes corrugatus	L	H	L	**L**
Lanzarana largeni	H	L	L	**L**
Laurentophryne parkeri	H	L	H	**L**
Lechriodus aganoposis	L	H	L	**L**
Lechriodus fletcheri	H	H	L	**L**
Lechriodus melanopyga	L	L	L	**L**
Lechriodus platyceps	H	H	H	**H**
Leiopelma archeyi	H	H	H	**H**
Leiopelma hamiltoni	H	H	L	**L**
Leiopelma hochstetteri	H	H	H	**H**
Leiopelma pakeka	H	H	H	**H**
Lepidobatrachus asper	H	L	L	**L**
Lepidobatrachus laevis	H	H	L	**L**
Lepidobatrachus llanensis	H	L	L	**L**
Leptobrachella baluensis	L	L	H	**L**
Leptobrachella brevicrus	H	H	H	**H**
Leptobrachella mjobergi	H	H	L	**L**
Leptobrachella natunae	H	L	L	**L**
Leptobrachella palmata	H	H	H	**H**
Leptobrachella parva	H	H	H	**H**
Leptobrachella serasanae	H	H	H	**H**
Leptobrachium abbotti	L	L	L	**L**
Leptobrachium ailaonicum	L	L	H	**L**
Leptobrachium banae	L	L	L	**L**
Leptobrachium boringii	L	L	H	**L**

Leptobrachium buchardi	H	H	L	**L**
Leptobrachium chapaense	L	L	L	**L**
Leptobrachium echinatum	L	L	H	**L**
Leptobrachium gunungense	H	H	H	**H**
Leptobrachium hainanense	L	H	L	**L**
Leptobrachium hasseltii	L	L	L	**L**
Leptobrachium hendricksoni	L	L	L	**L**
Leptobrachium huashen	L	L	L	**L**
Leptobrachium leishanense	L	L	L	**L**
Leptobrachium liui	L	L	L	**L**
Leptobrachium montanum	L	L	H	**L**
Leptobrachium mouhoti	U	L	L	**L**
Leptobrachium ngoclinhense	U	L	H	**L**
Leptobrachium nigrops	H	L	L	**L**
Leptobrachium promustache	U	L	H	**L**
Leptobrachium pullum	H	L	L	**L**
Leptobrachium smithi	L	L	L	**L**
Leptobrachium xanthospilum	U	L	L	**L**
Leptodactylodon albiventris	H	L	H	**L**
Leptodactylodon axillaris	H	H	H	**H**
Leptodactylodon bicolor	L	L	L	**L**
Leptodactylodon blanci	H	L	H	**L**
Leptodactylodon boulengeri	L	L	L	**L**
Leptodactylodon bueanus	L	H	H	**L**
Leptodactylodon erythrogaster	H	H	L	**L**
Leptodactylodon mertensi	L	L	L	**L**
Leptodactylodon ornatus	L	L	L	**L**
Leptodactylodon ovatus	L	L	H	**L**
Leptodactylodon perreti	L	L	L	**L**
Leptodactylodon polyacanthus	L	L	L	**L**
Leptodactylodon stevarti	H	H	L	**L**
Leptodactylodon ventrimarmoratus	H	L	H	**L**
Leptodactylodon wildi	H	H	L	**L**
Leptodactylus albilabris	H	H	L	**L**
Leptodactylus andreae	H	H	H	**H**
Leptodactylus araucaria	H	H	H	**H**
Leptodactylus bokermanni	H	H	L	**L**
Leptodactylus bolivianus	H	L	H	**L**
Leptodactylus bufonius	H	L	L	**L**
Leptodactylus caatingae	H	H	L	**L**
Leptodactylus camaquara	H	L	L	**L**
Leptodactylus chaquensis	H	H	L	**L**
Leptodactylus colombiensis	H	H	H	**H**
Leptodactylus cunicularius	H	L	L	**L**
Leptodactylus didymus	H	H	H	**H**

Leptodactylus diedrus	H	L	L	**L**
Leptodactylus diptyx	L	H	L	**L**
Leptodactylus discodactylus	H	L	L	**L**
Leptodactylus elenae	H	H	L	**L**
Leptodactylus fallax	H	H	H	**H**
Leptodactylus flavopictus	H	L	L	**L**
Leptodactylus fragilis	H	H	L	**L**
Leptodactylus furnarius	H	L	L	**L**
Leptodactylus fuscus	H	L	L	**L**
Leptodactylus gracilis	H	L	L	**L**
Leptodactylus griseigularis	H	H	H	**H**
Leptodactylus hallowelli	H	H	U	**L**
Leptodactylus heyeri	H	H	H	**H**
Leptodactylus hylaedactylus	H	H	L	**L**
Leptodactylus hylodes	H	L	H	**L**
Leptodactylus jolyi	H	H	H	**H**
Leptodactylus labrosus	H	L	H	**L**
Leptodactylus laticeps	H	L	L	**L**
Leptodactylus latinasus	H	L	L	**L**
Leptodactylus lauramiriamae	H	L	L	**L**
Leptodactylus leptodactyloides	H	L	H	**L**
Leptodactylus lineatus	H	H	H	**H**
Leptodactylus lithonaetes	H	L	H	**L**
Leptodactylus longirostris	H	L	H	**L**
Leptodactylus lutzi	U	L	H	**L**
Leptodactylus magistris	U	H	H	**L**
Leptodactylus marambaiae	H	H	L	**L**
Leptodactylus marmoratus	H	H	L	**L**
Leptodactylus martinezi	U	H	L	**L**
Leptodactylus melanonotus	H	L	H	**L**
Leptodactylus mystaceus	H	H	H	**H**
Leptodactylus mystacinus	H	H	L	**L**
Leptodactylus nanus	H	H	L	**L**
Leptodactylus natalensis	H	H	L	**L**
Leptodactylus nesiotus	H	H	U	**L**
Leptodactylus notoaktites	H	L	L	**L**
Leptodactylus ocellatus	H	L	L	**L**
Leptodactylus pallidirostris	H	L	H	**L**
Leptodactylus pascoensis	H	L	H	**L**
Leptodactylus petersii	H	H	H	**H**
Leptodactylus plaumanni	H	L	L	**L**
Leptodactylus podicipinus	H	L	L	**L**
Leptodactylus poecilochilus	H	L	H	**L**
Leptodactylus pustulatus	H	L	L	**L**
Leptodactylus rhodomystax	H	H	H	**H**

Leptodactylus rhodonotus	H	L	H	**L**
Leptodactylus rhodostima	H	H	H	**H**
Leptodactylus riveroi	H	L	H	**L**
Leptodactylus rugosus	H	L	H	**L**
Leptodactylus sabanensis	H	L	H	**L**
Leptodactylus savagei	L	L	H	**L**
Leptodactylus sertanejo	H	L	L	**L**
Leptodactylus silvanimbus	U	L	H	**L**
Leptodactylus spixi	H	L	L	**L**
Leptodactylus stenodema	H	L	H	**L**
Leptodactylus syphax	H	L	L	**L**
Leptodactylus tapiti	H	L	L	**L**
Leptodactylus thomei	H	H	L	**L**
Leptodactylus troglodytes	H	L	L	**L**
Leptodactylus validus	H	H	H	**H**
Leptodactylus ventrimaculatus	H	L	H	**L**
Leptodactylus viridis	H	L	H	**L**
Leptodactylus wagneri	H	L	H	**L**
Leptolalax alpinus	L	H	L	**L**
Leptolalax arayai	H	H	H	**H**
Leptolalax bourreti	U	L	H	**L**
Leptolalax dringi	H	H	H	**H**
Leptolalax fuliginosus	H	L	L	**L**
Leptolalax gracilis	H	L	H	**L**
Leptolalax hamidi	H	H	L	**L**
Leptolalax heteropus	L	L	L	**L**
Leptolalax kajangensis	H	H	U	**L**
Leptolalax liui	L	H	L	**L**
Leptolalax maurus	H	H	H	**H**
Leptolalax melanolecus	H	L	H	**L**
Leptolalax nahangensis	U	L	U	**L**
Leptolalax oshanensis	L	L	H	**L**
Leptolalax pelodytoides	L	H	L	**L**
Leptolalax pictus	H	L	H	**L**
Leptolalax pluvialis	U	L	H	**L**
Leptolalax solus	H	L	H	**L**
Leptolalax sungi	U	L	H	**L**
Leptolalax tuberosus	L	L	L	**L**
Leptolalax ventripunctatus	U	L	H	**L**
Leptopelis anchietae	H	L	U	**L**
Leptopelis argenteus	H	L	L	**L**
Leptopelis aubryi	H	H	L	**L**
Leptopelis barbouri	L	H	L	**L**
Leptopelis bequaerti	H	L	L	**L**
Leptopelis bocagii	H	L	L	**L**

Leptopelis boulengeri	L	L	L	**L**
Leptopelis brevipes	H	H	H	**H**
Leptopelis brevirostris	H	L	L	**L**
Leptopelis broadleyi	H	L	L	**L**
Leptopelis bufonides	H	L	H	**L**
Leptopelis calcaratus	H	H	L	**L**
Leptopelis christyi	L	L	L	**L**
Leptopelis concolor	H	H	L	**L**
Leptopelis crystallinoron	H	H	H	**H**
Leptopelis cynnamomeus	L	L	L	**L**
Leptopelis fenestratus	H	L	H	**L**
Leptopelis fiziensis	U	L	L	**L**
Leptopelis flavomaculatus	L	L	L	**L**
Leptopelis gramineus	H	L	L	**L**
Leptopelis jordani	H	L	L	**L**
Leptopelis karissimbensis	H	H	H	**H**
Leptopelis kivuensis	L	L	L	**L**
Leptopelis lebeaui	H	L	H	**L**
Leptopelis mackayi	H	L	H	**L**
Leptopelis macrotis	H	L	L	**L**
Leptopelis marginatus	H	L	L	**L**
Leptopelis millsoni	H	L	L	**L**
Leptopelis modestus	L	L	L	**L**
Leptopelis mossambicus	H	L	L	**L**
Leptopelis natalensis	H	H	L	**L**
Leptopelis nordequatorialis	H	H	L	**L**
Leptopelis notatus	H	L	L	**L**
Leptopelis occidentalis	H	L	L	**L**
Leptopelis ocellatus	H	H	L	**L**
Leptopelis omissus	H	L	L	**L**
Leptopelis oryi	H	L	L	**L**
Leptopelis palmatus	H	H	L	**L**
Leptopelis parbocagii	H	H	L	**L**
Leptopelis parkeri	L	L	L	**L**
Leptopelis parvus	H	L	H	**L**
Leptopelis ragazzii	L	L	L	**L**
Leptopelis rufus	L	L	L	**L**
Leptopelis spiritusnoctis	H	H	L	**L**
Leptopelis susanae	H	H	H	**H**
Leptopelis uluguruensis	L	H	H	**L**
Leptopelis vannutellii	L	L	L	**L**
Leptopelis vermiculatus	L	L	L	**L**
Leptopelis viridis	H	L	L	**L**
Leptopelis xenodactylus	H	H	L	**L**
Leptopelis yaldeni	H	L	L	**L**

Leptopelis zebra	H	H	L	**L**
Leptophryne borbonica	H	L	L	**L**
Leptophryne cruentata	H	H	H	**H**
Limnodynastes convexiusculus	H	H	L	**L**
Limnodynastes depressus	H	L	L	**L**
Limnodynastes dorsalis	H	H	L	**L**
Limnodynastes dumerilii	H	L	L	**L**
Limnodynastes fletcheri	H	L	L	**L**
Limnodynastes interioris	H	L	L	**L**
Limnodynastes lignarius	H	L	L	**L**
Limnodynastes peronii	H	L	L	**L**
Limnodynastes salmini	H	H	L	**L**
Limnodynastes tasmaniensis	H	L	L	**L**
Limnodynastes terraereginae	H	L	L	**L**
Limnomedusa macroglossa	H	L	L	**L**
Limnonectes acanthi	H	L	L	**L**
Limnonectes arathooni	L	H	H	**L**
Limnonectes asperatus	H	L	H	**L**
Limnonectes blythii	L	L	L	**L**
Limnonectes dabanus	U	L	L	**L**
Limnonectes dammermani	L	H	L	**L**
Limnonectes diuatus	L	H	H	**L**
Limnonectes doriae	U	L	L	**L**
Limnonectes finchi	L	H	H	**L**
Limnonectes fragilis	L	H	L	**L**
Limnonectes fujianensis	L	L	L	**L**
Limnonectes grunniens	L	H	L	**L**
Limnonectes gyldenstolpei	L	L	L	**L**
Limnonectes hascheanus	H	H	L	**L**
Limnonectes heinrichi	L	H	L	**L**
Limnonectes ibanorum	L	H	H	**L**
Limnonectes ingeri	H	L	H	**L**
Limnonectes kadarsani	L	H	L	**L**
Limnonectes kenepaiensis	H	L	L	**L**
Limnonectes khammonensis	H	H	H	**H**
Limnonectes khasianus	H	L	H	**L**
Limnonectes kohchangae	H	L	H	**L**
Limnonectes kuhlii	L	H	L	**L**
Limnonectes laticeps	L	L	L	**L**
Limnonectes leporinus	L	L	H	**L**
Limnonectes leytensis	L	L	L	**L**
Limnonectes limborgi	H	L	U	**L**
Limnonectes macrocephalus	L	L	L	**L**
Limnonectes macrodon	H	H	H	**H**
Limnonectes macrognathus	U	L	L	**L**

Limnonectes magnus	L	L	H	**L**
Limnonectes malesianus	H	L	L	**L**
Limnonectes mawlyndipi	U	L	H	**L**
Limnonectes mawphlangensis	U	L	H	**L**
Limnonectes micrixalus	H	L	H	**L**
Limnonectes microdiscus	L	L	H	**L**
Limnonectes microtympanum	L	L	H	**L**
Limnonectes modestus	L	L	H	**L**
Limnonectes namiyei	L	H	H	**L**
Limnonectes nitidus	H	L	L	**L**
Limnonectes palavanensis	L	H	L	**L**
Limnonectes paramacrodon	H	L	L	**L**
Limnonectes parvus	L	L	L	**L**
Limnonectes plicatellus	L	H	L	**L**
Limnonectes poilani	U	L	L	**L**
Limnonectes rhacoda	H	H	H	**H**
Limnonectes shompenorum	L	L	L	**L**
Limnonectes toumanoffi	L	L	L	**L**
Limnonectes tweediei	L	L	L	**L**
Limnonectes visayanus	H	L	L	**L**
Limnonectes woodworthi	L	L	L	**L**
Liophryne allisoni	H	H	L	**L**
Liophryne dentata	L	H	L	**L**
Liophryne rhododactyla	H	H	H	**H**
Liophryne rubra	H	H	H	**H**
Liophryne schlaginhaufeni	L	H	L	**L**
Liophryne similis	H	H	H	**H**
Lissotriton boscai	H	L	L	**L**
Lissotriton helveticus	H	L	L	**L**
Lissotriton italicus	H	H	L	**L**
Lissotriton montandoni	H	L	L	**L**
Lissotriton vulgaris	H	L	H	**L**
Lithobates areolatus	H	L	L	**L**
Lithobates berlandieri	H	H	H	**H**
Lithobates blairi	H	L	L	**L**
Lithobates bwana	H	L	H	**L**
Lithobates capito	H	H	L	**L**
Lithobates catesbeianus	H	L	L	**L**
Lithobates chichicuahutla	H	L	L	**L**
Lithobates chiricahuensis	H	L	L	**L**
Lithobates clamitans	H	L	L	**L**
Lithobates dunni	H	L	L	**L**
Lithobates fisheri	U	L	U	**L**
Lithobates forreri	H	L	L	**L**
Lithobates grylio	H	L	L	**L**

Lithobates heckscheri	H	L	L	**L**
Lithobates johni	H	L	H	**L**
Lithobates juliani	H	L	H	**L**
Lithobates lemosespinali	H	H	L	**L**
Lithobates macroglossa	L	L	H	**L**
Lithobates maculatus	H	L	H	**L**
Lithobates magnaocularis	H	L	H	**L**
Lithobates megapoda	H	L	L	**L**
Lithobates miadis	H	H	U	**L**
Lithobates montezumae	L	H	L	**L**
Lithobates neovolcanicus	H	L	L	**L**
Lithobates okaloosae	H	H	H	**H**
Lithobates omiltemanus	H	L	L	**L**
Lithobates onca	H	L	H	**L**
Lithobates palmipes	H	H	H	**H**
Lithobates palustris	H	L	L	**L**
Lithobates pipiens	H	L	L	**L**
Lithobates psilonota	U	L	L	**L**
Lithobates pueblae	L	L	H	**L**
Lithobates pustulosus	H	L	L	**L**
Lithobates septentrionalis	H	H	L	**L**
Lithobates sevosus	H	H	H	**H**
Lithobates sierramadrensis	H	L	L	**L**
Lithobates spectabilis	L	L	L	**L**
Lithobates sphenocephalus	H	L	L	**L**
Lithobates subaquavocalis	H	L	L	**L**
Lithobates sylvaticus	H	L	L	**L**
Lithobates tarahumarae	H	L	H	**L**
Lithobates taylori	H	L	H	**L**
Lithobates tlaloci	H	L	L	**L**
Lithobates vaillanti	H	L	H	**L**
Lithobates vibicarius	H	L	H	**L**
Lithobates virgatipes	H	L	L	**L**
Lithobates warszewitschii	H	L	H	**L**
Lithobates yavapaiensis	H	L	L	**L**
Lithobates zweifeli	H	L	H	**L**
Litoria adelaidensis	H	H	L	**L**
Litoria alboguttata	H	L	L	**L**
Litoria albolabris	H	H	H	**H**
Litoria amboinensis	H	L	L	**L**
Litoria andiirrmalin	H	L	L	**L**
Litoria angiana	L	H	H	**L**
Litoria arfakiana	H	H	L	**L**
Litoria aruensis	H	L	L	**L**
Litoria auae	H	L	L	**L**

Litoria aurea	H	H	L	**L**
Litoria australis	H	H	L	**L**
Litoria avocalis	H	H	L	**L**
Litoria becki	H	H	H	**H**
Litoria biakensis	H	L	L	**L**
Litoria bicolor	H	L	L	**L**
Litoria booroolongensis	H	L	L	**L**
Litoria brevipalmata	H	H	L	**L**
Litoria brevipes	H	H	L	**L**
Litoria brongersmai	H	L	H	**L**
Litoria bulmeri	U	L	H	**L**
Litoria burrowsi	H	H	H	**H**
Litoria caerulea	H	L	L	**L**
Litoria capitula	H	H	L	**L**
Litoria castanea	H	L	L	**L**
Litoria cavernicola	H	L	L	**L**
Litoria cheesmani	H	L	H	**L**
Litoria chloris	H	L	L	**L**
Litoria chloronota	H	L	H	**L**
Litoria chrisdahli	H	H	L	**L**
Litoria citropa	H	L	L	**L**
Litoria congenita	H	H	L	**L**
Litoria contrastens	H	H	L	**L**
Litoria cooloolensis	H	H	L	**L**
Litoria coplandi	H	L	L	**L**
Litoria cryptotis	H	L	L	**L**
Litoria cultripes	H	L	L	**L**
Litoria cyclorhyncha	H	H	L	**L**
Litoria dahlii	H	H	L	**L**
Litoria darlingtoni	H	H	L	**L**
Litoria daviesae	H	L	L	**L**
Litoria dayi	H	L	H	**L**
Litoria daymani	H	L	H	**L**
Litoria dentata	H	L	L	**L**
Litoria disrupta	L	L	H	**L**
Litoria dorsalis	H	H	L	**L**
Litoria dorsivena	H	L	H	**L**
Litoria dux	H	H	H	**H**
Litoria electrica	H	H	L	**L**
Litoria elkeae	H	L	H	**L**
Litoria eucnemis	H	L	L	**L**
Litoria everetti	H	L	L	**L**
Litoria ewingii	H	H	L	**L**
Litoria exophthalmia	H	H	L	**L**
Litoria fallax	H	L	L	**L**

Litoria fluviatilis	H	L	H	**L**
Litoria foricula	L	L	L	**L**
Litoria freycineti	H	H	L	**L**
Litoria fuscula	H	L	H	**L**
Litoria genimaculata	H	L	L	**L**
Litoria gilleni	H	H	L	**L**
Litoria gracilenta	H	H	L	**L**
Litoria graminea	H	L	L	**L**
Litoria granti	H	L	H	**L**
Litoria gularis	U	L	H	**L**
Litoria havina	H	H	H	**H**
Litoria hilli	H	L	L	**L**
Litoria humboldtorum	H	L	H	**L**
Litoria humeralis	H	L	L	**L**
Litoria hunti	H	H	H	**H**
Litoria impura	H	H	L	**L**
Litoria inermis	H	H	L	**L**
Litoria infrafrenata	H	L	L	**L**
Litoria iris	H	H	H	**H**
Litoria jervisiensis	H	H	L	**L**
Litoria jeudii	H	L	U	**L**
Litoria jungguy	H	L	H	**L**
Litoria kubori	L	L	L	**L**
Litoria kuduki	H	L	L	**L**
Litoria kumae	H	L	L	**L**
Litoria latopalmata	H	H	L	**L**
Litoria lesueurii	H	L	H	**L**
Litoria leucova	U	L	H	**L**
Litoria littlejohni	H	H	L	**L**
Litoria longicrus	H	L	H	**L**
Litoria longipes	H	H	L	**L**
Litoria longirostris	H	H	L	**L**
Litoria lorica	H	H	L	**L**
Litoria louisiadensis	H	H	L	**L**
Litoria lutea	H	H	L	**L**
Litoria macki	H	L	H	**L**
Litoria maculosa	H	L	L	**L**
Litoria maini	H	L	L	**L**
Litoria majikthise	H	L	H	**L**
Litoria manya	H	H	L	**L**
Litoria megalops	H	L	H	**L**
Litoria meiriana	H	H	L	**L**
Litoria michaeltyleri	H	L	H	**L**
Litoria microbelos	H	H	L	**L**
Litoria micromembrana	L	L	L	**L**

Litoria modica	L	H	L	**L**
Litoria montana	H	L	H	**L**
Litoria moorei	H	H	H	**H**
Litoria mucro	H	L	H	**L**
Litoria multicolor	H	L	H	**L**
Litoria multiplica	L	H	L	**L**
Litoria myola	H	L	H	**L**
Litoria mystax	H	L	H	**L**
Litoria nannotis	H	L	H	**L**
Litoria napaea	H	L	H	**L**
Litoria narinosa	L	L	H	**L**
Litoria nasuta	H	H	L	**L**
Litoria nigrofrenata	H	L	L	**L**
Litoria nigropunctata	H	L	L	**L**
Litoria novaehollandiae	H	L	L	**L**
Litoria nudidigita	H	H	H	**H**
Litoria nyakalensis	H	H	H	**H**
Litoria obsoleta	H	L	H	**L**
Litoria obtusirostris	H	L	H	**L**
Litoria oenicolen	H	L	L	**L**
Litoria oktediensis	U	L	H	**L**
Litoria ollauro	H	L	H	**L**
Litoria olongburensis	H	H	L	**L**
Litoria pallida	H	H	L	**L**
Litoria papua	U	L	H	**L**
Litoria paraewingi	H	L	H	**L**
Litoria pearsoniana	H	L	L	**L**
Litoria perimetri	H	H	L	**L**
Litoria peronii	H	L	L	**L**
Litoria persimilis	U	L	H	**L**
Litoria personata	H	L	L	**L**
Litoria phyllochroa	H	L	L	**L**
Litoria piperata	H	L	L	**L**
Litoria platycephala	H	H	L	**L**
Litoria pratti	H	L	H	**L**
Litoria pronimia	H	L	H	**L**
Litoria prora	H	L	H	**L**
Litoria pulchra	H	L	L	**L**
Litoria purpureolata	H	L	H	**L**
Litoria pygmaea	H	H	L	**L**
Litoria quadrilineata	H	H	L	**L**
Litoria raniformis	H	L	L	**L**
Litoria rara	H	L	H	**L**
Litoria revelata	H	H	L	**L**
Litoria rheocola	H	L	H	**L**

Litoria richardsi	H	L	H	**L**
Litoria rivicola	H	L	L	**L**
Litoria rothii	H	L	L	**L**
Litoria rubella	H	L	L	**L**
Litoria rubrops	H	L	L	**L**
Litoria rueppelli	H	L	H	**L**
Litoria sanguinolenta	H	H	H	**H**
Litoria sauroni	H	L	H	**L**
Litoria scabra	H	L	H	**L**
Litoria semipalmata	H	L	L	**L**
Litoria singadanae	H	H	H	**H**
Litoria spartacus	H	L	L	**L**
Litoria spenceri	H	H	H	**H**
Litoria spinifera	H	H	L	**L**
Litoria splendida	H	L	L	**L**
Litoria staccato	H	H	L	**L**
Litoria subglandulosa	H	L	L	**L**
Litoria thesaurensis	H	H	L	**L**
Litoria timida	H	L	H	**L**
Litoria tornieri	H	H	L	**L**
Litoria trachydermis	L	L	H	**L**
Litoria tyleri	H	L	L	**L**
Litoria umarensis	H	H	H	**H**
Litoria umbonata	H	H	H	**H**
Litoria vagabunda	H	H	U	**L**
Litoria vagitus	H	L	L	**L**
Litoria verae	H	L	H	**L**
Litoria verreauxii	H	L	L	**L**
Litoria verrucosa	H	L	L	**L**
Litoria vocivincens	H	H	L	**L**
Litoria wapogaensis	H	L	H	**L**
Litoria watjulumensis	H	L	L	**L**
Litoria wilcoxii	H	L	L	**L**
Litoria wisselensis	H	H	H	**H**
Litoria wollastoni	H	L	L	**L**
Litoria xanthomera	H	L	H	**L**
Litoria zweifeli	H	L	H	**L**
Liua shihi	L	H	L	**L**
Liua tsinpaensis	L	H	L	**L**
Liuixalus romeri	L	H	H	**L**
Luetkenotyphlus brasiliensis	H	L	L	**L**
Lyciasalamandra antalyana	H	H	H	**H**
Lyciasalamandra atifi	L	H	H	**L**
Lyciasalamandra billae	L	H	H	**L**
Lyciasalamandra fazilae	L	H	H	**L**

Lyciasalamandra flavimembris	L	H	H	**L**
Lyciasalamandra helverseni	L	H	H	**L**
Lyciasalamandra luschani	L	H	H	**L**
Lynchius flavomaculatus	H	H	H	**H**
Lynchius nebulanastes	H	L	H	**L**
Lynchius parkeri	H	H	H	**H**
Macrogenioglottus alipioi	L	H	H	**L**
Madecassophryne truebae	L	H	H	**L**
Mannophryne caquetio	H	H	H	**H**
Mannophryne collaris	H	H	H	**H**
Mannophryne cordilleriana	H	H	H	**H**
Mannophryne herminae	H	H	H	**H**
Mannophryne lamarcai	H	H	H	**H**
Mannophryne larandina	H	L	H	**L**
Mannophryne leonardoi	H	H	H	**H**
Mannophryne neblina	H	H	H	**H**
Mannophryne oblitterata	H	L	H	**L**
Mannophryne olmonae	H	H	H	**H**
Mannophryne riveroi	H	H	H	**H**
Mannophryne trinitatis	H	H	H	**H**
Mannophryne trujillensis	H	H	H	**H**
Mannophryne venezuelensis	H	H	H	**H**
Mannophryne yustizi	L	H	H	**L**
Mantella aurantiaca	L	H	L	**L**
Mantella baroni	L	H	L	**L**
Mantella bernhardi	H	H	L	**L**
Mantella betsileo	L	H	L	**L**
Mantella cowanii	H	H	L	**L**
Mantella crocea	L	H	H	**L**
Mantella ebenaui	L	H	H	**L**
Mantella expectata	H	H	L	**L**
Mantella haraldmeieri	L	H	H	**L**
Mantella laevigata	H	H	H	**H**
Mantella madagascariensis	L	H	L	**L**
Mantella manery	U	L	H	**L**
Mantella milotympanum	L	H	H	**L**
Mantella nigricans	L	H	H	**L**
Mantella pulchra	L	H	H	**L**
Mantella viridis	L	H	L	**L**
Mantidactylus aerumnalis	L	H	L	**L**
Mantidactylus albofrenatus	U	L	L	**L**
Mantidactylus alutus	L	H	L	**L**
Mantidactylus ambohimitombi	U	L	U	**L**
Mantidactylus ambreensis	L	H	H	**L**
Mantidactylus argenteus	H	H	H	**H**

Mantidactylus bellyi	L	H	H	**L**
Mantidactylus betsileanus	L	H	H	**L**
Mantidactylus biporus	L	H	H	**L**
Mantidactylus bourgati	U	L	L	**L**
Mantidactylus brevipalmatus	L	H	L	**L**
Mantidactylus charlotteae	L	H	H	**L**
Mantidactylus cowanii	L	H	L	**L**
Mantidactylus curtus	L	H	L	**L**
Mantidactylus delormei	L	H	L	**L**
Mantidactylus femoralis	L	H	H	**L**
Mantidactylus grandidieri	L	H	H	**L**
Mantidactylus guttulatus	L	H	H	**L**
Mantidactylus lugubris	L	H	H	**L**
Mantidactylus madecassus	L	H	L	**L**
Mantidactylus majori	L	H	H	**L**
Mantidactylus melanopleura	L	H	H	**L**
Mantidactylus mocquardi	L	H	H	**L**
Mantidactylus noralottae	H	H	L	**L**
Mantidactylus opiparis	L	H	H	**L**
Mantidactylus pauliani	L	H	H	**L**
Mantidactylus tricinctus	U	L	H	**L**
Mantidactylus ulcerosus	L	H	H	**L**
Mantidactylus zipperi	L	H	L	**L**
Mantidactylus zolitschka	U	L	H	**L**
Mantophryne infulata	H	H	H	**H**
Mantophryne lateralis	L	H	L	**L**
Mantophryne louisiadensis	H	L	L	**L**
Megaelosia apuana	U	L	H	**L**
Megaelosia bocainensis	H	L	H	**L**
Megaelosia boticariana	U	L	L	**L**
Megaelosia goeldii	L	L	L	**L**
Megaelosia lutzae	U	L	L	**L**
Megaelosia massarti	U	L	H	**L**
Megastomatohyla mixe	L	L	H	**L**
Megastomatohyla mixomaculata	L	L	H	**L**
Megastomatohyla nubicola	L	L	H	**L**
Megastomatohyla pellita	H	L	L	**L**
Megophrys kobayashii	H	H	H	**H**
Megophrys ligayae	H	H	L	**L**
Megophrys montana	L	H	H	**L**
Megophrys nasuta	L	L	L	**L**
Megophrys stejnegeri	L	H	L	**L**
Melanobatrachus indicus	L	H	H	**L**
Melanophryne barbatula	H	H	H	**H**
Melanophryne carpish	H	H	H	**H**

Melanophryniscus admirabilis	H	L	L	**L**
Melanophryniscus atroluteus	H	H	L	**L**
Melanophryniscus cambaraensis	H	L	H	**L**
Melanophryniscus cupreuscapularis	H	H	L	**L**
Melanophryniscus devincenzii	H	L	H	**L**
Melanophryniscus dorsalis	H	L	H	**L**
Melanophryniscus fulvoguttatus	H	H	H	**H**
Melanophryniscus klappenbachi	H	H	L	**L**
Melanophryniscus krauczuki	H	H	H	**H**
Melanophryniscus macrogranulosus	H	L	H	**L**
Melanophryniscus montevidensis	H	L	H	**L**
Melanophryniscus moreirae	H	H	L	**L**
Melanophryniscus orejasmirandai	H	L	H	**L**
Melanophryniscus pachyrhynus	H	H	H	**H**
Melanophryniscus rubriventris	L	L	H	**L**
Melanophryniscus sanmartini	H	L	H	**L**
Melanophryniscus simplex	H	L	H	**L**
Melanophryniscus spectabilis	H	L	L	**L**
Melanophryniscus stelzneri	H	L	L	**L**
Melanophryniscus tumifrons	H	L	L	**L**
Meristogenys amoropalamus	H	L	H	**L**
Meristogenys jerboa	H	H	H	**H**
Meristogenys kinabaluensis	H	L	H	**L**
Meristogenys macrophthalmus	H	L	H	**L**
Meristogenys orphnocnemis	H	L	H	**L**
Meristogenys phaeomerus	H	L	H	**L**
Meristogenys poecilus	H	L	H	**L**
Meristogenys whiteheadi	L	L	H	**L**
Mertensiella caucasica	H	H	L	**L**
Mertensophryne anotis	L	H	L	**L**
Mertensophryne howelli	H	H	H	**H**
Mertensophryne lindneri	L	H	L	**L**
Mertensophryne lonnbergi	L	H	H	**L**
Mertensophryne loveridgei	L	H	L	**L**
Mertensophryne melanopleura	L	H	L	**L**
Mertensophryne micranotis	L	H	L	**L**
Mertensophryne mocquardi	H	L	H	**L**
Mertensophryne nairobiensis	H	L	H	**L**
Mertensophryne nyikae	L	H	L	**L**
Mertensophryne schmidti	H	L	H	**L**
Mertensophryne taitana	H	H	L	**L**
Mertensophryne usambarae	H	H	L	**L**
Mertensophryne uzunguensis	H	H	L	**L**

Mesotriton alpestris	H	L	L	**L**
Metacrinia nichollsi	H	H	H	**H**
Metaphrynella pollicaris	L	H	L	**L**
Metaphrynella sundana	H	H	H	**H**
Metaphryniscus sosai	H	H	H	**H**
Micrixalus elegans	U	L	U	**L**
Micrixalus fuscus	L	L	H	**L**
Micrixalus gadgili	L	L	H	**L**
Micrixalus kottigeharensis	H	L	L	**L**
Micrixalus narainensis	H	L	L	**L**
Micrixalus nudis	L	L	H	**L**
Micrixalus phyllophilus	L	L	H	**L**
Micrixalus saxicola	L	L	L	**L**
Micrixalus silvaticus	U	L	H	**L**
Micrixalus swamianus	H	L	L	**L**
Micrixalus thampii	U	L	H	**L**
Microbatrachella capensis	H	H	L	**L**
Microcaecilia albiceps	H	L	H	**L**
Microcaecilia rabei	U	H	H	**L**
Microcaecilia supernumeraria	H	L	U	**L**
Microcaecilia taylori	H	L	H	**L**
Microcaecilia unicolor	H	L	H	**L**
Microhyla achatina	L	H	H	**L**
Microhyla annamensis	L	L	L	**L**
Microhyla annectens	U	L	L	**L**
Microhyla berdmorei	L	L	L	**L**
Microhyla borneensis	L	L	H	**L**
Microhyla butleri	L	H	L	**L**
Microhyla chakrapanii	H	L	H	**L**
Microhyla erythropoda	H	L	L	**L**
Microhyla fissipes	L	L	L	**L**
Microhyla fusca	H	L	H	**L**
Microhyla heymonsi	L	L	L	**L**
Microhyla karunaratnei	H	H	L	**L**
Microhyla maculifera	H	L	H	**L**
Microhyla mantheyi	H	L	L	**L**
Microhyla marmorata	L	L	H	**L**
Microhyla mixtura	H	L	H	**L**
Microhyla nanapollexa	U	L	L	**L**
Microhyla okinavensis	L	L	H	**L**
Microhyla ornata	L	L	L	**L**
Microhyla palmipes	L	L	L	**L**
Microhyla perparva	H	L	H	**L**
Microhyla petrigena	L	L	L	**L**
Microhyla picta	H	H	H	**H**

Microhyla pulchra	L	H	L	**L**
Microhyla pulverata	U	L	L	**L**
Microhyla rubra	L	L	L	**L**
Microhyla sholigari	L	L	L	**L**
Microhyla superciliaris	U	L	L	**L**
Microhyla zeylanica	L	H	H	**L**
Micryletta inornata	L	L	L	**L**
Micryletta steinegeri	L	H	L	**L**
Mimosiphonops reinhardti	H	H	U	**L**
Mimosiphonops vermiculatus	U	H	H	**L**
Minyobates steyermarki	H	H	H	**H**
Mixophyes balbus	H	L	L	**L**
Mixophyes carbinensis	H	H	L	**L**
Mixophyes coggeri	H	L	H	**L**
Mixophyes fasciolatus	H	L	L	**L**
Mixophyes fleayi	H	L	L	**L**
Mixophyes hihihorlo	H	H	H	**H**
Mixophyes iteratus	H	L	L	**L**
Mixophyes schevilli	H	L	H	**L**
Myersiella microps	L	H	L	**L**
Myersiohyla aromatica	H	L	H	**L**
Myersiohyla inparquesi	H	L	H	**L**
Myersiohyla kanaima	L	L	H	**L**
Myersiohyla loveridgei	H	L	H	**L**
Myobatrachus gouldii	H	H	H	**H**
Nannophryne apolobambica	H	L	H	**L**
Nannophryne cophotis	H	L	H	**L**
Nannophryne corynetes	H	L	H	**L**
Nannophryne variegata	L	H	H	**L**
Nannophrys ceylonensis	L	L	H	**L**
Nannophrys guentheri	H	L	U	**L**
Nannophrys marmorata	L	H	L	**L**
Nannophrys naeyakai	H	L	L	**L**
Nanorana aenea	H	L	H	**L**
Nanorana annandalii	L	L	H	**L**
Nanorana arnoldi	L	H	H	**L**
Nanorana blanfordii	L	H	H	**L**
Nanorana bourreti	U	L	H	**L**
Nanorana conaensis	H	L	H	**L**
Nanorana ercepeae	L	H	H	**L**
Nanorana fansipani	U	L	H	**L**
Nanorana feae	H	L	L	**L**
Nanorana liebigii	H	H	L	**L**
Nanorana liui	L	L	H	**L**
Nanorana maculosa	L	L	H	**L**

Nanorana medogensis	U	L	H	**L**
Nanorana minica	L	H	H	**L**
Nanorana mokokchungensis	U	L	L	**L**
Nanorana parkeri	H	L	H	**L**
Nanorana pleskei	H	L	L	**L**
Nanorana polunini	L	H	L	**L**
Nanorana quadranus	L	L	H	**L**
Nanorana rarica	H	H	H	**H**
Nanorana rostandi	L	H	H	**L**
Nanorana taihangnica	H	L	H	**L**
Nanorana unculuanus	H	L	H	**L**
Nanorana ventripunctata	H	L	L	**L**
Nanorana vicina	L	H	H	**L**
Nanorana yunnanensis	H	L	L	**L**
Nasikabatrachus sahyadrensis	L	H	H	**L**
Natalobatrachus bonebergi	H	H	L	**L**
Nectocaecilia petersii	H	H	H	**H**
Nectophryne afra	H	H	L	**L**
Nectophryne batesii	H	H	L	**L**
Nectophrynoides asperginis	H	H	L	**L**
Nectophrynoides cryptus	H	H	H	**H**
Nectophrynoides frontierei	H	H	H	**H**
Nectophrynoides laevis	U	H	H	**L**
Nectophrynoides laticeps	H	H	H	**H**
Nectophrynoides minutus	H	H	H	**H**
Nectophrynoides paulae	H	H	H	**H**
Nectophrynoides poyntoni	H	H	L	**L**
Nectophrynoides pseudotornieri	H	H	H	**H**
Nectophrynoides tornieri	L	H	H	**L**
Nectophrynoides vestergaardi	H	H	H	**H**
Nectophrynoides viviparus	H	H	L	**L**
Nectophrynoides wendyae	H	H	L	**L**
Necturus alabamensis	H	H	L	**L**
Necturus beyeri	H	H	L	**L**
Necturus lewisi	H	H	L	**L**
Necturus maculosus	H	H	L	**L**
Necturus punctatus	H	H	L	**L**
Nelsonophryne aequatorialis	H	H	H	**H**
Nelsonophryne aterrima	L	H	L	**L**
Neobatrachus albipes	H	H	L	**L**
Neobatrachus aquilonius	H	L	L	**L**
Neobatrachus centralis	H	L	L	**L**
Neobatrachus fulvus	H	L	L	**L**
Neobatrachus kunapalari	H	L	L	**L**
Neobatrachus pelobatoides	H	L	L	**L**

Neobatrachus pictus	H	L	L	**L**
Neobatrachus sudelli	H	L	L	**L**
Neobatrachus sutor	H	L	L	**L**
Neobatrachus wilsmorei	H	H	L	**L**
Neurergus crocatus	H	L	L	**L**
Neurergus kaiseri	H	H	L	**L**
Neurergus microspilotus	H	H	L	**L**
Neurergus strauchii	H	H	L	**L**
Niceforonia adenobrachia	U	H	H	**L**
Niceforonia columbiana	H	L	H	**L**
Niceforonia nana	U	H	H	**L**
Nimbaphrynoides liberiensis	H	H	L	**L**
Nimbaphrynoides occidentalis	H	H	L	**L**
Noblella carrascoicola	H	H	H	**H**
Noblella heyeri	U	H	H	**L**
Noblella lochites	H	H	L	**L**
Noblella lynchi	H	L	H	**L**
Noblella myrmecoides	H	H	L	**L**
Noblella peruviana	U	H	H	**L**
Noblella ritarasquinae	H	H	H	**H**
Notaden bennettii	H	L	L	**L**
Notaden melanoscaphus	H	L	L	**L**
Notaden nichollsi	H	L	L	**L**
Notaden weigeli	H	H	L	**L**
Nothophryne broadleyi	L	H	L	**L**
Notophthalmus meridionalis	H	L	H	**L**
Notophthalmus perstriatus	H	L	L	**L**
Notophthalmus viridescens	H	L	L	**L**
Nototriton abscondens	L	H	H	**L**
Nototriton barbouri	L	H	H	**L**
Nototriton brodiei	U	L	H	**L**
Nototriton gamezi	H	H	H	**H**
Nototriton guanacaste	H	H	H	**H**
Nototriton lignicola	H	H	H	**H**
Nototriton limnospectator	H	H	H	**H**
Nototriton major	H	H	H	**H**
Nototriton picadoi	H	H	H	**H**
Nototriton richardi	H	H	H	**H**
Nototriton saslaya	H	H	H	**H**
Nototriton stuarti	H	L	H	**L**
Nototriton tapanti	H	H	H	**H**
Nyctanolis pernix	H	H	H	**H**
Nyctibates corrugatus	L	L	L	**L**
Nyctibatrachus aliciae	L	H	L	**L**
Nyctibatrachus beddomii	L	H	H	**L**

Nyctibatrachus deccanensis	L	H	H	**L**
Nyctibatrachus humayuni	L	L	H	**L**
Nyctibatrachus karnatakaensis	L	H	H	**L**
Nyctibatrachus kempholeyensis	U	L	L	**L**
Nyctibatrachus major	L	H	H	**L**
Nyctibatrachus minimus	H	L	H	**L**
Nyctibatrachus minor	L	H	L	**L**
Nyctibatrachus petraeus	L	H	H	**L**
Nyctibatrachus sanctipalustris	L	H	L	**L**
Nyctibatrachus sholai	H	L	H	**L**
Nyctibatrachus sylvaticus	H	L	H	**L**
Nyctibatrachus vasanthi	L	H	L	**L**
Nyctimantis rugiceps	H	H	H	**H**
Nyctixalus margaritifer	L	H	H	**L**
Nyctixalus pictus	H	H	L	**L**
Nyctixalus spinosus	L	H	L	**L**
Nymphargus anomalus	H	H	L	**L**
Nymphargus armatus	L	H	H	**L**
Nymphargus bejaranoi	L	H	H	**L**
Nymphargus buenaventura	H	L	H	**L**
Nymphargus cariticommatus	H	L	H	**L**
Nymphargus chami	H	L	H	**L**
Nymphargus chancas	H	L	H	**L**
Nymphargus cochranae	L	H	L	**L**
Nymphargus cristinae	U	L	H	**L**
Nymphargus garciae	H	H	H	**H**
Nymphargus griffithsi	H	H	L	**L**
Nymphargus ignotus	L	H	H	**L**
Nymphargus laurae	H	H	H	**H**
Nymphargus luminosus	L	H	H	**L**
Nymphargus luteopunctatus	U	L	H	**L**
Nymphargus megacheirus	H	H	H	**H**
Nymphargus mixomaculatus	H	L	H	**L**
Nymphargus nephelophila	H	L	L	**L**
Nymphargus oreonympha	H	L	L	**L**
Nymphargus phenax	U	L	H	**L**
Nymphargus pluvialis	U	L	H	**L**
Nymphargus posadae	L	H	H	**L**
Nymphargus prasinus	H	H	H	**H**
Nymphargus rosada	L	H	L	**L**
Nymphargus ruizi	L	H	H	**L**
Nymphargus siren	H	H	L	**L**
Nymphargus spilotus	U	L	H	**L**
Nymphargus truebae	U	L	H	**L**
Nymphargus vicenteruedai	H	L	H	**L**

Nymphargus wileyi	U	L	H	**L**
Occidozyga baluensis	L	L	H	**L**
Occidozyga borealis	L	L	H	**L**
Occidozyga celebensis	H	L	H	**L**
Occidozyga diminutiva	H	L	L	**L**
Occidozyga floresiana	H	H	L	**L**
Occidozyga laevis	L	L	L	**L**
Occidozyga lima	H	L	L	**L**
Occidozyga magnapustulosa	L	L	H	**L**
Occidozyga martensii	L	L	L	**L**
Occidozyga semipalmata	L	L	H	**L**
Occidozyga sumatrana	L	L	L	**L**
Occidozyga vittata	H	L	L	**L**
Odontophrynus achalensis	H	L	H	**L**
Odontophrynus americanus	H	L	L	**L**
Odontophrynus barrioi	H	L	H	**L**
Odontophrynus carvalhoi	H	L	L	**L**
Odontophrynus cordobae	H	L	H	**L**
Odontophrynus cultripes	H	L	L	**L**
Odontophrynus lavillai	H	H	H	**H**
Odontophrynus moratoi	H	L	L	**L**
Odontophrynus occidentalis	H	L	L	**L**
Odontophrynus salvatori	H	L	L	**L**
Odorrana absita	U	L	L	**L**
Odorrana amamiensis	L	H	H	**L**
Odorrana andersonii	L	L	H	**L**
Odorrana anlungensis	U	L	H	**L**
Odorrana aureola	U	L	L	**L**
Odorrana bacboensis	U	L	H	**L**
Odorrana banaorum	U	L	L	**L**
Odorrana bolavensis	U	L	L	**L**
Odorrana chapaensis	L	L	H	**L**
Odorrana chloronota	L	L	L	**L**
Odorrana exiliversabilis	L	L	L	**L**
Odorrana gigatympana	U	L	L	**L**
Odorrana grahami	L	L	L	**L**
Odorrana graminea	U	L	H	**L**
Odorrana hainanensis	L	H	L	**L**
Odorrana hejiangensis	U	L	L	**L**
Odorrana hosii	L	L	L	**L**
Odorrana indeprensa	U	L	L	**L**
Odorrana ishikawae	L	H	H	**L**
Odorrana jingdongensis	L	L	H	**L**
Odorrana junlianensis	L	L	H	**L**
Odorrana khalam	U	L	L	**L**

Odorrana kuangwuensis	L	L	H	**L**
Odorrana leporipes	U	L	U	**L**
Odorrana livida	U	L	L	**L**
Odorrana lungshengensis	L	L	L	**L**
Odorrana margaretae	L	L	H	**L**
Odorrana melasma	U	L	L	**L**
Odorrana monjerai	H	L	H	**L**
Odorrana morafkai	U	L	L	**L**
Odorrana narina	L	H	H	**L**
Odorrana nasica	L	L	L	**L**
Odorrana nasuta	L	H	L	**L**
Odorrana orba	U	L	H	**L**
Odorrana schmackeri	L	L	H	**L**
Odorrana sinica	U	L	U	**L**
Odorrana supranarina	H	H	H	**H**
Odorrana swinhoana	L	H	H	**L**
Odorrana tiannanensis	L	L	H	**L**
Odorrana tormota	L	L	H	**L**
Odorrana trankieni	U	L	H	**L**
Odorrana utsunomiyaorum	H	H	H	**H**
Odorrana versabilis	L	L	L	**L**
Odorrana wuchuanensis	H	H	L	**L**
Oedipina alfaroi	L	H	H	**L**
Oedipina alleni	H	H	H	**H**
Oedipina altura	H	H	H	**H**
Oedipina carablanca	L	H	H	**L**
Oedipina collaris	H	L	H	**L**
Oedipina complex	H	H	L	**L**
Oedipina cyclocauda	L	H	H	**L**
Oedipina elongata	H	H	H	**H**
Oedipina gephyra	H	H	H	**H**
Oedipina gracilis	H	H	H	**H**
Oedipina grandis	H	H	H	**H**
Oedipina ignea	H	L	H	**L**
Oedipina maritima	H	H	H	**H**
Oedipina pacificensis	L	H	H	**L**
Oedipina parvipes	H	H	L	**L**
Oedipina paucidentata	H	H	H	**H**
Oedipina poelzi	H	H	H	**H**
Oedipina pseudouniformis	L	H	H	**L**
Oedipina savagei	H	L	H	**L**
Oedipina stenopodia	L	H	H	**L**
Oedipina stuarti	H	H	U	**L**
Oedipina taylori	L	H	H	**L**
Oedipina tomasi	H	H	H	**H**

Oedipina uniformis	H	H	H	**H**
Ombrana sikimensis	L	L	H	**L**
Ommatotriton ophryticus	H	L	L	**L**
Ommatotriton vittatus	H	L	H	**L**
Onychodactylus fischeri	L	H	H	**L**
Onychodactylus japonicus	L	H	L	**L**
Oophaga arborea	H	H	L	**L**
Oophaga granulifera	H	H	H	**H**
Oophaga histrionica	H	H	H	**H**
Oophaga lehmanni	L	H	H	**L**
Oophaga occultator	H	L	H	**L**
Oophaga pumilio	H	H	H	**H**
Oophaga speciosa	H	H	H	**H**
Oophaga sylvatica	H	H	H	**H**
Oophaga vicentei	H	L	L	**L**
Ophryophryne gerti	U	L	L	**L**
Ophryophryne hansi	U	L	L	**L**
Ophryophryne microstoma	L	L	L	**L**
Ophryophryne pachyproctus	L	L	L	**L**
Ophryophryne synoria	H	U	U	**L**
Opisthothylax immaculatus	H	H	L	**L**
Oreobates choristolemma	H	H	H	**H**
Oreobates cruralis	L	H	H	**L**
Oreobates discoidalis	H	H	H	**H**
Oreobates heterodactylus	U	H	L	**L**
Oreobates ibischi	H	H	H	**H**
Oreobates lehri	H	H	H	**H**
Oreobates madidi	H	H	H	**H**
Oreobates pereger	H	H	L	**L**
Oreobates quixensis	H	H	L	**L**
Oreobates sanctaecrucis	L	H	H	**L**
Oreobates sanderi	H	H	H	**H**
Oreobates saxatilis	H	L	H	**L**
Oreobates simmonsi	H	H	H	**H**
Oreobates zongoensis	H	H	H	**H**
Oreolalax chuanbeiensis	L	L	H	**L**
Oreolalax granulosus	L	L	L	**L**
Oreolalax jingdongensis	L	L	H	**L**
Oreolalax liangbeiensis	L	L	L	**L**
Oreolalax lichuanensis	L	L	L	**L**
Oreolalax major	L	L	H	**L**
Oreolalax multipunctatus	L	L	H	**L**
Oreolalax nanjiangensis	U	L	H	**L**
Oreolalax omeimontis	L	L	H	**L**
Oreolalax pingii	L	L	L	**L**

Oreolalax popei	H	L	H	**L**
Oreolalax puxiongensis	L	L	L	**L**
Oreolalax rhodostigmatus	L	L	L	**L**
Oreolalax rugosus	L	L	H	**L**
Oreolalax schmidti	L	L	H	**L**
Oreolalax weigoldi	H	L	U	**L**
Oreolalax xiangchengensis	L	L	L	**L**
Oreophryne albopunctata	H	H	H	**H**
Oreophryne alticola	H	H	H	**H**
Oreophryne anthonyi	H	H	H	**H**
Oreophryne anulata	L	H	L	**L**
Oreophryne asplenicola	H	L	H	**L**
Oreophryne atrigularis	H	L	H	**L**
Oreophryne biroi	H	H	H	**H**
Oreophryne brachypus	L	H	H	**L**
Oreophryne brevicrus	H	L	H	**L**
Oreophryne brevirostris	H	L	H	**L**
Oreophryne celebensis	H	H	H	**H**
Oreophryne clamata	H	L	H	**L**
Oreophryne crucifer	H	H	H	**H**
Oreophryne flava	H	L	H	**L**
Oreophryne frontifasciata	H	L	H	**L**
Oreophryne geislerorum	L	H	L	**L**
Oreophryne geminus	H	L	H	**L**
Oreophryne habbemensis	H	L	H	**L**
Oreophryne hypsiops	H	H	H	**H**
Oreophryne idenburgensis	H	L	H	**L**
Oreophryne inornata	H	H	L	**L**
Oreophryne insulana	H	L	L	**L**
Oreophryne jeffersoniana	H	H	L	**L**
Oreophryne kampeni	H	H	L	**L**
Oreophryne kapisa	H	H	H	**H**
Oreophryne loriae	H	H	L	**L**
Oreophryne minuta	H	H	H	**H**
Oreophryne moluccensis	H	H	H	**H**
Oreophryne monticola	H	H	H	**H**
Oreophryne nana	H	H	L	**L**
Oreophryne notata	U	H	H	**L**
Oreophryne parkeri	H	H	H	**H**
Oreophryne pseudasplenicola	H	H	H	**H**
Oreophryne rookmaakeri	H	H	L	**L**
Oreophryne sibilans	H	H	H	**H**
Oreophryne terrestris	H	H	H	**H**
Oreophryne unicolor	H	H	H	**H**
Oreophryne variabilis	H	H	H	**H**

Oreophryne waira	H	H	H	**H**
Oreophryne wapoga	H	H	H	**H**
Oreophryne wolterstorffi	U	H	U	**L**
Oreophryne zimmeri	H	H	U	**L**
Oreophrynella cryptica	H	H	H	**H**
Oreophrynella dendronastes	H	H	H	**H**
Oreophrynella huberi	H	H	H	**H**
Oreophrynella macconnelli	L	H	H	**L**
Oreophrynella nigra	H	H	H	**H**
Oreophrynella quelchii	H	H	H	**H**
Oreophrynella vasquezi	H	H	H	**H**
Oreophrynella weiassipuensis	H	H	H	**H**
Oscaecilia bassleri	H	H	H	**H**
Oscaecilia elongata	H	L	L	**L**
Oscaecilia equatorialis	U	H	U	**L**
Oscaecilia hypereumeces	H	H	L	**L**
Oscaecilia koepckeorum	H	L	H	**L**
Oscaecilia ochrocephala	H	H	L	**L**
Oscaecilia osae	H	H	H	**H**
Oscaecilia polyzona	H	H	L	**L**
Oscaecilia zweifeli	H	H	U	**L**
Osornophryne antisana	H	H	H	**H**
Osornophryne bufoniformis	H	H	H	**H**
Osornophryne guacamayo	H	H	H	**H**
Osornophryne percrassa	L	H	H	**L**
Osornophryne sumacoensis	H	H	L	**L**
Osornophryne talipes	H	H	H	**H**
Osteocephalus alboguttatus	H	L	H	**L**
Osteocephalus buckleyi	H	L	H	**L**
Osteocephalus cabrerai	H	L	H	**L**
Osteocephalus deridens	H	H	H	**H**
Osteocephalus elkejungingerae	L	L	H	**L**
Osteocephalus exophthalmus	H	H	H	**H**
Osteocephalus fuscifacies	H	H	H	**H**
Osteocephalus heyeri	H	H	L	**L**
Osteocephalus leoniae	H	H	H	**H**
Osteocephalus leprieurii	H	H	H	**H**
Osteocephalus mutabor	L	L	H	**L**
Osteocephalus oophagus	H	H	H	**H**
Osteocephalus pearsoni	L	H	H	**L**
Osteocephalus phasmatus	H	L	H	**L**
Osteocephalus planiceps	H	H	H	**H**
Osteocephalus subtilis	H	L	H	**L**
Osteocephalus taurinus	H	L	H	**L**
Osteocephalus verruciger	L	L	L	**L**

Osteocephalus yasuni	H	H	L	**L**
Osteopilus brunneus	L	H	H	**L**
Osteopilus crucialis	L	H	L	**L**
Osteopilus dominicensis	H	H	H	**H**
Osteopilus marianae	H	H	L	**L**
Osteopilus pulchrilineatus	H	H	H	**H**
Osteopilus septentrionalis	H	L	L	**L**
Osteopilus vastus	H	H	H	**H**
Osteopilus wilderi	H	L	L	**L**
Otophryne pyburni	H	L	H	**L**
Otophryne robusta	L	L	H	**L**
Otophryne steyermarki	H	H	H	**H**
Oxydactyla alpestris	H	H	H	**H**
Oxydactyla brevicrus	H	L	H	**L**
Oxydactyla coggeri	H	L	L	**L**
Oxydactyla crassa	U	H	H	**L**
Oxydactyla stenodactyla	H	H	H	**H**
Pachyhynobius shangchengensis	L	H	L	**L**
Pachyhynobius yunanicus	H	H	L	**L**
Pachymedusa dacnicolor	L	L	H	**L**
Pachytriton brevipes	L	H	L	**L**
Pachytriton labiatus	H	H	L	**L**
Palmatorappia solomonis	H	H	L	**L**
Paracassina kounhiensis	H	H	L	**L**
Paracassina obscura	H	H	L	**L**
Paracrinia haswelli	H	H	L	**L**
Paradactylodon gorganensis	H	H	H	**H**
Paradactylodon mustersi	H	H	H	**H**
Paradactylodon persicus	H	H	H	**H**
Paradoxophyla palmata	L	L	H	**L**
Paradoxophyla tiarano	U	L	H	**L**
Paramesotriton caudopunctatus	L	H	L	**L**
Paramesotriton chinensis	L	H	L	**L**
Paramesotriton deloustali	L	L	H	**L**
Paramesotriton fuzhongensis	L	H	L	**L**
Paramesotriton guanxiensis	L	H	H	**L**
Paramesotriton hongkongensis	L	H	H	**L**
Paramesotriton laoensis	H	L	L	**L**
Parapelophryne scalpta	L	H	L	**L**
Paratelmatobius cardosoi	H	L	H	**L**
Paratelmatobius gaigeae	H	H	H	**H**
Paratelmatobius lutzii	H	L	H	**L**
Paratelmatobius mantiqueira	H	L	U	**L**
Paratelmatobius poecilogaster	H	L	H	**L**
Parhoplophryne usambarica	H	H	H	**H**

Parvicaecilia nicefori	H	H	H	**H**
Parvicaecilia pricei	L	H	H	**L**
Parvimolge townsendi	L	H	H	**L**
Pedostibes everetti	H	L	H	**L**
Pedostibes hosii	L	L	L	**L**
Pedostibes kempi	H	L	H	**L**
Pedostibes rugosus	L	L	H	**L**
Pedostibes tuberculosus	L	L	H	**L**
Pelobates cultripes	H	H	L	**L**
Pelobates fuscus	H	H	H	**H**
Pelobates syriacus	H	L	L	**L**
Pelobates varaldii	H	H	H	**H**
Pelodytes caucasicus	H	L	L	**L**
Pelodytes ibericus	H	L	L	**L**
Pelodytes punctatus	H	L	L	**L**
Pelophryne albotaeniata	H	H	L	**L**
Pelophryne api	H	H	H	**H**
Pelophryne brevipes	L	H	L	**L**
Pelophryne guentheri	H	H	L	**L**
Pelophryne lighti	H	H	L	**L**
Pelophryne macrotis	H	H	U	**L**
Pelophryne misera	H	H	H	**H**
Pelophryne rhopophilia	H	H	H	**H**
Pelophryne signata	H	H	H	**H**
Pelophylax bedriagae	H	L	L	**L**
Pelophylax bergeri	H	H	L	**L**
Pelophylax caralitanus	H	L	L	**L**
Pelophylax cerigensis	H	H	H	**H**
Pelophylax chosenicus	H	H	H	**H**
Pelophylax cretensis	H	H	H	**H**
Pelophylax demarchii	H	H	U	**L**
Pelophylax epeiroticus	H	L	H	**L**
Pelophylax esculentus	H	L	L	**L**
Pelophylax fukienensis	L	H	L	**L**
Pelophylax grafi	H	L	L	**L**
Pelophylax hispanicus	H	L	L	**L**
Pelophylax hubeiensis	H	H	H	**H**
Pelophylax kurtmuelleri	H	L	L	**L**
Pelophylax lateralis	L	L	H	**L**
Pelophylax lessonae	H	L	H	**L**
Pelophylax nigromaculatus	L	H	H	**L**
Pelophylax perezi	H	L	L	**L**
Pelophylax plancyi	H	H	H	**H**
Pelophylax porosus	H	L	L	**L**
Pelophylax ridibundus	H	L	L	**L**

Pelophylax saharicus	H	L	H	**L**
Pelophylax shqipericus	H	L	H	**L**
Pelophylax tenggerensis	H	H	H	**H**
Pelophylax terentievi	H	L	H	**L**
Peltophryne cataulaciceps	H	L	L	**L**
Peltophryne empusa	U	H	L	**L**
Peltophryne florentinoi	H	H	L	**L**
Peltophryne fluviatica	H	L	H	**L**
Peltophryne fracta	H	L	L	**L**
Peltophryne fustiger	L	H	L	**L**
Peltophryne guentheri	H	H	H	**H**
Peltophryne gundlachi	L	H	L	**L**
Peltophryne lemur	H	H	L	**L**
Peltophryne longinasus	L	L	L	**L**
Peltophryne peltocephala	L	L	L	**L**
Peltophryne taladai	L	L	L	**L**
Petropedetes cameronensis	H	L	L	**L**
Petropedetes dutoiti	H	H	H	**H**
Petropedetes johnstoni	L	L	H	**L**
Petropedetes martiensseni	H	H	H	**H**
Petropedetes natator	H	L	L	**L**
Petropedetes newtoni	L	L	L	**L**
Petropedetes palmipes	H	L	L	**L**
Petropedetes parkeri	L	L	L	**L**
Petropedetes perreti	H	L	L	**L**
Petropedetes yakusini	H	H	H	**H**
Phaeognathus hubrichti	H	H	H	**H**
Phasmahyla cochranae	L	L	L	**L**
Phasmahyla exilis	H	L	H	**L**
Phasmahyla guttata	L	L	L	**L**
Phasmahyla jandaia	L	L	L	**L**
Pherohapsis menziesi	H	L	L	**L**
Philautus abditus	H	H	L	**L**
Philautus abundus	H	H	H	**H**
Philautus acutirostris	L	H	L	**L**
Philautus acutus	H	H	H	**H**
Philautus adspersus	H	H	U	**L**
Philautus alto	H	H	H	**H**
Philautus amoenus	H	H	H	**H**
Philautus asankai	H	H	H	**H**
Philautus aurantium	H	H	H	**H**
Philautus auratus	L	H	L	**L**
Philautus aurifasciatus	L	H	L	**L**
Philautus banaensis	U	H	U	**L**
Philautus bunitus	H	H	H	**H**

Philautus caeruleus	H	H	H	**H**
Philautus cardamonus	H	L	H	**L**
Philautus cavirostris	H	H	H	**H**
Philautus cinerascens	H	L	L	**L**
Philautus cornutus	H	L	H	**L**
Philautus cuspis	H	H	L	**L**
Philautus decoris	L	H	L	**L**
Philautus dimbullae	H	H	U	**L**
Philautus disgregus	H	H	H	**H**
Philautus erythrophthalmus	H	H	H	**H**
Philautus eximius	H	L	U	**L**
Philautus extirpo	H	H	U	**L**
Philautus femoralis	H	H	H	**H**
Philautus fergusonianus	H	H	L	**L**
Philautus folicola	H	H	H	**H**
Philautus frankenbergi	H	H	H	**H**
Philautus fulvus	L	H	H	**L**
Philautus gryllus	U	L	L	**L**
Philautus gunungensis	H	H	H	**H**
Philautus hainanus	H	L	L	**L**
Philautus hallidayi	L	H	H	**L**
Philautus halyi	H	H	U	**L**
Philautus hoffmanni	H	H	H	**H**
Philautus hoipolloi	H	H	L	**L**
Philautus hosii	H	H	H	**H**
Philautus hypomelas	H	H	U	**L**
Philautus ingeri	H	H	H	**H**
Philautus jacobsoni	U	H	H	**L**
Philautus jinxiuensis	L	H	L	**L**
Philautus kempii	U	L	H	**L**
Philautus kerangae	H	H	L	**L**
Philautus leitensis	L	H	L	**L**
Philautus leucorhinus	H	L	U	**L**
Philautus limbus	H	H	H	**H**
Philautus longchuanensis	H	H	H	**H**
Philautus longicrus	L	H	L	**L**
Philautus lunatus	H	H	H	**H**
Philautus macropus	H	H	H	**H**
Philautus maia	U	H	U	**L**
Philautus malcolmsmithi	H	H	U	**L**
Philautus maosonensis	U	H	H	**L**
Philautus menglaensis	U	L	H	**L**
Philautus microtympanum	H	H	H	**H**
Philautus mittermeieri	H	H	L	**L**
Philautus mjobergi	H	H	H	**H**

Philautus mooreorum	H	H	H	**H**
Philautus nanus	H	H	U	**L**
Philautus nasutus	H	H	U	**L**
Philautus nemus	H	H	H	**H**
Philautus ocellatus	L	H	L	**L**
Philautus ocularis	H	H	H	**H**
Philautus oxyrhynchus	H	L	U	**L**
Philautus pallidipes	H	H	H	**H**
Philautus papillosus	H	H	H	**H**
Philautus pardus	U	L	U	**L**
Philautus parvulus	L	H	L	**L**
Philautus petersi	L	H	L	**L**
Philautus petilus	H	L	H	**L**
Philautus pleurotaenia	H	H	H	**H**
Philautus poecilius	L	H	L	**L**
Philautus poppiae	H	H	H	**H**
Philautus popularis	H	H	H	**H**
Philautus procax	H	H	H	**H**
Philautus refugii	H	H	L	**L**
Philautus regius	U	L	H	**L**
Philautus reticulatus	H	H	H	**H**
Philautus rugatus	H	L	U	**L**
Philautus rus	H	H	H	**H**
Philautus sahai	H	L	H	**L**
Philautus sarasinorum	H	H	H	**H**
Philautus saueri	H	H	H	**H**
Philautus schmackeri	H	H	L	**L**
Philautus schmarda	L	H	H	**L**
Philautus semiruber	H	L	H	**L**
Philautus silus	L	H	H	**L**
Philautus silvaticus	L	H	L	**L**
Philautus simba	H	H	H	**H**
Philautus similis	H	H	H	**H**
Philautus sordidus	L	H	H	**L**
Philautus steineri	H	H	H	**H**
Philautus stellatus	H	H	U	**L**
Philautus stictomerus	H	H	H	**H**
Philautus stuarti	H	H	H	**H**
Philautus surdus	L	H	L	**L**
Philautus surrufus	L	H	H	**L**
Philautus tectus	H	H	H	**H**
Philautus temporalis	H	H	U	**L**
Philautus truongsonensis	L	L	L	**L**
Philautus tytthus	H	H	H	**H**
Philautus umbra	H	H	H	**H**

Philautus variabilis	H	H	U	**L**
Philautus vermiculatus	L	H	L	**L**
Philautus viridis	H	H	H	**H**
Philautus vittiger	H	H	H	**H**
Philautus worcesteri	L	H	L	**L**
Philautus zal	H	L	U	**L**
Philautus zimmeri	H	H	U	**L**
Philautus zorro	H	H	H	**H**
Philoria frosti	H	H	H	**H**
Philoria kundagungan	H	H	L	**L**
Philoria loveridgei	H	H	L	**L**
Philoria pughi	H	H	L	**L**
Philoria richmondensis	H	H	L	**L**
Philoria sphagnicolus	H	H	L	**L**
Phlyctimantis boulengeri	H	H	L	**L**
Phlyctimantis keithae	L	H	L	**L**
Phlyctimantis leonardi	H	H	L	**L**
Phlyctimantis verrucosus	L	H	L	**L**
Phrynella pulchra	H	H	L	**L**
Phrynobatrachus accraensis	H	L	L	**L**
Phrynobatrachus acridoides	H	L	L	**L**
Phrynobatrachus acutirostris	H	L	L	**L**
Phrynobatrachus africanus	H	L	L	**L**
Phrynobatrachus albolabris	H	H	L	**L**
Phrynobatrachus albomarginatus	H	H	U	**L**
Phrynobatrachus alleni	H	L	L	**L**
Phrynobatrachus annulatus	H	L	L	**L**
Phrynobatrachus anotis	H	L	H	**L**
Phrynobatrachus asper	U	H	L	**L**
Phrynobatrachus auritus	H	L	L	**L**
Phrynobatrachus batesii	L	L	L	**L**
Phrynobatrachus bequaerti	H	L	L	**L**
Phrynobatrachus breviceps	H	L	L	**L**
Phrynobatrachus brevipalmatus	H	H	H	**H**
Phrynobatrachus bullans	H	L	L	**L**
Phrynobatrachus calcaratus	L	L	L	**L**
Phrynobatrachus congicus	H	L	U	**L**
Phrynobatrachus cornutus	H	H	L	**L**
Phrynobatrachus cricogaster	L	L	L	**L**
Phrynobatrachus cryptotis	U	L	H	**L**
Phrynobatrachus dalcqi	H	H	U	**L**
Phrynobatrachus dendrobates	H	H	H	**H**
Phrynobatrachus dispar	H	H	L	**L**
Phrynobatrachus elberti	H	H	L	**L**
Phrynobatrachus francisci	H	H	L	**L**

Phrynobatrachus fraterculus	H	L	L	**L**
Phrynobatrachus gastoni	H	H	L	**L**
Phrynobatrachus ghanensis	H	L	L	**L**
Phrynobatrachus giorgii	H	H	L	**L**
Phrynobatrachus graueri	H	L	L	**L**
Phrynobatrachus guineensis	H	H	L	**L**
Phrynobatrachus gutturosus	L	L	L	**L**
Phrynobatrachus hylaios	H	L	L	**L**
Phrynobatrachus inexpectatus	H	L	L	**L**
Phrynobatrachus irangi	L	L	H	**L**
Phrynobatrachus keniensis	H	L	H	**L**
Phrynobatrachus kinangopensis	H	L	H	**L**
Phrynobatrachus krefftii	H	L	L	**L**
Phrynobatrachus leveleve	L	H	L	**L**
Phrynobatrachus liberiensis	H	L	L	**L**
Phrynobatrachus mababiensis	H	L	L	**L**
Phrynobatrachus manengoubensis	H	L	L	**L**
Phrynobatrachus minutus	H	L	L	**L**
Phrynobatrachus nanus	H	L	L	**L**
Phrynobatrachus natalensis	L	L	L	**L**
Phrynobatrachus nlonakoensis	H	L	L	**L**
Phrynobatrachus ogoensis	H	H	H	**H**
Phrynobatrachus pakenhami	H	H	L	**L**
Phrynobatrachus pallidus	H	H	L	**L**
Phrynobatrachus parkeri	H	L	L	**L**
Phrynobatrachus parvulus	H	L	L	**L**
Phrynobatrachus perpalmatus	L	H	L	**L**
Phrynobatrachus phyllophilus	H	L	L	**L**
Phrynobatrachus plicatus	H	L	L	**L**
Phrynobatrachus pygmaeus	H	L	L	**L**
Phrynobatrachus rouxi	H	H	L	**L**
Phrynobatrachus rungwensis	H	H	L	**L**
Phrynobatrachus sandersoni	L	L	L	**L**
Phrynobatrachus scapularis	H	L	L	**L**
Phrynobatrachus scheffleri	H	L	L	**L**
Phrynobatrachus steindachneri	L	L	L	**L**
Phrynobatrachus sternfeldi	H	L	L	**L**
Phrynobatrachus stewartae	U	L	L	**L**
Phrynobatrachus sulfureogularis	U	H	U	**L**
Phrynobatrachus taiensis	H	L	L	**L**
Phrynobatrachus tokba	H	L	L	**L**
Phrynobatrachus ukingensis	U	H	L	**L**
Phrynobatrachus ungujae	L	L	L	**L**
Phrynobatrachus uzungwensis	L	L	H	**L**
Phrynobatrachus versicolor	L	L	L	**L**

Phrynobatrachus villiersi	H	H	L	**L**
Phrynobatrachus vogti	H	H	L	**L**
Phrynobatrachus werneri	L	L	L	**L**
Phrynoidis aspera	L	L	L	**L**
Phrynoidis juxtaspera	L	L	H	**L**
Phrynomantis affinis	H	H	L	**L**
Phrynomantis annectens	H	H	L	**L**
Phrynomantis bifasciatus	H	H	L	**L**
Phrynomantis microps	H	L	L	**L**
Phrynomantis somalicus	H	L	H	**L**
Phrynomedusa appendiculata	H	L	L	**L**
Phrynomedusa bokermanni	H	L	H	**L**
Phrynomedusa fimbriata	U	L	U	**L**
Phrynomedusa marginata	H	L	L	**L**
Phrynomedusa vanzolinii	H	L	H	**L**
Phrynopus ayacucho	H	H	H	**H**
Phrynopus barthlenae	H	H	H	**H**
Phrynopus bracki	H	H	H	**H**
Phrynopus bufoides	H	L	H	**L**
Phrynopus dagmarae	H	H	H	**H**
Phrynopus heimorum	H	H	L	**L**
Phrynopus horstpauli	H	H	H	**H**
Phrynopus juninensis	H	H	H	**H**
Phrynopus kauneorum	H	H	H	**H**
Phrynopus kotosh	H	L	H	**L**
Phrynopus montium	H	H	H	**H**
Phrynopus oblivius	H	L	H	**L**
Phrynopus paucari	H	L	H	**L**
Phrynopus peruanus	H	L	H	**L**
Phrynopus pesantesi	H	L	H	**L**
Phrynopus tautzorum	H	H	H	**H**
Phrynopus thompsoni	H	L	H	**L**
Phyllobates aurotaenia	H	H	H	**H**
Phyllobates bicolor	H	H	H	**H**
Phyllobates lugubris	H	H	H	**H**
Phyllobates terribilis	H	H	H	**H**
Phyllobates vittatus	H	L	H	**L**
Phyllodytes acuminatus	H	H	H	**H**
Phyllodytes auratus	H	H	H	**H**
Phyllodytes brevirostris	H	L	H	**L**
Phyllodytes edelmoi	U	H	H	**L**
Phyllodytes gyrinaethes	H	L	L	**L**
Phyllodytes kautskyi	H	L	H	**L**
Phyllodytes luteolus	H	H	H	**H**
Phyllodytes maculosus	H	H	H	**H**

Phyllodytes melanomystax	H	H	H	**H**
Phyllodytes punctatus	H	H	H	**H**
Phyllodytes tuberculosus	H	L	U	**L**
Phyllodytes wuchereri	H	H	H	**H**
Phyllomedusa araguari	H	H	L	**L**
Phyllomedusa atelopoides	H	H	L	**L**
Phyllomedusa ayeaye	H	L	L	**L**
Phyllomedusa azurea	H	L	L	**L**
Phyllomedusa bahiana	H	L	H	**L**
Phyllomedusa baltea	H	L	H	**L**
Phyllomedusa bicolor	H	H	H	**H**
Phyllomedusa boliviana	H	H	L	**L**
Phyllomedusa burmeisteri	H	L	L	**L**
Phyllomedusa camba	H	H	L	**L**
Phyllomedusa centralis	H	L	L	**L**
Phyllomedusa coelestis	H	L	H	**L**
Phyllomedusa distincta	H	H	L	**L**
Phyllomedusa duellmani	H	L	L	**L**
Phyllomedusa ecuatoriana	H	L	L	**L**
Phyllomedusa hypochondrialis	H	H	L	**L**
Phyllomedusa iheringii	H	H	H	**H**
Phyllomedusa itacolomi	H	H	L	**L**
Phyllomedusa megacephala	H	L	L	**L**
Phyllomedusa neildi	H	H	H	**H**
Phyllomedusa nordestina	H	H	L	**L**
Phyllomedusa oreades	H	L	L	**L**
Phyllomedusa palliata	H	H	L	**L**
Phyllomedusa perinesos	U	H	L	**L**
Phyllomedusa rohdei	H	L	L	**L**
Phyllomedusa sauvagii	H	H	L	**L**
Phyllomedusa tarsius	H	H	L	**L**
Phyllomedusa tetraploidea	H	L	L	**L**
Phyllomedusa tomopterna	H	H	H	**H**
Phyllomedusa trinitatis	H	H	H	**H**
Phyllomedusa vaillantii	H	L	H	**L**
Phyllomedusa venusta	H	H	H	**H**
Physalaemus aguirrei	H	L	L	**L**
Physalaemus albifrons	H	L	L	**L**
Physalaemus albonotatus	H	L	L	**L**
Physalaemus angrensis	H	L	L	**L**
Physalaemus atlanticus	H	H	L	**L**
Physalaemus barrioi	H	L	L	**L**
Physalaemus biligonigerus	H	H	L	**L**
Physalaemus bokermanni	H	L	H	**L**
Physalaemus caete	H	H	H	**H**

Physalaemus camacan	H	L	H	**L**
Physalaemus centralis	H	H	L	**L**
Physalaemus cicada	H	L	L	**L**
Physalaemus crombiei	H	H	H	**H**
Physalaemus cuqui	H	H	H	**H**
Physalaemus cuvieri	H	H	L	**L**
Physalaemus deimaticus	H	L	L	**L**
Physalaemus ephippifer	H	H	H	**H**
Physalaemus erikae	H	H	H	**H**
Physalaemus erythros	H	L	L	**L**
Physalaemus evangelistai	H	L	L	**L**
Physalaemus fernandezae	H	H	L	**L**
Physalaemus fischeri	H	H	H	**H**
Physalaemus freibergi	H	L	L	**L**
Physalaemus gracilis	H	L	L	**L**
Physalaemus henselii	H	L	L	**L**
Physalaemus irroratus	H	L	H	**L**
Physalaemus jordanensis	H	L	L	**L**
Physalaemus kroyeri	H	L	L	**L**
Physalaemus lisei	H	H	L	**L**
Physalaemus maculiventris	H	L	L	**L**
Physalaemus marmoratus	H	H	L	**L**
Physalaemus maximus	U	L	L	**L**
Physalaemus moreirae	H	L	H	**L**
Physalaemus nanus	H	L	L	**L**
Physalaemus obtectus	H	L	H	**L**
Physalaemus olfersii	H	H	L	**L**
Physalaemus riograndensis	H	L	L	**L**
Physalaemus rupestris	U	L	L	**L**
Physalaemus santafecinus	H	L	L	**L**
Physalaemus signifer	H	H	H	**H**
Physalaemus soaresi	H	H	L	**L**
Physalaemus spiniger	H	L	H	**L**
Phyzelaphryne miriamae	H	H	L	**L**
Pipa arrabali	H	H	H	**H**
Pipa aspera	H	L	H	**L**
Pipa carvalhoi	U	L	L	**L**
Pipa myersi	H	L	L	**L**
Pipa parva	H	H	H	**H**
Pipa pipa	H	H	H	**H**
Pipa snethlageae	H	H	L	**L**
Platymantis acrochordus	H	L	L	**L**
Platymantis aculeodactylus	H	H	H	**H**
Platymantis adiastolus	H	H	H	**H**
Platymantis admiraltiensis	H	H	H	**H**

Platymantis akarithymus	H	H	H	**H**
Platymantis banahao	H	H	L	**L**
Platymantis batantae	H	L	H	**L**
Platymantis bimaculatus	H	H	H	**H**
Platymantis boulengeri	H	H	H	**H**
Platymantis browni	L	H	H	**L**
Platymantis bufonulus	H	H	H	**H**
Platymantis cagayanensis	H	H	L	**L**
Platymantis cheesmanae	H	L	H	**L**
Platymantis cornutus	L	H	H	**L**
Platymantis corrugatus	H	H	L	**L**
Platymantis cryptotis	H	H	H	**H**
Platymantis diesmosi	H	H	L	**L**
Platymantis dorsalis	H	H	L	**L**
Platymantis gilliardi	H	L	H	**L**
Platymantis guentheri	L	H	L	**L**
Platymantis guppyi	H	H	L	**L**
Platymantis hazelae	H	H	L	**L**
Platymantis indeprensus	H	H	L	**L**
Platymantis insulatus	H	H	U	**L**
Platymantis isarog	H	H	L	**L**
Platymantis latro	H	H	H	**H**
Platymantis lawtoni	H	H	H	**H**
Platymantis levigatus	H	H	H	**H**
Platymantis luzonensis	H	H	L	**L**
Platymantis macrops	H	L	H	**L**
Platymantis macrosceles	H	L	H	**L**
Platymantis magnus	H	H	H	**H**
Platymantis mamusiorum	H	L	H	**L**
Platymantis mimicus	H	L	H	**L**
Platymantis mimulus	H	H	L	**L**
Platymantis montanus	H	H	L	**L**
Platymantis myersi	H	L	L	**L**
Platymantis nakanaiorum	H	L	H	**L**
Platymantis naomii	H	H	L	**L**
Platymantis neckeri	H	H	H	**H**
Platymantis negrosensis	H	H	L	**L**
Platymantis nexipus	H	L	H	**L**
Platymantis paengi	H	L	H	**L**
Platymantis panayensis	H	H	H	**H**
Platymantis papuensis	L	H	L	**L**
Platymantis parkeri	H	H	H	**H**
Platymantis pelewensis	H	H	H	**H**
Platymantis polillensis	H	H	L	**L**
Platymantis pseudodorsalis	H	H	L	**L**

Platymantis punctatus	H	H	L	**L**
Platymantis pygmaeus	L	H	L	**L**
Platymantis rabori	L	H	L	**L**
Platymantis rhipiphalcus	H	L	H	**L**
Platymantis schmidti	H	H	L	**L**
Platymantis sierramadrensis	L	H	L	**L**
Platymantis solomonis	H	H	H	**H**
Platymantis spelaeus	H	H	L	**L**
Platymantis subterrestris	H	H	H	**H**
Platymantis sulcatus	H	H	H	**H**
Platymantis taylori	H	H	L	**L**
Platymantis vitianus	H	H	L	**L**
Platymantis vitiensis	L	H	L	**L**
Platymantis weberi	H	H	H	**H**
Platymantis wuenscheorum	H	H	H	**H**
Platypelis alticola	H	H	H	**H**
Platypelis barbouri	L	H	H	**L**
Platypelis cowanii	U	L	U	**L**
Platypelis grandis	L	H	H	**L**
Platypelis mavomavo	H	H	H	**H**
Platypelis milloti	H	H	H	**H**
Platypelis pollicaris	U	L	H	**L**
Platypelis tetra	L	H	H	**L**
Platypelis tsaratananaensis	H	H	H	**H**
Platypelis tuberifera	L	H	H	**L**
Platyplectrum ornatum	H	H	L	**L**
Platyplectrum spenceri	H	L	L	**L**
Plectrohyla acanthodes	H	L	H	**L**
Plectrohyla ameibothalame	H	L	L	**L**
Plectrohyla arborescandens	H	L	L	**L**
Plectrohyla avia	H	L	H	**L**
Plectrohyla bistincta	H	L	L	**L**
Plectrohyla calthula	H	L	L	**L**
Plectrohyla calvicollina	H	L	L	**L**
Plectrohyla celata	H	L	H	**L**
Plectrohyla cembra	H	L	H	**L**
Plectrohyla charadricola	H	L	H	**L**
Plectrohyla chryses	H	L	L	**L**
Plectrohyla chrysopleura	H	L	H	**L**
Plectrohyla crassa	H	L	H	**L**
Plectrohyla cyanomma	H	L	H	**L**
Plectrohyla cyclada	H	L	L	**L**
Plectrohyla dasypus	H	L	H	**L**
Plectrohyla ephemera	H	H	L	**L**
Plectrohyla exquisita	H	H	H	**H**

Plectrohyla glandulosa	H	L	H	**L**
Plectrohyla guatemalensis	H	L	H	**L**
Plectrohyla hartwegi	H	L	H	**L**
Plectrohyla hazelae	H	L	L	**L**
Plectrohyla ixil	H	L	H	**L**
Plectrohyla labedactyla	H	L	H	**L**
Plectrohyla lacertosa	H	L	L	**L**
Plectrohyla matudai	H	L	H	**L**
Plectrohyla miahuatlanensis	H	L	H	**L**
Plectrohyla mykter	H	L	L	**L**
Plectrohyla pachyderma	H	L	H	**L**
Plectrohyla pentheter	H	L	L	**L**
Plectrohyla pokomchi	H	L	H	**L**
Plectrohyla psarosema	H	L	H	**L**
Plectrohyla psiloderma	H	L	H	**L**
Plectrohyla pycnochila	H	L	H	**L**
Plectrohyla quecchi	H	L	H	**L**
Plectrohyla robertsorum	H	L	H	**L**
Plectrohyla sabrina	H	L	H	**L**
Plectrohyla sagorum	H	L	H	**L**
Plectrohyla siopela	H	L	H	**L**
Plectrohyla tecunumani	H	L	H	**L**
Plectrohyla teuchestes	H	L	H	**L**
Plectrohyla thorectes	H	L	L	**L**
Plethodon ainsworthi	U	H	U	**L**
Plethodon albagula	H	H	L	**L**
Plethodon amplus	H	H	L	**L**
Plethodon angusticlavius	H	H	L	**L**
Plethodon asupak	H	H	L	**L**
Plethodon aureolus	H	L	H	**L**
Plethodon caddoensis	H	H	L	**L**
Plethodon cheoah	H	H	H	**H**
Plethodon cinereus	H	H	L	**L**
Plethodon cylindraceus	H	H	H	**H**
Plethodon dorsalis	H	H	L	**L**
Plethodon dunni	L	H	L	**L**
Plethodon electromorphus	H	H	H	**H**
Plethodon elongatus	L	H	L	**L**
Plethodon fourchensis	H	H	L	**L**
Plethodon glutinosus	H	H	L	**L**
Plethodon hoffmani	H	H	L	**L**
Plethodon hubrichti	H	H	H	**H**
Plethodon idahoensis	H	H	H	**H**
Plethodon jordani	H	H	H	**H**
Plethodon kentucki	H	H	H	**H**

Plethodon kiamichi	H	L	L	**L**
Plethodon kisatchie	H	H	H	**H**
Plethodon larselli	L	H	L	**L**
Plethodon meridianus	H	H	H	**H**
Plethodon metcalfi	H	H	H	**H**
Plethodon montanus	H	H	L	**L**
Plethodon neomexicanus	H	H	H	**H**
Plethodon nettingi	H	H	L	**L**
Plethodon ouachitae	H	H	L	**L**
Plethodon petraeus	H	H	L	**L**
Plethodon punctatus	H	H	L	**L**
Plethodon richmondi	H	H	L	**L**
Plethodon sequoyah	H	H	L	**L**
Plethodon serratus	H	H	L	**L**
Plethodon shenandoah	H	H	H	**H**
Plethodon sherando	H	H	L	**L**
Plethodon shermani	H	H	H	**H**
Plethodon stormi	H	H	L	**L**
Plethodon teyahalee	H	H	H	**H**
Plethodon vandykei	L	H	H	**L**
Plethodon vehiculum	L	H	L	**L**
Plethodon ventralis	H	H	L	**L**
Plethodon virginia	H	H	L	**L**
Plethodon websteri	H	H	L	**L**
Plethodon wehrlei	H	H	L	**L**
Plethodon welleri	H	H	L	**L**
Plethodon yonahlossee	H	H	L	**L**
Plethodontohyla angulifera	H	L	U	**L**
Plethodontohyla bipunctata	H	H	H	**H**
Plethodontohyla brevipes	L	H	L	**L**
Plethodontohyla fonetana	L	H	L	**L**
Plethodontohyla guentheri	H	L	H	**L**
Plethodontohyla inguinalis	L	H	H	**L**
Plethodontohyla mihanika	L	H	L	**L**
Plethodontohyla notosticta	L	H	L	**L**
Plethodontohyla ocellata	H	H	H	**H**
Plethodontohyla tuberata	L	H	H	**L**
Pleurodeles nebulosus	H	H	U	**L**
Pleurodeles poireti	H	H	H	**H**
Pleurodeles waltl	H	L	L	**L**
Pleurodema bibroni	H	H	L	**L**
Pleurodema borellii	U	L	H	**L**
Pleurodema brachyops	H	H	H	**H**
Pleurodema bufoninum	H	H	L	**L**
Pleurodema cinereum	H	H	H	**H**

Pleurodema diplolister	H	L	L	**L**
Pleurodema fuscomaculatum	U	U	U	**L**
Pleurodema guayapae	H	L	H	**L**
Pleurodema kriegi	H	L	H	**L**
Pleurodema marmoratum	H	L	H	**L**
Pleurodema nebulosum	H	L	H	**L**
Pleurodema thaul	L	L	L	**L**
Pleurodema tucumanum	H	L	H	**L**
Polypedates chlorophthalmus	H	L	H	**L**
Polypedates colletti	L	L	L	**L**
Polypedates cruciger	L	H	L	**L**
Polypedates eques	L	H	H	**L**
Polypedates fastigo	H	H	H	**H**
Polypedates hecticus	H	L	U	**L**
Polypedates insularis	H	H	L	**L**
Polypedates leucomystax	L	L	L	**L**
Polypedates longinasus	L	H	L	**L**
Polypedates macrotis	L	L	L	**L**
Polypedates maculatus	L	L	L	**L**
Polypedates megacephalus	L	L	H	**L**
Polypedates mutus	L	H	H	**L**
Polypedates occidentalis	H	L	H	**L**
Polypedates otilophus	L	L	L	**L**
Polypedates pseudocruciger	L	L	H	**L**
Polypedates taeniatus	L	H	H	**L**
Polypedates zed	U	H	H	**L**
Potomotyphlus kaupii	H	H	L	**L**
Poyntonia paludicola	H	H	H	**H**
Poyntonophrynus beiranus	H	H	L	**L**
Poyntonophrynus damaranus	H	L	U	**L**
Poyntonophrynus dombensis	H	L	L	**L**
Poyntonophrynus fenoulheti	H	H	L	**L**
Poyntonophrynus grandisonae	H	L	L	**L**
Poyntonophrynus hoeschi	H	H	L	**L**
Poyntonophrynus kavangensis	H	H	L	**L**
Poyntonophrynus lughensis	H	H	L	**L**
Poyntonophrynus parkeri	H	L	L	**L**
Poyntonophrynus vertebralis	H	L	H	**L**
Praslinia cooperi	H	H	L	**L**
Pristimantis aaptus	H	H	L	**L**
Pristimantis acatallelus	H	H	H	**H**
Pristimantis acerus	H	H	H	**H**
Pristimantis achatinus	L	H	L	**L**
Pristimantis actinolaimus	H	H	H	**H**
Pristimantis actites	H	H	H	**H**

Pristimantis acuminatus	H	H	H	**H**
Pristimantis acutirostris	H	H	H	**H**
Pristimantis adiastolus	H	L	H	**L**
Pristimantis aemulatus	H	H	L	**L**
Pristimantis affinis	U	H	H	**L**
Pristimantis alalocophus	H	H	H	**H**
Pristimantis albericoi	H	H	H	**H**
Pristimantis albertus	H	H	H	**H**
Pristimantis altae	L	H	H	**L**
Pristimantis altamazonicus	H	H	H	**H**
Pristimantis amydrotus	H	H	H	**H**
Pristimantis anemerus	H	H	H	**H**
Pristimantis angustilineatus	H	H	H	**H**
Pristimantis aniptopalmatus	H	H	H	**H**
Pristimantis anolirex	L	H	H	**L**
Pristimantis anotis	H	L	H	**L**
Pristimantis apiculatus	H	H	H	**H**
Pristimantis appendiculatus	L	H	H	**L**
Pristimantis aquilonaris	H	H	H	**H**
Pristimantis aracamuni	H	H	H	**H**
Pristimantis ardalonychus	H	H	H	**H**
Pristimantis ashkapara	H	H	H	**H**
Pristimantis atrabracus	H	H	L	**L**
Pristimantis atratus	H	H	H	**H**
Pristimantis aurantiguttatus	H	H	H	**H**
Pristimantis aureolineatus	H	H	H	**H**
Pristimantis avicuporum	H	H	L	**L**
Pristimantis avius	H	H	H	**H**
Pristimantis bacchus	H	H	H	**H**
Pristimantis baiotis	H	H	L	**L**
Pristimantis balionotus	H	H	H	**H**
Pristimantis baryecuus	L	H	H	**L**
Pristimantis batrachites	H	H	H	**H**
Pristimantis bearsei	H	L	H	**L**
Pristimantis bellator	H	H	H	**H**
Pristimantis bellona	H	H	H	**H**
Pristimantis bernali	H	H	L	**L**
Pristimantis bicolor	H	H	H	**H**
Pristimantis bicumulus	H	H	H	**H**
Pristimantis bipunctatus	H	H	L	**L**
Pristimantis bisignatus	H	H	H	**H**
Pristimantis boconoensis	H	H	H	**H**
Pristimantis bogotensis	H	H	H	**H**
Pristimantis boulengeri	L	H	H	**L**
Pristimantis brevifrons	L	H	L	**L**

Pristimantis briceni	H	H	H	**H**
Pristimantis bromeliaceus	H	H	H	**H**
Pristimantis buccinator	H	H	H	**H**
Pristimantis buckleyi	L	H	H	**L**
Pristimantis cabrerai	H	H	H	**H**
Pristimantis cacao	H	H	H	**H**
Pristimantis caeruleonotus	H	L	H	**L**
Pristimantis cajamarcensis	H	H	H	**H**
Pristimantis calcaratus	H	H	L	**L**
Pristimantis calcarulatus	H	H	H	**H**
Pristimantis caliginosus	U	L	H	**L**
Pristimantis cantitans	H	H	H	**H**
Pristimantis capitonis	H	H	H	**H**
Pristimantis caprifer	H	H	H	**H**
Pristimantis carlossanchezi	H	L	H	**L**
Pristimantis carmelitae	H	L	H	**L**
Pristimantis carranguerorum	U	L	L	**L**
Pristimantis carvalhoi	H	H	L	**L**
Pristimantis caryophyllaceus	H	H	L	**L**
Pristimantis cavernibardus	H	L	H	**L**
Pristimantis celator	H	H	H	**H**
Pristimantis cerasinus	H	H	H	**H**
Pristimantis ceuthospilus	H	H	H	**H**
Pristimantis chalceus	H	H	H	**H**
Pristimantis charlottevillensis	H	H	H	**H**
Pristimantis chiastonotus	H	H	H	**H**
Pristimantis chimu	H	H	H	**H**
Pristimantis chloronotus	H	H	H	**H**
Pristimantis chrysops	L	H	H	**L**
Pristimantis citriogaster	H	L	H	**L**
Pristimantis colodactylus	H	H	H	**H**
Pristimantis colomai	H	H	H	**H**
Pristimantis colonensis	H	L	H	**L**
Pristimantis colostichos	H	H	H	**H**
Pristimantis condor	H	H	L	**L**
Pristimantis conspicillatus	H	H	H	**H**
Pristimantis cordovae	H	H	H	**H**
Pristimantis corniger	H	L	L	**L**
Pristimantis coronatus	H	H	H	**H**
Pristimantis corrugatus	H	H	H	**H**
Pristimantis cosnipatae	H	H	H	**H**
Pristimantis cremnobates	H	H	H	**H**
Pristimantis crenunguis	H	H	H	**H**
Pristimantis crepitans	U	H	L	**L**
Pristimantis cristinae	U	L	H	**L**

Pristimantis croceoinguinis	H	H	H	**H**
Pristimantis crucifer	H	H	H	**H**
Pristimantis cruciocularis	H	H	H	**H**
Pristimantis cruentus	H	H	H	**H**
Pristimantis cryophilius	H	H	H	**H**
Pristimantis cryptomelas	H	H	H	**H**
Pristimantis cuentasi	H	L	H	**L**
Pristimantis culatensis	U	L	H	**L**
Pristimantis cuneirostris	H	H	L	**L**
Pristimantis curtipes	H	H	H	**H**
Pristimantis danae	L	H	H	**L**
Pristimantis degener	L	H	H	**L**
Pristimantis deinops	H	H	L	**L**
Pristimantis delicatus	H	L	H	**L**
Pristimantis delius	H	H	H	**H**
Pristimantis dendrobatoides	H	H	H	**H**
Pristimantis devillei	L	H	H	**L**
Pristimantis diadematus	H	H	H	**H**
Pristimantis diaphonus	H	H	H	**H**
Pristimantis diogenes	H	H	H	**H**
Pristimantis dissimulatus	H	H	H	**H**
Pristimantis dorsopictus	H	H	H	**H**
Pristimantis douglasi	H	H	H	**H**
Pristimantis duellmani	H	H	H	**H**
Pristimantis duende	H	H	H	**H**
Pristimantis dundeei	U	H	L	**L**
Pristimantis elegans	L	H	H	**L**
Pristimantis epacrus	H	H	H	**H**
Pristimantis eremitus	H	H	H	**H**
Pristimantis eriphus	H	H	H	**H**
Pristimantis ernesti	H	H	L	**L**
Pristimantis erythropleura	H	H	L	**L**
Pristimantis esmeraldas	H	H	H	**H**
Pristimantis eugeniae	H	H	H	**H**
Pristimantis euphronides	H	H	H	**H**
Pristimantis eurydactylus	H	H	L	**L**
Pristimantis exoristus	H	H	H	**H**
Pristimantis factiosus	L	H	L	**L**
Pristimantis fallax	H	H	L	**L**
Pristimantis fenestratus	H	H	L	**L**
Pristimantis fetosus	H	H	H	**H**
Pristimantis flabellidiscus	H	L	H	**L**
Pristimantis flavobracatus	H	H	H	**H**
Pristimantis floridus	L	H	H	**L**
Pristimantis frater	H	H	H	**H**

Pristimantis fraudator	H	H	H	**H**
Pristimantis gaigei	H	H	H	**H**
Pristimantis galdi	H	H	L	**L**
Pristimantis ganonotus	H	H	H	**H**
Pristimantis gentryi	H	H	H	**H**
Pristimantis ginesi	H	H	H	**H**
Pristimantis gladiator	L	H	H	**L**
Pristimantis glandulosus	H	H	H	**H**
Pristimantis gracilis	H	H	H	**H**
Pristimantis grandiceps	H	H	H	**H**
Pristimantis grandoculis	H	L	H	**L**
Pristimantis gutturalis	H	H	H	**H**
Pristimantis hamiotae	L	H	H	**L**
Pristimantis hectus	H	H	H	**H**
Pristimantis helvolus	H	H	H	**H**
Pristimantis hernandezi	H	H	H	**H**
Pristimantis huicundo	H	H	H	**H**
Pristimantis hybotragus	L	H	H	**L**
Pristimantis ignicolor	H	H	H	**H**
Pristimantis illotus	H	H	H	**H**
Pristimantis imitatrix	H	H	H	**H**
Pristimantis incanus	H	H	H	**H**
Pristimantis incertus	H	L	H	**L**
Pristimantis incomptus	H	H	H	**H**
Pristimantis infraguttatus	H	H	L	**L**
Pristimantis inguinalis	H	H	H	**H**
Pristimantis insignitus	L	H	H	**L**
Pristimantis inusitatus	H	H	H	**H**
Pristimantis ixalus	H	H	L	**L**
Pristimantis jabonensis	H	H	H	**H**
Pristimantis jaimei	U	H	H	**L**
Pristimantis jester	H	H	H	**H**
Pristimantis johannesdei	H	H	H	**H**
Pristimantis jorgevelosai	H	H	H	**H**
Pristimantis juanchoi	L	H	H	**L**
Pristimantis jubatus	H	H	H	**H**
Pristimantis karcharias	H	H	L	**L**
Pristimantis kareliae	H	H	H	**H**
Pristimantis katoptroides	H	H	H	**H**
Pristimantis kelephas	H	H	H	**H**
Pristimantis labiosus	H	H	H	**H**
Pristimantis lacrimosus	H	H	H	**H**
Pristimantis lancinii	H	H	H	**H**
Pristimantis lanthanites	H	H	H	**H**
Pristimantis lasalleorum	H	H	H	**H**

Pristimantis laticlavius	H	L	H	**L**
Pristimantis latidiscus	L	H	L	**L**
Pristimantis lemur	H	H	L	**L**
Pristimantis lentiginosus	H	L	H	**L**
Pristimantis leoni	L	H	H	**L**
Pristimantis leptolophus	H	H	H	**H**
Pristimantis leucopus	H	L	H	**L**
Pristimantis librarius	H	H	H	**H**
Pristimantis lichenoides	H	H	H	**H**
Pristimantis lindae	H	L	H	**L**
Pristimantis lirellus	H	H	H	**H**
Pristimantis lividus	H	H	H	**H**
Pristimantis llojsintuta	H	H	H	**H**
Pristimantis loustes	H	H	H	**H**
Pristimantis luscombei	H	H	H	**H**
Pristimantis luteolateralis	L	H	H	**L**
Pristimantis lutitus	H	H	H	**H**
Pristimantis lymani	H	H	H	**H**
Pristimantis lynchi	H	L	H	**L**
Pristimantis lythrodes	H	H	L	**L**
Pristimantis maculosus	H	H	H	**H**
Pristimantis malkini	H	H	L	**L**
Pristimantis marahuaka	H	H	H	**H**
Pristimantis marmoratus	H	H	H	**H**
Pristimantis mars	H	H	L	**L**
Pristimantis martiae	H	H	L	**L**
Pristimantis medemi	L	H	H	**L**
Pristimantis megalops	H	H	H	**H**
Pristimantis melanogaster	H	H	H	**H**
Pristimantis melanoproctus	H	H	H	**H**
Pristimantis memorans	H	L	H	**L**
Pristimantis mendax	L	H	H	**L**
Pristimantis mercedesae	H	H	H	**H**
Pristimantis meridionalis	H	H	H	**H**
Pristimantis merostictus	H	H	H	**H**
Pristimantis metabates	H	H	H	**H**
Pristimantis minutulus	H	L	H	**L**
Pristimantis miyatai	H	H	H	**H**
Pristimantis mnionaetes	H	H	H	**H**
Pristimantis modipeplus	H	H	H	**H**
Pristimantis molybrignus	H	H	H	**H**
Pristimantis mondolfii	H	H	L	**L**
Pristimantis moro	L	H	L	**L**
Pristimantis muricatus	L	H	H	**L**
Pristimantis muscosus	H	L	L	**L**

Pristimantis museosus	H	H	L	**L**
Pristimantis myersi	H	H	H	**H**
Pristimantis myops	H	L	H	**L**
Pristimantis nephophilus	H	H	L	**L**
Pristimantis nervicus	L	H	H	**L**
Pristimantis nicefori	H	H	H	**H**
Pristimantis nigrogriseus	H	H	H	**H**
Pristimantis nyctophylax	H	H	H	**H**
Pristimantis obmutescens	H	H	H	**H**
Pristimantis ocellatus	H	L	H	**L**
Pristimantis ockendeni	H	H	H	**H**
Pristimantis ocreatus	H	H	H	**H**
Pristimantis olivaceus	U	L	H	**L**
Pristimantis orcesi	H	H	H	**H**
Pristimantis orestes	H	H	H	**H**
Pristimantis ornatissimus	L	H	H	**L**
Pristimantis ornatus	H	H	H	**H**
Pristimantis orpacobates	L	H	H	**L**
Pristimantis orphnolaimus	H	L	H	**L**
Pristimantis ortizi	H	L	H	**L**
Pristimantis padrecarlosi	U	L	U	**L**
Pristimantis paisa	L	H	H	**L**
Pristimantis palmeri	L	H	H	**L**
Pristimantis paramerus	H	H	H	**H**
Pristimantis pardalinus	H	H	H	**H**
Pristimantis pardalis	L	H	H	**L**
Pristimantis parectatus	H	H	H	**H**
Pristimantis parvillus	L	H	H	**L**
Pristimantis pastazensis	H	H	H	**H**
Pristimantis pataikos	H	H	L	**L**
Pristimantis paululus	H	H	H	**H**
Pristimantis pecki	H	L	L	**L**
Pristimantis pedimontanus	H	L	H	**L**
Pristimantis penelopus	H	H	L	**L**
Pristimantis peraticus	U	H	H	**L**
Pristimantis percnopterus	H	H	L	**L**
Pristimantis percultus	H	H	H	**H**
Pristimantis permixtus	L	H	H	**L**
Pristimantis peruvianus	H	H	H	**H**
Pristimantis petersorum	H	H	L	**L**
Pristimantis petrobardus	H	L	H	**L**
Pristimantis phalaroinguinis	H	L	H	**L**
Pristimantis phalarus	H	H	H	**H**
Pristimantis philipi	H	L	H	**L**
Pristimantis phoxocephalus	L	H	H	**L**

Pristimantis phragmipleuron	H	H	L	**L**
Pristimantis piceus	L	H	H	**L**
Pristimantis pinguis	H	L	H	**L**
Pristimantis pirrensis	H	H	L	**L**
Pristimantis platychilus	H	H	H	**H**
Pristimantis platydactylus	H	H	H	**H**
Pristimantis pleurostriatus	H	L	H	**L**
Pristimantis pluvicanorus	H	H	H	**H**
Pristimantis polemistes	L	H	H	**L**
Pristimantis polychrus	L	H	H	**L**
Pristimantis prolatus	H	H	L	**L**
Pristimantis prolixodiscus	L	H	H	**L**
Pristimantis proserpens	H	H	H	**H**
Pristimantis pruinatus	H	H	H	**H**
Pristimantis pseudoacuminatus	H	H	H	**H**
Pristimantis pteridophilus	H	H	H	**H**
Pristimantis ptochus	H	L	H	**L**
Pristimantis pugnax	H	H	H	**H**
Pristimantis pulvinatus	L	H	H	**L**
Pristimantis pycnodermis	L	H	H	**L**
Pristimantis pyrrhomerus	H	H	H	**H**
Pristimantis quantus	H	H	H	**H**
Pristimantis quaquaversus	L	H	H	**L**
Pristimantis quinquagesimus	H	H	H	**H**
Pristimantis racemus	H	H	H	**H**
Pristimantis reclusas	H	H	H	**H**
Pristimantis renjiforum	H	H	H	**H**
Pristimantis repens	H	H	H	**H**
Pristimantis restrepoi	L	H	H	**L**
Pristimantis reticulatus	H	L	H	**L**
Pristimantis rhabdocnemus	H	H	L	**L**
Pristimantis rhabdolaemus	H	H	H	**H**
Pristimantis rhigophilus	H	H	H	**H**
Pristimantis rhodoplichus	H	H	H	**H**
Pristimantis rhodostichus	H	H	L	**L**
Pristimantis ridens	L	H	H	**L**
Pristimantis riveroi	H	L	H	**L**
Pristimantis riveti	H	H	H	**H**
Pristimantis rosadoi	H	H	H	**H**
Pristimantis roseus	H	H	H	**H**
Pristimantis rozei	H	H	H	**H**
Pristimantis rubicundus	H	H	L	**L**
Pristimantis ruedai	H	H	H	**H**
Pristimantis rufioculis	H	L	H	**L**
Pristimantis ruidus	H	L	H	**L**

Pristimantis ruthveni	H	H	H	**H**
Pristimantis salaputium	H	L	H	**L**
Pristimantis saltissimus	H	H	H	**H**
Pristimantis samaipatae	H	H	H	**H**
Pristimantis sanctaemartae	H	H	H	**H**
Pristimantis sanguineus	H	H	H	**H**
Pristimantis satagius	H	H	H	**H**
Pristimantis savagei	H	H	H	**H**
Pristimantis schultei	H	H	L	**L**
Pristimantis scitulus	H	L	H	**L**
Pristimantis scoloblepharus	H	H	H	**H**
Pristimantis scolodiscus	H	H	H	**H**
Pristimantis scopaeus	U	L	H	**L**
Pristimantis seorsus	H	L	H	**L**
Pristimantis serendipitus	H	H	L	**L**
Pristimantis shrevei	H	H	H	**H**
Pristimantis signifer	H	H	H	**H**
Pristimantis silverstonei	H	H	H	**H**
Pristimantis simonbolivari	H	H	H	**H**
Pristimantis simonsii	H	H	H	**H**
Pristimantis simoteriscus	H	H	H	**H**
Pristimantis simoterus	H	H	H	**H**
Pristimantis siopelus	H	H	H	**H**
Pristimantis skydmainos	H	H	H	**H**
Pristimantis sobetes	H	H	H	**H**
Pristimantis spilogaster	H	H	H	**H**
Pristimantis spinosus	H	H	L	**L**
Pristimantis stenodiscus	H	L	H	**L**
Pristimantis sternothylax	H	H	H	**H**
Pristimantis stictoboubonus	H	L	L	**L**
Pristimantis stictogaster	H	L	L	**L**
Pristimantis subsigillatus	H	H	H	**H**
Pristimantis suetus	H	H	H	**H**
Pristimantis sulculus	H	H	H	**H**
Pristimantis supernatis	H	H	H	**H**
Pristimantis surdus	H	H	H	**H**
Pristimantis susaguae	H	L	H	**L**
Pristimantis taciturnus	H	L	H	**L**
Pristimantis taeniatus	L	H	H	**L**
Pristimantis tamsitti	H	H	L	**L**
Pristimantis tantanti	H	L	H	**L**
Pristimantis tanyrhynchus	H	L	H	**L**
Pristimantis tayrona	H	H	H	**H**
Pristimantis telefericus	H	L	H	**L**
Pristimantis tenebrionis	H	H	H	**H**

Pristimantis terraebolivaris	L	H	H	**L**
Pristimantis thectopternus	L	H	H	**L**
Pristimantis thyellus	H	H	H	**H**
Pristimantis thymalopsoides	H	H	H	**H**
Pristimantis thymelensis	H	H	H	**H**
Pristimantis toftae	H	H	H	**H**
Pristimantis torrenticola	H	H	H	**H**
Pristimantis trachyblepharis	U	L	H	**L**
Pristimantis trepidotus	U	H	U	**L**
Pristimantis tribulosus	H	H	H	**H**
Pristimantis truebae	H	H	H	**H**
Pristimantis tubernasus	H	L	H	**L**
Pristimantis turpinorum	H	H	H	**H**
Pristimantis turumiquirensis	L	H	H	**L**
Pristimantis uisae	H	L	H	**L**
Pristimantis unistrigatus	H	H	H	**H**
Pristimantis uranobates	H	H	H	**H**
Pristimantis urichi	H	H	H	**H**
Pristimantis vanadise	H	H	H	**H**
Pristimantis variabilis	H	H	L	**L**
Pristimantis veletis	L	H	H	**L**
Pristimantis ventriguttatus	H	H	H	**H**
Pristimantis ventrimarmoratus	L	H	H	**L**
Pristimantis verecundus	H	H	H	**H**
Pristimantis versicolor	L	H	H	**L**
Pristimantis vertebralis	L	H	H	**L**
Pristimantis vicarius	H	H	H	**H**
Pristimantis vidua	H	H	H	**H**
Pristimantis viejas	L	H	L	**L**
Pristimantis vilarsi	H	H	H	**H**
Pristimantis vilcabambae	H	H	H	**H**
Pristimantis viridicans	H	H	H	**H**
Pristimantis viridis	H	H	H	**H**
Pristimantis wagteri	H	H	L	**L**
Pristimantis walkeri	L	H	H	**L**
Pristimantis waoranii	H	H	H	**H**
Pristimantis wiensi	H	L	H	**L**
Pristimantis w-nigrum	L	H	H	**L**
Pristimantis xeniolum	H	L	H	**L**
Pristimantis xestus	H	H	H	**H**
Pristimantis xylochobates	H	H	H	**H**
Pristimantis yaviensis	H	L	H	**L**
Pristimantis yustizi	H	H	H	**H**
Pristimantis zeuctotylus	H	H	H	**H**
Pristimantis zimmermanae	H	H	H	**H**

Pristimantis zoilae	H	H	H	**H**
Pristimantis zophus	H	H	H	**H**
Probreviceps durirostris	H	H	L	**L**
Probreviceps loveridgei	H	H	H	**H**
Probreviceps macrodactylus	H	H	L	**L**
Probreviceps rhodesianus	H	H	L	**L**
Probreviceps rungwensis	L	H	L	**L**
Probreviceps uluguruensis	H	H	H	**H**
Proceratophrys appendiculata	L	L	L	**L**
Proceratophrys avelinoi	H	L	L	**L**
Proceratophrys bigibbosa	H	L	L	**L**
Proceratophrys boiei	L	L	L	**L**
Proceratophrys brauni	H	L	L	**L**
Proceratophrys concavitympanum	H	L	H	**L**
Proceratophrys cristiceps	H	L	L	**L**
Proceratophrys cururu	H	L	L	**L**
Proceratophrys fryi	L	L	L	**L**
Proceratophrys goyana	H	L	L	**L**
Proceratophrys laticeps	H	L	H	**L**
Proceratophrys melanopogon	L	L	L	**L**
Proceratophrys moehringi	U	L	L	**L**
Proceratophrys palustris	U	L	L	**L**
Proceratophrys paviotii	U	L	L	**L**
Proceratophrys phyllostoma	U	L	L	**L**
Proceratophrys schirchi	H	L	H	**L**
Proceratophrys subguttata	H	L	L	**L**
Prostherapis dunni	H	H	H	**H**
Proteus anguinus	H	H	H	**H**
Protohynobius puxiongensis	U	L	L	**L**
Pseudacris brachyphona	H	L	L	**L**
Pseudacris brimleyi	H	L	L	**L**
Pseudacris cadaverina	H	L	L	**L**
Pseudacris clarkii	H	H	L	**L**
Pseudacris crucifer	H	H	L	**L**
Pseudacris feriarum	H	H	L	**L**
Pseudacris nigrita	H	L	L	**L**
Pseudacris ocularis	H	H	L	**L**
Pseudacris ornata	H	H	L	**L**
Pseudacris regilla	H	L	H	**L**
Pseudacris streckeri	H	L	L	**L**
Pseudacris triseriata	H	L	L	**L**
Pseudepidalea brongersmai	H	H	H	**H**
Pseudepidalea latastii	H	L	H	**L**
Pseudepidalea luristanica	H	H	L	**L**
Pseudepidalea oblonga	H	H	H	**H**

Pseudepidalea pewzowi	H	L	H	**L**
Pseudepidalea pseudoraddei	H	H	H	**H**
Pseudepidalea raddei	H	H	H	**H**
Pseudepidalea surda	H	L	H	**L**
Pseudepidalea taxkorensis	H	L	H	**L**
Pseudepidalea viridis	H	L	L	**L**
Pseudepidalea zamdaensis	H	H	H	**H**
Pseudhymenochirus merlini	L	L	H	**L**
Pseudis bolbodactyla	H	H	L	**L**
Pseudis boliviana	H	L	H	**L**
Pseudis caraya	H	L	L	**L**
Pseudis cardosoi	H	L	H	**L**
Pseudis fusca	H	L	L	**L**
Pseudis laevis	H	L	H	**L**
Pseudis limellum	H	L	L	**L**
Pseudis minuta	H	L	L	**L**
Pseudis paradoxa	L	L	L	**L**
Pseudis platensis	H	L	L	**L**
Pseudis tocantins	H	H	L	**L**
Pseudobranchus axanthus	H	L	L	**L**
Pseudobranchus striatus	H	L	L	**L**
Pseudobufo subasper	H	H	H	**H**
Pseudoeurycea ahuitzotl	H	H	L	**L**
Pseudoeurycea altamontana	H	H	L	**L**
Pseudoeurycea amuzga	H	L	L	**L**
Pseudoeurycea anitae	H	H	H	**H**
Pseudoeurycea aquatica	L	H	H	**L**
Pseudoeurycea aurantia	H	H	L	**L**
Pseudoeurycea bellii	H	H	L	**L**
Pseudoeurycea boneti	H	H	H	**H**
Pseudoeurycea brunnata	H	H	H	**H**
Pseudoeurycea cephalica	L	H	L	**L**
Pseudoeurycea cochranae	L	H	H	**L**
Pseudoeurycea conanti	L	H	H	**L**
Pseudoeurycea exspectata	H	H	H	**H**
Pseudoeurycea firscheini	H	H	L	**L**
Pseudoeurycea gadovii	L	H	L	**L**
Pseudoeurycea galeanae	H	H	H	**H**
Pseudoeurycea gigantea	H	H	H	**H**
Pseudoeurycea goebeli	H	H	H	**H**
Pseudoeurycea juarezi	H	H	H	**H**
Pseudoeurycea leprosa	H	H	L	**L**
Pseudoeurycea lineola	L	H	H	**L**
Pseudoeurycea longicauda	H	H	L	**L**
Pseudoeurycea lynchi	L	H	H	**L**

Pseudoeurycea maxima	U	L	L	**L**
Pseudoeurycea melanomolga	L	H	L	**L**
Pseudoeurycea mixcoatl	H	L	L	**L**
Pseudoeurycea mixteca	H	H	H	**H**
Pseudoeurycea mystax	H	H	H	**H**
Pseudoeurycea naucampatepetl	H	H	H	**H**
Pseudoeurycea nigra	L	H	L	**L**
Pseudoeurycea nigromaculata	H	H	H	**H**
Pseudoeurycea obesa	U	L	L	**L**
Pseudoeurycea orchileucos	H	H	H	**H**
Pseudoeurycea orchimelas	H	H	H	**H**
Pseudoeurycea papenfussi	L	H	L	**L**
Pseudoeurycea parva	H	H	L	**L**
Pseudoeurycea praecellens	H	H	H	**H**
Pseudoeurycea quetzalanensis	H	H	H	**H**
Pseudoeurycea rex	H	H	H	**H**
Pseudoeurycea robertsi	H	H	H	**H**
Pseudoeurycea ruficauda	H	L	L	**L**
Pseudoeurycea saltator	H	H	L	**L**
Pseudoeurycea scandens	H	H	H	**H**
Pseudoeurycea smithi	H	H	H	**H**
Pseudoeurycea tenchalli	H	H	L	**L**
Pseudoeurycea teotepec	H	H	H	**H**
Pseudoeurycea tlahcuiloh	H	H	L	**L**
Pseudoeurycea tlilicxitl	H	H	L	**L**
Pseudoeurycea unguidentis	H	H	H	**H**
Pseudoeurycea werleri	L	H	H	**L**
Pseudohynobius flavomaculatus	L	H	L	**L**
Pseudohynobius kuankuoshuiensis	L	L	L	**L**
Pseudohynobius shuichengensis	L	L	L	**L**
Pseudopaludicola boliviana	L	L	L	**L**
Pseudopaludicola canga	H	L	L	**L**
Pseudopaludicola ceratophryes	H	H	L	**L**
Pseudopaludicola falcipes	H	L	L	**L**
Pseudopaludicola llanera	H	L	H	**L**
Pseudopaludicola mineira	H	L	L	**L**
Pseudopaludicola mirandae	H	L	H	**L**
Pseudopaludicola mystacalis	H	L	L	**L**
Pseudopaludicola pusilla	U	L	H	**L**
Pseudopaludicola riopiedadensis	U	H	U	**L**
Pseudopaludicola saltica	H	L	L	**L**
Pseudopaludicola ternetzi	H	L	L	**L**
Pseudophryne australis	H	H	L	**L**
Pseudophryne bibronii	H	H	L	**L**
Pseudophryne coriacea	H	H	L	**L**

Pseudophryne corroboree	H	H	H	**H**
Pseudophryne covacevichae	H	H	L	**L**
Pseudophryne dendyi	H	L	H	**L**
Pseudophryne douglasi	H	H	L	**L**
Pseudophryne guentheri	H	H	H	**H**
Pseudophryne major	H	L	L	**L**
Pseudophryne occidentalis	H	H	L	**L**
Pseudophryne pengilleyi	H	H	H	**H**
Pseudophryne raveni	H	L	L	**L**
Pseudophryne semimarmorata	H	H	L	**L**
Pseudorana sangzhiensis	U	L	L	**L**
Pseudorana weiningensis	H	L	L	**L**
Pseudotriton montanus	H	H	L	**L**
Pseudotriton ruber	H	H	L	**L**
Psychrophrynella adenopleura	L	H	H	**L**
Psychrophrynella ankohuma	H	H	H	**H**
Psychrophrynella bagrecitoi	H	H	H	**H**
Psychrophrynella boettgeri	H	H	H	**H**
Psychrophrynella chacaltaya	H	H	H	**H**
Psychrophrynella condoriri	H	H	H	**H**
Psychrophrynella guillei	H	H	H	**H**
Psychrophrynella harveyi	H	H	H	**H**
Psychrophrynella iani	H	H	H	**H**
Psychrophrynella iatamasi	L	H	H	**L**
Psychrophrynella illampu	H	H	H	**H**
Psychrophrynella illimani	H	H	H	**H**
Psychrophrynella kallawaya	H	H	H	**H**
Psychrophrynella katantika	H	H	H	**H**
Psychrophrynella kempffi	H	H	H	**H**
Psychrophrynella pinguis	L	H	H	**L**
Psychrophrynella quimsacruzis	H	H	H	**H**
Psychrophrynella saltator	H	H	H	**H**
Psychrophrynella wettsteini	H	H	H	**H**
Pterorana khare	L	L	H	**L**
Ptychadena aequiplicata	L	L	L	**L**
Ptychadena anchietae	H	L	L	**L**
Ptychadena ansorgii	L	L	L	**L**
Ptychadena arnei	H	L	L	**L**
Ptychadena bibroni	H	L	L	**L**
Ptychadena broadleyi	H	H	L	**L**
Ptychadena bunoderma	H	L	L	**L**
Ptychadena christyi	H	L	L	**L**
Ptychadena chrysogaster	H	L	L	**L**
Ptychadena cooperi	H	L	L	**L**
Ptychadena erlangeri	H	L	L	**L**

Ptychadena filwoha	H	L	H	**L**
Ptychadena gansi	H	L	L	**L**
Ptychadena grandisonae	H	L	L	**L**
Ptychadena guibei	H	L	L	**L**
Ptychadena harenna	H	L	H	**L**
Ptychadena ingeri	H	L	H	**L**
Ptychadena keilingi	H	H	L	**L**
Ptychadena longirostris	H	H	L	**L**
Ptychadena mahnerti	H	H	H	**H**
Ptychadena mapacha	H	L	L	**L**
Ptychadena mascareniensis	L	L	L	**L**
Ptychadena mossambica	H	H	L	**L**
Ptychadena nana	H	L	L	**L**
Ptychadena neumanni	L	L	L	**L**
Ptychadena newtoni	H	H	L	**L**
Ptychadena obscura	H	L	L	**L**
Ptychadena oxyrhynchus	H	L	L	**L**
Ptychadena perplicata	U	L	U	**L**
Ptychadena perreti	H	L	L	**L**
Ptychadena porosissima	L	L	L	**L**
Ptychadena pujoli	H	L	L	**L**
Ptychadena pumilio	H	L	L	**L**
Ptychadena retropunctata	H	H	L	**L**
Ptychadena schillukorum	H	L	L	**L**
Ptychadena stenocephala	H	H	L	**L**
Ptychadena straeleni	H	L	L	**L**
Ptychadena submascareniensis	H	H	L	**L**
Ptychadena subpunctata	H	L	L	**L**
Ptychadena superciliaris	H	L	L	**L**
Ptychadena taenioscelis	H	H	L	**L**
Ptychadena tellinii	H	L	L	**L**
Ptychadena tournieri	H	L	L	**L**
Ptychadena trinodis	H	H	L	**L**
Ptychadena upembae	H	L	L	**L**
Ptychadena uzungwensis	H	L	L	**L**
Ptychadena wadei	H	L	L	**L**
Ptychohyla acrochorda	H	L	H	**L**
Ptychohyla dendrophasma	H	H	H	**H**
Ptychohyla erythromma	H	L	L	**L**
Ptychohyla euthysanota	H	L	H	**L**
Ptychohyla hypomykter	H	L	H	**L**
Ptychohyla legleri	H	L	H	**L**
Ptychohyla leonhardschultzei	H	L	L	**L**
Ptychohyla macrotympanum	H	L	H	**L**
Ptychohyla panchoi	H	L	H	**L**

Ptychohyla salvadorensis	H	L	H	**L**
Ptychohyla sanctaecrucis	H	L	H	**L**
Ptychohyla spinipollex	H	L	H	**L**
Ptychohyla zophodes	H	L	H	**L**
Pyxicephalus adspersus	H	L	L	**L**
Pyxicephalus cordofanus	H	L	U	**L**
Pyxicephalus edulis	H	H	L	**L**
Pyxicephalus obbianus	H	L	L	**L**
Quasipaa boulengeri	H	L	H	**L**
Quasipaa exilispinosa	H	L	L	**L**
Quasipaa fasciculispina	L	L	H	**L**
Quasipaa jiulongensis	L	L	L	**L**
Quasipaa robertingeri	L	L	H	**L**
Quasipaa shini	H	L	L	**L**
Quasipaa spinosa	L	L	L	**L**
Quasipaa verrucospinosa	L	L	H	**L**
Ramanella anamalaiensis	H	H	L	**L**
Ramanella minor	H	L	L	**L**
Ramanella montana	L	H	H	**L**
Ramanella mormorata	H	L	H	**L**
Ramanella nagaoi	H	H	H	**H**
Ramanella obscura	L	H	H	**L**
Ramanella palmata	H	H	H	**H**
Ramanella triangularis	L	L	L	**L**
Ramanella variegata	L	H	L	**L**
Rana amurensis	H	L	H	**L**
Rana arvalis	H	L	H	**L**
Rana asiatica	H	L	H	**L**
Rana aurora	H	L	L	**L**
Rana boylii	L	L	L	**L**
Rana cascadae	H	L	L	**L**
Rana chaochiaoensis	L	L	L	**L**
Rana chensinensis	H	L	H	**L**
Rana chevronta	L	L	H	**L**
Rana coreana	L	L	H	**L**
Rana dalmatina	H	H	L	**L**
Rana dybowskii	H	L	H	**L**
Rana graeca	H	L	L	**L**
Rana hanluica	L	L	L	**L**
Rana holtzi	H	H	H	**H**
Rana huanrensis	L	L	H	**L**
Rana iberica	H	H	L	**L**
Rana italica	H	H	L	**L**
Rana japonica	H	L	L	**L**
Rana johnsi	H	L	L	**L**

Rana kukunoris	H	L	H	**L**
Rana kunyuensis	H	L	H	**L**
Rana latastei	H	H	H	**H**
Rana longicrus	H	H	H	**H**
Rana luteiventris	H	L	H	**L**
Rana macrocnemis	H	L	L	**L**
Rana multidenticulata	L	H	H	**L**
Rana muscosa	H	L	L	**L**
Rana omeimontis	H	L	H	**L**
Rana ornativentris	H	L	L	**L**
Rana pirica	H	H	H	**H**
Rana pretiosa	H	L	L	**L**
Rana pseudodalmatina	H	L	H	**L**
Rana pyrenaica	H	L	H	**L**
Rana sakuraii	L	L	L	**L**
Rana sauteri	H	H	H	**H**
Rana shuchinae	H	L	L	**L**
Rana sierrae	L	L	L	**L**
Rana tagoi	H	H	L	**L**
Rana tavasensis	H	L	H	**L**
Rana temporaria	H	L	H	**L**
Rana tsushimensis	L	H	H	**L**
Rana zhengi	U	L	H	**L**
Rana zhenhaiensis	H	H	L	**L**
Ranitomeya abdita	H	H	L	**L**
Ranitomeya altobueyensis	H	H	L	**L**
Ranitomeya amazonica	H	H	H	**H**
Ranitomeya biolat	H	H	H	**H**
Ranitomeya bombetes	L	H	H	**L**
Ranitomeya claudiae	H	L	L	**L**
Ranitomeya daleswansoni	H	L	H	**L**
Ranitomeya dorisswansonae	H	H	H	**H**
Ranitomeya duellmani	H	H	H	**H**
Ranitomeya fantastica	H	H	H	**H**
Ranitomeya flavovittata	H	L	U	**L**
Ranitomeya fulgurita	H	H	L	**L**
Ranitomeya ignea	H	L	H	**L**
Ranitomeya imitator	H	L	H	**L**
Ranitomeya intermedia	H	L	H	**L**
Ranitomeya lamasi	L	H	H	**L**
Ranitomeya minuta	H	H	L	**L**
Ranitomeya opisthomelas	H	H	L	**L**
Ranitomeya reticulata	H	H	H	**H**
Ranitomeya rubrocephala	U	L	U	**L**
Ranitomeya sirensis	H	H	H	**H**

Ranitomeya tolimensis	H	H	L	**L**
Ranitomeya uakarii	H	H	H	**H**
Ranitomeya vanzolinii	H	H	L	**L**
Ranitomeya variabilis	H	L	H	**L**
Ranitomeya ventrimaculata	H	H	H	**H**
Ranitomeya viridis	L	H	H	**L**
Ranitomeya virolinensis	L	H	L	**L**
Ranodon sibiricus	H	H	H	**H**
Relictivomer pearsei	L	H	H	**L**
Rhacophorus achantharrhena	H	L	H	**L**
Rhacophorus angulirostris	H	L	H	**L**
Rhacophorus annamensis	L	L	L	**L**
Rhacophorus appendiculatus	L	L	L	**L**
Rhacophorus arboreus	L	H	L	**L**
Rhacophorus arvalis	H	H	H	**H**
Rhacophorus aurantiventris	H	H	L	**L**
Rhacophorus baluensis	H	H	H	**H**
Rhacophorus barisani	H	L	H	**L**
Rhacophorus bifasciatus	L	L	L	**L**
Rhacophorus bimaculatus	L	L	L	**L**
Rhacophorus bipunctatus	L	L	L	**L**
Rhacophorus calcadensis	L	L	H	**L**
Rhacophorus calcaneus	L	L	L	**L**
Rhacophorus catamitus	H	L	H	**L**
Rhacophorus chenfui	L	L	H	**L**
Rhacophorus cyanopunctatus	L	L	L	**L**
Rhacophorus dennysi	L	L	L	**L**
Rhacophorus depressus	H	U	U	**L**
Rhacophorus dorsoviridis	U	H	H	**L**
Rhacophorus duboisi	U	H	H	**L**
Rhacophorus dugritei	L	H	L	**L**
Rhacophorus dulitensis	H	H	H	**H**
Rhacophorus edentulus	H	L	U	**L**
Rhacophorus everetti	L	H	H	**L**
Rhacophorus exechopygus	L	L	L	**L**
Rhacophorus fasciatus	L	H	H	**L**
Rhacophorus feae	L	L	L	**L**
Rhacophorus gadingensis	L	L	H	**L**
Rhacophorus gauni	L	L	H	**L**
Rhacophorus georgii	H	L	L	**L**
Rhacophorus gongshanensis	H	L	L	**L**
Rhacophorus harrissoni	H	H	H	**H**
Rhacophorus hoanglienensis	H	H	H	**H**
Rhacophorus hui	L	L	L	**L**
Rhacophorus hungfuensis	U	L	H	**L**

Rhacophorus jarujini	L	L	H	**L**
Rhacophorus kajau	L	L	H	**L**
Rhacophorus kio	L	L	L	**L**
Rhacophorus lateralis	H	H	H	**H**
Rhacophorus malabaricus	L	L	H	**L**
Rhacophorus margaritifer	L	H	H	**L**
Rhacophorus maximus	L	H	H	**L**
Rhacophorus minimus	L	L	H	**L**
Rhacophorus modestus	H	L	H	**L**
Rhacophorus moltrechti	L	H	H	**L**
Rhacophorus monticola	L	H	H	**L**
Rhacophorus nigropalmatus	H	H	L	**L**
Rhacophorus nigropunctatus	L	L	L	**L**
Rhacophorus notater	H	L	L	**L**
Rhacophorus omeimontis	L	L	H	**L**
Rhacophorus orlovi	U	L	H	**L**
Rhacophorus owstoni	H	H	H	**H**
Rhacophorus pardalis	L	L	L	**L**
Rhacophorus poecilonotus	U	L	H	**L**
Rhacophorus prasinatus	L	H	H	**L**
Rhacophorus prominanus	L	L	L	**L**
Rhacophorus pseudomalabaricus	L	H	H	**L**
Rhacophorus puerensis	U	H	H	**L**
Rhacophorus reinwardtii	L	H	L	**L**
Rhacophorus rhodopus	L	L	H	**L**
Rhacophorus rhyssocephalus	H	L	H	**L**
Rhacophorus robinsonii	H	H	L	**L**
Rhacophorus rufipes	H	L	L	**L**
Rhacophorus schlegelii	L	H	L	**L**
Rhacophorus suffry	H	L	H	**L**
Rhacophorus taipeianus	L	H	H	**L**
Rhacophorus taronensis	H	L	H	**L**
Rhacophorus translineatus	U	L	H	**L**
Rhacophorus tuberculatus	U	H	H	**L**
Rhacophorus turpes	H	L	H	**L**
Rhacophorus variabilis	U	U	U	**L**
Rhacophorus verrucopus	U	H	H	**L**
Rhacophorus viridis	L	H	H	**L**
Rhacophorus yaoshanensis	L	H	L	**L**
Rhacophorus yinggelingensis	L	H	L	**L**
Rhacophorus zhaojuensis	L	L	L	**L**
Rhaebo anderssoni	H	L	H	**L**
Rhaebo blombergi	H	L	H	**L**
Rhaebo caeruleostictus	H	L	H	**L**
Rhaebo glaberrimus	H	L	H	**L**

Rhaebo guttatus	H	L	H	**L**
Rhaebo haematiticus	H	L	H	**L**
Rhaebo hypomelas	H	L	H	**L**
Rhaebo lynchi	H	L	H	**L**
Rhaebo nasicus	L	L	H	**L**
Rheobates palmatus	L	H	H	**L**
Rheobates pseudopalmatus	H	L	H	**L**
Rheobatrachus silus	H	H	U	**L**
Rheobatrachus vitellinus	H	H	U	**L**
Rhinatrema bivittatum	H	H	H	**H**
Rhinella abei	H	L	L	**L**
Rhinella achalensis	H	L	H	**L**
Rhinella achavali	H	L	H	**L**
Rhinella acrolopha	U	L	L	**L**
Rhinella acutirostris	H	H	L	**L**
Rhinella alata	H	L	H	**L**
Rhinella amabilis	H	L	H	**L**
Rhinella amboroensis	U	L	H	**L**
Rhinella arborescandens	H	L	L	**L**
Rhinella arenarum	H	L	L	**L**
Rhinella arunco	H	L	L	**L**
Rhinella atacamensis	H	L	H	**L**
Rhinella beebei	H	H	H	**H**
Rhinella bergi	H	L	L	**L**
Rhinella boulengeri	H	L	H	**L**
Rhinella castaneotica	H	L	L	**L**
Rhinella ceratophrys	H	L	L	**L**
Rhinella cerradensis	H	H	L	**L**
Rhinella chavin	H	L	H	**L**
Rhinella chrysophora	H	L	H	**L**
Rhinella cristinae	H	L	L	**L**
Rhinella crucifer	H	L	L	**L**
Rhinella dapsilis	H	L	H	**L**
Rhinella diptycha	H	H	L	**L**
Rhinella dorbignyi	H	H	H	**H**
Rhinella fernandezae	H	L	L	**L**
Rhinella festae	L	H	H	**L**
Rhinella fissipes	H	L	H	**L**
Rhinella gallardoi	L	L	H	**L**
Rhinella gnustae	H	L	H	**L**
Rhinella granulosa	H	L	L	**L**
Rhinella henseli	H	L	L	**L**
Rhinella hoogmoedi	H	H	L	**L**
Rhinella icterica	H	L	L	**L**
Rhinella inca	L	L	H	**L**

Rhinella iserni	H	L	H	**L**
Rhinella jimi	H	H	L	**L**
Rhinella justinianoi	L	L	H	**L**
Rhinella lescurei	H	L	H	**L**
Rhinella limensis	H	L	H	**L**
Rhinella lindae	H	L	H	**L**
Rhinella macrorhina	H	H	L	**L**
Rhinella magnussoni	H	H	H	**H**
Rhinella manu	H	L	H	**L**
Rhinella margaritifera	H	L	L	**L**
Rhinella marina	H	L	L	**L**
Rhinella martyi	H	H	H	**H**
Rhinella multiverrucosa	H	L	H	**L**
Rhinella nesiotes	H	L	H	**L**
Rhinella nicefori	H	H	H	**H**
Rhinella ocellata	H	L	L	**L**
Rhinella ornata	H	L	L	**L**
Rhinella poeppigii	H	L	H	**L**
Rhinella pombali	H	L	L	**L**
Rhinella proboscidea	H	L	L	**L**
Rhinella pygmaea	H	L	L	**L**
Rhinella quechua	L	L	H	**L**
Rhinella roqueana	H	L	H	**L**
Rhinella rostrata	H	H	H	**H**
Rhinella rubescens	H	L	L	**L**
Rhinella rubropunctata	H	L	L	**L**
Rhinella ruizi	H	H	H	**H**
Rhinella rumbolli	L	L	H	**L**
Rhinella schneideri	H	L	L	**L**
Rhinella scitula	H	L	L	**L**
Rhinella sclerocephala	H	H	H	**H**
Rhinella sima	H	U	U	**L**
Rhinella spinulosa	H	L	L	**L**
Rhinella stanlaii	H	L	H	**L**
Rhinella sternosignata	H	L	L	**L**
Rhinella tacana	H	H	H	**H**
Rhinella tenrec	H	L	L	**L**
Rhinella truebae	H	L	U	**L**
Rhinella vellardi	H	L	H	**L**
Rhinella veraguensis	H	L	H	**L**
Rhinella veredas	H	H	L	**L**
Rhinella yanachaga	H	H	L	**L**
Rhinoderma darwinii	H	H	L	**L**
Rhinoderma rufum	H	H	L	**L**
Rhinophrynus dorsalis	L	L	H	**L**

Rhombophryne alluaudi	L	H	H	**L**
Rhombophryne coronata	L	H	L	**L**
Rhombophryne coudreaui	H	H	H	**H**
Rhombophryne guentherpetersi	H	H	H	**H**
Rhombophryne laevipes	H	H	H	**H**
Rhombophryne minuta	U	H	H	**L**
Rhombophryne serratopalpebrosa	L	H	H	**L**
Rhombophryne testudo	L	H	H	**L**
Rhyacotriton cascadae	L	H	L	**L**
Rhyacotriton kezeri	L	H	L	**L**
Rhyacotriton olympicus	L	H	H	**L**
Rhyacotriton variegatus	L	H	L	**L**
Rupirana cardosoi	H	L	L	**L**
Sabahphrynus anotis	H	H	H	**H**
Sabahphrynus maculatus	H	U	H	**L**
Salamandra algira	H	H	H	**H**
Salamandra atra	H	H	H	**H**
Salamandra corsica	H	H	L	**L**
Salamandra infraimmaculata	H	H	L	**L**
Salamandra lanzai	H	H	L	**L**
Salamandra salamandra	H	H	L	**L**
Salamandrella keyserlingii	H	H	H	**H**
Salamandrina perspicillata	H	H	L	**L**
Salamandrina terdigitata	H	H	L	**L**
Sanguirana sanguinea	H	H	H	**H**
Scaphiophryne boribory	L	L	H	**L**
Scaphiophryne brevis	L	H	L	**L**
Scaphiophryne calcarata	L	L	L	**L**
Scaphiophryne gottlebei	L	L	L	**L**
Scaphiophryne madagascariensis	L	H	L	**L**
Scaphiophryne marmorata	L	H	H	**L**
Scaphiophryne menabensis	L	L	L	**L**
Scaphiophryne obscura	H	L	U	**L**
Scaphiophryne spinosa	L	L	H	**L**
Scaphiophryne verrucosa	H	L	L	**L**
Scaphiopus couchii	H	H	H	**H**
Scaphiopus holbrookii	H	L	L	**L**
Scaphiopus hurterii	H	L	L	**L**
Scarthyla goinorum	H	L	L	**L**
Scarthyla vigilans	H	L	H	**L**
Schismaderma carens	H	L	L	**L**
Schistometopum gregorii	L	H	L	**L**
Schistometopum thomense	L	H	L	**L**
Scinax acuminatus	H	L	L	**L**
Scinax agilis	H	L	H	**L**

Scinax albicans	H	L	H	**L**
Scinax alcatraz	H	H	U	**L**
Scinax altae	H	H	L	**L**
Scinax alter	H	L	H	**L**
Scinax angrensis	H	L	L	**L**
Scinax arduous	H	L	L	**L**
Scinax argyreornatus	H	L	L	**L**
Scinax ariadne	H	L	L	**L**
Scinax aromothyella	H	L	L	**L**
Scinax atratus	H	L	L	**L**
Scinax auratus	H	L	L	**L**
Scinax baumgardneri	H	L	H	**L**
Scinax berthae	H	L	L	**L**
Scinax blairi	H	L	H	**L**
Scinax boesemani	H	L	H	**L**
Scinax boulengeri	H	L	H	**L**
Scinax brieni	H	L	L	**L**
Scinax cabralensis	H	L	L	**L**
Scinax caldarum	H	H	L	**L**
Scinax camposseabrai	H	U	U	**L**
Scinax canastrensis	H	L	L	**L**
Scinax cardosoi	H	L	L	**L**
Scinax carnevallii	H	H	L	**L**
Scinax castroviejoi	H	L	H	**L**
Scinax catharinae	H	L	L	**L**
Scinax centralis	H	L	L	**L**
Scinax chiquitanus	H	L	L	**L**
Scinax constrictus	H	L	H	**L**
Scinax crospedospilus	H	H	L	**L**
Scinax cruentommus	H	H	L	**L**
Scinax curicica	H	L	H	**L**
Scinax cuspidatus	H	H	H	**H**
Scinax danae	U	L	H	**L**
Scinax duartei	H	L	L	**L**
Scinax elaeochrous	H	H	H	**H**
Scinax eurydice	H	L	H	**L**
Scinax exiguus	H	L	H	**L**
Scinax faivovichi	H	H	U	**L**
Scinax flavidus	L	L	H	**L**
Scinax flavoguttatus	H	L	L	**L**
Scinax funereus	H	L	L	**L**
Scinax fuscomarginatus	H	L	L	**L**
Scinax fuscovarius	H	H	L	**L**
Scinax garbei	H	L	L	**L**
Scinax granulatus	H	H	L	**L**

Scinax hayii	H	L	L	**L**
Scinax heyeri	H	L	L	**L**
Scinax hiemalis	H	L	L	**L**
Scinax humilis	H	H	L	**L**
Scinax ictericus	H	L	H	**L**
Scinax jolyi	H	H	H	**H**
Scinax jureia	H	L	H	**L**
Scinax karenanneae	H	L	L	**L**
Scinax kautskyi	H	L	L	**L**
Scinax kennedyi	H	L	L	**L**
Scinax lindsayi	H	L	L	**L**
Scinax littoralis	H	L	H	**L**
Scinax littoreus	H	H	L	**L**
Scinax longilineus	H	L	L	**L**
Scinax luizotavioi	H	L	L	**L**
Scinax machadoi	H	L	L	**L**
Scinax maracaya	H	H	L	**L**
Scinax melloi	H	H	H	**H**
Scinax nasicus	H	L	L	**L**
Scinax nebulosus	H	H	H	**H**
Scinax obtriangulatus	H	L	L	**L**
Scinax oreites	H	L	L	**L**
Scinax pachycrus	H	H	L	**L**
Scinax parkeri	H	H	L	**L**
Scinax pedromedinae	H	H	H	**H**
Scinax peixotoi	H	H	U	**L**
Scinax perereca	H	H	L	**L**
Scinax perpusillus	L	H	L	**L**
Scinax pinima	H	H	L	**L**
Scinax proboscideus	H	H	H	**H**
Scinax quinquefasciatus	H	L	H	**L**
Scinax ranki	H	L	L	**L**
Scinax rizibilis	H	L	L	**L**
Scinax rostratus	H	H	H	**H**
Scinax ruber	H	H	H	**H**
Scinax similis	H	H	L	**L**
Scinax squalirostris	H	L	L	**L**
Scinax staufferi	H	H	H	**H**
Scinax strigilatus	H	L	U	**L**
Scinax sugillatus	H	H	H	**H**
Scinax trapicheiroi	H	L	L	**L**
Scinax trilineatus	H	L	H	**L**
Scinax uruguayus	H	L	L	**L**
Scinax v-signatus	L	H	L	**L**
Scinax wandae	H	L	H	**L**

Scinax x-signatus	H	L	L	**L**
Scolecomorphus kirkii	L	H	L	**L**
Scolecomorphus uluguruensis	H	H	H	**H**
Scolecomorphus vittatus	L	H	L	**L**
Scotobleps gabonicus	H	L	L	**L**
Scutiger adungensis	H	L	L	**L**
Scutiger bhutanensis	U	L	U	**L**
Scutiger boulengeri	H	L	H	**L**
Scutiger brevipes	H	L	H	**L**
Scutiger chintingensis	L	L	H	**L**
Scutiger glandulatus	L	L	L	**L**
Scutiger gongshanensis	L	H	L	**L**
Scutiger jiulongensis	U	L	L	**L**
Scutiger liupanensis	H	L	L	**L**
Scutiger maculatus	L	L	H	**L**
Scutiger mammatus	L	L	H	**L**
Scutiger muliensis	L	L	L	**L**
Scutiger nepalensis	H	H	H	**H**
Scutiger ningshanensis	H	L	L	**L**
Scutiger nyingchiensis	H	H	H	**H**
Scutiger pingwuensis	L	L	H	**L**
Scutiger ruginosus	L	L	H	**L**
Scutiger sikimmensis	L	H	L	**L**
Scutiger tuberculatus	L	L	H	**L**
Scythrophrys sawayae	H	H	L	**L**
Sechellophryne gardineri	H	H	L	**L**
Sechellophryne pipilodryas	H	H	U	**L**
Semnodactylus wealii	H	L	L	**L**
Silurana epitropicalis	H	L	L	**L**
Silurana tropicalis	L	L	L	**L**
Silverstoneia erasmios	H	L	H	**L**
Silverstoneia flotator	H	H	L	**L**
Silverstoneia nubicola	H	H	L	**L**
Siphonops annulatus	L	H	L	**L**
Siphonops hardyi	L	H	H	**L**
Siphonops insulanus	H	H	H	**H**
Siphonops leucoderus	H	H	U	**L**
Siphonops paulensis	L	H	L	**L**
Siren intermedia	H	L	L	**L**
Siren lacertina	H	L	L	**L**
Smilisca baudinii	H	L	H	**L**
Smilisca cyanosticta	H	L	H	**L**
Smilisca dentata	H	L	H	**L**
Smilisca fodiens	H	H	H	**H**
Smilisca phaeota	H	L	L	**L**

Smilisca puma	H	L	H	**L**
Smilisca sila	H	L	L	**L**
Smilisca sordida	H	H	H	**H**
Somuncuria somuncurensis	H	H	H	**H**
Sooglossus sechellensis	H	H	L	**L**
Sooglossus thomasseti	H	H	L	**L**
Spea bombifrons	H	L	L	**L**
Spea hammondii	H	L	L	**L**
Spea intermontana	H	L	H	**L**
Spea multiplicata	H	L	H	**L**
Spelaeophryne methneri	L	L	L	**L**
Speleomantes ambrosii	H	H	L	**L**
Speleomantes flavus	H	H	H	**H**
Speleomantes imperialis	H	H	H	**H**
Speleomantes italicus	H	H	L	**L**
Speleomantes sarrabusensis	H	H	H	**H**
Speleomantes strinatii	H	H	L	**L**
Speleomantes supramontis	H	H	H	**H**
Sphaenorhynchus bromelicola	H	L	L	**L**
Sphaenorhynchus caramaschii	H	H	L	**L**
Sphaenorhynchus carneus	H	L	L	**L**
Sphaenorhynchus dorisae	H	L	L	**L**
Sphaenorhynchus lacteus	H	L	H	**L**
Sphaenorhynchus orophilus	L	H	L	**L**
Sphaenorhynchus palustris	H	L	H	**L**
Sphaenorhynchus pauloalvini	H	H	H	**H**
Sphaenorhynchus planicola	H	L	L	**L**
Sphaenorhynchus platycephalus	H	U	U	**L**
Sphaenorhynchus prasinus	H	L	H	**L**
Sphaenorhynchus surdus	H	H	L	**L**
Sphaerotheca breviceps	L	H	L	**L**
Sphaerotheca dobsonii	L	H	H	**L**
Sphaerotheca leucorhynchus	H	H	L	**L**
Sphaerotheca maskeyi	H	H	H	**H**
Sphaerotheca rolandae	L	H	L	**L**
Sphaerotheca swani	H	H	H	**H**
Sphenophryne cornuta	L	H	L	**L**
Spicospina flammocaerulea	H	H	H	**H**
Spinomantis aglavei	L	H	H	**L**
Spinomantis bertini	L	H	L	**L**
Spinomantis brunae	L	H	H	**L**
Spinomantis elegans	L	H	L	**L**
Spinomantis fimbriatus	L	H	H	**L**
Spinomantis guibei	L	H	H	**L**
Spinomantis massi	L	H	H	**L**

Spinomantis microtis	L	H	H	**L**
Spinomantis peraccae	L	H	H	**L**
Spinomantis phantasticus	L	H	H	**L**
Staurois latopalmatus	L	L	H	**L**
Staurois natator	L	L	L	**L**
Staurois parvus	H	L	H	**L**
Staurois tuberilinguis	L	L	H	**L**
Stefania ackawaio	H	H	H	**H**
Stefania ayangannae	H	H	H	**H**
Stefania breweri	H	L	L	**L**
Stefania coxi	H	H	H	**H**
Stefania evansi	L	H	H	**L**
Stefania ginesi	U	H	H	**L**
Stefania goini	U	L	H	**L**
Stefania marahuaquensis	U	L	H	**L**
Stefania oculosa	U	L	H	**L**
Stefania percristata	U	L	H	**L**
Stefania riae	H	L	H	**L**
Stefania riveroi	H	H	H	**H**
Stefania roraimae	H	L	H	**L**
Stefania satelles	H	H	H	**H**
Stefania scalae	L	H	H	**L**
Stefania schuberti	H	H	H	**H**
Stefania tamacuarina	H	L	H	**L**
Stefania woodleyi	H	H	H	**H**
Stereochilus marginatus	H	H	L	**L**
Stereocyclops incrassatus	L	H	H	**L**
Stereocyclops parkeri	L	H	L	**L**
Strabomantis anatipes	L	L	H	**L**
Strabomantis anomalus	H	L	H	**L**
Strabomantis biporcatus	L	H	H	**L**
Strabomantis bufoniformis	H	L	L	**L**
Strabomantis cadenai	H	H	H	**H**
Strabomantis cerastes	L	H	H	**L**
Strabomantis cheiroplethus	H	L	H	**L**
Strabomantis cornutus	L	H	L	**L**
Strabomantis helonotus	H	H	H	**H**
Strabomantis ingeri	H	H	H	**H**
Strabomantis laticorpus	H	H	L	**L**
Strabomantis necerus	H	L	H	**L**
Strabomantis necopinus	H	H	H	**H**
Strabomantis ruizi	H	H	H	**H**
Strabomantis sulcatus	H	H	H	**H**
Strabomantis zygodactylus	H	L	L	**L**
Strongylopus bonaespei	H	H	H	**H**

Strongylopus fasciatus	H	L	L	**L**
Strongylopus fuelleborni	H	L	L	**L**
Strongylopus grayii	H	H	L	**L**
Strongylopus hymenopus	H	L	L	**L**
Strongylopus kilimanjaro	U	L	H	**L**
Strongylopus kitumbeine	L	L	L	**L**
Strongylopus merumontanus	L	H	H	**L**
Strongylopus rhodesianus	L	L	L	**L**
Strongylopus springbokensis	H	L	L	**L**
Strongylopus wageri	H	L	L	**L**
Stumpffia gimmeli	L	H	H	**L**
Stumpffia grandis	U	H	H	**L**
Stumpffia helenae	H	H	L	**L**
Stumpffia psologlossa	U	L	H	**L**
Stumpffia pygmaea	H	H	H	**H**
Stumpffia roseifemoralis	U	L	H	**L**
Stumpffia tetradactyla	U	L	L	**L**
Stumpffia tridactyla	U	H	H	**L**
Sylvacaecilia grandisonae	U	U	L	**L**
Synapturanus mirandaribeiroi	H	H	H	**H**
Synapturanus rabus	H	H	L	**L**
Synapturanus salseri	H	H	L	**L**
Syncope antenori	L	H	H	**L**
Syncope carvalhoi	H	H	L	**L**
Syncope tridactyla	H	H	L	**L**
Tachycnemis seychellensis	H	H	L	**L**
Taricha granulosa	L	H	L	**L**
Taricha rivularis	L	H	L	**L**
Taricha torosa	H	H	L	**L**
Taudactylus acutirostris	H	H	H	**H**
Taudactylus diurnus	H	H	U	**L**
Taudactylus eungellensis	H	H	H	**H**
Taudactylus liemi	H	H	H	**H**
Taudactylus pleione	H	H	L	**L**
Taudactylus rheophilus	H	H	L	**L**
Telmatobius arequipensis	H	L	H	**L**
Telmatobius atacamensis	H	L	H	**L**
Telmatobius atahualpai	H	L	H	**L**
Telmatobius bolivianus	H	L	H	**L**
Telmatobius brevipes	H	L	H	**L**
Telmatobius brevirostris	H	L	H	**L**
Telmatobius carrillae	H	L	H	**L**
Telmatobius ceiorum	H	L	H	**L**
Telmatobius chusmisensis	H	L	H	**L**
Telmatobius cirrhacelis	H	L	H	**L**

Telmatobius colanensis	H	L	L	**L**
Telmatobius contrerasi	H	L	H	**L**
Telmatobius culeus	H	L	L	**L**
Telmatobius dankoi	H	L	H	**L**
Telmatobius degener	H	L	H	**L**
Telmatobius edaphonastes	H	L	H	**L**
Telmatobius espadai	L	L	H	**L**
Telmatobius fronteriensis	H	L	H	**L**
Telmatobius gigas	H	L	H	**L**
Telmatobius halli	H	L	H	**L**
Telmatobius hauthali	H	H	L	**L**
Telmatobius hintoni	H	L	H	**L**
Telmatobius hockingi	H	L	H	**L**
Telmatobius huayra	H	L	H	**L**
Telmatobius hypselocephalus	H	L	H	**L**
Telmatobius ignavus	H	L	H	**L**
Telmatobius intermedius	H	L	H	**L**
Telmatobius jelskii	H	L	H	**L**
Telmatobius laticeps	H	L	H	**L**
Telmatobius latirostris	H	L	H	**L**
Telmatobius marmoratus	H	L	H	**L**
Telmatobius mayoloi	H	L	H	**L**
Telmatobius necopinus	H	L	L	**L**
Telmatobius niger	H	L	H	**L**
Telmatobius oxycephalus	H	L	H	**L**
Telmatobius pefauri	H	L	H	**L**
Telmatobius peruvianus	H	L	H	**L**
Telmatobius philippii	H	L	H	**L**
Telmatobius pinguiculus	H	L	H	**L**
Telmatobius pisanoi	H	L	H	**L**
Telmatobius platycephalus	H	L	H	**L**
Telmatobius punctatus	H	L	H	**L**
Telmatobius rimac	H	L	H	**L**
Telmatobius sanborni	H	L	H	**L**
Telmatobius schreiteri	H	L	H	**L**
Telmatobius scrocchii	H	L	H	**L**
Telmatobius sibiricus	H	L	H	**L**
Telmatobius simonsi	H	L	H	**L**
Telmatobius stephani	H	L	H	**L**
Telmatobius thompsoni	H	L	H	**L**
Telmatobius timens	H	L	H	**L**
Telmatobius truebae	H	L	H	**L**
Telmatobius vellardi	H	L	H	**L**
Telmatobius verrucosus	H	L	H	**L**
Telmatobius vilamensis	H	L	H	**L**

Telmatobius yuracare	H	L	H	**L**
Telmatobius zapahuirensis	H	L	H	**L**
Telmatobufo australis	L	L	L	**L**
Telmatobufo bullocki	H	L	L	**L**
Telmatobufo venustus	L	L	H	**L**
Tepuihyla aecii	U	L	H	**L**
Tepuihyla celsae	H	L	H	**L**
Tepuihyla edelcae	H	H	H	**H**
Tepuihyla galani	H	L	H	**L**
Tepuihyla luteolabris	H	L	H	**L**
Tepuihyla rimarum	H	H	H	**H**
Tepuihyla rodriguezi	H	H	H	**H**
Tepuihyla talbergae	H	L	H	**L**
Theloderma albopunctata	U	H	H	**L**
Theloderma asperum	L	H	H	**L**
Theloderma bicolor	L	H	H	**L**
Theloderma corticale	U	H	H	**L**
Theloderma gordoni	L	H	L	**L**
Theloderma horridum	H	H	L	**L**
Theloderma kwangsiense	H	L	L	**L**
Theloderma leporosum	L	H	L	**L**
Theloderma licin	L	H	L	**L**
Theloderma moloch	L	H	H	**L**
Theloderma nagalandense	U	U	U	**L**
Theloderma phrynoderma	H	L	L	**L**
Theloderma rhododiscus	L	H	L	**L**
Theloderma ryabovi	H	L	L	**L**
Theloderma stellatum	L	H	L	**L**
Thorius arboreus	H	H	H	**H**
Thorius aureus	H	H	L	**L**
Thorius boreas	H	H	H	**H**
Thorius dubitus	H	H	L	**L**
Thorius grandis	H	H	H	**H**
Thorius infernalis	H	H	L	**L**
Thorius insperatus	H	L	H	**L**
Thorius lunaris	H	H	L	**L**
Thorius macdougalli	H	H	H	**H**
Thorius magnipes	H	H	L	**L**
Thorius minutissimus	H	H	L	**L**
Thorius minydemus	H	H	H	**H**
Thorius munificus	H	H	H	**H**
Thorius narismagnus	H	H	H	**H**
Thorius narisovalis	H	H	H	**H**
Thorius omiltemi	H	H	L	**L**
Thorius papaloae	H	H	L	**L**

Thorius pennatulus	H	H	H	**H**
Thorius pulmonaris	H	H	H	**H**
Thorius schmidti	H	H	L	**L**
Thorius smithi	L	H	H	**L**
Thorius spilogaster	H	H	H	**H**
Thorius troglodytes	H	H	L	**L**
Thoropa lutzi	H	L	H	**L**
Thoropa megatympanum	H	L	L	**L**
Thoropa miliaris	H	L	L	**L**
Thoropa petropolitana	H	L	H	**L**
Thoropa saxatilis	H	L	H	**L**
Tlalocohyla godmani	L	L	H	**L**
Tlalocohyla loquax	L	L	H	**L**
Tlalocohyla picta	L	L	H	**L**
Tlalocohyla smithii	L	L	H	**L**
Tomopterna cryptotis	H	L	L	**L**
Tomopterna damarensis	H	L	L	**L**
Tomopterna delalandii	H	H	L	**L**
Tomopterna krugerensis	H	L	L	**L**
Tomopterna luganga	H	L	L	**L**
Tomopterna marmorata	H	L	L	**L**
Tomopterna natalensis	H	L	L	**L**
Tomopterna tandyi	H	L	L	**L**
Tomopterna tuberculosa	H	L	L	**L**
Trachycephalus atlas	H	L	L	**L**
Trachycephalus coriaceus	H	L	L	**L**
Trachycephalus hadroceps	H	L	H	**L**
Trachycephalus imitatrix	H	H	L	**L**
Trachycephalus jordani	H	L	H	**L**
Trachycephalus lepidus	H	L	L	**L**
Trachycephalus mesophaeus	L	H	L	**L**
Trachycephalus nigromaculatus	L	L	L	**L**
Trachycephalus resinifictrix	H	L	H	**L**
Trachycephalus venulosus	L	L	L	**L**
Trichobatrachus robustus	L	L	L	**L**
Triprion petasatus	L	H	H	**L**
Triturus carnifex	H	L	L	**L**
Triturus cristatus	H	L	H	**L**
Triturus dobrogicus	H	H	H	**H**
Triturus karelinii	H	L	L	**L**
Triturus marmoratus	H	L	L	**L**
Triturus pygmaeus	H	H	L	**L**
Truebella skoptes	H	H	H	**H**
Truebella tothastes	H	H	U	**L**
Tsingymantis antitra	H	H	H	**H**

Tylototriton asperrimus	L	H	L	**L**
Tylototriton hainanensis	L	H	L	**L**
Tylototriton kweichowensis	H	H	L	**L**
Tylototriton shanjing	L	H	L	**L**
Tylototriton taliangensis	L	H	L	**L**
Tylototriton verrucosus	L	H	H	**L**
Tylototriton vietnamensis	L	H	H	**L**
Tylototriton wenxianensis	L	H	H	**L**
Typhlonectes compressicauda	H	H	H	**H**
Typhlonectes cunhai	H	H	H	**H**
Typhlonectes natans	L	H	H	**L**
Uperodon globulosus	L	H	L	**L**
Uperodon systoma	L	H	L	**L**
Uperoleia altissima	L	L	L	**L**
Uperoleia arenicola	H	L	L	**L**
Uperoleia aspera	H	L	L	**L**
Uperoleia borealis	H	L	H	**L**
Uperoleia capitulata	H	L	L	**L**
Uperoleia crassa	H	L	L	**L**
Uperoleia daviesae	H	L	L	**L**
Uperoleia fusca	H	L	L	**L**
Uperoleia glandulosa	H	L	L	**L**
Uperoleia inundata	H	H	L	**L**
Uperoleia laevigata	H	H	L	**L**
Uperoleia lithomoda	H	L	L	**L**
Uperoleia littlejohni	H	L	L	**L**
Uperoleia marmorata	H	L	L	**L**
Uperoleia martini	H	L	L	**L**
Uperoleia micromeles	H	L	L	**L**
Uperoleia mimula	L	H	L	**L**
Uperoleia minima	H	L	L	**L**
Uperoleia mjobergii	H	H	L	**L**
Uperoleia orientalis	H	L	L	**L**
Uperoleia rugosa	H	H	L	**L**
Uperoleia russelli	H	L	L	**L**
Uperoleia talpa	H	L	L	**L**
Uperoleia trachyderma	H	L	L	**L**
Uperoleia tyleri	H	L	L	**L**
Uraeotyphlus interruptus	H	U	H	**L**
Uraeotyphlus malabaricus	H	U	H	**L**
Uraeotyphlus menoni	U	U	H	**L**
Uraeotyphlus narayani	U	U	H	**L**
Uraeotyphlus oommeni	U	U	U	**L**
Uraeotyphlus oxyurus	U	U	H	**L**
Vandijkophrynus amatolicus	H	H	H	**H**

Vandijkophrynus angusticeps	H	H	H	**H**
Vandijkophrynus gariepensis	H	L	L	**L**
Vandijkophrynus inyangae	H	H	H	**H**
Vandijkophrynus robinsoni	H	L	L	**L**
Wakea madinika	H	H	H	**H**
Werneria bambutensis	L	L	H	**L**
Werneria iboundji	H	L	H	**L**
Werneria mertensiana	L	L	L	**L**
Werneria preussi	L	L	L	**L**
Werneria submontana	L	L	L	**L**
Werneria tandyi	H	L	L	**L**
Wolterstorffina chirioi	H	H	H	**H**
Wolterstorffina mirei	H	H	H	**H**
Wolterstorffina parvipalmata	H	H	L	**L**
Xenohyla eugenioi	H	L	L	**L**
Xenohyla truncata	H	L	L	**L**
Xenophrys aceras	L	L	L	**L**
Xenophrys auralensis	H	L	L	**L**
Xenophrys baluensis	H	H	H	**H**
Xenophrys binchuanensis	L	L	H	**L**
Xenophrys boettgeri	L	L	H	**L**
Xenophrys brachykolos	L	L	H	**L**
Xenophrys caudoprocta	U	L	H	**L**
Xenophrys daweimontis	U	L	H	**L**
Xenophrys dringi	H	H	H	**H**
Xenophrys glandulosa	L	H	L	**L**
Xenophrys huangshanensis	U	L	L	**L**
Xenophrys jingdongensis	L	L	H	**L**
Xenophrys kuatunensis	L	L	L	**L**
Xenophrys lekaguli	U	L	L	**L**
Xenophrys longipes	L	L	H	**L**
Xenophrys major	L	L	H	**L**
Xenophrys mangshanensis	L	L	L	**L**
Xenophrys medogensis	U	L	H	**L**
Xenophrys minor	L	L	L	**L**
Xenophrys nankiangensis	H	L	H	**L**
Xenophrys omeimontis	L	L	H	**L**
Xenophrys pachyproctus	U	L	H	**L**
Xenophrys palpebralespinosa	L	L	H	**L**
Xenophrys parallela	H	L	H	**L**
Xenophrys parva	L	L	H	**L**
Xenophrys robusta	U	L	H	**L**
Xenophrys serchhipii	U	L	H	**L**
Xenophrys shuichengensis	U	L	L	**L**
Xenophrys spinata	L	L	L	**L**

Xenophrys wawuensis	U	L	H	**L**
Xenophrys wuliangshanensis	U	L	L	**L**
Xenophrys wushanensis	L	L	H	**L**
Xenophrys zhangi	U	L	H	**L**
Xenophrys zunhebotoensis	H	L	L	**L**
Xenopus amieti	H	L	L	**L**
Xenopus andrei	H	L	L	**L**
Xenopus borealis	H	L	H	**L**
Xenopus boumbaensis	H	L	L	**L**
Xenopus clivii	L	L	L	**L**
Xenopus fraseri	H	L	L	**L**
Xenopus gilli	H	H	H	**H**
Xenopus laevis	L	L	L	**L**
Xenopus largeni	H	L	L	**L**
Xenopus longipes	H	H	H	**H**
Xenopus muelleri	L	L	L	**L**
Xenopus petersii	L	L	L	**L**
Xenopus pygmaeus	H	L	L	**L**
Xenopus ruwenzoriensis	H	L	H	**L**
Xenopus vestitus	H	L	L	**L**
Xenopus victorianus	L	L	L	**L**
Xenopus wittei	H	L	L	**L**
Xenorhina adisca	H	H	H	**H**
Xenorhina anorbis	U	L	H	**L**
Xenorhina arboricola	H	H	H	**H**
Xenorhina arfakiana	H	L	H	**L**
Xenorhina bidens	H	H	U	**L**
Xenorhina bouwensi	L	H	H	**L**
Xenorhina eiponis	H	H	H	**H**
Xenorhina fuscigula	L	H	H	**L**
Xenorhina gigantea	H	L	H	**L**
Xenorhina huon	H	L	H	**L**
Xenorhina lanthanites	H	L	H	**L**
Xenorhina macrodisca	H	H	H	**H**
Xenorhina macrops	H	H	H	**H**
Xenorhina mehelyi	L	H	H	**L**
Xenorhina minima	H	H	H	**H**
Xenorhina multisica	H	L	H	**L**
Xenorhina obesa	H	H	H	**H**
Xenorhina ocellata	H	L	H	**L**
Xenorhina ophiodon	H	H	H	**H**
Xenorhina oxycephala	L	H	L	**L**
Xenorhina parkerorum	L	H	H	**L**
Xenorhina rostrata	L	H	H	**L**
Xenorhina scheepstrai	H	H	H	**H**

Xenorhina schiefenhoeveli	H	H	H	**H**
Xenorhina similis	H	H	H	**H**
Xenorhina subcrocea	H	H	H	**H**
Xenorhina tumulus	H	L	H	**L**
Xenorhina varia	H	H	H	**H**
Xenorhina zweifeli	H	L	H	**L**
Yerana yei	U	U	H	**L**
Zachaenus carvalhoi	H	H	L	**L**
Zachaenus parvulus	L	H	H	**L**
Zachaenus roseus	H	H	U	**L**
