# Supplementary material for: Identifying the World's Most Climate Change Vulnerable Species: A Systematic Trait-Based Assessment of all Birds, Amphibians and Corals
Source: PLoS One. 2013 Jun 12;8(6):e65427. doi: 10.1371/journal.pone.0065427 (PMC3680427; doi:10.1371/journal.pone.0065427)
Supplement: Appendix C — Climate change vulnerability scores for coral species. (PDF) [file pone.0065427.s038.pdf]

Appendix C:
Climate change vulnerability assessments by species for **corals**

*Assessments of overall vulnerability are based on optimistic assumptions for missing trait information.

Species	SENSI-TIVITY	UN-ADAPTA-BILITY	EXPO-SURE	**OVERALL VULNERA-BILITY***
Acanthastrea bowerbanki	H	H	L	**L**
Acanthastrea brevis	H	H	H	**H**
Acanthastrea echinata	H	H	L	**L**
Acanthastrea faviaformis	H	H	H	**H**
Acanthastrea hemprichii	H	H	L	**L**
Acanthastrea hillae	H	H	L	**L**
Acanthastrea ishigakiensis	H	H	L	**L**
Acanthastrea lordhowensis	H	H	H	**H**
Acanthastrea maxima	H	H	U	**L**
Acanthastrea regularis	H	H	L	**L**
Acanthastrea rotundoflora	H	H	L	**L**
Acanthastrea subechinata	H	H	L	**L**
Acropora abrolhosensis	H	L	H	**L**
Acropora abrotanoides	H	L	L	**L**
Acropora aculeus	H	L	L	**L**
Acropora acuminata	H	L	L	**L**
Acropora akajimensis	H	L	H	**L**
Acropora anthocercis	H	L	L	**L**
Acropora appressa	H	H	L	**L**
Acropora arabensis	H	H	U	**L**
Acropora aspera	H	L	L	**L**
Acropora austera	H	L	L	**L**
Acropora awi	H	L	H	**L**
Acropora batunai	H	L	H	**L**
Acropora bifurcata	H	L	H	**L**
Acropora branchi	H	H	L	**L**
Acropora bushyensis	H	L	H	**L**
Acropora cardenae	H	L	H	**L**
Acropora carduus	H	L	L	**L**
Acropora caroliniana	H	L	H	**L**
Acropora cerealis	H	L	L	**L**
Acropora cervicornis	H	L	H	**L**
Acropora chesterfieldensis	H	L	L	**L**
Acropora clathrata	H	L	L	**L**
Acropora convexa	H	L	H	**L**
Acropora cophodactyla	H	L	L	**L**

Acropora copiosa	H	L	L	**L**
Acropora cytherea	H	L	L	**L**
Acropora dendrum	H	L	L	**L**
Acropora derawanensis	H	L	H	**L**
Acropora desalwii	H	L	H	**L**
Acropora digitifera	H	L	L	**L**
Acropora divaricata	H	L	L	**L**
Acropora donei	H	L	L	**L**
Acropora downingi	H	H	U	**L**
Acropora echinata	H	L	L	**L**
Acropora efflorescens	H	L	L	**L**
Acropora elegans	H	L	H	**L**
Acropora elegantula	H	L	H	**L**
Acropora elseyi	H	L	L	**L**
Acropora exquisita	H	L	L	**L**
Acropora fastigata	H	L	H	**L**
Acropora fenneri	H	L	H	**L**
Acropora filiformis	H	L	H	**L**
Acropora florida	H	L	L	**L**
Acropora forskali	H	H	H	**H**
Acropora gemmifera	H	L	L	**L**
Acropora glauca	H	L	L	**L**
Acropora globiceps	H	L	L	**L**
Acropora gomezi	H	L	H	**L**
Acropora grandis	H	L	L	**L**
Acropora granulosa	H	L	L	**L**
Acropora haimei	H	H	U	**L**
Acropora halmaherae	H	L	H	**L**
Acropora hemprichii	H	H	L	**L**
Acropora hoeksemai	H	L	H	**L**
Acropora horrida	H	L	L	**L**
Acropora humilis	H	L	L	**L**
Acropora hyacinthus	H	L	L	**L**
Acropora indonesia	H	L	H	**L**
Acropora inermis	H	L	L	**L**
Acropora insignis	H	L	L	**L**
Acropora irregularis	H	L	H	**L**
Acropora jacquelineae	H	L	H	**L**
Acropora japonica	H	H	H	**H**
Acropora kimbeensis	H	L	H	**L**
Acropora kirstyae	H	L	L	**L**
Acropora kosurini	H	H	L	**L**
Acropora lamarcki	H	H	H	**H**
Acropora latistella	H	L	L	**L**
Acropora lianae	H	L	H	**L**

Acropora listeri	H	L	L	**L**
Acropora loisetteae	H	L	L	**L**
Acropora lokani	H	L	L	**L**
Acropora longicyathus	H	L	L	**L**
Acropora loripes	H	L	L	**L**
Acropora lovelli	H	L	L	**L**
Acropora lutkeni	H	L	L	**L**
Acropora macrostoma	H	L	H	**L**
Acropora maryae	H	H	U	**L**
Acropora massawensis	H	H	U	**L**
Acropora meridiana	H	L	H	**L**
Acropora microclados	H	L	L	**L**
Acropora microphthalma	H	L	L	**L**
Acropora millepora	H	L	L	**L**
Acropora minuta	H	L	H	**L**
Acropora mirabilis	H	L	H	**L**
Acropora monticulosa	H	L	L	**L**
Acropora multiacuta	H	L	L	**L**
Acropora muricata	H	L	U	**L**
Acropora nana	H	L	L	**L**
Acropora nasuta	H	L	L	**L**
Acropora natalensis	H	H	H	**H**
Acropora navini	H	L	H	**L**
Acropora nobilis	H	L	L	**L**
Acropora ocellata	H	H	U	**L**
Acropora orbicularis	H	L	H	**L**
Acropora palmata	H	L	H	**L**
Acropora palmerae	H	L	L	**L**
Acropora paniculata	H	L	L	**L**
Acropora papillare	H	L	H	**L**
Acropora parahemprichii	H	L	H	**L**
Acropora parapharaonis	H	H	U	**L**
Acropora parilis	H	L	H	**L**
Acropora pectinatus	H	L	L	**L**
Acropora pharaonis	H	H	L	**L**
Acropora pichoni	H	L	H	**L**
Acropora pinguis	H	L	H	**L**
Acropora plana	H	L	L	**L**
Acropora plantaginea	H	H	H	**H**
Acropora plumosa	H	L	H	**L**
Acropora polystoma	H	L	L	**L**
Acropora prostrata	H	L	L	**L**
Acropora proximalis	H	L	H	**L**
Acropora pruinosa	H	H	H	**H**
Acropora pulchra	H	L	L	**L**

Acropora rambleri	H	L	L	**L**
Acropora retusa	H	L	L	**L**
Acropora robusta	H	L	L	**L**
Acropora rongelapensis	H	L	H	**L**
Acropora rosaria	H	L	L	**L**
Acropora roseni	H	H	H	**H**
Acropora rudis	H	H	L	**L**
Acropora rufus	H	H	U	**L**
Acropora russelli	H	L	H	**L**
Acropora samoensis	H	L	L	**L**
Acropora sarmentosa	H	L	L	**L**
Acropora scherzeriana	H	L	H	**L**
Acropora schmitti	H	L	L	**L**
Acropora secale	H	L	L	**L**
Acropora sekiseiensis	H	L	H	**L**
Acropora selago	H	L	L	**L**
Acropora seriata	H	L	H	**L**
Acropora simplex	H	L	H	**L**
Acropora solitaryensis	H	L	L	**L**
Acropora spathulata	H	L	H	**L**
Acropora speciosa	H	L	L	**L**
Acropora spicifera	H	L	L	**L**
Acropora squarrosa	H	H	H	**H**
Acropora stoddarti	H	L	L	**L**
Acropora striata	H	L	H	**L**
Acropora subglabra	H	L	L	**L**
Acropora subulata	H	L	L	**L**
Acropora suharsonoi	H	L	H	**L**
Acropora sukarnoi	H	L	U	**L**
Acropora tanegashimensis	H	H	U	**L**
Acropora tenella	H	L	H	**L**
Acropora tenuis	H	L	L	**L**
Acropora teres	H	L	L	**L**
Acropora tizardi	H	L	L	**L**
Acropora torihalimeda	H	L	H	**L**
Acropora torresiana	H	L	H	**L**
Acropora tortuosa	H	L	L	**L**
Acropora tumida	H	L	H	**L**
Acropora turaki	H	L	H	**L**
Acropora tutuilensis	H	L	H	**L**
Acropora valenciennesi	H	L	L	**L**
Acropora valida	H	L	L	**L**
Acropora variabilis	H	L	H	**L**
Acropora variolosa	H	H	U	**L**
Acropora vaughani	H	L	L	**L**

Acropora vermiculata	H	L	H	**L**
Acropora verweyi	H	L	L	**L**
Acropora walindii	H	L	H	**L**
Acropora wallaceae	H	L	L	**L**
Acropora willisae	H	L	H	**L**
Acropora yongei	H	L	L	**L**
Agaricia agaricites	H	L	H	**L**
Agaricia fragilis	H	L	H	**L**
Agaricia grahamae	H	L	H	**L**
Agaricia humilis	H	H	H	**H**
Agaricia lamarcki	H	L	H	**L**
Agaricia tenuifolia	H	L	H	**L**
Agaricia undata	H	L	H	**L**
Alveopora allingi	H	L	L	**L**
Alveopora catalai	H	L	L	**L**
Alveopora daedalea	H	L	H	**L**
Alveopora excelsa	H	L	H	**L**
Alveopora fenestrata	H	H	L	**L**
Alveopora gigas	H	L	H	**L**
Alveopora japonica	H	H	U	**L**
Alveopora marionensis	H	H	L	**L**
Alveopora minuta	H	H	H	**H**
Alveopora ocellata	H	L	H	**L**
Alveopora spongiosa	H	L	L	**L**
Alveopora tizardi	H	L	L	**L**
Alveopora verrilliana	H	L	L	**L**
Alveopora viridis	H	H	H	**H**
Anacropora forbesi	H	L	L	**L**
Anacropora matthai	H	H	L	**L**
Anacropora pillai	H	L	H	**L**
Anacropora puertogalerae	H	L	L	**L**
Anacropora reticulata	H	L	H	**L**
Anacropora spinosa	H	L	H	**L**
Anacropora spumosa	H	H	U	**L**
Anomastraea irregularis	H	H	U	**L**
Astrangia poculata	H	L	H	**L**
Astreopora cucullata	H	L	L	**L**
Astreopora expansa	H	L	L	**L**
Astreopora gracilis	H	H	L	**L**
Astreopora incrustans	H	L	H	**L**
Astreopora listeri	H	H	L	**L**
Astreopora macrostoma	H	H	L	**L**
Astreopora moretonensis	H	L	L	**L**
Astreopora myriophthalma	H	H	L	**L**
Astreopora ocellata	H	H	L	**L**

Astreopora randalli	H	L	L	**L**
Astreopora scabra	H	H	L	**L**
Astreopora suggesta	H	H	L	**L**
Australogyra zelli	H	H	H	**H**
Australomussa rowleyensis	H	H	L	**L**
Balanophyllia europaea	H	H	U	**L**
Barabattoia amicorum	H	H	L	**L**
Barabattoia laddi	H	H	L	**L**
Blastomussa merleti	H	H	L	**L**
Blastomussa wellsi	H	H	L	**L**
Boninastrea boninensis	H	U	H	**L**
Calathiscus tantillus	H	H	U	**L**
Cantharellus doederleini	H	H	U	**L**
Cantharellus jebbi	H	H	L	**L**
Cantharellus noumeae	H	H	H	**H**
Catalaphyllia jardinei	H	L	L	**L**
Caulastrea connata	H	H	H	**H**
Caulastrea curvata	H	L	L	**L**
Caulastrea echinulata	H	L	H	**L**
Caulastrea furcata	H	L	L	**L**
Caulastrea tumida	H	L	L	**L**
Cladocora arbuscula	H	L	H	**L**
Cladocora caespitosa	H	H	U	**L**
Coeloseris mayeri	H	H	L	**L**
Colpophyllia natans	H	H	H	**H**
Coscinaraea columna	H	L	L	**L**
Coscinaraea crassa	H	L	H	**L**
Coscinaraea exesa	L	L	L	**L**
Coscinaraea hahazimaensis	H	H	U	**L**
Coscinaraea marshae	H	H	H	**H**
Coscinaraea mcneilli	H	H	H	**H**
Coscinaraea monile	H	L	L	**L**
Coscinaraea wellsi	H	L	L	**L**
Ctenactis albitentaculata	H	H	L	**L**
Ctenactis crassa	H	H	L	**L**
Ctenactis echinata	H	H	L	**L**
Ctenella chagius	H	H	H	**H**
Cycloseris costulata	H	H	U	**L**
Cycloseris curvata	H	H	U	**L**
Cycloseris cyclolites	H	H	U	**L**
Cycloseris hexagonalis	H	H	U	**L**
Cycloseris sinensis	H	H	U	**L**
Cycloseris somervillei	H	H	U	**L**
Cycloseris tenuis	H	H	U	**L**
Cycloseris vaughani	H	H	U	**L**

Cynarina lacrymalis	H	H	L	**L**
Cyphastrea agassizi	H	H	L	**L**
Cyphastrea chalcidicum	H	H	L	**L**
Cyphastrea decadia	H	L	L	**L**
Cyphastrea hexasepta	H	H	U	**L**
Cyphastrea japonica	H	H	H	**H**
Cyphastrea microphthalma	H	H	L	**L**
Cyphastrea ocellina	H	H	L	**L**
Cyphastrea serailia	H	H	L	**L**
Dendrogyra cylindrus	H	H	H	**H**
Diaseris distorta	H	H	U	**L**
Diaseris fragilis	H	H	U	**L**
Dichocoenia stokesi	H	H	U	**L**
Diploastrea heliopora	H	H	L	**L**
Diploria clivosa	H	H	H	**H**
Diploria labyrinthiformis	H	H	H	**H**
Diploria strigosa	H	H	H	**H**
Duncanopsammia axifuga	H	L	H	**L**
Echinomorpha nishihirai	H	H	L	**L**
Echinophyllia aspera	H	L	L	**L**
Echinophyllia costata	H	L	H	**L**
Echinophyllia echinata	H	L	L	**L**
Echinophyllia echinoporoides	H	L	L	**L**
Echinophyllia orpheensis	H	L	H	**L**
Echinophyllia patula	H	L	L	**L**
Echinophyllia pectinata	H	L	H	**L**
Echinopora ashmorensis	H	L	H	**L**
Echinopora forskaliana	H	H	H	**H**
Echinopora fruticulosa	H	H	H	**H**
Echinopora gemmacea	H	L	L	**L**
Echinopora hirsutissima	H	L	L	**L**
Echinopora horrida	H	L	L	**L**
Echinopora irregularis	H	H	U	**L**
Echinopora lamellosa	H	L	L	**L**
Echinopora mammiformis	H	L	L	**L**
Echinopora pacificus	H	L	L	**L**
Echinopora robusta	H	L	L	**L**
Echinopora taylorae	H	L	H	**L**
Echinopora tiranensis	H	H	U	**L**
Erythrastrea flabellata	H	H	U	**L**
Euphyllia ancora	H	L	L	**L**
Euphyllia cristata	H	L	L	**L**
Euphyllia divisa	H	L	L	**L**
Euphyllia glabrescens	H	L	L	**L**
Euphyllia paraancora	H	L	H	**L**

Euphyllia paradivisa	H	L	H	**L**
Euphyllia paraglabrescens	H	H	H	**H**
Euphyllia yaeyamaensis	H	L	L	**L**
Eusmilia fastigiata	H	H	H	**H**
Favia albidus	H	H	U	**L**
Favia danae	H	H	L	**L**
Favia favus	H	H	L	**L**
Favia fragum	H	H	H	**H**
Favia helianthoides	H	H	L	**L**
Favia lacuna	H	H	U	**L**
Favia laxa	H	H	L	**L**
Favia leptophylla	H	H	L	**L**
Favia lizardensis	H	H	L	**L**
Favia maritima	H	H	L	**L**
Favia marshae	H	H	H	**H**
Favia matthaii	H	H	L	**L**
Favia maxima	H	H	L	**L**
Favia pallida	H	H	L	**L**
Favia rosaria	H	H	L	**L**
Favia rotumana	H	H	L	**L**
Favia rotundata	H	H	L	**L**
Favia speciosa	H	H	L	**L**
Favia stelligera	H	H	L	**L**
Favia truncatus	H	H	L	**L**
Favia veroni	H	H	L	**L**
Favia vietnamensis	H	H	H	**H**
Favites abdita	H	H	L	**L**
Favites acuticollis	H	H	H	**H**
Favites bestae	H	H	L	**L**
Favites chinensis	H	H	L	**L**
Favites complanata	H	H	L	**L**
Favites flexuosa	H	H	L	**L**
Favites halicora	H	H	L	**L**
Favites micropentagona	H	H	H	**H**
Favites paraflexuosa	H	H	L	**L**
Favites pentagona	H	H	L	**L**
Favites russelli	H	H	L	**L**
Favites spinosa	H	H	L	**L**
Favites stylifera	H	H	H	**H**
Favites vasta	H	H	L	**L**
Fungia concinna	H	H	L	**L**
Fungia fralinae	H	H	H	**H**
Fungia fungites	H	H	L	**L**
Fungia granulosa	H	H	L	**L**
Fungia horrida	H	H	L	**L**

Fungia moluccensis	H	H	L	**L**
Fungia paumotensis	H	H	L	**L**
Fungia puishani	H	H	L	**L**
Fungia repanda	H	H	L	**L**
Fungia scabra	H	H	L	**L**
Fungia scruposa	H	H	L	**L**
Fungia scutaria	H	H	L	**L**
Fungia seychellensis	H	H	H	**H**
Fungia spinifer	H	H	L	**L**
Fungia taiwanensis	H	H	H	**H**
Galaxea acrhelia	H	L	H	**L**
Galaxea astreata	H	H	L	**L**
Galaxea cryptoramosa	H	L	H	**L**
Galaxea fascicularis	H	L	L	**L**
Galaxea horrescens	H	L	L	**L**
Galaxea longisepta	H	L	H	**L**
Galaxea paucisepta	H	L	H	**L**
Gardineroseris planulata	H	H	L	**L**
Goniastrea aspera	H	H	L	**L**
Goniastrea australensis	H	H	L	**L**
Goniastrea columella	H	H	H	**H**
Goniastrea deformis	H	H	H	**H**
Goniastrea edwardsi	H	H	L	**L**
Goniastrea favulus	H	H	L	**L**
Goniastrea minuta	H	H	L	**L**
Goniastrea palauensis	H	H	L	**L**
Goniastrea pectinata	H	H	L	**L**
Goniastrea peresi	H	H	H	**H**
Goniastrea ramosa	H	H	L	**L**
Goniastrea retiformis	H	H	L	**L**
Goniastrea thecata	H	H	U	**L**
Goniopora albiconus	H	L	H	**L**
Goniopora burgosi	H	L	H	**L**
Goniopora cellulosa	H	H	H	**H**
Goniopora ciliatus	H	H	U	**L**
Goniopora columna	H	L	L	**L**
Goniopora djiboutiensis	H	L	L	**L**
Goniopora eclipsensis	H	L	H	**L**
Goniopora fruticosa	H	L	L	**L**
Goniopora lobata	H	L	L	**L**
Goniopora minor	H	H	L	**L**
Goniopora norfolkensis	H	L	H	**L**
Goniopora palmensis	H	L	H	**L**
Goniopora pandoraensis	H	L	L	**L**
Goniopora pearsoni	H	H	U	**L**

Goniopora planulata	H	L	L	**L**
Goniopora polyformis	H	L	H	**L**
Goniopora savignyi	H	H	U	**L**
Goniopora somaliensis	H	L	L	**L**
Goniopora stokesi	H	H	L	**L**
Goniopora stutchburyi	H	L	L	**L**
Goniopora sultani	H	H	U	**L**
Goniopora tenella	H	L	H	**L**
Goniopora tenuidens	H	H	L	**L**
Gyrosmilia interrupta	H	H	U	**L**
Halomitra clavator	H	H	H	**H**
Halomitra pileus	H	H	L	**L**
Heliofungia actiniformis	H	H	L	**L**
Heliopora coerulea	H	H	L	**L**
Herpolitha limax	H	H	L	**L**
Heterocyathus aequicostatus	H	H	L	**L**
Heteropsammia cochlea	H	H	L	**L**
Horastrea indica	H	H	L	**L**
Hydnophora bonsai	H	L	H	**L**
Hydnophora exesa	H	L	L	**L**
Hydnophora grandis	H	L	L	**L**
Hydnophora microconos	H	H	L	**L**
Hydnophora pilosa	H	L	L	**L**
Hydnophora rigida	H	L	L	**L**
Indophyllia macassarensis	H	H	H	**H**
Isophyllia rigida	H	H	U	**L**
Isophyllia sinuosa	H	H	H	**H**
Isopora brueggemanni	H	L	H	**L**
Isopora crateriformis	H	L	L	**L**
Isopora cuneata	H	L	L	**L**
Isopora cylindrica	H	L	H	**L**
Isopora elizabethensis	H	H	H	**H**
Isopora palifera	H	L	L	**L**
Isopora togianensis	H	L	H	**L**
Leptastrea aequalis	H	H	L	**L**
Leptastrea bewickensis	H	H	L	**L**
Leptastrea bottae	H	H	L	**L**
Leptastrea inaequalis	H	H	L	**L**
Leptastrea pruinosa	H	H	L	**L**
Leptastrea purpurea	H	H	L	**L**
Leptastrea transversa	H	H	L	**L**
Leptoria irregularis	H	H	L	**L**
Leptoria phrygia	H	H	L	**L**
Leptoseris amitoriensis	H	L	H	**L**
Leptoseris cailleti	H	L	H	**L**

Leptoseris cucullata	H	L	U	**L**
Leptoseris explanata	H	L	L	**L**
Leptoseris foliosa	H	L	L	**L**
Leptoseris gardineri	H	L	L	**L**
Leptoseris hawaiiensis	H	L	L	**L**
Leptoseris incrustans	H	L	L	**L**
Leptoseris mycetoseroides	H	L	L	**L**
Leptoseris papyracea	H	L	L	**L**
Leptoseris scabra	H	L	L	**L**
Leptoseris solida	H	L	L	**L**
Leptoseris striata	H	L	H	**L**
Leptoseris tubulifera	H	L	H	**L**
Leptoseris yabei	H	L	L	**L**
Lithophyllon lobata	H	L	U	**L**
Lithophyllon mokai	H	H	L	**L**
Lithophyllon undulatum	H	L	H	**L**
Lobophyllia corymbosa	H	H	L	**L**
Lobophyllia dentatus	H	H	L	**L**
Lobophyllia diminuta	H	H	L	**L**
Lobophyllia flabelliformis	H	H	H	**H**
Lobophyllia hataii	H	H	L	**L**
Lobophyllia hemprichii	H	H	L	**L**
Lobophyllia pachysepta	H	H	L	**L**
Lobophyllia robusta	H	H	L	**L**
Lobophyllia serratus	H	H	H	**H**
Madracis asanoi	H	H	H	**H**
Madracis asperula	H	H	H	**H**
Madracis decactis	H	L	H	**L**
Madracis formosa	H	H	H	**H**
Madracis kirbyi	H	H	L	**L**
Madracis mirabilis	H	H	U	**L**
Madracis pharensis	H	H	H	**H**
Madracis senaria	H	H	H	**H**
Manicina areolata	H	H	H	**H**
Meandrina braziliensis	H	H	L	**L**
Meandrina meandrites	H	H	H	**H**
Merulina ampliata	H	L	L	**L**
Merulina scabricula	H	L	L	**L**
Merulina scheeri	H	H	U	**L**
Micromussa amakusensis	H	H	L	**L**
Micromussa diminuta	H	H	H	**H**
Micromussa minuta	H	H	H	**H**
Millepora alcicornis	H	U	H	**L**
Millepora complanata	H	L	H	**L**
Millepora dichotoma	H	L	L	**L**

Millepora exaesa	H	L	L	**L**
Millepora intricata	H	L	L	**L**
Millepora platyphylla	H	L	L	**L**
Millepora squarrosa	H	L	L	**L**
Millepora tenera	H	L	L	**L**
Montastrea annularis	H	H	U	**L**
Montastrea annuligera	H	H	L	**L**
Montastrea cavernosa	H	H	U	**L**
Montastrea colemani	H	H	L	**L**
Montastrea curta	H	H	L	**L**
Montastrea magnistellata	H	H	L	**L**
Montastrea multipunctata	H	H	L	**L**
Montastrea salebrosa	H	H	L	**L**
Montastrea serageldini	H	H	L	**L**
Montastrea valenciennesi	H	H	L	**L**
Montipora aequituberculata	H	H	L	**L**
Montipora altasepta	H	H	L	**L**
Montipora angulata	H	H	L	**L**
Montipora aspergillus	H	H	U	**L**
Montipora australiensis	H	H	L	**L**
Montipora cactus	H	H	H	**H**
Montipora calcarea	H	H	L	**L**
Montipora caliculata	H	H	L	**L**
Montipora capitata	H	H	L	**L**
Montipora capricornis	H	H	L	**L**
Montipora cebuensis	H	H	L	**L**
Montipora circumvallata	H	H	U	**L**
Montipora cocosensis	H	H	H	**H**
Montipora confusa	H	H	L	**L**
Montipora corbettensis	H	H	L	**L**
Montipora crassituberculata	H	H	L	**L**
Montipora cryptus	H	H	U	**L**
Montipora danae	H	H	L	**L**
Montipora delicatula	H	H	H	**H**
Montipora digitata	H	H	L	**L**
Montipora dilatata	H	H	H	**H**
Montipora echinata	H	H	U	**L**
Montipora efflorescens	H	H	L	**L**
Montipora effusa	H	H	L	**L**
Montipora flabellata	H	H	H	**H**
Montipora florida	H	H	H	**H**
Montipora floweri	H	H	L	**L**
Montipora foliosa	H	H	L	**L**
Montipora foveolata	H	H	L	**L**
Montipora friabilis	H	H	H	**H**

Montipora gaimardi	H	H	H	**H**
Montipora grisea	H	H	L	**L**
Montipora hemispherica	H	H	U	**L**
Montipora hirsuta	H	H	H	**H**
Montipora hispida	H	H	L	**L**
Montipora hodgsoni	H	H	H	**H**
Montipora hoffmeisteri	H	H	L	**L**
Montipora incrassata	H	H	L	**L**
Montipora informis	H	H	L	**L**
Montipora kellyi	H	H	U	**L**
Montipora lobulata	H	H	L	**L**
Montipora mactanensis	H	H	H	**H**
Montipora malampaya	H	H	H	**H**
Montipora meandrina	H	H	H	**H**
Montipora millepora	H	H	L	**L**
Montipora mollis	H	H	L	**L**
Montipora monasteriata	H	H	L	**L**
Montipora niugini	H	H	H	**H**
Montipora nodosa	H	H	L	**L**
Montipora orientalis	H	H	H	**H**
Montipora pachytuberculata	H	H	U	**L**
Montipora palawanensis	H	H	H	**H**
Montipora patula	H	H	H	**H**
Montipora peltiformis	H	H	L	**L**
Montipora porites	H	H	H	**H**
Montipora samarensis	H	H	L	**L**
Montipora saudii	H	H	U	**L**
Montipora setosa	H	H	H	**H**
Montipora spongiosa	H	H	U	**L**
Montipora spongodes	H	H	L	**L**
Montipora spumosa	H	H	L	**L**
Montipora stellata	H	H	H	**H**
Montipora stilosa	H	H	H	**H**
Montipora taiwanensis	H	H	H	**H**
Montipora tuberculosa	H	H	L	**L**
Montipora turgescens	H	H	L	**L**
Montipora turtlensis	H	H	H	**H**
Montipora undata	H	H	L	**L**
Montipora venosa	H	H	L	**L**
Montipora verrilli	H	H	L	**L**
Montipora verrucosa	H	L	L	**L**
Montipora verruculosus	H	H	H	**H**
Montipora vietnamensis	H	H	H	**H**
Moseleya latistellata	H	H	H	**H**
Mussa angulosa	H	H	H	**H**

Mussismilia braziliensis	H	H	L	**L**
Mussismilia harttii	H	H	L	**L**
Mussismilia hispida	H	H	L	**L**
Mycedium elephantotus	H	L	L	**L**
Mycedium mancaoi	H	L	L	**L**
Mycedium robokaki	H	L	L	**L**
Mycedium steeni	H	L	H	**L**
Mycedium umbra	H	H	U	**L**
Mycetophyllia aliciae	H	H	H	**H**
Mycetophyllia danaana	H	H	H	**H**
Mycetophyllia ferox	H	H	H	**H**
Mycetophyllia lamarckiana	H	H	H	**H**
Mycetophyllia reesi	H	L	H	**L**
Nemenzophyllia turbida	H	L	H	**L**
Oculina diffusa	H	H	H	**H**
Oculina patagonica	H	H	U	**L**
Oculina robusta	H	H	H	**H**
Oculina valenciennesi	H	H	H	**H**
Oculina varicosa	H	H	H	**H**
Oulastrea crispata	H	H	L	**L**
Oulophyllia bennettae	H	H	L	**L**
Oulophyllia crispa	H	H	L	**L**
Oulophyllia levis	H	H	H	**H**
Oxypora convoluta	H	H	U	**L**
Oxypora crassispinosa	H	L	L	**L**
Oxypora egyptensis	H	H	U	**L**
Oxypora glabra	H	L	L	**L**
Oxypora lacera	H	L	L	**L**
Pachyseris foliosa	H	L	H	**L**
Pachyseris gemmae	H	L	L	**L**
Pachyseris involuta	H	L	H	**L**
Pachyseris rugosa	H	L	L	**L**
Pachyseris speciosa	H	L	L	**L**
Palauastrea ramosa	H	H	L	**L**
Paraclavarina triangularis	H	L	L	**L**
Parasimplastrea sheppardi	H	H	L	**L**
Pavona bipartita	H	H	L	**L**
Pavona cactus	H	L	L	**L**
Pavona clavus	H	H	L	**L**
Pavona danai	H	L	H	**L**
Pavona decussata	H	L	L	**L**
Pavona diffluens	H	H	U	**L**
Pavona duerdeni	H	H	L	**L**
Pavona explanulata	H	L	L	**L**
Pavona frondifera	H	L	L	**L**

Pavona gigantea	H	H	L	**L**
Pavona maldivensis	H	H	L	**L**
Pavona minuta	H	L	L	**L**
Pavona varians	H	L	L	**L**
Pavona venosa	H	L	L	**L**
Pectinia africanus	H	H	L	**L**
Pectinia alcicornis	H	L	L	**L**
Pectinia ayleni	H	L	L	**L**
Pectinia elongata	H	L	L	**L**
Pectinia lactuca	H	L	L	**L**
Pectinia maxima	H	L	H	**L**
Pectinia paeonia	H	L	L	**L**
Pectinia pygmaeus	H	L	H	**L**
Pectinia teres	H	L	H	**L**
Physogyra lichtensteini	H	H	L	**L**
Platygyra acuta	H	H	L	**L**
Platygyra carnosus	H	H	H	**H**
Platygyra contorta	H	H	L	**L**
Platygyra crosslandi	H	H	L	**L**
Platygyra daedalea	H	H	L	**L**
Platygyra lamellina	H	H	L	**L**
Platygyra pini	H	H	L	**L**
Platygyra ryukyuensis	H	H	L	**L**
Platygyra sinensis	H	H	L	**L**
Platygyra verweyi	H	H	L	**L**
Platygyra yaeyamaensis	H	H	H	**H**
Plerogyra discus	H	L	H	**L**
Plerogyra simplex	H	L	L	**L**
Plerogyra sinuosa	H	L	L	**L**
Plesiastrea devantieri	H	H	L	**L**
Plesiastrea versipora	H	H	L	**L**
Pocillopora ankeli	H	L	L	**L**
Pocillopora capitata	H	L	L	**L**
Pocillopora damicornis	H	L	L	**L**
Pocillopora danae	H	L	H	**L**
Pocillopora effusus	H	H	H	**H**
Pocillopora elegans	H	L	L	**L**
Pocillopora eydouxi	H	L	L	**L**
Pocillopora fungiformis	H	H	H	**H**
Pocillopora indiania	H	L	H	**L**
Pocillopora inflata	H	H	H	**H**
Pocillopora kelleheri	H	L	H	**L**
Pocillopora ligulata	H	L	L	**L**
Pocillopora meandrina	H	L	L	**L**
Pocillopora molokensis	H	H	H	**H**

Pocillopora verrucosa	H	L	L	**L**
Pocillopora woodjonesi	H	L	L	**L**
Pocillopora zelli	H	L	H	**L**
Podabacia crustacea	H	L	L	**L**
Podabacia motuporensis	H	L	L	**L**
Podabacia sinai	H	H	U	**L**
Polyphyllia novaehiberniae	H	H	L	**L**
Polyphyllia talpina	H	H	L	**L**
Porites annae	H	H	L	**L**
Porites aranetai	H	H	L	**L**
Porites arnaudi	H	H	L	**L**
Porites astreoides	H	H	H	**H**
Porites attenuata	H	L	L	**L**
Porites australiensis	H	H	L	**L**
Porites branneri	H	H	L	**L**
Porites brighami	H	H	H	**H**
Porites cocosensis	H	H	H	**H**
Porites colonensis	H	L	U	**L**
Porites columnaris	H	H	U	**L**
Porites compressa	H	H	H	**H**
Porites cumulatus	H	L	H	**L**
Porites cylindrica	H	L	L	**L**
Porites decasepta	H	H	U	**L**
Porites deformis	H	L	L	**L**
Porites densa	H	H	L	**L**
Porites desilveri	H	L	L	**L**
Porites divaricata	H	L	H	**L**
Porites echinulata	H	H	U	**L**
Porites eridani	H	L	H	**L**
Porites evermanni	H	H	L	**L**
Porites flavus	H	L	H	**L**
Porites furcata	H	L	H	**L**
Porites harrisoni	H	H	U	**L**
Porites heronensis	H	H	H	**H**
Porites horizontalata	H	L	L	**L**
Porites latistella	H	L	L	**L**
Porites lichen	H	L	L	**L**
Porites lobata	H	H	L	**L**
Porites lutea	H	H	L	**L**
Porites mayeri	H	H	H	**H**
Porites monticulosa	H	L	L	**L**
Porites murrayensis	H	H	L	**L**
Porites myrmidonensis	H	H	H	**H**
Porites napopora	H	L	H	**L**
Porites negrosensis	H	L	H	**L**

Porites nigrescens	H	L	L	**L**
Porites nodifera	H	H	U	**L**
Porites okinawensis	H	H	H	**H**
Porites ornata	H	L	H	**L**
Porites panamensis	H	H	H	**H**
Porites porites	H	L	H	**L**
Porites profundus	H	L	H	**L**
Porites pukoensis	H	H	H	**H**
Porites rugosa	H	L	H	**L**
Porites rus	H	H	L	**L**
Porites sillimaniana	H	L	H	**L**
Porites solida	H	H	L	**L**
Porites somaliensis	H	H	L	**L**
Porites stephensoni	H	H	L	**L**
Porites tuberculosa	H	L	H	**L**
Porites vaughani	H	L	L	**L**
Poritipora paliformis	H	H	H	**H**
Psammocora albopicta	H	H	L	**L**
Psammocora contigua	H	L	L	**L**
Psammocora decussata	H	H	H	**H**
Psammocora digitata	H	L	L	**L**
Psammocora explanulata	H	L	L	**L**
Psammocora haimeana	H	L	L	**L**
Psammocora nierstraszi	H	L	L	**L**
Psammocora obtusangula	H	L	H	**L**
Psammocora profundacella	H	L	L	**L**
Psammocora stellata	H	L	H	**L**
Psammocora superficialis	H	L	L	**L**
Psammocora vaughani	H	L	H	**L**
Psammocora verrilli	H	H	H	**H**
Pseudosiderastrea tayami	H	H	L	**L**
Sandalolitha dentata	H	H	L	**L**
Sandalolitha robusta	H	H	L	**L**
Scapophyllia cylindrica	H	L	L	**L**
Schizoculina africana	H	U	H	**L**
Schizoculina fissipara	H	U	H	**L**
Scolymia australis	H	H	H	**H**
Scolymia cubensis	H	H	H	**H**
Scolymia vitiensis	H	H	L	**L**
Seriatopora aculeata	H	L	H	**L**
Seriatopora caliendrum	H	L	L	**L**
Seriatopora dendritica	H	L	H	**L**
Seriatopora guttatus	H	L	H	**L**
Seriatopora hystrix	H	L	L	**L**
Seriatopora stellata	H	L	H	**L**

Siderastrea glynni	H	H	U	**L**
Siderastrea radians	H	H	H	**H**
Siderastrea savignyana	H	H	L	**L**
Siderastrea siderea	H	H	H	**H**
Siderastrea stellata	H	H	L	**L**
Simplastrea vesicularis	H	L	H	**L**
Solenastrea bournoni	H	H	H	**H**
Solenastrea hyades	H	H	H	**H**
Stephanocoenia michelinii	H	H	U	**L**
Stylaraea punctata	H	H	L	**L**
Stylocoeniella armata	H	L	L	**L**
Stylocoeniella cocosensis	H	L	H	**L**
Stylocoeniella guentheri	H	L	L	**L**
Stylophora danae	H	L	U	**L**
Stylophora kuehlmanni	H	H	U	**L**
Stylophora madagascarensis	H	H	H	**H**
Stylophora mamillata	H	H	U	**L**
Stylophora pistillata	H	L	L	**L**
Stylophora subseriata	H	L	H	**L**
Stylophora wellsi	H	H	U	**L**
Symphyllia agaricia	H	H	L	**L**
Symphyllia erythraea	H	H	U	**L**
Symphyllia hassi	H	H	L	**L**
Symphyllia radians	H	H	L	**L**
Symphyllia recta	H	H	L	**L**
Symphyllia valenciennesii	H	H	L	**L**
Symphyllia wilsoni	H	H	H	**H**
Trachyphyllia geoffroyi	H	H	L	**L**
Tubipora musica	H	L	L	**L**
Turbinaria bifrons	H	L	H	**L**
Turbinaria conspicua	H	L	H	**L**
Turbinaria frondens	H	L	L	**L**
Turbinaria heronensis	H	L	L	**L**
Turbinaria irregularis	H	L	H	**L**
Turbinaria mesenterina	H	L	L	**L**
Turbinaria patula	H	L	L	**L**
Turbinaria peltata	H	L	L	**L**
Turbinaria radicalis	H	L	L	**L**
Turbinaria reniformis	H	L	L	**L**
Turbinaria stellulata	H	L	L	**L**
Zoopilus echinatus	H	L	L	**L**
